# Comparative omics of CCM signaling complex (CSC)

**DOI:** 10.1186/s41016-019-0183-6

**Published:** 2020-01-15

**Authors:** Johnathan Abou-Fadel, Mark Smith, Kamran Falahati, Jun Zhang

**Affiliations:** Department of Molecular and Translational Medicine (MTM), Texas Tech University Health Science Center El Paso, 5001 El Paso Drive, El Paso, TX 79905 USA

**Keywords:** Cerebral cavernous malformation (CCM), CCM signaling complex (CSC), Angiogenesis, Proteomics, Transcriptomics, Interactome, Omics, Comparative omics, Biomarkers, Genetic modifiers, Systems biology

## Abstract

**Background:**

Cerebral cavernous malformations (CCMs), a major neurosurgical condition, characterized by abnormally dilated intracranial capillaries, result in increased susceptibility to stroke. KRIT1 (CCM1), MGC4607 (CCM2), and PDCD10 (CCM3) have been identified as causes of CCMs in which at least one of them is disrupted in most familial cases. Our goal is to identify potential biomarkers and genetic modifiers of CCMs, using a global comparative omics approach across several in vitro studies and multiple in vivo animal models. We hypothesize that through analysis of the CSC utilizing various omics, we can identify potential biomarkers and genetic modifiers, by systemically evaluating effectors and binding partners of the CSC as well as second layer interactors.

**Methods:**

We utilize a comparative omics approach analyzing multiple CCMs deficient animal models across nine independent studies at the genomic, transcriptomic, and proteomic levels to dissect alterations in various signaling cascades*.*

**Results:**

Our analysis revealed a large set of genes that were validated across multiple independent studies, suggesting an important role for these identified genes in CCM pathogenesis.

**Conclusion:**

This is currently one of the largest comparative omics analysis of CCM deficiencies across multiple models, allowing us to investigate global alterations among multiple signaling cascades involved in both angiogenic and non-angiogenic events and to also identify potential biomarker candidates of CCMs, which can be used for new therapeutic strategies.

## Background

Cerebral cavernous malformations (CCMs) are intracranial capillary lesions in the brain, leading to focal neurological defects and increased susceptibility to hemorrhagic strokes [1]. CCM lesions are mainly found in the central nervous system (CNS), skin, and liver. Clinically, CCMs in the brain are diagnosed through magnetic resonance imaging (MRI) and have characteristic densely packed microvessels and deficient interstitial brain parenchyma [2]. Effective treatment for patients suffering from CCMs is limited to surgical excision, while other options such as antiepileptic drugs have very limited role for seizure suppression [3].

Three CCM genes KRIT1 (CCM1), MGC4607 (CCM2), and PDCD10 (CCM3) are known causes for familial forms of the disease, which accounts for about half of CCM patients; interestingly, the etiology of the remaining half of CCM cases are still unknown [3–5]. Furthermore, the Hispanic population has a higher percentage of having clinical symptoms associated with CCMs compared to non-Hispanic populations [3, 6]. These evidences support the notion that there are apparently unidentified genetic modifiers or even environmental factors that perhaps play major roles in the pathogenesis of CCMs.

The CCM signaling complex (CSC), composed of all three CCM genes, interacts with numerous proteins and has affinity for a wide range of ligands, contributing to cellular processes including numerous angiogenic signaling cascades, adhesion, cell migration, and apoptotic pathways [7–11]. CCM proteins have been shown to be essential for angiogenic processes including new blood vessel development [12–15], blood vessel stability [16], vascular angiogenesis [9, 17], cell adhesion to extracellular matrix, tube formation, and endothelial cell morphogenesis [14, 18].

System-wide evaluation of CCM deficiencies across multiple animal models allow us to investigate signaling cascades involved in pathogenesis of CCMs. Omics integrates in-depth analysis of alterations at the molecular level to elucidate observable phenotypes resulting from knockdown/knockout models across multiple species. Comparative omics analysis provides the possibility for discovery of novel biomarkers that can assess risk of treatment, help predict response to treatment, and aid in drug screening, determination of differential diagnosis and prognosis, and monitoring of progression of CCMs. We hypothesize that through analysis of the CSC utilizing various omics, we can identify potential biomarkers and genetic modifiers, by systemically evaluating effectors and binding partners of the CSC as well as second layer interactors.

**Fig. 1:**
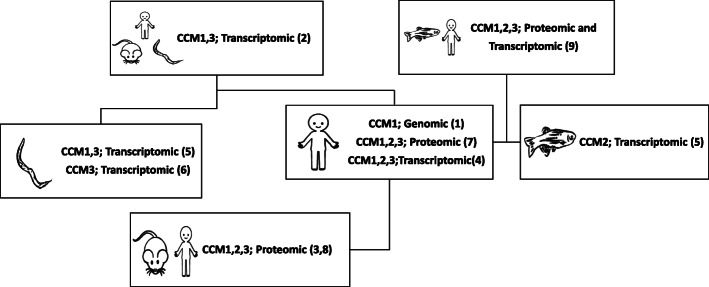
Flow diagram illustrating organization of nine cohorts to assemble comparative database. Diagram illustrates hierarchy organizational process used to siphon and filter data from nine independent omics studies to assemble the comparative database foundation used in this study. Information provided within each box include the model evaluated in the identified cohort, background CCM mutation(s) being assessed, omics approach used, and the associated cohort information in parentheses corresponding to numbers found in Additional file 11

To our knowledge, this is the first comparative omics attempt in systems biology, which analyzes CCM-deficient animal models, in nine independent studies, across multiple species at the genomic, transcriptomic, and proteomic levels (Fig. 1). Our analysis revealed a large set of genes that were validated across multiple independent studies, suggesting an important role for these identified genes in CCM pathogenesis. Alterations in various signaling cascades among validated genes revealed angiogenic and non-angiogenic pathways including hemidesmosome assembly, integrin complex and mediated signaling pathways, adherens junctions, focal adhesion, cell adhesion, cellular morphology, and regulation of cell cycle cascades. Our goal is to identify potential biomarkers and genetic modifiers of CCMs, using a global comparative omics approach across several in vitro studies and multiple in vivo animal models. This comparative omics analysis of CCM deficiencies allow us to investigate global alterations among multiple signaling cascades involved in both angiogenic and non-angiogenic events and to also identify potential biomarker candidates of CCMs, which can be used for new therapeutic strategies.

## Materials and methods

### Data acquisition

A comparative database in excel (Additional files 1 and 2) was set up for the nine total available CCM cohorts that were analyzed for our comparative omics studies [8, 10, 19–25] (Fig. 1). To do this, gene names from these nine studies were gathered from both published figures as well as supplemental figures and tables and input into excel to create a database that could be used to identify overlaps between cohorts (Additional files 1 and 2). Gene names were used regardless of detection method used in cohort, to assure overlapped genes were identified due to variations between gene name and protein names for several candidates. Once combined, the data was organized and assembled based off detection method, background strain (i.e., mouse BMEC *Ccm1*/Krit1 ECKO) and organism (Additional file 3).

### Data analysis

Once the comparative database was assembled, a script was created within excel to identify shared differentially expressed genes between cohorts. These were annotated in a new Excel sheet, and simple set operations (used to find duplicate gene names) were used to find the number of overlaps for quantitative purposes. Identified overlaps were compared between papers and overlaps within the same study were not considered “validated”; only overlaps within two or more different studies were considered “validated.” The validated genes were then compiled into 2, 3, and 4 validation groups which were further assessed by detection method, background strain (i.e., mouse BMEC *Ccm1*/Krit1 ECKO) and organism (Additional file 3). Details provided in this table include the protein, details of detection method, strain and organism, and a list of the corresponding literature (Additional file 11) the data was extracted from. Genomic validation methods involved detection through RT-PCR or other DNA detection methods, while proteomic methods involved pull-down assays and/or subsequent mass spectrometry applications; transcriptomics methods involved identification of genes through various RNA detection methods.

The identified overlapped genes (for 2, 3, and 4 validations separately) were then input into the Search Tool for the Retrieval of Interacting Genes/Proteins database (STRING) and using external links extracted to generate higher resolution interactome figures using Cytoscape. The output from STRING database with query proteins only (“2 validations,” Additional file 4), or query and ten interactor(s) for smaller sets of validated genes (“3 and 4 validations,” Additional files 7 and 9 respectively) were generated. Enrichment data including KEGG pathways, GO cellular components, GO molecular functions, and GO biological processes were also extracted that were used to analyze signaling pathways involved (Additional files 5, 8, and 10). Pathways of interest relating to the CSC involving altered genes for two, three, and four validations were assembled to provide summary figure (Additional file 6). All CCM studies used in this comparative omics analysis (Additional file 11) are listed with corresponding reference numbers used in Additional files 1, 2, and 3 and in Fig. 1.

## Results

### Genes validated in two independent studies reveals importance of CCM signaling complex (CSC) during angiogenesis

**Fig. 2:**
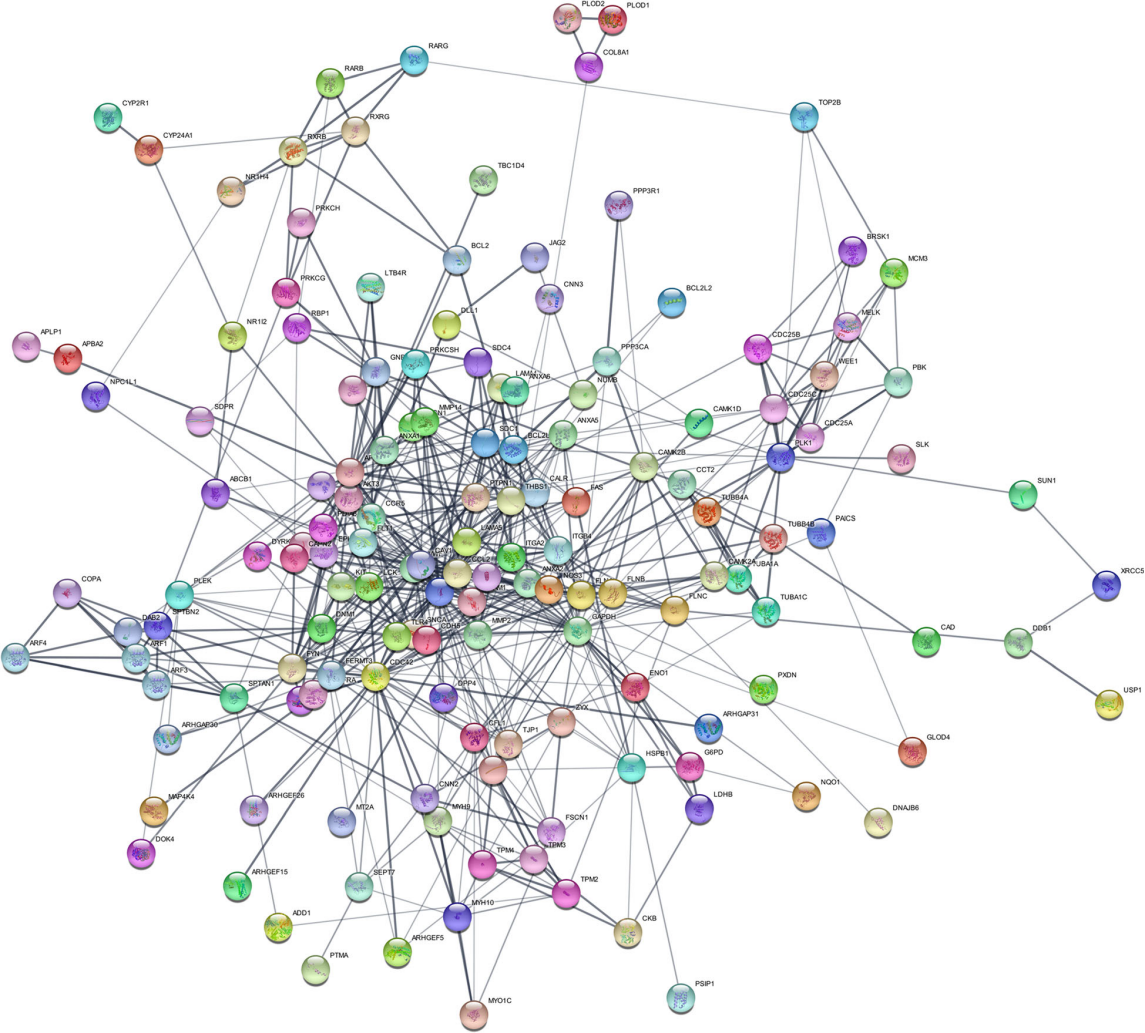
Interactome of altered genes in CCM-deficient models with two validations. A total of 152 genes (nodes) were matched and identified as perturbed in two different CCM studies. For datasets, non-human genes were converted to human homologs before processing and all genes capitalized to ensure overlaps were not misidentified due to case changes. Interactomes were constructed using Cytoscape software equipped with STRING application. Interactomes illustrate affected pathways with only query proteins being incorporated into the interactome with a false discovery rate (FDR) set to 0.4 due to the large number of query proteins. No post modifications were made to the layout of the interactome once generated in STRING and exported to Cytoscape. Crystal structures, if available, for identified proteins are shown within the corresponding nodes. Non-interacting nodes were removed from figure

**Fig. 3:**
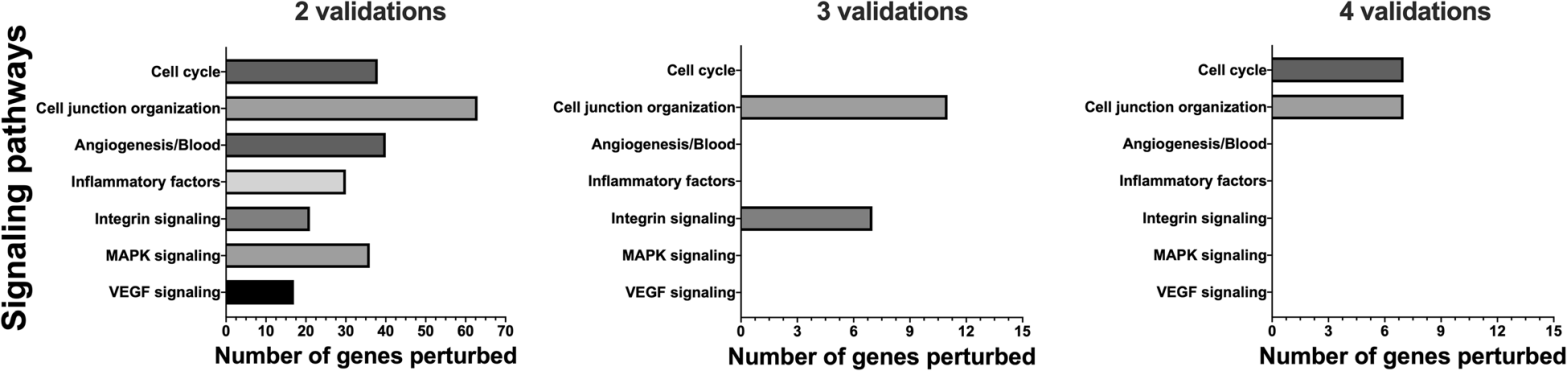
Summarized signaling pathways and number of genes involved in cohort comparisons. Pathways impacted in various models with perturbed CSC signaling for two, three, and four validations. Bar graphs demonstrate the number of perturbed genes found in each of the major CSC associated signaling pathways. Genes that are involved in more than one signaling pathway were counted and added to each pathway. Missing bars indicate a lack of perturbed genes (for either proteomics or transcriptomics) for that particular pathway in this meta-analysis study

Our comparative omics analysis revealed a large subset of genes (152) that were altered in two of the nine evaluated independent studies (Fig. 2 and Additional file 4). This large group of potential CSC-associated query genes allowed for the generation of the complex interactome demonstrated in Fig. 2 without the need of additional interactors to be included through the STRING database. This complex interactome revealed a large subset of genes involved in multiple signaling cascades that was further dissected using the enrichment data provided through the STRING database (Additional file 5). When analyzing signaling pathways involved within this interactome, we observed eight various angiogenic signaling pathways that involved our queries, further supporting our previous notion that the CSC plays an essential role in angiogenesis (Table 1). Intriguingly, regulation of sprouting angiogenesis pathways was dominant among the angiogenic pathways identified, validating our previous data in zebrafish CCM-deficient models [16, 18]. Out of the 152 genes analyzed, 40 of them were involved in angiogenesis signaling cascades, which was the second largest signaling pathway affected with a perturbed CSC (Fig. 3, left panel).

**Table 1:** Altered genes in CCM-deficient models with two validations having documented roles in angiogenic pathways. An enrichment category was exported along with Fig. 1 that detailed altered pathways involved with the identified 152 genes. Among the exported pathways, a filter search was applied to identify all enrichment data involved with angiogenesis specifically. Information provided in the table includes the term id, number of enriched genes in each enrichment category, description of the category, genes specifically involved, false discovery rate (FDR) value, and the term name for each category (which includes GO terms if applicable)

Term id	Enriched genes	Category	Description	Enriched genes	FDR value	Term name
94	19	GO Process	Angiogenesis	MYH9|MMP2|CCL2|PPP3R1|LAMA5|PDGFRA|THBS1|COL8A1|RORA|FLT1|NOS3|MMP14|CAV1|ANXA2|FN1|EPHA2|DLL1|MMRN2|CLIC4	1.18E−09	GO.0001525
610	5	GO Process	Regulation of cell migration involved in sprouting angiogenesis	MAP2K5|THBS1|AKT3|MMRN2|ANXA1	3.20E−04	GO.0090049
616	6	GO Process	Regulation of sprouting angiogenesis	MAP2K5|THBS1|AKT3|DLL1|MMRN2|ANXA1	3.30E−04	GO.1903670
631	11	GO Process	Regulation of angiogenesis	MAP2K5|HSPB1|THBS1|AKT3|FLT1|NOS3|CDH5|EPHA2|DLL1|MMRN2|ANXA1	3.80E−04	GO.0045765
716	8	GO Process	Positive regulation of angiogenesis	HSPB1|THBS1|AKT3|FLT1|NOS3|CDH5|DLL1|ANXA1	9.90E−04	GO.0045766
910	3	GO Process	Negative regulation of cell migration involved in sprouting angiogenesis	MAP2K5|THBS1|MMRN2	0.0034	GO.0090051
1485	3	GO Process	Positive regulation of sprouting angiogenesis	AKT3|DLL1|ANXA1	0.0226	GO.1903672
1646	3	GO Process	Sprouting angiogenesis	THBS1|DLL1|MMRN2	0.0329	GO.0002040
152	12	Reference publications	(2014) PPARGamma activation but not PPARGamma haplodeficiency affects proangiogenic potential of endothelial cells and bone marrow-derived progenitors.	MMP2|THBS1|VWF|NGEF|ICAM1|FLT1|KIT|NOS3|CCT2|MMP14|CAV1|FN1	2.31E−08	PMID.25361524
275	9	Reference publications	(2017) Talin Modulation by a Synthetic N-Acylurea Derivative Reduces Angiogenesis in Human Endothelial Cells.	GAPDH|VWF|ICAM1|FLT1|ANXA5|NOS3|CDH5|FN1|TLN2	1.34E−06	PMID.28117756
346	10	Reference publications	(2017) Platelets and cancer angiogenesis nexus.	MMP2|THBS1|VWF|ICAM1|FLT1|KIT|ITGA2|NOS3|MMP14|FN1	3.18E−06	PMID.28681240
352	8	Reference publications	(2010) PPARalpha is essential for microparticle-induced differentiation of mouse bone marrow-derived endothelial progenitor cells and angiogenesis.	THBS1|ICAM1|FLT1|CSF1R|ANXA5|NOS3|CDH5|FN1	3.51E−06	PMID.20811625
1596	5	UniProt Keywords	Angiogenesis	MMP2|COL8A1|FLT1|EPHA2|MMRN2	0.0297	KW-0037

### Genes validated in two independent studies also reveal importance of other various signaling pathways associated with altered CSC

The large set of genes identified within this cohort also demonstrated impacts in other signaling cascades which included regulation of vascular endothelial growth factor (VEGF) pathways, regulation of various mitogen-activated protein kinase signaling cascades (MAPK), various binding and regulation of integrin signaling pathways, inflammatory responses, numerous cell cycle, and cell junction organization pathways (Fig. 3, left panel), all of which were also identified in our previous multi-omics analysis of CCM mutants across multiple models [26]. Intriguingly, the largest signaling pathway affected was cell junction organization through numerous focal adhesion as well as gap, tight, adherens, and cell-cell junctions’ pathways (Additional file 6), which is consistently also the largest group in the 3 and 4 validation groups (Fig. 3).

### Genes validated in three independent studies emphasizes the promiscuous roles of the CSC in non-angiogenic signaling pathways

**Fig. 4:**
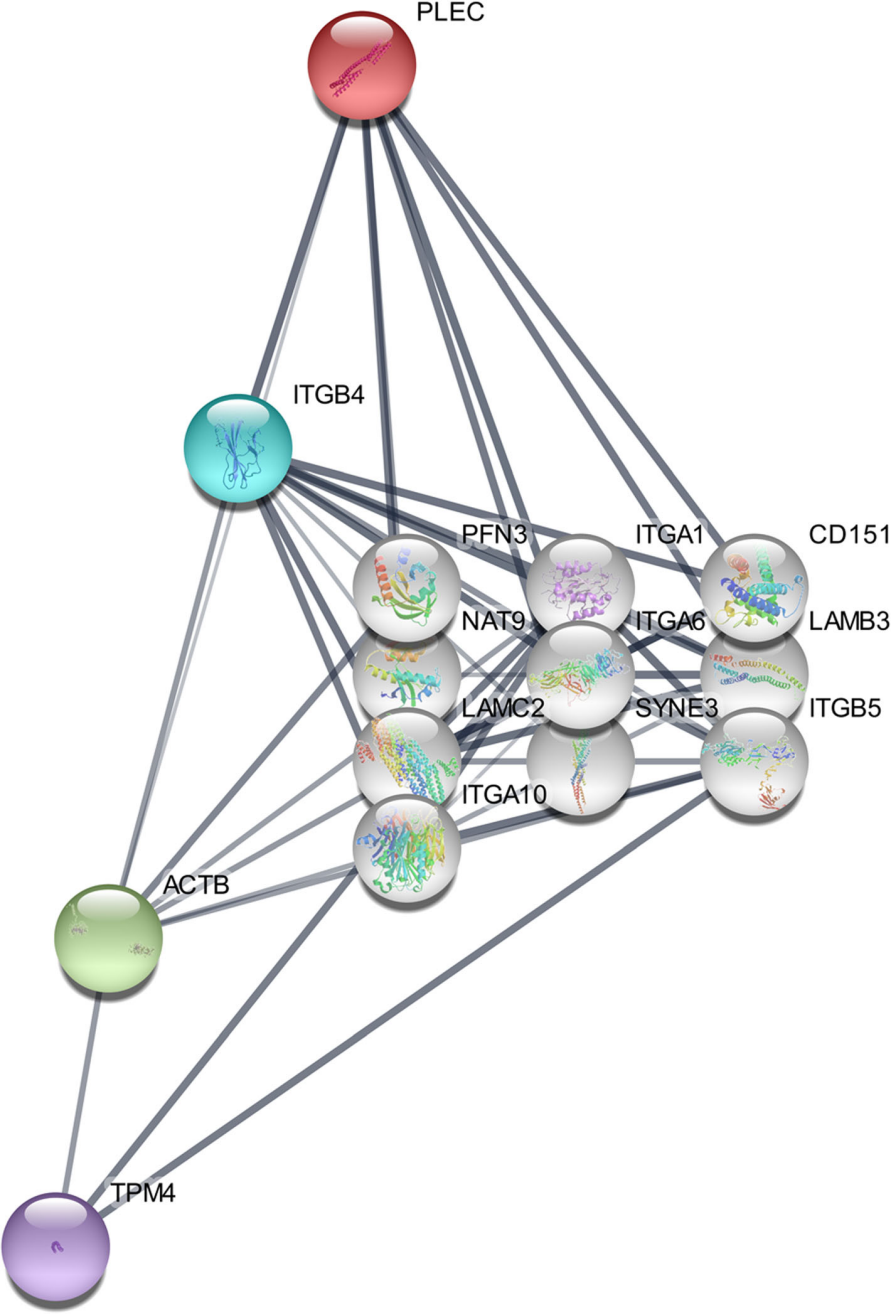
Interactome of altered genes in CCM-deficient models with three validations. A total of five genes (nodes) were matched that were identified as perturbed in three different CCM studies. For datasets, non-human genes were converted to human homologs before processing and all genes capitalized to ensure overlaps were not misidentified due to case changes. Interactomes were constructed using Cytoscape software equipped with STRING application. Interactomes illustrate affected pathways with query proteins and ten interactors being incorporated into the interactome with a false discovery rate (FDR) set to 0.4 due to the smaller number of query proteins. No post modifications were made to the layout of the interactome once generated in STRING and exported to Cytoscape. Crystal structures, if available, for identified proteins are shown within the corresponding nodes

Further analysis revealed a smaller subset of genes (4) that were altered in three independent studies (Fig. 4 and Additional file 7). This smaller group of potential CSC-associated query proteins allowed for the generation of the less complex interactome demonstrated in Fig. 4 with ten additional interactors included through the STRING database with a selectivity cutoff score of 0.5. This smaller interactome still revealed a large group of signaling cascades that were further dissected using the enrichment data provided through the STRING database (Additional file 8). Interestingly, our analysis did not reveal alterations in angiogenic pathways with our query results, but did reveal other pathways impacted including hemidesmosome assembly, integrin complex, integrin-mediated signaling pathways, adherens junctions, focal adhesion, and cell adhesion pathways (Fig. 3, middle panel), also validating our systems biology study, analyzing CCM-deficient models in the human brain microvascular endothelial cells (HBMVEC) in vitro and zebrafish lines in vivo [26].

### Gene validated in four independent studies reveals potential role of the CSC in cell junction organization and regulation of cell cycle

**Fig. 5:**
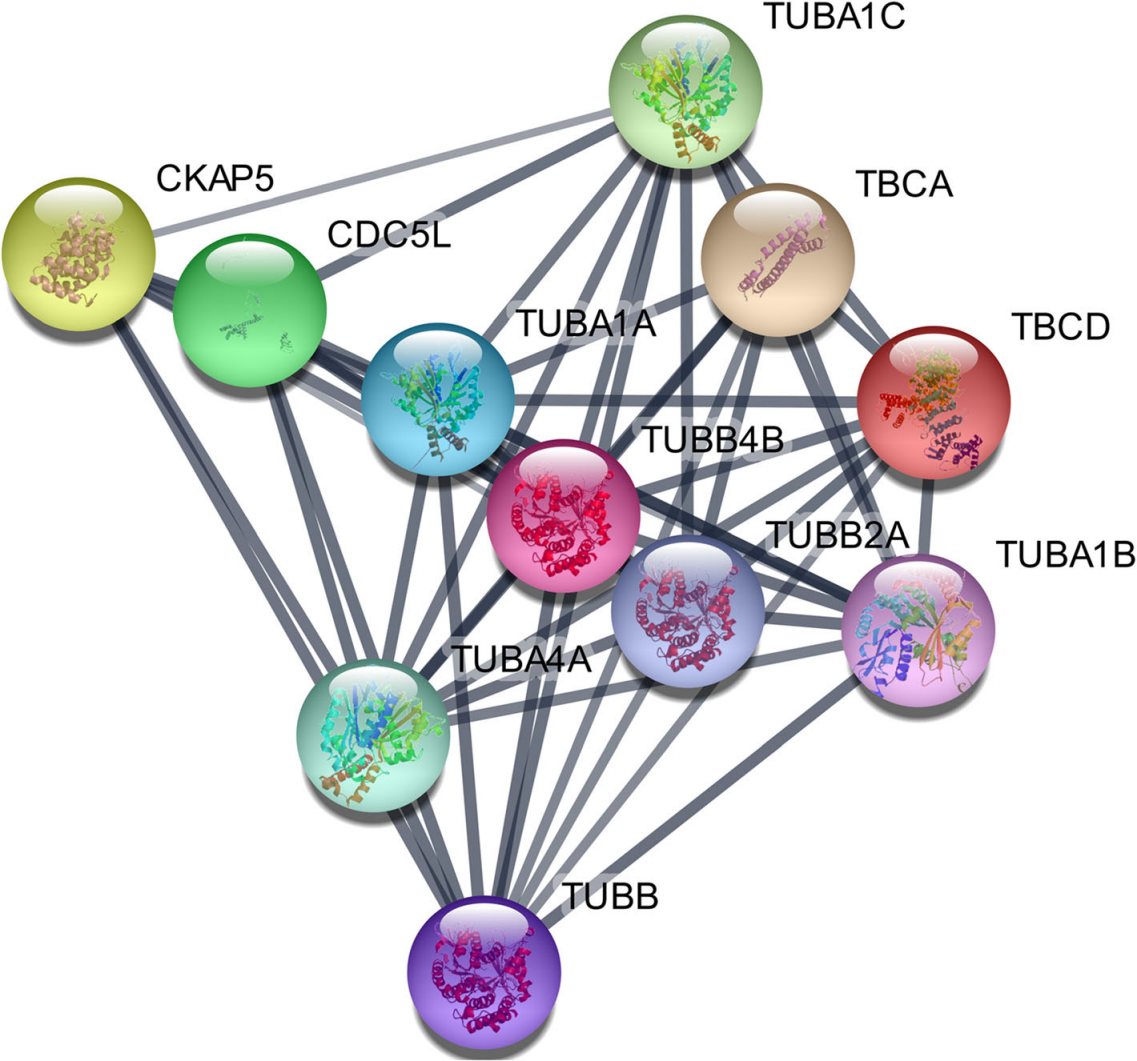
Interactome of altered genes in CCM-deficient models with four validations. Only one gene (node) was matched that was identified as perturbed in four different CCM studies. For datasets, non-human genes were converted to human homologs before processing and all genes capitalized to ensure overlaps were not misidentified due to case changes. Interactomes were constructed using Cytoscape software equipped with STRING application. Interactomes illustrate affected pathways with query protein and ten interactors being incorporated into the interactome with a false discovery rate (FDR) set to 0.4 due to the smaller number of query proteins. No post modifications were made to the layout of the interactome once generated in STRING and exported to Cytoscape. Crystal structures, if available, for identified proteins are shown within the corresponding nodes

Only one gene was identified that was validated across four different independent studies (Fig. 5 and Additional file 9). In order to allow for the generation of the interactome involved with our one identified gene, an additional ten interactors were included through the STRING database with a selectivity cutoff score of 0.5. The generated interactome still revealed a large group of signaling cascades that were further dissected using the enrichment data provided through the STRING database (Additional file 10). Interestingly, the pathways analysis still revealed more than 40 GO pathways affected, even with our limited interactome data. The majority of the identified pathways included cell junction pathways such as cytoskeleton and tubulin processes as well as regulation of cell cycle signaling pathways (Fig. 3, right panel). This data further supports the promiscuous role of the CSC in non-angiogenic signaling cascades, including essential roles of the CSC in cell junction/integrin/cell cycle signaling pathways as previously described (Fig. 3 and Additional file 6).

## Discussion

### Genes validated in two independent studies display dominant roles in angiogenesis

It has been demonstrated that the three CCM proteins form the CSC, which plays an essential role in the pathogenesis of CCMs when disrupted [26]. Efforts to dissect CSC signaling cascades have been made (Additional file 11) [8, 10, 20–26], but with some contradicting outcomes. It is essential to establish comparative omics methods to assess similarities among those independent studies, which can aid in identification of potential biomarker candidates for prognostic and diagnostic applications. In this novel report, we utilized multiple omics approaches to integrate all current studies in various CCMs models. We identified 152 overlapped genes that were altered in two independent studies (Fig. 2 and Additional file 4). Interestingly, the interactome generated with these proteins not only displayed the most amount of angiogenic changes but also displayed other non-angiogenic changes in signaling cascades (Additional file 5). In total, we observed potential alterations to eight major angiogenic signaling pathways as well as various signaling cascades associated with angiogenesis (VEGF, MAPK integrin and cell junction signaling, etc.), further supporting our previous notion that the CSC plays an essential role in angiogenesis (Table 1). Intriguingly, regulation of sprouting angiogenesis pathways was dominant among the angiogenic pathways identified, validating our previous data in zebrafish Ccm-deficient models [16, 18]. Sprouting, an initial step of angiogenesis, is essential to provide new blood vessels to satisfy metabolic requirements of neurovascular endothelial cells. In concert with our previous studies [26], we also identified cytoskeleton remodeling, cell-cell adherens junctions, and tubulin binding signaling cascades largely affected with these 152 identified targets (Additional file 5).

### Genes validated in more than two independent studies display dominant role of CSC in cell-cell adhesion pathways

Interestingly, when we evaluated overlapped genes in more than two independent studies, our analysis did not reveal alterations in angiogenic pathways with our query results, but did reveal other pathways impacted including hemidesmosome assembly, integrin complex and mediated signaling pathways, adherens junctions, focal adhesion, and cell adhesion pathways (Figs. 4 and 5 and Additional files 7, 8, 9, and 10), further validating our previous systems biology study [26]. Two genes identified in this cohort included integrin β4 (ITGB4) and its docking partner Plectin (PLEC), which are both key components involved in hemidesmosome assembly [27]. ITGB4 signaling pathways are known to play an essential role in the integrity of the blood-brain barrier (BBB) and hypothesized to be a negative regulator of angiogenesis at quiescent state [28]. Pathways associated with ITGB4 include β-adrenergic signaling pathways and BBB [29, 30] and was recently reported that upregulation of ITGB4 in cerebrovascular endothelial cells is associated with neuroinflammatory events [31–33], elucidating a possible role of ITGB4 in connection between neuroinflammation and disruption of the CSC. Finally, analysis of tubulin, beta 4B class IVb (TUBB4B), revealed more than 40 GO pathways affected, even with only one gene being identified across four independent studies. The majority of the identified pathways included cell morphology pathways such as cytoskeleton and tubulin processes, as well as regulation of cell cycle signaling pathways. Further evaluation of expression patterns of TUBB4B, among the four independent studies, displayed no contradicting trends of expression, demonstrating high confidence in identification of altered genes and impacted pathways using omics approaches in various models. This data further supports the promiscuous role of the CSC in non-angiogenic signaling cascades as previously described (Fig. 4 and Additional file 8) [26].

### Analysis of heterogeneity among the nine omics studies used for meta-analysis

It is important to assess the heterogeneity of the various animal models evaluated in this comparative study, as well as assessing the differences in methods of detection for proper interpretation of results. At first, we were surprised by the low number of overlapped genes seen in more than two cohorts, especially when constructing our pathways analysis. A likely possibility of not obtaining more identified overlapped genes, especially those relating to angiogenic signaling pathways, could result from the heterogeneity of the four different models used in this meta-analysis. To help understand the variation of impacted signaling pathways resulting from altered CCM expression in various models, a brief look at the evolutionary relationship of CCM proteins among animal models, particularly *C*. *elegans*, which displays the lowest conservation of CCM1 and CCM3 with a similarity score of 19.3% and 56% respectively [34], helps elucidate one possibility for these discrepancies. Another consideration includes method of detection, with some genes being detected using genomic and transcriptomic approaches (higher sensitivity to gene alterations) while others were only detected using proteomic approaches. By comparing both transcriptomic and proteomic data, it is frequently common that messenger RNA levels do not always correlate with their coded protein levels at steady state. We suspect there is various regulation mechanisms existing at both RNA and protein levels for the CSC, especially among the four animal models used. Secondly, RNA-seq transcriptome profiling methods are unable to detect the expressional adjustment by post-transcriptional mechanisms [35–37]. Third, the existence of major post-transcriptional [38, 39] and post-translational mechanisms, such as protein synthesis and protein degradation control pathways, separates RNA expression and protein abundancy at static state [40]. Finally, these lacks of overlapped genes might also indicate a form of feedback inhibition on transcriptional regulation based on protein abundance at static state, as we previously described [26].

## Conclusion

This is one of the largest comparative omics analyses of CCM deficiencies across multiple models in systems biology, which analyzes CCMs deficient animal models, across multiple species at the genomic, transcriptomic, and proteomic levels*.* Our analysis revealed a large set of genes that were validated across nine independent studies, suggesting an important role for these identified genes in CCM pathogenesis. Associated signaling cascades among validated genes revealed angiogenic and non-angiogenic pathways including hemidesmosome assembly, integrin complex and mediated signaling pathways, adherens junctions, cellular morphology, and regulation of cell cycle cascades (Fig. 3). This comparative omics analysis of CCM deficiencies allow us to investigate global alterations among multiple signaling cascades involved in both angiogenic and non-angiogenic events. This study has identified inconsistencies among various omics studies when utilizing multiple animal models, which must be evaluated and addressed regarding the essential role of the CSC. Furthermore, our efforts have built a basic framework for CSC signaling cascades, which will allow for further evaluation and validation of potential biomarker candidates of CCMs identified across various models, in hopes of identifying potential biomarker candidates of CCMs for new diagnostic and therapeutic strategies.

## Supplementary information

**Additional file 1: Table S1A.** Identification of altered genes in CCM models with various validations. A total of 9 CCM studies were analyzed to identify genes that overlapped as perturbed in various CCM models. For Datasets, non-human genes were converted to human homologs before processing and all genes capitalized to ensure overlaps were not misidentified due to case changes. Studies are divided into columns based on detection method, background strain (i.e. mouse BMEC *Ccm1*/Krit1 ECKO) and organism. Genes that are italicized and bolded are genes duplicated in another column. Overlaps within the same study (EX: First 6 columns) were not considered “validated”, only overlaps within two different studies are considered "validated". Abbreviations: Differentially expressed genes (DEG's), human umbilical vein endothelial cells (HUVEC), Human Brain Microvascular Endothelial Cells (HBMVEC). Reference numbering can be found in Additional file 11.

**Additional file 2: Table S1B.** Summary of Identified altered genes in CCM models with various validations. A summary of "validated genes" across 9 CCM studies were analyzed to identify genes that overlapped as perturbed in various CCM models. Studies are divided into columns based on detection method, background strain (i.e. mouse BMEC *Ccm1*/Krit1 ECKO) and organism. Genes that are bolded are genes duplicated in two studies, genes italicized are duplicated in three studies, and gene underlined is duplicated in 4 studies. The largest cohort (First 6 columns in supplemental table 1A) are not included in this table as they have significant overlaps within each column, complicating the summarized data, but were counted as "validated" if gene(s) was found in another cohort (EX: TUBB4B was identified in 3 cohorts, shown here in this table, as well as the largest cohort (not shown in this table) therefore making it validated across 4 studies). Abbreviations: Differentially expressed genes (DEG's), human umbilical vein endothelial cells (HUVEC), Human Brain Microvascular Endothelial Cells (HBMVEC). Reference numbering can be found in Additional file 11.

**Additional file 3: Table S2.** Details of identified altered genes in CCM models with various validations. Genes validated were compiled into **A)** 2 validations, **B)** 3 validations and **C)** 4 validation groups. Details included here include additional sorting of data by detection method, background strain (i.e. mouse BMEC *Ccm1*/Krit1 ECKO) and organism. Details provided include the protein, details of detection method, strain and organism, and a list of the corresponding literature the data was extracted from. For a list of the references and corresponding numbers used, please reference supplemental table 7. Genomic methods involved detection through RT-PCR or other DNA detection methods, proteomics methods involved pull-down assays and subsequent mass spectrometry applications while transcriptomics methods involved identification of genes through various RNA detection methods. Abbreviations: NVU: Neuro Vascular unit from surgically resected lesions.

**Additional file 4: Table S3A.** Detailed description of altered genes in CCM models with 2 validations. A total of 152 genes were analyzed that overlapped in two different CCM studies. Details provided for each protein include mechanisms associated with each, functions, binding partners, motifs and domains. Protein details were extracted from STRING enrichment data after construction of Figure 1 Interactome.

**Additional file 5: Table S3B.** Altered genes in CCM models with 2 validations enrichment data. An enrichment category was exported along with Fig. 1 that detailed altered pathways involved with the identified 152 genes. Information provided in the table includes the number of enriched genes in each enrichment category, description of the category, genes specifically involved, FDR value, and the term name for each category (which includes GO terms if applicable). Among the exported pathways identified in this group, a filter search was applied to identify all enrichment data involved with angiogenesis specifically which are bolded and re-summarized in Table 1.

**Additional file 6: Table S4.** Altered genes in CSC signaling pathways assessed with various validations. An enrichment category was exported that detailed altered pathways involved with the identified genes with **A)** 2, **B)** 3 or **C)** 4 validations. Among the exported pathways, a filter search was applied to identify all enrichment data involved with VEGF, MAPK, Integrin, Inflammatory, Angiogenesis, Blood, cell junction organization and cell cycle signaling pathways. Information provided in the table includes the number of enriched genes in each enrichment category, description of the category, genes specifically involved, False Discovery Rate (FDR) value, and the term name for each category (which includes GO terms if applicable). Duplicated genes within each pathway sub-categories were only counted once towards each signaling pathway to construct (Fig. 3).

**Additional file 7: Table S5A.** Detailed description of altered genes in CCM models with 3 validations. A total of 4 genes were analyzed that overlapped in three different CCM studies. Details provided for each protein include mechanisms associated with each, functions, binding partners, motifs and domains. Protein details were extracted from STRING enrichment data after construction of Fig. 4 Interactome. Proteins in bold are the 4 validated proteins, while other proteins are the 10 interactors added to interactome.

**Additional file 8: Table S5B.** Altered genes in CCM models with 3 validations enrichment data. An enrichment category was exported along with Figure 4 that detailed altered pathways involved with the identified 4 genes and 10 interactors. Information provided in the table includes the number of enriched genes in each enrichment category, description of the category, genes specifically involved, FDR value, and the term name for each category (which includes GO terms if applicable).

**Additional file 9: Table S6A.** Detailed description of altered gene in CCM models with 4 validations. 1 gene was analyzed that overlapped in four different CCM studies. Details provided for identified protein include mechanisms associated with each, functions, binding partners, motifs and domains. Protein details were extracted from STRING enrichment data after construction of Figure 5 Interactome. Protein in bold is the 1 validated protein, while other proteins are the 10 interactors added to interactome.

**Additional file 10: Table S6B.** Altered gene in CCM models with 4 validations enrichment data. An enrichment category was exported along with Figure 5 that detailed altered pathways involved with the identified 1 gene and 10 interactors. Information provided in the table includes the number of enriched genes in each enrichment category, description of the category, genes specifically involved, FDR value, and the term name for each category (which includes GO terms if applicable).

**Additional file 11: Table S7.** 9 CCM studies used for comparative omics analysis. A list of 9 CCM studies used for the comparative omics analysis are detailed. Corresponding numbers used for each reference are used in supplemental tables 1A, 1B and 2 and in Fig. 1.

## Data Availability

Please contact author for data requests.

## Abbreviations

CCM: Cerebral cavernous malformation
CSC: CCM signaling complex
CNS: Central nervous system
FDR: False discovery rate
STRING: Search Tool for the Retrieval of Interacting Genes/Proteins database
