# Supplementary material for: Comparative omics of CCM signaling complex (CSC)
Source: Chin Neurosurg J. 2020 Jan 15;6:4. doi: 10.1186/s41016-019-0183-6 (PMC7398211; doi:10.1186/s41016-019-0183-6)
Supplement: Supplementary file 1 — Additional file 1: Table S1A. Identification of altered genes in CCM models with various validations. A total of 9 CCM studies were analyzed to identify genes that overlapped as perturbed in various CCM models. For Datasets, non-human genes were converted to human homologs before processing and all genes capitalized to ensure overlaps were not misidentified due to case changes. Studies are divided into columns based on detection method, background strain (i.e. mouse BMEC Ccm1/Krit1 ECKO) and organism. Genes that are italicized and bolded are genes duplicated in another column. Overlaps within the same study (EX: First 6 columns) were not considered “validated”, only overlaps within two different studies are considered "validated". Abbreviations: Differentially expressed genes (DEG's), human umbilical vein endothelial cells (HUVEC), Human Brain Microvascular Endothelial Cells (HBMVEC). Reference numbering can be found in Additional file 11. [file 41016_2019_183_MOESM1_ESM.pdf]

**Identification of altered genes in CCM models with various validations.**

| Important genes with document roles in ccm Disease (2) | Differentially expressed genes (DEGs) in human (2) | DEGs in mouse BMEC Ccm1/Krit1ECKO (2) | DEGs in mouse BMEC Ccm3/Pdcd10ECKO (2) | DEGs common across mouse BMECs and *C.elegans* only in Ccm1/Krit1 mutant models (2) | DEGs common across mouse BMECs and *C.elegans* only in Ccm3/Pdcd10 mutant models (2) | CCM1 delimits relationship of PTB/Ph domains (1) | proteomic analysis of cerebral cavernous malformation in HUVEC's (7) | Biomechanics of endothelial tubule formation differentially modulated by cerebral cavernous malformation proteins (4) | Target: (*H.sapiens* ) FROM zebrafish De pick target proteins (5) | Target: (*H.sapiens* ) FROM *C.Elegans* De pick target proteins (5) | Proteomic identification of the CSC (8) | Proteomic analysis of Krit1 Loss-of Function (3) | CCM3 Gene Network (6) | ccm1 proteomics in HBMVEC's (9) | CCM2 PROTEOMICS in HBMVEC's (9) | CCM3 PROTEOMICS in HBMVEC's (9) | ccm1 RNA in zebrafish (9) | CCM2 RNA in zebrafish (9) | CCM3 RNA in zebrafish (9) |
|---|---|---|---|---|---|---|---|---|---|---|---|---|---|---|---|---|---|---|---|
| ***CALM1*** | A2ML1 | 1190002N15RIK | 1110065P20RIK | ***AAMP*** | ***ABCB1*** | ***DAB2*** | ***SEPT7*** | ***ADD1*** | ***ABCB1*** | ***AKT3*** | | ***Actb*** | ***APP*** | ***CDH5*** | ***CDH5*** | STK24 | ***APBA2*** | ***CCM2*** | HMOX2 |
| ***CALM3*** | AADACL3 | ***2210013O21RIK*** | ***2210013O21RIK*** | ***AARS*** | ***ALDH4A1*** | ***DOK4*** | 1433B | ***COL8A1*** | ***CSF1R*** | ***APP*** | **Proteins Identified from SDS-PAGE Gel and ESI-MS** | ***Ckb*** | ***APLP1*** | ***CAPN2*** | ***SUN1*** | ***SUN1*** | IL1A | TRIM44 | |
| ***CLDN5*** | ABCA2 | 2310022A10RIK | ***2610008E11RIK*** | ***ABCA3*** | ***COL4A3BP*** | ***NUMB*** | 1433E | ***FN1*** | ***CYP24A1*** | ***BCL2*** | **Proteins that selectively interact with FLAG-OSM** | ***Tpm4*** | ***CDC25A*** | ECSCR | ***MCM3*** | ***PLOD2*** | MSN | ***ITGB4*** | |
| ***DLL4*** | ABCA4 | 2310022B05RIK | 5430416O09RIK | ***ACY1*** | ***DNM1L*** | ***PLEK*** | 1433F | ***ITGA2*** | ***CYP27A1*** | ***BCL2L1*** | ***HSP90*** | ***Tubb4b*** | ***CDC25B*** | ***MCM3*** | ***SFPQ*** | ***PRKCSH*** | EGR1 | SULT1A3 | |
| ***HEG1*** | ABCC1 | ***2610008E11RIK*** | 9330182L06RIK | ***AK1*** | ***NOP58*** | ***RGS12*** | 1433G | ***ITGB4*** | ***CYP2R1*** | ***BCL2L2*** | ***KRIT1*** | ***Vim*** | ***CDC25C*** | ***SUN1*** | PA2G4 | ***CAV1*** | ***ITGB4*** | ***TJP1*** | |
| ***ICAM1*** | ABCC3 | 3110043O21RIK | ***A430105I19RIK*** | ***AMD1*** | ***NPRL3*** | ***TBC1D4*** | 1433T | ***LAMA5*** | ***DYRK1A*** | ***BRSK1*** | HSP70 | ***Calr*** | ***CDC42*** | ***GLOD4*** | ***CNN3*** | ***DDB1*** | EXOC5 | U2AF1 | |
| ***KLF2*** | ABCC8 | 4930402H24RIK | ***AAED1*** | ***ATP8B2*** | ***PDCD10*** | ***TLN2*** | 1433Z | ***MYO1B*** | ***FLT1*** | ***CAMK1D*** | ***TUBA4A*** | ***Eno1*** | ***ARHGEF5*** | ***GAPDH*** | ***PSMEI*** | ***GLOD4*** | ***TJP1*** | | |
| ***KLF4*** | ABHD15 | 4930427A07RIK | ABCB1A | C16orf13 | ***RPL21*** | ***TNS2*** | 1B59 | ***PLEC*** | ***KIT*** | ***CAMK2A*** | ***TUBA1C*** | ***Gapdh*** | ***ARHGEF15*** | ***CNN3*** | ***NOS3*** | ***PSMEI*** | ***CCL2*** | | |
| ***KRIT1*** | ***ABI3*** | 4930452B06RIK | ***ABCB1B*** | ***C1GALT1*** | ***RPS18*** | AGAP2 | 1B56 | ***SPTAN1*** | ***LCK*** | ***CAMK2B*** | ***TUBB4A*** | Hspd1 | ***ARHGEF26*** | CTSB | TALDO1 | | PRPF8 | | |
| ***NCAM1*** | ABI3BP | 4930453N24RIK | ***ACER3*** | ***CAMKK2*** | ***SCAP*** | APBB1 | 1B55 | ***SPTBN2*** | ***NR1I2*** | ***CCR5*** | EEF1A1 | ***Tpm3*** | ***NGEF*** | ERH | ***PRKCSH*** | | BCL2A1 | | |
| ***PDCD10*** | ***ABR*** | 4930523C07RIK | ***ACOT11*** | ***CD63*** | ***SNX2*** | APPL1 | 1B46 | ***THBS1*** | ***PDGFRA*** | ***CDC25A*** | CAPZA1 | | ***DLL1*** | ***NOS3*** | ***DDB1*** | | LMAN1 | | |
| ***PECAM1*** | ACSBG1 | 4930578C19RIK | ***ADAMTS12*** | ***CELF2*** | | CAPON | 1B15 | ***CDH5*** | ***PLK1*** | ***CDC25C*** | RPS3 | | ***DLL4*** | ***PRKCSH*** | HSPA4 | | NOV | | |
| ***RAP1A*** | ACSL3 | 5730508B09RIK | ***ADK*** | ***CLCN3*** | | CCM1 | 1B53 | ***CFL1*** | ***PPP3CA*** | ***CYP24A1*** | ***PDCD10*** | | ***JAG2*** | ***DDB1*** | ***HSPB1*** | | KLF8 | | |
| ***SLC2A1*** | ***ADAM12*** | 6430548M08RIK | AEBP2 | ***CPE*** | | DAB1 | 1B35 | CFL2 | ***RARA*** | ***CYP27A1*** | SLC25A4 | | ***RORA*** | ***CAV1*** | SGTA | | DHRSX | | |
| ***SLC3A2*** | ADAM19 | 9130008F23RIK | ***AFAP1L2*** | ***CRIM1*** | | DOK1 | 1B67 | ***CNN2*** | ***RARB*** | ***CYP2R1*** | **Proteins that nonspecifically interact with anti-FLAG antibody-conjugated beads** | | ***RORC*** | CAPN9 | | | | | |
| ***TSC2*** | ADAM23 | 9930111J21RIK1 | AGFG2 | ***CSRP2*** | | EPS8 | 1B39 | COL1A2 | ***RARG*** | ***DNM1*** | FLII | | ***ARHGAP30*** | STRN3 | | | | | |
| ***VIM*** | ***ADAM8*** | ***A430105I19RIK*** | AGGF1 | ***DHRS7B*** | | EPS8L3 | 1B38 | COL4A6 | ***RXRA*** | ***DPP4*** | STK38 | | ***ARHGAP31*** | | | | | | |
| ANGPT1 | ADAMTS1 | A4GALT | AGO2 | ***DPAGT1*** | | FAM43A | 1B57 | DAG1 | ***RXRB*** | ***DYRK1A*** | ACTG1 | | ***SDC1*** | | | | | | |
| CALM2 | ***ADAMTS12*** | ***AAED1*** | ***AHNAK*** | ***EFHD2*** | | FRS2 | 1C07 | ***FLNB*** | ***RXRG*** | ***EPHA2*** | TMOD3 | | ***SDC4*** | | | | | | |
| ***CCM2*** | ADAMTS2 | ***AAMP*** | AI837181 | ***ENPP6*** | | GULP1 | 2AAA | ITGA10 | ***SNCA*** | ***FYN*** | | | ***EXOC6B*** | | | | | | |
| CD58 | ADAMTS5 | ***AARS*** | ***AIDA*** | ***EXT1*** | | GULP1-t | 4F2 | LAMB3 | ***TUBB4B*** | ***G6PD*** | **Proteins Identified by MudPIT Analysis That Selectively Interact with FLAG-OSM** | | ***STXBP6*** | | | | | | |
| CEACAM5 | ADAMTSL1 | AATK | ***ALDH4A1*** | ***FAXC*** | | ICAP1 | 5NTD | MLCP | ***USP1*** | ***LTB4R*** | **Previously identified CCM complex members** | | ***FERMT3*** | | | | | | |
| CTTN | ADAMTSL3 | ***ABCA3*** | ALG10B | ***FGFR3*** | | JIP1 | 6PGD | RAC1 | ***WEE1*** | ***MAP2K5*** | MEKK3 | | ***CCT2*** | | | | | | |
| CYP2B6 | ADCY1 | ABCA5 | ***ALS2CL*** | ***G6PD*** | | LDLRAP1 | A2MG | | ADORA3 | ***MAP4K4*** | Rac | | ARHGEF16 | | | | | | |
| EPHB2 | ***ADD1*** | ABCA7 | ***ANTXR2*** | ***GABARAPL1*** | | mPiD1 | AATM | | CACNA1D | ***MELK*** | ICAP-1 | | ARHGEF19 | | | | | | |
| GLI3 | ADD2 | ABCA8B | ***ANXA2*** | ***GNA12*** | | mpiD1-t | ACLY | | CACNB2 | ***MMP14*** | ***KRIT1*** | | ETF1 | | | | | | |
| IL6 | AEBP1 | ABCA9 | ***AP3M1*** | ***HLCS*** | | NUMB-L | ACTG | | CCNB2 | ***MMP2*** | ***PDCD10*** | | JAG1 | | | | | | |
| ITGB1BP1 | AFAP1 | ***ABCB1B*** | ***APLNR*** | ***HMGCL*** | | NUMB-ss | ***ACTB*** | | CDK5 | ***NPC1L1*** | **Cytoskeletal proteins** | | HNRNPA0 | | | | | | |
| MAP3K3 | AGTPBP1 | ABCB9 | ***ARF2*** | ***IPMK*** | | RABGAP1 | ACTBL | | CLK1 | ***NQO1*** | ***MYH10*** | | HNRNPA1 | | | | | | |
| MAPK1 | AHI1 | ABCC5 | ***ARHGAP28*** | ***IQGAP3*** | | SHC1 | ACTN1 | | CRABP1 | ***NR1H4*** | TUBB6 | | HNRNPA3 | | | | | | |
| MAPK3 | AHNAK2 | ABCD1 | ARNTL | ***ITGA8*** | | SNX17 | ACTN4 | | CRABP2 | ***NR1I2*** | ***TUBB4A*** | | HNRNPA1L2 | | | | | | |
| MSH2 | ***AIM1*** | ABCG1 | ***ASAP1*** | ***KIF3A*** | | TBC1D1 | ACTZ | | CYP27B1 | ***PBK*** | ***TUBB4B*** | | HNRNPA2B1 | | | | | | |
| PRRT2 | AK5 | ABHD17A | ***ATF6B*** | ***KRIT1*** | | TNS2-t | ADH2 | | DHODH | ***PPP3R1*** | ***CFL1*** | | HIST1H3A | | | | | | |
| PTEN | ***AKT3*** | ABHD17B | ***ATP2B4*** | ***LRRC57*** | | Ttbc1d4-t | ADT3 | | EHHADH | ***PRKCG*** | ***TUBA4A*** | | HIST1H3B | | | | | | |
| PTGS2 | ALPK2 | ***ABI3*** | ***ATP7A*** | ***MGAT5*** | | | AHNK | | FLT4 | ***PRKCH*** | ***TUBA1C*** | | HIST1H3C | | | | | | |
| RABGEF1 | ALPL | ABLIM3 | AZIN1 | ***MOSPD2*** | | | AKA12 | | GPR35 | ***PSIP1*** | ***TUBA1A*** | | HIST1H3D | | | | | | |
| SERPINI1 | AMER3 | ***ABR*** | ***B4GALT5*** | ***MRAS*** | | | ALBU | | KDR | ***PTGES*** | **Protein translation and folding** | | HIST1H3E | | | | | | |
| SMARCE1 | AMICA1 | ACACB | B9D2 | ***NEK7*** | | | ALDOA | | LRRK2 | ***PTPN1*** | ***EF1A1*** | | HIST1H3F | | | | | | |
| TERF2IP | AMPH | ACAD12 | ***BAG5*** | ***NMT2*** | | | AMPL | | MST1R | ***RARA*** | CCT3 | | HIST1H3G | | | | | | |
| TP53 | ANK2 | ACADSB | ***BAIAP2*** | ***NT5C2*** | | | AMPN | | PDGFRB | ***RARB*** | CCT6A | | HIST1H3H | | | | | | |
| ULK1 | ANK3 | ***ACER3*** | ***BAIAP2L1*** | ***NTN1*** | | | ANR31 | | SRC | ***RARG*** | CCT7 | | HIST1H3I | | | | | | |
| VEGFA | ANKRD36B | ***ACOT11*** | ***BCAS3*** | ***NUP85*** | | | ***ANXA1*** | | TNNC2 | ***RBP1*** | TCP1 | | HIST1H3J | | | | | | |
| | ANKS1B | ACTA2 | BCCIP | ***OXCT1*** | | | ***ANXA2*** | | ***TUBA1A*** | ***RORA*** | ***DNAJB6*** | | HIST3H3 | | | | | | |
| | ***ANTXR1*** | ***ACTB*** | ***BCL10*** | ***PAQR8*** | | | ANXA3 | | TUBA1B | ***RORC*** | DNAJA2 | | BTF3 | | | | | | |
| | ***ANTXR2*** | ACTR3 | ***BCL2*** | ***PDGFRA*** | | | ***ANXA5*** | | TUBB | ***RXRA*** | ***CCT2*** | | BTF3L4 | | | | | | |
| | ***ANXA1*** | ACVRL1 | ***BEND7*** | ***PLCD3*** | | | ***ANXA6*** | | TUBB1 | ***RXRB*** | CCT5 | | BTRC | | | | | | |
| | AOAH | ***ACY1*** | ***BICD2*** | ***PLK1*** | | | APMAP | | ***TUBB4A*** | ***RXRG*** | CCT8 | | FBXW11 | | | | | | |
| | ***APBA2*** | ACY3 | ***BMP4*** | ***PLXDC2*** | | | ***ARF3*** | | | ***SLK*** | CCT4 | | CAB39 | | | | | | |
| | APC | ADA | ***BMP6*** | ***POLD1*** | | | ***ARF1*** | | | ***TLR4*** | DNAJA1 | | CAB39L | | | | | | |
| | APLN | ADAM11 | ***BST1*** | ***POLH*** | | | ***ARF4*** | | | ***TOP2B*** | ***HSP90*** | | RORB | | | | | | |
| | ***APLNR*** | ***ADAM12*** | BTD | ***PRPS2*** | | | ARP3 | | | AGTR1 | **Signaling** | | HNF4A | | | | | | |
| | APOBR | ADAM15 | ***BTG2*** | ***RAD51*** | | | ARPC4 | | | AKR1B1 | RIN2 | | HNF4G | | | | | | |
| | APOL6 | ***ADAM8*** | ***C1QTNF1*** | ***REEP2*** | | | ATPA | | | AKT1 | PPP2R2D | | PABPC1 | | | | | | |
| | ARHGAP21 | ADAMTS10 | ***C2CD2L*** | ***RFC2*** | | | ATPB | | | AKT2 | PPP2CA | | PPP4R2 | | | | | | |
| | ARHGAP25 | ADAT2 | ***CACNA1A*** | ***RPS6KA5*** | | | ATPO | | | ALDH1A2 | GNB2L1 | | RAB5A | | | | | | |
| | ARHGAP26 | ADD3 | ***CACNB3*** | ***RRAS2*** | | | B2MG | | | ALDH1A3 | ***GNB4*** | | RAB5B | | | | | | |
| | ***ARHGAP30*** | ADGRA3 | ***CAMK2N1*** | ***SLC12A8*** | | | BASP1 | | | ALOX12 | PPP2R1A | | RAB5C | | | | | | |
| | ARHGAP35 | ADGRE5 | ***CAND2*** | ***SLIT2*** | | | BGAL | | | ALOX15 | PPP1CC | | RAD21 | | | | | | |
| | ARHGDIB | ADGRG1 | ***CAPN2*** | ***SPARCL1*** | | | BST2 | | | ALOX15B | **Vesicular transport proteins** | | RAD21L1 | | | | | | |
| | ***ARHGEF9*** | ADGRG3 | ***CAR2*** | ***SUSD2*** | | | C1QBP | | | ALOX5 | TFRC | | SDC2 | | | | | | |
| | ARL11 | ADGRL3 | ***CAR5B*** | TMEM180 | | | CALD1 | | | ALOX5AP | COPB1 | | SDC3 | | | | | | |
| | ***ARNT2*** | ADGRL4 | ***CAR7*** | ***TRPC6*** | | | CALM | | | AMY1A | ***COPA*** | | EXOC6 | | | | | | |
| | ARSD | ADIPOR2 | ***CASP3*** | ***TTC38*** | | | ***CALR*** | | | APEX1 | ***ARF1*** | | EXOC1 | | | | | | |
| | ASH1L | ***ADK*** | ***CAST*** | ***TUBB2B*** | | | CALX | | | BACE1 | ***ARF2*** | | MAP2K4 | | | | | | |
| | ASPM | ADM | ***CBX6*** | ***UBR7*** | | | CAND1 | | | BAD | ***ARF3*** | | SKP1 | | | | | | |
| | ASPN | ADORA2A | ***CCDC12*** | ***UNC13A*** | | | CAP1 | | | CAMK1 | ***ARF4*** | | AC104109.3 | | | | | | |
| | ATCAY | ***AFAP1L2*** | CCDC88C | ***VEPH1*** | | | CATB | | | CAMK2D | ARF5B | | ACAT1 | | | | | | |
| | ATL1 | AFF4 | CCDC93 | ***WARS*** | | | CATD | | | CAMK2G | COPG1 | | AASDHPPT | | | | | | |
| | ATP12A | AGAP1 | ***CCL2*** | ***WLS*** | | | ***CAV1*** | | | CCKAR | **Metabolism and biosynthetic proteins** | | RPS27A | | | | | | |
| | ATP1A2 | AGMO | ***CCM2L*** | ***XPO4*** | | | CAZA1 | | | CDC42BPA | ***ENO1*** | | FERMT1 | | | | | | |
| | ATP1A3 | AGPAT2 | CCNI | ***ZDHHC14*** | | | ***CDC42*** | | | CLK3 | ***CAD*** | | FERMT2 | | | | | | |
| | ATP1B1 | ***AHNAK*** | CD164 | | | | CH10 | | | CNR1 | ***PAICS*** | | | | | | | | |
| | ATP2B2 | AI467606 | ***CD247*** | | | | CH60 | | | CREB1 | **Miscellaneous** | | | | | | | | |
| | ATP2B3 | AI661453 | CD38 | | | | CISY | | | CYP1A2 | FAR1 | | | | | | | | |
| | ***ATP8A1*** | ***AIDA*** | ***CD40*** | | | | CKAP4 | | | CYP26A1 | H2-D1 | | | | | | | | |

| | | | | | | | | | | | | | | | | | | | |
|---|---|---|---|---|---|---|---|---|---|---|---|---|---|---|---|---|---|---|---|
| | ATP8B4 | ***AIM1*** | ***CD82*** | | | | CLH1 | | | CYP2C19 | OAS1 | | | | | | | | |
| | ***ATP9A*** | ***AK1*** | ***CD93*** | | | | CLIC1 | | | CYP2C9 | PSMD7 | | | | | | | | |
| | ATRNL1 | AK4 | CDCA7L | | | | ***CLIC4*** | | | CYP2D6 | SV2 | | | | | | | | |
| | BAI1 | AK6 | CDK4 | | | | ***CNN2*** | | | CYP3A4 | HLA-A | | | | | | | | |
| | BCAT1 | AKAP13 | ***CDKN2B*** | | | | CO4A1 | | | DCLK1 | | | | | | | | | |
| | BEND3 | AKIRIN2 | CEP131 | | | | CO4A2 | | | DRD3 | | | | | | | | | |
| | BEX1 | AKR1B8 | ***CERK*** | | | | COF1 | | | DYRK4 | | | | | | | | | |
| | BICC1 | AKR1C14 | ***CFAP43*** | | | | COIA1 | | | EGFR | | | | | | | | | |
| | BIN1 | ALAS1 | ***CFTR*** | | | | ***COPA*** | | | EIF2AK4 | | | | | | | | | |
| | BMF | ALCAM | ***CGNL1*** | | | | COTL1 | | | EIF4G1 | | | | | | | | | |
| | BNC2 | ALDH16A1 | ***CHAC1*** | | | | COX2 | | | EP300 | | | | | | | | | |
| | BOC | ALDH18A1 | CHD1 | | | | COX5A | | | ERBB2 | | | | | | | | | |
| | ***BRCA2*** | ALDH1A1 | ***CHN2*** | | | | CTNB1 | | | FAAH | | | | | | | | | |
| | ***BRSK1*** | ALDH1L2 | ***CHRD*** | | | | CYC | | | GC | | | | | | | | | |
| | BSN | ALDH2 | ***CHST7*** | | | | DDX3X | | | GRK5 | | | | | | | | | |
| | BTBD10 | ALDH3A2 | CLIP1 | | | | DPY30 | | | HIPK4 | | | | | | | | | |
| | C16orf54 | ***ALS2CL*** | ***CLTB*** | | | | DX39B | | | HMGCR | | | | | | | | | |
| | C1orf68 | ***AMD1*** | ***CMIP*** | | | | DYHC1 | | | HSP90AA1 | | | | | | | | | |
| | C1QC | AMMECR1 | ***CMKLR1*** | | | | ECHA | | | IDH1 | | | | | | | | | |
| | C1R | ANGPT2 | CNNM3 | | | | ECHB | | | LSS | | | | | | | | | |
| | C1S | ANKRD1 | COG1 | | | | ***EF1A1*** | | | MAP3K1 | | | | | | | | | |
| | C5AR1 | ANKRD10 | ***COL4A3BP*** | | | | EF1A3 | | | MAPK12 | | | | | | | | | |
| | C5orf63 | ANKRD29 | COL7A1 | | | | EF1D | | | MAPK13 | | | | | | | | | |
| | CA10 | ANKRD33B | ***CPM*** | | | | EF1G | | | MAPKAPK3 | | | | | | | | | |
| | CABP1 | ANKRD46 | ***CPNE2*** | | | | EF2 | | | MARK3 | | | | | | | | | |
| | CACNA1B | ANO1 | CPSF4 | | | | EFTU | | | MATK | | | | | | | | | |
| | CACNA2D3 | ***ANTXR1*** | CRACR2B | | | | EGLN | | | MCL1 | | | | | | | | | |
| | CACNG2 | ***ANTXR2*** | ***CREB3L2*** | | | | ***EHD4*** | | | MMP13 | | | | | | | | | |
| | CACNG3 | ***ANXA2*** | ***CRIP1*** | | | | EIF3F | | | MMP7 | | | | | | | | | |
| | CADM3-AS1 | ***ANXA5*** | CSNK1A1 | | | | ENOA | | | MPG | | | | | | | | | |
| | ***CALM1*** | ***ANXA6*** | CTNND1 | | | | ENPL | | | PIP4K2C | | | | | | | | | |
| | ***CALM3*** | ***AP3M1*** | CXCL16 | | | | ERAP1 | | | PLD1 | | | | | | | | | |
| | CALY | APBA1 | ***CXCR4*** | | | | ERO1A | | | PLK4 | | | | | | | | | |
| | ***CAMK2A*** | APBB2 | CYB561D2 | | | | ERP29 | | | POLA1 | | | | | | | | | |
| | ***CAMK2B*** | APCDD1 | ***CYB5R1*** | | | | ESYT1 | | | PRKCD | | | | | | | | | |
| | ***CAMK2N1*** | ***APLNR*** | ***CYFIP1*** | | | | EZRI | | | PRKCE | | | | | | | | | |
| | CAMKV | ***APLP1*** | ***CYP4V3*** | | | | F213A | | | PRKCI | | | | | | | | | |
| | CAMSAP2 | APOE | ***CYTH3*** | | | | ***FAS*** | | | PRKCQ | | | | | | | | | |
| | CAP2 | APOL10B | ***D930015E06RIK*** | | | | FETA | | | PRKCZ | | | | | | | | | |
| | ***CAPG*** | APOLD1 | ***DACH1*** | | | | FETUA | | | PRKD2 | | | | | | | | | |
| | CARD11 | APRT | ***DAPK2*** | | | | FINC | | | PTPN2 | | | | | | | | | |
| | CASC18 | ARAP1 | ***DCBLD2*** | | | | ***FLNA*** | | | RBP4 | | | | | | | | | |
| | CASC5 | ***ARF2*** | ***DHH*** | | | | ***FLNB*** | | | RCE1 | | | | | | | | | |
| | CASP1 | ARFGAP3 | ***DIP2C*** | | | | ***FLNC*** | | | ROS1 | | | | | | | | | |
| | CASP14 | ARHGAP10 | ***DMTN*** | | | | ***FSCN1*** | | | SGK2 | | | | | | | | | |
| | ***CASP8*** | ARHGAP20 | ***DNM1L*** | | | | G3P | | | SLC25A20 | | | | | | | | | |
| | CASQ2 | ARHGAP22 | ***DNM3*** | | | | G6PI | | | SLCO1B1 | | | | | | | | | |
| | ***CBX6*** | ARHGAP23 | ***DOCK9*** | | | | GANAB | | | SLCO1B3 | | | | | | | | | |
| | CCAR1 | ARHGAP27 | ***DOK4*** | | | | GBLP | | | SREBF2 | | | | | | | | | |
| | CCDC102B | ***ARHGAP28*** | ***DPP4*** | | | | GDIB | | | STK11 | | | | | | | | | |
| | CCDC66 | ARHGAP29 | DUSP6 | | | | GDIR1 | | | STK3 | | | | | | | | | |
| | CCDC88A | ***ARHGAP31*** | ***DYRK1B*** | | | | GDIR2 | | | TACR2 | | | | | | | | | |
| | CCK | ARHGAP33 | ***DYSF*** | | | | GELS | | | TAOK1 | | | | | | | | | |
| | CCR1 | ARHGAP8 | E130309D02RIK | | | | GLGB | | | TDP2 | | | | | | | | | |
| | CCR2 | ARHGEF10 | ***E2F2*** | | | | GLU2B | | | TNK2 | | | | | | | | | |
| | ***CCR5*** | ***ARHGEF15*** | ECE1 | | | | GNAI2 | | | TOP2A | | | | | | | | | |
| | CD109 | ***ARHGEF26*** | ***EDN1*** | | | | GNS | | | TRPV1 | | | | | | | | | |
| | ***CD14*** | ARHGEF28 | ***EFEMP1*** | | | | GRP75 | | | TXN2 | | | | | | | | | |
| | CD163 | ***ARHGEF5*** | ***EHD1*** | | | | GRP78 | | | VDR | | | | | | | | | |
| | CD163L1 | ***ARHGEF9*** | EIF3A | | | | ***GSTO1*** | | | | | | | | | | | | |
| | CD1D | ARID3A | ***ELF4*** | | | | GSTP1 | | | | | | | | | | | | |
| | CD209 | ARL4A | ***ELN*** | | | | GT251 | | | | | | | | | | | | |
| | CD28 | ARL4C | EML1 | | | | H12 | | | | | | | | | | | | |
| | CD36 | ARL4D | EML6 | | | | H15 | | | | | | | | | | | | |
| | CD4 | ARL5A | ***EMP1*** | | | | H2AX | | | | | | | | | | | | |
| | CD44 | ***ARNT2*** | ***ENDOU*** | | | | H2BFS | | | | | | | | | | | | |
| | CD5 | ARPC1B | ***ENPP2*** | | | | H2B2F | | | | | | | | | | | | |
| | CD52 | ARPIN | ***ENTPD1*** | | | | H2B1N | | | | | | | | | | | | |
| | CD6 | ARRDC4 | EPHA4 | | | | H2B1M | | | | | | | | | | | | |
| | CD68 | ***ASAP1*** | ***EPN2*** | | | | H2B1L | | | | | | | | | | | | |
| | CD74 | ASF1B | ERGIC2 | | | | H2B1K | | | | | | | | | | | | |
| | CD84 | ASNS | ***ERN1*** | | | | H2B1H | | | | | | | | | | | | |
| | CD86 | ASRGL1 | ETL4 | | | | H2B1D | | | | | | | | | | | | |
| | CD8A | ASS1 | EXOSC7 | | | | H2B1C | | | | | | | | | | | | |
| | CD99L2 | ATAD5 | ***F2RL2*** | | | | H33 | | | | | | | | | | | | |
| | CDH10 | ATE1 | F8A | | | | H4 | | | | | | | | | | | | |
| | CDH11 | ATF3 | FADS1 | | | | HBA | | | | | | | | | | | | |
| | CDH18 | ATF4 | ***FADS2*** | | | | HHIP | | | | | | | | | | | | |
| | CDH23 | ***ATF6B*** | ***FAM107A*** | | | | HMGA1 | | | | | | | | | | | | |
| | ***CDKN2B*** | ATG7 | ***FAM129A*** | | | | HMGB1 | | | | | | | | | | | | |
| | CDKN2B-AS1 | ATOH8 | ***FAM129B*** | | | | HNRH1 | | | | | | | | | | | | |
| | CELF3 | ATOX1 | FAM134C | | | | HNRPC | | | | | | | | | | | | |
| | CELSR1 | ATP11C | ***FAM189A2*** | | | | HNRPD | | | | | | | | | | | | |
| | CEMIP | ATP2B1 | ***FAM20C*** | | | | HNRPK | | | | | | | | | | | | |
| | ***CENPF*** | ***ATP2B4*** | FAM216A | | | | HNRPQ | | | | | | | | | | | | |
| | CHD5 | ATP6V0B | ***FAM49A*** | | | | HNRPU | | | | | | | | | | | | |
| | CHD6 | ATP6V1A | ***FAS*** | | | | HS90A | | | | | | | | | | | | |
| | CHI3L1 | ***ATP7A*** | ***FAT1*** | | | | HS90B | | | | | | | | | | | | |
| | CHIT1 | ***ATP8A1*** | FBXO10 | | | | HSP71 | | | | | | | | | | | | |
| | CHN1 | ***ATP8B2*** | ***FHL3*** | | | | HSP7C | | | | | | | | | | | | |
| | CHRNA10 | ***ATP9A*** | ***FKBP4*** | | | | ***HSPB1*** | | | | | | | | | | | | |
| | ***CHST11*** | ATXN1 | ***FLNC*** | | | | HTRA1 | | | | | | | | | | | | |
| | CIITA | AURKB | FOXN2 | | | | HUWE1 | | | | | | | | | | | | |
| | CISH | AUTS2 | ***FOXO1*** | | | | HYOU1 | | | | | | | | | | | | |
| | ***CIT*** | AVL9 | ***FRZB*** | | | | ***ICAM1*** | | | | | | | | | | | | |
| | ***CKAP2L*** | B3GALNT1 | ***FSCN1*** | | | | IDHC | | | | | | | | | | | | |
| | CLASP2 | B3GNTL1 | ***FUT8*** | | | | IF4A1 | | | | | | | | | | | | |
| | CLCN1 | B4GALT1 | ***FXYD5*** | | | | IF5A1 | | | | | | | | | | | | |
| | ***CLDN5*** | B4GALT3 | ***FYN*** | | | | ILF2 | | | | | | | | | | | | |
| | CLEC10A | B4GALT4 | G6PC3 | | | | IMA2 | | | | | | | | | | | | |
| | CLEC11A | ***B4GALT5*** | ***GADD45A*** | | | | IMB1 | | | | | | | | | | | | |
| | CLEC2D | ***BAG5*** | ***GBP4*** | | | | IPO5 | | | | | | | | | | | | |
| | CLEC5A | BAHCC1 | GDE1 | | | | IPO7 | | | | | | | | | | | | |
| | CLEC7A | ***BAIAP2*** | ***GDPD5*** | | | | IQGA1 | | | | | | | | | | | | |
| | CLIP3 | ***BAIAP2L1*** | GINM1 | | | | ISG15 | | | | | | | | | | | | |
| | CLK2P | BAMBI | ***GIT1*** | | | | ITA5 | | | | | | | | | | | | |
| | CLMP | BARD1 | GK5 | | | | ITB1 | | | | | | | | | | | | |
| | CLVS2 | BBIP1 | ***GM525*** | | | | K1C10 | | | | | | | | | | | | |
| | ***CMKLR1*** | BC052040 | ***GNG2*** | | | | K1C14 | | | | | | | | | | | | |
| | CNKSR2 | BC055324 | ***GNG5*** | | | | K1C9 | | | | | | | | | | | | |
| | CNTN3 | BCAM | GNL2 | | | | K22E | | | | | | | | | | | | |
| | CNTNAP2 | BCAR3 | GOSR2 | | | | K2C1 | | | | | | | | | | | | |
| | CNTNAP5 | ***BCAS3*** | ***GRAMD4*** | | | | K2C7 | | | | | | | | | | | | |
| | COL14A1 | BCAT2 | ***GRAP*** | | | | KPYM | | | | | | | | | | | | |
| | ***COL15A1*** | ***BCL10*** | ***GRASP*** | | | | ***LAMA4*** | | | | | | | | | | | | |
| | COL22A1 | ***BCL2*** | ***GSDMD*** | | | | LAMC1 | | | | | | | | | | | | |
| | ***COL27A1*** | ***BCL2L1*** | H2-K1 | | | | LAMP1 | | | | | | | | | | | | |
| | COL4A4 | BCL2L11 | HCFC1R1 | | | | LAMP2 | | | | | | | | | | | | |
| | COL6A2 | ***BCL2L2*** | ***HEATR5A*** | | | | LDHA | | | | | | | | | | | | |
| | COL6A3 | BCL3 | ***HECW2*** | | | | ***LDHB*** | | | | | | | | | | | | |
| | COL6A4P2 | BCL6 | ***HEG1*** | | | | LEG1 | | | | | | | | | | | | |
| | ***COL8A1*** | BCL7C | HERPUD2 | | | | LMNA | | | | | | | | | | | | |
| | ***COLEC12*** | BCL9L | HHEX | | | | MAP1B | | | | | | | | | | | | |

| | | | | | | | | | | | | | | | | | | | |
|---|---|---|---|---|---|---|---|---|---|---|---|---|---|---|---|---|---|---|---|
| | CPLX1 | BCORL1 | ***HID1*** | | | | MAP4 | | | | | | | | | | | | |
| | CPLX2 | BEAN1 | HNRNPH2 | | | | MARCS | | | | | | | | | | | | |
| | CPNE6 | ***BEND7*** | HPS6 | | | | MATR3 | | | | | | | | | | | | |
| | CPVL | BEST1 | HRAS | | | | MDHM | | | | | | | | | | | | |
| | CPXM1 | BEX3 | HSDL2 | | | | ML12A | | | | | | | | | | | | |
| | CPXM2 | BHLHE40 | ***HSPA12B*** | | | | ML12B | | | | | | | | | | | | |
| | CPZ | BHLHE41 | HSPH1 | | | | MMP1 | | | | | | | | | | | | |
| | CR1 | ***BICD2*** | HTT | | | | ***MMRN2*** | | | | | | | | | | | | |
| | ***CRCT1*** | BID | ***HYAL1*** | | | | MOES | | | | | | | | | | | | |
| | CRISPLD1 | BLVRB | IER5L | | | | MOGS | | | | | | | | | | | | |
| | CRNN | BMP2 | IFI47 | | | | ***MT2*** | | | | | | | | | | | | |
| | ***CSF1R*** | ***BMP4*** | ***IFNAR1*** | | | | MUC18 | | | | | | | | | | | | |
| | CSF3R | ***BMP6*** | ***IFNGR1*** | | | | MVP | | | | | | | | | | | | |
| | ***CTGF*** | BRCA1 | IFNGR2 | | | | MX1 | | | | | | | | | | | | |
| | ***CTSC*** | ***BRCA2*** | IFT81 | | | | ***MYH9*** | | | | | | | | | | | | |
| | CTSK | BRIP1 | ***IL13RA1*** | | | | MYL6 | | | | | | | | | | | | |
| | ***CTSL*** | ***BST1*** | ***IL3RA*** | | | | ***MYO1C*** | | | | | | | | | | | | |
| | CTSS | ***BTG2*** | IL7 | | | | NACA | | | | | | | | | | | | |
| | CTSZ | BUB1B | ***INPP4B*** | | | | NB5R3 | | | | | | | | | | | | |
| | CUX2 | ***C1GALT1*** | ***IQGAP2*** | | | | NDKA | | | | | | | | | | | | |
| | ***CX3CL1*** | ***C1QTNF1*** | IRF2BP1 | | | | NEST | | | | | | | | | | | | |
| | CXCL12 | C1QTNF9 | ***IRF8*** | | | | NP1L1 | | | | | | | | | | | | |
| | ***CXCR4*** | C1RA | ***ITGA2*** | | | | NPM | | | | | | | | | | | | |
| | CYBB | ***C2CD2L*** | ***ITGA9*** | | | | NUCL | | | | | | | | | | | | |
| | CYFIP2 | C3 | ***ITPR3*** | | | | OCAD2 | | | | | | | | | | | | |
| | ***CYP27A1*** | C330027C09RIK | ***JAM3*** | | | | ODO2 | | | | | | | | | | | | |
| | CYP2S1 | C430049E01RIK | ***JUP*** | | | | OLA1 | | | | | | | | | | | | |
| | ***CYTH4*** | CABLES1 | KANK3 | | | | OST48 | | | | | | | | | | | | |
| | CYTIP | ***CACNA1A*** | ***KAT2B*** | | | | P26069-R | | | | | | | | | | | | |
| | ***DAB2*** | ***CACNB3*** | KCNA5 | | | | P43064-R | | | | | | | | | | | | |
| | DACT1 | ***CAD*** | ***KCNJ2*** | | | | PA1B2 | | | | | | | | | | | | |
| | DAPP1 | CADM3 | ***KCNQ4*** | | | | PABP1 | | | | | | | | | | | | |
| | DCN | ***CAMK1D*** | ***KCTD12B*** | | | | PABP3 | | | | | | | | | | | | |
| | DENND1C | ***CAMK2A*** | KCTD17 | | | | PAI1 | | | | | | | | | | | | |
| | DENND2D | ***CAMK2N1*** | KCTD9 | | | | PAXI1 | | | | | | | | | | | | |
| | DENND3 | ***CAMKK2*** | KDELR1 | | | | PCBP1 | | | | | | | | | | | | |
| | ***DHRS3*** | ***CAND2*** | ***KDSR*** | | | | PCBP2 | | | | | | | | | | | | |
| | DIRAS1 | ***CAPG*** | ***KIF13A*** | | | | PCP | | | | | | | | | | | | |
| | DIRAS2 | ***CAPN2*** | ***KIF15*** | | | | PDIA1 | | | | | | | | | | | | |
| | DLG2 | CAPN5 | ***KIF23*** | | | | PDIA3 | | | | | | | | | | | | |
| | ***DLG4*** | ***CAR2*** | ***KIT*** | | | | PDIA4 | | | | | | | | | | | | |
| | DLGAP1 | CAR4 | ***KLC1*** | | | | ***PDIA6*** | | | | | | | | | | | | |
| | DLGAP3 | ***CAR5B*** | ***KLHL6*** | | | | PDLI5 | | | | | | | | | | | | |
| | DMBT1P1 | ***CAR7*** | ***KRAS*** | | | | PEA15 | | | | | | | | | | | | |
| | ***DMTN*** | CARD10 | KRTCAP2 | | | | PEBP1 | | | | | | | | | | | | |
| | DNAJC6 | CARHSP1 | KSR1 | | | | PECA1 | | | | | | | | | | | | |
| | ***DNM1*** | CARS | ***L1CAM*** | | | | PGAM1 | | | | | | | | | | | | |
| | ***DNM3*** | CASD1 | ***LAMB1*** | | | | PGBM | | | | | | | | | | | | |
| | DOCK2 | CASKIN2 | ***LAMB2*** | | | | PGK1 | | | | | | | | | | | | |
| | ***DOCK8*** | CASP12 | ***LCP2*** | | | | PHB | | | | | | | | | | | | |
| | ***DOCK9*** | ***CASP3*** | ***LHFPL2*** | | | | PHB2 | | | | | | | | | | | | |
| | DPYSL2 | ***CASP8*** | ***LIMD2*** | | | | ***PIR*** | | | | | | | | | | | | |
| | DSCAM | ***CAST*** | ***LMBR1*** | | | | ***PLEC*** | | | | | | | | | | | | |
| | DSG2 | CASZ1 | LMF1 | | | | ***PLOD1*** | | | | | | | | | | | | |
| | DST | CBX7 | ***LMO4*** | | | | ***PLOD2*** | | | | | | | | | | | | |
| | DTHD1 | CC2D2A | LMO7 | | | | PLOD3 | | | | | | | | | | | | |
| | DTNB | ***CCDC12*** | ***LONRF2*** | | | | PLST | | | | | | | | | | | | |
| | DUSP4 | CCDC138 | ***LOXL4*** | | | | PNPH | | | | | | | | | | | | |
| | DYNC1H1 | CCDC14 | LPCAT1 | | | | POLG_BSTV1 | | | | | | | | | | | | |
| | EBF2 | CCDC141 | LRFN4 | | | | PP1A | | | | | | | | | | | | |
| | EDA | CCDC149 | LRRC58 | | | | PPIA | | | | | | | | | | | | |
| | EFCAB4B | CCDC18 | ***LSR*** | | | | PPIB | | | | | | | | | | | | |
| | ***EFEMP1*** | CCDC184 | ***LTBP2*** | | | | PRDX1 | | | | | | | | | | | | |
| | ELAVL2 | CCDC61 | ***LUZP1*** | | | | PRDX2 | | | | | | | | | | | | |
| | ELAVL4 | CCDC85A | LY96 | | | | PRDX3 | | | | | | | | | | | | |
| | ***ELN*** | ***CCM2L*** | LYL1 | | | | PRDX5 | | | | | | | | | | | | |
| | ELOVL7 | CCNA2 | LYSMD2 | | | | PRDX6 | | | | | | | | | | | | |
| | EMILIN1 | CCNB1 | ***MAFB*** | | | | PRKDC | | | | | | | | | | | | |
| | EMILIN2 | CCND1 | ***MAGED1*** | | | | PROF1 | | | | | | | | | | | | |
| | EMP3 | CCND3 | MAGEH1 | | | | PSA | | | | | | | | | | | | |
| | ENC1 | CCNE1 | MAGIX | | | | PSA2 | | | | | | | | | | | | |
| | ENO2 | CCNE2 | ***MAL*** | | | | PSB6 | | | | | | | | | | | | |
| | ENPP1 | CCNF | ***MAN1A*** | | | | PSMD2 | | | | | | | | | | | | |
| | ***EPAS1*** | CCNJ | ***MAN2A1*** | | | | PSME1 | | | | | | | | | | | | |
| | EPB41L1 | CCRL2 | MAP1LC3B | | | | PSME2 | | | | | | | | | | | | |
| | ***EPN2*** | CCSER1 | ***MAP2*** | | | | PTBP1 | | | | | | | | | | | | |
| | EPN3 | ***CD14*** | ***MAP2K5*** | | | | ***PTMA*** | | | | | | | | | | | | |
| | EPPK1 | CD1D1 | ***MAP3K8*** | | | | ***PTRF*** | | | | | | | | | | | | |
| | EVI2B | CD200 | ***MAPK11*** | | | | ***PXDN*** | | | | | | | | | | | | |
| | ***EXOC6B*** | ***CD247*** | ***MARCKSL1*** | | | | Q75008-R | | | | | | | | | | | | |
| | EYA2 | CD276 | ***MB21D2*** | | | | RAB1A | | | | | | | | | | | | |
| | F13A1 | ***CD40*** | ***MCFD2*** | | | | RAN | | | | | | | | | | | | |
| | ***F2R*** | CD55 | ***MCPH1*** | | | | ***RCN1*** | | | | | | | | | | | | |
| | F5 | CD59A | MED19 | | | | RL11 | | | | | | | | | | | | |
| | FAIM2 | ***CD63*** | MEF2A | | | | RL12 | | | | | | | | | | | | |
| | FAIM3 | CD81 | MEOX1 | | | | RL15 | | | | | | | | | | | | |
| | FAM101B | ***CD82*** | MEST | | | | RL18 | | | | | | | | | | | | |
| | FAM105A | CD9 | ***METRNL*** | | | | RL18A | | | | | | | | | | | | |
| | ***FAM107A*** | ***CD93*** | ***MEX3D*** | | | | RL19 | | | | | | | | | | | | |
| | ***FAM117A*** | CDC20 | MFSD10 | | | | RL22 | | | | | | | | | | | | |
| | ***FAM160A1*** | ***CDC25B*** | MFSD9 | | | | RL27 | | | | | | | | | | | | |
| | ***FAM20A*** | CDC42EP2 | ***MGLL*** | | | | RL3 | | | | | | | | | | | | |
| | FAM76A | CDC42EP3 | ***MGP*** | | | | RL31 | | | | | | | | | | | | |
| | FAM83A | CDC6 | MLEC | | | | RL4 | | | | | | | | | | | | |
| | FAM96A | CDCA3 | MLH3 | | | | RL40 | | | | | | | | | | | | |
| | FAP | CDCA5 | ***MMD*** | | | | RL6 | | | | | | | | | | | | |
| | ***FAT1*** | CDCA7 | ***MMP25*** | | | | RL7 | | | | | | | | | | | | |
| | ***FAXC*** | CDK2AP2 | ***MNAT1*** | | | | RL7A | | | | | | | | | | | | |
| | FBLN1 | CDK5RAP2 | MOSPD3 | | | | RL8 | | | | | | | | | | | | |
| | FBLN2 | CDKL1 | MRPL10 | | | | RL9 | | | | | | | | | | | | |
| | FBN1 | CDKL2 | ***MTCH1*** | | | | RLA0 | | | | | | | | | | | | |
| | FBN2 | CDKN1A | ***NCKAP5L*** | | | | RLA2 | | | | | | | | | | | | |
| | FBXL16 | ***CDKN2B*** | ***NCOA3*** | | | | ROA1 | | | | | | | | | | | | |
| | FCRL3 | CDKN2C | ***NDFIP1*** | | | | ROA2 | | | | | | | | | | | | |
| | FCRL5 | CDR2 | ***NDUFC2*** | | | | ROA3 | | | | | | | | | | | | |
| | FDXACB1 | CDR2L | NEBL | | | | ROAA | | | | | | | | | | | | |
| | FER1L4 | CDT1 | ***NECTIN3*** | | | | RPN1 | | | | | | | | | | | | |
| | FER1L5 | CEACAM1 | ***NEDD4*** | | | | RPN2 | | | | | | | | | | | | |
| | ***FERMT3*** | CEACAM2 | NEK6 | | | | RRBP1 | | | | | | | | | | | | |
| | FGD2 | ***CELF2*** | NEMF | | | | RS10 | | | | | | | | | | | | |
| | FGD3 | ***CENPF*** | ***NEURL2*** | | | | RS13 | | | | | | | | | | | | |
| | FGF14 | CENPH | NFKBIE | | | | RS14 | | | | | | | | | | | | |
| | FGF7 | CENPI | NFKBIL1 | | | | RS15A | | | | | | | | | | | | |
| | FHAD1 | CENPN | ***NID2*** | | | | RS16 | | | | | | | | | | | | |
| | FHL2 | CENPT | ***NKD2*** | | | | RS18 | | | | | | | | | | | | |
| | FLJ22184 | CENPU | ***NLRC3*** | | | | RS19 | | | | | | | | | | | | |
| | ***FLT1*** | CEP128 | NOA1 | | | | RS2 | | | | | | | | | | | | |
| | FMNL2 | CEP57L1 | ***NOP58*** | | | | RS20 | | | | | | | | | | | | |
| | FMOD | CEP68 | ***NOS2*** | | | | RS25 | | | | | | | | | | | | |
| | ***FN1*** | CEP72 | ***NOTCH2*** | | | | RS3 | | | | | | | | | | | | |
| | ***FNDC1*** | ***CERK*** | ***NPRL3*** | | | | RS4X | | | | | | | | | | | | |
| | FOXL1 | CES2B | ***NQO1*** | | | | RS7 | | | | | | | | | | | | |
| | FPR3 | CES2E | ***NRROS*** | | | | RS8 | | | | | | | | | | | | |

| | | | | | | | |
|---|---|---|---|---|---|---|---|
| | ***FRMD6*** | CES2G | NSRP1 | | | | RS9 |
| | FSTL1 | ***CFAP43*** | ***OAF*** | | | | RSMB |
| | FUCA1 | CFAP45 | OAZ1 | | | | RSMN |
| | ***FUT4*** | CFAP57 | ODF2L | | | | RSSA |
| | ***FUT8*** | ***CFTR*** | OLFM2 | | | | RTN4 |
| | FXYD7 | ***CGNL1*** | OPHN1 | | | | S10A6 |
| | ***FZD1*** | ***CHAC1*** | ***P3H3*** | | | | S10AA |
| | GABBR2 | CHAF1A | PACSIN3 | | | | S10AB |
| | GABRA4 | CHAF1B | ***PAK4*** | | | | S10AG |
| | GABRA5 | CHD3 | ***PALM*** | | | | SAHH |
| | GABRB2 | CHD7 | PAPSS2 | | | | ***SDPR*** |
| | GABRE | CHIC1 | ***PAQR7*** | | | | SEPT2 |
| | GABRG2 | CHIC2 | PATZ1 | | | | SERPH |
| | GALNT10 | CHMP4C | ***PCDHGA5*** | | | | SET |
| | GALNT5 | ***CHN2*** | ***PDCD10*** | | | | ***SFPQ*** |
| | GALNT9 | CHRAC1 | PDE8A | | | | SH3L3 |
| | GAS1 | ***CHRD*** | PDK4 | | | | SPTA2 |
| | GAS7 | CHRNB1 | ***PDLIM2*** | | | | SPTB2 |
| | GATA6 | ***CHST11*** | ***PECAM1*** | | | | SSRD |
| | ***GBP5*** | CHST12 | ***PGGHG*** | | | | STAT1 |
| | GCNT1 | CHST15 | ***PGM5*** | | | | STIP1 |
| | GDI1 | ***CHST7*** | ***PHACTR1*** | | | | SYWC |
| | ***GDPD5*** | CHTF18 | ***PHLDB1*** | | | | TAGL |
| | ***GIT1*** | ***CIT*** | PIGP | | | | TAGL2 |
| | GLI2 | CKAP2 | ***PIH1D1*** | | | | TALDO |
| | GM2A | ***CKAP2L*** | ***PIK3R6*** | | | | TBA1A |
| | GMIP | ***CKB*** | ***PINK1*** | | | | TBA1B |
| | ***GNAO1*** | ***CLCN3*** | ***PITPNM3*** | | | | TBB2A |
| | GPNMB | CLCN4 | ***PLEC*** | | | | TBB2B |
| | GPR126 | CLCN5 | ***PLEKHM1*** | | | | TBB4A |
| | GPR132 | CLDN1 | ***PLOD1*** | | | | TBB4B |
| | GPR39 | CLDN12 | ***PLPP3*** | | | | TBB5 |
| | GPRIN3 | CLDN6 | PLXND1 | | | | TBB6 |
| | GRB10 | CLGN | ***PMP22*** | | | | TCPA |
| | GRIA2 | ***CLIC4*** | ***PNRC1*** | | | | TCPB |
| | GRIK2 | CLIC5 | ***PODXL*** | | | | TCPD |
| | GRIK3 | CLIC6 | ***PPP2R1B*** | | | | TCPE |
| | GRIN1 | CLSPN | ***PPP2R3A*** | | | | TCPG |
| | GRM5 | ***CLTB*** | PPT2 | | | | TCPH |
| | GRM7 | CMAS | ***PTCH1*** | | | | TCPQ |
| | GVINP1 | CMBL | ***PTMA*** | | | | TCPZ |
| | GXYLT1 | ***CMIP*** | PTMS | | | | TERA |
| | GYPC | ***CMKLR1*** | PTPN18 | | | | ***TGM2*** |
| | HAVCR2 | CNGB1 | ***PTPRJ*** | | | | THIL |
| | HCLS1 | CNN1 | ***PTPRR*** | | | | THIO |
| | HDC | CNNM4 | ***PTPRU*** | | | | TINAL |
| | HECTD4 | CNP | PURG | | | | TITIN |
| | HEPHL1 | CNST | ***PWWP2B*** | | | | TKT |
| | HGF | CNTLN | ***PXDN*** | | | | TLN1 |
| | ***HIPK2*** | CNTROB | PYCARD | | | | TOIP1 |
| | HIST1H1C | ***COL15A1*** | QK | | | | TPIS |
| | HK2 | COL18A1 | ***RAB11A*** | | | | ***TPM2*** |
| | HK3 | COL20A1 | ***RALGPS2*** | | | | ***TPM3*** |
| | HLA-DPB1 | ***COL27A1*** | ***RAPGEF1*** | | | | TPM3L |
| | HMCN1 | COL28A1 | ***RAPGEF4*** | | | | ***TPM4*** |
| | HMHA1 | COL4A3 | ***RASGRP2*** | | | | TPP1 |
| | HMOX1 | COL5A3 | RASSF1 | | | | TRI25 |
| | HPCA | ***COLEC12*** | ***RASSF2*** | | | | TRXR1 |
| | HS6ST3 | CORO1B | ***RASSF4*** | | | | TSP1 |
| | HSD17B4 | CORO2A | RBM28 | | | | TXND5 |
| | HSH2D | CORO2B | ***RCBTB2*** | | | | TYB4 |
| | HTR7 | COX6C | ***RCC2*** | | | | UB2L3 |
| | HTRA3 | CP | ***RDH12*** | | | | UBA1 |
| | IBSP | ***CPE*** | ***RHPN2*** | | | | UBE2N |
| | IDS | CPEB1 | ***RNF144A*** | | | | UE2NL |
| | IFI30 | ***CPM*** | RNF144B | | | | VAT1 |
| | ***IFITM10*** | ***CPNE2*** | RNF149 | | | | VDAC1 |
| | IGF1 | CPNE7 | ***RPL21*** | | | | VDAC2 |
| | IGF2 | CPNE8 | ***RPS18*** | | | | VIME |
| | IGFBP2 | ***CRCT1*** | RRP15 | | | | VINC |
| | IGFBP5 | ***CREB3L2*** | ***RTP3*** | | | | VPS35 |
| | IGFN1 | CREB5 | ***S100A10*** | | | | ***VWF*** |
| | IGJ | ***CRIM1*** | ***S100A7A*** | | | | WDR1 |
| | IGLL5 | ***CRIP1*** | ***SAMD4B*** | | | | XPO2 |
| | IKBIP | CRIP2 | ***SAMD5*** | | | | ***XRCC5*** |
| | IKZF1 | CRYAB | SBDS | | | | YBOX1 |
| | IKZF3 | CRYL1 | ***SCAP*** | | | | ***ZYX*** |
| | IL10RA | CSRNP1 | SCARF1 | | | | |
| | IL16 | CSRP1 | ***SCMH1*** | | | | |
| | IL1R2 | ***CSRP2*** | ***SCUBE3*** | | | | |
| | ***IL20RB*** | CST6 | ***SEC14L1*** | | | | |
| | IL21R | CTBP2 | ***SELENBP1*** | | | | |
| | IL2RA | CTDP1 | ***SEPT1*** | | | | |
| | IL2RB | ***CTGF*** | ***SEPT4*** | | | | |
| | IL7R | CTIF | SERINC3 | | | | |
| | INA | CTLA2A | SERPINB8 | | | | |
| | INSRR | CTLA2B | ***SESN3*** | | | | |
| | ***IQGAP2*** | CTNNA1 | ***SETX*** | | | | |
| | IQSEC1 | CTNNBIP1 | ***SFT2D2*** | | | | |
| | IRF4 | CTNND2 | ***SH3PXD2B*** | | | | |
| | IRF5 | ***CTSC*** | ***SIPA1*** | | | | |
| | ***IRF8*** | CTSD | ***SIX1*** | | | | |
| | ISLR | ***CTSL*** | ***SIX4*** | | | | |
| | ITGA11 | CTTNBP2 | SKA1 | | | | |
| | ITGA4 | CUEDC1 | SLC16A9 | | | | |
| | ITGAL | CUL7 | SLC22A23 | | | | |
| | ITGAM | ***CX3CL1*** | ***SLC23A2*** | | | | |
| | ITGAX | ***CXCR4*** | SLC25A11 | | | | |
| | ITGB2 | CXX1A | ***SLC38A5*** | | | | |
| | ITGB5 | CXX1B | ***SLC39A4*** | | | | |
| | ITGBL1 | CXX1C | ***SLC39A8*** | | | | |
| | ITIH2 | ***CYB5R1*** | ***SLC43A2*** | | | | |
| | ***ITM2C*** | CYCS | ***SLC9A3R2*** | | | | |
| | ***ITPR1*** | ***CYFIP1*** | ***SLK*** | | | | |
| | ***JAG2*** | CYLD | ***SMAP2*** | | | | |
| | JAKMIP3 | CYP1A1 | SMIM10L1 | | | | |
| | JPH3 | CYP4B1 | ***SMO*** | | | | |
| | JPH4 | ***CYP4V3*** | ***SNAP29*** | | | | |
| | KCNA1 | CYR61 | ***SNCG*** | | | | |
| | KCNC2 | CYSLTR1 | ***SNED1*** | | | | |
| | KCNH3 | CYTH1 | ***SNX2*** | | | | |
| | KCNIP4-IT1 | ***CYTH3*** | ***SOCS5*** | | | | |
| | KCNJ5 | ***CYTH4*** | ***SOD3*** | | | | |
| | ***KCNK6*** | D1ERTD622E | ***SOX4*** | | | | |
| | KCNQ1OT1 | D3ERTD751E | SPECC1L | | | | |
| | KCNQ2 | D430042O09RIK | SPRY1 | | | | |
| | KCNT1 | ***D930015E06RIK*** | ***SPTAN1*** | | | | |
| | ***KCTD12*** | DAB2IP | ***SPTBN1*** | | | | |
| | KHDRBS2 | ***DACH1*** | ***SPTSSA*** | | | | |
| | KIAA0125 | DAGLA | SQLE | | | | |
| | KIAA0226L | DAPK1 | ***SRGN*** | | | | |
| | KIAA0513 | ***DAPK2*** | ***SRMS*** | | | | |
| | KIAA1107 | DARS | ***ST14*** | | | | |

| | | | | | | | | | | | | | | | | | | | |
|---|---|---|---|---|---|---|---|---|---|---|---|---|---|---|---|---|---|---|---|
| | KIAA1109 | DBN1 | ***STK10*** | | | | | | | | | | | | | | | | |
| | KIAA1211 | DBNDD2 | ***SUN1*** | | | | | | | | | | | | | | | | |
| | KIAA1244 | DCAF11 | ***SWAP70*** | | | | | | | | | | | | | | | | |
| | KIAA1755 | DCAF4 | ***SYDE1*** | | | | | | | | | | | | | | | | |
| | KIDINS220 | ***DCBLD2*** | ***SYK*** | | | | | | | | | | | | | | | | |
| | KIF1A | DCLRE1A | ***SYNE3*** | | | | | | | | | | | | | | | | |
| | KIF1B | DCTN1 | TAF6L | | | | | | | | | | | | | | | | |
| | KIF3C | DCUN1D3 | TAPBP | | | | | | | | | | | | | | | | |
| | KIF5A | DCUN1D5 | TCF3 | | | | | | | | | | | | | | | | |
| | KIF5C | DDIT4 | TCF7L1 | | | | | | | | | | | | | | | | |
| | KIRREL | DDX39 | ***TEAD4*** | | | | | | | | | | | | | | | | |
| | ***KLHL6*** | DEK | ***TGFBI*** | | | | | | | | | | | | | | | | |
| | KPRP | DEPDC1B | TIMM23 | | | | | | | | | | | | | | | | |
| | KRT6A | DGKA | ***TIMP1*** | | | | | | | | | | | | | | | | |
| | ***LAMA4*** | DGKD | ***TIMP4*** | | | | | | | | | | | | | | | | |
| | ***LAMB1*** | DGKE | ***TINAGL1*** | | | | | | | | | | | | | | | | |
| | ***LAMC3*** | DGKK | ***TMEM120B*** | | | | | | | | | | | | | | | | |
| | LAMP3 | DHCR24 | ***TMEM123*** | | | | | | | | | | | | | | | | |
| | LANCL1 | ***DHH*** | TMEM158 | | | | | | | | | | | | | | | | |
| | LAX1 | ***DHRS3*** | ***TMEM173*** | | | | | | | | | | | | | | | | |
| | LCE3D | ***DHRS7B*** | ***TMEM176A*** | | | | | | | | | | | | | | | | |
| | ***LCK*** | DHX16 | ***TMEM176B*** | | | | | | | | | | | | | | | | |
| | ***LCP1*** | DIAPH3 | TMEM231 | | | | | | | | | | | | | | | | |
| | ***LCP2*** | DIP2B | TMEM258 | | | | | | | | | | | | | | | | |
| | LDB2 | ***DIP2C*** | ***TMEM47*** | | | | | | | | | | | | | | | | |
| | LGMN | DLG1 | TMEM55A | | | | | | | | | | | | | | | | |
| | ***LHFPL2*** | ***DLG4*** | TMEM55B | | | | | | | | | | | | | | | | |
| | LILRB4 | ***DLL1*** | ***TNFAIP2*** | | | | | | | | | | | | | | | | |
| | LIMD1 | ***DLL4*** | TNFAIP8 | | | | | | | | | | | | | | | | |
| | LINC00861 | DMD | TNFAIP8L1 | | | | | | | | | | | | | | | | |
| | LINC00886 | DNA2 | ***TNFRSF21*** | | | | | | | | | | | | | | | | |
| | LINC00959 | DNAAF2 | ***TNFRSF8*** | | | | | | | | | | | | | | | | |
| | LINC01094 | DNAH10 | TNFSF12 | | | | | | | | | | | | | | | | |
| | LINC01279 | DNAH6 | ***TNS2*** | | | | | | | | | | | | | | | | |
| | LINGO1 | DNAJB4 | TNXB | | | | | | | | | | | | | | | | |
| | ***LMTK2*** | ***DNAJB6*** | TOR1B | | | | | | | | | | | | | | | | |
| | LOC100506585 | DNAJC10 | TOR4A | | | | | | | | | | | | | | | | |
| | LOC102724814 | DNAJC21 | ***TPST1*** | | | | | | | | | | | | | | | | |
| | LOC389834 | ***DNM3*** | TRAPPC6B | | | | | | | | | | | | | | | | |
| | ***LONRF2*** | DNMT1 | ***TRF*** | | | | | | | | | | | | | | | | |
| | LOX | DOCK4 | ***TRIB1*** | | | | | | | | | | | | | | | | |
| | LOXL1 | ***DOCK8*** | ***TRIB3*** | | | | | | | | | | | | | | | | |
| | LOXL3 | DOK3 | ***TRP53INP2*** | | | | | | | | | | | | | | | | |
| | ***LOXL4*** | ***DOK4*** | ***TSC2*** | | | | | | | | | | | | | | | | |
| | LPHN1 | ***DPAGT1*** | TSG101 | | | | | | | | | | | | | | | | |
| | LRRC15 | DPYSL3 | TSPAN13 | | | | | | | | | | | | | | | | |
| | LRRTM3 | DTL | TSPAN15 | | | | | | | | | | | | | | | | |
| | LSAMP | DTYMK | TSPAN31 | | | | | | | | | | | | | | | | |
| | LSP1 | DUSP1 | ***TSPAN7*** | | | | | | | | | | | | | | | | |
| | ***LTB4R*** | DUSP10 | ***TSPYL4*** | | | | | | | | | | | | | | | | |
| | ***LTBP2*** | DUSP18 | ***TSSC4*** | | | | | | | | | | | | | | | | |
| | ***LTBP4*** | DUSP23 | TTC37 | | | | | | | | | | | | | | | | |
| | ***LTF*** | DUSP7 | TTYH3 | | | | | | | | | | | | | | | | |
| | LUM | ***DYRK1B*** | ***TUBB2A*** | | | | | | | | | | | | | | | | |
| | LYNX1 | DYRK2 | ***TXNDC5*** | | | | | | | | | | | | | | | | |
| | LYZ | ***DYSF*** | ***UBE2G1*** | | | | | | | | | | | | | | | | |
| | LZTS3 | E130308A19RIK | UBL7 | | | | | | | | | | | | | | | | |
| | MACC1 | ***E2F2*** | ***UBQLN2*** | | | | | | | | | | | | | | | | |
| | ***MAFB*** | E2F7 | UGDH | | | | | | | | | | | | | | | | |
| | MAGI2 | E2F8 | VCAM1 | | | | | | | | | | | | | | | | |
| | MAN1A2 | ECM1 | ***VIL1*** | | | | | | | | | | | | | | | | |
| | MAN2B1 | EDA2R | VPS13D | | | | | | | | | | | | | | | | |
| | ***MAP1A*** | ***EDN1*** | VPS72 | | | | | | | | | | | | | | | | |
| | ***MAP2*** | EEF2K | ***VSTM5*** | | | | | | | | | | | | | | | | |
| | MAP7D2 | EFCAB14 | WBP2 | | | | | | | | | | | | | | | | |
| | MAPK10 | EFEMP2 | ***YBX1*** | | | | | | | | | | | | | | | | |
| | MAPK7 | ***EFHD2*** | ZBTB45 | | | | | | | | | | | | | | | | |
| | MAPK8IP1 | EFNA3 | ***ZBTB46*** | | | | | | | | | | | | | | | | |
| | MAPK8IP2 | EFNA4 | ZC3H7A | | | | | | | | | | | | | | | | |
| | MAPRE2 | EFNA5 | ***ZFP28*** | | | | | | | | | | | | | | | | |
| | MARCO | EFNB2 | ***ZFP366*** | | | | | | | | | | | | | | | | |
| | ***MASP1*** | EFR3B | ZFP407 | | | | | | | | | | | | | | | | |
| | MAST3 | EGFL8 | ***ZFP521*** | | | | | | | | | | | | | | | | |
| | ***MATN2*** | EGLN3 | ***ZFP697*** | | | | | | | | | | | | | | | | |
| | MCF2L | EHBP1L1 | ***ZFP703*** | | | | | | | | | | | | | | | | |
| | ***MCM2*** | ***EHD1*** | ZFP706 | | | | | | | | | | | | | | | | |
| | MDFIC | EHD2 | ZFP715 | | | | | | | | | | | | | | | | |
| | ***MDGA1*** | ***EHD4*** | ***ZFP787*** | | | | | | | | | | | | | | | | |
| | MDGA2 | EIF2B2 | ZNHIT6 | | | | | | | | | | | | | | | | |
| | MEF2C | EIF5 | ***ZNRF1*** | | | | | | | | | | | | | | | | |
| | MEG3 | ***ELF4*** | | | | | | | | | | | | | | | | | |
| | MEGF6 | ELMO2 | | | | | | | | | | | | | | | | | |
| | MGAT3 | ***ELN*** | | | | | | | | | | | | | | | | | |
| | MGAT5B | ELOVL4 | | | | | | | | | | | | | | | | | |
| | MGC27345 | ELOVL5 | | | | | | | | | | | | | | | | | |
| | MGEA5 | ***EMP1*** | | | | | | | | | | | | | | | | | |
| | MICU3 | EMP2 | | | | | | | | | | | | | | | | | |
| | MIR3648 | ***ENDOU*** | | | | | | | | | | | | | | | | | |
| | ***MKI67*** | ENHO | | | | | | | | | | | | | | | | | |
| | MLPH | ***ENPP2*** | | | | | | | | | | | | | | | | | |
| | ***MMP14*** | ***ENPP6*** | | | | | | | | | | | | | | | | | |
| | ***MMP2*** | ***ENTPD1*** | | | | | | | | | | | | | | | | | |
| | MMP9 | ENTPD5 | | | | | | | | | | | | | | | | | |
| | MOBP | ***EPAS1*** | | | | | | | | | | | | | | | | | |
| | MPEG1 | EPB41 | | | | | | | | | | | | | | | | | |
| | MR1 | EPB41L2 | | | | | | | | | | | | | | | | | |
| | MRC2 | ***EPHA2*** | | | | | | | | | | | | | | | | | |
| | MS4A6A | EPHA7 | | | | | | | | | | | | | | | | | |
| | MSC | EPHB4 | | | | | | | | | | | | | | | | | |
| | MSR1 | EPHX1 | | | | | | | | | | | | | | | | | |
| | MT3 | EPS8L2 | | | | | | | | | | | | | | | | | |
| | MTRNR2L1 | ERCC6L | | | | | | | | | | | | | | | | | |
| | MTURN | ERGIC1 | | | | | | | | | | | | | | | | | |
| | MUC17 | ERI1 | | | | | | | | | | | | | | | | | |
| | MUC3A | ***ERN1*** | | | | | | | | | | | | | | | | | |
| | MUC6 | ESPL1 | | | | | | | | | | | | | | | | | |
| | MXRA5 | ESR1 | | | | | | | | | | | | | | | | | |
| | MYCBPAP | ETHE1 | | | | | | | | | | | | | | | | | |
| | ***MYO7A*** | ETV6 | | | | | | | | | | | | | | | | | |
| | MYO9A | EVI5L | | | | | | | | | | | | | | | | | |
| | MYOF | EXOC3L2 | | | | | | | | | | | | | | | | | |
| | MYT1L | ***EXT1*** | | | | | | | | | | | | | | | | | |
| | NABP1 | EXTL2 | | | | | | | | | | | | | | | | | |
| | NAPB | EZH2 | | | | | | | | | | | | | | | | | |
| | NBEA | EZR | | | | | | | | | | | | | | | | | |
| | ***NCAPG*** | ***F2R*** | | | | | | | | | | | | | | | | | |
| | NCDN | ***F2RL2*** | | | | | | | | | | | | | | | | | |
| | NCF2 | FAAP100 | | | | | | | | | | | | | | | | | |
| | NCKAP1 | FAAP20 | | | | | | | | | | | | | | | | | |
| | NCKAP1L | ***FADS2*** | | | | | | | | | | | | | | | | | |
| | NCS1 | FAH | | | | | | | | | | | | | | | | | |
| | ***NDFIP1*** | FAM102B | | | | | | | | | | | | | | | | | |
| | ***NDNF*** | ***FAM107A*** | | | | | | | | | | | | | | | | | |

| | | | | | | | | | | | | | | | | | | | |
|---|---|---|---|---|---|---|---|---|---|---|---|---|---|---|---|---|---|---|---|
| | NDRG2 | FAM110C | | | | | | | | | | | | | | | | | |
| | ***NDRG4*** | FAM111A | | | | | | | | | | | | | | | | | |
| | NECAB1 | ***FAM117A*** | | | | | | | | | | | | | | | | | |
| | NEFL | FAM117B | | | | | | | | | | | | | | | | | |
| | NEFM | FAM118A | | | | | | | | | | | | | | | | | |
| | NFAM1 | FAM122B | | | | | | | | | | | | | | | | | |
| | NFASC | ***FAM129A*** | | | | | | | | | | | | | | | | | |
| | NFATC2 | ***FAM129B*** | | | | | | | | | | | | | | | | | |
| | NFIX | FAM13A | | | | | | | | | | | | | | | | | |
| | ***NGEF*** | FAM13B | | | | | | | | | | | | | | | | | |
| | NGRN | ***FAM160A1*** | | | | | | | | | | | | | | | | | |
| | ***NID2*** | FAM160B1 | | | | | | | | | | | | | | | | | |
| | NINL | FAM167B | | | | | | | | | | | | | | | | | |
| | NLGN1 | FAM171A2 | | | | | | | | | | | | | | | | | |
| | ***NLRC3*** | FAM174B | | | | | | | | | | | | | | | | | |
| | NLRC5 | ***FAM189A2*** | | | | | | | | | | | | | | | | | |
| | NLRP3 | ***FAM20A*** | | | | | | | | | | | | | | | | | |
| | ***NMNAT2*** | ***FAM20C*** | | | | | | | | | | | | | | | | | |
| | NNMT | FAM212A | | | | | | | | | | | | | | | | | |
| | ***NOTCH2*** | FAM214B | | | | | | | | | | | | | | | | | |
| | NOTCH2NL | FAM217A | | | | | | | | | | | | | | | | | |
| | ***NPC1L1*** | FAM229B | | | | | | | | | | | | | | | | | |
| | NPTX1 | ***FAM49A*** | | | | | | | | | | | | | | | | | |
| | NPTXR | FAM57A | | | | | | | | | | | | | | | | | |
| | NR1H3 | FAM63A | | | | | | | | | | | | | | | | | |
| | ***NR1H4*** | FAM64A | | | | | | | | | | | | | | | | | |
| | ***NRGN*** | FAM65A | | | | | | | | | | | | | | | | | |
| | ***NRP1*** | FAM65B | | | | | | | | | | | | | | | | | |
| | ***NRP2*** | FAM69B | | | | | | | | | | | | | | | | | |
| | NRXN1 | FAM84B | | | | | | | | | | | | | | | | | |
| | NT5E | FANCA | | | | | | | | | | | | | | | | | |
| | NTRK1 | FANCD2 | | | | | | | | | | | | | | | | | |
| | NUGGC | FANCI | | | | | | | | | | | | | | | | | |
| | NUP210L | FAR2 | | | | | | | | | | | | | | | | | |
| | OGDHL | ***FAS*** | | | | | | | | | | | | | | | | | |
| | OLFM1 | ***FAT1*** | | | | | | | | | | | | | | | | | |
| | OLFM3 | FAT4 | | | | | | | | | | | | | | | | | |
| | OLFML2B | ***FAXC*** | | | | | | | | | | | | | | | | | |
| | OLFML3 | FBLIM1 | | | | | | | | | | | | | | | | | |
| | OPCML | FBLN5 | | | | | | | | | | | | | | | | | |
| | OSBPL1A | FBXL22 | | | | | | | | | | | | | | | | | |
| | OSBPL3 | FBXL7 | | | | | | | | | | | | | | | | | |
| | OXR1 | FBXO32 | | | | | | | | | | | | | | | | | |
| | OXTR | FBXO34 | | | | | | | | | | | | | | | | | |
| | PACSIN1 | FBXO38 | | | | | | | | | | | | | | | | | |
| | PAK3 | FBXW2 | | | | | | | | | | | | | | | | | |
| | PALLD | FBXW8 | | | | | | | | | | | | | | | | | |
| | PAPPA | FCGRT | | | | | | | | | | | | | | | | | |
| | PARVA | FCHSD1 | | | | | | | | | | | | | | | | | |
| | PAX5 | FES | | | | | | | | | | | | | | | | | |
| | PBLD | FGFBP1 | | | | | | | | | | | | | | | | | |
| | PCDH9 | FGFR1 | | | | | | | | | | | | | | | | | |
| | PCDHB7 | ***FGFR3*** | | | | | | | | | | | | | | | | | |
| | PCDHGA1 | ***FHL3*** | | | | | | | | | | | | | | | | | |
| | PCK2 | FIBP | | | | | | | | | | | | | | | | | |
| | PCOLCE | FIGNL1 | | | | | | | | | | | | | | | | | |
| | PCSK1N | FILIP1L | | | | | | | | | | | | | | | | | |
| | ***PDGFRA*** | FKBP14 | | | | | | | | | | | | | | | | | |
| | ***PDLIM2*** | ***FKBP4*** | | | | | | | | | | | | | | | | | |
| | PDLIM4 | FKBP5 | | | | | | | | | | | | | | | | | |
| | PDPN | FLCN | | | | | | | | | | | | | | | | | |
| | PDXP | ***FLNA*** | | | | | | | | | | | | | | | | | |
| | ***PDZD2*** | ***FLT1*** | | | | | | | | | | | | | | | | | |
| | PDZD4 | FLT3 | | | | | | | | | | | | | | | | | |
| | ***PHACTR1*** | FLT3L | | | | | | | | | | | | | | | | | |
| | ***PHLDB1*** | FMO1 | | | | | | | | | | | | | | | | | |
| | PHYHIP | ***FN1*** | | | | | | | | | | | | | | | | | |
| | PI4KA | ***FNDC1*** | | | | | | | | | | | | | | | | | |
| | ***PIK3CG*** | FOSB | | | | | | | | | | | | | | | | | |
| | ***PIK3R5*** | FOSL1 | | | | | | | | | | | | | | | | | |
| | PIN1 | FOSL2 | | | | | | | | | | | | | | | | | |
| | ***PINK1*** | FOXF2 | | | | | | | | | | | | | | | | | |
| | PKD1L1 | FOXM1 | | | | | | | | | | | | | | | | | |
| | PKD1L3 | ***FOXO1*** | | | | | | | | | | | | | | | | | |
| | PLA2G7 | FOXP4 | | | | | | | | | | | | | | | | | |
| | PLAGL1 | FOXQ1 | | | | | | | | | | | | | | | | | |
| | PLAU | FRAT2 | | | | | | | | | | | | | | | | | |
| | ***PLAUR*** | ***FRMD6*** | | | | | | | | | | | | | | | | | |
| | PLB1 | ***FRZB*** | | | | | | | | | | | | | | | | | |
| | PLBD1 | ***FSCN1*** | | | | | | | | | | | | | | | | | |
| | PLCB2 | ***FUT4*** | | | | | | | | | | | | | | | | | |
| | ***PLCD3*** | ***FUT8*** | | | | | | | | | | | | | | | | | |
| | ***PLEK*** | ***FXYD5*** | | | | | | | | | | | | | | | | | |
| | PLEKHA4 | FXYD6 | | | | | | | | | | | | | | | | | |
| | PLEKHG3 | ***FYN*** | | | | | | | | | | | | | | | | | |
| | PLEKHG4B | ***FZD1*** | | | | | | | | | | | | | | | | | |
| | PLEKHO2 | FZD4 | | | | | | | | | | | | | | | | | |
| | ***PLTP*** | FZD6 | | | | | | | | | | | | | | | | | |
| | ***PLXDC2*** | G3BP1 | | | | | | | | | | | | | | | | | |
| | PML | G6PDX | | | | | | | | | | | | | | | | | |
| | PNMA2 | GAB1 | | | | | | | | | | | | | | | | | |
| | PNMAL2 | ***GABARAPL1*** | | | | | | | | | | | | | | | | | |
| | POSTN | ***GADD45A*** | | | | | | | | | | | | | | | | | |
| | PPFIA2 | GADD45G | | | | | | | | | | | | | | | | | |
| | PPP1R12A | GALC | | | | | | | | | | | | | | | | | |
| | PPP1R16B | GARS | | | | | | | | | | | | | | | | | |
| | PPP1R3B | GATM | | | | | | | | | | | | | | | | | |
| | PPP2R2C | GATSL3 | | | | | | | | | | | | | | | | | |
| | ***PPP3CA*** | ***GBP4*** | | | | | | | | | | | | | | | | | |
| | ***PPP3R1*** | ***GBP5*** | | | | | | | | | | | | | | | | | |
| | PRKAR1B | ***GDPD5*** | | | | | | | | | | | | | | | | | |
| | PRKCB | GEN1 | | | | | | | | | | | | | | | | | |
| | PRRX1 | GFOD1 | | | | | | | | | | | | | | | | | |
| | PSD | GFPT1 | | | | | | | | | | | | | | | | | |
| | PSD4 | GFPT2 | | | | | | | | | | | | | | | | | |
| | ***PSIP1*** | GGPS1 | | | | | | | | | | | | | | | | | |
| | PSRC1 | GIMAP4 | | | | | | | | | | | | | | | | | |
| | PTAFR | GINS2 | | | | | | | | | | | | | | | | | |
| | ***PTGFRN*** | ***GIT1*** | | | | | | | | | | | | | | | | | |
| | PTGIS | GJA1 | | | | | | | | | | | | | | | | | |
| | ***PTGS1*** | GJA4 | | | | | | | | | | | | | | | | | |
| | PTK2 | GJC1 | | | | | | | | | | | | | | | | | |
| | PTK7 | GLIPR2 | | | | | | | | | | | | | | | | | |
| | PTP4A3 | GLIS3 | | | | | | | | | | | | | | | | | |
| | PTPN6 | GLRX2 | | | | | | | | | | | | | | | | | |
| | ***PTPN7*** | GLTP | | | | | | | | | | | | | | | | | |
| | PTPRC | ***GM525*** | | | | | | | | | | | | | | | | | |
| | PTPRE | GM684 | | | | | | | | | | | | | | | | | |
| | ***PTPRK*** | GM694 | | | | | | | | | | | | | | | | | |
| | PTPRT | GMFG | | | | | | | | | | | | | | | | | |
| | PVRL4 | GNA11 | | | | | | | | | | | | | | | | | |
| | R3HDM1 | ***GNA12*** | | | | | | | | | | | | | | | | | |
| | ***RAB3A*** | GNA14 | | | | | | | | | | | | | | | | | |

| | | | | | | | | | | | | | | | | | | | |
|---|---|---|---|---|---|---|---|---|---|---|---|---|---|---|---|---|---|---|---|
| | RAB6B | ***GNAO1*** | | | | | | | | | | | | | | | | | |
| | RAI14 | ***GNB4*** | | | | | | | | | | | | | | | | | |
| | RAP1GAP | GNG11 | | | | | | | | | | | | | | | | | |
| | RAP2A | ***GNG2*** | | | | | | | | | | | | | | | | | |
| | RAP2B | ***GNG5*** | | | | | | | | | | | | | | | | | |
| | RAPGEF2 | GOLGA4 | | | | | | | | | | | | | | | | | |
| | ***RAPGEF3*** | GOLM1 | | | | | | | | | | | | | | | | | |
| | RASAL3 | GPAT3 | | | | | | | | | | | | | | | | | |
| | RASSF5 | GPATCH4 | | | | | | | | | | | | | | | | | |
| | RBFOX1 | GPC4 | | | | | | | | | | | | | | | | | |
| | RBFOX2 | GPD2 | | | | | | | | | | | | | | | | | |
| | RBFOX3 | GPM6A | | | | | | | | | | | | | | | | | |
| | RBM47 | GPR137B | | | | | | | | | | | | | | | | | |
| | ***RBP1*** | GPR143 | | | | | | | | | | | | | | | | | |
| | RCAN2 | GPR146 | | | | | | | | | | | | | | | | | |
| | ***RCN1*** | GPR157 | | | | | | | | | | | | | | | | | |
| | RCN3 | GPR160 | | | | | | | | | | | | | | | | | |
| | RDH10 | GPR176 | | | | | | | | | | | | | | | | | |
| | RGS7 | GPR55 | | | | | | | | | | | | | | | | | |
| | RIMS1 | GPRC5A | | | | | | | | | | | | | | | | | |
| | RIMS2 | GPSM1 | | | | | | | | | | | | | | | | | |
| | RIMS3 | GPX3 | | | | | | | | | | | | | | | | | |
| | RIMS4 | GPX7 | | | | | | | | | | | | | | | | | |
| | ***RIN3*** | GPX8 | | | | | | | | | | | | | | | | | |
| | ***RPS6KA2*** | GRAMD1B | | | | | | | | | | | | | | | | | |
| | RTN1 | GRAMD3 | | | | | | | | | | | | | | | | | |
| | RTN3 | ***GRAMD4*** | | | | | | | | | | | | | | | | | |
| | RUNX1 | ***GRAP*** | | | | | | | | | | | | | | | | | |
| | RUNX2 | ***GRASP*** | | | | | | | | | | | | | | | | | |
| | S100A11 | GRRP1 | | | | | | | | | | | | | | | | | |
| | ***S1PR1*** | ***GSDMD*** | | | | | | | | | | | | | | | | | |
| | SAMD11 | GSE1 | | | | | | | | | | | | | | | | | |
| | SAMD4A | GSTA4 | | | | | | | | | | | | | | | | | |
| | SARDH | GSTK1 | | | | | | | | | | | | | | | | | |
| | SATB2 | ***GSTO1*** | | | | | | | | | | | | | | | | | |
| | SBF1 | H1F0 | | | | | | | | | | | | | | | | | |
| | SBSN | H2-Q6 | | | | | | | | | | | | | | | | | |
| | SCARA5 | H2-Q7 | | | | | | | | | | | | | | | | | |
| | SCN2A | H2-T23 | | | | | | | | | | | | | | | | | |
| | SCN3A | HAGH | | | | | | | | | | | | | | | | | |
| | SCUBE1 | HAPLN3 | | | | | | | | | | | | | | | | | |
| | ***SCUBE3*** | HAUS2 | | | | | | | | | | | | | | | | | |
| | ***SDC1*** | HBP1 | | | | | | | | | | | | | | | | | |
| | SEL1L3 | HCAR1 | | | | | | | | | | | | | | | | | |
| | SELL | HCN2 | | | | | | | | | | | | | | | | | |
| | ***SELP*** | HDAC9 | | | | | | | | | | | | | | | | | |
| | ***SEMA3C*** | HDGF | | | | | | | | | | | | | | | | | |
| | SEMA4A | ***HEATR5A*** | | | | | | | | | | | | | | | | | |
| | SEPT3 | HEBP2 | | | | | | | | | | | | | | | | | |
| | ***SEPT4*** | ***HECW2*** | | | | | | | | | | | | | | | | | |
| | SERPINE1 | ***HEG1*** | | | | | | | | | | | | | | | | | |
| | SFRP2 | HELLS | | | | | | | | | | | | | | | | | |
| | SFRP4 | HEPH | | | | | | | | | | | | | | | | | |
| | SGCD | HES6 | | | | | | | | | | | | | | | | | |
| | ***SH2B3*** | HEXIM1 | | | | | | | | | | | | | | | | | |
| | SH3BP2 | HEY1 | | | | | | | | | | | | | | | | | |
| | SH3GL3 | HHIPL2 | | | | | | | | | | | | | | | | | |
| | SH3RF3 | ***HID1*** | | | | | | | | | | | | | | | | | |
| | SHANK1 | HIF1A | | | | | | | | | | | | | | | | | |
| | SHC3 | HIP1R | | | | | | | | | | | | | | | | | |
| | SIGLEC1 | ***HIPK2*** | | | | | | | | | | | | | | | | | |
| | SIK3 | HIVEP2 | | | | | | | | | | | | | | | | | |
| | SLA | HJURP | | | | | | | | | | | | | | | | | |
| | SLAMF8 | ***HLCS*** | | | | | | | | | | | | | | | | | |
| | SLC12A2 | HLX | | | | | | | | | | | | | | | | | |
| | SLC12A5 | ***HMGCL*** | | | | | | | | | | | | | | | | | |
| | SLC16A1 | HMGN2 | | | | | | | | | | | | | | | | | |
| | SLC17A4 | HMGN5 | | | | | | | | | | | | | | | | | |
| | SLC17A7 | HN1 | | | | | | | | | | | | | | | | | |
| | SLC1A2 | HOGA1 | | | | | | | | | | | | | | | | | |
| | SLC1A3 | HPCAL1 | | | | | | | | | | | | | | | | | |
| | ***SLC1A5*** | HPN | | | | | | | | | | | | | | | | | |
| | ***SLC2A1*** | HR | | | | | | | | | | | | | | | | | |
| | SLC2A10 | HS3ST1 | | | | | | | | | | | | | | | | | |
| | SLC37A2 | HS6ST1 | | | | | | | | | | | | | | | | | |
| | SLC39A10 | ***HSPA12B*** | | | | | | | | | | | | | | | | | |
| | SLC39A11 | HSPB6 | | | | | | | | | | | | | | | | | |
| | ***SLC40A1*** | HSPB8 | | | | | | | | | | | | | | | | | |
| | SLC6A15 | HTR2A | | | | | | | | | | | | | | | | | |
| | SLC6A17 | ***HYAL1*** | | | | | | | | | | | | | | | | | |
| | SLC7A8 | HYAL2 | | | | | | | | | | | | | | | | | |
| | ***SLCO2A1*** | IARS | | | | | | | | | | | | | | | | | |
| | SLITRK3 | ICA1L | | | | | | | | | | | | | | | | | |
| | SLITRK5 | ***ICAM1*** | | | | | | | | | | | | | | | | | |
| | SLITRK6 | ICAM2 | | | | | | | | | | | | | | | | | |
| | SNAI2 | ICK | | | | | | | | | | | | | | | | | |
| | SNAP25 | ICOSL | | | | | | | | | | | | | | | | | |
| | SNCB | ID1 | | | | | | | | | | | | | | | | | |
| | SNPH | ID3 | | | | | | | | | | | | | | | | | |
| | SNX8 | IDI1 | | | | | | | | | | | | | | | | | |
| | SOGA3 | IER5 | | | | | | | | | | | | | | | | | |
| | SORT1 | IFI27 | | | | | | | | | | | | | | | | | |
| | ***SOX4*** | IFI27L2B | | | | | | | | | | | | | | | | | |
| | SOX5 | ***IFITM10*** | | | | | | | | | | | | | | | | | |
| | SPACA6P | IFITM2 | | | | | | | | | | | | | | | | | |
| | SPAG17 | ***IFNAR1*** | | | | | | | | | | | | | | | | | |
| | ***SPARCL1*** | ***IFNGR1*** | | | | | | | | | | | | | | | | | |
| | SPECC1 | IFT122 | | | | | | | | | | | | | | | | | |
| | SPHKAP | IFT140 | | | | | | | | | | | | | | | | | |
| | SPINK5 | IGF1R | | | | | | | | | | | | | | | | | |
| | SPN | IGFBP3 | | | | | | | | | | | | | | | | | |
| | SPON2 | IGSF5 | | | | | | | | | | | | | | | | | |
| | SPTLC3 | IKBKE | | | | | | | | | | | | | | | | | |
| | SRP14 | IKBKG | | | | | | | | | | | | | | | | | |
| | SRRM1 | ***IL13RA1*** | | | | | | | | | | | | | | | | | |
| | SSC5D | IL15 | | | | | | | | | | | | | | | | | |
| | ST8SIA3 | ***IL20RB*** | | | | | | | | | | | | | | | | | |
| | STAB1 | IL27RA | | | | | | | | | | | | | | | | | |
| | STC2 | IL2RG | | | | | | | | | | | | | | | | | |
| | STEAP3 | ***IL3RA*** | | | | | | | | | | | | | | | | | |
| | STEAP4 | IL4RA | | | | | | | | | | | | | | | | | |
| | STK4 | ILK | | | | | | | | | | | | | | | | | |
| | STMN1 | ILVBL | | | | | | | | | | | | | | | | | |
| | STMN2 | IMPDH1 | | | | | | | | | | | | | | | | | |
| | STMN3 | INCENP | | | | | | | | | | | | | | | | | |
| | ***STOX1*** | ***INPP4B*** | | | | | | | | | | | | | | | | | |
| | STRA6 | INPP5K | | | | | | | | | | | | | | | | | |
| | STX1A | INTS9 | | | | | | | | | | | | | | | | | |
| | STX1B | IP6K3 | | | | | | | | | | | | | | | | | |
| | ***STXBP1*** | ***IPMK*** | | | | | | | | | | | | | | | | | |
| | STXBP2 | IPPK | | | | | | | | | | | | | | | | | |
| | SULF1 | ***IQGAP2*** | | | | | | | | | | | | | | | | | |
| | SULT4A1 | ***IQGAP3*** | | | | | | | | | | | | | | | | | |

| | | | | | | | | | | | | | | | | | | | |
|---|---|---|---|---|---|---|---|---|---|---|---|---|---|---|---|---|---|---|---|
| | SV2A | IRAK2 | | | | | | | | | | | | | | | | | |
| | SV2B | IRAK3 | | | | | | | | | | | | | | | | | |
| | SVOP | IRF2BPL | | | | | | | | | | | | | | | | | |
| | ***SYK*** | ***IRF8*** | | | | | | | | | | | | | | | | | |
| | SYN1 | IRS1 | | | | | | | | | | | | | | | | | |
| | ***SYN2*** | IRX3 | | | | | | | | | | | | | | | | | |
| | SYNE1 | ***ITGA8*** | | | | | | | | | | | | | | | | | |
| | SYNGR1 | ***ITGA9*** | | | | | | | | | | | | | | | | | |
| | SYNPR | ITGAV | | | | | | | | | | | | | | | | | |
| | SYP | ITGB3 | | | | | | | | | | | | | | | | | |
| | SYT1 | ITGB3BP | | | | | | | | | | | | | | | | | |
| | SYT11 | ***ITGB4*** | | | | | | | | | | | | | | | | | |
| | SYT16 | ITIH5 | | | | | | | | | | | | | | | | | |
| | SYT4 | ***ITM2C*** | | | | | | | | | | | | | | | | | |
| | ***SYT7*** | ***ITPR1*** | | | | | | | | | | | | | | | | | |
| | SYTL3 | ***ITPR3*** | | | | | | | | | | | | | | | | | |
| | TAGLN3 | ITPRIPL2 | | | | | | | | | | | | | | | | | |
| | ***TARSL2*** | IVNS1ABP | | | | | | | | | | | | | | | | | |
| | ***TBC1D16*** | JAM2 | | | | | | | | | | | | | | | | | |
| | TBR1 | ***JAM3*** | | | | | | | | | | | | | | | | | |
| | TBXAS1 | JKAMP | | | | | | | | | | | | | | | | | |
| | TCF7 | JMJD4 | | | | | | | | | | | | | | | | | |
| | TCHHL1 | ***JUP*** | | | | | | | | | | | | | | | | | |
| | TEP1 | KALRN | | | | | | | | | | | | | | | | | |
| | TEX41 | KANK1 | | | | | | | | | | | | | | | | | |
| | TFAP2A | KANSL1L | | | | | | | | | | | | | | | | | |
| | TFCP2L1 | KAT2A | | | | | | | | | | | | | | | | | |
| | TFEC | ***KAT2B*** | | | | | | | | | | | | | | | | | |
| | ***TGFBI*** | KCNAB2 | | | | | | | | | | | | | | | | | |
| | TGIF1 | KCNB1 | | | | | | | | | | | | | | | | | |
| | THBS2 | ***KCNJ2*** | | | | | | | | | | | | | | | | | |
| | THEMIS2 | ***KCNK6*** | | | | | | | | | | | | | | | | | |
| | ***TIMP1*** | KCNMB1 | | | | | | | | | | | | | | | | | |
| | ***TLL1*** | ***KCNQ4*** | | | | | | | | | | | | | | | | | |
| | TLR6 | KCNT2 | | | | | | | | | | | | | | | | | |
| | ***TMEM106A*** | KCP | | | | | | | | | | | | | | | | | |
| | TMEM130 | ***KCTD12*** | | | | | | | | | | | | | | | | | |
| | ***TMEM176B*** | ***KCTD12B*** | | | | | | | | | | | | | | | | | |
| | TMEM185B | KCTD3 | | | | | | | | | | | | | | | | | |
| | TMEM200A | KDM1A | | | | | | | | | | | | | | | | | |
| | ***TMEM86A*** | KDM6A | | | | | | | | | | | | | | | | | |
| | ***TMOD2*** | ***KDSR*** | | | | | | | | | | | | | | | | | |
| | TMPRSS13 | ***KIF13A*** | | | | | | | | | | | | | | | | | |
| | TNC | ***KIF15*** | | | | | | | | | | | | | | | | | |
| | TNFSF15 | KIF20A | | | | | | | | | | | | | | | | | |
| | TNFSF8 | ***KIF23*** | | | | | | | | | | | | | | | | | |
| | TPO | KIF2C | | | | | | | | | | | | | | | | | |
| | TPPP | ***KIF3A*** | | | | | | | | | | | | | | | | | |
| | TRAF3IP3 | KIF4 | | | | | | | | | | | | | | | | | |
| | TRAM2 | KIF7 | | | | | | | | | | | | | | | | | |
| | TREM1 | KIF9 | | | | | | | | | | | | | | | | | |
| | TREML2 | KIFC3 | | | | | | | | | | | | | | | | | |
| | TREML4 | KIRREL3 | | | | | | | | | | | | | | | | | |
| | TRIM2 | ***KIT*** | | | | | | | | | | | | | | | | | |
| | TRIOBP | ***KLC1*** | | | | | | | | | | | | | | | | | |
| | TSC22D1 | KLC2 | | | | | | | | | | | | | | | | | |
| | TSIX | KLF10 | | | | | | | | | | | | | | | | | |
| | TSPAN18 | KLF11 | | | | | | | | | | | | | | | | | |
| | ***TSPAN7*** | ***KLF2*** | | | | | | | | | | | | | | | | | |
| | ***TSPYL4*** | ***KLF4*** | | | | | | | | | | | | | | | | | |
| | TTN | KLF7 | | | | | | | | | | | | | | | | | |
| | TULP4 | KLHDC2 | | | | | | | | | | | | | | | | | |
| | TYROBP | KLHDC4 | | | | | | | | | | | | | | | | | |
| | UBASH3B | KLHL5 | | | | | | | | | | | | | | | | | |
| | UBR3 | ***KLHL6*** | | | | | | | | | | | | | | | | | |
| | UBXN11 | KLK8 | | | | | | | | | | | | | | | | | |
| | UCHL1 | KLRG2 | | | | | | | | | | | | | | | | | |
| | ***UNC13A*** | KNOP1 | | | | | | | | | | | | | | | | | |
| | UNC13C | KNSTRN | | | | | | | | | | | | | | | | | |
| | UNC80 | KPNA2 | | | | | | | | | | | | | | | | | |
| | URB2 | ***KRAS*** | | | | | | | | | | | | | | | | | |
| | USP11 | ***KRIT1*** | | | | | | | | | | | | | | | | | |
| | USP35 | ***L1CAM*** | | | | | | | | | | | | | | | | | |
| | USP54 | LAMA1 | | | | | | | | | | | | | | | | | |
| | VAMP2 | ***LAMA4*** | | | | | | | | | | | | | | | | | |
| | VAV3 | ***LAMA5*** | | | | | | | | | | | | | | | | | |
| | VCAN | ***LAMB1*** | | | | | | | | | | | | | | | | | |
| | VSNL1 | ***LAMB2*** | | | | | | | | | | | | | | | | | |
| | VSTM2A | ***LAMC3*** | | | | | | | | | | | | | | | | | |
| | WASF1 | LAPTM4B | | | | | | | | | | | | | | | | | |
| | WBSCR17 | LAYN | | | | | | | | | | | | | | | | | |
| | WDFY3 | LBP | | | | | | | | | | | | | | | | | |
| | WDFY4 | ***LCP1*** | | | | | | | | | | | | | | | | | |
| | WDR52 | ***LCP2*** | | | | | | | | | | | | | | | | | |
| | WDR72 | ***LDHB*** | | | | | | | | | | | | | | | | | |
| | WISP1 | LDLRAD4 | | | | | | | | | | | | | | | | | |
| | WNT4 | LEPROTL1 | | | | | | | | | | | | | | | | | |
| | XIRP1 | LFNG | | | | | | | | | | | | | | | | | |
| | YWHAG | LGALS1 | | | | | | | | | | | | | | | | | |
| | YWHAH | LGALS3 | | | | | | | | | | | | | | | | | |
| | ZBTB7C | LGALS3BP | | | | | | | | | | | | | | | | | |
| | ZC3H7B | LIG1 | | | | | | | | | | | | | | | | | |
| | ZDHHC22 | LIMA1 | | | | | | | | | | | | | | | | | |
| | ZER1 | LIMCH1 | | | | | | | | | | | | | | | | | |
| | ZG16 | ***LIMD2*** | | | | | | | | | | | | | | | | | |
| | ZMYND15 | LIMK1 | | | | | | | | | | | | | | | | | |
| | ZNF469 | LIMS1 | | | | | | | | | | | | | | | | | |
| | ZNF483 | LIMS2 | | | | | | | | | | | | | | | | | |
| | ZNF516 | LIN54 | | | | | | | | | | | | | | | | | |
| | ZNF646 | LIPA | | | | | | | | | | | | | | | | | |
| | ZNF710 | LIPE | | | | | | | | | | | | | | | | | |
| | ZNF831 | LIPT2 | | | | | | | | | | | | | | | | | |
| | | LMAN2L | | | | | | | | | | | | | | | | | |
| | | ***LMBR1*** | | | | | | | | | | | | | | | | | |
| | | LMCD1 | | | | | | | | | | | | | | | | | |
| | | LMNB2 | | | | | | | | | | | | | | | | | |
| | | ***LMO4*** | | | | | | | | | | | | | | | | | |
| | | LMOD1 | | | | | | | | | | | | | | | | | |
| | | ***LMTK2*** | | | | | | | | | | | | | | | | | |
| | | ***LONRF2*** | | | | | | | | | | | | | | | | | |
| | | LOXL2 | | | | | | | | | | | | | | | | | |
| | | ***LOXL4*** | | | | | | | | | | | | | | | | | |
| | | LPAR4 | | | | | | | | | | | | | | | | | |
| | | LPAR6 | | | | | | | | | | | | | | | | | |
| | | LPCAT2 | | | | | | | | | | | | | | | | | |
| | | LPCAT4 | | | | | | | | | | | | | | | | | |
| | | LPIN2 | | | | | | | | | | | | | | | | | |
| | | LPIN3 | | | | | | | | | | | | | | | | | |
| | | LRG1 | | | | | | | | | | | | | | | | | |
| | | LRIG1 | | | | | | | | | | | | | | | | | |
| | | LRP5 | | | | | | | | | | | | | | | | | |
| | | LRP8 | | | | | | | | | | | | | | | | | |
| | | ***LRRC57*** | | | | | | | | | | | | | | | | | |

| | | | | | | | | | | | | | | | | | | | |
|---|---|---|---|---|---|---|---|---|---|---|---|---|---|---|---|---|---|---|---|
| | | LRRFIP1 | | | | | | | | | | | | | | | | | |
| | | LSM14B | | | | | | | | | | | | | | | | | |
| | | ***LSR*** | | | | | | | | | | | | | | | | | |
| | | LTB | | | | | | | | | | | | | | | | | |
| | | ***LTBP2*** | | | | | | | | | | | | | | | | | |
| | | ***LTBP4*** | | | | | | | | | | | | | | | | | |
| | | LTC4S | | | | | | | | | | | | | | | | | |
| | | ***LTF*** | | | | | | | | | | | | | | | | | |
| | | ***LUZP1*** | | | | | | | | | | | | | | | | | |
| | | LY6A | | | | | | | | | | | | | | | | | |
| | | LY6C1 | | | | | | | | | | | | | | | | | |
| | | LY75 | | | | | | | | | | | | | | | | | |
| | | M6PR | | | | | | | | | | | | | | | | | |
| | | MAD2L1 | | | | | | | | | | | | | | | | | |
| | | MAF | | | | | | | | | | | | | | | | | |
| | | MAF1 | | | | | | | | | | | | | | | | | |
| | | ***MAFB*** | | | | | | | | | | | | | | | | | |
| | | MAFK | | | | | | | | | | | | | | | | | |
| | | ***MAGED1*** | | | | | | | | | | | | | | | | | |
| | | MAGI3 | | | | | | | | | | | | | | | | | |
| | | MAIP1 | | | | | | | | | | | | | | | | | |
| | | ***MAL*** | | | | | | | | | | | | | | | | | |
| | | MALL | | | | | | | | | | | | | | | | | |
| | | MALRD1 | | | | | | | | | | | | | | | | | |
| | | MAMSTR | | | | | | | | | | | | | | | | | |
| | | ***MAN1A*** | | | | | | | | | | | | | | | | | |
| | | ***MAN2A1*** | | | | | | | | | | | | | | | | | |
| | | MAOA | | | | | | | | | | | | | | | | | |
| | | ***MAP1A*** | | | | | | | | | | | | | | | | | |
| | | ***MAP2*** | | | | | | | | | | | | | | | | | |
| | | MAP3K13 | | | | | | | | | | | | | | | | | |
| | | MAP3K6 | | | | | | | | | | | | | | | | | |
| | | ***MAP3K8*** | | | | | | | | | | | | | | | | | |
| | | MAP4K2 | | | | | | | | | | | | | | | | | |
| | | ***MAP4K4*** | | | | | | | | | | | | | | | | | |
| | | MAP4K5 | | | | | | | | | | | | | | | | | |
| | | MAP6 | | | | | | | | | | | | | | | | | |
| | | ***MAPK11*** | | | | | | | | | | | | | | | | | |
| | | MARCKS | | | | | | | | | | | | | | | | | |
| | | ***MARCKSL1*** | | | | | | | | | | | | | | | | | |
| | | MARS | | | | | | | | | | | | | | | | | |
| | | MARS2 | | | | | | | | | | | | | | | | | |
| | | MARVELD1 | | | | | | | | | | | | | | | | | |
| | | ***MASP1*** | | | | | | | | | | | | | | | | | |
| | | MAST2 | | | | | | | | | | | | | | | | | |
| | | MASTL | | | | | | | | | | | | | | | | | |
| | | MAT2B | | | | | | | | | | | | | | | | | |
| | | ***MATN2*** | | | | | | | | | | | | | | | | | |
| | | ***MB21D2*** | | | | | | | | | | | | | | | | | |
| | | MBD4 | | | | | | | | | | | | | | | | | |
| | | MBD5 | | | | | | | | | | | | | | | | | |
| | | ***MCFD2*** | | | | | | | | | | | | | | | | | |
| | | MCM10 | | | | | | | | | | | | | | | | | |
| | | ***MCM2*** | | | | | | | | | | | | | | | | | |
| | | MCM4 | | | | | | | | | | | | | | | | | |
| | | MCM5 | | | | | | | | | | | | | | | | | |
| | | MCM7 | | | | | | | | | | | | | | | | | |
| | | MCM8 | | | | | | | | | | | | | | | | | |
| | | MCOLN2 | | | | | | | | | | | | | | | | | |
| | | ***MCPH1*** | | | | | | | | | | | | | | | | | |
| | | MCTP2 | | | | | | | | | | | | | | | | | |
| | | MDC1 | | | | | | | | | | | | | | | | | |
| | | MDFI | | | | | | | | | | | | | | | | | |
| | | ***MDGA1*** | | | | | | | | | | | | | | | | | |
| | | MDM1 | | | | | | | | | | | | | | | | | |
| | | MECOM | | | | | | | | | | | | | | | | | |
| | | MEGF9 | | | | | | | | | | | | | | | | | |
| | | ***MELK*** | | | | | | | | | | | | | | | | | |
| | | MESDC1 | | | | | | | | | | | | | | | | | |
| | | MET | | | | | | | | | | | | | | | | | |
| | | ***METRNL*** | | | | | | | | | | | | | | | | | |
| | | METTL26 | | | | | | | | | | | | | | | | | |
| | | METTL7A1 | | | | | | | | | | | | | | | | | |
| | | MEX3A | | | | | | | | | | | | | | | | | |
| | | ***MEX3D*** | | | | | | | | | | | | | | | | | |
| | | MFAP3L | | | | | | | | | | | | | | | | | |
| | | MFNG | | | | | | | | | | | | | | | | | |
| | | MFSD13A | | | | | | | | | | | | | | | | | |
| | | MFSD2A | | | | | | | | | | | | | | | | | |
| | | MFSD7C | | | | | | | | | | | | | | | | | |
| | | ***MGAT5*** | | | | | | | | | | | | | | | | | |
| | | ***MGLL*** | | | | | | | | | | | | | | | | | |
| | | ***MGP*** | | | | | | | | | | | | | | | | | |
| | | MGST3 | | | | | | | | | | | | | | | | | |
| | | MICAL2 | | | | | | | | | | | | | | | | | |
| | | MID1 | | | | | | | | | | | | | | | | | |
| | | MIDN | | | | | | | | | | | | | | | | | |
| | | MIF4GD | | | | | | | | | | | | | | | | | |
| | | MINK1 | | | | | | | | | | | | | | | | | |
| | | ***MKI67*** | | | | | | | | | | | | | | | | | |
| | | MKNK2 | | | | | | | | | | | | | | | | | |
| | | MLXIP | | | | | | | | | | | | | | | | | |
| | | ***MMD*** | | | | | | | | | | | | | | | | | |
| | | MMP11 | | | | | | | | | | | | | | | | | |
| | | ***MMP14*** | | | | | | | | | | | | | | | | | |
| | | MMP15 | | | | | | | | | | | | | | | | | |
| | | ***MMP2*** | | | | | | | | | | | | | | | | | |
| | | MMP24 | | | | | | | | | | | | | | | | | |
| | | ***MMP25*** | | | | | | | | | | | | | | | | | |
| | | MMP28 | | | | | | | | | | | | | | | | | |
| | | MMRN1 | | | | | | | | | | | | | | | | | |
| | | ***MMRN2*** | | | | | | | | | | | | | | | | | |
| | | MN1 | | | | | | | | | | | | | | | | | |
| | | ***MNAT1*** | | | | | | | | | | | | | | | | | |
| | | MNS1 | | | | | | | | | | | | | | | | | |
| | | MOB2 | | | | | | | | | | | | | | | | | |
| | | MOCS1 | | | | | | | | | | | | | | | | | |
| | | MORC4 | | | | | | | | | | | | | | | | | |
| | | ***MOSPD2*** | | | | | | | | | | | | | | | | | |
| | | MPC1 | | | | | | | | | | | | | | | | | |
| | | MPND | | | | | | | | | | | | | | | | | |
| | | MPP5 | | | | | | | | | | | | | | | | | |
| | | MPP6 | | | | | | | | | | | | | | | | | |
| | | MPZL3 | | | | | | | | | | | | | | | | | |
| | | ***MRAS*** | | | | | | | | | | | | | | | | | |
| | | MROH1 | | | | | | | | | | | | | | | | | |
| | | MRPL41 | | | | | | | | | | | | | | | | | |
| | | MRPS28 | | | | | | | | | | | | | | | | | |
| | | MRPS5 | | | | | | | | | | | | | | | | | |
| | | MRVI1 | | | | | | | | | | | | | | | | | |
| | | MS4A6D | | | | | | | | | | | | | | | | | |
| | | MSH3 | | | | | | | | | | | | | | | | | |
| | | MSMP | | | | | | | | | | | | | | | | | |

| | | | | | | | | | | | | | | | | | | | |
|---|---|---|---|---|---|---|---|---|---|---|---|---|---|---|---|---|---|---|---|
| | | MT1 | | | | | | | | | | | | | | | | | |
| | | ***MT2*** | | | | | | | | | | | | | | | | | |
| | | ***MTCH1*** | | | | | | | | | | | | | | | | | |
| | | MTFR1 | | | | | | | | | | | | | | | | | |
| | | MTHFD2 | | | | | | | | | | | | | | | | | |
| | | MTMR10 | | | | | | | | | | | | | | | | | |
| | | MTMR2 | | | | | | | | | | | | | | | | | |
| | | MTMR4 | | | | | | | | | | | | | | | | | |
| | | MTSS1 | | | | | | | | | | | | | | | | | |
| | | MTTP | | | | | | | | | | | | | | | | | |
| | | MUSTN1 | | | | | | | | | | | | | | | | | |
| | | MVB12A | | | | | | | | | | | | | | | | | |
| | | MXD3 | | | | | | | | | | | | | | | | | |
| | | MYC | | | | | | | | | | | | | | | | | |
| | | MYCL | | | | | | | | | | | | | | | | | |
| | | ***MYH10*** | | | | | | | | | | | | | | | | | |
| | | MYH11 | | | | | | | | | | | | | | | | | |
| | | ***MYH9*** | | | | | | | | | | | | | | | | | |
| | | MYL12A | | | | | | | | | | | | | | | | | |
| | | MYL9 | | | | | | | | | | | | | | | | | |
| | | MYO10 | | | | | | | | | | | | | | | | | |
| | | ***MYO1B*** | | | | | | | | | | | | | | | | | |
| | | ***MYO1C*** | | | | | | | | | | | | | | | | | |
| | | ***MYO7A*** | | | | | | | | | | | | | | | | | |
| | | MYRIP | | | | | | | | | | | | | | | | | |
| | | NAAA | | | | | | | | | | | | | | | | | |
| | | NAP1L1 | | | | | | | | | | | | | | | | | |
| | | NAPRT | | | | | | | | | | | | | | | | | |
| | | NAPSA | | | | | | | | | | | | | | | | | |
| | | NARS | | | | | | | | | | | | | | | | | |
| | | NAT6 | | | | | | | | | | | | | | | | | |
| | | NAV2 | | | | | | | | | | | | | | | | | |
| | | NAXD | | | | | | | | | | | | | | | | | |
| | | NBEAL2 | | | | | | | | | | | | | | | | | |
| | | NCALD | | | | | | | | | | | | | | | | | |
| | | ***NCAM1*** | | | | | | | | | | | | | | | | | |
| | | NCAPD2 | | | | | | | | | | | | | | | | | |
| | | NCAPD3 | | | | | | | | | | | | | | | | | |
| | | ***NCAPG*** | | | | | | | | | | | | | | | | | |
| | | NCAPG2 | | | | | | | | | | | | | | | | | |
| | | NCEH1 | | | | | | | | | | | | | | | | | |
| | | NCK1 | | | | | | | | | | | | | | | | | |
| | | NCK2 | | | | | | | | | | | | | | | | | |
| | | ***NCKAP5L*** | | | | | | | | | | | | | | | | | |
| | | NCKIPSD | | | | | | | | | | | | | | | | | |
| | | ***NCOA3*** | | | | | | | | | | | | | | | | | |
| | | NDC80 | | | | | | | | | | | | | | | | | |
| | | NDE1 | | | | | | | | | | | | | | | | | |
| | | ***NDNF*** | | | | | | | | | | | | | | | | | |
| | | ***NDRG4*** | | | | | | | | | | | | | | | | | |
| | | NDUFA8 | | | | | | | | | | | | | | | | | |
| | | ***NDUFC2*** | | | | | | | | | | | | | | | | | |
| | | NDUFS8 | | | | | | | | | | | | | | | | | |
| | | NECTIN2 | | | | | | | | | | | | | | | | | |
| | | ***NECTIN3*** | | | | | | | | | | | | | | | | | |
| | | ***NEDD4*** | | | | | | | | | | | | | | | | | |
| | | NEIL1 | | | | | | | | | | | | | | | | | |
| | | NEIL3 | | | | | | | | | | | | | | | | | |
| | | ***NEK7*** | | | | | | | | | | | | | | | | | |
| | | NET1 | | | | | | | | | | | | | | | | | |
| | | ***NEURL2*** | | | | | | | | | | | | | | | | | |
| | | NEXN | | | | | | | | | | | | | | | | | |
| | | NFATC1 | | | | | | | | | | | | | | | | | |
| | | NFIC | | | | | | | | | | | | | | | | | |
| | | NFXL1 | | | | | | | | | | | | | | | | | |
| | | NGF | | | | | | | | | | | | | | | | | |
| | | ***NID2*** | | | | | | | | | | | | | | | | | |
| | | NIPAL1 | | | | | | | | | | | | | | | | | |
| | | NIPAL3 | | | | | | | | | | | | | | | | | |
| | | NKD1 | | | | | | | | | | | | | | | | | |
| | | ***NKD2*** | | | | | | | | | | | | | | | | | |
| | | ***NLRC3*** | | | | | | | | | | | | | | | | | |
| | | ***NMNAT2*** | | | | | | | | | | | | | | | | | |
| | | ***NMT2*** | | | | | | | | | | | | | | | | | |
| | | NOCT | | | | | | | | | | | | | | | | | |
| | | NOD2 | | | | | | | | | | | | | | | | | |
| | | NOS1 | | | | | | | | | | | | | | | | | |
| | | ***NOS2*** | | | | | | | | | | | | | | | | | |
| | | ***NOS3*** | | | | | | | | | | | | | | | | | |
| | | NOSTRIN | | | | | | | | | | | | | | | | | |
| | | NOVA2 | | | | | | | | | | | | | | | | | |
| | | NOX4 | | | | | | | | | | | | | | | | | |
| | | NPAS2 | | | | | | | | | | | | | | | | | |
| | | ***NQO1*** | | | | | | | | | | | | | | | | | |
| | | NR1D1 | | | | | | | | | | | | | | | | | |
| | | NR2F2 | | | | | | | | | | | | | | | | | |
| | | NR3C1 | | | | | | | | | | | | | | | | | |
| | | NR4A1 | | | | | | | | | | | | | | | | | |
| | | NR5A2 | | | | | | | | | | | | | | | | | |
| | | NRARP | | | | | | | | | | | | | | | | | |
| | | NRBP2 | | | | | | | | | | | | | | | | | |
| | | NREP | | | | | | | | | | | | | | | | | |
| | | ***NRGN*** | | | | | | | | | | | | | | | | | |
| | | NRM | | | | | | | | | | | | | | | | | |
| | | ***NRP1*** | | | | | | | | | | | | | | | | | |
| | | ***NRP2*** | | | | | | | | | | | | | | | | | |
| | | ***NRROS*** | | | | | | | | | | | | | | | | | |
| | | NSF | | | | | | | | | | | | | | | | | |
| | | NSG1 | | | | | | | | | | | | | | | | | |
| | | NSMCE1 | | | | | | | | | | | | | | | | | |
| | | NSUN6 | | | | | | | | | | | | | | | | | |
| | | NT5C | | | | | | | | | | | | | | | | | |
| | | ***NT5C2*** | | | | | | | | | | | | | | | | | |
| | | ***NTN1*** | | | | | | | | | | | | | | | | | |
| | | NTNG2 | | | | | | | | | | | | | | | | | |
| | | NUCB2 | | | | | | | | | | | | | | | | | |
| | | NUDT4 | | | | | | | | | | | | | | | | | |
| | | NUF2 | | | | | | | | | | | | | | | | | |
| | | ***NUMB*** | | | | | | | | | | | | | | | | | |
| | | NUP160 | | | | | | | | | | | | | | | | | |
| | | ***NUP85*** | | | | | | | | | | | | | | | | | |
| | | NUS1 | | | | | | | | | | | | | | | | | |
| | | NUSAP1 | | | | | | | | | | | | | | | | | |
| | | NXN | | | | | | | | | | | | | | | | | |
| | | NXPE2 | | | | | | | | | | | | | | | | | |
| | | NXPH3 | | | | | | | | | | | | | | | | | |
| | | NYNRIN | | | | | | | | | | | | | | | | | |
| | | ***OAF*** | | | | | | | | | | | | | | | | | |
| | | OAS2 | | | | | | | | | | | | | | | | | |
| | | OAZ2 | | | | | | | | | | | | | | | | | |
| | | ORAI3 | | | | | | | | | | | | | | | | | |
| | | ORC1 | | | | | | | | | | | | | | | | | |
| | | OSBP2 | | | | | | | | | | | | | | | | | |

| | | | | | | | | | | | | | | | | | | | |
|---|---|---|---|---|---|---|---|---|---|---|---|---|---|---|---|---|---|---|---|
| | | OSCP1 | | | | | | | | | | | | | | | | | |
| | | OSMR | | | | | | | | | | | | | | | | | |
| | | OSTM1 | | | | | | | | | | | | | | | | | |
| | | OTUD1 | | | | | | | | | | | | | | | | | |
| | | ***OXCT1*** | | | | | | | | | | | | | | | | | |
| | | P2RY1 | | | | | | | | | | | | | | | | | |
| | | P2RY2 | | | | | | | | | | | | | | | | | |
| | | P2RY6 | | | | | | | | | | | | | | | | | |
| | | P3H2 | | | | | | | | | | | | | | | | | |
| | | ***P3H3*** | | | | | | | | | | | | | | | | | |
| | | PACS1 | | | | | | | | | | | | | | | | | |
| | | ***PAICS*** | | | | | | | | | | | | | | | | | |
| | | ***PAK4*** | | | | | | | | | | | | | | | | | |
| | | ***PALM*** | | | | | | | | | | | | | | | | | |
| | | PANX1 | | | | | | | | | | | | | | | | | |
| | | PAPD5 | | | | | | | | | | | | | | | | | |
| | | PAQR4 | | | | | | | | | | | | | | | | | |
| | | ***PAQR7*** | | | | | | | | | | | | | | | | | |
| | | ***PAQR8*** | | | | | | | | | | | | | | | | | |
| | | PARD3B | | | | | | | | | | | | | | | | | |
| | | PARP16 | | | | | | | | | | | | | | | | | |
| | | PARP8 | | | | | | | | | | | | | | | | | |
| | | PARPBP | | | | | | | | | | | | | | | | | |
| | | PASK | | | | | | | | | | | | | | | | | |
| | | PAWR | | | | | | | | | | | | | | | | | |
| | | ***PBK*** | | | | | | | | | | | | | | | | | |
| | | PBP2 | | | | | | | | | | | | | | | | | |
| | | PBXIP1 | | | | | | | | | | | | | | | | | |
| | | PCDH19 | | | | | | | | | | | | | | | | | |
| | | PCDH7 | | | | | | | | | | | | | | | | | |
| | | ***PCDHGA5*** | | | | | | | | | | | | | | | | | |
| | | PCED1B | | | | | | | | | | | | | | | | | |
| | | PCGF2 | | | | | | | | | | | | | | | | | |
| | | PCMTD1 | | | | | | | | | | | | | | | | | |
| | | PCYOX1 | | | | | | | | | | | | | | | | | |
| | | PDCL3 | | | | | | | | | | | | | | | | | |
| | | PDE4A | | | | | | | | | | | | | | | | | |
| | | PDE4B | | | | | | | | | | | | | | | | | |
| | | PDE4C | | | | | | | | | | | | | | | | | |
| | | PDE7A | | | | | | | | | | | | | | | | | |
| | | PDGFA | | | | | | | | | | | | | | | | | |
| | | ***PDGFRA*** | | | | | | | | | | | | | | | | | |
| | | ***PDIA6*** | | | | | | | | | | | | | | | | | |
| | | ***PDLIM2*** | | | | | | | | | | | | | | | | | |
| | | ***PDZD2*** | | | | | | | | | | | | | | | | | |
| | | ***PECAM1*** | | | | | | | | | | | | | | | | | |
| | | PER3 | | | | | | | | | | | | | | | | | |
| | | PEX11A | | | | | | | | | | | | | | | | | |
| | | PFAS | | | | | | | | | | | | | | | | | |
| | | PFKFB3 | | | | | | | | | | | | | | | | | |
| | | PFKP | | | | | | | | | | | | | | | | | |
| | | PGAP1 | | | | | | | | | | | | | | | | | |
| | | PGF | | | | | | | | | | | | | | | | | |
| | | ***PGGHG*** | | | | | | | | | | | | | | | | | |
| | | PGLYRP1 | | | | | | | | | | | | | | | | | |
| | | PGM2L1 | | | | | | | | | | | | | | | | | |
| | | ***PGM5*** | | | | | | | | | | | | | | | | | |
| | | ***PHACTR1*** | | | | | | | | | | | | | | | | | |
| | | PHF19 | | | | | | | | | | | | | | | | | |
| | | PHF23 | | | | | | | | | | | | | | | | | |
| | | ***PHLDB1*** | | | | | | | | | | | | | | | | | |
| | | PICALM | | | | | | | | | | | | | | | | | |
| | | PIEZO1 | | | | | | | | | | | | | | | | | |
| | | PIGG | | | | | | | | | | | | | | | | | |
| | | PIGT | | | | | | | | | | | | | | | | | |
| | | ***PIH1D1*** | | | | | | | | | | | | | | | | | |
| | | PIK3C2A | | | | | | | | | | | | | | | | | |
| | | PIK3CB | | | | | | | | | | | | | | | | | |
| | | ***PIK3CG*** | | | | | | | | | | | | | | | | | |
| | | PIK3R3 | | | | | | | | | | | | | | | | | |
| | | ***PIK3R5*** | | | | | | | | | | | | | | | | | |
| | | ***PIK3R6*** | | | | | | | | | | | | | | | | | |
| | | ***PINK1*** | | | | | | | | | | | | | | | | | |
| | | PIP5K1C | | | | | | | | | | | | | | | | | |
| | | ***PIR*** | | | | | | | | | | | | | | | | | |
| | | PITPNC1 | | | | | | | | | | | | | | | | | |
| | | PITPNM2 | | | | | | | | | | | | | | | | | |
| | | ***PITPNM3*** | | | | | | | | | | | | | | | | | |
| | | PITRM1 | | | | | | | | | | | | | | | | | |
| | | PKD1 | | | | | | | | | | | | | | | | | |
| | | PKN3 | | | | | | | | | | | | | | | | | |
| | | PKNOX2 | | | | | | | | | | | | | | | | | |
| | | PKP4 | | | | | | | | | | | | | | | | | |
| | | PLA1A | | | | | | | | | | | | | | | | | |
| | | PLA2R1 | | | | | | | | | | | | | | | | | |
| | | PLAT | | | | | | | | | | | | | | | | | |
| | | ***PLAUR*** | | | | | | | | | | | | | | | | | |
| | | PLCB4 | | | | | | | | | | | | | | | | | |
| | | ***PLCD3*** | | | | | | | | | | | | | | | | | |
| | | PLD2 | | | | | | | | | | | | | | | | | |
| | | ***PLEC*** | | | | | | | | | | | | | | | | | |
| | | PLEKHA1 | | | | | | | | | | | | | | | | | |
| | | PLEKHA7 | | | | | | | | | | | | | | | | | |
| | | PLEKHG5 | | | | | | | | | | | | | | | | | |
| | | ***PLEKHM1*** | | | | | | | | | | | | | | | | | |
| | | PLEKHO1 | | | | | | | | | | | | | | | | | |
| | | ***PLK1*** | | | | | | | | | | | | | | | | | |
| | | ***PLOD1*** | | | | | | | | | | | | | | | | | |
| | | ***PLPP3*** | | | | | | | | | | | | | | | | | |
| | | PLPPR2 | | | | | | | | | | | | | | | | | |
| | | PLS3 | | | | | | | | | | | | | | | | | |
| | | PLSCR3 | | | | | | | | | | | | | | | | | |
| | | ***PLTP*** | | | | | | | | | | | | | | | | | |
| | | PLVAP | | | | | | | | | | | | | | | | | |
| | | ***PLXDC2*** | | | | | | | | | | | | | | | | | |
| | | PLXNA2 | | | | | | | | | | | | | | | | | |
| | | PLXNB1 | | | | | | | | | | | | | | | | | |
| | | ***PMP22*** | | | | | | | | | | | | | | | | | |
| | | PNKD | | | | | | | | | | | | | | | | | |
| | | PNPLA2 | | | | | | | | | | | | | | | | | |
| | | PNPLA7 | | | | | | | | | | | | | | | | | |
| | | ***PNRC1*** | | | | | | | | | | | | | | | | | |
| | | ***PODXL*** | | | | | | | | | | | | | | | | | |
| | | POFUT2 | | | | | | | | | | | | | | | | | |
| | | POGK | | | | | | | | | | | | | | | | | |
| | | POLA2 | | | | | | | | | | | | | | | | | |
| | | ***POLD1*** | | | | | | | | | | | | | | | | | |
| | | POLE | | | | | | | | | | | | | | | | | |
| | | ***POLH*** | | | | | | | | | | | | | | | | | |
| | | PON2 | | | | | | | | | | | | | | | | | |
| | | POU2F3 | | | | | | | | | | | | | | | | | |
| | | PPL | | | | | | | | | | | | | | | | | |
| | | PPM1E | | | | | | | | | | | | | | | | | |

| | | | | | | | | | | | | | | | | | | | |
|---|---|---|---|---|---|---|---|---|---|---|---|---|---|---|---|---|---|---|---|
| | | PPM1F | | | | | | | | | | | | | | | | | |
| | | PPP1R11 | | | | | | | | | | | | | | | | | |
| | | PPP1R2 | | | | | | | | | | | | | | | | | |
| | | PPP1R37 | | | | | | | | | | | | | | | | | |
| | | ***PPP2R1B*** | | | | | | | | | | | | | | | | | |
| | | ***PPP2R3A*** | | | | | | | | | | | | | | | | | |
| | | PPP3CB | | | | | | | | | | | | | | | | | |
| | | PQLC3 | | | | | | | | | | | | | | | | | |
| | | PRADC1 | | | | | | | | | | | | | | | | | |
| | | PRC1 | | | | | | | | | | | | | | | | | |
| | | PRDM11 | | | | | | | | | | | | | | | | | |
| | | PRELP | | | | | | | | | | | | | | | | | |
| | | PRICKLE1 | | | | | | | | | | | | | | | | | |
| | | PRIM2 | | | | | | | | | | | | | | | | | |
| | | PRKAB1 | | | | | | | | | | | | | | | | | |
| | | PRKAR2A | | | | | | | | | | | | | | | | | |
| | | ***PRKCG*** | | | | | | | | | | | | | | | | | |
| | | ***PRKCH*** | | | | | | | | | | | | | | | | | |
| | | PRKG1 | | | | | | | | | | | | | | | | | |
| | | PROCR | | | | | | | | | | | | | | | | | |
| | | PROX2 | | | | | | | | | | | | | | | | | |
| | | ***PRPS2*** | | | | | | | | | | | | | | | | | |
| | | PRR5L | | | | | | | | | | | | | | | | | |
| | | PRRC1 | | | | | | | | | | | | | | | | | |
| | | PRRT4 | | | | | | | | | | | | | | | | | |
| | | PRUNE2 | | | | | | | | | | | | | | | | | |
| | | PRX | | | | | | | | | | | | | | | | | |
| | | PSMA7 | | | | | | | | | | | | | | | | | |
| | | PSMC3IP | | | | | | | | | | | | | | | | | |
| | | PSME2B | | | | | | | | | | | | | | | | | |
| | | ***PTCH1*** | | | | | | | | | | | | | | | | | |
| | | PTER | | | | | | | | | | | | | | | | | |
| | | ***PTGES*** | | | | | | | | | | | | | | | | | |
| | | ***PTGFRN*** | | | | | | | | | | | | | | | | | |
| | | ***PTGS1*** | | | | | | | | | | | | | | | | | |
| | | PTOV1 | | | | | | | | | | | | | | | | | |
| | | ***PTPN1*** | | | | | | | | | | | | | | | | | |
| | | PTPN14 | | | | | | | | | | | | | | | | | |
| | | ***PTPN7*** | | | | | | | | | | | | | | | | | |
| | | PTPRG | | | | | | | | | | | | | | | | | |
| | | ***PTPRJ*** | | | | | | | | | | | | | | | | | |
| | | ***PTPRK*** | | | | | | | | | | | | | | | | | |
| | | PTPRN | | | | | | | | | | | | | | | | | |
| | | ***PTPRR*** | | | | | | | | | | | | | | | | | |
| | | PTPRS | | | | | | | | | | | | | | | | | |
| | | ***PTPRU*** | | | | | | | | | | | | | | | | | |
| | | PTPRZ1 | | | | | | | | | | | | | | | | | |
| | | ***PTRF*** | | | | | | | | | | | | | | | | | |
| | | PTTG1IP | | | | | | | | | | | | | | | | | |
| | | PTX3 | | | | | | | | | | | | | | | | | |
| | | ***PWWP2B*** | | | | | | | | | | | | | | | | | |
| | | ***PXDN*** | | | | | | | | | | | | | | | | | |
| | | ***RAB11A*** | | | | | | | | | | | | | | | | | |
| | | ***RAB3A*** | | | | | | | | | | | | | | | | | |
| | | RAB3D | | | | | | | | | | | | | | | | | |
| | | RABAC1 | | | | | | | | | | | | | | | | | |
| | | RABEP2 | | | | | | | | | | | | | | | | | |
| | | RABGGTA | | | | | | | | | | | | | | | | | |
| | | RACGAP1 | | | | | | | | | | | | | | | | | |
| | | ***RAD51*** | | | | | | | | | | | | | | | | | |
| | | RAD51AP1 | | | | | | | | | | | | | | | | | |
| | | RADIL | | | | | | | | | | | | | | | | | |
| | | RAET1D | | | | | | | | | | | | | | | | | |
| | | RALB | | | | | | | | | | | | | | | | | |
| | | RALGDS | | | | | | | | | | | | | | | | | |
| | | RALGPS1 | | | | | | | | | | | | | | | | | |
| | | ***RALGPS2*** | | | | | | | | | | | | | | | | | |
| | | RAMP2 | | | | | | | | | | | | | | | | | |
| | | RAMP3 | | | | | | | | | | | | | | | | | |
| | | ***RAP1A*** | | | | | | | | | | | | | | | | | |
| | | RAP1B | | | | | | | | | | | | | | | | | |
| | | ***RAPGEF1*** | | | | | | | | | | | | | | | | | |
| | | ***RAPGEF3*** | | | | | | | | | | | | | | | | | |
| | | ***RAPGEF4*** | | | | | | | | | | | | | | | | | |
| | | RAPH1 | | | | | | | | | | | | | | | | | |
| | | ***RARA*** | | | | | | | | | | | | | | | | | |
| | | RARRES1 | | | | | | | | | | | | | | | | | |
| | | RARS | | | | | | | | | | | | | | | | | |
| | | ***RASGRP2*** | | | | | | | | | | | | | | | | | |
| | | RASL10A | | | | | | | | | | | | | | | | | |
| | | ***RASSF2*** | | | | | | | | | | | | | | | | | |
| | | ***RASSF4*** | | | | | | | | | | | | | | | | | |
| | | RBCK1 | | | | | | | | | | | | | | | | | |
| | | RBL1 | | | | | | | | | | | | | | | | | |
| | | RBM18 | | | | | | | | | | | | | | | | | |
| | | RBM20 | | | | | | | | | | | | | | | | | |
| | | RBPMS2 | | | | | | | | | | | | | | | | | |
| | | ***RCBTB2*** | | | | | | | | | | | | | | | | | |
| | | ***RCC2*** | | | | | | | | | | | | | | | | | |
| | | RD3 | | | | | | | | | | | | | | | | | |
| | | ***RDH12*** | | | | | | | | | | | | | | | | | |
| | | RECQL | | | | | | | | | | | | | | | | | |
| | | REEP1 | | | | | | | | | | | | | | | | | |
| | | ***REEP2*** | | | | | | | | | | | | | | | | | |
| | | RET | | | | | | | | | | | | | | | | | |
| | | ***RFC2*** | | | | | | | | | | | | | | | | | |
| | | RFC4 | | | | | | | | | | | | | | | | | |
| | | RFC5 | | | | | | | | | | | | | | | | | |
| | | RFFL | | | | | | | | | | | | | | | | | |
| | | RFK | | | | | | | | | | | | | | | | | |
| | | RFTN2 | | | | | | | | | | | | | | | | | |
| | | RGP1 | | | | | | | | | | | | | | | | | |
| | | ***RGS12*** | | | | | | | | | | | | | | | | | |
| | | RGS16 | | | | | | | | | | | | | | | | | |
| | | RHBDF2 | | | | | | | | | | | | | | | | | |
| | | RHOA | | | | | | | | | | | | | | | | | |
| | | RHOBTB1 | | | | | | | | | | | | | | | | | |
| | | RHOBTB3 | | | | | | | | | | | | | | | | | |
| | | ***RHPN2*** | | | | | | | | | | | | | | | | | |
| | | RICTOR | | | | | | | | | | | | | | | | | |
| | | RILPL1 | | | | | | | | | | | | | | | | | |
| | | RIN1 | | | | | | | | | | | | | | | | | |
| | | ***RIN3*** | | | | | | | | | | | | | | | | | |
| | | RIPK2 | | | | | | | | | | | | | | | | | |
| | | RIPPLY3 | | | | | | | | | | | | | | | | | |
| | | RND2 | | | | | | | | | | | | | | | | | |
| | | RND3 | | | | | | | | | | | | | | | | | |
| | | RNF11 | | | | | | | | | | | | | | | | | |
| | | RNF122 | | | | | | | | | | | | | | | | | |
| | | ***RNF144A*** | | | | | | | | | | | | | | | | | |
| | | RNF168 | | | | | | | | | | | | | | | | | |
| | | RNF169 | | | | | | | | | | | | | | | | | |
| | | RNF19A | | | | | | | | | | | | | | | | | |

| | | | | | | | | | | | | | | | | | | | |
|---|---|---|---|---|---|---|---|---|---|---|---|---|---|---|---|---|---|---|---|
| | | RNF2 | | | | | | | | | | | | | | | | | |
| | | RNPC3 | | | | | | | | | | | | | | | | | |
| | | ROGDI | | | | | | | | | | | | | | | | | |
| | | RPA1 | | | | | | | | | | | | | | | | | |
| | | RPA2 | | | | | | | | | | | | | | | | | |
| | | RPH3AL | | | | | | | | | | | | | | | | | |
| | | RPS26 | | | | | | | | | | | | | | | | | |
| | | ***RPS6KA2*** | | | | | | | | | | | | | | | | | |
| | | ***RPS6KA5*** | | | | | | | | | | | | | | | | | |
| | | ***RRAS2*** | | | | | | | | | | | | | | | | | |
| | | RRM1 | | | | | | | | | | | | | | | | | |
| | | RRM2 | | | | | | | | | | | | | | | | | |
| | | ***RTP3*** | | | | | | | | | | | | | | | | | |
| | | ***RXRA*** | | | | | | | | | | | | | | | | | |
| | | RYR3 | | | | | | | | | | | | | | | | | |
| | | S100A1 | | | | | | | | | | | | | | | | | |
| | | ***S100A10*** | | | | | | | | | | | | | | | | | |
| | | S100A6 | | | | | | | | | | | | | | | | | |
| | | ***S100A7A*** | | | | | | | | | | | | | | | | | |
| | | ***S1PR1*** | | | | | | | | | | | | | | | | | |
| | | S1PR4 | | | | | | | | | | | | | | | | | |
| | | SACS | | | | | | | | | | | | | | | | | |
| | | ***SAMD4B*** | | | | | | | | | | | | | | | | | |
| | | ***SAMD5*** | | | | | | | | | | | | | | | | | |
| | | SARAF | | | | | | | | | | | | | | | | | |
| | | SARS | | | | | | | | | | | | | | | | | |
| | | SAT1 | | | | | | | | | | | | | | | | | |
| | | SCAI | | | | | | | | | | | | | | | | | |
| | | SCAMP1 | | | | | | | | | | | | | | | | | |
| | | SCAPER | | | | | | | | | | | | | | | | | |
| | | SCARB1 | | | | | | | | | | | | | | | | | |
| | | SCD1 | | | | | | | | | | | | | | | | | |
| | | SCD2 | | | | | | | | | | | | | | | | | |
| | | ***SCMH1*** | | | | | | | | | | | | | | | | | |
| | | SCRN1 | | | | | | | | | | | | | | | | | |
| | | SCRN3 | | | | | | | | | | | | | | | | | |
| | | ***SCUBE3*** | | | | | | | | | | | | | | | | | |
| | | ***SDC4*** | | | | | | | | | | | | | | | | | |
| | | SDCBP2 | | | | | | | | | | | | | | | | | |
| | | ***SDPR*** | | | | | | | | | | | | | | | | | |
| | | SDR42E1 | | | | | | | | | | | | | | | | | |
| | | ***SEC14L1*** | | | | | | | | | | | | | | | | | |
| | | SEC23A | | | | | | | | | | | | | | | | | |
| | | SEC61B | | | | | | | | | | | | | | | | | |
| | | SELE | | | | | | | | | | | | | | | | | |
| | | ***SELENBP1*** | | | | | | | | | | | | | | | | | |
| | | SELENON | | | | | | | | | | | | | | | | | |
| | | SELENOO | | | | | | | | | | | | | | | | | |
| | | ***SELP*** | | | | | | | | | | | | | | | | | |
| | | SEMA3A | | | | | | | | | | | | | | | | | |
| | | ***SEMA3C*** | | | | | | | | | | | | | | | | | |
| | | SEMA3E | | | | | | | | | | | | | | | | | |
| | | SEMA3F | | | | | | | | | | | | | | | | | |
| | | SEMA4B | | | | | | | | | | | | | | | | | |
| | | SEMA4C | | | | | | | | | | | | | | | | | |
| | | SEMA7A | | | | | | | | | | | | | | | | | |
| | | SENP2 | | | | | | | | | | | | | | | | | |
| | | ***SEPT1*** | | | | | | | | | | | | | | | | | |
| | | ***SEPT4*** | | | | | | | | | | | | | | | | | |
| | | ***SEPT7*** | | | | | | | | | | | | | | | | | |
| | | SEPT8 | | | | | | | | | | | | | | | | | |
| | | SEPT9 | | | | | | | | | | | | | | | | | |
| | | SERINC5 | | | | | | | | | | | | | | | | | |
| | | SERPINA3F | | | | | | | | | | | | | | | | | |
| | | SERPINA3G | | | | | | | | | | | | | | | | | |
| | | SERPINB1C | | | | | | | | | | | | | | | | | |
| | | SERPINB6B | | | | | | | | | | | | | | | | | |
| | | SERPINB9B | | | | | | | | | | | | | | | | | |
| | | SERTAD1 | | | | | | | | | | | | | | | | | |
| | | SERTAD2 | | | | | | | | | | | | | | | | | |
| | | ***SESN3*** | | | | | | | | | | | | | | | | | |
| | | ***SETX*** | | | | | | | | | | | | | | | | | |
| | | SFN | | | | | | | | | | | | | | | | | |
| | | ***SFT2D2*** | | | | | | | | | | | | | | | | | |
| | | SGK1 | | | | | | | | | | | | | | | | | |
| | | SGPL1 | | | | | | | | | | | | | | | | | |
| | | SH2B1 | | | | | | | | | | | | | | | | | |
| | | ***SH2B3*** | | | | | | | | | | | | | | | | | |
| | | SH2D3C | | | | | | | | | | | | | | | | | |
| | | SH3BGRL | | | | | | | | | | | | | | | | | |
| | | SH3BGRL2 | | | | | | | | | | | | | | | | | |
| | | SH3D21 | | | | | | | | | | | | | | | | | |
| | | SH3PXD2A | | | | | | | | | | | | | | | | | |
| | | ***SH3PXD2B*** | | | | | | | | | | | | | | | | | |
| | | SHANK3 | | | | | | | | | | | | | | | | | |
| | | SHMT2 | | | | | | | | | | | | | | | | | |
| | | SHROOM1 | | | | | | | | | | | | | | | | | |
| | | SHROOM2 | | | | | | | | | | | | | | | | | |
| | | SIAE | | | | | | | | | | | | | | | | | |
| | | SIGMAR1 | | | | | | | | | | | | | | | | | |
| | | SIK1 | | | | | | | | | | | | | | | | | |
| | | SIKE1 | | | | | | | | | | | | | | | | | |
| | | ***SIPA1*** | | | | | | | | | | | | | | | | | |
| | | SIPA1L1 | | | | | | | | | | | | | | | | | |
| | | SIPA1L2 | | | | | | | | | | | | | | | | | |
| | | SIRPA | | | | | | | | | | | | | | | | | |
| | | ***SIX1*** | | | | | | | | | | | | | | | | | |
| | | ***SIX4*** | | | | | | | | | | | | | | | | | |
| | | SKIL | | | | | | | | | | | | | | | | | |
| | | SLBP | | | | | | | | | | | | | | | | | |
| | | SLC12A7 | | | | | | | | | | | | | | | | | |
| | | ***SLC12A8*** | | | | | | | | | | | | | | | | | |
| | | SLC16A3 | | | | | | | | | | | | | | | | | |
| | | SLC1A4 | | | | | | | | | | | | | | | | | |
| | | ***SLC1A5*** | | | | | | | | | | | | | | | | | |
| | | SLC22A14 | | | | | | | | | | | | | | | | | |
| | | SLC22A18 | | | | | | | | | | | | | | | | | |
| | | ***SLC23A2*** | | | | | | | | | | | | | | | | | |
| | | SLC25A12 | | | | | | | | | | | | | | | | | |
| | | SLC25A24 | | | | | | | | | | | | | | | | | |
| | | SLC29A1 | | | | | | | | | | | | | | | | | |
| | | SLC29A4 | | | | | | | | | | | | | | | | | |
| | | ***SLC2A1*** | | | | | | | | | | | | | | | | | |
| | | SLC2A6 | | | | | | | | | | | | | | | | | |
| | | SLC2A9 | | | | | | | | | | | | | | | | | |
| | | SLC30A9 | | | | | | | | | | | | | | | | | |
| | | SLC31A1 | | | | | | | | | | | | | | | | | |
| | | SLC35F1 | | | | | | | | | | | | | | | | | |
| | | SLC38A1 | | | | | | | | | | | | | | | | | |
| | | SLC38A2 | | | | | | | | | | | | | | | | | |
| | | ***SLC38A5*** | | | | | | | | | | | | | | | | | |
| | | SLC38A6 | | | | | | | | | | | | | | | | | |
| | | SLC38A7 | | | | | | | | | | | | | | | | | |

| | | | | | | | | | | | | | | | | | | | |
|---|---|---|---|---|---|---|---|---|---|---|---|---|---|---|---|---|---|---|---|
| | | ***SLC39A4*** | | | | | | | | | | | | | | | | | |
| | | ***SLC39A8*** | | | | | | | | | | | | | | | | | |
| | | ***SLC3A2*** | | | | | | | | | | | | | | | | | |
| | | ***SLC40A1*** | | | | | | | | | | | | | | | | | |
| | | SLC41A2 | | | | | | | | | | | | | | | | | |
| | | ***SLC43A2*** | | | | | | | | | | | | | | | | | |
| | | SLC43A3 | | | | | | | | | | | | | | | | | |
| | | SLC44A2 | | | | | | | | | | | | | | | | | |
| | | SLC45A4 | | | | | | | | | | | | | | | | | |
| | | SLC46A3 | | | | | | | | | | | | | | | | | |
| | | SLC48A1 | | | | | | | | | | | | | | | | | |
| | | SLC4A1AP | | | | | | | | | | | | | | | | | |
| | | SLC4A8 | | | | | | | | | | | | | | | | | |
| | | SLC52A2 | | | | | | | | | | | | | | | | | |
| | | SLC52A3 | | | | | | | | | | | | | | | | | |
| | | SLC6A6 | | | | | | | | | | | | | | | | | |
| | | SLC6A9 | | | | | | | | | | | | | | | | | |
| | | SLC7A3 | | | | | | | | | | | | | | | | | |
| | | SLC7A5 | | | | | | | | | | | | | | | | | |
| | | ***SLC9A3R2*** | | | | | | | | | | | | | | | | | |
| | | SLCO1A5 | | | | | | | | | | | | | | | | | |
| | | ***SLCO2A1*** | | | | | | | | | | | | | | | | | |
| | | SLFN3 | | | | | | | | | | | | | | | | | |
| | | SLFN5 | | | | | | | | | | | | | | | | | |
| | | SLFN9 | | | | | | | | | | | | | | | | | |
| | | ***SLIT2*** | | | | | | | | | | | | | | | | | |
| | | ***SLK*** | | | | | | | | | | | | | | | | | |
| | | SLX4 | | | | | | | | | | | | | | | | | |
| | | SMAD3 | | | | | | | | | | | | | | | | | |
| | | SMAD5 | | | | | | | | | | | | | | | | | |
| | | SMAD6 | | | | | | | | | | | | | | | | | |
| | | SMAGP | | | | | | | | | | | | | | | | | |
| | | ***SMAP2*** | | | | | | | | | | | | | | | | | |
| | | SMC2 | | | | | | | | | | | | | | | | | |
| | | SMIM13 | | | | | | | | | | | | | | | | | |
| | | ***SMO*** | | | | | | | | | | | | | | | | | |
| | | SMOX | | | | | | | | | | | | | | | | | |
| | | SMPD5 | | | | | | | | | | | | | | | | | |
| | | SMPDL3A | | | | | | | | | | | | | | | | | |
| | | SMPDL3B | | | | | | | | | | | | | | | | | |
| | | SMTN | | | | | | | | | | | | | | | | | |
| | | ***SNAP29*** | | | | | | | | | | | | | | | | | |
| | | ***SNCA*** | | | | | | | | | | | | | | | | | |
| | | SNCAIP | | | | | | | | | | | | | | | | | |
| | | ***SNCG*** | | | | | | | | | | | | | | | | | |
| | | ***SNED1*** | | | | | | | | | | | | | | | | | |
| | | SNTA1 | | | | | | | | | | | | | | | | | |
| | | SNX21 | | | | | | | | | | | | | | | | | |
| | | SNX33 | | | | | | | | | | | | | | | | | |
| | | SOCS3 | | | | | | | | | | | | | | | | | |
| | | ***SOCS5*** | | | | | | | | | | | | | | | | | |
| | | ***SOD3*** | | | | | | | | | | | | | | | | | |
| | | SORBS1 | | | | | | | | | | | | | | | | | |
| | | SORBS3 | | | | | | | | | | | | | | | | | |
| | | SOX13 | | | | | | | | | | | | | | | | | |
| | | SOX17 | | | | | | | | | | | | | | | | | |
| | | ***SOX4*** | | | | | | | | | | | | | | | | | |
| | | SP5 | | | | | | | | | | | | | | | | | |
| | | SPAG7 | | | | | | | | | | | | | | | | | |
| | | ***SPARCL1*** | | | | | | | | | | | | | | | | | |
| | | SPATA13 | | | | | | | | | | | | | | | | | |
| | | SPATA25 | | | | | | | | | | | | | | | | | |
| | | SPC24 | | | | | | | | | | | | | | | | | |
| | | SPEN | | | | | | | | | | | | | | | | | |
| | | SPHK1 | | | | | | | | | | | | | | | | | |
| | | SPHK2 | | | | | | | | | | | | | | | | | |
| | | SPICE1 | | | | | | | | | | | | | | | | | |
| | | SPNS2 | | | | | | | | | | | | | | | | | |
| | | SPOCK2 | | | | | | | | | | | | | | | | | |
| | | SPPL2A | | | | | | | | | | | | | | | | | |
| | | SPRY2 | | | | | | | | | | | | | | | | | |
| | | SPRY3 | | | | | | | | | | | | | | | | | |
| | | SPRY4 | | | | | | | | | | | | | | | | | |
| | | SPSB1 | | | | | | | | | | | | | | | | | |
| | | SPSB2 | | | | | | | | | | | | | | | | | |
| | | ***SPTBN1*** | | | | | | | | | | | | | | | | | |
| | | ***SPTBN2*** | | | | | | | | | | | | | | | | | |
| | | ***SPTSSA*** | | | | | | | | | | | | | | | | | |
| | | SPTY2D1 | | | | | | | | | | | | | | | | | |
| | | SQRDL | | | | | | | | | | | | | | | | | |
| | | ***SRGN*** | | | | | | | | | | | | | | | | | |
| | | ***SRMS*** | | | | | | | | | | | | | | | | | |
| | | SRXN1 | | | | | | | | | | | | | | | | | |
| | | SSBP2 | | | | | | | | | | | | | | | | | |
| | | ***ST14*** | | | | | | | | | | | | | | | | | |
| | | ST3GAL1 | | | | | | | | | | | | | | | | | |
| | | ST3GAL5 | | | | | | | | | | | | | | | | | |
| | | ST3GAL6 | | | | | | | | | | | | | | | | | |
| | | ST5 | | | | | | | | | | | | | | | | | |
| | | ST6GALNAC4 | | | | | | | | | | | | | | | | | |
| | | ST8SIA4 | | | | | | | | | | | | | | | | | |
| | | STARD13 | | | | | | | | | | | | | | | | | |
| | | STARD8 | | | | | | | | | | | | | | | | | |
| | | STIL | | | | | | | | | | | | | | | | | |
| | | ***STK10*** | | | | | | | | | | | | | | | | | |
| | | ***STOX1*** | | | | | | | | | | | | | | | | | |
| | | ***STXBP1*** | | | | | | | | | | | | | | | | | |
| | | STXBP5 | | | | | | | | | | | | | | | | | |
| | | ***STXBP6*** | | | | | | | | | | | | | | | | | |
| | | SUB1 | | | | | | | | | | | | | | | | | |
| | | SUCO | | | | | | | | | | | | | | | | | |
| | | SULF2 | | | | | | | | | | | | | | | | | |
| | | ***SUN1*** | | | | | | | | | | | | | | | | | |
| | | ***SUSD2*** | | | | | | | | | | | | | | | | | |
| | | SVIL | | | | | | | | | | | | | | | | | |
| | | ***SWAP70*** | | | | | | | | | | | | | | | | | |
| | | SYBU | | | | | | | | | | | | | | | | | |
| | | ***SYDE1*** | | | | | | | | | | | | | | | | | |
| | | ***SYK*** | | | | | | | | | | | | | | | | | |
| | | ***SYN2*** | | | | | | | | | | | | | | | | | |
| | | ***SYNE3*** | | | | | | | | | | | | | | | | | |
| | | SYNJ2 | | | | | | | | | | | | | | | | | |
| | | SYNM | | | | | | | | | | | | | | | | | |
| | | SYNPO2 | | | | | | | | | | | | | | | | | |
| | | ***SYT7*** | | | | | | | | | | | | | | | | | |
| | | TACC2 | | | | | | | | | | | | | | | | | |
| | | TACC3 | | | | | | | | | | | | | | | | | |
| | | TAF9B | | | | | | | | | | | | | | | | | |
| | | TAGLN | | | | | | | | | | | | | | | | | |
| | | TANC1 | | | | | | | | | | | | | | | | | |
| | | TANC2 | | | | | | | | | | | | | | | | | |
| | | TANK | | | | | | | | | | | | | | | | | |
| | | TARBP1 | | | | | | | | | | | | | | | | | |

| | | | | | | | | | | | | | | | | | | | |
|---|---|---|---|---|---|---|---|---|---|---|---|---|---|---|---|---|---|---|---|
| | | ***TARSL2*** | | | | | | | | | | | | | | | | | |
| | | ***TBC1D16*** | | | | | | | | | | | | | | | | | |
| | | TBC1D2 | | | | | | | | | | | | | | | | | |
| | | TBC1D23 | | | | | | | | | | | | | | | | | |
| | | ***TBC1D4*** | | | | | | | | | | | | | | | | | |
| | | TBC1D9 | | | | | | | | | | | | | | | | | |
| | | TBCC | | | | | | | | | | | | | | | | | |
| | | TBX1 | | | | | | | | | | | | | | | | | |
| | | TCAF2 | | | | | | | | | | | | | | | | | |
| | | TCEAL9 | | | | | | | | | | | | | | | | | |
| | | TCF12 | | | | | | | | | | | | | | | | | |
| | | TCF15 | | | | | | | | | | | | | | | | | |
| | | TCF19 | | | | | | | | | | | | | | | | | |
| | | TCF23 | | | | | | | | | | | | | | | | | |
| | | TCF4 | | | | | | | | | | | | | | | | | |
| | | TCF7L2 | | | | | | | | | | | | | | | | | |
| | | TCTA | | | | | | | | | | | | | | | | | |
| | | TCTN3 | | | | | | | | | | | | | | | | | |
| | | TDRKH | | | | | | | | | | | | | | | | | |
| | | ***TEAD4*** | | | | | | | | | | | | | | | | | |
| | | TEC | | | | | | | | | | | | | | | | | |
| | | TEK | | | | | | | | | | | | | | | | | |
| | | TFDP1 | | | | | | | | | | | | | | | | | |
| | | TGFA | | | | | | | | | | | | | | | | | |
| | | TGFB1I1 | | | | | | | | | | | | | | | | | |
| | | TGFB2 | | | | | | | | | | | | | | | | | |
| | | ***TGFBI*** | | | | | | | | | | | | | | | | | |
| | | TGFBR1 | | | | | | | | | | | | | | | | | |
| | | ***TGM2*** | | | | | | | | | | | | | | | | | |
| | | THBD | | | | | | | | | | | | | | | | | |
| | | ***THBS1*** | | | | | | | | | | | | | | | | | |
| | | THSD1 | | | | | | | | | | | | | | | | | |
| | | THSD7A | | | | | | | | | | | | | | | | | |
| | | TIAM1 | | | | | | | | | | | | | | | | | |
| | | TIFA | | | | | | | | | | | | | | | | | |
| | | TIMELESS | | | | | | | | | | | | | | | | | |
| | | TIMM10B | | | | | | | | | | | | | | | | | |
| | | ***TIMP1*** | | | | | | | | | | | | | | | | | |
| | | TIMP2 | | | | | | | | | | | | | | | | | |
| | | TIMP3 | | | | | | | | | | | | | | | | | |
| | | ***TIMP4*** | | | | | | | | | | | | | | | | | |
| | | ***TINAGL1*** | | | | | | | | | | | | | | | | | |
| | | ***TJP1*** | | | | | | | | | | | | | | | | | |
| | | TK1 | | | | | | | | | | | | | | | | | |
| | | TLE1 | | | | | | | | | | | | | | | | | |
| | | TLE3 | | | | | | | | | | | | | | | | | |
| | | ***TLL1*** | | | | | | | | | | | | | | | | | |
| | | ***TLN2*** | | | | | | | | | | | | | | | | | |
| | | TLR2 | | | | | | | | | | | | | | | | | |
| | | ***TLR4*** | | | | | | | | | | | | | | | | | |
| | | TM4SF1 | | | | | | | | | | | | | | | | | |
| | | TM9SF3 | | | | | | | | | | | | | | | | | |
| | | TMBIM1 | | | | | | | | | | | | | | | | | |
| | | TMC8 | | | | | | | | | | | | | | | | | |
| | | TMCC1 | | | | | | | | | | | | | | | | | |
| | | TMCC3 | | | | | | | | | | | | | | | | | |
| | | TMEFF1 | | | | | | | | | | | | | | | | | |
| | | ***TMEM106A*** | | | | | | | | | | | | | | | | | |
| | | ***TMEM120B*** | | | | | | | | | | | | | | | | | |
| | | ***TMEM123*** | | | | | | | | | | | | | | | | | |
| | | TMEM140 | | | | | | | | | | | | | | | | | |
| | | ***TMEM173*** | | | | | | | | | | | | | | | | | |
| | | ***TMEM176A*** | | | | | | | | | | | | | | | | | |
| | | ***TMEM176B*** | | | | | | | | | | | | | | | | | |
| | | TMEM184B | | | | | | | | | | | | | | | | | |
| | | TMEM184C | | | | | | | | | | | | | | | | | |
| | | TMEM198B | | | | | | | | | | | | | | | | | |
| | | TMEM220 | | | | | | | | | | | | | | | | | |
| | | TMEM237 | | | | | | | | | | | | | | | | | |
| | | TMEM238 | | | | | | | | | | | | | | | | | |
| | | TMEM40 | | | | | | | | | | | | | | | | | |
| | | TMEM44 | | | | | | | | | | | | | | | | | |
| | | ***TMEM47*** | | | | | | | | | | | | | | | | | |
| | | TMEM70 | | | | | | | | | | | | | | | | | |
| | | ***TMEM86A*** | | | | | | | | | | | | | | | | | |
| | | TMEM88 | | | | | | | | | | | | | | | | | |
| | | TMF1 | | | | | | | | | | | | | | | | | |
| | | TMIE | | | | | | | | | | | | | | | | | |
| | | TMLHE | | | | | | | | | | | | | | | | | |
| | | ***TMOD2*** | | | | | | | | | | | | | | | | | |
| | | TMPO | | | | | | | | | | | | | | | | | |
| | | TMSB10 | | | | | | | | | | | | | | | | | |
| | | TMSB4X | | | | | | | | | | | | | | | | | |
| | | TMTC1 | | | | | | | | | | | | | | | | | |
| | | TMTC2 | | | | | | | | | | | | | | | | | |
| | | ***TNFAIP2*** | | | | | | | | | | | | | | | | | |
| | | TNFRSF11A | | | | | | | | | | | | | | | | | |
| | | TNFRSF1A | | | | | | | | | | | | | | | | | |
| | | TNFRSF1B | | | | | | | | | | | | | | | | | |
| | | ***TNFRSF21*** | | | | | | | | | | | | | | | | | |
| | | TNFRSF22 | | | | | | | | | | | | | | | | | |
| | | TNFRSF23 | | | | | | | | | | | | | | | | | |
| | | ***TNFRSF8*** | | | | | | | | | | | | | | | | | |
| | | TNKS2 | | | | | | | | | | | | | | | | | |
| | | TNRC6C | | | | | | | | | | | | | | | | | |
| | | TNS1 | | | | | | | | | | | | | | | | | |
| | | ***TNS2*** | | | | | | | | | | | | | | | | | |
| | | TOM1L2 | | | | | | | | | | | | | | | | | |
| | | ***TOP2B*** | | | | | | | | | | | | | | | | | |
| | | TOPBP1 | | | | | | | | | | | | | | | | | |
| | | TOR2A | | | | | | | | | | | | | | | | | |
| | | TOX | | | | | | | | | | | | | | | | | |
| | | TOX3 | | | | | | | | | | | | | | | | | |
| | | TPCN1 | | | | | | | | | | | | | | | | | |
| | | TPD52L2 | | | | | | | | | | | | | | | | | |
| | | TPK1 | | | | | | | | | | | | | | | | | |
| | | TPM1 | | | | | | | | | | | | | | | | | |
| | | ***TPM2*** | | | | | | | | | | | | | | | | | |
| | | ***TPM4*** | | | | | | | | | | | | | | | | | |
| | | TPRN | | | | | | | | | | | | | | | | | |
| | | ***TPST1*** | | | | | | | | | | | | | | | | | |
| | | TPST2 | | | | | | | | | | | | | | | | | |
| | | TPX2 | | | | | | | | | | | | | | | | | |
| | | TRAF1 | | | | | | | | | | | | | | | | | |
| | | TRAF5 | | | | | | | | | | | | | | | | | |
| | | TRAF6 | | | | | | | | | | | | | | | | | |
| | | TRAIP | | | | | | | | | | | | | | | | | |
| | | ***TRF*** | | | | | | | | | | | | | | | | | |
| | | ***TRIB1*** | | | | | | | | | | | | | | | | | |
| | | ***TRIB3*** | | | | | | | | | | | | | | | | | |
| | | TRIL | | | | | | | | | | | | | | | | | |
| | | TRIM25 | | | | | | | | | | | | | | | | | |
| | | TRIP13 | | | | | | | | | | | | | | | | | |

| | | | | | | | | | | | | | | | | | | | |
|---|---|---|---|---|---|---|---|---|---|---|---|---|---|---|---|---|---|---|---|
| | | TRNAU1AP | | | | | | | | | | | | | | | | | |
| | | TRP53I11 | | | | | | | | | | | | | | | | | |
| | | ***TRP53INP2*** | | | | | | | | | | | | | | | | | |
| | | TRPC3 | | | | | | | | | | | | | | | | | |
| | | ***TRPC6*** | | | | | | | | | | | | | | | | | |
| | | TRPV4 | | | | | | | | | | | | | | | | | |
| | | ***TSC2*** | | | | | | | | | | | | | | | | | |
| | | TSC22D3 | | | | | | | | | | | | | | | | | |
| | | TSPAN2 | | | | | | | | | | | | | | | | | |
| | | TSPAN6 | | | | | | | | | | | | | | | | | |
| | | ***TSPAN7*** | | | | | | | | | | | | | | | | | |
| | | TSPAN9 | | | | | | | | | | | | | | | | | |
| | | TSPO | | | | | | | | | | | | | | | | | |
| | | TSPYL5 | | | | | | | | | | | | | | | | | |
| | | ***TSSC4*** | | | | | | | | | | | | | | | | | |
| | | TSTD3 | | | | | | | | | | | | | | | | | |
| | | TTC28 | | | | | | | | | | | | | | | | | |
| | | TTC3 | | | | | | | | | | | | | | | | | |
| | | ***TTC38*** | | | | | | | | | | | | | | | | | |
| | | TTC7 | | | | | | | | | | | | | | | | | |
| | | TTC9 | | | | | | | | | | | | | | | | | |
| | | TTK | | | | | | | | | | | | | | | | | |
| | | ***TUBA1C*** | | | | | | | | | | | | | | | | | |
| | | ***TUBB2A*** | | | | | | | | | | | | | | | | | |
| | | ***TUBB2B*** | | | | | | | | | | | | | | | | | |
| | | TUBB3 | | | | | | | | | | | | | | | | | |
| | | ***TUBB4B*** | | | | | | | | | | | | | | | | | |
| | | TUBB5 | | | | | | | | | | | | | | | | | |
| | | ***TXNDC5*** | | | | | | | | | | | | | | | | | |
| | | TXNRD3 | | | | | | | | | | | | | | | | | |
| | | TYK2 | | | | | | | | | | | | | | | | | |
| | | TYMS | | | | | | | | | | | | | | | | | |
| | | UAP1 | | | | | | | | | | | | | | | | | |
| | | UBAP2 | | | | | | | | | | | | | | | | | |
| | | UBE2C | | | | | | | | | | | | | | | | | |
| | | ***UBE2G1*** | | | | | | | | | | | | | | | | | |
| | | UBE2I | | | | | | | | | | | | | | | | | |
| | | UBE2T | | | | | | | | | | | | | | | | | |
| | | ***UBQLN2*** | | | | | | | | | | | | | | | | | |
| | | ***UBR7*** | | | | | | | | | | | | | | | | | |
| | | UCHL3 | | | | | | | | | | | | | | | | | |
| | | UCP2 | | | | | | | | | | | | | | | | | |
| | | UGCG | | | | | | | | | | | | | | | | | |
| | | UHRF1 | | | | | | | | | | | | | | | | | |
| | | UHRF1BP1L | | | | | | | | | | | | | | | | | |
| | | ***UNC13A*** | | | | | | | | | | | | | | | | | |
| | | UNC45B | | | | | | | | | | | | | | | | | |
| | | UNC5B | | | | | | | | | | | | | | | | | |
| | | UNC93B1 | | | | | | | | | | | | | | | | | |
| | | UPP1 | | | | | | | | | | | | | | | | | |
| | | ***USP1*** | | | | | | | | | | | | | | | | | |
| | | USP31 | | | | | | | | | | | | | | | | | |
| | | USP46 | | | | | | | | | | | | | | | | | |
| | | USP53 | | | | | | | | | | | | | | | | | |
| | | UVSSA | | | | | | | | | | | | | | | | | |
| | | VAMP1 | | | | | | | | | | | | | | | | | |
| | | VAMP5 | | | | | | | | | | | | | | | | | |
| | | VANGL1 | | | | | | | | | | | | | | | | | |
| | | VASH1 | | | | | | | | | | | | | | | | | |
| | | VASP | | | | | | | | | | | | | | | | | |
| | | ***VEPH1*** | | | | | | | | | | | | | | | | | |
| | | ***VIL1*** | | | | | | | | | | | | | | | | | |
| | | VLDLR | | | | | | | | | | | | | | | | | |
| | | VPS53 | | | | | | | | | | | | | | | | | |
| | | VSIR | | | | | | | | | | | | | | | | | |
| | | ***VSTM5*** | | | | | | | | | | | | | | | | | |
| | | VWA1 | | | | | | | | | | | | | | | | | |
| | | ***VWF*** | | | | | | | | | | | | | | | | | |
| | | ***WARS*** | | | | | | | | | | | | | | | | | |
| | | WDHD1 | | | | | | | | | | | | | | | | | |
| | | WDR62 | | | | | | | | | | | | | | | | | |
| | | WDR76 | | | | | | | | | | | | | | | | | |
| | | WDR78 | | | | | | | | | | | | | | | | | |
| | | ***WEE1*** | | | | | | | | | | | | | | | | | |
| | | WFDC1 | | | | | | | | | | | | | | | | | |
| | | WHAMM | | | | | | | | | | | | | | | | | |
| | | ***WLS*** | | | | | | | | | | | | | | | | | |
| | | WNK1 | | | | | | | | | | | | | | | | | |
| | | WNK4 | | | | | | | | | | | | | | | | | |
| | | WNT5A | | | | | | | | | | | | | | | | | |
| | | WNT9A | | | | | | | | | | | | | | | | | |
| | | WSB1 | | | | | | | | | | | | | | | | | |
| | | XDH | | | | | | | | | | | | | | | | | |
| | | XPC | | | | | | | | | | | | | | | | | |
| | | ***XPO4*** | | | | | | | | | | | | | | | | | |
| | | XRCC2 | | | | | | | | | | | | | | | | | |
| | | XRCC3 | | | | | | | | | | | | | | | | | |
| | | ***XRCC5*** | | | | | | | | | | | | | | | | | |
| | | YARS | | | | | | | | | | | | | | | | | |
| | | ***YBX1*** | | | | | | | | | | | | | | | | | |
| | | YBX3 | | | | | | | | | | | | | | | | | |
| | | YIPF2 | | | | | | | | | | | | | | | | | |
| | | YPEL2 | | | | | | | | | | | | | | | | | |
| | | YRDC | | | | | | | | | | | | | | | | | |
| | | ZBTB43 | | | | | | | | | | | | | | | | | |
| | | ***ZBTB46*** | | | | | | | | | | | | | | | | | |
| | | ZC2HC1A | | | | | | | | | | | | | | | | | |
| | | ZC3H12A | | | | | | | | | | | | | | | | | |
| | | ZC3H12C | | | | | | | | | | | | | | | | | |
| | | ZCCHC17 | | | | | | | | | | | | | | | | | |
| | | ZCCHC24 | | | | | | | | | | | | | | | | | |
| | | ZCWPW1 | | | | | | | | | | | | | | | | | |
| | | ***ZDHHC14*** | | | | | | | | | | | | | | | | | |
| | | ZDHHC15 | | | | | | | | | | | | | | | | | |
| | | ZFAND5 | | | | | | | | | | | | | | | | | |
| | | ZFP101 | | | | | | | | | | | | | | | | | |
| | | ZFP26 | | | | | | | | | | | | | | | | | |
| | | ***ZFP28*** | | | | | | | | | | | | | | | | | |
| | | ZFP281 | | | | | | | | | | | | | | | | | |
| | | ***ZFP366*** | | | | | | | | | | | | | | | | | |
| | | ZFP367 | | | | | | | | | | | | | | | | | |
| | | ZFP423 | | | | | | | | | | | | | | | | | |
| | | ZFP462 | | | | | | | | | | | | | | | | | |
| | | ***ZFP521*** | | | | | | | | | | | | | | | | | |
| | | ZFP54 | | | | | | | | | | | | | | | | | |
| | | ***ZFP697*** | | | | | | | | | | | | | | | | | |
| | | ***ZFP703*** | | | | | | | | | | | | | | | | | |
| | | ZFP704 | | | | | | | | | | | | | | | | | |
| | | ZFP711 | | | | | | | | | | | | | | | | | |
| | | ***ZFP787*** | | | | | | | | | | | | | | | | | |
| | | ZFP827 | | | | | | | | | | | | | | | | | |
| | | ZFP948 | | | | | | | | | | | | | | | | | |
| | | ZFP958 | | | | | | | | | | | | | | | | | |

| | | | | | | | | | | | | | | | | | | | |
|---|---|---|---|---|---|---|---|---|---|---|---|---|---|---|---|---|---|---|---|
| | | ZFPM1 | | | | | | | | | | | | | | | | | |
| | | ZFX | | | | | | | | | | | | | | | | | |
| | | ZGRF1 | | | | | | | | | | | | | | | | | |
| | | ZIC3 | | | | | | | | | | | | | | | | | |
| | | ZIK1 | | | | | | | | | | | | | | | | | |
| | | ZKSCAN4 | | | | | | | | | | | | | | | | | |
| | | ZMIZ1 | | | | | | | | | | | | | | | | | |
| | | ***ZNRF1*** | | | | | | | | | | | | | | | | | |
| | | ZRANB3 | | | | | | | | | | | | | | | | | |
| | | ZWINT | | | | | | | | | | | | | | | | | |
| | | ***ZYX*** | | | | | | | | | | | | | | | | | |
| | | ZZEF1 | | | | | | | | | | | | | | | | | |

**Supplemental Table 1A. Identification of altered genes in CCM models with various validations.** A total of 9 CCM studies were analyzed to identify genes that overlapped as perturbed in various CCM models. For Datasets, non-human genes were converted to human homologs before processing and all genes capitalized to ensure overlaps were not misidentified due to case changes. Studies are divided into columns based on detection method, background strain (i.e. mouse BMEC Ccm1/Krit1 ECKO) and organism. Genes that are italicized and bolded are genes duplicated in another column. Overlaps within the same study (EX: First 6 columns) were not considered “validated”, only overlaps within two different studies are considered "validated". Abbreviations: Differentially expressed genes (DEG's), human umbilical vein endothelial cells (HUVEC), Human Brain Microvascular Endothelial Cells (HBMVEC). Reference numbering can be found in Supplemental Table 7.
