# Supplementary material for: Comparative omics of CCM signaling complex (CSC)
Source: Chin Neurosurg J. 2020 Jan 15;6:4. doi: 10.1186/s41016-019-0183-6 (PMC7398211; doi:10.1186/s41016-019-0183-6)
Supplement: Supplementary file 2 — Additional file 2: Table S1B. Summary of Identified altered genes in CCM models with various validations. A summary of "validated genes" across 9 CCM studies were analyzed to identify genes that overlapped as perturbed in various CCM models. Studies are divided into columns based on detection method, background strain (i.e. mouse BMEC Ccm1/Krit1 ECKO) and organism. Genes that are bolded are genes duplicated in two studies, genes italicized are duplicated in three studies, and gene underlined is duplicated in 4 studies. The largest cohort (First 6 columns in supplemental table 1A) are not included in this table as they have significant overlaps within each column, complicating the summarized data, but were counted as "validated" if gene(s) was found in another cohort (EX: TUBB4B was identified in 3 cohorts, shown here in this table, as well as the largest cohort (not shown in this table) therefore making it validated across 4 studies). Abbreviations: Differentially expressed genes (DEG's), human umbilical vein endothelial cells (HUVEC), Human Brain Microvascular Endothelial Cells (HBMVEC). Reference numbering can be found in Additional file 11. [file 41016_2019_183_MOESM2_ESM.pdf]

Summary of Identified altered genes in CCM models with various validations.

CCM1 delimits relationship of PTB/Ph domains (1)	proteomic analysis of cerebral cavernous malformation in HUVEC's (7)	Biomechanics of endothelial tubule formation differentially modulated by cerebral cavernous malformation proteins (4)	Target: (*H.sapiens*) FROM zebrafish De pick target proteins (5)	Target: (*H.sapiens*) FROM *C.Elegans* De pick target proteins (5)	Proteomic identification of the CSC (8)	Proteomic analysis of Krit1 Loss-of Function (3)	CCM3 Gene Network (6)	ccm1 proteomics in HBMVEC's (9)	CCM2 PROTEOMICS in HBMVEC's (9)	CCM3 PROTEOMICS in HBMVEC's (9)	ccm1 RNA in zebrafish (9)	CCM2 RNA in zebrafish (9)	CCM3 RNA in zebrafish (9)
**DAB2**	**SEPT7**	**ADD1**	**ABCB1**	**AKT3**		*Actb*	**APP**	**CDH5**	**CDH5**	**SUN1**	**APBA2**	**CCM2**	
**DOK4**	*ACTB*	**COL8A1**	**CSF1R**	**APP**	**Proteins Identified from SDS-PAGE Gel and ESI-MS**	**Ckb**	**APLP1**	**CAPN2**	**SUN1**	PRKCSH	*ITGB4*	*ITGB4*	
**NUMB**	**ANXA1**	**FN1**	**CYP27A1**	**BCL2**	**Proteins that selectively interact with FLAG-OSM**	*Tpm4*	**CDC25A**	MCM3	MCM3	**CAV1**	**TJP1**	**TJP1**	
**PLEK**	**ANXA2**	**ITGA2**		**BCL2L1**	HSP90	Tubb4b	**CDC25B**	**SUN1**	CNN3	DDB1	**CCL2**		
**RGS12**	**ANXA5**	*ITGB4*	**FLT1**	**BCL2L2**	TUBA4A	**Vim**	**CDC25C**	GLOD4	PSMEI	GLOD4			
**TBC1D4**	**ANXA6**	**LAMA5**	**KIT**	**BRSK1**	**TUBA1C**	**Eno1**	**CDC42**	**GAPDH**	**NOS3**	PSMEI			
**TLN2**	**ARF3**	**MYO1B**	**LCK**	**CAMK1D**	**TUBB4A**	**Gapdh**	**ARHGEF5**	CNN3	PRKCSH	**PLOD2**			
**TNS2**	**ARF1**	*PLEC*	**PDGFRA**	**CAMK2A**	**PDCD10**	**Calr**	**ARHGEF15**	**NOS3**	DDB1				
	**ARF4**	**SPTAN1**	**PLK1**	**CAMK2B**	**Proteins that nonspecifically interact with anti-FLAG antibody-conjugated beads**	**TPM3**	**ARHGEF26**	PRKCSH	**HSPB1**				
	**CALR**	**SPTBN2**	**PPP3CA**	**CCR5**			**NGEF**	DDB1	**SFPQ**				
	**CAV1**	**THBS1**	**RARA**	**CDC25A**	**Proteins Identified by MudPIT Analysis That Selectively Interact with FLAG-OSM**		**DLL1**	**CAV1**					
	**CDC42**	**CDH5**	**RXRA**	**CDC25C**	**Previously identified CCM complex members**		**DLL4**						
	**CLIC4**	**CFL1**	**SNCA**	**CYP27A1**	**PDCD10**		**JAG2**						
	**CNN2**	**CNN2**	TUBB4B	**DNM1**	**Cytoskeletal proteins**		**RORA**						
	**COPA**	**FLNB**	**USP1**	**DPP4**	**MYH10**		**RORC**						
	**EF1A1**		**WEE1**	**EPHA2**	**TUBB4A**		**ARHGAP30**						
	**EHD4**		**TUBA1A**	**FYN**	TUBB4B		**ARHGAP31**						
	**FAS**		**TUBB4A**	**G6PD**	**CFL1**		**SDC1**						
	**FLNA**			**LTB4R**	TUBA4A		**SDC4**						
	**FLNB**			**MAP2K5**	**TUBA1C**		**EXOC6B**						
	**FLNC**			**MAP4K4**	**TUBA1A**		**STXBP6**						
	**FSCN1**			**MELK**	**Protein translation and folding**		**FERMT3**						
	**GSTO1**			**MMP14**	**EF1A1**		**CCT2**						
	**HSPB1**			**MMP2**	**DNAJB6**								
	**ICAM1**			**NPC1L1**	**CCT2**								
	**LAMA4**			**NQO1**	HSP90								
	**LDHB**			**NR1H4**	**Signaling**								
	**MMRN2**			**PBK**	**GNB4**								
	**MT2**			**PPP3R1**	**Vesicular transport proteins**								
	**MYH9**			**PRKCG**	**COPA**								
	**MYO1C**			**PRKCH**	**ARF1**								
	**PDIA6**			**PSIP1**	**ARF2**								
	**PIR**			**PTGES**	**ARF3**								
	PLEC			**PTPN1**	**ARF4**								
	**PLOD1**			**RARA**	**Metabolism and biosynthetic proteins**								
	**PLOD2**			**RBP1**	**ENO1**								
	**PTMA**			**RORA**	**CAD**								
	**PTRF**			**RORC**	**PAICS**								
	**PXDN**			**RXRA**	**Miscellaneous**								
	**RCN1**			**SLK**									
	**SDPR**			**TLR4**									
	**SFPQ**			**TOP2B**									
	**TGM2**												
	**TPM2**												
	**TPM3**												
	TPM4												
	**VWF**												
	**XRCC5**												
	**ZYX**												

Supplemental Table 1B. Summary of Identified altered genes in CCM models with various validations. A summary of "validated genes" across 9 CCM studies were analyzed to identify genes that overlapped as perturbed in various CCM models. Studies are divided into columns based on detection method, background strain (i.e. mouse BMEC Ccm1/Krit1 ECKO) and organism. Genes that are bolded are genes duplicated in two studies, genes italicized are duplicated in three studies, and gene undelined is duplicated in 4 studies. The largest cohort (First 6 columns in supplemental table 1A) are not included in this table as they have significant overlaps within each column, complicating the summarized data, but were counted as "validated" if gene(s) was found in another cohort (EX: TUBB4B was identified in 3 cohorts, shown here in this table, as well as the largest cohort (not shown in this table) therefore making it validated across 4 studies). Abbreviations: Differentially expressed genes (DEG's), human umbilical vein endothelial cells (HUVEC), Human Brain Microvascular Endothelial Cells (HBMVEC). Reference numbering can be found in Supplemental Table 7.
