# Supplementary material for: Comparative omics of CCM signaling complex (CSC)
Source: Chin Neurosurg J. 2020 Jan 15;6:4. doi: 10.1186/s41016-019-0183-6 (PMC7398211; doi:10.1186/s41016-019-0183-6)
Supplement: Supplementary file 3 — Additional file 3: Table S2. Details of identified altered genes in CCM models with various validations. Genes validated were compiled into A) 2 validations, B) 3 validations and C) 4 validation groups. Details included here include additional sorting of data by detection method, background strain (i.e. mouse BMEC Ccm1/Krit1 ECKO) and organism. Details provided include the protein, details of detection method, strain and organism, and a list of the corresponding literature the data was extracted from. For a list of the references and corresponding numbers used, please reference supplemental table 7. Genomic methods involved detection through RT-PCR or other DNA detection methods, proteomics methods involved pull-down assays and subsequent mass spectrometry applications while transcriptomics methods involved identification of genes through various RNA detection methods. Abbreviations: NVU: Neuro Vascular unit from surgically resected lesions. [file 41016_2019_183_MOESM3_ESM.pdf]

Details of identified altered genes in CCM models with 2 validations

Gene	Detection method 1	Detection method 2	References
ABCB1	GENOMICS(Mouse and C. elegans, CCM3)	Transcriptome (Zebrafish, CCM2)	2,5
ADD1	Transcriptome (Human, NVU)	Transcriptome (Human, CCM1,2,3)	2,4
AKT3	Transcriptome (Human, NVU)	Transcriptome (C. elegans, CCM1,3)	2,5
ANXA1	Transcriptome (Human, NVU)	PROTEOMICS (HUMAN CCM1,2,3)	2,7
ANXA2	Transcriptome (Mouse, CCM1,3)	PROTEOMICS (HUMAN CCM1,2,3)	2,7
ANXA5	Transcriptome (Mouse, CCM1)	PROTEOMICS (HUMAN CCM1,2,3)	2,7
ANXA6	Transcriptome (Mouse, CCM1)	PROTEOMICS (HUMAN CCM1,2,3)	2,7
APBA2	Transcriptome (Human, NVU)	Transcriptome (Human and Zebrafish, CCM1)	2,9
APLP1	Transcriptome (Mouse, CCM1)	Transcriptome (C. elegans, CCM3)	2,6
APP	Transcriptome (C. elegans, CCM1,3)	Transcriptome (C. elegans, CCM3)	5,6
ARF1	PROTEOMICS (HUMAN CCM1,2,3)	Proteomics with FLAG-OSM	7,8
ARF2	Transcriptome (Mouse, CCM1,3)	Proteomics with FLAG-OSM	2,8
ARF3	PROTEOMICS (HUMAN CCM1,2,3)	Proteomics with FLAG-OSM	7,8
ARF4	PROTEOMICS (HUMAN CCM1,2,3)	Proteomics with FLAG-OSM	7,8
ARHGAP30	Transcriptome (Human, NVU)	Transcriptome (C. elegans, CCM3)	2,6
ARHGAP31	Transcriptome (Mouse, CCM1)	Transcriptome (C. elegans, CCM3)	2,6
ARHGEF15	Transcriptome (Mouse, CCM1)	Transcriptome (C. elegans, CCM3)	2,6
ARHGEF26	Transcriptome (Mouse, CCM1)	Transcriptome (C. elegans, CCM3)	2,6
ARHGEF5	Transcriptome (Mouse, CCM1)	Transcriptome (C. elegans, CCM3)	2,6
BCL2	Transcriptome (Mouse, CCM1,3)	Transcriptome (C. elegans, CCM1,3)	2,5
BCL2L1	Transcriptome (Mouse, CCM1)	Transcriptome (C. elegans, CCM1,3)	2,5
BCL2L2	Transcriptome (Mouse, CCM1)	Transcriptome (C. elegans, CCM1,3)	2,5
BRSK1	Transcriptome (Human, NVU)	Transcriptome (C. elegans, CCM1,3)	2,5
CAD	Transcriptome (Mouse, CCM1)	Proteomics with FLAG-OSM	2,8
CALR	PROTEOMICS (Mouse and Human, CCM1,2,3)	PROTEOMICS (HUMAN CCM1,2,3)	3,7
CAMK1D	Transcriptome (Mouse, CCM1)	Transcriptome (C. elegans, CCM1,3)	2,5
CAMK2A	Transcriptome (Human, NVU and Mouse, CCM1)	Transcriptome (C. elegans, CCM1,3)	2,5
CAMK2B	Transcriptome (Human, NVU)	Transcriptome (C. elegans, CCM1,3)	2,5
CAPN2	Transcriptome (Mouse, CCM1,3)	Proteomics (Human and Zebrafish, CCM1)	2,9
CAV1	PROTEOMICS (HUMAN, CCM1,2,3)	Proteomics (Human and Zebrafish, CCM1,3)	7,9
CCL2	Transcriptome (Mouse, CCM3)	Transcriptome (Human and Zebrafish CCM1)	2,9
CCM2	PROTEOMICS (HUMAN CCM1,2,3)	Transcriptome (Human and Zebrafish CCM2)	2,9
CCR5	Transcriptome (Human, NVU)	Transcriptome (C. elegans, CCM1,3)	2,5
CCT2	Transcriptome (C. elegans, CCM3)	Proteomics with FLAG-OSM	6,8
CDC25A	Transcriptome (C. elegans, CCM1,3)	Transcriptome (C. elegans, CCM3)	5,6
CDC25B	Transcriptome (Mouse, CCM1)	Transcriptome (C. elegans, CCM3)	2,6
CDC25C	Transcriptome (Mouse, CCM1)	Transcriptome (C. elegans, CCM3)	2,6
CDC42	Transcriptome (C. elegans, CCM3)	PROTEOMICS (HUMAN CCM1,2,3)	6,7
CDH5	Transcriptome (Human, CCM1,2,3)	Proteomics (Human and Zebrafish, CCM1,2)	4,9
CFL1	Transcriptome (Human, CCM1,2,3)	Proteomics with FLAG-OSM	4,8
Ckb	Transcriptome (Mouse, CCM1)	PROTEOMICS (Mouse and Human, CCM1,2,3)	2,3
CLIC4	Transcriptome (Mouse, CCM1)	PROTEOMICS (Mouse and Human, CCM1,2,3)	2,7
CNN2	Transcriptome (Human, CCM1,2,3)	PROTEOMICS (HUMAN CCM1,2,3)	4,7
COL8A1	Transcriptome (Human, NVU)	Transcriptome (Human, CCM1,2,3)	2,4
COPA	PROTEOMICS (HUMAN CCM1,2,3)	Proteomics with FLAG-OSM	7,8
CSF1R	Transcriptome (Human, NVU)	Transcriptome (Zebrafish, CCM2)	2,5
CYP27A1	Transcriptome (Human, NVU)	Transcriptome (Zebrafish, CCM2 and C. elegans, CCM1,3)	2,5
DAB2	Genomic (Human CCM1)	Transcriptome (Human, NVU and Mouse, CCM1)	1,2
DLL1	Transcriptome (Mouse, CCM1)	Transcriptome (C. elegans, CCM3)	2,6
DLL4	Transcriptome (Human and Mouse, CCM1, 2, 3)	Transcriptome (C. elegans, CCM3)	2,6
DNAJB6	Transcriptome (Mouse, CCM1)	Proteomics with FLAG-OSM	2,8
DNM1	Transcriptome (Human, NVU)	Transcriptome (C. elegans, CCM1,3)	2,5
DOK4	Genomic (Human CCM1)	Transcriptome (Mouse, CCM1,3)	1,2
DPP4	Transcriptome (Mouse, CCM3)	Transcriptome (C. elegans, CCM1,3)	2,5
EF1A1	PROTEOMICS (HUMAN CCM1,2,3)	Proteomics with FLAG-OSM	7,8
EHD4	Transcriptome (Mouse, CCM1)	PROTEOMICS (Mouse and Human, CCM1,2,3)	2,7
ENO1	PROTEOMICS (Mouse and Human, CCM1,2,3)	Proteomics with FLAG-OSM	3,8
EPHA2	Transcriptome (Mouse, CCM1)	Transcriptome (C. elegans, CCM1,3)	2,5
EXOC6B	Transcriptome (Human, NVU)	Transcriptome (C. elegans, CCM3)	2,6
FAS	Transcriptome (Human, NVU and Mouse CCM3)	PROTEOMICS (Mouse and Human, CCM1,2,3)	2,7
FERMT3	Transcriptome (Human, NVU)	Transcriptome (C. elegans, CCM3)	2,6
FLNA	Transcriptome (Mouse, CCM1)	PROTEOMICS (Mouse and Human, CCM1,2,3)	2,7
FLNB	Transcriptome (Human, CCM1,2,3)	PROTEOMICS (HUMAN CCM1,2,3)	4,7
FLNC	Transcriptome (Mouse, CCM3)	PROTEOMICS (HUMAN CCM1,2,3)	2,7
FLT1	Transcriptome (Human, NVU and Mouse CCM1)	Transcriptome (Zebrafish, CCM2)	2,5
FN1	Transcriptome (Human, NVU and Mouse CCM1)	Transcriptome (Human, CCM1,2,3)	2,4
FSCN1	Transcriptome (Mouse, CCM1,3)	PROTEOMICS (HUMAN CCM1,2,3)	2,7
FYN	Transcriptome (Mouse, CCM1,3)	Transcriptome (C. elegans, CCM1,3)	2,5
G6PD	Transcriptome (Mouse, CCM1,3 and C. elegans, CCM3)	Transcriptome (C. elegans, CCM1,3)	2,5
GAPDH	PROTEOMICS (Mouse and Human, CCM1,2,3)	Proteomics (Human and Zebrafish, CCM1)	3,9
GNB4	Transcriptome (Mouse, CCM1)	Proteomics with FLAG-OSM	2,8
GSTO1	Transcriptome (Mouse, CCM1)	PROTEOMICS (HUMAN CCM1,2,3)	2,7
HSPB1	PROTEOMICS (HUMAN CCM1,2,3)	Proteomics (Human and Zebrafish, CCM2)	7,9
ICAM1	PROTEOMICS (HUMAN, CCM1,2,3) and Transcriptome (Mouse, CCM1)	PROTEOMICS (HUMAN CCM1,2,3)	2,7
ITGA2	Transcriptome (Mouse, CCM3)	Transcriptome (Human, CCM1,2,3)	2,4
JAG2	Transcriptome (Human, NVU)	Transcriptome (C. elegans, CCM3)	2,6
KIT	Transcriptome (Mouse, CCM1,3)	Transcriptome (Zebrafish, CCM2)	2,5
LAMA4	Transcriptome (Human, NVU and Mouse CCM1)	PROTEOMICS (HUMAN CCM1,2,3)	2,7
LAMA5	Transcriptome (Mouse, CCM1)	Transcriptome (Human, CCM1,2,3)	2,4
LCK	Transcriptome (Human, NVU)	Transcriptome (Zebrafish, CCM2)	2,5
LDHB	Transcriptome (Mouse, CCM1)	PROTEOMICS (HUMAN CCM1,2,3)	2,7
LTB4R	Transcriptome (Human, NVU)	Transcriptome (C. elegans, CCM1,3)	2,5
MAP2K5	Transcriptome (Mouse, CCM3)	Transcriptome (C. elegans, CCM1,3)	2,5
MAP4K4	Transcriptome (Mouse, CCM1)	Transcriptome (C. elegans, CCM1,3)	2,5
MELK	Transcriptome (Mouse, CCM1)	Transcriptome (C. elegans, CCM1,3)	2,5
MMP14	Transcriptome (Human, NVU and Mouse, CCM1)	Transcriptome (C. elegans, CCM1,3)	2,5
MMP2	Transcriptome (Human, NVU and Mouse, CCM1)	Transcriptome (C. elegans, CCM1,3)	2,5
MMRN2	Transcriptome (Mouse, CCM1)	PROTEOMICS (HUMAN CCM1,2,3)	2,7
MT2	Transcriptome (Mouse, CCM1)	PROTEOMICS (HUMAN CCM1,2,3)	2,7
MYH10	Transcriptome (Mouse, CCM1)	Proteomics with FLAG-OSM (Mouse and Human, CCM1,2,3)	2,8
MYH9	Transcriptome (Mouse, CCM1)	PROTEOMICS (HUMAN CCM1,2,3)	2,7
MYO1B	Transcriptome (Mouse, CCM1)	Transcriptome (Human, CCM1,2,3)	2,4
MYO1C	Transcriptome (Mouse, CCM1)	PROTEOMICS (HUMAN CCM1,2,3)	2,7
NGEF	Transcriptome (Human, NVU)	Transcriptome (C. elegans, CCM3)	2,6
NOS3	Transcriptome (Mouse, CCM1)	Proteomics (Human and Zebrafish, CCM1,2)	2,9
NPC1L1	Transcriptome (Human, NVU)	Transcriptome (C. elegans, CCM1,3)	2,5
NQO1	Transcriptome (Mouse, CCM1,3)	Transcriptome (C. elegans, CCM1,3)	2,5
NR1H4	Transcriptome (Human, NVU)	Transcriptome (C. elegans, CCM1,3)	2,5
NUMB	Genomic (Human CCM1)	Transcriptome (Mouse, CCM1)	1,2
PAICS	Transcriptome (Mouse, CCM1)	Proteomics with FLAG-OSM (Mouse and Human, CCM1,2,3)	2,8
PBK	Transcriptome (Mouse, CCM1)	Transcriptome (C. elegans, CCM1,3)	2,5
PDCD10	PROTEOMICS (HUMAN CCM1,2,3 and) and Transcriptome (Mouse and C. elegans, CCM3)	Proteomics with FLAG-OSM (Mouse and Human, CCM1,2,3)	2,8
PDGFRA	Transcriptome (Human, NVU and Mouse, CCM1 and Mouse and C. elegans, CCM3)	Transcriptome (Zebrafish, CCM2)	2,5
PDIA6	Transcriptome (Mouse, CCM1)	PROTEOMICS (HUMAN CCM1,2,3)	2,7
PIR	Transcriptome (Mouse, CCM1)	PROTEOMICS (HUMAN CCM1,2,3)	2,7
PLEK	Genomic (Human CCM1)	Transcriptome (Human, NVU)	1,2
PLK1	Transcriptome (Mouse, CCM1 and Mouse and C. elegans, CCM3)	Transcriptome (Zebrafish, CCM2)	2,5
PLOD1	Transcriptome (Mouse, CCM1,3)	PROTEOMICS (HUMAN CCM1,2,3)	2,7
PLOD2	PROTEOMICS (HUMAN CCM1,2,3)	Proteomics (Human and Zebrafish, CCM3)	7,9
PPP3CA	Transcriptome (Human, NVU)	Transcriptome (Zebrafish, CCM2)	2,5
PPP3R1	Transcriptome (Human, NVU)	Transcriptome (C. elegans, CCM1,3)	2,5
PRKCG	Transcriptome (Mouse, CCM1)	Transcriptome (C. elegans, CCM1,3)	2,5
PRKCH	Transcriptome (Mouse, CCM1)	Transcriptome (C. elegans, CCM1,3)	2,5
PSIP1	Transcriptome (Human, NVU)	Transcriptome (C. elegans, CCM1,3)	2,5
PTGES	Transcriptome (Mouse, CCM1)	Transcriptome (C. elegans, CCM1,3)	2,5
PTMA	Transcriptome (Mouse, CCM3)	PROTEOMICS (HUMAN CCM1,2,3)	2,7
PTPN1	Transcriptome (Mouse, CCM1)	Transcriptome (C. elegans, CCM1,3)	2,5
PTRF	Transcriptome (Mouse, CCM1)	PROTEOMICS (HUMAN CCM1,2,3)	2,7
PXDN	Transcriptome (Mouse, CCM1,3)	PROTEOMICS (HUMAN CCM1,2,3)	2,7
RBP1	Transcriptome (Human, NVU)	Transcriptome (C. elegans, CCM1,3)	2,5
RCN1	Transcriptome (Human, NVU)	PROTEOMICS (HUMAN CCM1,2,3)	2,7
RGS12	Genomic (Human CCM1)	Transcriptome (Mouse, CCM1)	1,2
RORA	Transcriptome (C. elegans, CCM1,3)	Transcriptome (C. elegans, CCM3)	5,6
RORC	Transcriptome (C. elegans, CCM1,3)	Transcriptome (C. elegans, CCM3)	5,6
SDC1	Transcriptome (Human, NVU and Mouse, CCM1)	Transcriptome (C. elegans, CCM3)	2,6
SDC4	Transcriptome (Mouse, CCM1)	Transcriptome (C. elegans, CCM3)	2,6
SDPR	Transcriptome (Mouse, CCM1)	PROTEOMICS (HUMAN CCM1,2,3)	2,7
SEPT7	Transcriptome (Mouse, CCM1)	PROTEOMICS (HUMAN CCM1,2,3)	2,7
SFPQ	PROTEOMICS (HUMAN CCM1,2,3)	Proteomics (Human and Zebrafish, CCM2)	7,9

SLK	Transcriptome (Mouse, CCM1,3)	Transcriptome (C. elegans, CCM1,3)	2,5
SNCA	Transcriptome (Mouse, CCM1)	Transcriptome (Zebrafish, CCM2)	2,5
SPTAN1	Transcriptome (Mouse, CCM3)	Transcriptome (Human, CCM1,2,3)	2,4
SPTBN2	Transcriptome (Mouse, CCM1)	Transcriptome (Human, CCM1,2,3)	2,4
STXBP6	Transcriptome (Mouse, CCM1)	Transcriptome (C. elegans, CCM3)	2,6
TBC1D4	Genomic (Human CCM1)	Transcriptome (Mouse, CCM1)	1,2
TGM2	Transcriptome (Mouse, CCM1)	PROTEOMICS (HUMAN CCM1,2,3)	2,7
THBS1	Transcriptome (Mouse, CCM1)	Transcriptome (Human, CCM1,2,3)	2,4
TJP1	PROTEOMICS (HUMAN CCM1,2,3) and Transcriptome (Mouse, CCM1)	Transcriptome (Human and Zebrafish CCM1)	2,9
TLN2	Genomic (Human CCM1)	Transcriptome (Mouse, CCM1)	1,2
TLR4	Transcriptome (Mouse, CCM1)	Transcriptome (C. elegans, CCM1,3)	2,5
TNS2	Genomic (Human CCM1)	Transcriptome (Mouse, CCM1,3)	1,2
TOP2B	Transcriptome (Mouse, CCM1)	Transcriptome (C. elegans, CCM1,3)	2,5
TPM2	Transcriptome (Mouse, CCM1)	PROTEOMICS (HUMAN CCM1,2,3)	2,7
TPM3	PROTEOMICS (Mouse and Human, CCM1,2,3)	PROTEOMICS (HUMAN CCM1,2,3)	3,7
TUBA1C	Transcriptome (Mouse, CCM1)	Proteomics with FLAG-OSM (Mouse and Human, CCM1,2,3)	2,8
TUBB4A	Transcriptome (Zebrafish, CCM2)	Proteomics with FLAG-OSM (Mouse and Human, CCM1,2,3)	5,8
USP1	Transcriptome (Human, NVU and Mouse, CCM1)	Transcriptome (Zebrafish, CCM2)	2,5
Vim	PROTEOMICS (HUMAN CCM1,2,3)	PROTEOMICS (Mouse and Human, CCM1,2,3)	2,3
VWF	Transcriptome (Mouse, CCM1)	PROTEOMICS (HUMAN CCM1,2,3)	2,7
WEE1	Transcriptome (Mouse, CCM1)	Transcriptome (Zebrafish, CCM2)	2,5
XRCC5	Transcriptome (Mouse, CCM1)	PROTEOMICS (HUMAN CCM1,2,3)	2,7
ZYX	Transcriptome (Mouse, CCM1)	PROTEOMICS (HUMAN CCM1,2,3)	2,7

Details of identified altered genes in CCM models with 3 validations

Gene	detection method 1	detection method 2	detection method 3	References
ACTB	Transcriptome (MOUSE ccm1)	PROTEOMICS (MOUSE AND HUMAN CCM1)	PROTEOMICS (HUMAN CCM1,2,3)	2,3,7
ITGB4	Transcriptome (Mouse, CCM1)	Transcriptome (Human, CCM1,2,3)	Transcriptome (Human and Zebrafish CCM1,2)	2,4,9
PLEC	Transcriptome (Mouse, CCM1,3)	Transcriptome (Human, CCM1,2,3)	PROTEOMICS (HUMAN CCM1,2,3)	2,4,7
TPM4	Transcriptome (Mouse, CCM1)	PROTEOMICS (Mouse and Human, CCM1,2,3)	PROTEOMICS (HUMAN CCM1,2,3)	2,3,7

Details of identified altered genes in CCM models with 4 validations

Gene	detection method 1	detection method 2	detection method 3	detection method 4	References
TUBB4B	Transcriptome (Mouse, CCM1)	PROTEOMICS (Mouse and Human, CCM1,2,3)	Transcriptome (Zebrafish, CCM2)	Proteomics with FLAG-OSM (Mouse and Human, CCM1,2,3)	2,3,5,8

Supplemental Table 2. Details of identified altered genes in CCM models with various validations. Genes validated were compiled into **A)** 2 validations, **B)** 3 validations and **C)** 4 validation groups. Details included here include additional sorting of data by detection method, background strain (i.e. mouse BMEC Ccm1/Krit1 ECKO) and organism. Details provided include the protein, details of detection method, strain and organism, and a list of the corresponding literature the data was extracted from. For a list of the references and corresponding numbers used, please reference supplemental table 7. Genomic methods involved detection through RT-PCR or other DNA detection methods, proteomics methods involved pull-down assays and subsequent mass spectrometry applications while transcriptomics methods involved identification of genes through various RNA detection methods. Abbreviations: NVU: Neuro Vascular unit from surgically resected lesions.
