# Supplementary material for: Comparative omics of CCM signaling complex (CSC)
Source: Chin Neurosurg J. 2020 Jan 15;6:4. doi: 10.1186/s41016-019-0183-6 (PMC7398211; doi:10.1186/s41016-019-0183-6)
Supplement: Supplementary file 4 — Additional file 4: Table S3A. Detailed description of altered genes in CCM models with 2 validations. A total of 152 genes were analyzed that overlapped in two different CCM studies. Details provided for each protein include mechanisms associated with each, functions, binding partners, motifs and domains. Protein details were extracted from STRING enrichment data after construction of Figure 1 Interactome. [file 41016_2019_183_MOESM4_ESM.pdf]

Detailed description of altered genes in CCM models with 2 validations.

Query Item	Description
DAB2	Disabled homolog 2; Adapter protein that functions as clathrin-associated sorting protein (CLASP) required for clathrin-mediated endocytosis of selected cargo proteins. Can bind and assemble clathrin, and binds simultaneously to phosphatidylinositol 4,5-bisphosphate (PtdIns(4,5)P2) and cargos containing non-phosphorylated NPXY internalization motifs, such as the LDL receptor, to recruit them to clathrin-coated pits. Can function in clathrin-mediated endocytosis independently of the AP-2 complex. Involved in endocytosis of integrin beta-1.
DOK4	Docking protein 4; DOK proteins are enzymatically inert adaptor or scaffolding proteins. They provide a docking platform for the assembly of multimolecular signaling complexes. DOK4 functions in RET-mediated neurite outgrowth and plays a positive role in activation of the MAP kinase pathway (By similarity). Putative link with downstream effectors of RET in neuronal differentiation. May be involved in the regulation of the immune response induced by T-cells.
NUMB	Protein numb homolog; Plays a role in the process of neurogenesis. Required throughout embryonic neurogenesis to maintain neural progenitor cells, also called radial glial cells (RGCs), by allowing their daughter cells to choose progenitor over neuronal cell fate. Not required for the proliferation of neural progenitor cells before the onset of neurogenesis. Also involved postnatally in the subventricular zone (SVZ) neurogenesis by regulating SVZ neuroblasts survival and ependymal wall integrity. May also mediate local repair of brain ventricular wall damage.
PLEK	Pleckstrin; Major protein kinase C substrate of platelets; Pleckstrin homology domain containing.
RGS12	Regulator of G-protein signaling 12; Regulates G protein-coupled receptor signaling cascades. Inhibits signal transduction by increasing the GTPase activity of G protein alpha subunits, thereby driving them into their inactive GDP-bound form; PDZ domain containing.
TBC1D4	TBC1 domain family member 4; May act as a GTPase-activating protein for RAB2A, RAB8A, RAB10 and RAB14. Isoform 2 promotes insulin-induced glucose transporter SLC2A4/GLUT4 translocation at the plasma membrane, thus increasing glucose uptake.
TLN2	Talin-2; As a major component of focal adhesion plaques that links integrin to the actin cytoskeleton, may play an important role in cell adhesion. Recruits PIP5K1C to focal adhesion plaques and strongly activates its kinase activity (By similarity); FERM domain containing.
TNS2	Tensin-2; Regulates cell motility and proliferation. May have phosphatase activity. Reduces AKT1 phosphorylation. Lowers AKT1 kinase activity and interferes with AKT1 signaling.
SEPT7	Septin-7; Filament-forming cytoskeletal GTPase. Required for normal organization of the actin cytoskeleton. Required for normal progress through mitosis. Involved in cytokinesis. Required for normal association of CENPE with the kinetochore. Plays a role in ciliogenesis and collective cell movements. Forms a filamentous structure with SEPT12, SEPT6, SEPT2 and probably SEPT4 at the sperm annulus which is required for the structural integrity and motility of the sperm tail during postmeiotic differentiation.
ANXA1	Annexin A1; Plays important roles in the innate immune response as effector of glucocorticoid-mediated responses and regulator of the inflammatory process. Has anti-inflammatory activity. Plays a role in glucocorticoid-mediated down- regulation of the early phase of the inflammatory response (By similarity). Promotes resolution of inflammation and wound healing. Functions at least in part by activating the formyl peptide receptors and downstream signaling cascades. Promotes chemotaxis of granulocytes and monocytes via activation of the formyl peptide receptors.
ANXA2	Annexin A2; Calcium-regulated membrane-binding protein whose affinity for calcium is greatly enhanced by anionic phospholipids. It binds two calcium ions with high affinity. May be involved in heat-stress response. Inhibits PCSK9-enhanced LDLR degradation, probably reduces PCSK9 protein levels via a translational mechanism but also competes with LDLR for binding with PCSK9; Belongs to the annexin family.

ANXA5	Annexin A5; This protein is an anticoagulant protein that acts as an indirect inhibitor of the thromboplastin-specific complex, which is involved in the blood coagulation cascade.
ANXA6	Annexin A6; May associate with CD21. May regulate the release of Ca(2+) from intracellular stores.
ARF3	ADP-ribosylation factor 3; GTP-binding protein that functions as an allosteric activator of the cholera toxin catalytic subunit, an ADP- ribosyltransferase. Involved in protein trafficking; may modulate vesicle budding and uncoating within the Golgi apparatus; Belongs to the small GTPase superfamily.
ARF1	ADP-ribosylation factor 1; GTP-binding protein that functions as an allosteric activator of the cholera toxin catalytic subunit, an ADP- ribosyltransferase. Involved in protein trafficking among different compartments. Modulates vesicle budding and uncoating within the Golgi complex. Deactivation induces the redistribution of the entire Golgi complex to the endoplasmic reticulum, suggesting a crucial role in protein trafficking. In its GTP-bound form, its triggers the association with coat proteins with the Golgi membrane.
ARF4	ADP-ribosylation factor 4; GTP-binding protein that functions as an allosteric activator of the cholera toxin catalytic subunit, an ADP- ribosyltransferase. Involved in protein trafficking; may modulate vesicle budding and uncoating within the Golgi apparatus.
CALR	Calreticulin; Calcium-binding chaperone that promotes folding, oligomeric assembly and quality control in the endoplasmic reticulum (ER) via the calreticulin/calnexin cycle. This lectin interacts transiently with almost all of the monoglucosylated glycoproteins that are synthesized in the ER. Interacts with the DNA-binding domain of NR3C1 and mediates its nuclear export. Involved in maternal gene expression regulation. May participate in oocyte maturation via the regulation of calcium homeostasis (By similarity).
CAV1	Caveolin-1; May act as a scaffolding protein within caveolar membranes. Interacts directly with G-protein alpha subunits and can functionally regulate their activity (By similarity). Involved in the costimulatory signal essential for T-cell receptor (TCR)- mediated T-cell activation. Its binding to DPP4 induces T-cell proliferation and NF-kappa-B activation in a T-cell receptor/CD3-dependent manner. Recruits CTNNB1 to caveolar membranes and may regulate CTNNB1-mediated signaling through the Wnt pathway. Negatively regulates TGFB1-mediated activation of SMAD2/3.
CDC42	Cell division control protein 42 homolog; Plasma membrane-associated small GTPase which cycles between an active GTP-bound and an inactive GDP-bound state. In active state binds to a variety of effector proteins to regulate cellular responses. Involved in epithelial cell polarization processes. Regulates the bipolar attachment of spindle microtubules to kinetochores before chromosome congression in metaphase. Plays a role in the extension and maintenance of the formation of thin, actin-rich surface projections called filopodia. Mediates CDC42-dependent cell migration.
CLIC4	Chloride intracellular channel protein 4; Can insert into membranes and form poorly selective ion channels that may also transport chloride ions. Channel activity depends on the pH. Membrane insertion seems to be redox-regulated and may occur only under oxydizing conditions. Promotes cell- surface expression of HRH3. Has alternate cellular functions like a potential role in angiogenesis or in maintaining apical- basolateral membrane polarity during mitosis and cytokinesis. Could also promote endothelial cell proliferation and regulate endothelial morphogenesis (tubulogenesis).
CNN2	Calponin-2; Thin filament-associated protein that is implicated in the regulation and modulation of smooth muscle contraction. It is capable of binding to actin, calmodulin, troponin C and tropomyosin. The interaction of calponin with actin inhibits the actomyosin Mg-ATPase activity.

COPA	Coatomer subunit alpha; The coatomer is a cytosolic protein complex that binds to dilysine motifs and reversibly associates with Golgi non- clathrin-coated vesicles, which further mediate biosynthetic protein transport from the ER, via the Golgi up to the trans Golgi network. Coatomer complex is required for budding from Golgi membranes, and is essential for the retrograde Golgi-to-ER transport of dilysine-tagged proteins. In mammals, the coatomer can only be recruited by membranes associated to ADP-ribosylation factors (ARFs), which are small GTP-binding proteins.
EHD4	EH domain-containing protein 4; ATP- and membrane-binding protein that probably controls membrane reorganization/tubulation upon ATP hydrolysis. Plays a role in early endosomal transport; EF-hand domain containing.
FAS	Tumor necrosis factor receptor superfamily member 6; Receptor for TNFSF6/FASLG. The adapter molecule FADD recruits caspase-8 to the activated receptor. The resulting death-inducing signaling complex (DISC) performs caspase-8 proteolytic activation which initiates the subsequent cascade of caspases (aspartate-specific cysteine proteases) mediating apoptosis. FAS- mediated apoptosis may have a role in the induction of peripheral tolerance, in the antigen-stimulated suicide of mature T-cells, or both. The secreted isoforms 2 to 6 block apoptosis (in vitro).
FLNA	Filamin-A; Promotes orthogonal branching of actin filaments and links actin filaments to membrane glycoproteins. Anchors various transmembrane proteins to the actin cytoskeleton and serves as a scaffold for a wide range of cytoplasmic signaling proteins. Interaction with FLNA may allow neuroblast migration from the ventricular zone into the cortical plate. Tethers cell surface- localized furin, modulates its rate of internalization and directs its intracellular trafficking (By similarity). Involved in ciliogenesis. Plays a role in cell-cell contacts and adherens junctions
FLNB	Filamin-B; Connects cell membrane constituents to the actin cytoskeleton. May promote orthogonal branching of actin filaments and links actin filaments to membrane glycoproteins. Anchors various transmembrane proteins to the actin cytoskeleton. Interaction with FLNA may allow neuroblast migration from the ventricular zone into the cortical plate. Various interactions and localizations of isoforms affect myotube morphology and myogenesis. Isoform 6 accelerates muscle differentiation in vitro; Belongs to the filamin family.
FLNC	Filamin-C; Muscle-specific filamin, which plays a central role in muscle cells, probably by functioning as a large actin-cross- linking protein. May be involved in reorganizing the actin cytoskeleton in response to signaling events, and may also display structural functions at the Z lines in muscle cells. Critical for normal myogenesis and for maintaining the structural integrity of the muscle fibers.
FSCN1	Fascin; Organizes filamentous actin into bundles with a minimum of 4.1:1 actin/fascin ratio. Plays a role in the organization of actin filament bundles and the formation of microspikes, membrane ruffles, and stress fibers. Important for the formation of a diverse set of cell protrusions, such as filopodia, and for cell motility and migration.
GSTO1	Glutathione S-transferase omega-1; Exhibits glutathione-dependent thiol transferase and dehydroascorbate reductase activities. Has S-(phenacyl)glutathione reductase activity. Has also glutathione S-transferase activity. Participates in the biotransformation of inorganic arsenic and reduces monomethylarsonic acid (MMA) and dimethylarsonic acid.
HSPB1	Heat shock protein beta-1; Small heat shock protein which functions as a molecular chaperone probably maintaining denatured proteins in a folding- competent state. Plays a role in stress resistance and actin organization. Through its molecular chaperone activity may regulate numerous biological processes including the phosphorylation and the axonal transport of neurofilament proteins.
ICAM1	Intercellular adhesion molecule 1; ICAM proteins are ligands for the leukocyte adhesion protein LFA-1 (integrin alpha-L/beta-2). During leukocyte trans- endothelial migration, ICAM1 engagement promotes the assembly of endothelial apical cups through ARHGEF26/SGEF and RHOG activation.

LAMA4	Laminin subunit alpha-4; Binding to cells via a high affinity receptor, laminin is thought to mediate the attachment, migration and organization of cells into tissues during embryonic development by interacting with other extracellular matrix components.
LDHB	Lactate dehydrogenase B; Belongs to the LDH/MDH superfamily. LDH family.
MMRN2	Multimerin-2; Inhibits endothelial cells motility and acts as a negative regulator of angiogenesis; it downregulates KDR activation by binding VEGFA; EMI domain containing.
MT2	Metallothionein-2; Metallothioneins have a high content of cysteine residues that bind various heavy metals; these proteins are transcriptionally regulated by both heavy metals and glucocorticoids; Belongs to the metallothionein superfamily.
MYH9	Myosin-9; Cellular myosin that appears to play a role in cytokinesis, cell shape, and specialized functions such as secretion and capping. During cell spreading, plays an important role in cytoskeleton reorganization, focal contacts formation (in the margins but not the central part of spreading cells), and lamellipodial retraction; this function is mechanically antagonized by MYH10; Deafness associated genes.
MYO1C	Unconventional myosin-Ic; Myosins are actin-based motor molecules with ATPase activity. Unconventional myosins serve in intracellular movements. Their highly divergent tails are presumed to bind to membranous compartments, which would be moved relative to actin filaments. Involved in glucose transporter recycling in response to insulin by regulating movement of intracellular GLUT4-containing vesicles to the plasma membrane. Component of the hair cell's (the sensory cells of the inner ear) adaptation-motor complex. Acts as a mediator of adaptation of mechanoelectrical transduction.
PDIA6	Protein disulfide-isomerase A6; May function as a chaperone that inhibits aggregation of misfolded proteins. Negatively regulates the unfolded protein response (UPR) through binding to UPR sensors such as ERN1, which in turn inactivates ERN1 signaling. May also regulate the UPR via the EIF2AK3 UPR sensor. Plays a role in platelet aggregation and activation by agonists such as convulxin, collagen and thrombin.
PIR	Pirin; Transcriptional coregulator of NF-kappa-B which facilitates binding of NF-kappa-B proteins to target kappa-B genes in a redox-state-dependent manner. May be required for efficient terminal myeloid maturation of hematopoietic cells. Has quercetin 2,3-dioxygenase activity (in vitro); Belongs to the pirin family.
PLOD1	Procollagen-lysine,2-oxoglutarate 5-dioxygenase 1; Part of a complex composed of PLOD1, P3H3 and P3H4 that catalyzes hydroxylation of lysine residues in collagen alpha chains and is required for normal assembly and cross-linkling of collagen fibrils (By similarity). Forms hydroxylysine residues in -Xaa-Lys-Gly- sequences in collagens. These hydroxylysines serve as sites of attachment for carbohydrate units and are essential for the stability of the intermolecular collagen cross-links (Probable).
PLOD2	Procollagen-lysine,2-oxoglutarate 5-dioxygenase 2; Forms hydroxylysine residues in -Xaa-Lys-Gly- sequences in collagens. These hydroxylysines serve as sites of attachment for carbohydrate units and are essential for the stability of the intermolecular collagen cross-links.
PTMA	Prothymosin alpha; Prothymosin alpha may mediate immune function by conferring resistance to certain opportunistic infections.
PTRF	Caveolae-associated protein 1; Plays an important role in caveolae formation and organization. Essential for the formation of caveolae in all tissues. Core component of the CAVIN complex which is essential for recruitment of the complex to the caveolae in presence of calveolin-1 (CAV1). Essential for normal oligomerization of CAV1. Promotes ribosomal transcriptional activity in response to metabolic challenges in the adipocytes and plays an important role in the formation of the ribosomal transcriptional loop.
PXDN	Peroxidasin homolog; Displays low peroxidase activity and is likely to participate in H(2)O(2) metabolism and peroxidative reactions in the cardiovascular system. Plays a role in extracellular matrix formation; I-set domain containing.
RCN1	Reticulocalbin-1; May regulate calcium-dependent activities in the endoplasmic reticulum lumen or post-ER compartment; EF-hand domain containing.

SDPR	Caveolae-associated protein 2; Plays an important role in caveolar biogenesis and morphology. Regulates caveolae morphology by inducing membrane curvature within caveolae. Plays a role in caveola formation in a tissue-specific manner. Required for the formation of caveolae in the lung and fat endothelia but not in the heart endothelia. Negatively regulates the size or stability of CAVIN complexes in the lung endothelial cells. May play a role in targeting PRKCA to caveolae (By similarity).
SFPQ	Splicing factor, proline- and glutamine-rich; DNA- and RNA binding protein, involved in several nuclear processes. Essential pre-mRNA splicing factor required early in spliceosome formation and for splicing catalytic step II, probably as a heteromer with NONO. Binds to pre-mRNA in spliceosome C complex, and specifically binds to intronic polypyrimidine tracts. Involved in regulation of signal-induced alternative splicing. During splicing of PTPRC/CD45, a phosphorylated form is sequestered by THRAP3 from the pre-mRNA in resting T-cells.
TGM2	Protein-glutamine gamma-glutamyltransferase 2; Catalyzes the cross-linking of proteins and the conjugation of polyamines to proteins.
TPM2	Tropomyosin beta chain; Tropomyosin 2; Belongs to the tropomyosin family.
TPM3	Tropomyosin alpha-3 chain; Binds to actin filaments in muscle and non-muscle cells. Plays a central role, in association with the troponin complex, in the calcium dependent regulation of vertebrate striated muscle contraction. Smooth muscle contraction is regulated by interaction with caldesmon. In non-muscle cells is implicated in stabilizing cytoskeleton actin filaments; Belongs to the tropomyosin family.
VWF	Von Willebrand factor; Important in the maintenance of hemostasis, it promotes adhesion of platelets to the sites of vascular injury by forming a molecular bridge between sub-endothelial collagen matrix and platelet-surface receptor complex GPIb-IX-V. Also acts as a chaperone for coagulation factor VIII, delivering it to the site of injury, stabilizing its heterodimeric structure and protecting it from premature clearance from plasma.
XRCC5	X-ray repair cross-complementing protein 5; Single-stranded DNA-dependent ATP-dependent helicase. Has a role in chromosome translocation. The DNA helicase II complex binds preferentially to fork-like ends of double-stranded DNA in a cell cycle-dependent manner. It works in the 3'-5' direction. Binding to DNA may be mediated by XRCC6. Involved in DNA non-homologous end joining (NHEJ) required for double-strand break repair and V(D)J recombination. The XRCC5/6 dimer acts as regulatory subunit of the DNA-dependent protein kinase complex DNA-PK by increasing the affinity of the catalytic site.
ZYX	Zyxin; Adhesion plaque protein. Binds alpha-actinin and the CRP protein. Important for targeting TES and ENA/VASP family members to focal adhesions and for the formation of actin-rich structures. May be a component of a signal transduction pathway that mediates adhesion-stimulated changes in gene expression (By similarity).
ADD1	Alpha-adducin; Membrane-cytoskeleton-associated protein that promotes the assembly of the spectrin-actin network. Binds to calmodulin.
COL8A1	Collagen alpha-1(VIII) chain; Macromolecular component of the subendothelium. Major component of the Descemet's membrane (basement membrane) of corneal endothelial cells. Also component of the endothelia of blood vessels. Necessary for migration and proliferation of vascular smooth muscle cells and thus, has a potential role in the maintenance of vessel wall integrity and structure, in particular in atherogenesis.
FN1	Fibronectin type III domain containing; Endogenous ligands
ITGA2	Integrin alpha-2; Integrin alpha-2/beta-1 is a receptor for laminin, collagen, collagen C-propeptides, fibronectin and E-cadherin. It recognizes the proline-hydroxylated sequence G-F-P-G-E-R in collagen. It is responsible for adhesion of platelets and other cells to collagens, modulation of collagen and collagenase gene expression, force generation and organization of newly synthesized extracellular matrix.
LAMA5	Laminin subunit alpha-5; Binding to cells via a high affinity receptor, laminin is thought to mediate the attachment, migration and organization of cells into tissues during embryonic development by interacting with other extracellular matrix components.

MYO1B	Unconventional myosin-Ib; Motor protein that may participate in process critical to neuronal development and function such as cell migration, neurite outgrowth and vesicular transport.
SPTAN1	Spectrin alpha chain, non-erythrocytic 1; Fodrin, which seems to be involved in secretion, interacts with calmodulin in a calcium-dependent manner and is thus candidate for the calcium-dependent movement of the cytoskeleton at the membrane; EF-hand domain containing.
SPTBN2	Spectrin beta chain, non-erythrocytic 2; Probably plays an important role in neuronal membrane skeleton; Belongs to the spectrin family.
THBS1	Thrombospondin-1; Adhesive glycoprotein that mediates cell-to-cell and cell-to-matrix interactions. Binds heparin. May play a role in dentinogenesis and/or maintenance of dentin and dental pulp (By similarity). Ligand for CD36 mediating antiangiogenic properties. Plays a role in ER stress response, via its interaction with the activating transcription factor 6 alpha (ATF6) which produces adaptive ER stress response factors (By similarity).
CDH5	Cadherin-5; Cadherins are calcium-dependent cell adhesion proteins. They preferentially interact with themselves in a homophilic manner in connecting cells; cadherins may thus contribute to the sorting of heterogeneous cell types. This cadherin may play a important role in endothelial cell biology through control of the cohesion and organization of the intercellular junctions. It associates with alpha-catenin forming a link to the cytoskeleton. Acts in concert with KRIT1 to establish and maintain correct endothelial cell polarity and vascular lumen.
CFL1	Cofilin-1; Binds to F-actin and exhibits pH-sensitive F-actin depolymerizing activity. Regulates actin cytoskeleton dynamics. Important for normal progress through mitosis and normal cytokinesis. Plays a role in the regulation of cell morphology and cytoskeletal organization. Required for the up-regulation of atypical chemokine receptor ACKR2 from endosomal compartment to cell membrane, increasing its efficiency in chemokine uptake and degradation. Required for neural tube morphogenesis and neural crest cell migration (By similarity).
CNN2	Calponin-2; Thin filament-associated protein that is implicated in the regulation and modulation of smooth muscle contraction. It is capable of binding to actin, calmodulin, troponin C and tropomyosin. The interaction of calponin with actin inhibits the actomyosin Mg-ATPase activity.
FLNB	Filamin-B; Connects cell membrane constituents to the actin cytoskeleton. May promote orthogonal branching of actin filaments and links actin filaments to membrane glycoproteins. Anchors various transmembrane proteins to the actin cytoskeleton. Interaction with FLNA may allow neuroblast migration from the ventricular zone into the cortical plate. Various interactions and localizations of isoforms affect myotube morphology and myogenesis. Isoform 6 accelerates muscle differentiation in vitro; Belongs to the filamin family.
ABCB1	Multidrug resistance protein 1; Energy-dependent efflux pump responsible for decreased drug accumulation in multidrug-resistant cells; ATP binding cassette subfamily B.
CSF1R	Macrophage colony-stimulating factor 1 receptor; Tyrosine-protein kinase that acts as cell-surface receptor for CSF1 and IL34 and plays an essential role in the regulation of survival, proliferation and differentiation of hematopoietic precursor cells, especially mononuclear phagocytes, such as macrophages and monocytes. Promotes the release of proinflammatory chemokines in response to IL34 and CSF1, and thereby plays an important role in innate immunity and in inflammatory processes. Plays an important role in the regulation of osteoclast proliferation and differentiation.
CYP24A1	1,25-dihydroxyvitamin D(3) 24-hydroxylase, mitochondrial; Has a role in maintaining calcium homeostasis. Catalyzes the NADPH-dependent 24-hydroxylation of calcidiol (25-hydroxyvitamin D(3)) and calcitriol (1-alpha,25-dihydroxyvitamin D(3)). The enzyme can perform up to 6 rounds of hydroxylation of calcitriol leading to calcitroic acid. It also shows 23-hydroxylating activity leading to 1-alpha,25-dihydroxyvitamin D(3)-26,23-lactone as end product; Cytochrome P450 family 24.
CYP2R1	Vitamin D 25-hydroxylase; Has a D-25-hydroxylase activity on both forms of vitamin D, vitamin D(2) and D(3); Cytochrome P450 family 2.

DYRK1A	Dual specificity tyrosine-phosphorylation-regulated kinase 1A; Dual-specificity kinase which possesses both serine/threonine and tyrosine kinase activities. May play a role in a signaling pathway regulating nuclear functions of cell proliferation. Modulates alternative splicing by phosphorylating the splice factor SRSF6 (By similarity). Exhibits a substrate preference for proline at position P+1 and arginine at position P- 3. Has pro-survival function and negatively regulates the apoptotic process. Promotes cell survival upon genotoxic stress through phosphorylation of SIRT1.
FLT1	Vascular endothelial growth factor receptor 1; Tyrosine-protein kinase that acts as a cell-surface receptor for VEGFA, VEGFB and PGF, and plays an essential role in the development of embryonic vasculature, the regulation of angiogenesis, cell survival, cell migration, macrophage function, chemotaxis, and cancer cell invasion. May play an essential role as a negative regulator of embryonic angiogenesis by inhibiting excessive proliferation of endothelial cells. Can promote endothelial cell proliferation, survival and angiogenesis in adulthood.
KIT	Mast/stem cell growth factor receptor Kit; Tyrosine-protein kinase that acts as cell-surface receptor for the cytokine KITLG/SCF and plays an essential role in the regulation of cell survival and proliferation, hematopoiesis, stem cell maintenance, gametogenesis, mast cell development, migration and function, and in melanogenesis. In response to KITLG/SCF binding, KIT can activate several signaling pathways. Phosphorylates PIK3R1, PLCG1, SH2B2/APS and CBL. Activates the AKT1 signaling pathway by phosphorylation of PIK3R1.
LCK	Tyrosine-protein kinase Lck; Non-receptor tyrosine-protein kinase that plays an essential role in the selection and maturation of developing T- cells in the thymus and in the function of mature T-cells. Plays a key role in T-cell antigen receptor (TCR)-linked signal transduction pathways. Constitutively associated with the cytoplasmic portions of the CD4 and CD8 surface receptors. Association of the TCR with a peptide antigen-bound MHC complex facilitates the interaction of CD4 and CD8 with MHC class II and class I molecules, respectively.
NR1I2	Nuclear receptor subfamily 1 group I member 2; Nuclear receptor that binds and is activated by variety of endogenous and xenobiotic compounds. Transcription factor that activates the transcription of multiple genes involved in the metabolism and secretion of potentially harmful xenobiotics, drugs and endogenous compounds. Activated by the antibiotic rifampicin and various plant metabolites, such as hyperforin, guggulipid, colupulone, and isoflavones. Response to specific ligands is species-specific. Activated by naturally occurring steroids, such as pregnenolone and progesterone.
PDGFRA	Platelet-derived growth factor receptor alpha; Tyrosine-protein kinase that acts as a cell-surface receptor for PDGFA, PDGFB and PDGFC and plays an essential role in the regulation of embryonic development, cell proliferation, survival and chemotaxis. Depending on the context, promotes or inhibits cell proliferation and cell migration. Plays an important role in the differentiation of bone marrow-derived mesenchymal stem cells. Required for normal skeleton development and cephalic closure during embryonic development.
PLK1	Serine/threonine-protein kinase PLK1; Serine/threonine-protein kinase that performs several important functions throughout M phase of the cell cycle, including the regulation of centrosome maturation and spindle assembly, the removal of cohesins from chromosome arms, the inactivation of anaphase-promoting complex/cyclosome (APC/C) inhibitors, and the regulation of mitotic exit and cytokinesis. Polo-like kinase proteins acts by binding and phosphorylating proteins are that already phosphorylated on a specific motif recognized by the POLO box domains.
PPP3CA	Serine/threonine-protein phosphatase 2B catalytic subunit alpha isoform; Calcium-dependent, calmodulin-stimulated protein phosphatase. Many of the substrates contain a PxIxIT motif. This subunit may have a role in the calmodulin activation of calcineurin. Dephosphorylates DNM1L, HSPB1 and SSH1; Belongs to the PPP phosphatase family. PP-2B subfamily.

RARB	Retinoic acid receptor beta; Receptor for retinoic acid. Retinoic acid receptors bind as heterodimers to their target response elements in response to their ligands, all-trans or 9-cis retinoic acid, and regulate gene expression in various biological processes. The RXR/RAR heterodimers bind to the retinoic acid response elements (RARE) composed of tandem 5'-AGGTCA-3' sites known as DR1-DR5. In the absence or presence of hormone ligand, acts mainly as an activator of gene expression due to weak binding to corepressors. In concert with RARG, required for skeletal growth, matrix homeostasis.
RARG	Retinoic acid receptor gamma; Receptor for retinoic acid. Retinoic acid receptors bind as heterodimers to their target response elements in response to their ligands, all-trans or 9-cis retinoic acid, and regulate gene expression in various biological processes. The RAR/RXR heterodimers bind to the retinoic acid response elements (RARE) composed of tandem 5'-AGGTCA-3' sites known as DR1-DR5. In the absence of ligand, acts mainly as an activator of gene expression due to weak binding to corepressors. Required for limb bud development. In concert with RARA or RARB.
RXRB	Retinoic acid receptor RXR-beta; Receptor for retinoic acid. Retinoic acid receptors bind as heterodimers to their target response elements in response to their ligands, all-trans or 9-cis retinoic acid, and regulate gene expression in various biological processes. The RAR/RXR heterodimers bind to the retinoic acid response elements (RARE); Nuclear hormone receptors.
RXRG	Retinoic acid receptor RXR-gamma; Receptor for retinoic acid. Retinoic acid receptors bind as heterodimers to their target response elements in response to their ligands, all-trans or 9-cis retinoic acid, and regulate gene expression in various biological processes. The RAR/RXR heterodimers bind to the retinoic acid response elements (RARE) composed of tandem 5'-AGGTCA-3' sites known as DR1-DR5. The high affinity ligand for RXRs is 9-cis retinoic acid (By similarity); Nuclear hormone receptors.
SNCA	Alpha-synuclein; May be involved in the regulation of dopamine release and transport. Induces fibrillization of microtubule-associated protein tau. Reduces neuronal responsiveness to various apoptotic stimuli, leading to a decreased caspase-3 activation; Parkinson disease associated genes.
USP1	Ubiquitin carboxyl-terminal hydrolase 1; Negative regulator of DNA damage repair which specifically deubiquitinates monoubiquitinated FANCD2. Also involved in PCNA-mediated translesion synthesis (TLS) by deubiquitinating monoubiquitinated PCNA. Has almost no deubiquitinating activity by itself and requires the interaction with WDR48 to have a high activity.
WEE1	Wee1-like protein kinase; Acts as a negative regulator of entry into mitosis (G2 to M transition) by protecting the nucleus from cytoplasmically activated cyclin B1-complexed CDK1 before the onset of mitosis by mediating phosphorylation of CDK1 on 'Tyr-15'. Specifically phosphorylates and inactivates cyclin B1-complexed CDK1 reaching a maximum during G2 phase and a minimum as cells enter M phase. Phosphorylation of cyclin B1-CDK1 occurs exclusively on 'Tyr-15' and phosphorylation of monomeric CDK1 does not occur. Its activity increases during S and G2 phases and decreases at M phase
TUBA1A	Tubulin alpha-1A chain; Tubulin is the major constituent of microtubules. It binds two moles of GTP, one at an exchangeable site on the beta chain and one at a non-exchangeable site on the alpha chain; Tubulins.
TUBB4A	Tubulin beta-4A chain; Tubulin is the major constituent of microtubules. It binds two moles of GTP, one at an exchangeable site on the beta chain and one at a non-exchangeable site on the alpha chain; Tubulins.

AKT3	RAC-gamma serine/threonine-protein kinase; AKT3 is one of 3 closely related serine/threonine-protein kinases (AKT1, AKT2 and AKT3) called the AKT kinase, and which regulate many processes including metabolism, proliferation, cell survival, growth and angiogenesis. This is mediated through serine and/or threonine phosphorylation of a range of downstream substrates. Over 100 substrate candidates have been reported so far, but for most of them, no isoform specificity has been reported. AKT3 is the least studied AKT isoform. It plays an important role in brain development.
APP	Amyloid-beta A4 protein; N-APP binds TNFRSF21 triggering caspase activation and degeneration of both neuronal cell bodies (via caspase-3) and axons (via caspase-6); Endogenous ligands.
BCL2	Apoptosis regulator Bcl-2; Suppresses apoptosis in a variety of cell systems including factor-dependent lymphohematopoietic and neural cells. Regulates cell death by controlling the mitochondrial membrane permeability. Appears to function in a feedback loop system with caspases. Inhibits caspase activity either by preventing the release of cytochrome c from the mitochondria and/or by binding to the apoptosis-activating factor (APAF-1). May attenuate inflammation by impairing NLRP1-inflammasome activation, hence CASP1 activation and IL1B release; BCL2 family.
BCL2L1	Bcl-2-like protein 1; Potent inhibitor of cell death. Inhibits activation of caspases. Appears to regulate cell death by blocking the voltage- dependent anion channel (VDAC) by binding to it and preventing the release of the caspase activator, CYC1, from the mitochondrial membrane. Also acts as a regulator of G2 checkpoint and progression to cytokinesis during mitosis; Belongs to the Bcl-2 family.
BCL2L2	Bcl-2-like protein 2; Promotes cell survival. Blocks dexamethasone-induced apoptosis. Mediates survival of postmitotic Sertoli cells by suppressing death-promoting activity of BAX; Belongs to the Bcl-2 family.
BRSK1	Serine/threonine-protein kinase BRSK1; Serine/threonine-protein kinase that plays a key role in polarization of neurons and centrosome duplication. Phosphorylates CDC25B, CDC25C, MAPT/TAU, RIMS1, TUBG1, TUBG2 and WEE1. Following phosphorylation and activation by STK11/LKB1, acts as a key regulator of polarization of cortical neurons, probably by mediating phosphorylation of microtubule-associated proteins such as MAPT/TAU at 'Thr-529' and 'Ser-579'. Also regulates neuron polarization by mediating phosphorylation of WEE1 at 'Ser-642' in post-mitotic neurons.
CAMK1D	Calcium/calmodulin-dependent protein kinase type 1D; Calcium/calmodulin-dependent protein kinase that operates in the calcium-triggered CaMKK-CaMK1 signaling cascade and, upon calcium influx, activates CREB-dependent gene transcription, regulates calcium-mediated granulocyte function and respiratory burst and promotes basal dendritic growth of hippocampal neurons. In neutrophil cells, required for cytokine- induced proliferative responses and activation of the respiratory burst. Activates the transcription factor CREB1 in hippocampal neuron nuclei.
CAMK2A	Calcium/calmodulin-dependent protein kinase type II subunit alpha; CaM-kinase II (CAMK2) is a prominent kinase in the central nervous system that may function in long-term potentiation and neurotransmitter release. Member of the NMDAR signaling complex in excitatory synapses it may regulate NMDAR-dependent potentiation of the AMPAR and synaptic plasticity (By similarity). Phosphorylates transcription factor FOXO3 on 'Ser-298'. Activates FOXO3 transcriptional activity (By similarity); Belongs to the protein kinase superfamily. CAMK Ser/Thr protein kinase family. CaMK subfamily.
CAMK2B	Calcium/calmodulin-dependent protein kinase type II subunit beta; Calcium/calmodulin-dependent protein kinase that functions autonomously after Ca(2+)/calmodulin-binding and autophosphorylation, and is involved in dendritic spine and synapse formation, neuronal plasticity and regulation of sarcoplasmic reticulum Ca(2+) transport in skeletal muscle. In neurons, plays an essential structural role in the reorganization of the actin cytoskeleton during plasticity by binding and bundling actin filaments in a kinase-independent manner.

CCR5	C-C chemokine receptor type 5; Receptor for a number of inflammatory CC-chemokines including MIP-1-alpha, MIP-1-beta and RANTES and subsequently transduces a signal by increasing the intracellular calcium ion level. May play a role in the control of granulocytic lineage proliferation or differentiation. Acts as a coreceptor (CD4 being the primary receptor) for HIV-1 R5 isolates.
CDC25A	M-phase inducer phosphatase 1; Tyrosine protein phosphatase which functions as a dosage-dependent inducer of mitotic progression. Directly dephosphorylates CDK1 and stimulates its kinase activity. Also dephosphorylates CDK2 in complex with cyclin E, in vitro; Class III Cys-based CDC25 phosphatases.
CDC25C	M-phase inducer phosphatase 3; Functions as a dosage-dependent inducer in mitotic control. Tyrosine protein phosphatase required for progression of the cell cycle. When phosphorylated, highly effective in activating G2 cells into prophase. Directly dephosphorylates CDK1 and activates its kinase activity.
CYP24A1	1,25-dihydroxyvitamin D(3) 24-hydroxylase, mitochondrial; Has a role in maintaining calcium homeostasis. Catalyzes the NADPH-dependent 24-hydroxylation of calcidiol (25-hydroxyvitamin D(3)) and calcitriol (1-alpha,25-dihydroxyvitamin D(3)). The enzyme can perform up to 6 rounds of hydroxylation of calcitriol leading to calcitroic acid. It also shows 23-hydroxylating activity leading to 1-alpha,25-dihydroxyvitamin D(3)-26,23-lactone as end product; Cytochrome P450 family 24.
CYP2R1	Vitamin D 25-hydroxylase; Has a D-25-hydroxylase activity on both forms of vitamin D, vitamin D(2) and D(3); Cytochrome P450 family 2.
DNM1	Dynamin-1; Microtubule-associated force-producing protein involved in producing microtubule bundles and able to bind and hydrolyze GTP. Most probably involved in vesicular trafficking processes. Involved in receptor-mediated endocytosis; Pleckstrin homology domain containing.
DPP4	Dipeptidyl peptidase 4; Cell surface glycoprotein receptor involved in the costimulatory signal essential for T-cell receptor (TCR)-mediated T-cell activation. Acts as a positive regulator of T-cell coactivation, by binding at least ADA, CAV1, IGF2R, and PTPRC. Its binding to CAV1 and CARD11 induces T-cell proliferation and NF- kappa-B activation in a T-cell receptor/CD3-dependent manner. Its interaction with ADA also regulates lymphocyte-epithelial cell adhesion. In association with FAP is involved in the pericellular proteolysis of the extracellular matrix (ECM).
DYRK1A	Dual specificity tyrosine-phosphorylation-regulated kinase 1A; Dual-specificity kinase which possesses both serine/threonine and tyrosine kinase activities. May play a role in a signaling pathway regulating nuclear functions of cell proliferation. Modulates alternative splicing by phosphorylating the splice factor SRSF6 (By similarity). Exhibits a substrate preference for proline at position P+1 and arginine at position P- 3. Has pro-survival function and negatively regulates the apoptotic process. Promotes cell survival upon genotoxic stress through phosphorylation of SIRT1.
EPHA2	Ephrin type-A receptor 2; Receptor tyrosine kinase which binds promiscuously membrane-bound ephrin-A family ligands residing on adjacent cells, leading to contact-dependent bidirectional signaling into neighboring cells. The signaling pathway downstream of the receptor is referred to as forward signaling while the signaling pathway downstream of the ephrin ligand is referred to as reverse signaling. Activated by the ligand ephrin-A1/EFNA1 regulates migration, integrin-mediated adhesion, proliferation and differentiation of cells. Regulates cell adhesion and differentiation.
FYN	Tyrosine-protein kinase Fyn; Non-receptor tyrosine-protein kinase that plays a role in many biological processes including regulation of cell growth and survival, cell adhesion, integrin-mediated signaling, cytoskeletal remodeling, cell motility, immune response and axon guidance. Inactive FYN is phosphorylated on its C-terminal tail within the catalytic domain. Following activation by PKA, the protein subsequently associates with PTK2/FAK1, allowing PTK2/FAK1 phosphorylation, activation and targeting to focal adhesions. Involved in the regulation of cell adhesion and motility.

G6PD	Glucose-6-phosphate 1-dehydrogenase; Catalyzes the rate-limiting step of the oxidative pentose-phosphate pathway, which represents a route for the dissimilation of carbohydrates besides glycolysis. The main function of this enzyme is to provide reducing power (NADPH) and pentose phosphates for fatty acid and nucleic acid synthesis; Belongs to the glucose-6-phosphate dehydrogenase family.
LTB4R	Leukotriene B4 receptor 1; Receptor for extracellular ATP > UTP and ADP. The activity of this receptor is mediated by G proteins which activate a phosphatidylinositol-calcium second messenger system. May be the cardiac P2Y receptor involved in the regulation of cardiac muscle contraction through modulation of L-type calcium currents. Is a receptor for leukotriene B4, a potent chemoattractant involved in inflammation and immune response; Belongs to the G-protein coupled receptor 1 family.
MAP2K5	Dual specificity mitogen-activated protein kinase kinase 5; Acts as a scaffold for the formation of a ternary MAP3K2/MAP3K3-MAP3K5-MAPK7 signaling complex. Activation of this pathway appears to play a critical role in protecting cells from stress-induced apoptosis, neuronal survival and cardiac development and angiogenesis; Belongs to the protein kinase superfamily. STE Ser/Thr protein kinase family. MAP kinase kinase subfamily.
MAP4K4	Mitogen-activated protein kinase kinase kinase kinase 4; Serine/threonine kinase that may play a role in the response to environmental stress and cytokines such as TNF-alpha. Appears to act upstream of the JUN N-terminal pathway. Phosphorylates SMAD1 on Thr-322; Belongs to the protein kinase superfamily. STE Ser/Thr protein kinase family. STE20 subfamily.
MELK	Maternal embryonic leucine zipper kinase; Serine/threonine-protein kinase involved in various processes such as cell cycle regulation, self-renewal of stem cells, apoptosis and splicing regulation. Has a broad substrate specificity; phosphorylates BCL2L14, CDC25B, MAP3K5/ASK1 and ZNF622. Acts as an activator of apoptosis by phosphorylating and activating MAP3K5/ASK1. Acts as a regulator of cell cycle, notably by mediating phosphorylation of CDC25B, promoting localization of CDC25B to the centrosome and the spindle poles during mitosis. Plays a key role in cell proliferation
MMP14	Matrix metalloproteinase-14; Endopeptidase that degrades various components of the extracellular matrix such as collagen. Activates progelatinase A. Essential for pericellular collagenolysis and modeling of skeletal and extraskeletal connective tissues during development (By similarity). May be involved in actin cytoskeleton reorganization by cleaving PTK7. Acts as a positive regulator of cell growth and migration via activation of MMP15. Involved in the formation of the fibrovascular tissues in association with pro-MMP2. Cleaves ADGRB1 to release vasculostatin-40.
MMP2	72 kDa type IV collagenase; Ubiquitinous metalloproteinase that is involved in diverse functions such as remodeling of the vasculature, angiogenesis, tissue repair, tumor invasion, inflammation, and atherosclerotic plaque rupture. As well as degrading extracellular matrix proteins, can also act on several nonmatrix proteins such as big endothelial 1 and beta-type CGRP promoting vasoconstriction. Also cleaves KISS at a Gly-|-Leu bond. Appears to have a role in myocardial cell death pathways. Contributes to myocardial oxidative stress by regulating the activity of GSK3beta.
NPC1L1	Niemann-Pick C1-like protein 1; Plays a major role in cholesterol homeostasis. Is critical for the uptake of cholesterol across the plasma membrane of the intestinal enterocyte. Is the direct molecular target of ezetimibe, a drug that inhibits cholesterol absorption. Lack of activity leads to multiple lipid transport defects. The protein may have a function in the transport of multiple lipids and their homeostasis, and may play a critical role in regulating lipid metabolism. Acts as a negative regulator of NPC2 and down- regulates its expression and secretion.
NQO1	NAD(P)H dehydrogenase [quinone] 1; The enzyme apparently serves as a quinone reductase in connection with conjugation reactions of hydroquinons involved in detoxification pathways as well as in biosynthetic processes such as the vitamin K-dependent gamma-carboxylation of glutamate residues in prothrombin synthesis; Belongs to the NAD(P)H dehydrogenase (quinone) family.

NR1H4	Bile acid receptor; Isoform 4: Promotes transcriptional activation of target genes ABCB11/BSEP (inducible by unconjugated CDCA, ACA and DCA), NR0B2/SHP (inducible by unconjugated CDCA, ACA and DCA), SLC51B/OSTB (inducible by unconjugated CDCA and DCA) and FABP6/IBAP; most efficient isoform compared to isoforms 1 to 3; not inducible by taurine- and glycine-amidated CDCA; Belongs to the nuclear hormone receptor family. NR1 subfamily.
NR1I2	Nuclear receptor subfamily 1 group I member 2; Nuclear receptor that binds and is activated by variety of endogenous and xenobiotic compounds. Transcription factor that activates the transcription of multiple genes involved in the metabolism and secretion of potentially harmful xenobiotics, drugs and endogenous compounds. Activated by the antibiotic rifampicin and various plant metabolites, such as hyperforin, guggulipid, colupulone, and isoflavones. Response to specific ligands is species-specific. Activated by naturally occurring steroids, such as pregnenolone and progesterone.
PBK	Lymphokine-activated killer T-cell-originated protein kinase; Phosphorylates MAP kinase p38. Seems to be active only in mitosis. May also play a role in the activation of lymphoid cells. When phosphorylated, forms a complex with TP53, leading to TP53 destabilization and attenuation of G2/M checkpoint during doxorubicin-induced DNA damage; Belongs to the protein kinase superfamily. STE Ser/Thr protein kinase family. MAP kinase kinase subfamily.
PPP3R1	Calcineurin subunit B type 1; Regulatory subunit of calcineurin, a calcium-dependent, calmodulin stimulated protein phosphatase. Confers calcium sensitivity; EF-hand domain containing.
PRKCG	Protein kinase C gamma type; Calcium-activated, phospholipid- and diacylglycerol (DAG)-dependent serine/threonine-protein kinase that plays diverse roles in neuronal cells and eye tissues, such as regulation of the neuronal receptors GRIA4/GLUR4 and GRIN1/NMDAR1, modulation of receptors and neuronal functions related to sensitivity to opiates, pain and alcohol, mediation of synaptic function and cell survival after ischemia, and inhibition of gap junction activity after oxidative stress. Binds and phosphorylates GRIA4/GLUR4 glutamate receptor and regulates its function.
PRKCH	Protein kinase C eta type; Calcium-independent, phospholipid- and diacylglycerol (DAG)-dependent serine/threonine-protein kinase that is involved in the regulation of cell differentiation in keratinocytes and pre-B cell receptor, mediates regulation of epithelial tight junction integrity and foam cell formation, and is required for glioblastoma proliferation and apoptosis prevention in MCF-7 cells. In keratinocytes, binds and activates the tyrosine kinase FYN, which in turn blocks epidermal growth factor receptor (EGFR) signaling and leads to keratinocyte growth arrest
PSIP1	PC4 and SFRS1-interacting protein; Transcriptional coactivator involved in neuroepithelial stem cell differentiation and neurogenesis. Involved in particular in lens epithelial cell gene regulation and stress responses. May play an important role in lens epithelial to fiber cell terminal differentiation. May play a protective role during stress-induced apoptosis. Isoform 2 is a more general and stronger transcriptional coactivator. Isoform 2 may also act as an adapter to coordinate pre-mRNA splicing. Cellular cofactor for lentiviral integration; Heparin binding growth factor family.
PTGES	Prostaglandin E synthase; Catalyzes the oxidoreduction of prostaglandin endoperoxide H2 (PGH2) to prostaglandin E2 (PGE2); Belongs to the MAPEG family.
PTPN1	Tyrosine-protein phosphatase non-receptor type 1; Tyrosine-protein phosphatase which acts as a regulator of endoplasmic reticulum unfolded protein response. Mediates dephosphorylation of EIF2AK3/PERK; inactivating the protein kinase activity of EIF2AK3/PERK. May play an important role in CKII- and p60c-src-induced signal transduction cascades. May regulate the EFNA5-EPHA3 signaling pathway which modulates cell reorganization and cell-cell repulsion. May also regulate the hepatocyte growth factor receptor signaling pathway through dephosphorylation of MET.

RARB	Retinoic acid receptor beta; Receptor for retinoic acid. Retinoic acid receptors bind as heterodimers to their target response elements in response to their ligands, all-trans or 9-cis retinoic acid, and regulate gene expression in various biological processes. The RXR/RAR heterodimers bind to the retinoic acid response elements (RARE) composed of tandem 5'-AGGTCA-3' sites known as DR1-DR5. In the absence or presence of hormone ligand, acts mainly as an activator of gene expression due to weak binding to corepressors. In concert with RARG, required for skeletal growth, matrix homeostasis
RARG	Retinoic acid receptor gamma; Receptor for retinoic acid. Retinoic acid receptors bind as heterodimers to their target response elements in response to their ligands, all-trans or 9-cis retinoic acid, and regulate gene expression in various biological processes. The RAR/RXR heterodimers bind to the retinoic acid response elements (RARE) composed of tandem 5'-AGGTCA-3' sites known as DR1-DR5. In the absence of ligand, acts mainly as an activator of gene expression due to weak binding to corepressors. Required for limb bud development. In concert with RARA or RARB.
RBP1	Retinol-binding protein 1; Cytoplasmic retinol-binding protein. Accepts retinol from the transport protein STRA6, and thereby contributes to retinol uptake, storage and retinoid homeostasis; Belongs to the calycin superfamily. Fatty-acid binding protein (FABP) family.
RORA	Nuclear receptor ROR-alpha; Nuclear receptor that binds DNA as a monomer to ROR response elements (RORE) containing a single core motif half-site 5'-AGGTCA-3' preceded by a short A-T-rich sequence. Key regulator of embryonic development, cellular differentiation, immunity, circadian rhythm as well as lipid, steroid, xenobiotics and glucose metabolism. Considered to have intrinsic transcriptional activity, have some natural ligands like oxysterols that act as agonists (25-hydroxycholesterol) or inverse agonists (7-oxygenated sterols).
RORC	Nuclear receptor ROR-gamma; Nuclear receptor that binds DNA as a monomer to ROR response elements (RORE) containing a single core motif half-site 5'-AGGTCA-3' preceded by a short A-T-rich sequence. Key regulator of cellular differentiation, immunity, peripheral circadian rhythm as well as lipid, steroid, xenobiotics and glucose metabolism. Considered to have intrinsic transcriptional activity, have some natural ligands like oxysterols that act as agonists (25-hydroxycholesterol) or inverse agonists (7-oxygenated sterols), enhancing or repressing the transcriptional activity, respectively.
RXRB	Retinoic acid receptor RXR-beta; Receptor for retinoic acid. Retinoic acid receptors bind as heterodimers to their target response elements in response to their ligands, all-trans or 9-cis retinoic acid, and regulate gene expression in various biological processes. The RAR/RXR heterodimers bind to the retinoic acid response elements (RARE); Nuclear hormone receptors.
RXRG	Retinoic acid receptor RXR-gamma; Receptor for retinoic acid. Retinoic acid receptors bind as heterodimers to their target response elements in response to their ligands, all-trans or 9-cis retinoic acid, and regulate gene expression in various biological processes. The RAR/RXR heterodimers bind to the retinoic acid response elements (RARE) composed of tandem 5'-AGGTCA-3' sites known as DR1-DR5. The high affinity ligand for RXRs is 9-cis retinoic acid (By similarity); Nuclear hormone receptors.
SLK	STE20-like serine/threonine-protein kinase; Mediates apoptosis and actin stress fiber dissolution; Belongs to the protein kinase superfamily. STE Ser/Thr protein kinase family. STE20 subfamily.
TLR4	Toll-like receptor 4; Cooperates with LY96 and CD14 to mediate the innate immune response to bacterial lipopolysaccharide (LPS). Acts via MYD88, TIRAP and TRAF6, leading to NF-kappa-B activation, cytokine secretion and the inflammatory response. Also involved in LPS-independent inflammatory responses triggered by free fatty acids, such as palmitate, and Ni(2+). Responses triggered by Ni(2+) require non-conserved histidines and are, therefore, species-specific. Both M.tuberculosis HSP70 (dnaK) and HSP65 (groEL-2) act via this protein to stimulate NF-kappa-B expression.

TOP2B	DNA topoisomerase 2-beta; Control of topological states of DNA by transient breakage and subsequent rejoining of DNA strands. Topoisomerase II makes double-strand breaks; Belongs to the type II topoisomerase family.
TUBA1C	Tubulin alpha-1C chain; Tubulin is the major constituent of microtubules. It binds two moles of GTP, one at an exchangeable site on the beta chain and one at a non-exchangeable site on the alpha chain; Belongs to the tubulin family.
TUBB4A	Tubulin beta-4A chain; Tubulin is the major constituent of microtubules. It binds two moles of GTP, one at an exchangeable site on the beta chain and one at a non-exchangeable site on the alpha chain.
MYH10	Myosin-10; Cellular myosin that appears to play a role in cytokinesis, cell shape, and specialized functions such as secretion and capping. Involved with LARP6 in the stabilization of type I collagen mRNAs for CO1A1 and CO1A2. During cell spreading, plays an important role in cytoskeleton reorganization, focal contacts formation (in the central part but not the margins of spreading cells), and lamellipodial extension; this function is mechanically antagonized by MYH9; Belongs to the TRAFAC class myosin-kinesin ATPase superfamily. Myosin family.
TUBB4A	Tubulin beta-4A chain; Tubulin is the major constituent of microtubules. It binds two moles of GTP, one at an exchangeable site on the beta chain and one at a non-exchangeable site on the alpha chain.
CFL1	Cofilin-1; Binds to F-actin and exhibits pH-sensitive F-actin depolymerizing activity. Regulates actin cytoskeleton dynamics. Important for normal progress through mitosis and normal cytokinesis. Plays a role in the regulation of cell morphology and cytoskeletal organization. Required for the up-regulation of atypical chemokine receptor ACKR2 from endosomal compartment to cell membrane, increasing its efficiency in chemokine uptake and degradation. Required for neural tube morphogenesis and neural crest cell migration (By similarity).
TUBA1C	Tubulin alpha-1C chain; Tubulin is the major constituent of microtubules. It binds two moles of GTP, one at an exchangeable site on the beta chain and one at a non-exchangeable site on the alpha chain; Belongs to the tubulin family.
TUBA1A	Tubulin alpha-1A chain; Tubulin is the major constituent of microtubules. It binds two moles of GTP, one at an exchangeable site on the beta chain and one at a non-exchangeable site on the alpha chain.
DNAJB6	DnaJ homolog subfamily B member 6; Plays an indispensable role in the organization of KRT8/KRT18 filaments. Acts as an endogenous molecular chaperone for neuronal proteins including huntingtin. Suppresses aggregation and toxicity of polyglutamine-containing, aggregation-prone proteins. Isoform B but not isoform A inhibits huntingtin aggregation. Has a stimulatory effect on the ATPase activity of HSP70 in a dose-dependent and time-dependent manner and hence acts as a co-chaperone of HSP70. Also reduces cellular toxicity and caspase-3 activity.
CCT2	T-complex protein 1 subunit beta; Molecular chaperone; assists the folding of proteins upon ATP hydrolysis. As part of the BBS/CCT complex may play a role in the assembly of BBSome, a complex involved in ciliogenesis regulating transports vesicles to the cilia. Known to play a role, in vitro, in the folding of actin and tubulin.
GNB4	Guanine nucleotide-binding protein subunit beta-4; Guanine nucleotide-binding proteins (G proteins) are involved as a modulator or transducer in various transmembrane signaling systems. The beta and gamma chains are required for the GTPase activity, for replacement of GDP by GTP, and for G protein- effector interaction.
COPA	Coatomer subunit alpha; The coatomer is a cytosolic protein complex that binds to dilysine motifs and reversibly associates with Golgi non- clathrin-coated vesicles, which further mediate biosynthetic protein transport from the ER, via the Golgi up to the trans Golgi network. Coatomer complex is required for budding from Golgi membranes, and is essential for the retrograde Golgi-to-ER transport of dilysine-tagged proteins. In mammals, the coatomer can only be recruited by membranes associated to ADP-ribosylation factors (ARFs), which are small GTP-binding proteins.

ARF1	ADP-ribosylation factor 1; GTP-binding protein that functions as an allosteric activator of the cholera toxin catalytic subunit, an ADP- ribosyltransferase. Involved in protein trafficking among different compartments. Modulates vesicle budding and uncoating within the Golgi complex. Deactivation induces the redistribution of the entire Golgi complex to the endoplasmic reticulum, suggesting a crucial role in protein trafficking. In its GTP-bound form, its triggers the association with coat proteins with the Golgi membrane.
ARF2	ADP-ribosylation factor 4; GTP-binding protein that functions as an allosteric activator of the cholera toxin catalytic subunit, an ADP- ribosyltransferase. Involved in protein trafficking; may modulate vesicle budding and uncoating within the Golgi apparatus; ARF GTPase family.
ARF3	ADP-ribosylation factor 3; GTP-binding protein that functions as an allosteric activator of the cholera toxin catalytic subunit, an ADP- ribosyltransferase. Involved in protein trafficking; may modulate vesicle budding and uncoating within the Golgi apparatus; Belongs to the small GTPase superfamily. Arf family.
ARF4	ADP-ribosylation factor 4; GTP-binding protein that functions as an allosteric activator of the cholera toxin catalytic subunit, an ADP- ribosyltransferase. Involved in protein trafficking; may modulate vesicle budding and uncoating within the Golgi apparatus; ARF GTPase family.
ENO1	Alpha-enolase; Multifunctional enzyme that, as well as its role in glycolysis, plays a part in various processes such as growth control, hypoxia tolerance and allergic responses. May also function in the intravascular and pericellular fibrinolytic system due to its ability to serve as a receptor and activator of plasminogen on the cell surface of several cell-types such as leukocytes and neurons. Stimulates immunoglobulin production; Belongs to the enolase family.
CAD	CAD protein; This protein is a "fusion" protein encoding four enzymatic activities of the pyrimidine pathway (GATase, CPSase, ATCase and DHOase); In the central section; belongs to the metallo- dependent hydrolases superfamily. DHOase family. CAD subfamily.
PAICS	Multifunctional protein ADE2; Phosphoribosylaminoimidazole carboxylase and phosphoribosylaminoimidazolesuccinocarboxamide synthase; In the C-terminal section; belongs to the AIR carboxylase family. Class II subfamily.
Ckb	Creatine kinase B-type; Reversibly catalyzes the transfer of phosphate between ATP and various phosphogens (e.g. creatine phosphate). Creatine kinase isoenzymes play a central role in energy transduction in tissues with large, fluctuating energy demands, such as skeletal muscle, heart, brain and spermatozoa.
Tpm4	Tropomyosin alpha-4 chain; Tropomyosin 4.
Tubb4b	Tubulin beta-4B chain; Tubulin is the major constituent of microtubules. It binds two moles of GTP, one at an exchangeable site on the beta chain and one at a non-exchangeable site on the alpha chain; Belongs to the tubulin family.
Vim	Vimentin; Vimentins are class-III intermediate filaments found in various non-epithelial cells, especially mesenchymal cells. Vimentin is attached to the nucleus, endoplasmic reticulum, and mitochondria, either laterally or terminally.
Eno1	Alpha-enolase; Multifunctional enzyme that, as well as its role in glycolysis, plays a part in various processes such as growth control, hypoxia tolerance and allergic responses. May also function in the intravascular and pericellular fibrinolytic system due to its ability to serve as a receptor and activator of plasminogen on the cell surface of several cell-types such as leukocytes and neurons. Stimulates immunoglobulin production; Belongs to the enolase family.
Gapdh	Glyceraldehyde-3-phosphate dehydrogenase; Has both glyceraldehyde-3-phosphate dehydrogenase and nitrosylase activities, thereby playing a role in glycolysis and nuclear functions, respectively. Participates in nuclear events including transcription, RNA transport, DNA replication and apoptosis. Nuclear functions are probably due to the nitrosylase activity that mediates cysteine S-nitrosylation of nuclear target proteins such as SIRT1, HDAC2 and PRKDC. Modulates the organization and assembly of the cytoskeleton. Facilitates the CHP1-dependent microtubule and membrane associations

Calr	Calreticulin; Calcium-binding chaperone that promotes folding, oligomeric assembly and quality control in the endoplasmic reticulum (ER) via the calreticulin/calnexin cycle. This lectin interacts transiently with almost all of the monoglucosylated glycoproteins that are synthesized in the ER. Interacts with the DNA-binding domain of NR3C1 and mediates its nuclear export. Involved in maternal gene expression regulation. May participate in oocyte maturation via the regulation of calcium homeostasis (By similarity).
TPM3	Tropomyosin alpha-3 chain; Binds to actin filaments in muscle and non-muscle cells. Plays a central role, in association with the troponin complex, in the calcium dependent regulation of vertebrate striated muscle contraction. Smooth muscle contraction is regulated by interaction with caldesmon. In non-muscle cells is implicated in stabilizing cytoskeleton actin filaments; Belongs to the tropomyosin family.
APP	Amyloid-beta A4 protein; N-APP binds TNFRSF21 triggering caspase activation and degeneration of both neuronal cell bodies (via caspase-3) and axons (via caspase-6).
APLP1	Amyloid-like protein 1; May play a role in postsynaptic function. The C-terminal gamma-secretase processed fragment, ALID1, activates transcription activation through APBB1 (Fe65) binding (By similarity). Couples to JIP signal transduction through C-terminal binding. May interact with cellular G-protein signaling pathways. Can regulate neurite outgrowth through binding to components of the extracellular matrix such as heparin and collagen I; Belongs to the APP family.
CDC25A	M-phase inducer phosphatase 1; Tyrosine protein phosphatase which functions as a dosage-dependent inducer of mitotic progression. Directly dephosphorylates CDK1 and stimulates its kinase activity. Also dephosphorylates CDK2 in complex with cyclin E, in vitro; Class III Cys-based CDC25 phosphatases.
CDC25B	M-phase inducer phosphatase 2; Tyrosine protein phosphatase which functions as a dosage-dependent inducer of mitotic progression. Required for G2/M phases of the cell cycle progression and abscission during cytokinesis in a ECT2-dependent manner. Directly dephosphorylates CDK1 and stimulates its kinase activity. The three isoforms seem to have a different level of activity.
CDC25C	M-phase inducer phosphatase 3; Functions as a dosage-dependent inducer in mitotic control. Tyrosine protein phosphatase required for progression of the cell cycle. When phosphorylated, highly effective in activating G2 cells into prophase. Directly dephosphorylates CDK1 and activates its kinase activity.
CDC42	Cell division control protein 42 homolog; Plasma membrane-associated small GTPase which cycles between an active GTP-bound and an inactive GDP-bound state. In active state binds to a variety of effector proteins to regulate cellular responses. Involved in epithelial cell polarization processes. Regulates the bipolar attachment of spindle microtubules to kinetochores before chromosome congression in metaphase. Plays a role in the extension and maintenance of the formation of thin, actin-rich surface projections called filopodia. Mediates CDC42-dependent cell migration.
ARHGEF5	Rho guanine nucleotide exchange factor 5; Guanine nucleotide exchange factor which activates Rho GTPases. Strongly activates RHOA. Also strongly activates RHOB, weakly activates RHOC and RHOG and shows no effect on RHOD, RHOV, RHOQ or RAC1 (By similarity). Involved in regulation of cell shape and actin cytoskeletal organization. Plays a role in actin organization by generating a loss of actin stress fibers and the formation of membrane ruffles and filopodia. Required for SRC-induced podosome formation (By similarity). Involved in positive regulation of immature dendritic cell migration.
ARHGEF15	Rho guanine nucleotide exchange factor 15; Specific GEF for RhoA activation. Does not activate RAC1 or CDC42. Regulates vascular smooth muscle contractility. Negatively regulates excitatory synapse development by suppressing the synapse-promoting activity of EPHB2.

ARHGEF26	Rho guanine nucleotide exchange factor 26; Activates RhoG GTPase by promoting the exchange of GDP by GTP. Required for the formation of membrane ruffles during macropinocytosis. Required for the formation of cup-like structures during trans-endothelial migration of leukocytes. In case of Salmonella enterica infection, activated by SopB, which induces cytoskeleton rearrangements and promotes bacterial entry; Pleckstrin homology domain containing.
NGEF	Ephexin-1; Acts as a guanine nucleotide exchange factor (GEF) which differentially activates the GTPases RHOA, RAC1 and CDC42. Plays a role in axon guidance regulating ephrin-induced growth cone collapse and dendritic spine morphogenesis. Upon activation by ephrin through EPHA4, the GEF activity switches toward RHOA resulting in its activation. Activated RHOA promotes cone retraction at the expense of RAC1- and CDC42-stimulated growth cone extension (By similarity).
DLL1	Delta-like protein 1; Transmembrane ligand protein of NOTCH1, NOTCH2 and NOTCH3 receptors that binds the extracellular domain (ECD) of Notch receptor in a cis and trans fashion manner. Following transinteraction, ligand cells produce mechanical force that depends of a clathrin-mediated endocytosis, requiring ligand ubiquitination, EPN1 interaction, and actin polymerisation; these events promote Notch receptor extracellular domain (NECD) transendocytosis and triggers Notch signaling.
JAG2	Protein jagged-2; Putative Notch ligand involved in the mediation of Notch signaling. Involved in limb development (By similarity).
RORA	Nuclear receptor ROR-alpha; Nuclear receptor that binds DNA as a monomer to ROR response elements (RORE) containing a single core motif half-site 5'-AGGTCA-3' preceded by a short A-T-rich sequence. Key regulator of embryonic development, cellular differentiation, immunity, circadian rhythm as well as lipid, steroid, xenobiotics and glucose metabolism. Considered to have intrinsic transcriptional activity, have some natural ligands like oxysterols that act as agonists (25-hydroxycholesterol) or inverse agonists (7-oxygenated sterols).
RORC	Nuclear receptor ROR-gamma; Nuclear receptor that binds DNA as a monomer to ROR response elements (RORE) containing a single core motif half-site 5'-AGGTCA-3' preceded by a short A-T-rich sequence. Key regulator of cellular differentiation, immunity, peripheral circadian rhythm as well as lipid, steroid, xenobiotics and glucose metabolism. Considered to have intrinsic transcriptional activity, have some natural ligands like oxysterols that act as agonists (25-hydroxycholesterol) or inverse agonists (7-oxygenated sterols), enhancing or repressing the transcriptional activity, respectively
ARHGAP30	Rho GTPase-activating protein 30; GTPase-activating protein (GAP) for RAC1 and RHOA, but not for CDC42.
ARHGAP31	Rho GTPase-activating protein 31; Functions as a GTPase-activating protein (GAP) for RAC1 and CDC42. Required for cell spreading, polarized lamellipodia formation and cell migration.
SDC1	Syndecan-1; Cell surface proteoglycan that bears both heparan sulfate and chondroitin sulfate and that links the cytoskeleton to the interstitial matrix. Regulates exosome biogenesis in concert with SDCBP and PDCD6IP.
SDC4	Syndecan-4; Cell surface proteoglycan that bears heparan sulfate. Regulates exosome biogenesis in concert with SDCBP and PDCD6IP; Belongs to the syndecan proteoglycan family.
EXOC6B	Exocyst complex component 6B; Component of the exocyst complex involved in the docking of exocytic vesicles with fusion sites on the plasma membrane; Belongs to the SEC15 family.
STXBP6	Syntaxin-binding protein 6; Forms non-fusogenic complexes with SNAP25 and STX1A and may thereby modulate the formation of functional SNARE complexes and exocytosis.
FERMT3	Fermitin family homolog 3; Plays a central role in cell adhesion in hematopoietic cells. Acts by activating the integrin beta-1-3 (ITGB1, ITGB2 and ITGB3) (By similarity). Required for integrin-mediated platelet adhesion and leukocyte adhesion to endothelial cells. Required for activation of integrin beta-2 (ITGB2) in polymorphonuclear granulocytes (PMNs) (By similarity); Belongs to the kindlin family.

CCT2	T-complex protein 1 subunit beta; Molecular chaperone; assists the folding of proteins upon ATP hydrolysis. As part of the BBS/CCT complex may play a role in the assembly of BBSome, a complex involved in ciliogenesis regulating transports vesicles to the cilia. Known to play a role, in vitro, in the folding of actin and tubulin.
CDH5	Cadherin-5; Cadherins are calcium-dependent cell adhesion proteins. They preferentially interact with themselves in a homophilic manner in connecting cells; cadherins may thus contribute to the sorting of heterogeneous cell types. This cadherin may play a important role in endothelial cell biology through control of the cohesion and organization of the intercellular junctions. It associates with alpha-catenin forming a link to the cytoskeleton. Acts in concert with KRIT1 to establish and maintain correct endothelial cell polarity and vascular lumen.
CAPN2	Calpain-2 catalytic subunit; Calcium-regulated non-lysosomal thiol-protease which catalyzes limited proteolysis of substrates involved in cytoskeletal remodeling and signal transduction. Proteolytically cleaves MYOC at 'Arg-226'. Proteolytically cleaves CPEB3 following neuronal stimulation which abolishes CPEB3 translational repressor activity, leading to translation of CPEB3 target mRNAs (By similarity).
MCM3	DNA replication licensing factor MCM3; Acts as component of the MCM2-7 complex (MCM complex) which is the putative replicative helicase essential for 'once per cell cycle' DNA replication initiation and elongation in eukaryotic cells. The active ATPase sites in the MCM2-7 ring are formed through the interaction surfaces of two neighboring subunits such that a critical structure of a conserved arginine finger motif is provided in trans relative to the ATP-binding site of the Walker A box of the adjacent subunit.
SUN1	SUN domain-containing protein 1; As a component of the LINC (LInker of Nucleoskeleton and Cytoskeleton) complex involved in the connection between the nuclear lamina and the cytoskeleton. The nucleocytoplasmic interactions established by the LINC complex play an important role in the transmission of mechanical forces across the nuclear envelope and in nuclear movement and positioning. Required for interkinetic nuclear migration (INM) and essential for nucleokinesis and centrosome-nucleus coupling during radial neuronal migration in the cerebral cortex and during glial migration.
GLOD4	Glyoxalase domain containing 4
GAPDH	Glyceraldehyde-3-phosphate dehydrogenase; Has both glyceraldehyde-3-phosphate dehydrogenase and nitrosylase activities, thereby playing a role in glycolysis and nuclear functions, respectively. Participates in nuclear events including transcription, RNA transport, DNA replication and apoptosis. Nuclear functions are probably due to the nitrosylase activity that mediates cysteine S-nitrosylation of nuclear target proteins such as SIRT1, HDAC2 and PRKDC. Modulates the organization and assembly of the cytoskeleton. Facilitates the CHP1-dependent microtubule and membrane association.
CNN3	Calponin-3; Thin filament-associated protein that is implicated in the regulation and modulation of smooth muscle contraction. It is capable of binding to actin, calmodulin, troponin C and tropomyosin. The interaction of calponin with actin inhibits the actomyosin Mg-ATPase activity.
NOS3	Nitric oxide synthase, endothelial; Produces nitric oxide (NO) which is implicated in vascular smooth muscle relaxation through a cGMP-mediated signal transduction pathway. NO mediates vascular endothelial growth factor (VEGF)-induced angiogenesis in coronary vessels and promotes blood clotting through the activation of platelets.
PRKCSH	Glucosidase 2 subunit beta; Regulatory subunit of glucosidase II; EF-hand domain containing.
DDB1	DNA damage-binding protein 1; Required for DNA repair. Binds to DDB2 to form the UV-damaged DNA-binding protein complex (the UV-DDB complex). The UV- DDB complex may recognize UV-induced DNA damage and recruit proteins of the nucleotide excision repair pathway (the NER pathway) to initiate DNA repair. The UV-DDB complex preferentially binds to cyclobutane pyrimidine dimers (CPD), 6-4 photoproducts (6-4 PP), apurinic sites and short mismatches. Also appears to function as a component of numerous distinct DCX (DDB1-CUL4-X-box) E3 ubiquitin-protein ligase complexes.

CAV1	Caveolin-1; May act as a scaffolding protein within caveolar membranes. Interacts directly with G-protein alpha subunits and can functionally regulate their activity (By similarity). Involved in the costimulatory signal essential for T-cell receptor (TCR)- mediated T-cell activation. Its binding to DPP4 induces T-cell proliferation and NF-kappa-B activation in a T-cell receptor/CD3-dependent manner. Recruits CTNNB1 to caveolar membranes and may regulate CTNNB1-mediated signaling through the Wnt pathway. Negatively regulates TGFB1-mediated activation of SMAD2/3.
HSPB1	Heat shock protein beta-1; Small heat shock protein which functions as a molecular chaperone probably maintaining denatured proteins in a folding- competent state. Plays a role in stress resistance and actin organization. Through its molecular chaperone activity may regulate numerous biological processes including the phosphorylation and the axonal transport of neurofilament proteins.
SFPQ	Splicing factor, proline- and glutamine-rich; DNA- and RNA binding protein, involved in several nuclear processes. Essential pre-mRNA splicing factor required early in spliceosome formation and for splicing catalytic step II, probably as a heteromer with NONO. Binds to pre-mRNA in spliceosome C complex, and specifically binds to intronic polypyrimidine tracts. Involved in regulation of signal-induced alternative splicing. During splicing of PTPRC/CD45, a phosphorylated form is sequestered by THRAP3 from the pre-mRNA in resting T-cells.
PLOD2	Procollagen-lysine,2-oxoglutarate 5-dioxygenase 2; Forms hydroxylysine residues in -Xaa-Lys-Gly- sequences in collagens. These hydroxylysines serve as sites of attachment for carbohydrate units and are essential for the stability of the intermolecular collagen cross-links.
APBA2	Amyloid-beta A4 precursor protein-binding family A member 2; Putative function in synaptic vesicle exocytosis by binding to STXBP1, an essential component of the synaptic vesicle exocytotic machinery. May modulate processing of the amyloid-beta precursor protein (APP) and hence formation of APP-beta; PDZ domain containing.
ITGB4	Integrin beta-4; Integrin alpha-6/beta-4 is a receptor for laminin. Plays a critical structural role in the hemidesmosome of epithelial cells. Is required for the regulation of keratinocyte polarity and motility. ITGA6:ITGB4 binds to NRG1 (via EGF domain) and this binding is essential for NRG1-ERBB signaling. ITGA6:ITGB4 binds to IGF1 and this binding is essential for IGF1 signaling
TJP1	Tight junction protein ZO-1; The N-terminal may be involved in transducing a signal required for tight junction assembly, while the C-terminal may have specific properties of tight junctions. The alpha domain might be involved in stabilizing junctions. Plays a role in the regulation of cell migration by targeting CDC42BPB to the leading edge of migrating cells; Belongs to the MAGUK family.
CCL2	C-C motif chemokine 2; Chemotactic factor that attracts monocytes and basophils but not neutrophils or eosinophils. Augments monocyte anti-tumor activity. Has been implicated in the pathogenesis of diseases characterized by monocytic infiltrates, like psoriasis, rheumatoid arthritis or atherosclerosis. May be involved in the recruitment of monocytes into the arterial wall during the disease process of atherosclerosis; Belongs to the intercrine beta (chemokine CC) family.

Supplemental Table 3A. Detailed description of altered genes in CCM models with 2 validations. A total of 152 genes were analyzed that overlapped in two different CCM studies. Details provided for each protein include mechanisms associated with each, functions, binding partners, motifs and domains. Protein details were extracted from STRING enrichment data after construction of Figure 1 Interactome.
