# Supplementary material for: Comparative omics of CCM signaling complex (CSC)
Source: Chin Neurosurg J. 2020 Jan 15;6:4. doi: 10.1186/s41016-019-0183-6 (PMC7398211; doi:10.1186/s41016-019-0183-6)
Supplement: Supplementary file 5 — Additional file 5: Table S3B. Altered genes in CCM models with 2 validations enrichment data. An enrichment category was exported along with Fig. 1 that detailed altered pathways involved with the identified 152 genes. Information provided in the table includes the number of enriched genes in each enrichment category, description of the category, genes specifically involved, FDR value, and the term name for each category (which includes GO terms if applicable). Among the exported pathways identified in this group, a filter search was applied to identify all enrichment data involved with angiogenesis specifically which are bolded and re-summarized in Table 1. [file 41016_2019_183_MOESM5_ESM.pdf]

Altered genes in CCM models with 2 validations enrichment data.

<table>
<tr><th># enriched genes</th><th>category</th><th>description</th><th>enriched genes</th><th>FDR value</th><th>term name</th></tr>
<tr><td>28</td><td>GO Component</td><td>actin cytoskeleton</td><td>ARHGEF5|MYH9|CNN2|ADD1|FERMT3|TJP1|FLT1|CAPN2|SDPR|ZYX|SNCA|TPM4|FYN|MYO1C|MYH10|TPM3|FLNA|CNN3|SPTAN1|CLIC4|TPM2|FSCN1|MYO1B|SEPT7|FLNB|SPTBN2|CFL1|TLN2</td><td>6.94E-14</td><td>GO.0015629</td></tr>
<tr><td>130</td><td>GO Component</td><td>cytoplasmic part</td><td>RCN1|ARHGEF5|MAP2K5|PLOD1|MYH9|CYP24A1|MMP2|EHD4|APLP1|GAPDH|RBP1|GNB4|PPP3R1|PLEK|ENO1|MT2A|CDC25B|HSPB1|BCL2L2|PXDN|ARF3|THBS1|COL8A1|VWF|DNAJB6|CNN2|PRKCG|NGEF|TUBB4A|ARHGAP31|CAD|ADD1|EXOC6B|FERMT3|TJP1|FLT1|PLOD2|APP|KIT|NPC1L1|CCR5|CAPN2|ANXA5|ITGA2|NOS3|MELK|CKB|CCT2|PLK1|TUBA1A|GLOD4|BCL2L1|CDC25A|SDPR|ARF4|MMP14|BRSK1|DAB2|NQO1|CALR|CDC25C|STXBP6|ZYX|FLNC|PRKCH|CYP2R1|LCK|SNCA|CAV1|RGS12|TUBB4B|PTGES|DOK4|PTMA|TPM4|ANXA2|ANXA6|FYN|FN1|FAS|ARHGEF26|PTRF|MYO1C|MYH10|DPP4|TGM2|DLL1|ARHGAP30|COPA|TPM3|GSTO1|SLK|FLNA|CNN3|PTPN1|SDC4|SPTAN1|DNM1|TLR4|CLIC4|ANXA1|TBC1D4|TPM2|PIR|PSIP1|SDC1|FSCN1|XRCC5|MYO1B|G6PD|PPP3CA|CAMK2B|LDHB|BCL2|CAMK2A|SEPT7|PAICS|CDC42|SUN1|PDIA6|TOP2B|FLNB|SPTBN2|CFL1|ARF1|VIM|NUMB|APBA2|PRKCSH|MCM3</td><td>6.94E-14</td><td>GO.0044444</td></tr>
<tr><td>142</td><td>GO Component</td><td>cytoplasm</td><td>RCN1|ARHGEF5|MAP2K5|PLOD1|MYH9|CYP24A1|MMP2|EHD4|APLP1|GAPDH|RBP1|GNB4|PPP3R1|PLEK|ENO1|MT2A|CDC25B|HSPB1|BCL2L2|PXDN|ARF3|PDGFRA|THBS1|COL8A1|VWF|DNAJB6|CNN2|PRKCG|AKT3|NGEF|TUBB4A|ARHGAP31|CAD|ADD1|EXOC6B|FERMT3|TJP1|FLT1|PLOD2|APP|KIT|NPC1L1|CCR5|CAPN2|ANXA5|ITGA2|NOS3|MELK|CKB|CCT2|PLK1|TUBA1A|TUBA1C|GLOD4|DDB1|BCL2L1|CDC25A|SDPR|ARF4|MMP14|BRSK1|DAB2|NQO1|CALR|CDC25C|STXBP6|ZYX|FLNC|PRKCH|RARB|CYP2R1|LCK|SNCA|CAV1|RGS12|TUBB4B|PTGES|MAP4K4|DOK4|PTMA|TPM4|ANXA2|ANXA6|FYN|FN1|FAS|ARHGEF26|PTRF|SFPQ|MYO1C|MYH10|DPP4|ARHGEF15|TGM2|DLL1|ARHGAP30|COPA|TPM3|GSTO1|SLK|FLNA|CNN3|PTPN1|SDC4|SPTAN1|DNM1|TLR4|CLIC4|ANXA1|TBC1D4|TPM2|PIR|PSIP1|SDC1|FSCN1|XRCC5|MYO1B|G6PD|PPP3CA|CAMK2B|LDHB|BCL2|CAMK2A|DYRK1A|SEPT7|PAICS|CDC42|SUN1|PDIA6|TOP2B|WEE1|FLNB|SPTBN2|CFL1|ARF1|VIM|NUMB|APBA2|TLN2|PRKCSH|CAMK1D|MCM3</td><td>1.28E-13</td><td>GO.0005737</td></tr>
<tr><td>88</td><td>GO Component</td><td>cytosol</td><td>ARHGEF5|MAP2K5|MYH9|GAPDH|RBP1|GNB4|PPP3R1|PLEK|ENO1|MT2A|CDC25B|HSPB1|BCL2L2|DNAJB6|PRKCG|NGEF|TUBB4A|ARHGAP31|CAD|ADD1|TJP1|APP|CAPN2|ANXA5|NOS3|CKB|CCT2|PLK1|TUBA1A|BCL2L1|CDC25A|SDPR|ARF4|MMP14|DAB2|NQO1|CALR|CDC25C|ZYX|FLNC|PRKCH|LCK|SNCA|RGS12|TUBB4B|DOK4|PTMA|TPM4|ANXA2|FYN|FAS|ARHGEF26|PTRF|MYO1C|MYH10|TGM2|ARHGAP30|COPA|TPM3|GSTO1|FLNA|CNN3|PTPN1|SPTAN1|CLIC4|ANXA1|TBC1D4|TPM2|PIR|PSIP1|FSCN1|XRCC5|G6PD|PPP3CA|CAMK2B|LDHB|BCL2|CAMK2A|SEPT7|PAICS|CDC42|PDIA6|TOP2B|FLNB|SPTBN2|CFL1|ARF1|VIM</td><td>1.72E-12</td><td>GO.0005829</td></tr>
<tr><td>52</td><td>GO Component</td><td>cytoskeleton</td><td>ARHGEF5|MAP2K5|MYH9|GAPDH|CDC25B|HSPB1|CNN2|TUBB4A|ADD1|FERMT3|TJP1|FLT1|APP|CAPN2|NOS3|CCT2|PLK1|TUBA1A|TUBA1C|BCL2L1|SDPR|MMP14|BRSK1|ZYX|FLNC|NR1I2|LCK|SNCA|TUBB4B|TPM4|FYN|MYO1C|MYH10|TPM3|FLNA|CNN3|SPTAN1|DNM1|CLIC4|TPM2|FSCN1|MYO1B|CAMK2B|DYRK1A|SEPT7|CDC42|FLNB|SPTBN2|CFL1|VIM|TLN2|MCM3</td><td>2.51E-11</td><td>GO.0005856</td></tr>
<tr><td>155</td><td>GO Component</td><td>intracellular</td><td>RCN1|ARHGEF5|MAP2K5|PLOD1|MYH9|CYP24A1|MMP2|EHD4|APLP1|CCL2|GAPDH|RBP1|GNB4|PPP3R1|PLEK|ENO1|MT2A|CDC25B|HSPB1|BCL2L2|PXDN|ARF3|PDGFRA|THBS1|COL8A1|VWF|RORA|DNAJB6|CNN2|PRKCG|AKT3|NGEF|TUBB4A|ARHGAP31|CAD|ADD1|EXOC6B|FERMT3|TJP1|FLT1|PLOD2|APP|CSF1R|KIT|NPC1L1|CCR5|CAPN2|ANXA5|ITGA2|NOS3|MELK|CKB|CCT2|PLK1|TUBA1A|TUBA1C|GLOD4|DDB1|BCL2L1|CDC25A|SDPR|ARF4|MMP14|BRSK1|DAB2|TENC1|NQO1|CALR|CDC25C|STXBP6|ZYX|RORC|FLNC|PRKCH|RARB|CYP2R1|NR1I2|LCK|SNCA|CAV1|RGS12|TUBB4B|PTGES|USP1|MAP4K4|DOK4|PTMA|TPM4|ANXA2|ANXA6|FYN|FN1|FAS|ARHGEF26|PTRF|SFPQ|EPHA2|MYO1C|RXRG|MYH10|DPP4|ARHGEF15|TGM2|DLL1|ARHGAP30|COPA|TPM3|GSTO1|SLK|FLNA|CNN3|PTPN1|SDC4|SPTAN1|DNM1|TLR4|CLIC4|RXRB|ANXA1|TBC1D4|TPM2|PIR|PSIP1|SDC1|FSCN1|XRCC5|MYO1B|G6PD|PPP3CA|CAMK2B|LDHB|BCL2|CAMK2A|DYRK1A|SEPT7|PAICS|CDC42|SUN1|PDIA6|RARG|TOP2B|WEE1|FLNB|PBK|SPTBN2|CFL1|ARF1|VIM|NR1H4|NUMB|APBA2|TLN2|PRKCSH|CAMK1D|MCM3</td><td>1.12E-10</td><td>GO.0005622</td></tr>
<tr><td>48</td><td>GO Component</td><td>cell projection</td><td>ARHGEF5|MYH9|PLEK|HSPB1|PDGFRA|PRKCG|NGEF|TUBB4A|ARHGAP31|CAD|FERMT3|TJP1|APP|NPC1L1|CAPN2|ITGA2|CKB|ARF4|BRSK1|NQO1|SNCA|CAV1|RGS12|ANXA2|FYN|ARHGEF26|EPHA2|MYO1C|MYH10|DPP4|ARHGEF15|FLNA|CNN3|DNM1|CLIC4|ANXA1|FSCN1|MYO1B|PPP3CA|CAMK2B|CAMK2A|DYRK1A|SEPT7|CDC42|CFL1|ARF1|VIM|TLN2</td><td>6.20E-10</td><td>GO.0042995</td></tr>
<tr><td>152</td><td>GO Component</td><td>intracellular part</td><td>RCN1|ARHGEF5|MAP2K5|PLOD1|MYH9|CYP24A1|MMP2|EHD4|APLP1|GAPDH|RBP1|GNB4|PPP3R1|PLEK|ENO1|MT2A|CDC25B|HSPB1|BCL2L2|PXDN|ARF3|PDGFRA|THBS1|COL8A1|VWF|RORA|DNAJB6|CNN2|PRKCG|AKT3|NGEF|TUBB4A|ARHGAP31|CAD|ADD1|EXOC6B|FERMT3|TJP1|FLT1|PLOD2|APP|CSF1R|KIT|NPC1L1|CCR5|CAPN2|ANXA5|ITGA2|NOS3|MELK|CKB|CCT2|PLK1|TUBA1A|TUBA1C|GLOD4|DDB1|BCL2L1|CDC25A|SDPR|ARF4|MMP14|BRSK1|DAB2|NQO1|CALR|CDC25C|STXBP6|ZYX|RORC|FLNC|PRKCH|RARB|CYP2R1|NR1I2|LCK|SNCA|CAV1|RGS12|TUBB4B|PTGES|USP1|MAP4K4|DOK4|PTMA|TPM4|ANXA2|ANXA6|FYN|FN1|FAS|ARHGEF26|PTRF|SFPQ|MYO1C|RXRG|MYH10|DPP4|ARHGEF15|TGM2|DLL1|ARHGAP30|COPA|TPM3|GSTO1|SLK|FLNA|CNN3|PTPN1|SDC4|SPTAN1|DNM1|TLR4|CLIC4|RXRB|ANXA1|TBC1D4|TPM2|PIR|PSIP1|SDC1|FSCN1|XRCC5|MYO1B|G6PD|PPP3CA|CAMK2B|LDHB|BCL2|CAMK2A|DYRK1A|SEPT7|PAICS|CDC42|SUN1|PDIA6|RARG|TOP2B|WEE1|FLNB|PBK|SPTBN2|CFL1|ARF1|VIM|NR1H4|NUMB|APBA2|TLN2|PRKCSH|CAMK1D|MCM3</td><td>1.03E-09</td><td>GO.0044424</td></tr>
<tr><td>33</td><td>GO Component</td><td>cell junction</td><td>ARHGEF5|ITGB4|MYH9|PDGFRA|CNN2|PRKCG|ARHGAP31|ADD1|FERMT3|TJP1|APP|KIT|ITGA2|BCL2L1|BRSK1|TENC1|ZYX|PRKCH|SNCA|RGS12|CDH5|EPHA2|DPP4|DLL1|FLNA|SDC4|CLIC4|FSCN1|PPP3CA|CAMK2B|CAMK2A|SPTBN2|CFL1</td><td>1.26E-09</td><td>GO.0030054</td></tr>
<tr><td>41</td><td>GO Component</td><td>cytoskeletal part</td><td>ARHGEF5|MAP2K5|MYH9|CDC25B|HSPB1|CNN2|TUBB4A|ADD1|FERMT3|TJP1|APP|CAPN2|CCT2|PLK1|TUBA1A|TUBA1C|BCL2L1|BRSK1|ZYX|LCK|TUBB4B|TPM4|FYN|MYO1C|MYH10|TPM3|FLNA|SPTAN1|DNM1|CLIC4|TPM2|FSCN1|MYO1B|CAMK2B|SEPT7|CDC42|FLNB|SPTBN2|CFL1|VIM|MCM3</td><td>1.88E-09</td><td>GO.0044430</td></tr>
<tr><td>84</td><td>GO Component</td><td>cell periphery</td><td>ARHGEF5|ITGB4|MYH9|CYP24A1|MMP2|EHD4|APLP1|GAPDH|PPP3R1|PLEK|ENO1|HSPB1|PDGFRA|THBS1|PRKCG|ADD1|ICAM1|EXOC6B|TJP1|FLT1|APP|CSF1R|KIT|NPC1L1|CCR5|CAPN2|ANXA5|ITGA2|NOS3|MELK|SDPR|ARF4|MMP14|DAB2|TENC1|CALR|STXBP6|FLNC|JAG2|PRKCH|LCK|SNCA|CAV1|RGS12|CDH5|ANXA2|FYN|FN1|FAS|PTRF|EPHA2|MYO1C|MYH10|DPP4|TGM2|DLL1|FLNA|SDC4|SPTAN1|DNM1|TLR4|CLIC4|ANXA1|SDC1|FSCN1|XRCC5|MYO1B|G6PD|PPP3CA|CAMK2B|LTB4R|CAMK2A|SEPT7|CDC42|PDIA6|FLNB|SPTBN2|CFL1|ARF1|VIM|NUMB|APBA2|TLN2|ABCB1</td><td>1.88E-09</td><td>GO.0071944</td></tr>
</table>

<table>
<tr><td>82</td><td>GO Component</td><td>plasma membrane</td><td>ARHGEF5|ITGB4|MYH9|CYP24A1|MMP2|EHD4|APLP1|GAPDH|PPP3R1|PLEK|ENO1|HSPB1|PDGFRA|THBS1|PRKCG|ADD1|ICAM1|TJP1|FLT1|APP|CSF1R|KIT|NPC1L1|CCR5|CAPN2|ANXA5|ITGA2|NOS3|MELK|SDPR|ARF4|MMP14|DAB2|TENC1|CALR|STXBP6|FLNC|JAG2|PRKCH|LCK|SNCA|CAV1|RGS12|CDH5|ANXA2|FYN|FN1|FAS|PTRF|EPHA2|MYO1C|MYH10|DPP4|TGM2|DLL1|FLNA|SDC4|DNM1|TLR4|CLIC4|ANXA1|SDC1|FSCN1|XRCC5|MYO1B|G6PD|PPP3CA|CAMK2B|LTB4R|CAMK2A|SEPT7|CDC42|PDIA6|FLNB|SPTBN2|CFL1|ARF1|VIM|NUMB|APBA2|TLN2|ABCB1</td><td>4.84E-09</td><td>GO.0005886</td></tr>
<tr><td>45</td><td>GO Component</td><td>plasma membrane bounded cell projection</td><td>MYH9|PLEK|HSPB1|PDGFRA|PRKCG|NGEF|TUBB4A|ARHGAP31|CAD|APP|NPC1L1|CAPN2|ITGA2|CKB|ARF4|BRSK1|NQO1|SNCA|CAV1|RGS12|ANXA2|FYN|ARHGEF26|EPHA2|MYO1C|MYH10|DPP4|ARHGEF15|FLNA|CNN3|DNM1|CLIC4|ANXA1|FSCN1|MYO1B|PPP3CA|CAMK2B|CAMK2A|DYRK1A|SEPT7|CDC42|CFL1|ARF1|VIM|TLN2</td><td>4.84E-09</td><td>GO.0120025</td></tr>
<tr><td>18</td><td>GO Component</td><td>membrane raft</td><td>ICAM1|APP|CAPN2|NOS3|TUBA1A|SDPR|LCK|CAV1|ANXA2|FYN|FAS|PTRF|MYO1C|DPP4|DLL1|SDC4|LDHB|LTB4R</td><td>6.24E-09</td><td>GO.0045121</td></tr>
<tr><td>16</td><td>GO Component</td><td>cell cortex</td><td>MYH9|ENO1|EXOC6B|CAPN2|MELK|STXBP6|SNCA|CAV1|ANXA2|MYH10|FLNA|SPTAN1|SEPT7|FLNB|SPTBN2|CFL1</td><td>7.82E-09</td><td>GO.0005938</td></tr>
<tr><td>69</td><td>GO Component</td><td>intracellular non-membrane-bounded organelle</td><td>ARHGEF5|MAP2K5|MYH9|MMP2|GAPDH|RBP1|ENO1|CDC25B|HSPB1|DNAJB6|CNN2|TUBB4A|ADD1|FERMT3|TJP1|FLT1|APP|CAPN2|NOS3|CCT2|PLK1|TUBA1A|TUBA1C|BCL2L1|SDPR|MMP14|BRSK1|DAB2|ZYX|FLNC|NR1I2|LCK|SNCA|CAV1|RGS12|TUBB4B|TPM4|ANXA2|FYN|SFPQ|MYO1C|MYH10|TPM3|FLNA|CNN3|SDC4|SPTAN1|DNM1|CLIC4|TPM2|PSIP1|FSCN1|XRCC5|MYO1B|PPP3CA|CAMK2B|DYRK1A|SEPT7|CDC42|RARG|TOP2B|WEE1|FLNB|SPTBN2|CFL1|VIM|NR1H4|TLN2|MCM3</td><td>1.01E-08</td><td>GO.0043232</td></tr>
<tr><td>161</td><td>GO Component</td><td>cell part</td><td>RCN1|ARHGEF5|MAP2K5|PLOD1|ITGB4|MYH9|CYP24A1|MMP2|EHD4|APLP1|CCL2|GAPDH|RBP1|GNB4|PPP3R1|PLEK|ENO1|MT2A|CDC25B|HSPB1|BCL2L2|PXDN|ARF3|PDGFRA|THBS1|COL8A1|VWF|RORA|DNAJB6|CNN2|PRKCG|AKT3|NGEF|TUBB4A|ARHGAP31|CAD|ADD1|ICAM1|EXOC6B|FERMT3|TJP1|FLT1|PLOD2|APP|CSF1R|KIT|NPC1L1|CCR5|CAPN2|ANXA5|ITGA2|NOS3|MELK|CKB|CCT2|PLK1|TUBA1A|TUBA1C|GLOD4|DDB1|BCL2L1|CDC25A|SDPR|ARF4|MMP14|BRSK1|DAB2|TENC1|NQO1|CALR|CDC25C|STXBP6|ZYX|RORC|FLNC|JAG2|PRKCH|RARB|CYP2R1|NR1I2|LCK|SNCA|CAV1|RGS12|TUBB4B|PTGES|USP1|MAP4K4|CDH5|DOK4|PTMA|TPM4|ANXA2|ANXA6|FYN|FN1|FAS|ARHGEF26|PTRF|SFPQ|EPHA2|MYO1C|RXRG|MYH10|DPP4|ARHGEF15|TGM2|DLL1|ARHGAP30|COPA|TPM3|GSTO1|SLK|FLNA|CNN3|PTPN1|SDC4|SPTAN1|DNM1|TLR4|CLIC4|RXRB|ANXA1|TBC1D4|TPM2|PIR|PSIP1|SDC1|FSCN1|XRCC5|MYO1B|G6PD|PPP3CA|CAMK2B|LDHB|LTB4R|BCL2|CAMK2A|DYRK1A|SEPT7|PAICS|CDC42|SUN1|PDIA6|RARG|TOP2B|WEE1|FLNB|PBK|SPTBN2|CFL1|ARF1|VIM|NR1H4|NUMB|APBA2|TLN2|PRKCSH|ABCB1|CAMK1D|MCM3</td><td>1.06E-08</td><td>GO.0044464</td></tr>
<tr><td>49</td><td>GO Component</td><td>vesicle</td><td>EHD4|THBS1|VWF|CNN2|ICAM1|FERMT3|FLT1|APP|KIT|NPC1L1|CCR5|NOS3|CCT2|TUBA1A|BCL2L1|MMP14|BRSK1|DAB2|CALR|ZYX|SNCA|CAV1|TUBB4B|ANXA2|ANXA6|FYN|FN1|MYO1C|DPP4|DLL1|ARHGAP30|COPA|PTPN1|SPTAN1|DNM1|TLR4|CLIC4|ANXA1|TBC1D4|XRCC5|MYO1B|CAMK2B|CAMK2A|SUN1|PDIA6|FLNB|VIM|NUMB|APBA2</td><td>1.53E-08</td><td>GO.0031982</td></tr>
<tr><td>25</td><td>GO Component</td><td>perinuclear region of cytoplasm</td><td>EHD4|APLP1|GAPDH|MT2A|ARF3|DNAJB6|PRKCG|APP|CAPN2|ITGA2|CALR|CDC25C|SNCA|CAV1|PTGES|ANXA2|ANXA6|FYN|SLK|FLNA|TLR4|CLIC4|MYO1B|ARF1|MCM3</td><td>1.53E-08</td><td>GO.0048471</td></tr>
<tr><td>9</td><td>GO Component</td><td>stress fiber</td><td>MYH9|CNN2|ZYX|TPM4|MYH10|TPM3|FSCN1|SEPT7|FLNB</td><td>2.79E-08</td><td>GO.0001725</td></tr>
<tr><td>47</td><td>GO Component</td><td>cytoplasmic vesicle</td><td>EHD4|THBS1|VWF|CNN2|FERMT3|FLT1|APP|KIT|NPC1L1|CCR5|NOS3|CCT2|TUBA1A|BCL2L1|MMP14|BRSK1|DAB2|CALR|ZYX|SNCA|CAV1|TUBB4B|ANXA2|ANXA6|FYN|FN1|MYO1C|DPP4|DLL1|ARHGAP30|COPA|PTPN1|SPTAN1|DNM1|TLR4|CLIC4|ANXA1|XRCC5|MYO1B|CAMK2B|CAMK2A|SUN1|PDIA6|FLNB|VIM|NUMB|APBA2</td><td>3.45E-08</td><td>GO.0031410</td></tr>
<tr><td>18</td><td>GO Component</td><td>cell leading edge</td><td>ITGB4|MYH9|PLEK|ARHGAP31|APP|ARF4|ANXA2|ARHGEF26|EPHA2|MYO1C|MYH10|DPP4|SLK|FSCN1|CFL1|ARF1|VIM|TLN2</td><td>7.49E-08</td><td>GO.0031252</td></tr>
<tr><td>18</td><td>GO Component</td><td>cytoplasmic region</td><td>MYH9|ENO1|HSPB1|TUBB4A|EXOC6B|CAPN2|MELK|STXBP6|SNCA|CAV1|ANXA2|MYH10|FLNA|SPTAN1|SEPT7|FLNB|SPTBN2|CFL1</td><td>2.30E-07</td><td>GO.0099568</td></tr>
<tr><td>50</td><td>GO Component</td><td>plasma membrane part</td><td>ITGB4|MYH9|PLEK|PDGFRA|THBS1|PRKCG|ICAM1|TJP1|FLT1|APP|CSF1R|KIT|NPC1L1|CCR5|ANXA5|ITGA2|NOS3|SDPR|ARF4|MMP14|CALR|JAG2|LCK|CAV1|CDH5|ANXA2|FYN|FN1|FAS|PTRF|EPHA2|MYO1C|MYH10|DPP4|TGM2|DLL1|SDC4|TLR4|ANXA1|SDC1|FSCN1|G6PD|LTB4R|CAMK2A|SEPT7|SPTBN2|CFL1|ARF1|NUMB|ABCB1</td><td>2.95E-07</td><td>GO.0044459</td></tr>
<tr><td>23</td><td>GO Component</td><td>cell surface</td><td>ITGB4|ENO1|PDGFRA|THBS1|ICAM1|APP|CSF1R|KIT|CCR5|ANXA5|ITGA2|CALR|CDH5|ANXA2|FAS|EPHA2|DPP4|SDC4|TLR4|CLIC4|ANXA1|SDC1|ABCB1</td><td>4.01E-07</td><td>GO.0009986</td></tr>
<tr><td>29</td><td>GO Component</td><td>neuron projection</td><td>HSPB1|PRKCG|NGEF|TUBB4A|CAD|APP|CAPN2|ITGA2|CKB|ARF4|BRSK1|NQO1|SNCA|RGS12|FYN|MYO1C|MYH10|ARHGEF15|FLNA|CNN3|DNM1|FSCN1|PPP3CA|CAMK2B|CAMK2A|DYRK1A|CDC42|ARF1|VIM</td><td>1.67E-06</td><td>GO.0043005</td></tr>
<tr><td>135</td><td>GO Component</td><td>organelle</td><td>RCN1|ARHGEF5|MAP2K5|PLOD1|MYH9|CYP24A1|MMP2|EHD4|GAPDH|RBP1|PPP3R1|ENO1|MT2A|CDC25B|HSPB1|BCL2L2|PXDN|ARF3|PDGFRA|THBS1|COL8A1|VWF|RORA|DNAJB6|CNN2|PRKCG|AKT3|TUBB4A|CAD|ADD1|ICAM1|FERMT3|TJP1|FLT1|PLOD2|APP|CSF1R|KIT|NPC1L1|CCR5|CAPN2|ITGA2|NOS3|MELK|CKB|CCT2|PLK1|TUBA1A|TUBA1C|GLOD4|DDB1|BCL2L1|CDC25A|SDPR|ARF4|MMP14|BRSK1|DAB2|CALR|CDC25C|ZYX|RORC|FLNC|RARB|CYP2R1|NR1I2|LCK|SNCA|CAV1|RGS12|TUBB4B|PTGES|USP1|PTMA|TPM4|ANXA2|ANXA6|FYN|FN1|FAS|PTRF|SFPQ|MYO1C|RXRG|MYH10|DPP4|TGM2|DLL1|ARHGAP30|COPA|TPM3|FLNA|CNN3|PTPN1|SDC4|SPTAN1|DNM1|TLR4|CLIC4|RXRB|ANXA1|TBC1D4|TPM2|PIR|PSIP1|SDC1|FSCN1|XRCC5|MYO1B|G6PD|PPP3CA|CAMK2B|BCL2|CAMK2A|DYRK1A|SEPT7|CDC42|SUN1|PDIA6|RARG|TOP2B|WEE1|FLNB|PBK|SPTBN2|CFL1|ARF1|VIM|NR1H4|NUMB|APBA2|TLN2|PRKCSH|CAMK1D|MCM3</td><td>1.75E-06</td><td>GO.0043226</td></tr>
<tr><td>25</td><td>GO Component</td><td>supramolecular polymer</td><td>MMP2|ENO1|HSPB1|COL8A1|DNAJB6|TUBB4A|CCT2|TUBA1A|TUBA1C|FLNC|SNCA|TUBB4B|TPM4|FYN|MYO1C|TPM3|FLNA|SDC4|DNM1|TPM2|FSCN1|MYO1B|PPP3CA|FLNB|VIM</td><td>1.75E-06</td><td>GO.0099081</td></tr>
<tr><td>10</td><td>GO Component</td><td>cell cortex part</td><td>MYH9|ENO1|EXOC6B|CAPN2|STXBP6|FLNA|SPTAN1|SEPT7|SPTBN2|CFL1</td><td>1.78E-06</td><td>GO.0044448</td></tr>
<tr><td>11</td><td>GO Component</td><td>myelin sheath</td><td>GNB4|TUBB4A|CKB|CCT2|TUBA1A|TUBB4B|ANXA2|DNM1|FSCN1|LDHB|BCL2</td><td>2.11E-06</td><td>GO.0043209</td></tr>
<tr><td>133</td><td>GO Component</td><td>intracellular organelle</td><td>RCN1|ARHGEF5|MAP2K5|PLOD1|MYH9|CYP24A1|MMP2|EHD4|GAPDH|RBP1|PPP3R1|ENO1|MT2A|CDC25B|HSPB1|BCL2L2|PXDN|ARF3|PDGFRA|THBS1|COL8A1|VWF|RORA|DNAJB6|CNN2|PRKCG|AKT3|TUBB4A|CAD|ADD1|FERMT3|TJP1|FLT1|PLOD2|APP|CSF1R|KIT|NPC1L1|CCR5|CAPN2|ITGA2|NOS3|MELK|CKB|CCT2|PLK1|TUBA1A|TUBA1C|GLOD4|DDB1|BCL2L1|CDC25A|SDPR|ARF4|MMP14|BRSK1|DAB2|CALR|CDC25C|ZYX|RORC|FLNC|RARB|CYP2R1|NR1I2|LCK|SNCA|CAV1|RGS12|TUBB4B|PTGES|USP1|PTMA|TPM4|ANXA2|ANXA6|FYN|FN1|FAS|PTRF|SFPQ|MYO1C|RXRG|MYH10|DPP4|TGM2|DLL1|ARHGAP30|COPA|TPM3|FLNA|CNN3|PTPN1|SDC4|SPTAN1|DNM1|TLR4|CLIC4|RXRB|ANXA1|TPM2|PIR|PSIP1|SDC1|FSCN1|XRCC5|MYO1B|G6PD|PPP3CA|CAMK2B|BCL2|CAMK2A|DYRK1A|SEPT7|CDC42|SUN1|PDIA6|RARG|TOP2B|WEE1|FLNB|PBK|SPTBN2|CFL1|ARF1|VIM|NR1H4|NUMB|APBA2|TLN2|PRKCSH|CAMK1D|MCM3</td><td>2.11E-06</td><td>GO.0043229</td></tr>
<tr><td>8</td><td>GO Component</td><td>actin filament</td><td>TPM4|FYN|MYO1C|TPM3|FLNA|TPM2|FSCN1|MYO1B</td><td>2.41E-06</td><td>GO.0005884</td></tr>
<tr><td>31</td><td>GO Component</td><td>plasma membrane bounded cell projection part</td><td>PLEK|HSPB1|PRKCG|NGEF|TUBB4A|CAD|APP|NPC1L1|CAPN2|ITGA2|CKB|ARF4|BRSK1|NQO1|SNCA|RGS12|FYN|EPHA2|MYO1C|MYH10|DPP4|ARHGEF15|FLNA|CNN3|DNM1|FSCN1|PPP3CA|DYRK1A|SEPT7|CDC42|CFL1</td><td>2.41E-06</td><td>GO.0120038</td></tr>
</table>

<table>
<tr><th></th><th></th><th></th><th></th><th></th><th></th></tr>
<tr><td>109</td><td>GO Component</td><td>organelle part</td><td>RCN1|ARHGEF5|MAP2K5|PLOD1|MYH9|CYP24A1|MMP2|EHD4|GAPDH|RBP1|PPP3R1|ENO1|CDC25B|HSPB1|BCL2L2|ARF3|THBS1|COL8A1|VWF|RORA|DNAJB6|CNN2|TUBB4A|CAD|ADD1|FERMT3|TJP1|PLOD2|APP|CSF1R|NPC1L1|CAPN2|NOS3|CCT2|PLK1|TUBA1A|TUBA1C|DDB1|BCL2L1|CDC25A|SDPR|MMP14|BRSK1|DAB2|CALR|CDC25C|ZYX|RORC|FLNC|RARB|CYP2R1|NR1I2|LCK|SNCA|CAV1|RGS12|TUBB4B|PTGES|USP1|PTMA|TPM4|ANXA2|ANXA6|FYN|FN1|FAS|PTRF|SFPQ|MYO1C|RXRG|MYH10|COPA|TPM3|FLNA|PTPN1|SDC4|SPTAN1|DNM1|TLR4|CLIC4|RXRB|ANXA1|TPM2|PIR|PSIP1|SDC1|FSCN1|XRCC5|MYO1B|PPP3CA|CAMK2B|BCL2|CAMK2A|DYRK1A|SEPT7|CDC42|SUN1|PDIA6|RARG|TOP2B|WEE1|FLNB|SPTBN2|CFL1|ARF1|VIM|NR1H4|PRKCSH|MCM3</td><td>2.82E-06</td><td>GO.0044422</td></tr>
<tr><td>34</td><td>GO Component</td><td>whole membrane</td><td>CYP24A1|EHD4|BCL2L2|ICAM1|APP|CAPN2|NOS3|TUBA1A|BCL2L1|SDPR|DAB2|CALR|LCK|SNCA|CAV1|ANXA2|ANXA6|FYN|FAS|PTRF|MYO1C|DPP4|DLL1|COPA|SDC4|TLR4|ANXA1|MYO1B|CAMK2B|LDHB|LTB4R|BCL2|CAMK2A|SUN1</td><td>2.82E-06</td><td>GO.0098805</td></tr>
<tr><td>12</td><td>GO Component</td><td>contractile fiber part</td><td>MMP2|ENO1|HSPB1|DNAJB6|FLNC|TPM4|TPM3|FLNA|SDC4|TPM2|PPP3CA|FLNB</td><td>4.28E-06</td><td>GO.0044449</td></tr>
<tr><td>24</td><td>GO Component</td><td>supramolecular fiber</td><td>MMP2|ENO1|HSPB1|DNAJB6|TUBB4A|CCT2|TUBA1A|TUBA1C|FLNC|SNCA|TUBB4B|TPM4|FYN|MYO1C|TPM3|FLNA|SDC4|DNM1|TPM2|FSCN1|MYO1B|PPP3CA|FLNB|VIM</td><td>4.39E-06</td><td>GO.0099512</td></tr>
<tr><td>16</td><td>GO Component</td><td>cell-cell junction</td><td>MYH9|CNN2|PRKCG|ADD1|TJP1|APP|KIT|ZYX|PRKCH|CDH5|DPP4|FLNA|CLIC4|FSCN1|PPP3CA|CFL1</td><td>4.54E-06</td><td>GO.0005911</td></tr>
<tr><td>12</td><td>GO Component</td><td>myofibril</td><td>MMP2|ENO1|HSPB1|DNAJB6|FLNC|TPM4|TPM3|FLNA|SDC4|TPM2|PPP3CA|FLNB</td><td>4.85E-06</td><td>GO.0030016</td></tr>
<tr><td>32</td><td>GO Component</td><td>neuron part</td><td>HSPB1|PRKCG|NGEF|TUBB4A|CAD|APP|CAPN2|ITGA2|CKB|BCL2L1|ARF4|BRSK1|NQO1|SNCA|RGS12|FYN|MYO1C|MYH10|ARHGEF15|FLNA|CNN3|DNM1|FSCN1|PPP3CA|CAMK2B|CAMK2A|DYRK1A|CDC42|SPTBN2|ARF1|VIM|APBA2</td><td>4.96E-06</td><td>GO.0097458</td></tr>
<tr><td>106</td><td>GO Component</td><td>intracellular organelle part</td><td>RCN1|ARHGEF5|MAP2K5|PLOD1|MYH9|CYP24A1|EHD4|GAPDH|RBP1|PPP3R1|CDC25B|HSPB1|BCL2L2|ARF3|THBS1|COL8A1|VWF|RORA|DNAJB6|CNN2|TUBB4A|CAD|ADD1|FERMT3|TJP1|PLOD2|APP|CSF1R|NPC1L1|CAPN2|NOS3|CCT2|PLK1|TUBA1A|TUBA1C|DDB1|BCL2L1|CDC25A|SDPR|MMP14|BRSK1|DAB2|CALR|CDC25C|ZYX|RORC|RARB|CYP2R1|NR1I2|LCK|SNCA|CAV1|RGS12|TUBB4B|PTGES|USP1|PTMA|TPM4|ANXA2|ANXA6|FYN|FN1|FAS|PTRF|SFPQ|MYO1C|RXRG|MYH10|COPA|TPM3|FLNA|PTPN1|SDC4|SPTAN1|DNM1|TLR4|CLIC4|RXRB|ANXA1|TPM2|PIR|PSIP1|SDC1|FSCN1|XRCC5|MYO1B|PPP3CA|CAMK2B|BCL2|CAMK2A|DYRK1A|SEPT7|CDC42|SUN1|PDIA6|RARG|TOP2B|WEE1|FLNB|SPTBN2|CFL1|ARF1|VIM|NR1H4|PRKCSH|MCM3</td><td>6.03E-06</td><td>GO.0044446</td></tr>
<tr><td>12</td><td>GO Component</td><td>external side of plasma membrane</td><td>PDGFRA|THBS1|ICAM1|KIT|CCR5|ANXA5|ITGA2|CALR|CDH5|TLR4|ANXA1|SDC1</td><td>6.30E-06</td><td>GO.0009897</td></tr>
<tr><td>26</td><td>GO Component</td><td>plasma membrane region</td><td>MYH9|PLEK|PRKCG|TJP1|NPC1L1|NOS3|SDPR|ARF4|CAV1|ANXA2|FN1|PTRF|EPHA2|MYO1C|MYH10|DPP4|DLL1|ANXA1|FSCN1|CAMK2A|SEPT7|SPTBN2|CFL1|ARF1|NUMB|ABCB1</td><td>1.00E-05</td><td>GO.0098590</td></tr>
<tr><td>16</td><td>GO Component</td><td>side of membrane</td><td>PDGFRA|THBS1|ICAM1|KIT|CCR5|ANXA5|ITGA2|CALR|LCK|CDH5|FYN|PTPN1|TLR4|ANXA1|SDC1|G6PD</td><td>1.11E-05</td><td>GO.0098552</td></tr>
<tr><td>11</td><td>GO Component</td><td>sarcomere</td><td>MMP2|ENO1|HSPB1|DNAJB6|FLNC|TPM4|TPM3|FLNA|TPM2|PPP3CA|FLNB</td><td>1.12E-05</td><td>GO.0030017</td></tr>
<tr><td>10</td><td>GO Component</td><td>ruffle</td><td>MYH9|PLEK|ARF4|ANXA2|ARHGEF26|EPHA2|MYO1C|FSCN1|CFL1|TLN2</td><td>1.76E-05</td><td>GO.0001726</td></tr>
<tr><td>123</td><td>GO Component</td><td>membrane-bounded organelle</td><td>RCN1|ARHGEF5|MAP2K5|PLOD1|MYH9|CYP24A1|MMP2|EHD4|GAPDH|RBP1|PPP3R1|ENO1|MT2A|CDC25B|HSPB1|BCL2L2|PXDN|ARF3|PDGFRA|THBS1|COL8A1|VWF|RORA|DNAJB6|CNN2|PRKCG|AKT3|CAD|ADD1|ICAM1|FERMT3|FLT1|PLOD2|APP|CSF1R|KIT|NPC1L1|CCR5|CAPN2|ITGA2|NOS3|MELK|CKB|CCT2|PLK1|TUBA1A|TUBA1C|GLOD4|DDB1|BCL2L1|CDC25A|SDPR|ARF4|MMP14|BRSK1|DAB2|CALR|CDC25C|ZYX|RORC|RARB|CYP2R1|NR1I2|SNCA|CAV1|RGS12|TUBB4B|PTGES|USP1|PTMA|ANXA2|ANXA6|FYN|FN1|FAS|PTRF|SFPQ|MYO1C|RXRG|DPP4|TGM2|DLL1|ARHGAP30|COPA|FLNA|PTPN1|SDC4|SPTAN1|DNM1|TLR4|CLIC4|RXRB|ANXA1|TBC1D4|PIR|PSIP1|SDC1|XRCC5|MYO1B|G6PD|PPP3CA|CAMK2B|BCL2|CAMK2A|DYRK1A|SEPT7|CDC42|SUN1|PDIA6|RARG|TOP2B|WEE1|FLNB|PBK|CFL1|ARF1|VIM|NR1H4|NUMB|APBA2|PRKCSH|CAMK1D|MCM3</td><td>2.08E-05</td><td>GO.0043227</td></tr>
<tr><td>100</td><td>GO Component</td><td>membrane</td><td>ARHGEF5|PLOD1|ITGB4|MYH9|CYP24A1|MMP2|EHD4|APLP1|GAPDH|PPP3R1|PLEK|ENO1|HSPB1|BCL2L2|ARF3|PDGFRA|THBS1|PRKCG|AKT3|NGEF|ADD1|ICAM1|TJP1|FLT1|PLOD2|APP|CSF1R|KIT|NPC1L1|CCR5|CAPN2|ANXA5|ITGA2|NOS3|MELK|TUBA1A|BCL2L1|SDPR|ARF4|MMP14|DAB2|TENC1|CALR|STXBP6|FLNC|JAG2|PRKCH|CYP2R1|LCK|SNCA|CAV1|RGS12|PTGES|CDH5|ANXA2|ANXA6|FYN|FN1|FAS|PTRF|EPHA2|MYO1C|MYH10|DPP4|TGM2|DLL1|COPA|FLNA|PTPN1|SDC4|SPTAN1|DNM1|TLR4|CLIC4|ANXA1|SDC1|FSCN1|XRCC5|MYO1B|G6PD|PPP3CA|CAMK2B|LDHB|LTB4R|BCL2|CAMK2A|SEPT7|CDC42|SUN1|PDIA6|RARG|FLNB|SPTBN2|CFL1|ARF1|VIM|NUMB|APBA2|TLN2|ABCB1</td><td>2.68E-05</td><td>GO.0016020</td></tr>
<tr><td>22</td><td>GO Component</td><td>synapse</td><td>MYH9|LAMA5|PRKCG|CAD|APP|ITGA2|BCL2L1|ARF4|BRSK1|SNCA|RGS12|FYN|MYH10|CNN3|DNM1|PPP3CA|CAMK2B|CAMK2A|CDC42|ARF1|APBA2|TLN2</td><td>2.80E-05</td><td>GO.0045202</td></tr>
<tr><td>12</td><td>GO Component</td><td>extracellular matrix</td><td>APLP1|LAMA4|PXDN|LAMA5|THBS1|COL8A1|VWF|MMP14|CALR|ANXA2|FN1|MMRN2</td><td>5.69E-05</td><td>GO.0031012</td></tr>
<tr><td>69</td><td>GO Component</td><td>intracellular organelle lumen</td><td>RCN1|ARHGEF5|CYP24A1|RBP1|PPP3R1|CDC25B|THBS1|COL8A1|VWF|RORA|DNAJB6|CNN2|CAD|ADD1|FERMT3|APP|CSF1R|CCT2|PLK1|DDB1|BCL2L1|CDC25A|SDPR|MMP14|BRSK1|DAB2|CALR|CDC25C|RORC|RARB|NR1I2|SNCA|RGS12|TUBB4B|PTGES|USP1|PTMA|ANXA2|FN1|FAS|PTRF|SFPQ|MYO1C|RXRG|FLNA|SDC4|SPTAN1|DNM1|CLIC4|RXRB|ANXA1|PIR|PSIP1|SDC1|XRCC5|PPP3CA|CAMK2B|BCL2|CAMK2A|DYRK1A|PDIA6|RARG|TOP2B|WEE1|CFL1|VIM|NR1H4|PRKCSH|MCM3</td><td>8.54E-05</td><td>GO.0070013</td></tr>
<tr><td>29</td><td>GO Component</td><td>cytoplasmic vesicle part</td><td>EHD4|THBS1|VWF|CNN2|FERMT3|APP|NPC1L1|NOS3|CCT2|BCL2L1|DAB2|CALR|SNCA|CAV1|TUBB4B|ANXA2|ANXA6|FN1|MYO1C|COPA|SPTAN1|TLR4|CLIC4|ANXA1|XRCC5|MYO1B|CAMK2B|CAMK2A|SUN1</td><td>9.14E-05</td><td>GO.0044433</td></tr>
<tr><td>11</td><td>GO Component</td><td>adherens junction</td><td>MYH9|ARHGAP31|ADD1|TJP1|ITGA2|TENC1|ZYX|CDH5|EPHA2|DLL1|SDC4</td><td>9.90E-05</td><td>GO.0005912</td></tr>
<tr><td>19</td><td>GO Component</td><td>somatodendritic compartment</td><td>PRKCG|TUBB4A|CAD|APP|CAPN2|CKB|ARF4|NQO1|SNCA|RGS12|FYN|MYH10|ARHGEF15|FLNA|CNN3|PPP3CA|DYRK1A|CDC42|SPTBN2</td><td>1.20E-04</td><td>GO.0036477</td></tr>
<tr><td>7</td><td>GO Component</td><td>basement membrane</td><td>APLP1|LAMA4|LAMA5|COL8A1|ANXA2|FN1|MMRN2</td><td>1.30E-04</td><td>GO.0005604</td></tr>
<tr><td>3</td><td>GO Component</td><td>muscle thin filament tropomyosin</td><td>TPM4|TPM3|TPM2</td><td>1.40E-04</td><td>GO.0005862</td></tr>
<tr><td>13</td><td>GO Component</td><td>apical part of cell</td><td>TJP1|APP|NPC1L1|CAV1|FN1|DPP4|DLL1|CLIC4|ANXA1|MYO1B|SPTBN2|NUMB|ABCB1</td><td>1.40E-04</td><td>GO.0045177</td></tr>
<tr><td>18</td><td>GO Component</td><td>synapse part</td><td>LAMA5|PRKCG|CAD|APP|ITGA2|BCL2L1|ARF4|BRSK1|SNCA|FYN|MYH10|CNN3|DNM1|PPP3CA|CAMK2A|CDC42|ARF1|APBA2</td><td>2.40E-04</td><td>GO.0044456</td></tr>
<tr><td>12</td><td>GO Component</td><td>cytoplasmic vesicle lumen</td><td>THBS1|VWF|CNN2|FERMT3|APP|CCT2|CALR|TUBB4B|ANXA2|FN1|SPTAN1|XRCC5</td><td>2.70E-04</td><td>GO.0060205</td></tr>
<tr><td>8</td><td>GO Component</td><td>cell-substrate junction</td><td>ITGB4|MYH9|ARHGAP31|ITGA2|TENC1|ZYX|EPHA2|SDC4</td><td>2.80E-04</td><td>GO.0030055</td></tr>
<tr><td>15</td><td>GO Component</td><td>dendrite</td><td>PRKCG|APP|CAPN2|CKB|ARF4|NQO1|RGS12|FYN|MYH10|ARHGEF15|FLNA|CNN3|PPP3CA|DYRK1A|CDC42</td><td>3.50E-04</td><td>GO.0030425</td></tr>
<tr><td>6</td><td>GO Component</td><td>cortical cytoskeleton</td><td>MYH9|CAPN2|FLNA|SPTAN1|SPTBN2|CFL1</td><td>4.30E-04</td><td>GO.0030863</td></tr>
<tr><td>23</td><td>GO Component</td><td>microtubule cytoskeleton</td><td>MAP2K5|MYH9|GAPDH|CDC25B|HSPB1|TUBB4A|APP|CCT2|PLK1|TUBA1A|TUBA1C|BCL2L1|BRSK1|LCK|TUBB4B|MYH10|SPTAN1|DNM1|CLIC4|CAMK2B|SEPT7|CDC42|MCM3</td><td>4.60E-04</td><td>GO.0015630</td></tr>
<tr><td>58</td><td>GO Component</td><td>endomembrane system</td><td>RCN1|PLOD1|EHD4|GAPDH|PXDN|ARF3|THBS1|COL8A1|VWF|CNN2|FERMT3|FLT1|PLOD2|APP|KIT|CCR5|CAPN2|NOS3|CCT2|TUBA1A|BCL2L1|ARF4|MMP14|BRSK1|DAB2|CALR|CYP2R1|SNCA|CAV1|TUBB4B|PTGES|ANXA2|ANXA6|FYN|FN1|PTRF|MYO1C|TGM2|COPA|PTPN1|SDC4|SPTAN1|DNM1|TLR4|ANXA1|TBC1D4|SDC1|XRCC5|MYO1B|CAMK2B|BCL2|CDC42|SUN1|PDIA6|ARF1|NUMB|APBA2|PRKCSH</td><td>5.70E-04</td><td>GO.0012505</td></tr>
<tr><td>11</td><td>GO Component</td><td>secretory granule lumen</td><td>THBS1|VWF|CNN2|FERMT3|APP|CCT2|TUBB4B|ANXA2|FN1|SPTAN1|XRCC5</td><td>7.10E-04</td><td>GO.0034774</td></tr>
</table>

5	GO Component	cortical actin cytoskeleton	MYH9|CAPN2|SPTAN1|SPTBN2|CFL1	7.30E-04	GO.0030864
10	GO Component	endocytic vesicle	NOS3|CALR|ZYX|CAV1|MYO1C|DPP4|CAMK2B|CAMK2A|FLNB|VIM	8.50E-04	GO.0030139
20	GO Component	secretory vesicle	THBS1|VWF|CNN2|FERMT3|APP|KIT|CCT2|BCL2L1|BRSK1|CALR|SNCA|CAV1|TUBB4B|ANXA2|FN1|SPTAN1|DNM1|XRCC5|SUN1|APBA2	9.60E-04	GO.0099503
4	GO Component	podosome	ARHGEF5|FERMT3|TJP1|FSCN1	0.001	GO.0002102
7	GO Component	focal adhesion	MYH9|ARHGAP31|ITGA2|TENC1|ZYX|EPHA2|SDC4	0.001	GO.0005925
11	GO Component	axon part	HSPB1|NGEF|TUBB4A|CAD|APP|ITGA2|BRSK1|SNCA|MYH10|DNM1|FSCN1	0.001	GO.0033267
7	GO Component	nuclear periphery	CAD|RGS12|SFPQ|CLIC4|PSIP1|CFL1|VIM	0.001	GO.0034399
6	GO Component	brush border	MYH9|NPC1L1|MYO1C|MYH10|MYO1B|FLNB	0.0012	GO.0005903
61	GO Component	protein-containing complex	ARHGEF5|PLOD1|ITGB4|MYH9|GAPDH|PPP3R1|ENO1|HSPB1|BCL2L2|LAMA5|PDGFRA|THBS1|COL8A1|CAD|ADD1|EXOC6B|FERMT3|TJP1|FLT1|APP|CSF1R|ITGA2|CCT2|PLK1|TUBA1A|DDB1|BCL2L1|CALR|STXBP6|SNCA|CAV1|ANXA2|FN1|FAS|PTRF|SFPQ|MYO1C|MYH10|COPA|FLNA|DNM1|TLR4|CLIC4|ANXA1|SDC1|FSCN1|XRCC5|MYO1B|PPP3CA|BCL2|DYRK1A|SEPT7|CDC42|SUN1|PDIA6|RARG|TOP2B|ARF1|VIM|PRKCSH|MCM3	0.0012	GO.0032991
109	GO Component	intracellular membrane-bounded organelle	RCN1|ARHGEF5|MAP2K5|PLOD1|MYH9|CYP24A1|MMP2|EHD4|GAPDH|RBP1|PPP3R1|ENO1|MT2A|CDC25B|HSPB1|BCL2L2|PXDN|ARF3|PDGFRA|THBS1|COL8A1|VWF|RORA|DNAJB6|PRKCG|AKT3|CAD|ADD1|PLOD2|APP|CSF1R|KIT|CAPN2|ITGA2|NOS3|MELK|CKB|CCT2|PLK1|TUBA1C|GLOD4|DDB1|BCL2L1|CDC25A|SDPR|ARF4|MMP14|BRSK1|DAB2|CALR|CDC25C|ZYX|RORC|RARB|CYP2R1|NR1I2|SNCA|CAV1|RGS12|TUBB4B|PTGES|USP1|PTMA|ANXA2|ANXA6|FYN|FN1|FAS|PTRF|SFPQ|MYO1C|RXRG|TGM2|ARHGAP30|COPA|FLNA|PTPN1|SDC4|SPTAN1|DNM1|CLIC4|RXRB|ANXA1|PIR|PSIP1|SDC1|XRCC5|G6PD|PPP3CA|CAMK2B|BCL2|CAMK2A|DYRK1A|SEPT7|CDC42|SUN1|PDIA6|RARG|TOP2B|WEE1|PBK|CFL1|ARF1|VIM|NR1H4|NUMB|PRKCSH|CAMK1D|MCM3	0.0018	GO.0043231
6	GO Component	nuclear matrix	CAD|RGS12|SFPQ|CLIC4|CFL1|VIM	0.0019	GO.0016363
5	GO Component	platelet alpha granule lumen	THBS1|VWF|FERMT3|APP|FN1	0.0019	GO.0031093
15	GO Component	polymeric cytoskeletal fiber	TUBB4A|CCT2|TUBA1A|TUBA1C|TUBB4B|TPM4|FYN|MYO1C|TPM3|FLNA|DNM1|TPM2|FSCN1|MYO1B|VIM	0.0022	GO.0099513
79	GO Component	nucleus	ARHGEF5|MAP2K5|MYH9|CYP24A1|MMP2|EHD4|GAPDH|RBP1|PPP3R1|ENO1|MT2A|CDC25B|HSPB1|PDGFRA|RORA|DNAJB6|PRKCG|AKT3|CAD|ADD1|APP|CSF1R|CAPN2|ITGA2|NOS3|MELK|CKB|PLK1|TUBA1C|DDB1|BCL2L1|CDC25A|SDPR|MMP14|BRSK1|DAB2|CALR|CDC25C|ZYX|RORC|RARB|NR1I2|SNCA|RGS12|PTGES|USP1|PTMA|FYN|FAS|PTRF|SFPQ|MYO1C|RXRG|FLNA|DNM1|CLIC4|RXRB|ANXA1|PIR|PSIP1|XRCC5|G6PD|PPP3CA|CAMK2B|BCL2|CAMK2A|DYRK1A|SEPT7|SUN1|RARG|TOP2B|WEE1|PBK|CFL1|VIM|NR1H4|NUMB|CAMK1D|MCM3	0.0024	GO.0005634
5	GO Component	cell-cell adherens junction	MYH9|ADD1|TJP1|ZYX|CDH5	0.0024	GO.0005913
24	GO Component	extracellular region part	MMP2|APLP1|CCL2|LAMA4|PXDN|LAMA5|THBS1|COL8A1|VWF|ICAM1|FLT1|APP|KIT|ANXA5|CKB|MMP14|CALR|SNCA|ANXA2|FN1|COPA|MMRN2|ANXA1|PDIA6	0.0028	GO.0044421
55	GO Component	nuclear part	ARHGEF5|CYP24A1|GAPDH|RBP1|PPP3R1|CDC25B|RORA|DNAJB6|CAD|ADD1|APP|CSF1R|PLK1|DDB1|BCL2L1|CDC25A|SDPR|BRSK1|DAB2|CALR|CDC25C|RORC|RARB|NR1I2|SNCA|RGS12|PTGES|USP1|PTMA|FAS|PTRF|SFPQ|MYO1C|RXRG|FLNA|DNM1|CLIC4|RXRB|ANXA1|PIR|PSIP1|XRCC5|PPP3CA|CAMK2B|BCL2|CAMK2A|DYRK1A|SUN1|RARG|TOP2B|WEE1|CFL1|VIM|NR1H4|MCM3	0.0033	GO.0044428
2	GO Component	myosin II filament	MYH9|MYH10	0.0034	GO.0097513
6	GO Component	Z disc	HSPB1|DNAJB6|FLNC|FLNA|PPP3CA|FLNB	0.0035	GO.0030018
36	GO Component	extracellular region	MMP2|APLP1|CCL2|LAMA4|PLEK|PXDN|LAMA5|THBS1|COL8A1|VWF|CNN2|ICAM1|FERMT3|FLT1|APP|KIT|ANXA5|CKB|CCT2|MMP14|CALR|SNCA|TUBB4B|ANXA2|FN1|FAS|DPP4|DLL1|COPA|FLNA|MMRN2|SDC4|SPTAN1|ANXA1|XRCC5|PDIA6	0.0041	GO.0005576
2	GO Component	calcineurin complex	PPP3R1|PPP3CA	0.0049	GO.0005955
5	GO Component	ruffle membrane	PLEK|ARF4|EPHA2|MYO1C|CFL1	0.0049	GO.0032587
7	GO Component	lamellipodium	ARHGAP31|APP|EPHA2|MYH10|DPP4|FSCN1|CFL1	0.0054	GO.0030027
3	GO Component	lamellipodium membrane	EPHA2|DPP4|CFL1	0.0054	GO.0031258
11	GO Component	postsynapse	PRKCG|APP|ARF4|SNCA|FYN|MYH10|CNN3|PPP3CA|CAMK2A|CDC42|ARF1	0.0058	GO.0098794
2	GO Component	macropinosome	MMP14|ANXA2	0.0063	GO.0044354
7	GO Component	actin-based cell projection	PDGFRA|APP|MYO1C|CLIC4|FSCN1|MYO1B|CDC42	0.0063	GO.0098858
8	GO Component	distal axon	NGEF|CAD|APP|ITGA2|BRSK1|SNCA|MYH10|FSCN1	0.0063	GO.0150034
20	GO Component	extracellular space	MMP2|CCL2|PXDN|LAMA5|THBS1|ICAM1|FLT1|APP|KIT|ANXA5|CKB|MMP14|CALR|SNCA|ANXA2|FN1|COPA|MMRN2|ANXA1|PDIA6	0.0064	GO.0005615
6	GO Component	dendritic spine	APP|ARF4|MYH10|CNN3|PPP3CA|CDC42	0.0067	GO.0043197
9	GO Component	spindle	MAP2K5|MYH9|CDC25B|HSPB1|APP|PLK1|MYH10|SEPT7|CDC42	0.0076	GO.0005819
12	GO Component	cell body	TUBB4A|CAD|CKB|CCT2|NQO1|SNCA|FYN|MYH10|FLNA|CNN3|CDC42|SPTBN2	0.0076	GO.0044297
4	GO Component	extracellular matrix component	LAMA4|LAMA5|COL8A1|FN1	0.0076	GO.0044420
73	GO Component	membrane part	PLOD1|ITGB4|MYH9|APLP1|PLEK|PDGFRA|THBS1|PRKCG|ICAM1|TJP1|FLT1|PLOD2|APP|CSF1R|KIT|NPC1L1|CCR5|CAPN2|ANXA5|ITGA2|NOS3|TUBA1A|BCL2L1|SDPR|ARF4|MMP14|DAB2|CALR|STXBP6|JAG2|CYP2R1|LCK|SNCA|CAV1|PTGES|CDH5|ANXA2|FYN|FN1|FAS|PTRF|EPHA2|MYO1C|MYH10|DPP4|TGM2|DLL1|COPA|PTPN1|SDC4|DNM1|TLR4|CLIC4|ANXA1|SDC1|FSCN1|G6PD|CAMK2B|LDHB|LTB4R|BCL2|CAMK2A|SEPT7|CDC42|SUN1|PDIA6|RARG|FLNB|SPTBN2|CFL1|ARF1|NUMB|ABCB1	0.0077	GO.0044425
12	GO Component	axon	HSPB1|NGEF|TUBB4A|CAD|APP|ITGA2|BRSK1|SNCA|MYH10|DNM1|FSCN1|DYRK1A	0.0078	GO.0030424
6	GO Component	leading edge membrane	PLEK|ARF4|EPHA2|MYO1C|DPP4|CFL1	0.0078	GO.0031256
27	GO Component	endoplasmic reticulum	RCN1|PLOD1|EHD4|PXDN|THBS1|COL8A1|VWF|PLOD2|APP|CAPN2|BCL2L1|CALR|CYP2R1|SNCA|CAV1|PTGES|FYN|FN1|PTRF|TGM2|COPA|PTPN1|CAMK2B|BCL2|CDC42|PDIA6|PRKCSH	0.0088	GO.0005783
2	GO Component	Bcl-2 family protein complex	BCL2L2|BCL2L1	0.0093	GO.0097136
9	GO Component	early endosome	EHD4|APP|CAV1|ANXA2|PTPN1|TLR4|ANXA1|MYO1B|NUMB	0.0104	GO.0005769
3	GO Component	immunological synapse	MYH9|ICAM1|LCK	0.0108	GO.0001772
3	GO Component	spindle midzone	APP|PLK1|CDC42	0.0108	GO.0051233
2	GO Component	fibrinogen complex	THBS1|FN1	0.0112	GO.0005577
2	GO Component	nuclear envelope lumen	APP|PTGES	0.0112	GO.0005641
2	GO Component	spectrin	SPTAN1|SPTBN2	0.0112	GO.0008091
6	GO Component	midbody	PLK1|ANXA2|MYH10|CLIC4|SEPT7|CDC42	0.0115	GO.0030496
4	GO Component	myosin complex	MYH9|MYO1C|MYH10|MYO1B	0.0118	GO.0016459
4	GO Component	sarcoplasm	THBS1|CALR|FLNC|CAMK2B	0.0123	GO.0016528
14	GO Component	cytoplasmic vesicle membrane	NPC1L1|NOS3|BCL2L1|DAB2|CALR|SNCA|CAV1|MYO1C|COPA|CLIC4|ANXA1|CAMK2B|CAMK2A|SUN1	0.0127	GO.0030659
6	GO Component	exocytic vesicle	APP|BCL2L1|BRSK1|SNCA|DNM1|APBA2	0.0136	GO.0070382
4	GO Component	rough endoplasmic reticulum	PLOD1|PLOD2|APP|SNCA	0.0146	GO.0005791
4	GO Component	lipid droplet	GAPDH|RBP1|CAV1|ANXA2	0.0146	GO.0005811
4	GO Component	caveola	NOS3|SDPR|CAV1|PTRF	0.0146	GO.0005901
8	GO Component	endoplasmic reticulum lumen	RCN1|THBS1|COL8A1|APP|CALR|FN1|PDIA6|PRKCSH	0.0147	GO.0005788
5	GO Component	sarcolemma	PPP3R1|FLNC|ANXA2|ANXA1|PPP3CA	0.0147	GO.0042383
5	GO Component	phagocytic vesicle	CALR|ZYX|MYO1C|FLNB|VIM	0.0147	GO.0045335
15	GO Component	secretory granule	THBS1|VWF|CNN2|FERMT3|APP|KIT|CCT2|CALR|CAV1|TUBB4B|ANXA2|FN1|SPTAN1|XRCC5|SUN1	0.0156	GO.0030141
8	GO Component	apical plasma membrane	NPC1L1|CAV1|FN1|DPP4|DLL1|ANXA1|SPTBN2|ABCB1	0.0167	GO.0016324

<table>
<tr><td>4</td><td>GO Component</td><td>extracellular exosome</td><td>ICAM1|ANXA2|FN1|ANXA1</td><td>0.0178</td><td>GO.0070062</td></tr>
<tr><td>48</td><td>GO Component</td><td>nuclear lumen</td><td>ARHGEF5|CYP24A1|RBP1|PPP3R1|CDC25B|RORA|DNAJB6|CAD|ADD1|CSF1R|PLK1|DDB1|CDC25A|SDPR|BRSK1|DAB2|CDC25C|RORC|RARB|NR1I2|RGS12|USP1|PTMA|FAS|PTRF|SFPQ|MYO1C|RXRG|FLNA|DNM1|CLIC4|RXRB|ANXA1|PIR|PSIP1|XRCC5|PPP3CA|CAMK2B|BCL2|CAMK2A|DYRK1A|RARG|TOP2B|WEE1|CFL1|VIM|NR1H4|MCM3</td><td>0.0184</td><td>GO.0031981</td></tr>
<tr><td>4</td><td>GO Component</td><td>microvillus</td><td>PDGFRA|MYO1C|CLIC4|FSCN1</td><td>0.0191</td><td>GO.0005902</td></tr>
<tr><td>8</td><td>GO Component</td><td>cell projection membrane</td><td>PLEK|NPC1L1|ARF4|EPHA2|MYO1C|DPP4|FSCN1|CFL1</td><td>0.0195</td><td>GO.0031253</td></tr>
<tr><td>10</td><td>GO Component</td><td>neuronal cell body</td><td>TUBB4A|CAD|CKB|NQO1|SNCA|MYH10|FLNA|CNN3|CDC42|SPTBN2</td><td>0.0198</td><td>GO.0043025</td></tr>
<tr><td>20</td><td>GO Component</td><td>endoplasmic reticulum part</td><td>RCN1|PLOD1|THBS1|COL8A1|PLOD2|APP|CAPN2|CALR|CYP2R1|CAV1|PTGES|FYN|FN1|COPA|PTPN1|CAMK2B|BCL2|CDC42|PDIA6|PRKCSH</td><td>0.0208</td><td>GO.0044432</td></tr>
<tr><td>42</td><td>GO Component</td><td>nucleoplasm</td><td>ARHGEF5|CYP24A1|RBP1|PPP3R1|CDC25B|RORA|DNAJB6|CAD|ADD1|CSF1R|PLK1|DDB1|CDC25A|SDPR|BRSK1|CDC25C|RORC|RARB|NR1I2|USP1|PTMA|FAS|PTRF|SFPQ|MYO1C|RXRG|DNM1|RXRB|ANXA1|PIR|PSIP1|XRCC5|PPP3CA|CAMK2B|BCL2|CAMK2A|DYRK1A|RARG|TOP2B|WEE1|NR1H4|MCM3</td><td>0.0218</td><td>GO.0005654</td></tr>
<tr><td>15</td><td>GO Component</td><td>endosome</td><td>EHD4|FLT1|APP|CCR5|TUBA1A|MMP14|CAV1|ANXA2|ANXA6|FYN|PTPN1|TLR4|ANXA1|MYO1B|NUMB</td><td>0.024</td><td>GO.0005768</td></tr>
<tr><td>5</td><td>GO Component</td><td>extrinsic component of plasma membrane</td><td>LCK|ANXA2|FYN|ANXA1|NUMB</td><td>0.0267</td><td>GO.0019897</td></tr>
<tr><td>2</td><td>GO Component</td><td>perinuclear endoplasmic reticulum</td><td>CAPN2|FYN</td><td>0.0271</td><td>GO.0097038</td></tr>
<tr><td>2</td><td>GO Component</td><td>exocyst</td><td>EXOC6B|STXBP6</td><td>0.0298</td><td>GO.0000145</td></tr>
<tr><td>4</td><td>GO Component</td><td>filopodium</td><td>APP|FSCN1|MYO1B|CDC42</td><td>0.0298</td><td>GO.0030175</td></tr>
<tr><td>2</td><td>GO Component</td><td>filamentous actin</td><td>MYO1C|FSCN1</td><td>0.0298</td><td>GO.0031941</td></tr>
<tr><td>2</td><td>GO Component</td><td>invadopodium</td><td>DPP4|FSCN1</td><td>0.0298</td><td>GO.0071437</td></tr>
<tr><td>6</td><td>GO Component</td><td>basolateral plasma membrane</td><td>TJP1|CAV1|ANXA2|MYO1C|ANXA1|NUMB</td><td>0.0305</td><td>GO.0016323</td></tr>
<tr><td>5</td><td>GO Component</td><td>endocytic vesicle membrane</td><td>NOS3|CALR|CAV1|CAMK2B|CAMK2A</td><td>0.0305</td><td>GO.0030666</td></tr>
<tr><td>11</td><td>GO Component</td><td>lysosome</td><td>KIT|CAPN2|CCT2|DAB2|SNCA|TUBB4B|ANXA2|ANXA6|SDC4|ANXA1|SDC1</td><td>0.0322</td><td>GO.0005764</td></tr>
<tr><td>5</td><td>GO Component</td><td>synaptic vesicle</td><td>BCL2L1|BRSK1|SNCA|DNM1|APBA2</td><td>0.0322</td><td>GO.0008021</td></tr>
<tr><td>3</td><td>GO Component</td><td>cleavage furrow</td><td>MYH9|MYH10|SEPT7</td><td>0.0322</td><td>GO.0032154</td></tr>
<tr><td>8</td><td>GO Component</td><td>presynapse</td><td>CAD|ITGA2|BCL2L1|BRSK1|SNCA|DNM1|CAMK2A|APBA2</td><td>0.0322</td><td>GO.0098793</td></tr>
<tr><td>4</td><td>GO Component</td><td>Golgi lumen</td><td>APP|MMP14|SDC4|SDC1</td><td>0.0333</td><td>GO.0005796</td></tr>
<tr><td>2</td><td>GO Component</td><td>glial cell projection</td><td>APP|FYN</td><td>0.0341</td><td>GO.0097386</td></tr>
<tr><td>5</td><td>GO Component</td><td>growth cone</td><td>NGEF|APP|SNCA|MYH10|FSCN1</td><td>0.0345</td><td>GO.0030426</td></tr>
<tr><td>4</td><td>GO Component</td><td>melanosome</td><td>MMP14|ANXA2|ANXA6|PDIA6</td><td>0.037</td><td>GO.0042470</td></tr>
<tr><td>2</td><td>GO Component</td><td>costamere</td><td>FLNC|SDC4</td><td>0.037</td><td>GO.0043034</td></tr>
<tr><td>3</td><td>GO Component</td><td>neuromuscular junction</td><td>MYH9|APP|MYH10</td><td>0.038</td><td>GO.0031594</td></tr>
<tr><td>9</td><td>GO Component</td><td>nuclear envelope</td><td>GAPDH|APP|BCL2L1|CALR|SNCA|PTGES|MYO1C|BCL2|SUN1</td><td>0.0388</td><td>GO.0005635</td></tr>
<tr><td>3</td><td>GO Component</td><td>tethering complex</td><td>EXOC6B|STXBP6|CDC42</td><td>0.0393</td><td>GO.0099023</td></tr>
<tr><td>5</td><td>GO Component</td><td>vacuolar lumen</td><td>CCT2|TUBB4B|ANXA2|SDC4|SDC1</td><td>0.0444</td><td>GO.0005775</td></tr>
<tr><td>3</td><td>GO Component</td><td>sarcoplasmic reticulum</td><td>THBS1|CALR|CAMK2B</td><td>0.0444</td><td>GO.0016529</td></tr>
<tr><td>2</td><td>GO Component</td><td>mast cell granule</td><td>KIT|ANXA1</td><td>0.045</td><td>GO.0042629</td></tr>
<tr><td>4</td><td>GO Component</td><td>acrosomal vesicle</td><td>KIT|CALR|CAV1|SUN1</td><td>0.0463</td><td>GO.0001669</td></tr>
<tr><td>2</td><td>GO Component</td><td>acrosomal membrane</td><td>CAV1|SUN1</td><td>0.0479</td><td>GO.0002080</td></tr>
<tr><td>2</td><td>GO Component</td><td>rough endoplasmic reticulum membrane</td><td>PLOD1|PLOD2</td><td>0.0479</td><td>GO.0030867</td></tr>
<tr><td>121</td><td>GO Function</td><td>protein binding</td><td>ARHGEF5|PLOD1|ITGB4|MYH9|APLP1|CCL2|GAPDH|LAMA4|PPP3R1|PLEK|ENO1|CDC25B|HSPB1|BCL2L2|PXDN|LAMA5|PDGFRA|THBS1|VWF|RORA|DNAJB6|CNN2|NGEF|ARHGAP31|CAD|ADD1|ICAM1|FERMT3|TJP1|FLT1|APP|CSF1R|KIT|NPC1L1|CCR5|CAPN2|ITGA2|NOS3|CKB|CCT2|PLK1|TUBA1A|DDB1|BCL2L1|CDC25A|SDPR|ARF4|MMP14|BRSK1|DAB2|TENC1|NQO1|CALR|CDC25C|STXBP6|FLNC|JAG2|PRKCH|RARB|LCK|SNCA|CAV1|TUBB4B|CDH5|PTMA|TPM4|ANXA2|ANXA6|FYN|FN1|FAS|ARHGEF26|PTRF|SFPQ|MYO1C|MYH10|DPP4|ARHGEF15|TGM2|DLL1|COPA|TPM3|SLK|FLNA|CNN3|PTPN1|SDC4|SPTAN1|DNM1|TLR4|ANXA1|TBC1D4|TPM2|PSIP1|SDC1|FSCN1|XRCC5|MYO1B|G6PD|PPP3CA|CAMK2B|LDHB|BCL2|CAMK2A|DYRK1A|SEPT7|PAICS|CDC42|SUN1|RARG|TOP2B|FLNB|SPTBN2|CFL1|ARF1|VIM|NR1H4|NUMB|TLN2|PRKCSH|CAMK1D</td><td>1.38E-22</td><td>GO.0005515</td></tr>
<tr><td>153</td><td>GO Function</td><td>binding</td><td>RCN1|ARHGEF5|MAP2K5|PLOD1|ITGB4|MYH9|CYP24A1|MMP2|EHD4|APLP1|CCL2|GAPDH|LAMA4|RBP1|GNB4|PPP3R1|PLEK|ENO1|MT2A|CDC25B|HSPB1|BCL2L2|PXDN|LAMA5|ARF3|PDGFRA|THBS1|VWF|RORA|DNAJB6|CNN2|PRKCG|AKT3|NGEF|TUBB4A|ARHGAP31|CAD|ADD1|ICAM1|FERMT3|TJP1|FLT1|PLOD2|APP|CSF1R|KIT|NPC1L1|CCR5|CAPN2|ANXA5|ITGA2|NOS3|MELK|CKB|CCT2|PLK1|TUBA1A|TUBA1C|DDB1|BCL2L1|CDC25A|SDPR|ARF4|MMP14|BRSK1|DAB2|TENC1|NQO1|CALR|CDC25C|STXBP6|ZYX|RORC|FLNC|JAG2|PRKCH|RARB|CYP2R1|NR1I2|LCK|SNCA|CAV1|TUBB4B|PTGES|MAP4K4|CDH5|PTMA|TPM4|ANXA2|ANXA6|FYN|FN1|FAS|ARHGEF26|PTRF|SFPQ|EPHA2|MYO1C|RXRG|MYH10|DPP4|ARHGEF15|TGM2|DLL1|COPA|TPM3|SLK|FLNA|CNN3|PTPN1|SDC4|SPTAN1|DNM1|TLR4|RXRB|ANXA1|TBC1D4|TPM2|PIR|PSIP1|SDC1|FSCN1|XRCC5|MYO1B|G6PD|PPP3CA|CAMK2B|LDHB|LTB4R|BCL2|CAMK2A|DYRK1A|SEPT7|PAICS|CDC42|SUN1|RARG|TOP2B|WEE1|FLNB|PBK|SPTBN2|CFL1|ARF1|VIM|NR1H4|NUMB|APBA2|TLN2|PRKCSH|ABCB1|CAMK1D|MCM3</td><td>4.35E-19</td><td>GO.0005488</td></tr>
<tr><td>66</td><td>GO Function</td><td>anion binding</td><td>ARHGEF5|MAP2K5|PLOD1|MYH9|EHD4|APLP1|PLEK|ARF3|PDGFRA|THBS1|PRKCG|AKT3|TUBB4A|CAD|FLT1|PLOD2|APP|CSF1R|KIT|ANXA5|ITGA2|NOS3|MELK|CKB|CCT2|PLK1|TUBA1A|TUBA1C|SDPR|ARF4|BRSK1|STXBP6|PRKCH|LCK|SNCA|TUBB4B|PTGES|MAP4K4|ANXA2|ANXA6|FYN|FN1|EPHA2|MYO1C|MYH10|TGM2|SLK|DNM1|ANXA1|XRCC5|MYO1B|CAMK2B|CAMK2A|DYRK1A|SEPT7|PAICS|CDC42|TOP2B|WEE1|PBK|SPTBN2|ARF1|NR1H4|ABCB1|CAMK1D|MCM3</td><td>1.86E-14</td><td>GO.0043168</td></tr>
<tr><td>52</td><td>GO Function</td><td>identical protein binding</td><td>PLOD1|MYH9|APLP1|GAPDH|PLEK|ENO1|HSPB1|BCL2L2|PDGFRA|THBS1|VWF|DNAJB6|CAD|ADD1|APP|CSF1R|KIT|PLK1|BCL2L1|NQO1|FLNC|LCK|SNCA|CAV1|TPM4|ANXA2|ANXA6|FYN|FN1|FAS|PTRF|SFPQ|DPP4|SLK|FLNA|SDC4|DNM1|TLR4|ANXA1|TPM2|SDC1|G6PD|CAMK2B|LDHB|BCL2|CAMK2A|DYRK1A|SEPT7|PAICS|CDC42|FLNB|VIM</td><td>5.47E-14</td><td>GO.0042802</td></tr>
<tr><td>56</td><td>GO Function</td><td>enzyme binding</td><td>ARHGEF5|PPP3R1|PLEK|ENO1|CDC25B|HSPB1|PDGFRA|VWF|NGEF|CAD|APP|CSF1R|KIT|NPC1L1|CKB|CCT2|PLK1|BCL2L1|CDC25A|SDPR|ARF4|BRSK1|TENC1|CALR|CDC25C|STXBP6|PRKCH|LCK|SNCA|CAV1|CDH5|ANXA2|FYN|FN1|FAS|ARHGEF26|SFPQ|MYO1C|DPP4|ARHGEF15|FLNA|PTPN1|SDC4|SPTAN1|DNM1|TBC1D4|XRCC5|PPP3CA|CAMK2B|LDHB|BCL2|CAMK2A|CDC42|TOP2B|SPTBN2|PRKCSH</td><td>1.33E-12</td><td>GO.0019899</td></tr>
<tr><td>55</td><td>GO Function</td><td>carbohydrate derivative binding</td><td>ARHGEF5|MAP2K5|MYH9|EHD4|APLP1|ARF3|PDGFRA|THBS1|PRKCG|AKT3|TUBB4A|CAD|FLT1|APP|CSF1R|KIT|ITGA2|NOS3|MELK|CKB|CCT2|PLK1|TUBA1A|TUBA1C|ARF4|BRSK1|PRKCH|LCK|TUBB4B|MAP4K4|ANXA6|FYN|FN1|EPHA2|MYO1C|MYH10|TGM2|SLK|DNM1|TLR4|XRCC5|MYO1B|CAMK2B|CAMK2A|DYRK1A|SEPT7|PAICS|CDC42|TOP2B|WEE1|PBK|ARF1|ABCB1|CAMK1D|MCM3</td><td>2.35E-12</td><td>GO.0097367</td></tr>
<tr><td>58</td><td>GO Function</td><td>small molecule binding</td><td>ARHGEF5|MAP2K5|PLOD1|MYH9|EHD4|GAPDH|RBP1|ARF3|PDGFRA|PRKCG|AKT3|TUBB4A|CAD|FLT1|PLOD2|CSF1R|KIT|NOS3|MELK|CKB|CCT2|PLK1|TUBA1A|TUBA1C|ARF4|BRSK1|PRKCH|LCK|CAV1|TUBB4B|MAP4K4|ANXA6|FYN|EPHA2|MYO1C|MYH10|TGM2|SLK|DNM1|XRCC5|MYO1B|G6PD|CAMK2B|LDHB|LTB4R|CAMK2A|DYRK1A|SEPT7|PAICS|CDC42|TOP2B|WEE1|PBK|ARF1|NR1H4|ABCB1|CAMK1D|MCM3</td><td>7.32E-12</td><td>GO.0036094</td></tr>
</table>

<table>
<tr><td>53</td><td>GO Function</td><td>nucleotide binding</td><td>ARHGEF5|MAP2K5|MYH9|EHD4|GAPDH|ARF3|PDGFRA|PRKCG|AKT3|TUBB4A|CAD|FLT1|CSF1R|KIT|NOS3|MELK|CKB|CCT2|PLK1|TUBA1A|TUBA1C|ARF4|BRSK1|PRKCH|LCK|TUBB4B|MAP4K4|ANXA6|FYN|EPHA2|MYO1C|MYH10|TGM2|SLK|DNM1|XRCC5|MYO1B|G6PD|CAMK2B|LDHB|LTB4R|CAMK2A|DYRK1A|SEPT7|PAICS|CDC42|TOP2B|WEE1|PBK|ARF1|ABCB1|CAMK1D|MCM3</td><td>8.06E-12</td><td>GO.0000166</td></tr>
<tr><td>49</td><td>GO Function</td><td>ribonucleotide binding</td><td>ARHGEF5|MAP2K5|MYH9|EHD4|ARF3|PDGFRA|PRKCG|AKT3|TUBB4A|CAD|FLT1|CSF1R|KIT|NOS3|MELK|CKB|CCT2|PLK1|TUBA1A|TUBA1C|ARF4|BRSK1|PRKCH|LCK|TUBB4B|MAP4K4|ANXA6|FYN|EPHA2|MYO1C|MYH10|TGM2|SLK|DNM1|XRCC5|MYO1B|CAMK2B|CAMK2A|DYRK1A|SEPT7|PAICS|CDC42|TOP2B|WEE1|PBK|ARF1|ABCB1|CAMK1D|MCM3</td><td>1.88E-11</td><td>GO.0032553</td></tr>
<tr><td>48</td><td>GO Function</td><td>purine ribonucleoside triphosphate binding</td><td>ARHGEF5|MAP2K5|MYH9|EHD4|ARF3|PDGFRA|PRKCG|AKT3|TUBB4A|CAD|FLT1|CSF1R|KIT|MELK|CKB|CCT2|PLK1|TUBA1A|TUBA1C|ARF4|BRSK1|PRKCH|LCK|TUBB4B|MAP4K4|ANXA6|FYN|EPHA2|MYO1C|MYH10|TGM2|SLK|DNM1|XRCC5|MYO1B|CAMK2B|CAMK2A|DYRK1A|SEPT7|PAICS|CDC42|TOP2B|WEE1|PBK|ARF1|ABCB1|CAMK1D|MCM3</td><td>1.88E-11</td><td>GO.0035639</td></tr>
<tr><td>35</td><td>GO Function</td><td>protein-containing complex binding</td><td>MYH9|GNB4|LAMA5|PDGFRA|THBS1|VWF|ADD1|ICAM1|FERMT3|APP|ITGA2|PLK1|DDB1|MMP14|CALR|FLNC|RARB|LCK|SNCA|TPM4|FYN|FN1|MYO1C|MYH10|TPM3|FLNA|PTPN1|DNM1|TPM2|FSCN1|XRCC5|MYO1B|CFL1|VIM|TLN2</td><td>1.88E-11</td><td>GO.0044877</td></tr>
<tr><td>48</td><td>GO Function</td><td>purine ribonucleotide binding</td><td>ARHGEF5|MAP2K5|MYH9|EHD4|ARF3|PDGFRA|PRKCG|AKT3|TUBB4A|CAD|FLT1|CSF1R|KIT|MELK|CKB|CCT2|PLK1|TUBA1A|TUBA1C|ARF4|BRSK1|PRKCH|LCK|TUBB4B|MAP4K4|ANXA6|FYN|EPHA2|MYO1C|MYH10|TGM2|SLK|DNM1|XRCC5|MYO1B|CAMK2B|CAMK2A|DYRK1A|SEPT7|PAICS|CDC42|TOP2B|WEE1|PBK|ARF1|ABCB1|CAMK1D|MCM3</td><td>4.61E-11</td><td>GO.0032555</td></tr>
<tr><td>95</td><td>GO Function</td><td>ion binding</td><td>RCN1|ARHGEF5|MAP2K5|PLOD1|MYH9|CYP24A1|MMP2|EHD4|APLP1|PPP3R1|PLEK|ENO1|MT2A|PXDN|ARF3|PDGFRA|THBS1|RORA|PRKCG|AKT3|TUBB4A|CAD|FLT1|PLOD2|APP|CSF1R|KIT|CAPN2|ANXA5|ITGA2|NOS3|MELK|CKB|CCT2|PLK1|TUBA1A|TUBA1C|SDPR|ARF4|MMP14|BRSK1|TENC1|CALR|STXBP6|ZYX|RORC|JAG2|PRKCH|RARB|CYP2R1|NR1I2|LCK|SNCA|TUBB4B|PTGES|MAP4K4|CDH5|TPM4|ANXA2|ANXA6|FYN|FN1|EPHA2|MYO1C|RXRG|MYH10|TGM2|DLL1|SLK|PTPN1|SPTAN1|DNM1|RXRB|ANXA1|PIR|XRCC5|MYO1B|PPP3CA|CAMK2B|CAMK2A|DYRK1A|SEPT7|PAICS|CDC42|RARG|TOP2B|WEE1|PBK|SPTBN2|ARF1|NR1H4|PRKCSH|ABCB1|CAMK1D|MCM3</td><td>5.31E-11</td><td>GO.0043167</td></tr>
<tr><td>23</td><td>GO Function</td><td>actin binding</td><td>MYH9|CNN2|ADD1|CCR5|NOS3|FLNC|SNCA|TPM4|MYO1C|MYH10|TPM3|FLNA|CNN3|SPTAN1|TPM2|FSCN1|MYO1B|CAMK2B|DYRK1A|FLNB|SPTBN2|CFL1|TLN2</td><td>6.57E-11</td><td>GO.0003779</td></tr>
<tr><td>31</td><td>GO Function</td><td>cytoskeletal protein binding</td><td>MYH9|GAPDH|CNN2|ADD1|NPC1L1|CCR5|CAPN2|NOS3|PLK1|BRSK1|FLNC|SNCA|TPM4|ANXA2|FYN|MYO1C|MYH10|TPM3|FLNA|CNN3|SPTAN1|DNM1|TPM2|FSCN1|MYO1B|CAMK2B|DYRK1A|FLNB|SPTBN2|CFL1|TLN2</td><td>6.60E-10</td><td>GO.0008092</td></tr>
<tr><td>16</td><td>GO Function</td><td>calmodulin binding</td><td>MYH9|PPP3R1|CNN2|ADD1|TJP1|NOS3|FAS|MYO1C|MYH10|CNN3|SPTAN1|MYO1B|PPP3CA|CAMK2B|CAMK2A|CAMK1D</td><td>7.68E-10</td><td>GO.0005516</td></tr>
<tr><td>43</td><td>GO Function</td><td>drug binding</td><td>MAP2K5|MYH9|EHD4|PPP3R1|MT2A|PDGFRA|PRKCG|AKT3|CAD|FLT1|CSF1R|KIT|NPC1L1|NOS3|MELK|CKB|CCT2|PLK1|BRSK1|PRKCH|RARB|NR1I2|LCK|MAP4K4|FYN|EPHA2|MYO1C|MYH10|SLK|FSCN1|XRCC5|MYO1B|PPP3CA|CAMK2B|CAMK2A|DYRK1A|PAICS|TOP2B|WEE1|PBK|ABCB1|CAMK1D|MCM3</td><td>1.76E-09</td><td>GO.0008144</td></tr>
<tr><td>25</td><td>GO Function</td><td>protein kinase activity</td><td>MAP2K5|CCL2|PDGFRA|PRKCG|AKT3|CAD|FLT1|CSF1R|KIT|MELK|PLK1|BRSK1|PRKCH|LCK|MAP4K4|FYN|EPHA2|SLK|CAMK2B|CAMK2A|DYRK1A|CDC42|WEE1|PBK|CAMK1D</td><td>6.75E-09</td><td>GO.0004672</td></tr>
<tr><td>38</td><td>GO Function</td><td>signaling receptor binding</td><td>ITGB4|APLP1|CCL2|LAMA4|PXDN|LAMA5|PDGFRA|THBS1|VWF|ICAM1|FERMT3|APP|ITGA2|ARF4|MMP14|CALR|JAG2|RARB|LCK|CAV1|TUBB4B|CDH5|FYN|FN1|MYO1C|DPP4|DLL1|COPA|FLNA|PTPN1|DNM1|TLR4|ANXA1|CAMK2A|CDC42|RARG|CFL1|NR1H4</td><td>2.90E-08</td><td>GO.0005102</td></tr>
<tr><td>13</td><td>GO Function</td><td>actin filament binding</td><td>MYH9|ADD1|FLNC|TPM4|MYO1C|MYH10|TPM3|FLNA|TPM2|FSCN1|MYO1B|CFL1|TLN2</td><td>5.18E-08</td><td>GO.0051015</td></tr>
<tr><td>26</td><td>GO Function</td><td>kinase activity</td><td>MAP2K5|CCL2|PDGFRA|PRKCG|AKT3|CAD|FLT1|CSF1R|KIT|MELK|CKB|PLK1|BRSK1|PRKCH|LCK|MAP4K4|FYN|EPHA2|SLK|CAMK2B|CAMK2A|DYRK1A|CDC42|WEE1|PBK|CAMK1D</td><td>2.79E-07</td><td>GO.0016301</td></tr>
<tr><td>33</td><td>GO Function</td><td>protein dimerization activity</td><td>PLOD1|MYH9|PLEK|ENO1|HSPB1|BCL2L2|PDGFRA|VWF|ADD1|APP|CSF1R|KIT|CAPN2|ITGA2|BCL2L1|CAV1|TPM4|ANXA6|SFPQ|DPP4|SLK|FLNA|DNM1|TLR4|ANXA1|TPM2|G6PD|PPP3CA|CAMK2B|BCL2|CAMK2A|TOP2B|ARF1</td><td>3.07E-07</td><td>GO.0046983</td></tr>
<tr><td>35</td><td>GO Function</td><td>ATP binding</td><td>MAP2K5|MYH9|EHD4|PDGFRA|PRKCG|AKT3|CAD|FLT1|CSF1R|KIT|MELK|CKB|CCT2|PLK1|BRSK1|PRKCH|LCK|MAP4K4|FYN|EPHA2|MYO1C|MYH10|SLK|XRCC5|MYO1B|CAMK2B|CAMK2A|DYRK1A|PAICS|TOP2B|WEE1|PBK|ABCB1|CAMK1D|MCM3</td><td>4.10E-07</td><td>GO.0005524</td></tr>
<tr><td>8</td><td>GO Function</td><td>nuclear receptor activity</td><td>RORA|RORC|RARB|NR1I2|RXRG|RXRB|RARG|NR1H4</td><td>5.63E-07</td><td>GO.0004879</td></tr>
<tr><td>25</td><td>GO Function</td><td>protein homodimerization activity</td><td>PLOD1|MYH9|PLEK|ENO1|HSPB1|BCL2L2|PDGFRA|VWF|ADD1|APP|CSF1R|KIT|BCL2L1|TPM4|ANXA6|SFPQ|DPP4|SLK|FLNA|ANXA1|TPM2|G6PD|CAMK2B|BCL2|CAMK2A</td><td>8.56E-07</td><td>GO.0042803</td></tr>
<tr><td>21</td><td>GO Function</td><td>GTPase binding</td><td>ARHGEF5|ENO1|PDGFRA|NGEF|KIT|NPC1L1|ARF4|STXBP6|PRKCH|CAV1|ANXA2|FYN|ARHGEF26|MYO1C|ARHGEF15|FLNA|SPTAN1|TBC1D4|CAMK2B|CAMK2A|SPTBN2</td><td>1.37E-06</td><td>GO.0051020</td></tr>
<tr><td>8</td><td>GO Function</td><td>steroid hormone receptor activity</td><td>RORA|RORC|RARB|NR1I2|RXRG|RXRB|RARG|NR1H4</td><td>1.47E-06</td><td>GO.0003707</td></tr>
<tr><td>19</td><td>GO Function</td><td>Ras GTPase binding</td><td>ARHGEF5|PDGFRA|NGEF|KIT|NPC1L1|STXBP6|PRKCH|CAV1|ANXA2|FYN|ARHGEF26|MYO1C|ARHGEF15|FLNA|SPTAN1|TBC1D4|CAMK2B|CAMK2A|SPTBN2</td><td>1.58E-06</td><td>GO.0017016</td></tr>
<tr><td>10</td><td>GO Function</td><td>integrin binding</td><td>LAMA5|THBS1|VWF|ICAM1|FERMT3|APP|ITGA2|MMP14|CALR|FN1</td><td>2.55E-06</td><td>GO.0005178</td></tr>
<tr><td>42</td><td>GO Function</td><td>catalytic activity, acting on a protein</td><td>MAP2K5|PLOD1|MMP2|CCL2|GAPDH|PPP3R1|CDC25B|PDGFRA|PRKCG|AKT3|CAD|FLT1|PLOD2|CSF1R|KIT|CAPN2|MELK|PLK1|CDC25A|MMP14|BRSK1|TENC1|CDC25C|PRKCH|LCK|USP1|MAP4K4|FYN|EPHA2|DPP4|TGM2|SLK|PTPN1|PPP3CA|CAMK2B|CAMK2A|DYRK1A|CDC42|PDIA6|WEE1|PBK|CAMK1D</td><td>3.32E-06</td><td>GO.0140096</td></tr>
<tr><td>76</td><td>GO Function</td><td>organic cyclic compound binding</td><td>ARHGEF5|MAP2K5|MYH9|CYP24A1|EHD4|GAPDH|ENO1|PXDN|ARF3|PDGFRA|RORA|PRKCG|AKT3|TUBB4A|CAD|FLT1|APP|CSF1R|KIT|NOS3|MELK|CKB|CCT2|PLK1|TUBA1A|TUBA1C|DDB1|ARF4|BRSK1|CALR|RORC|PRKCH|RARB|CYP2R1|NR1I2|LCK|SNCA|CAV1|TUBB4B|MAP4K4|ANXA6|FYN|PTRF|SFPQ|EPHA2|MYO1C|RXRG|MYH10|TGM2|SLK|DNM1|RXRB|ANXA1|PSIP1|XRCC5|MYO1B|G6PD|CAMK2B|LDHB|LTB4R|BCL2|CAMK2A|DYRK1A|SEPT7|PAICS|CDC42|RARG|TOP2B|WEE1|PBK|ARF1|VIM|NR1H4|ABCB1|CAMK1D|MCM3</td><td>3.65E-06</td><td>GO.0097159</td></tr>
<tr><td>75</td><td>GO Function</td><td>heterocyclic compound binding</td><td>ARHGEF5|MAP2K5|MYH9|CYP24A1|EHD4|GAPDH|ENO1|PXDN|ARF3|PDGFRA|RORA|PRKCG|AKT3|TUBB4A|CAD|FLT1|APP|CSF1R|KIT|NOS3|MELK|CKB|CCT2|PLK1|TUBA1A|TUBA1C|DDB1|ARF4|BRSK1|CALR|RORC|PRKCH|RARB|CYP2R1|NR1I2|LCK|SNCA|TUBB4B|MAP4K4|ANXA6|FYN|PTRF|SFPQ|EPHA2|MYO1C|RXRG|MYH10|TGM2|SLK|DNM1|RXRB|ANXA1|PSIP1|XRCC5|MYO1B|G6PD|CAMK2B|LDHB|LTB4R|BCL2|CAMK2A|DYRK1A|SEPT7|PAICS|CDC42|RARG|TOP2B|WEE1|PBK|ARF1|VIM|NR1H4|ABCB1|CAMK1D|MCM3</td><td>4.30E-06</td><td>GO.1901363</td></tr>
<tr><td>21</td><td>GO Function</td><td>kinase binding</td><td>PLEK|CDC25B|HSPB1|PLK1|BCL2L1|CDC25A|SDPR|BRSK1|TENC1|CDC25C|LCK|CAV1|FAS|FLNA|PTPN1|SDC4|DNM1|LDHB|CDC42|TOP2B|PRKCSH</td><td>5.24E-06</td><td>GO.0019900</td></tr>
<tr><td>11</td><td>GO Function</td><td>protein tyrosine kinase activity</td><td>MAP2K5|PDGFRA|FLT1|CSF1R|KIT|MELK|LCK|FYN|EPHA2|DYRK1A|WEE1</td><td>8.33E-06</td><td>GO.0004713</td></tr>
<tr><td>20</td><td>GO Function</td><td>calcium ion binding</td><td>RCN1|EHD4|PPP3R1|THBS1|TUBB4A|CAPN2|ANXA5|MELK|CALR|JAG2|SNCA|CDH5|TPM4|ANXA2|ANXA6|DLL1|SPTAN1|ANXA1|PPP3CA|PRKCSH</td><td>3.24E-05</td><td>GO.0005509</td></tr>
<tr><td>20</td><td>GO Function</td><td>protein domain specific binding</td><td>MYH9|GAPDH|PPP3R1|BCL2L2|ARHGAP31|APP|TUBA1A|DDB1|BCL2L1|CDC25C|LCK|SNCA|FYN|FN1|TGM2|DNM1|BCL2|CDC42|ARF1|VIM</td><td>3.59E-05</td><td>GO.0019904</td></tr>
</table>

14	GO Function	GTP binding	ARHGEF5|EHD4|ARF3|TUBB4A|TUBA1A|TUBA1C|ARF4|TUBB4B|ANXA6|TGM2|DNM1|SEPT7|CDC42|ARF1	4.81E-05	GO.0005525
8	GO Function	structural constituent of cytoskeleton	TUBB4A|TUBA1A|TUBA1C|TUBB4B|SPTAN1|SPTBN2|VIM|TLN2	6.01E-05	GO.0005200
19	GO Function	lipid binding	ARHGEF5|RBP1|PLEK|THBS1|RORA|ANXA5|MELK|SDPR|STXBP6|RORC|SNCA|CAV1|ANXA2|ANXA6|TLR4|ANXA1|MYO1B|SPTBN2|NR1H4	6.01E-05	GO.0008289
74	GO Function	catalytic activity	MAP2K5|PLOD1|MYH9|CYP24A1|MMP2|CCL2|GAPDH|PPP3R1|ENO1|CDC25B|PXDN|ARF3|PDGFRA|PRKCG|AKT3|TUBB4A|CAD|FLT1|PLOD2|CSF1R|KIT|CCR5|CAPN2|NOS3|MELK|CKB|PLK1|TUBA1A|TUBA1C|CDC25A|ARF4|MMP14|BRSK1|TENC1|NQO1|CDC25C|PRKCH|CYP2R1|LCK|SNCA|TUBB4B|PTGES|USP1|MAP4K4|FYN|EPHA2|MYO1C|MYH10|DPP4|TGM2|GSTO1|SLK|PTPN1|DNM1|ANXA1|PIR|XRCC5|MYO1B|G6PD|PPP3CA|CAMK2B|LDHB|CAMK2A|DYRK1A|PAICS|CDC42|PDIA6|TOP2B|WEE1|PBK|ARF1|ABCB1|CAMK1D|MCM3	6.28E-05	GO.0003824
4	GO Function	actin-dependent ATPase activity	MYH9|MYO1C|MYH10|MYO1B	6.49E-05	GO.0030898
6	GO Function	protein kinase C binding	PLEK|HSPB1|SDPR|SDC4|TOP2B|PRKCSH	6.78E-05	GO.0005080
15	GO Function	protein serine/threonine kinase activity	MAP2K5|PRKCG|AKT3|MELK|PLK1|BRSK1|PRKCH|MAP4K4|SLK|CAMK2B|CAMK2A|DYRK1A|CDC42|PBK|CAMK1D	7.23E-05	GO.0004674
11	GO Function	Ras guanyl-nucleotide exchange factor activity	ARHGEF5|PDGFRA|NGEF|KIT|FYN|ARHGEF26|ARHGEF15|SPTAN1|CAMK2B|CAMK2A|SPTBN2	9.53E-05	GO.0005088
17	GO Function	protein kinase binding	PLEK|CDC25B|HSPB1|PLK1|BCL2L1|CDC25A|SDPR|BRSK1|CDC25C|LCK|CAV1|PTPN1|SDC4|DNM1|CDC42|TOP2B|PRKCSH	1.50E-04	GO.0019901
33	GO Function	molecular function regulator	ARHGEF5|CCL2|GAPDH|HSPB1|PXDN|PDGFRA|DNAJB6|NGEF|ARHGAP31|APP|KIT|ANXA5|ARF4|JAG2|SNCA|CAV1|RGS12|ANXA2|FYN|FN1|ARHGEF26|ARHGEF15|ARHGAP30|COPA|FLNA|SPTAN1|ANXA1|TBC1D4|XRCC5|CAMK2B|BCL2|CAMK2A|SPTBN2	1.50E-04	GO.0098772
12	GO Function	guanyl-nucleotide exchange factor activity	ARHGEF5|PDGFRA|NGEF|KIT|ARF4|FYN|ARHGEF26|ARHGEF15|SPTAN1|CAMK2B|CAMK2A|SPTBN2	1.60E-04	GO.0005085
18	GO Function	structural molecule activity	LAMA4|PXDN|TUBB4A|ADD1|TUBA1A|TUBA1C|CAV1|TUBB4B|TPM4|COPA|TPM3|SPTAN1|ANXA1|TPM2|SEPT7|SPTBN2|VIM|TLN2	1.90E-04	GO.0005198
23	GO Function	transition metal ion binding	PLOD1|CYP24A1|MMP2|APLP1|MT2A|RORA|PRKCG|CAD|PLOD2|APP|NOS3|MMP14|CALR|RORC|RARB|CYP2R1|NR1I2|SNCA|RXRG|PTPN1|RXRB|RARG|NR1H4	2.60E-04	GO.0046914
57	GO Function	metal ion binding	RCN1|MAP2K5|PLOD1|CYP24A1|MMP2|EHD4|APLP1|PPP3R1|ENO1|MT2A|PXDN|THBS1|RORA|PRKCG|TUBB4A|CAD|PLOD2|APP|KIT|CAPN2|ANXA5|ITGA2|NOS3|MELK|PLK1|MMP14|BRSK1|TENC1|CALR|ZYX|RORC|JAG2|PRKCH|RARB|CYP2R1|NR1I2|SNCA|CDH5|TPM4|ANXA2|ANXA6|FYN|RXRG|TGM2|DLL1|PTPN1|SPTAN1|RXRB|ANXA1|PIR|PPP3CA|CAMK2A|RARG|TOP2B|WEE1|NR1H4|PRKCSH	2.80E-04	GO.0046872
12	GO Function	tubulin binding	MYH9|GAPDH|PLK1|BRSK1|SNCA|FYN|MYO1C|MYH10|CNN3|DNM1|MYO1B|DYRK1A	4.00E-04	GO.0015631
4	GO Function	microfilament motor activity	MYH9|MYO1C|MYH10|MYO1B	4.50E-04	GO.0000146
9	GO Function	protein C-terminus binding	DAB2|LCK|FN1|MYO1C|DNM1|SDC1|XRCC5|TOP2B|VIM	4.50E-04	GO.0008022
5	GO Function	non-membrane spanning protein tyrosine kinase activity	MELK|LCK|FYN|DYRK1A|WEE1	8.50E-04	GO.0004715
18	GO Function	nucleoside-triphosphatase activity	MYH9|ARF3|TUBB4A|TUBA1A|TUBA1C|ARF4|TUBB4B|MYO1C|MYH10|DNM1|ANXA1|XRCC5|MYO1B|CDC42|TOP2B|ARF1|ABCB1|MCM3	9.60E-04	GO.0017111
8	GO Function	phosphoprotein phosphatase activity	PPP3R1|CDC25B|CDC25A|TENC1|CDC25C|LCK|PTPN1|PPP3CA	0.0012	GO.0004721
14	GO Function	protein heterodimerization activity	BCL2L2|ADD1|APP|CAPN2|ITGA2|BCL2L1|CAV1|TPM4|TLR4|TPM2|PPP3CA|BCL2|TOP2B|ARF1	0.0012	GO.0046982
3	GO Function	BH domain binding	BCL2L2|BCL2L1|BCL2	0.0012	GO.0051400
3	GO Function	phospholipase inhibitor activity	ANXA5|ANXA2|ANXA1	0.0015	GO.0004859
5	GO Function	transmembrane receptor protein tyrosine kinase activity	PDGFRA|FLT1|CSF1R|KIT|EPHA2	0.0017	GO.0004714
6	GO Function	cytokine binding	PXDN|THBS1|CSF1R|KIT|CCR5|FAS	0.0018	GO.0019955
4	GO Function	disordered domain specific binding	GAPDH|BCL2L2|FYN|FN1	0.0018	GO.0097718
6	GO Function	chaperone binding	VWF|DNAJB6|APP|CDC25A|CALR|FN1	0.0019	GO.0051087
9	GO Function	microtubule binding	MYH9|GAPDH|PLK1|SNCA|MYO1C|MYH10|CNN3|DNM1|MYO1B	0.0025	GO.0008017
2	GO Function	CD8 receptor binding	LCK|FYN	0.0029	GO.0042610
3	GO Function	retinoid X receptor binding	RARB|RARG|NR1H4	0.0029	GO.0046965
11	GO Function	phospholipid binding	PLEK|THBS1|ANXA5|SDPR|STXBP6|SNCA|ANXA2|ANXA6|ANXA1|MYO1B|SPTBN2	0.0032	GO.0005543
3	GO Function	Ral GTPase binding	PRKCH|MYO1C|FLNA	0.0033	GO.0017160
5	GO Function	virus receptor activity	ICAM1|CCR5|ITGA2|EPHA2|DPP4	0.0036	GO.0001618
7	GO Function	Rho GTPase binding	ARHGEF5|NGEF|STXBP6|CAV1|ARHGEF26|ARHGEF15|FLNA	0.0036	GO.0017048
2	GO Function	procollagen-lysine 5-dioxygenase activity	PLOD1|PLOD2	0.0042	GO.0008475
3	GO Function	tau protein binding	SNCA|FYN|DYRK1A	0.0042	GO.0048156
9	GO Function	GTPase activity	ARF3|TUBB4A|TUBA1A|TUBA1C|ARF4|TUBB4B|DNM1|CDC42|ARF1	0.0048	GO.0003924
4	GO Function	calcium-dependent phospholipid binding	ANXA5|ANXA2|ANXA6|ANXA1	0.0052	GO.0005544
2	GO Function	phospholipase A2 inhibitor activity	ANXA2|ANXA1	0.0061	GO.0019834
7	GO Function	protein binding, bridging	MYH9|DDB1|DAB2|CAV1|ANXA1|FSCN1|SUN1	0.0062	GO.0030674
4	GO Function	sterol binding	RORA|RORC|CAV1|ANXA6	0.0062	GO.0032934
5	GO Function	steroid binding	RORA|RORC|CAV1|ANXA6|NR1H4	0.0063	GO.0005496
6	GO Function	protease binding	VWF|KIT|ANXA2|FN1|DPP4|BCL2	0.0068	GO.0002020
16	GO Function	zinc ion binding	MMP2|MT2A|RORA|PRKCG|CAD|MMP14|CALR|RORC|RARB|NR1I2|SNCA|RXRG|PTPN1|RXRB|RARG|NR1H4	0.0073	GO.0008270
2	GO Function	oxysterol binding	RORA|RORC	0.0079	GO.0008142
2	GO Function	BH3 domain binding	BCL2L1|BCL2	0.0079	GO.0051434
3	GO Function	protein folding chaperone	HSPB1|CCT2|CALR	0.0087	GO.0044183
6	GO Function	iron ion binding	PLOD1|CYP24A1|PLOD2|CALR|CYP2R1|SNCA	0.0097	GO.0005506
2	GO Function	creatine kinase activity	CKB|MAP4K4	0.01	GO.0004111
4	GO Function	collagen binding	THBS1|VWF|ITGA2|FN1	0.011	GO.0005518
3	GO Function	calmodulin-dependent protein kinase activity	CAMK2B|CAMK2A|CAMK1D	0.0111	GO.0004683
3	GO Function	ephrin receptor binding	APP|FYN|PTPN1	0.0111	GO.0046875
2	GO Function	calcium-dependent protein serine/threonine phosphatase activity	PPP3R1|PPP3CA	0.0122	GO.0004723
2	GO Function	vascular endothelial growth factor-activated receptor activity	PDGFRA|FLT1	0.0122	GO.0005021
2	GO Function	T cell receptor binding	LCK|FYN	0.0149	GO.0042608
2	GO Function	CD4 receptor binding	LCK|FYN	0.0149	GO.0042609
3	GO Function	proteoglycan binding	THBS1|APP|ITGA2	0.015	GO.0043394
4	GO Function	phosphatidylinositol-4,5-bisphosphate 3-kinase activity	PDGFRA|KIT|LCK|FYN	0.015	GO.0046934
14	GO Function	oxidoreductase activity	PLOD1|CYP24A1|GAPDH|PXDN|PLOD2|NOS3|NQO1|CYP2R1|SNCA|GSTO1|PIR|G6PD|LDHB|PDIA6	0.0154	GO.0016491
6	GO Function	phosphatase binding	PPP3R1|CSF1R|LCK|CDH5|PTPN1|BCL2	0.0163	GO.0019902
9	GO Function	phosphoric ester hydrolase activity	PPP3R1|CDC25B|CCR5|CDC25A|TENC1|CDC25C|LCK|PTPN1|PPP3CA	0.0163	GO.0042578
5	GO Function	ion channel binding	CAV1|CDH5|FYN|FLNA|PRKCSH	0.0185	GO.0044325
33	GO Function	hydrolase activity	MYH9|MMP2|PPP3R1|CDC25B|ARF3|TUBB4A|CAD|CCR5|CAPN2|TUBA1A|TUBA1C|CDC25A|ARF4|MMP14|TENC1|CDC25C|LCK|TUBB4B|USP1|MYO1C|MYH10|DPP4|PTPN1|DNM1|ANXA1|XRCC5|MYO1B|PPP3CA|CDC42|TOP2B|ARF1|ABCB1|MCM3	0.0187	GO.0016787
5	GO Function	protein phosphatase binding	CSF1R|LCK|CDH5|PTPN1|BCL2	0.02	GO.0019903

<table>
<tr><th></th><th></th><th></th><th></th><th></th><th></th></tr>
<tr><td>4</td><td>GO Function</td><td>Rho guanyl-nucleotide exchange factor activity</td><td>ARHGEF5|NGEF|ARHGEF26|ARHGEF15</td><td>0.0203</td><td>GO.0005089</td></tr>
<tr><td>3</td><td>GO Function</td><td>peptidase activator activity</td><td>APP|CAV1|FN1</td><td>0.0203</td><td>GO.0016504</td></tr>
<tr><td>2</td><td>GO Function</td><td>calcium-dependent protein serine/threonine kinase activity</td><td>PRKCG|CAMK2A</td><td>0.0225</td><td>GO.0009931</td></tr>
<tr><td>2</td><td>GO Function</td><td>oxidoreductase activity, acting on NAD(P)H, heme protein as acceptor</td><td>NOS3|NQO1</td><td>0.0259</td><td>GO.0016653</td></tr>
<tr><td>2</td><td>GO Function</td><td>protein membrane anchor</td><td>MYH9|SUN1</td><td>0.0259</td><td>GO.0043495</td></tr>
<tr><td>8</td><td>GO Function</td><td>ATPase activity, coupled</td><td>MYH9|MYO1C|MYH10|ANXA1|XRCC5|MYO1B|TOP2B|ABCB1</td><td>0.027</td><td>GO.0042623</td></tr>
<tr><td>2</td><td>GO Function</td><td>peptide disulfide oxidoreductase activity</td><td>GSTO1|PDIA6</td><td>0.0288</td><td>GO.0015037</td></tr>
<tr><td>2</td><td>GO Function</td><td>cyclosporin A binding</td><td>PPP3R1|PPP3CA</td><td>0.0288</td><td>GO.0016018</td></tr>
<tr><td>2</td><td>GO Function</td><td>nitric-oxide synthase binding</td><td>CAV1|DNM1</td><td>0.0288</td><td>GO.0050998</td></tr>
<tr><td>3</td><td>GO Function</td><td>structural constituent of muscle</td><td>TPM4|TPM3|TPM2</td><td>0.0311</td><td>GO.0008307</td></tr>
<tr><td>2</td><td>GO Function</td><td>heparan sulfate proteoglycan binding</td><td>APP|ITGA2</td><td>0.0318</td><td>GO.0043395</td></tr>
<tr><td>3</td><td>GO Function</td><td>NADP binding</td><td>GAPDH|NOS3|G6PD</td><td>0.0323</td><td>GO.0050661</td></tr>
<tr><td>10</td><td>GO Function</td><td>cofactor binding</td><td>PLOD1|CYP24A1|GAPDH|PXDN|PLOD2|NOS3|CYP2R1|PTGES|G6PD|LDHB</td><td>0.0331</td><td>GO.0048037</td></tr>
<tr><td>7</td><td>GO Function</td><td>peptide binding</td><td>PPP3R1|APP|ITGA2|CALR|PTGES|PPP3CA|APBA2</td><td>0.0348</td><td>GO.0042277</td></tr>
<tr><td>7</td><td>GO Function</td><td>G protein-coupled receptor binding</td><td>ITGB4|APLP1|CCL2|APP|FYN|FLNA|DNM1</td><td>0.0359</td><td>GO.0001664</td></tr>
<tr><td>4</td><td>GO Function</td><td>phosphatidylinositol bisphosphate binding</td><td>PLEK|STXBP6|ANXA2|MYO1B</td><td>0.0363</td><td>GO.1902936</td></tr>
<tr><td>2</td><td>GO Function</td><td>protein kinase C activity</td><td>PRKCG|PRKCH</td><td>0.0382</td><td>GO.0004697</td></tr>
<tr><td>5</td><td>GO Function</td><td>oxidoreductase activity, acting on paired donors, with incorporation or reduction of molecular oxygen</td><td>PLOD1|CYP24A1|PLOD2|NOS3|CYP2R1</td><td>0.0382</td><td>GO.0016705</td></tr>
<tr><td>3</td><td>GO Function</td><td>extracellular matrix binding</td><td>THBS1|ITGA2|ANXA2</td><td>0.0402</td><td>GO.0050840</td></tr>
<tr><td>4</td><td>GO Function</td><td>protein tyrosine phosphatase activity</td><td>CDC25B|CDC25A|CDC25C|PTPN1</td><td>0.0433</td><td>GO.0004725</td></tr>
<tr><td>10</td><td>GO Function</td><td>enzyme activator activity</td><td>DNAJB6|ARHGAP31|APP|CAV1|RGS12|FN1|ARHGEF15|ARHGAP30|TBC1D4|XRCC5</td><td>0.0457</td><td>GO.0008047</td></tr>
<tr><td>16</td><td>GO Function</td><td>enzyme regulator activity</td><td>GAPDH|HSPB1|DNAJB6|ARHGAP31|APP|ANXA5|SNCA|CAV1|RGS12|ANXA2|FN1|ARHGEF15|ARHGAP30|ANXA1|TBC1D4|XRCC5</td><td>0.0469</td><td>GO.0030234</td></tr>
<tr><td>29</td><td>GO Function</td><td>transferase activity</td><td>MAP2K5|CCL2|GAPDH|PDGFRA|PRKCG|AKT3|CAD|FLT1|CSF1R|KIT|MELK|CKB|PLK1|BRSK1|PRKCH|LCK|MAP4K4|FYN|EPHA2|TGM2|GSTO1|SLK|CAMK2B|CAMK2A|DYRK1A|CDC42|WEE1|PBK|CAMK1D</td><td>0.0489</td><td>GO.0016740</td></tr>
<tr><td>70</td><td>GO Process</td><td>regulation of cellular component organization</td><td>ARHGEF5|MAP2K5|MYH9|EHD4|CCL2|PLEK|ENO1|PDGFRA|THBS1|DNAJB6|PRKCG|NGEF|TUBB4A|ADD1|ICAM1|TJP1|APP|CSF1R|KIT|CAPN2|ITGA2|CCT2|PLK1|DDB1|BCL2L1|MMP14|BRSK1|DAB2|CALR|CDC25C|STXBP6|PRKCH|SNCA|CAV1|MAP4K4|ANXA2|FYN|FN1|SFPQ|EPHA2|MYO1C|MYH10|DPP4|ARHGEF15|DLL1|SLK|FLNA|PTPN1|SDC4|SPTAN1|DNM1|TLR4|CLIC4|ANXA1|TBC1D4|SDC1|FSCN1|XRCC5|G6PD|PPP3CA|CAMK2B|BCL2|DYRK1A|SEPT7|CDC42|SPTBN2|CFL1|ARF1|VIM|CAMK1D</td><td>1.88E-19</td><td>GO.0051128</td></tr>
<tr><td>127</td><td>GO Process</td><td>response to stimulus</td><td>ARHGEF5|MAP2K5|PLOD1|ITGB4|MYH9|CYP24A1|MMP2|EHD4|APLP1|CCL2|GAPDH|GNB4|PPP3R1|PLEK|ENO1|MT2A|HSPB1|BCL2L2|PXDN|LAMA5|PDGFRA|THBS1|VWF|RORA|CNN2|PRKCG|AKT3|NGEF|ARHGAP31|CAD|ADD1|ICAM1|FERMT3|TJP1|FLT1|PLOD2|APP|CSF1R|KIT|NPC1L1|CCR5|CAPN2|ANXA5|ITGA2|NOS3|MELK|CCT2|PLK1|DDB1|BCL2L1|CDC25A|ARF4|MMP14|BRSK1|DAB2|TENC1|NQO1|CALR|CDC25C|ZYX|RORC|JAG2|PRKCH|RARB|CYP2R1|NR1I2|LCK|SNCA|CAV1|RGS12|TUBB4B|PTGES|USP1|MAP4K4|CDH5|DOK4|ANXA2|ANXA6|FYN|FN1|FAS|ARHGEF26|SFPQ|EPHA2|MYO1C|RXRG|MYH10|DPP4|TGM2|DLL1|ARHGAP30|GSTO1|SLK|FLNA|PTPN1|SDC4|SPTAN1|DNM1|TLR4|CLIC4|RXRB|ANXA1|TBC1D4|PSIP1|SDC1|FSCN1|XRCC5|G6PD|PPP3CA|CAMK2B|LTB4R|BCL2|CAMK2A|CDC42|SUN1|PDIA6|RARG|FLNB|PBK|SPTBN2|CFL1|ARF1|VIM|NR1H4|PRKCSH|ABCB1|CAMK1D</td><td>5.52E-19</td><td>GO.0050896</td></tr>
<tr><td>113</td><td>GO Process</td><td>cellular response to stimulus</td><td>ARHGEF5|MAP2K5|ITGB4|MYH9|CYP24A1|MMP2|EHD4|APLP1|CCL2|GAPDH|GNB4|PPP3R1|PLEK|MT2A|HSPB1|BCL2L2|PXDN|LAMA5|PDGFRA|THBS1|RORA|CNN2|PRKCG|AKT3|NGEF|ARHGAP31|CAD|ADD1|ICAM1|FERMT3|TJP1|FLT1|APP|CSF1R|KIT|NPC1L1|CCR5|CAPN2|ANXA5|ITGA2|NOS3|MELK|PLK1|DDB1|BCL2L1|CDC25A|ARF4|BRSK1|DAB2|TENC1|NQO1|CALR|CDC25C|ZYX|RORC|JAG2|PRKCH|RARB|NR1I2|LCK|SNCA|CAV1|RGS12|PTGES|USP1|MAP4K4|CDH5|ANXA2|ANXA6|FYN|FN1|FAS|ARHGEF26|SFPQ|EPHA2|MYO1C|RXRG|TGM2|DLL1|ARHGAP30|GSTO1|SLK|FLNA|PTPN1|SDC4|SPTAN1|DNM1|TLR4|CLIC4|RXRB|ANXA1|TBC1D4|SDC1|FSCN1|XRCC5|G6PD|PPP3CA|CAMK2B|LTB4R|BCL2|CAMK2A|CDC42|PDIA6|RARG|FLNB|PBK|SPTBN2|CFL1|ARF1|VIM|NR1H4|PRKCSH|CAMK1D</td><td>5.52E-19</td><td>GO.0051716</td></tr>
<tr><td>96</td><td>GO Process</td><td>signal transduction</td><td>ARHGEF5|MAP2K5|ITGB4|MYH9|CYP24A1|MMP2|CCL2|GNB4|PPP3R1|PLEK|MT2A|HSPB1|BCL2L2|LAMA5|PDGFRA|RORA|CNN2|PRKCG|AKT3|NGEF|ARHGAP31|ADD1|ICAM1|FERMT3|TJP1|FLT1|APP|CSF1R|KIT|CCR5|ANXA5|ITGA2|NOS3|MELK|PLK1|DDB1|BCL2L1|ARF4|BRSK1|DAB2|TENC1|CALR|CDC25C|ZYX|RORC|JAG2|PRKCH|RARB|NR1I2|LCK|SNCA|CAV1|RGS12|PTGES|MAP4K4|CDH5|ANXA2|ANXA6|FYN|FN1|FAS|ARHGEF26|EPHA2|MYO1C|RXRG|TGM2|DLL1|ARHGAP30|GSTO1|SLK|FLNA|PTPN1|SDC4|SPTAN1|DNM1|TLR4|RXRB|ANXA1|SDC1|FSCN1|PPP3CA|CAMK2B|LTB4R|BCL2|CAMK2A|CDC42|PDIA6|RARG|FLNB|SPTBN2|CFL1|ARF1|VIM|NR1H4|PRKCSH|CAMK1D</td><td>1.40E-17</td><td>GO.0007165</td></tr>
<tr><td>98</td><td>GO Process</td><td>signaling</td><td>ARHGEF5|MAP2K5|ITGB4|MYH9|CYP24A1|MMP2|CCL2|GNB4|PPP3R1|PLEK|MT2A|HSPB1|BCL2L2|LAMA5|PDGFRA|RORA|CNN2|PRKCG|AKT3|NGEF|ARHGAP31|ADD1|ICAM1|FERMT3|TJP1|FLT1|APP|CSF1R|KIT|CCR5|ANXA5|ITGA2|NOS3|MELK|PLK1|DDB1|BCL2L1|ARF4|BRSK1|DAB2|TENC1|NQO1|CALR|CDC25C|ZYX|RORC|JAG2|PRKCH|RARB|NR1I2|LCK|SNCA|CAV1|RGS12|PTGES|MAP4K4|CDH5|ANXA2|ANXA6|FYN|FN1|FAS|ARHGEF26|EPHA2|MYO1C|RXRG|TGM2|DLL1|ARHGAP30|GSTO1|SLK|FLNA|PTPN1|SDC4|SPTAN1|DNM1|TLR4|RXRB|ANXA1|SDC1|FSCN1|PPP3CA|CAMK2B|LTB4R|BCL2|CAMK2A|CDC42|PDIA6|RARG|FLNB|SPTBN2|CFL1|ARF1|VIM|NR1H4|APBA2|PRKCSH|CAMK1D</td><td>1.61E-16</td><td>GO.0023052</td></tr>
<tr><td>98</td><td>GO Process</td><td>cell communication</td><td>ARHGEF5|MAP2K5|ITGB4|MYH9|CYP24A1|MMP2|CCL2|GNB4|PPP3R1|PLEK|MT2A|HSPB1|BCL2L2|LAMA5|PDGFRA|RORA|CNN2|PRKCG|AKT3|NGEF|ARHGAP31|ADD1|ICAM1|FERMT3|TJP1|FLT1|APP|CSF1R|KIT|CCR5|ANXA5|ITGA2|NOS3|MELK|PLK1|DDB1|BCL2L1|ARF4|BRSK1|DAB2|TENC1|NQO1|CALR|CDC25C|ZYX|RORC|JAG2|PRKCH|RARB|NR1I2|LCK|SNCA|CAV1|RGS12|PTGES|MAP4K4|CDH5|ANXA2|ANXA6|FYN|FN1|FAS|ARHGEF26|EPHA2|MYO1C|RXRG|TGM2|DLL1|ARHGAP30|GSTO1|SLK|FLNA|PTPN1|SDC4|SPTAN1|DNM1|TLR4|RXRB|ANXA1|SDC1|FSCN1|PPP3CA|CAMK2B|LTB4R|BCL2|CAMK2A|CDC42|PDIA6|RARG|FLNB|SPTBN2|CFL1|ARF1|VIM|NR1H4|APBA2|PRKCSH|CAMK1D</td><td>6.56E-16</td><td>GO.0007154</td></tr>
<tr><td>66</td><td>GO Process</td><td>regulation of localization</td><td>ARHGEF5|MAP2K5|EHD4|CCL2|GAPDH|LAMA4|HSPB1|LAMA5|PDGFRA|THBS1|DNAJB6|PRKCG|AKT3|ICAM1|FERMT3|FLT1|APP|CSF1R|KIT|CCR5|ITGA2|NOS3|CCT2|PLK1|BCL2L1|MMP14|BRSK1|DAB2|CALR|STXBP6|PRKCH|LCK|SNCA|CAV1|MAP4K4|ANXA2|FYN|FN1|PTRF|EPHA2|MYO1C|MYH10|DPP4|DLL1|GSTO1|SLK|FLNA|PTPN1|MMRN2|SDC4|DNM1|TLR4|CLIC4|ANXA1|TBC1D4|SDC1|G6PD|PPP3CA|CAMK2B|BCL2|CAMK2A|ARF1|NR1H4|NUMB|ABCB1|CAMK1D</td><td>2.01E-15</td><td>GO.0032879</td></tr>
<tr><td>68</td><td>GO Process</td><td>cellular response to chemical stimulus</td><td>ARHGEF5|MAP2K5|CYP24A1|MMP2|EHD4|APLP1|CCL2|GAPDH|MT2A|HSPB1|PXDN|LAMA5|PDGFRA|THBS1|RORA|CNN2|CAD|ADD1|ICAM1|FLT1|APP|CSF1R|KIT|CCR5|CAPN2|ITGA2|NOS3|MELK|BCL2L1|NQO1|CALR|ZYX|RORC|RARB|NR1I2|SNCA|CAV1|CDH5|ANXA2|FYN|FN1|FAS|EPHA2|MYO1C|RXRG|GSTO1|PTPN1|TLR4|CLIC4|RXRB|ANXA1|TBC1D4|SDC1|FSCN1|XRCC5|G6PD|PPP3CA|CAMK2B|BCL2|CAMK2A|CDC42|PDIA6|RARG|FLNB|CFL1|ARF1|VIM|NR1H4</td><td>2.01E-15</td><td>GO.0070887</td></tr>
</table>

<table>
<tr><td>75</td><td>GO Process</td><td>response to stress</td><td>MAP2K5|PLOD1|ITGB4|MMP2|CCL2|GAPDH|MT2A|HSPB1|BCL2L2|PXDN|PDGFRA|THBS1|VWF|RORA|PRKCG|CAD|ADD1|ICAM1|PLOD2|APP|CSF1R|KIT|NPC1L1|CCR5|CAPN2|ANXA5|ITGA2|NOS3|MELK|PLK1|DDB1|BCL2L1|ARF4|MMP14|BRSK1|NQO1|CALR|CDC25C|ZYX|PRKCH|LCK|SNCA|CAV1|TUBB4B|PTGES|USP1|FYN|FN1|FAS|SFPQ|EPHA2|MYO1C|MYH10|DPP4|SLK|FLNA|PTPN1|SDC4|TLR4|ANXA1|PSIP1|SDC1|XRCC5|G6PD|PPP3CA|CAMK2B|LTB4R|BCL2|CAMK2A|CDC42|PDIA6|FLNB|VIM|NR1H4|CAMK1D</td><td>3.17E-15</td><td>GO.0006950</td></tr>
<tr><td>96</td><td>GO Process</td><td>cellular component organization</td><td>ARHGEF5|ITGB4|MYH9|MMP2|EHD4|CCL2|GAPDH|LAMA4|PLEK|CDC25B|PXDN|LAMA5|PDGFRA|THBS1|COL8A1|VWF|DNAJB6|CNN2|AKT3|TUBB4A|ADD1|ICAM1|FERMT3|TJP1|APP|CSF1R|KIT|CCR5|ITGA2|NOS3|CCT2|PLK1|TUBA1A|TUBA1C|DDB1|BCL2L1|SDPR|ARF4|MMP14|BRSK1|DAB2|CALR|ZYX|FLNC|SNCA|CAV1|TUBB4B|MAP4K4|CDH5|DOK4|PTMA|TPM4|ANXA2|ANXA6|FYN|FN1|FAS|ARHGEF26|SFPQ|EPHA2|MYO1C|RXRG|MYH10|TGM2|TPM3|SLK|FLNA|CNN3|PTPN1|SDC4|SPTAN1|DNM1|CLIC4|ANXA1|TPM2|PSIP1|FSCN1|XRCC5|MYO1B|BCL2|CAMK2A|SEPT7|CDC42|SUN1|RARG|TOP2B|WEE1|FLNB|SPTBN2|CFL1|ARF1|VIM|NR1H4|NUMB|TLN2|ABCB1</td><td>3.17E-15</td><td>GO.0016043</td></tr>
<tr><td>69</td><td>GO Process</td><td>response to organic substance</td><td>MAP2K5|CYP24A1|MMP2|EHD4|APLP1|CCL2|GAPDH|MT2A|HSPB1|LAMA5|PDGFRA|THBS1|RORA|CNN2|PRKCG|CAD|ADD1|ICAM1|FLT1|APP|CSF1R|KIT|CCR5|CAPN2|ANXA5|ITGA2|NOS3|BCL2L1|MMP14|NQO1|CALR|ZYX|RORC|RARB|NR1I2|SNCA|CAV1|PTGES|CDH5|ANXA2|FYN|FN1|FAS|EPHA2|MYO1C|RXRG|GSTO1|PTPN1|DNM1|TLR4|RXRB|ANXA1|TBC1D4|SDC1|FSCN1|XRCC5|G6PD|PPP3CA|CAMK2B|BCL2|CAMK2A|CDC42|PDIA6|RARG|FLNB|CFL1|ARF1|VIM|NR1H4</td><td>4.53E-15</td><td>GO.0010033</td></tr>
<tr><td>61</td><td>GO Process</td><td>cellular response to organic substance</td><td>MAP2K5|CYP24A1|MMP2|EHD4|APLP1|CCL2|GAPDH|MT2A|HSPB1|LAMA5|PDGFRA|THBS1|RORA|CNN2|CAD|ADD1|ICAM1|FLT1|APP|CSF1R|KIT|CCR5|CAPN2|ITGA2|NOS3|BCL2L1|CALR|ZYX|RORC|RARB|NR1I2|SNCA|CAV1|CDH5|ANXA2|FYN|FN1|FAS|MYO1C|RXRG|GSTO1|PTPN1|TLR4|RXRB|ANXA1|TBC1D4|SDC1|FSCN1|XRCC5|PPP3CA|CAMK2B|BCL2|CAMK2A|CDC42|PDIA6|RARG|FLNB|CFL1|ARF1|VIM|NR1H4</td><td>4.53E-15</td><td>GO.0071310</td></tr>
<tr><td>50</td><td>GO Process</td><td>regulation of programmed cell death</td><td>ARHGEF5|MAP2K5|CCL2|GAPDH|ENO1|HSPB1|BCL2L2|THBS1|DNAJB6|PRKCG|NGEF|ICAM1|APP|CSF1R|KIT|CCR5|ANXA5|NOS3|MELK|PLK1|DDB1|BCL2L1|ARF4|DAB2|NQO1|CALR|RORC|PRKCH|RARB|LCK|SNCA|CAV1|MAP4K4|FYN|FAS|ARHGEF26|SFPQ|TGM2|DLL1|SLK|FLNA|PTPN1|ANXA1|G6PD|BCL2|CAMK2A|RARG|CFL1|NR1H4|CAMK1D</td><td>8.45E-15</td><td>GO.0043067</td></tr>
<tr><td>40</td><td>GO Process</td><td>cytoskeleton organization</td><td>ARHGEF5|MYH9|CCL2|GAPDH|PLEK|LAMA5|PDGFRA|DNAJB6|CNN2|TUBB4A|ADD1|KIT|PLK1|TUBA1A|TUBA1C|BRSK1|CALR|ZYX|TUBB4B|TPM4|MYH10|TPM3|SLK|FLNA|CNN3|PTPN1|SPTAN1|ANXA1|TPM2|FSCN1|MYO1B|BCL2|CDC42|SUN1|WEE1|FLNB|SPTBN2|CFL1|VIM|TLN2</td><td>1.13E-14</td><td>GO.0007010</td></tr>
<tr><td>49</td><td>GO Process</td><td>regulation of apoptotic process</td><td>ARHGEF5|MAP2K5|CCL2|GAPDH|ENO1|HSPB1|BCL2L2|THBS1|DNAJB6|PRKCG|NGEF|ICAM1|APP|CSF1R|CCR5|ANXA5|NOS3|MELK|PLK1|DDB1|BCL2L1|ARF4|DAB2|NQO1|CALR|RORC|PRKCH|RARB|LCK|SNCA|CAV1|MAP4K4|FYN|FAS|ARHGEF26|SFPQ|TGM2|DLL1|SLK|FLNA|PTPN1|ANXA1|G6PD|BCL2|CAMK2A|RARG|CFL1|NR1H4|CAMK1D</td><td>2.37E-14</td><td>GO.0042981</td></tr>
<tr><td>51</td><td>GO Process</td><td>regulation of cell death</td><td>ARHGEF5|MAP2K5|CCL2|GAPDH|ENO1|HSPB1|BCL2L2|THBS1|DNAJB6|PRKCG|NGEF|ICAM1|APP|CSF1R|KIT|CCR5|ANXA5|NOS3|MELK|PLK1|DDB1|BCL2L1|ARF4|DAB2|NQO1|CALR|RORC|PRKCH|RARB|LCK|SNCA|CAV1|MAP4K4|FYN|FAS|ARHGEF26|SFPQ|TGM2|DLL1|SLK|FLNA|PTPN1|TLR4|ANXA1|G6PD|BCL2|CAMK2A|RARG|CFL1|NR1H4|CAMK1D</td><td>2.86E-14</td><td>GO.0010941</td></tr>
<tr><td>59</td><td>GO Process</td><td>cell surface receptor signaling pathway</td><td>ITGB4|MYH9|MMP2|CCL2|PPP3R1|PLEK|MT2A|HSPB1|BCL2L2|LAMA5|PDGFRA|RORA|CNN2|NGEF|ICAM1|FERMT3|FLT1|APP|CSF1R|KIT|CCR5|ITGA2|NOS3|DDB1|BCL2L1|ARF4|DAB2|ZYX|RORC|JAG2|LCK|SNCA|CAV1|CDH5|ANXA2|FYN|FN1|FAS|EPHA2|MYO1C|DLL1|GSTO1|FLNA|PTPN1|DNM1|TLR4|ANXA1|SDC1|FSCN1|PPP3CA|CAMK2B|BCL2|CAMK2A|CDC42|RARG|CFL1|ARF1|VIM|NR1H4</td><td>3.66E-14</td><td>GO.0007166</td></tr>
<tr><td>37</td><td>GO Process</td><td>negative regulation of programmed cell death</td><td>MAP2K5|CCL2|ENO1|HSPB1|BCL2L2|THBS1|DNAJB6|PRKCG|ICAM1|CSF1R|KIT|CCR5|ANXA5|NOS3|PLK1|DDB1|BCL2L1|ARF4|DAB2|NQO1|RORC|PRKCH|RARB|SNCA|CAV1|MAP4K4|FYN|FAS|DLL1|FLNA|PTPN1|ANXA1|BCL2|RARG|CFL1|NR1H4|CAMK1D</td><td>1.20E-13</td><td>GO.0043069</td></tr>
<tr><td>85</td><td>GO Process</td><td>negative regulation of cellular process</td><td>MAP2K5|MYH9|APLP1|CCL2|GAPDH|PLEK|ENO1|HSPB1|BCL2L2|PXDN|PDGFRA|THBS1|RORA|DNAJB6|PRKCG|AKT3|NGEF|TUBB4A|ADD1|ICAM1|FLT1|APP|CSF1R|KIT|CCR5|ANXA5|NOS3|PLK1|DDB1|BCL2L1|ARF4|MMP14|BRSK1|DAB2|TENC1|NQO1|CALR|CDC25C|STXBP6|RORC|PRKCH|RARB|NR1I2|LCK|SNCA|CAV1|RGS12|PTGES|MAP4K4|CDH5|ANXA2|FYN|FN1|FAS|SFPQ|EPHA2|DPP4|ARHGEF15|DLL1|GSTO1|FLNA|PTPN1|MMRN2|SDC4|SPTAN1|DNM1|TLR4|CLIC4|ANXA1|TBC1D4|XRCC5|G6PD|PPP3CA|BCL2|DYRK1A|CDC42|RARG|TOP2B|PBK|SPTBN2|CFL1|ARF1|VIM|NR1H4|CAMK1D</td><td>1.72E-13</td><td>GO.0048523</td></tr>
<tr><td>75</td><td>GO Process</td><td>regulation of biological quality</td><td>MYH9|CCL2|GAPDH|RBP1|PLEK|MT2A|HSPB1|PDGFRA|THBS1|VWF|RORA|PRKCG|AKT3|NGEF|CAD|ADD1|ICAM1|APP|CSF1R|KIT|CCR5|ANXA5|ITGA2|NOS3|CKB|CCT2|PLK1|DDB1|BCL2L1|BRSK1|TENC1|NQO1|CALR|PRKCH|CYP2R1|LCK|SNCA|CAV1|MAP4K4|ANXA2|ANXA6|FYN|FN1|MYO1C|MYH10|DPP4|ARHGEF15|TGM2|DLL1|COPA|GSTO1|FLNA|SPTAN1|TLR4|CLIC4|ANXA1|XRCC5|G6PD|PPP3CA|CAMK2B|BCL2|CAMK2A|SEPT7|CDC42|SUN1|PDIA6|RARG|FLNB|SPTBN2|CFL1|ARF1|VIM|NR1H4|TLN2|ABCB1</td><td>1.72E-13</td><td>GO.0065008</td></tr>
<tr><td>145</td><td>GO Process</td><td>biological regulation</td><td>ARHGEF5|MAP2K5|ITGB4|MYH9|CYP24A1|MMP2|EHD4|APLP1|CCL2|GAPDH|LAMA4|RBP1|GNB4|PPP3R1|PLEK|ENO1|MT2A|CDC25B|HSPB1|BCL2L2|PXDN|LAMA5|PDGFRA|THBS1|COL8A1|VWF|RORA|DNAJB6|CNN2|PRKCG|AKT3|NGEF|TUBB4A|ARHGAP31|CAD|ADD1|ICAM1|FERMT3|TJP1|FLT1|APP|CSF1R|KIT|CCR5|CAPN2|ANXA5|ITGA2|NOS3|MELK|CKB|CCT2|PLK1|TUBA1A|DDB1|BCL2L1|CDC25A|ARF4|MMP14|BRSK1|DAB2|TENC1|NQO1|CALR|CDC25C|STXBP6|ZYX|RORC|JAG2|PRKCH|RARB|CYP2R1|NR1I2|LCK|SNCA|CAV1|RGS12|TUBB4B|PTGES|USP1|MAP4K4|CDH5|ANXA2|ANXA6|FYN|FN1|FAS|ARHGEF26|PTRF|SFPQ|EPHA2|MYO1C|RXRG|MYH10|DPP4|ARHGEF15|TGM2|DLL1|ARHGAP30|COPA|GSTO1|SLK|FLNA|CNN3|PTPN1|MMRN2|SDC4|SPTAN1|DNM1|TLR4|CLIC4|RXRB|ANXA1|TBC1D4|TPM2|PIR|PSIP1|SDC1|FSCN1|XRCC5|G6PD|PPP3CA|CAMK2B|LTB4R|BCL2|CAMK2A|DYRK1A|SEPT7|CDC42|SUN1|PDIA6|RARG|TOP2B|FLNB|PBK|SPTBN2|CFL1|ARF1|VIM|NR1H4|NUMB|APBA2|TLN2|PRKCSH|ABCB1|CAMK1D</td><td>1.94E-13</td><td>GO.0065007</td></tr>
</table>

<table>
<tr><td>160</td><td>GO Process</td><td>cellular process</td><td>RCN1|ARHGEF5|MAP2K5|PLOD1|ITGB4|MYH9|CYP24A1|MMP2|EHD4|APLP1|CCL2|GAPDH|LAMA4|RBP1|GNB4|PPP3R1|PLEK|ENO1|MT2A|CDC25B|HSPB1|BCL2L2|PXDN|LAMA5|ARF3|PDGFRA|THBS1|COL8A1|VWF|RORA|DNAJB6|CNN2|PRKCG|AKT3|NGEF|TUBB4A|ARHGAP31|CAD|ADD1|ICAM1|EXOC6B|FERMT3|TJP1|FLT1|PLOD2|APP|CSF1R|KIT|NPC1L1|CCR5|CAPN2|ANXA5|ITGA2|NOS3|MELK|CKB|CCT2|PLK1|TUBA1A|TUBA1C|DDB1|BCL2L1|CDC25A|SDPR|ARF4|MMP14|BRSK1|DAB2|TENC1|NQO1|CALR|CDC25C|ZYX|RORC|FLNC|JAG2|PRKCH|RARB|CYP2R1|NR1I2|LCK|SNCA|CAV1|RGS12|TUBB4B|PTGES|USP1|MAP4K4|CDH5|DOK4|PTMA|TPM4|ANXA2|ANXA6|FYN|FN1|FAS|ARHGEF26|PTRF|SFPQ|EPHA2|MYO1C|RXRG|MYH10|DPP4|TGM2|DLL1|ARHGAP30|TPM3|GSTO1|SLK|FLNA|CNN3|PTPN1|MMRN2|SDC4|SPTAN1|DNM1|TLR4|CLIC4|RXRB|ANXA1|TBC1D4|TPM2|PIR|PSIP1|SDC1|FSCN1|XRCC5|MYO1B|G6PD|PPP3CA|CAMK2B|LDHB|LTB4R|BCL2|CAMK2A|DYRK1A|SEPT7|PAICS|CDC42|SUN1|PDIA6|RARG|TOP2B|WEE1|FLNB|PBK|SPTBN2|CFL1|ARF1|VIM|NR1H4|NUMB|APBA2|TLN2|PRKCSH|ABCB1|CAMK1D|MCM3</td><td>2.27E-13</td><td>GO.0009987</td></tr>
<tr><td>38</td><td>GO Process</td><td>negative regulation of cell death</td><td>MAP2K5|CCL2|ENO1|HSPB1|BCL2L2|THBS1|DNAJB6|PRKCG|ICAM1|APP|CSF1R|KIT|CCR5|ANXA5|NOS3|PLK1|DDB1|BCL2L1|ARF4|DAB2|NQO1|RORC|PRKCH|RARB|SNCA|CAV1|MAP4K4|FYN|FAS|DLL1|FLNA|PTPN1|ANXA1|BCL2|RARG|CFL1|NR1H4|CAMK1D</td><td>2.37E-13</td><td>GO.0060548</td></tr>
<tr><td>89</td><td>GO Process</td><td>positive regulation of cellular process</td><td>ARHGEF5|MAP2K5|MYH9|MMP2|EHD4|CCL2|GAPDH|PPP3R1|PLEK|ENO1|CDC25B|HSPB1|PDGFRA|THBS1|RORA|PRKCG|AKT3|NGEF|ADD1|ICAM1|FERMT3|FLT1|APP|CSF1R|KIT|ITGA2|NOS3|MELK|CCT2|PLK1|DDB1|BCL2L1|CDC25A|ARF4|MMP14|DAB2|NQO1|CALR|CDC25C|RORC|JAG2|PRKCH|RARB|NR1I2|LCK|SNCA|CAV1|MAP4K4|CDH5|ANXA2|FYN|FN1|FAS|ARHGEF26|PTRF|SFPQ|MYO1C|RXRG|MYH10|DPP4|ARHGEF15|TGM2|DLL1|GSTO1|SLK|FLNA|PTPN1|SDC4|DNM1|TLR4|RXRB|ANXA1|PSIP1|SDC1|FSCN1|XRCC5|PPP3CA|CAMK2B|BCL2|CAMK2A|DYRK1A|SEPT7|CDC42|RARG|CFL1|VIM|NR1H4|NUMB|CAMK1D</td><td>3.19E-13</td><td>GO.0048522</td></tr>
<tr><td>94</td><td>GO Process</td><td>developmental process</td><td>MAP2K5|PLOD1|ITGB4|MYH9|CYP24A1|MMP2|APLP1|CCL2|PPP3R1|PLEK|CDC25B|BCL2L2|LAMA5|PDGFRA|THBS1|COL8A1|RORA|PRKCG|AKT3|NGEF|CAD|ADD1|ICAM1|FERMT3|TJP1|FLT1|APP|CSF1R|KIT|CAPN2|ITGA2|NOS3|MELK|CKB|BCL2L1|ARF4|MMP14|BRSK1|DAB2|TENC1|NQO1|CALR|RORC|FLNC|JAG2|PRKCH|RARB|LCK|SNCA|CAV1|USP1|MAP4K4|CDH5|DOK4|ANXA2|FYN|FN1|FAS|ARHGEF26|EPHA2|MYH10|ARHGEF15|TGM2|DLL1|FLNA|CNN3|MMRN2|SDC4|SPTAN1|CLIC4|ANXA1|PIR|SDC1|FSCN1|XRCC5|G6PD|PPP3CA|CAMK2B|BCL2|CAMK2A|DYRK1A|SEPT7|CDC42|SUN1|RARG|TOP2B|WEE1|FLNB|SPTBN2|CFL1|VIM|NUMB|APBA2|CAMK1D</td><td>3.27E-13</td><td>GO.0032502</td></tr>
<tr><td>81</td><td>GO Process</td><td>response to chemical</td><td>ARHGEF5|MAP2K5|CYP24A1|MMP2|EHD4|APLP1|CCL2|GAPDH|MT2A|HSPB1|PXDN|LAMA5|PDGFRA|THBS1|RORA|CNN2|PRKCG|CAD|ADD1|ICAM1|FLT1|APP|CSF1R|KIT|NPC1L1|CCR5|CAPN2|ANXA5|ITGA2|NOS3|MELK|BCL2L1|MMP14|NQO1|CALR|ZYX|RORC|RARB|CYP2R1|NR1I2|LCK|SNCA|CAV1|PTGES|CDH5|DOK4|ANXA2|FYN|FN1|FAS|EPHA2|MYO1C|RXRG|MYH10|GSTO1|PTPN1|SPTAN1|DNM1|TLR4|CLIC4|RXRB|ANXA1|TBC1D4|SDC1|FSCN1|XRCC5|G6PD|PPP3CA|CAMK2B|BCL2|CAMK2A|CDC42|PDIA6|RARG|FLNB|SPTBN2|CFL1|ARF1|VIM|NR1H4|ABCB1</td><td>3.27E-13</td><td>GO.0042221</td></tr>
<tr><td>36</td><td>GO Process</td><td>negative regulation of apoptotic process</td><td>MAP2K5|CCL2|ENO1|HSPB1|BCL2L2|THBS1|DNAJB6|PRKCG|ICAM1|CSF1R|CCR5|ANXA5|NOS3|PLK1|DDB1|BCL2L1|ARF4|DAB2|NQO1|RORC|PRKCH|RARB|SNCA|CAV1|MAP4K4|FYN|FAS|DLL1|FLNA|PTPN1|ANXA1|BCL2|RARG|CFL1|NR1H4|CAMK1D</td><td>3.27E-13</td><td>GO.0043066</td></tr>
<tr><td>54</td><td>GO Process</td><td>anatomical structure morphogenesis</td><td>ITGB4|MYH9|MMP2|APLP1|CCL2|PPP3R1|LAMA5|PDGFRA|THBS1|COL8A1|RORA|AKT3|ADD1|FERMT3|FLT1|APP|CSF1R|CAPN2|ITGA2|NOS3|BCL2L1|MMP14|BRSK1|JAG2|RARB|CAV1|MAP4K4|DOK4|ANXA2|FYN|FN1|ARHGEF26|EPHA2|MYH10|ARHGEF15|TGM2|DLL1|FLNA|MMRN2|SDC4|SPTAN1|CLIC4|SDC1|FSCN1|BCL2|CAMK2A|CDC42|RARG|TOP2B|WEE1|FLNB|SPTBN2|CFL1|NUMB</td><td>5.02E-13</td><td>GO.0009653</td></tr>
<tr><td>90</td><td>GO Process</td><td>anatomical structure development</td><td>MAP2K5|PLOD1|ITGB4|MYH9|MMP2|APLP1|CCL2|PPP3R1|PLEK|CDC25B|BCL2L2|LAMA5|PDGFRA|THBS1|COL8A1|RORA|PRKCG|AKT3|NGEF|CAD|ADD1|ICAM1|FERMT3|TJP1|FLT1|APP|CSF1R|KIT|CAPN2|ITGA2|NOS3|MELK|CKB|BCL2L1|ARF4|MMP14|BRSK1|DAB2|TENC1|CALR|RORC|FLNC|JAG2|PRKCH|RARB|LCK|CAV1|USP1|MAP4K4|CDH5|DOK4|ANXA2|FYN|FN1|FAS|ARHGEF26|EPHA2|MYH10|ARHGEF15|TGM2|DLL1|FLNA|CNN3|MMRN2|SDC4|SPTAN1|CLIC4|ANXA1|PIR|SDC1|FSCN1|XRCC5|G6PD|PPP3CA|CAMK2B|BCL2|CAMK2A|DYRK1A|CDC42|SUN1|RARG|TOP2B|WEE1|FLNB|SPTBN2|CFL1|VIM|NUMB|APBA2|CAMK1D</td><td>8.31E-13</td><td>GO.0048856</td></tr>
<tr><td>44</td><td>GO Process</td><td>movement of cell or subcellular component</td><td>ARHGEF5|ITGB4|MYH9|CCL2|HSPB1|LAMA5|PDGFRA|THBS1|ICAM1|FLT1|APP|CSF1R|KIT|CCR5|ITGA2|NOS3|ARF4|MMP14|LCK|CAV1|DOK4|TPM4|FYN|FN1|EPHA2|MYO1C|MYH10|DPP4|TPM3|FLNA|MMRN2|SDC4|SPTAN1|ANXA1|TPM2|SDC1|FSCN1|MYO1B|CDC42|SUN1|TOP2B|SPTBN2|CFL1|VIM</td><td>8.59E-13</td><td>GO.0006928</td></tr>
<tr><td>93</td><td>GO Process</td><td>positive regulation of biological process</td><td>ARHGEF5|MAP2K5|MYH9|MMP2|EHD4|CCL2|GAPDH|PPP3R1|PLEK|ENO1|CDC25B|HSPB1|PDGFRA|THBS1|COL8A1|RORA|PRKCG|AKT3|NGEF|ADD1|ICAM1|FERMT3|FLT1|APP|CSF1R|KIT|ITGA2|NOS3|MELK|CCT2|PLK1|DDB1|BCL2L1|CDC25A|ARF4|MMP14|DAB2|NQO1|CALR|CDC25C|RORC|JAG2|PRKCH|RARB|NR1I2|LCK|SNCA|CAV1|MAP4K4|CDH5|ANXA2|FYN|FN1|FAS|ARHGEF26|PTRF|SFPQ|EPHA2|MYO1C|RXRG|MYH10|DPP4|ARHGEF15|TGM2|DLL1|GSTO1|SLK|FLNA|PTPN1|SDC4|DNM1|TLR4|RXRB|ANXA1|PSIP1|SDC1|FSCN1|XRCC5|G6PD|PPP3CA|CAMK2B|BCL2|CAMK2A|DYRK1A|SEPT7|CDC42|RARG|CFL1|VIM|NR1H4|NUMB|ABCB1|CAMK1D</td><td>1.92E-12</td><td>GO.0048518</td></tr>
<tr><td>79</td><td>GO Process</td><td>system development</td><td>MAP2K5|ITGB4|MYH9|MMP2|APLP1|CCL2|PPP3R1|PLEK|BCL2L2|LAMA5|PDGFRA|THBS1|COL8A1|RORA|PRKCG|AKT3|NGEF|CAD|ADD1|ICAM1|FLT1|APP|CSF1R|KIT|ITGA2|NOS3|MELK|CKB|BCL2L1|ARF4|MMP14|BRSK1|TENC1|CALR|RORC|JAG2|PRKCH|RARB|LCK|CAV1|USP1|MAP4K4|CDH5|DOK4|ANXA2|FYN|FN1|EPHA2|MYH10|ARHGEF15|TGM2|DLL1|FLNA|MMRN2|SDC4|SPTAN1|CLIC4|ANXA1|PIR|SDC1|XRCC5|G6PD|PPP3CA|CAMK2B|BCL2|CAMK2A|DYRK1A|CDC42|SUN1|RARG|TOP2B|WEE1|FLNB|SPTBN2|CFL1|VIM|NUMB|APBA2|CAMK1D</td><td>2.97E-12</td><td>GO.0048731</td></tr>
<tr><td>71</td><td>GO Process</td><td>cell differentiation</td><td>ITGB4|MYH9|CYP24A1|MMP2|CCL2|PPP3R1|PLEK|CDC25B|LAMA5|PDGFRA|COL8A1|RORA|NGEF|ADD1|ICAM1|FERMT3|TJP1|FLT1|APP|CSF1R|KIT|CAPN2|ITGA2|BCL2L1|ARF4|MMP14|BRSK1|DAB2|CALR|RORC|FLNC|JAG2|PRKCH|RARB|LCK|CAV1|MAP4K4|DOK4|ANXA2|FYN|FN1|ARHGEF26|EPHA2|MYH10|DLL1|FLNA|CNN3|SDC4|SPTAN1|CLIC4|ANXA1|PIR|SDC1|XRCC5|G6PD|PPP3CA|CAMK2B|BCL2|CAMK2A|SEPT7|CDC42|SUN1|RARG|TOP2B|WEE1|FLNB|SPTBN2|CFL1|VIM|NUMB|CAMK1D</td><td>4.02E-12</td><td>GO.0030154</td></tr>
<tr><td>87</td><td>GO Process</td><td>negative regulation of biological process</td><td>MAP2K5|MYH9|APLP1|CCL2|GAPDH|PLEK|ENO1|MT2A|HSPB1|BCL2L2|PXDN|PDGFRA|THBS1|RORA|DNAJB6|PRKCG|AKT3|NGEF|TUBB4A|ADD1|ICAM1|FLT1|APP|CSF1R|KIT|CCR5|ANXA5|NOS3|PLK1|DDB1|BCL2L1|ARF4|MMP14|BRSK1|DAB2|TENC1|NQO1|CALR|CDC25C|STXBP6|RORC|PRKCH|RARB|NR1I2|LCK|SNCA|CAV1|RGS12|PTGES|MAP4K4|CDH5|ANXA2|FYN|FN1|FAS|SFPQ|EPHA2|DPP4|ARHGEF15|DLL1|GSTO1|FLNA|PTPN1|MMRN2|SDC4|SPTAN1|DNM1|TLR4|CLIC4|ANXA1|TBC1D4|XRCC5|G6PD|PPP3CA|BCL2|DYRK1A|CDC42|RARG|TOP2B|PBK|SPTBN2|CFL1|ARF1|VIM|NR1H4|NUMB|CAMK1D</td><td>5.37E-12</td><td>GO.0048519</td></tr>
<tr><td>37</td><td>GO Process</td><td>response to cytokine</td><td>MMP2|CCL2|GAPDH|MT2A|LAMA5|THBS1|RORA|CNN2|ICAM1|CSF1R|KIT|CCR5|BCL2L1|ZYX|RORC|SNCA|PTGES|ANXA2|FYN|FN1|FAS|MYO1C|GSTO1|TLR4|ANXA1|SDC1|FSCN1|XRCC5|CAMK2B|BCL2|CAMK2A|CDC42|RARG|FLNB|CFL1|ARF1|VIM</td><td>1.05E-11</td><td>GO.0034097</td></tr>
<tr><td>34</td><td>GO Process</td><td>regulation of locomotion</td><td>MAP2K5|CCL2|LAMA4|HSPB1|LAMA5|PDGFRA|THBS1|AKT3|ICAM1|FERMT3|FLT1|APP|CSF1R|KIT|ITGA2|NOS3|DDB1|MMP14|DAB2|CALR|SNCA|FN1|PTRF|EPHA2|MYO1C|SLK|FLNA|MMRN2|SDC4|CLIC4|ANXA1|BCL2|NUMB|CAMK1D</td><td>1.52E-11</td><td>GO.0040012</td></tr>
</table>

<table>
<tr><td>133</td><td>GO Process</td><td>regulation of cellular process</td><td>ARHGEF5|MAP2K5|ITGB4|MYH9|CYP24A1|MMP2|EHD4|APLP1|CCL2|GAPDH|LAMA4|GNB4|PPP3R1|PLEK|ENO1|MT2A|CDC25B|HSPB1|BCL2L2|PXDN|LAMA5|PDGFRA|THBS1|RORA|DNAJB6|CNN2|PRKCG|AKT3|NGEF|TUBB4A|ARHGAP31|ADD1|ICAM1|FERMT3|TJP1|FLT1|APP|CSF1R|KIT|CCR5|CAPN2|ANXA5|ITGA2|NOS3|MELK|CCT2|PLK1|TUBA1A|DDB1|BCL2L1|CDC25A|ARF4|MMP14|BRSK1|DAB2|TENC1|NQO1|CALR|CDC25C|STXBP6|ZYX|RORC|JAG2|PRKCH|RARB|NR1I2|LCK|SNCA|CAV1|RGS12|TUBB4B|PTGES|USP1|MAP4K4|CDH5|ANXA2|ANXA6|FYN|FN1|FAS|ARHGEF26|PTRF|SFPQ|EPHA2|MYO1C|RXRG|MYH10|DPP4|ARHGEF15|TGM2|DLL1|ARHGAP30|COPA|GSTO1|SLK|FLNA|PTPN1|MMRN2|SDC4|SPTAN1|DNM1|TLR4|CLIC4|RXRB|ANXA1|TBC1D4|PIR|PSIP1|SDC1|FSCN1|XRCC5|G6PD|PPP3CA|CAMK2B|LTB4R|BCL2|CAMK2A|DYRK1A|SEPT7|CDC42|PDIA6|RARG|TOP2B|FLNB|PBK|SPTBN2|CFL1|ARF1|VIM|NR1H4|NUMB|PRKCSH|CAMK1D</td><td>2.12E-11</td><td>GO.0050794</td></tr>
<tr><td>35</td><td>GO Process</td><td>cellular response to cytokine stimulus</td><td>MMP2|CCL2|GAPDH|MT2A|LAMA5|THBS1|RORA|CNN2|ICAM1|CSF1R|KIT|CCR5|BCL2L1|ZYX|RORC|ANXA2|FYN|FN1|FAS|MYO1C|GSTO1|TLR4|ANXA1|SDC1|FSCN1|XRCC5|CAMK2B|BCL2|CAMK2A|CDC42|RARG|FLNB|CFL1|ARF1|VIM</td><td>2.37E-11</td><td>GO.0071345</td></tr>
<tr><td>56</td><td>GO Process</td><td>immune system process</td><td>ARHGEF5|MYH9|CCL2|GAPDH|PLEK|MT2A|PXDN|PDGFRA|THBS1|RORA|CNN2|ADD1|ICAM1|FLT1|APP|CSF1R|KIT|CCR5|MELK|CCT2|CALR|ZYX|RORC|JAG2|LCK|SNCA|CAV1|TUBB4B|ANXA2|FYN|FN1|FAS|SFPQ|EPHA2|MYO1C|DPP4|DLL1|SDC4|SPTAN1|TLR4|ANXA1|PIR|SDC1|XRCC5|G6PD|PPP3CA|CAMK2B|LTB4R|BCL2|CAMK2A|CDC42|FLNB|SPTBN2|ARF1|VIM|NR1H4</td><td>2.77E-11</td><td>GO.0002376</td></tr>
<tr><td>31</td><td>GO Process</td><td>regulation of cell migration</td><td>MAP2K5|CCL2|LAMA4|HSPB1|LAMA5|PDGFRA|THBS1|AKT3|ICAM1|FERMT3|FLT1|APP|CSF1R|KIT|ITGA2|NOS3|MMP14|DAB2|CALR|FN1|EPHA2|MYO1C|SLK|FLNA|MMRN2|SDC4|CLIC4|ANXA1|BCL2|NUMB|CAMK1D</td><td>3.86E-11</td><td>GO.0030334</td></tr>
<tr><td>32</td><td>GO Process</td><td>regulation of cell motility</td><td>MAP2K5|CCL2|LAMA4|HSPB1|LAMA5|PDGFRA|THBS1|AKT3|ICAM1|FERMT3|FLT1|APP|CSF1R|KIT|ITGA2|NOS3|MMP14|DAB2|CALR|FN1|PTRF|EPHA2|MYO1C|SLK|FLNA|MMRN2|SDC4|CLIC4|ANXA1|BCL2|NUMB|CAMK1D</td><td>3.92E-11</td><td>GO.2000145</td></tr>
<tr><td>100</td><td>GO Process</td><td>multicellular organismal process</td><td>MAP2K5|ITGB4|MYH9|CYP24A1|MMP2|APLP1|CCL2|PPP3R1|PLEK|CDC25B|BCL2L2|LAMA5|PDGFRA|THBS1|COL8A1|VWF|RORA|PRKCG|AKT3|NGEF|CAD|ADD1|ICAM1|FLT1|APP|CSF1R|KIT|NPC1L1|CAPN2|ANXA5|ITGA2|NOS3|MELK|CKB|PLK1|BCL2L1|ARF4|MMP14|BRSK1|DAB2|TENC1|CALR|CDC25C|RORC|JAG2|PRKCH|RARB|LCK|SNCA|CAV1|USP1|MAP4K4|CDH5|DOK4|TPM4|ANXA2|FYN|FN1|FAS|EPHA2|MYH10|DPP4|ARHGEF15|TGM2|DLL1|COPA|TPM3|FLNA|MMRN2|SDC4|SPTAN1|DNM1|TLR4|CLIC4|ANXA1|TPM2|PIR|SDC1|XRCC5|G6PD|PPP3CA|CAMK2B|LTB4R|BCL2|CAMK2A|DYRK1A|SEPT7|CDC42|SUN1|RARG|TOP2B|WEE1|FLNB|SPTBN2|CFL1|VIM|NR1H4|NUMB|APBA2|CAMK1D</td><td>4.51E-11</td><td>GO.0032501</td></tr>
<tr><td>34</td><td>GO Process</td><td>protein phosphorylation</td><td>MAP2K5|CCL2|CDC25B|PDGFRA|PRKCG|AKT3|CAD|FLT1|APP|CSF1R|KIT|CCR5|MELK|PLK1|BRSK1|PRKCH|LCK|MAP4K4|FYN|EPHA2|SLK|PTPN1|SPTAN1|TLR4|CAMK2B|BCL2|CAMK2A|DYRK1A|CDC42|WEE1|PBK|SPTBN2|CFL1|CAMK1D</td><td>4.57E-11</td><td>GO.0006468</td></tr>
<tr><td>43</td><td>GO Process</td><td>cell development</td><td>MYH9|PPP3R1|CDC25B|LAMA5|PDGFRA|ICAM1|FERMT3|TJP1|APP|CSF1R|KIT|BCL2L1|ARF4|BRSK1|FLNC|RARB|MAP4K4|DOK4|ANXA2|FYN|FN1|ARHGEF26|EPHA2|MYH10|DLL1|FLNA|SDC4|SPTAN1|CLIC4|SDC1|G6PD|PPP3CA|BCL2|CAMK2A|CDC42|RARG|TOP2B|WEE1|FLNB|SPTBN2|CFL1|VIM|NUMB</td><td>6.94E-11</td><td>GO.0048468</td></tr>
<tr><td>33</td><td>GO Process</td><td>regulation of cellular component movement</td><td>MAP2K5|CCL2|LAMA4|HSPB1|LAMA5|PDGFRA|THBS1|AKT3|ICAM1|FERMT3|FLT1|APP|CSF1R|KIT|ITGA2|NOS3|MMP14|DAB2|CALR|CAV1|FN1|PTRF|EPHA2|MYO1C|SLK|FLNA|MMRN2|SDC4|CLIC4|ANXA1|BCL2|NUMB|CAMK1D</td><td>7.65E-11</td><td>GO.0051270</td></tr>
<tr><td>82</td><td>GO Process</td><td>multicellular organism development</td><td>MAP2K5|ITGB4|MYH9|MMP2|APLP1|CCL2|PPP3R1|PLEK|BCL2L2|LAMA5|PDGFRA|THBS1|COL8A1|RORA|PRKCG|AKT3|NGEF|CAD|ADD1|ICAM1|FLT1|APP|CSF1R|KIT|CAPN2|ITGA2|NOS3|MELK|CKB|BCL2L1|ARF4|MMP14|BRSK1|DAB2|TENC1|CALR|RORC|JAG2|PRKCH|RARB|LCK|CAV1|USP1|MAP4K4|CDH5|DOK4|ANXA2|FYN|FN1|FAS|EPHA2|MYH10|ARHGEF15|TGM2|DLL1|FLNA|MMRN2|SDC4|SPTAN1|CLIC4|ANXA1|PIR|SDC1|XRCC5|G6PD|PPP3CA|CAMK2B|BCL2|CAMK2A|DYRK1A|CDC42|SUN1|RARG|TOP2B|WEE1|FLNB|SPTBN2|CFL1|VIM|NUMB|APBA2|CAMK1D</td><td>8.93E-11</td><td>GO.0007275</td></tr>
<tr><td>136</td><td>GO Process</td><td>regulation of biological process</td><td>ARHGEF5|MAP2K5|ITGB4|MYH9|CYP24A1|MMP2|EHD4|APLP1|CCL2|GAPDH|LAMA4|GNB4|PPP3R1|PLEK|ENO1|MT2A|CDC25B|HSPB1|BCL2L2|PXDN|LAMA5|PDGFRA|THBS1|COL8A1|RORA|DNAJB6|CNN2|PRKCG|AKT3|NGEF|TUBB4A|ARHGAP31|ADD1|ICAM1|FERMT3|TJP1|FLT1|APP|CSF1R|KIT|CCR5|CAPN2|ANXA5|ITGA2|NOS3|MELK|CCT2|PLK1|TUBA1A|DDB1|BCL2L1|CDC25A|ARF4|MMP14|BRSK1|DAB2|TENC1|NQO1|CALR|CDC25C|STXBP6|ZYX|RORC|JAG2|PRKCH|RARB|NR1I2|LCK|SNCA|CAV1|RGS12|TUBB4B|PTGES|USP1|MAP4K4|CDH5|ANXA2|ANXA6|FYN|FN1|FAS|ARHGEF26|PTRF|SFPQ|EPHA2|MYO1C|RXRG|MYH10|DPP4|ARHGEF15|TGM2|DLL1|ARHGAP30|COPA|GSTO1|SLK|FLNA|PTPN1|MMRN2|SDC4|SPTAN1|DNM1|TLR4|CLIC4|RXRB|ANXA1|TBC1D4|PIR|PSIP1|SDC1|FSCN1|XRCC5|G6PD|PPP3CA|CAMK2B|LTB4R|BCL2|CAMK2A|DYRK1A|SEPT7|CDC42|PDIA6|RARG|TOP2B|FLNB|PBK|SPTBN2|CFL1|ARF1|VIM|NR1H4|NUMB|APBA2|PRKCSH|ABCB1|CAMK1D</td><td>1.07E-10</td><td>GO.0050789</td></tr>
<tr><td>25</td><td>GO Process</td><td>actin filament-based process</td><td>ARHGEF5|MYH9|PLEK|PDGFRA|CNN2|ADD1|KIT|CALR|ZYX|TPM4|MYO1C|MYH10|TPM3|FLNA|CNN3|PTPN1|ANXA1|TPM2|FSCN1|MYO1B|BCL2|CDC42|FLNB|CFL1|VIM</td><td>1.10E-10</td><td>GO.0030029</td></tr>
<tr><td>23</td><td>GO Process</td><td>actin cytoskeleton organization</td><td>ARHGEF5|MYH9|PLEK|PDGFRA|CNN2|ADD1|KIT|CALR|ZYX|TPM4|MYH10|TPM3|FLNA|CNN3|PTPN1|ANXA1|TPM2|FSCN1|MYO1B|BCL2|CDC42|FLNB|CFL1</td><td>1.92E-10</td><td>GO.0030036</td></tr>
<tr><td>32</td><td>GO Process</td><td>regulation of cellular component biogenesis</td><td>ARHGEF5|PLEK|THBS1|DNAJB6|TUBB4A|ADD1|ICAM1|TJP1|KIT|PLK1|DDB1|MMP14|STXBP6|PRKCH|SNCA|CAV1|EPHA2|MYO1C|ARHGEF15|SLK|FLNA|SDC4|SPTAN1|TLR4|SDC1|FSCN1|XRCC5|DYRK1A|SEPT7|CDC42|SPTBN2|ARF1</td><td>2.05E-10</td><td>GO.0044087</td></tr>
<tr><td>61</td><td>GO Process</td><td>animal organ development</td><td>MAP2K5|ITGB4|MYH9|MMP2|APLP1|CCL2|PPP3R1|PLEK|BCL2L2|LAMA5|PDGFRA|COL8A1|RORA|AKT3|CAD|ADD1|ICAM1|APP|CSF1R|KIT|ITGA2|NOS3|MELK|CKB|BCL2L1|ARF4|MMP14|TENC1|CALR|RORC|JAG2|RARB|LCK|CAV1|ANXA2|FYN|FN1|EPHA2|MYH10|ARHGEF15|TGM2|DLL1|FLNA|SDC4|CLIC4|ANXA1|PIR|SDC1|XRCC5|G6PD|PPP3CA|BCL2|CDC42|SUN1|RARG|TOP2B|FLNB|SPTBN2|CFL1|VIM|NUMB</td><td>2.57E-10</td><td>GO.0048513</td></tr>
<tr><td>38</td><td>GO Process</td><td>phosphorylation</td><td>MAP2K5|CCL2|GAPDH|ENO1|CDC25B|PDGFRA|PRKCG|AKT3|CAD|FLT1|APP|CSF1R|KIT|CCR5|MELK|CKB|PLK1|BRSK1|PRKCH|LCK|SNCA|MAP4K4|FYN|EPHA2|SLK|PTPN1|SPTAN1|TLR4|CAMK2B|BCL2|CAMK2A|DYRK1A|CDC42|WEE1|PBK|SPTBN2|CFL1|CAMK1D</td><td>2.69E-10</td><td>GO.0016310</td></tr>
<tr><td>50</td><td>GO Process</td><td>phosphate-containing compound metabolic process</td><td>MAP2K5|CCL2|GAPDH|PPP3R1|PLEK|ENO1|CDC25B|ARF3|PDGFRA|RORA|PRKCG|AKT3|CAD|FLT1|APP|CSF1R|KIT|CCR5|MELK|CKB|PLK1|CDC25A|BRSK1|TENC1|CDC25C|PRKCH|LCK|SNCA|MAP4K4|FYN|EPHA2|SLK|PTPN1|SPTAN1|TLR4|G6PD|PPP3CA|CAMK2B|LDHB|BCL2|CAMK2A|DYRK1A|PAICS|CDC42|WEE1|PBK|SPTBN2|CFL1|ARF1|CAMK1D</td><td>2.72E-10</td><td>GO.0006796</td></tr>
<tr><td>45</td><td>GO Process</td><td>positive regulation of molecular function</td><td>ARHGEF5|MAP2K5|CCL2|PLEK|CDC25B|PDGFRA|THBS1|DNAJB6|ARHGAP31|ADD1|ICAM1|FLT1|APP|CSF1R|KIT|ITGA2|NOS3|CCT2|PLK1|ARF4|PRKCH|LCK|SNCA|CAV1|RGS12|MAP4K4|ANXA2|FYN|FN1|FAS|EPHA2|ARHGEF15|ARHGAP30|GSTO1|SLK|PTPN1|SDC4|TLR4|TBC1D4|XRCC5|PPP3CA|BCL2|CAMK2A|ABCB1|CAMK1D</td><td>3.19E-10</td><td>GO.0044093</td></tr>
<tr><td>36</td><td>GO Process</td><td>positive regulation of cellular component organization</td><td>ARHGEF5|CCL2|PLEK|ADD1|ICAM1|APP|KIT|ITGA2|CCT2|DDB1|DAB2|CALR|SNCA|CAV1|ANXA2|FYN|FN1|SFPQ|MYO1C|ARHGEF15|DLL1|FLNA|SDC4|DNM1|TLR4|ANXA1|SDC1|FSCN1|XRCC5|PPP3CA|CAMK2B|BCL2|SEPT7|CDC42|CFL1|CAMK1D</td><td>3.78E-10</td><td>GO.0051130</td></tr>
<tr><td>42</td><td>GO Process</td><td>intracellular signal transduction</td><td>ARHGEF5|MAP2K5|CCL2|PPP3R1|PLEK|HSPB1|BCL2L2|PDGFRA|PRKCG|AKT3|ARHGAP31|TJP1|APP|CSF1R|KIT|CCR5|NOS3|MELK|PLK1|BCL2L1|BRSK1|TENC1|CDC25C|PRKCH|MAP4K4|FYN|EPHA2|ARHGAP30|GSTO1|SLK|PTPN1|SPTAN1|TLR4|PPP3CA|CAMK2B|BCL2|CAMK2A|CDC42|SPTBN2|CFL1|PRKCSH|CAMK1D</td><td>4.74E-10</td><td>GO.0035556</td></tr>
</table>

65	GO Process	regulation of molecular function	ARHGEF5|MAP2K5|CCL2|GAPDH|PLEK|CDC25B|HSPB1|PXDN|PDGFRA|THBS1|DNAJB6|NGEF|ARHGAP31|ADD1|ICAM1|FLT1|APP|CSF1R|KIT|ANXA5|ITGA2|NOS3|CCT2|PLK1|CDC25A|ARF4|DAB2|NQO1|CDC25C|JAG2|PRKCH|LCK|SNCA|CAV1|RGS12|MAP4K4|ANXA2|FYN|FN1|FAS|ARHGEF26|EPHA2|ARHGEF15|ARHGAP30|COPA|GSTO1|SLK|FLNA|CNN3|PTPN1|SDC4|SPTAN1|TLR4|ANXA1|TBC1D4|TPM2|XRCC5|PPP3CA|CAMK2B|BCL2|CAMK2A|SPTBN2|NR1H4|ABCB1|CAMK1D	5.02E-10	GO.0065009
36	GO Process	locomotion	ARHGEF5|ITGB4|MYH9|CCL2|LAMA5|PDGFRA|THBS1|ICAM1|FLT1|APP|CSF1R|KIT|CCR5|ITGA2|NOS3|ARF4|MMP14|LCK|CAV1|DOK4|FYN|FN1|EPHA2|MYH10|DPP4|FLNA|MMRN2|SDC4|SPTAN1|ANXA1|SDC1|FSCN1|SUN1|TOP2B|SPTBN2|CFL1	5.26E-10	GO.0040011
36	GO Process	regulation of organelle organization	ARHGEF5|PLEK|PDGFRA|TUBB4A|ADD1|ICAM1|APP|CSF1R|CAPN2|CCT2|PLK1|BCL2L1|CALR|CDC25C|STXBP6|SNCA|ANXA2|SFPQ|MYO1C|ARHGEF15|FLNA|SDC4|SPTAN1|CLIC4|ANXA1|TBC1D4|SDC1|FSCN1|XRCC5|BCL2|DYRK1A|SEPT7|CDC42|SPTBN2|CFL1|ARF1	6.74E-10	GO.0033043
71	GO Process	regulation of response to stimulus	ARHGEF5|MAP2K5|MMP2|APLP1|CCL2|PPP3R1|PLEK|ENO1|HSPB1|BCL2L2|PXDN|PDGFRA|THBS1|RORA|DNAJB6|PRKCG|AKT3|NGEF|ARHGAP31|ICAM1|FLT1|APP|CSF1R|KIT|ITGA2|NOS3|BCL2L1|MMP14|DAB2|CALR|ZYX|JAG2|PRKCH|LCK|SNCA|CAV1|RGS12|USP1|MAP4K4|CDH5|ANXA2|FYN|FN1|FAS|ARHGEF26|SFPQ|EPHA2|MYO1C|ARHGEF15|TGM2|DLL1|ARHGAP30|COPA|GSTO1|FLNA|PTPN1|MMRN2|DNM1|TLR4|ANXA1|XRCC5|G6PD|PPP3CA|CAMK2B|BCL2|DYRK1A|CDC42|PBK|NR1H4|ABCB1|CAMK1D	6.74E-10	GO.0048583
85	GO Process	localization	ARHGEF5|ITGB4|MYH9|EHD4|APLP1|CCL2|PPP3R1|PLEK|HSPB1|LAMA5|ARF3|PDGFRA|THBS1|VWF|CNN2|TUBB4A|CAD|ADD1|ICAM1|EXOC6B|FERMT3|FLT1|APP|KIT|NPC1L1|CCR5|ANXA5|ITGA2|NOS3|CCT2|PLK1|TUBA1A|TUBA1C|BCL2L1|ARF4|MMP14|BRSK1|DAB2|CALR|STXBP6|NR1I2|LCK|SNCA|CAV1|TUBB4B|ANXA2|ANXA6|FYN|FN1|EPHA2|MYO1C|MYH10|DPP4|TGM2|DLL1|COPA|FLNA|MMRN2|SDC4|SPTAN1|DNM1|TLR4|CLIC4|ANXA1|TBC1D4|PSIP1|SDC1|FSCN1|XRCC5|MYO1B|PPP3CA|BCL2|CAMK2A|CDC42|SUN1|PDIA6|TOP2B|FLNB|SPTBN2|CFL1|ARF1|NR1H4|APBA2|TLN2|ABCB1	7.19E-10	GO.0051179
24	GO Process	positive regulation of locomotion	HSPB1|PDGFRA|THBS1|AKT3|ICAM1|FERMT3|FLT1|APP|CSF1R|KIT|ITGA2|NOS3|DDB1|MMP14|DAB2|CALR|FN1|PTRF|MYO1C|FLNA|ANXA1|BCL2|NUMB|CAMK1D	8.43E-10	GO.0040017
30	GO Process	cell migration	ARHGEF5|MYH9|CCL2|LAMA5|PDGFRA|THBS1|ICAM1|FLT1|KIT|CCR5|ITGA2|NOS3|ARF4|MMP14|LCK|CAV1|FYN|FN1|EPHA2|MYH10|DPP4|FLNA|MMRN2|SDC4|ANXA1|SDC1|FSCN1|SUN1|TOP2B|CFL1	8.51E-10	GO.0016477
27	GO Process	cytokine-mediated signaling pathway	MMP2|CCL2|MT2A|LAMA5|RORA|CNN2|ICAM1|CSF1R|KIT|CCR5|BCL2L1|RORC|ANXA2|FYN|FN1|FAS|GSTO1|ANXA1|SDC1|FSCN1|CAMK2B|BCL2|CAMK2A|CDC42|CFL1|ARF1|VIM	9.48E-10	GO.0019221
**19**	**GO Process**	**angiogenesis**	**MYH9|MMP2|CCL2|PPP3R1|LAMA5|PDGFRA|THBS1|COL8A1|RORA|FLT1|NOS3|MMP14|CAV1|ANXA2|FN1|EPHA2|DLL1|MMRN2|CLIC4**	**1.18E-09**	**GO.0001525**
23	GO Process	positive regulation of cell motility	HSPB1|PDGFRA|THBS1|AKT3|ICAM1|FERMT3|FLT1|APP|CSF1R|KIT|ITGA2|NOS3|MMP14|DAB2|CALR|FN1|PTRF|MYO1C|FLNA|ANXA1|BCL2|NUMB|CAMK1D	1.31E-09	GO.2000147
44	GO Process	regulation of transport	ARHGEF5|EHD4|CCL2|GAPDH|THBS1|PRKCG|ICAM1|APP|CSF1R|ITGA2|NOS3|BRSK1|DAB2|CALR|STXBP6|SNCA|CAV1|MAP4K4|ANXA2|FYN|FN1|MYO1C|MYH10|DPP4|DLL1|GSTO1|FLNA|PTPN1|SDC4|DNM1|TLR4|CLIC4|ANXA1|TBC1D4|SDC1|G6PD|PPP3CA|CAMK2B|BCL2|CAMK2A|ARF1|NR1H4|ABCB1|CAMK1D	1.41E-09	GO.0051049
53	GO Process	regulation of developmental process	MAP2K5|MYH9|CCL2|LAMA4|HSPB1|LAMA5|PDGFRA|THBS1|RORA|AKT3|NGEF|ADD1|ICAM1|FLT1|APP|CSF1R|KIT|NOS3|MMP14|BRSK1|DAB2|CALR|RORC|PRKCH|RARB|SNCA|CAV1|MAP4K4|CDH5|ANXA2|FYN|FN1|EPHA2|MYH10|ARHGEF15|DLL1|FLNA|MMRN2|TLR4|ANXA1|XRCC5|G6PD|PPP3CA|CAMK2B|BCL2|SEPT7|CDC42|RARG|CFL1|VIM|NR1H4|NUMB|CAMK1D	1.53E-09	GO.0050793
26	GO Process	regulation of cell adhesion	MAP2K5|CCL2|LAMA4|LAMA5|THBS1|COL8A1|ICAM1|FERMT3|ITGA2|MMP14|CALR|JAG2|LCK|CAV1|FYN|FN1|EPHA2|DPP4|TGM2|DLL1|SLK|FLNA|SDC4|ANXA1|BCL2|CDC42	1.70E-09	GO.0030155
64	GO Process	regulation of signaling	ARHGEF5|MAP2K5|APLP1|CCL2|PLEK|ENO1|HSPB1|BCL2L2|PXDN|PDGFRA|THBS1|RORA|AKT3|NGEF|ARHGAP31|ICAM1|FLT1|APP|CSF1R|KIT|ITGA2|NOS3|BCL2L1|MMP14|BRSK1|DAB2|CALR|JAG2|PRKCH|LCK|SNCA|CAV1|RGS12|MAP4K4|ANXA2|FYN|FN1|FAS|ARHGEF26|SFPQ|EPHA2|MYO1C|DPP4|ARHGEF15|TGM2|DLL1|ARHGAP30|COPA|GSTO1|FLNA|PTPN1|MMRN2|DNM1|TLR4|ANXA1|PPP3CA|CAMK2B|BCL2|CAMK2A|DYRK1A|CDC42|PBK|ARF1|NR1H4	2.11E-09	GO.0023051
29	GO Process	tube development	ITGB4|MYH9|MMP2|CCL2|PPP3R1|LAMA5|PDGFRA|THBS1|COL8A1|RORA|FLT1|CSF1R|KIT|NOS3|MMP14|RARB|CAV1|ANXA2|FN1|EPHA2|TGM2|DLL1|MMRN2|SDC4|CLIC4|SDC1|BCL2|RARG|CFL1	2.19E-09	GO.0035295
40	GO Process	negative regulation of response to stimulus	MAP2K5|APLP1|CCL2|PLEK|ENO1|HSPB1|BCL2L2|PXDN|PDGFRA|THBS1|RORA|AKT3|ICAM1|APP|NOS3|BCL2L1|MMP14|DAB2|CALR|SNCA|CAV1|RGS12|MAP4K4|CDH5|ANXA2|FYN|FAS|EPHA2|GSTO1|PTPN1|MMRN2|DNM1|TLR4|ANXA1|PPP3CA|BCL2|DYRK1A|CDC42|PBK|NR1H4	2.35E-09	GO.0048585
31	GO Process	cell motility	ARHGEF5|ITGB4|MYH9|CCL2|LAMA5|PDGFRA|THBS1|ICAM1|FLT1|KIT|CCR5|ITGA2|NOS3|ARF4|MMP14|LCK|CAV1|FYN|FN1|EPHA2|MYH10|DPP4|FLNA|MMRN2|SDC4|ANXA1|SDC1|FSCN1|SUN1|TOP2B|CFL1	2.56E-09	GO.0048870
22	GO Process	positive regulation of cell migration	HSPB1|PDGFRA|THBS1|AKT3|ICAM1|FERMT3|FLT1|APP|CSF1R|KIT|ITGA2|NOS3|MMP14|DAB2|CALR|FN1|MYO1C|FLNA|ANXA1|BCL2|NUMB|CAMK1D	3.72E-09	GO.0030335
63	GO Process	regulation of cell communication	ARHGEF5|MAP2K5|APLP1|CCL2|PLEK|ENO1|HSPB1|BCL2L2|PXDN|PDGFRA|THBS1|RORA|AKT3|NGEF|ARHGAP31|ICAM1|FLT1|APP|CSF1R|KIT|ITGA2|NOS3|BCL2L1|MMP14|BRSK1|DAB2|CALR|JAG2|PRKCH|LCK|SNCA|CAV1|RGS12|MAP4K4|FYN|FN1|FAS|ARHGEF26|SFPQ|EPHA2|MYO1C|DPP4|ARHGEF15|TGM2|DLL1|ARHGAP30|COPA|GSTO1|FLNA|PTPN1|MMRN2|DNM1|TLR4|ANXA1|PPP3CA|CAMK2B|BCL2|CAMK2A|DYRK1A|CDC42|PBK|ARF1|NR1H4	3.94E-09	GO.0010646
27	GO Process	cellular component morphogenesis	MYH9|LAMA5|PDGFRA|ADD1|FERMT3|APP|CSF1R|BCL2L1|BRSK1|MAP4K4|DOK4|FYN|FN1|ARHGEF26|EPHA2|MYH10|FLNA|SPTAN1|CLIC4|BCL2|CAMK2A|CDC42|TOP2B|WEE1|FLNB|SPTBN2|NUMB	6.12E-09	GO.0032989
25	GO Process	tube morphogenesis	MYH9|MMP2|CCL2|PPP3R1|LAMA5|PDGFRA|THBS1|COL8A1|RORA|FLT1|CSF1R|NOS3|MMP14|CAV1|ANXA2|FN1|EPHA2|TGM2|DLL1|MMRN2|SDC4|CLIC4|BCL2|RARG|CFL1	6.37E-09	GO.0035239
19	GO Process	regulation of actin filament-based process	ARHGEF5|MYH9|PLEK|PDGFRA|CNN2|ADD1|ICAM1|CSF1R|CAV1|MYO1C|ARHGEF15|FLNA|SDC4|SPTAN1|FSCN1|CDC42|SPTBN2|CFL1|ARF1	7.85E-09	GO.0032970
56	GO Process	regulation of multicellular organismal process	MAP2K5|GAPDH|LAMA4|PLEK|ENO1|HSPB1|LAMA5|PDGFRA|THBS1|RORA|AKT3|NGEF|ADD1|ICAM1|FLT1|APP|CSF1R|KIT|ANXA5|ITGA2|NOS3|MMP14|BRSK1|DAB2|CALR|PRKCH|RARB|SNCA|CAV1|MAP4K4|CDH5|ANXA2|ANXA6|FYN|FN1|EPHA2|ARHGEF15|DLL1|GSTO1|FLNA|MMRN2|TLR4|ANXA1|XRCC5|G6PD|PPP3CA|CAMK2B|BCL2|SEPT7|CDC42|RARG|CFL1|VIM|NR1H4|NUMB|CAMK1D	8.30E-09	GO.0051239
25	GO Process	cell morphogenesis	MYH9|LAMA5|ADD1|FERMT3|APP|CSF1R|BRSK1|MAP4K4|DOK4|FYN|FN1|ARHGEF26|EPHA2|MYH10|FLNA|SPTAN1|CLIC4|BCL2|CAMK2A|CDC42|TOP2B|WEE1|FLNB|SPTBN2|NUMB	8.78E-09	GO.0000902
22	GO Process	regulation of cytoskeleton organization	ARHGEF5|PLEK|PDGFRA|TUBB4A|ADD1|ICAM1|CSF1R|CAPN2|PLK1|SNCA|MYO1C|ARHGEF15|FLNA|SDC4|SPTAN1|CLIC4|FSCN1|DYRK1A|CDC42|SPTBN2|CFL1|ARF1	9.12E-09	GO.0051493
16	GO Process	regulation of endocytosis	EHD4|CCL2|PRKCG|APP|ITGA2|DAB2|CALR|SNCA|CAV1|ANXA2|DLL1|PTPN1|DNM1|PPP3CA|ARF1|CAMK1D	1.17E-08	GO.0030100

22	GO Process	vasculature development	MYH9|MMP2|CCL2|PPP3R1|LAMA5|PDGFRA|THBS1|COL8A1|RORA|FLT1|NOS3|MMP14|CAV1|CDH5|ANXA2|FN1|EPHA2|MYH10|ARHGEF15|DLL1|MMRN2|CLIC4	1.30E-08	GO.0001944
15	GO Process	cell junction organization	ITGB4|LAMA5|TJP1|CSF1R|ITGA2|FLNC|CDH5|FN1|FLNA|FSCN1|BCL2|CDC42|NR1H4|NUMB|TLN2	1.40E-08	GO.0034330
44	GO Process	regulation of multicellular organismal development	MAP2K5|LAMA4|HSPB1|LAMA5|THBS1|AKT3|NGEF|ADD1|FLT1|APP|KIT|NOS3|MMP14|BRSK1|DAB2|CALR|PRKCH|RARB|SNCA|CAV1|MAP4K4|CDH5|FYN|FN1|EPHA2|ARHGEF15|DLL1|FLNA|MMRN2|TLR4|ANXA1|XRCC5|G6PD|PPP3CA|CAMK2B|BCL2|SEPT7|CDC42|RARG|CFL1|VIM|NR1H4|NUMB|CAMK1D	1.40E-08	GO.2000026
37	GO Process	positive regulation of catalytic activity	ARHGEF5|MAP2K5|CCL2|PLEK|CDC25B|PDGFRA|THBS1|DNAJB6|ARHGAP31|ICAM1|FLT1|APP|CSF1R|KIT|ITGA2|NOS3|CCT2|PLK1|ARF4|LCK|SNCA|CAV1|RGS12|MAP4K4|FYN|FN1|FAS|EPHA2|ARHGEF15|ARHGAP30|SLK|PTPN1|SDC4|TLR4|TBC1D4|XRCC5|BCL2	1.51E-08	GO.0043085
13	GO Process	cell junction assembly	ITGB4|LAMA5|TJP1|ITGA2|FLNC|CDH5|FN1|FLNA|FSCN1|BCL2|CDC42|NR1H4|TLN2	1.53E-08	GO.0034329
29	GO Process	response to organic cyclic compound	CYP24A1|APLP1|CCL2|THBS1|RORA|PRKCG|CAD|ICAM1|APP|CCR5|ITGA2|BCL2L1|MMP14|NQO1|CALR|RORC|RARB|NR1I2|SNCA|CAV1|PTGES|RXRG|RXRB|ANXA1|SDC1|G6PD|BCL2|RARG|NR1H4	1.57E-08	GO.0014070
35	GO Process	negative regulation of cell communication	MAP2K5|APLP1|PLEK|ENO1|HSPB1|BCL2L2|PXDN|THBS1|RORA|ICAM1|APP|NOS3|BCL2L1|MMP14|DAB2|CALR|SNCA|CAV1|RGS12|MAP4K4|FYN|FAS|EPHA2|GSTO1|PTPN1|MMRN2|DNM1|TLR4|PPP3CA|BCL2|DYRK1A|CDC42|PBK|ARF1|NR1H4	1.73E-08	GO.0010648
22	GO Process	cell morphogenesis involved in differentiation	MYH9|LAMA5|FERMT3|APP|CSF1R|BRSK1|DOK4|FYN|FN1|ARHGEF26|EPHA2|MYH10|FLNA|SPTAN1|CLIC4|BCL2|CAMK2A|CDC42|TOP2B|FLNB|SPTBN2|NUMB	1.78E-08	GO.0000904
35	GO Process	negative regulation of signaling	MAP2K5|APLP1|PLEK|ENO1|HSPB1|BCL2L2|PXDN|THBS1|RORA|ICAM1|APP|NOS3|BCL2L1|MMP14|DAB2|CALR|SNCA|CAV1|RGS12|MAP4K4|FYN|FAS|EPHA2|GSTO1|PTPN1|MMRN2|DNM1|TLR4|PPP3CA|BCL2|DYRK1A|CDC42|PBK|ARF1|NR1H4	1.80E-08	GO.0023057
42	GO Process	regulation of intracellular signal transduction	ARHGEF5|MAP2K5|APLP1|CCL2|PLEK|ENO1|HSPB1|BCL2L2|PDGFRA|THBS1|RORA|AKT3|NGEF|ARHGAP31|ICAM1|FLT1|APP|CSF1R|KIT|BCL2L1|CALR|LCK|CAV1|MAP4K4|FYN|FN1|FAS|ARHGEF26|SFPQ|EPHA2|ARHGEF15|TGM2|ARHGAP30|GSTO1|PTPN1|TLR4|PPP3CA|BCL2|DYRK1A|CDC42|PBK|NR1H4	2.34E-08	GO.1902531
29	GO Process	positive regulation of transport	ARHGEF5|CCL2|GAPDH|APP|CSF1R|ITGA2|DAB2|CALR|SNCA|CAV1|ANXA2|FYN|MYO1C|MYH10|DLL1|GSTO1|FLNA|SDC4|DNM1|TLR4|ANXA1|SDC1|G6PD|PPP3CA|BCL2|CAMK2A|NR1H4|ABCB1|CAMK1D	2.40E-08	GO.0051050
41	GO Process	vesicle-mediated transport	MYH9|EHD4|APLP1|PLEK|ARF3|THBS1|VWF|CNN2|EXOC6B|FERMT3|APP|KIT|ANXA5|CCT2|BCL2L1|ARF4|DAB2|CALR|STXBP6|SNCA|CAV1|TUBB4B|ANXA2|FYN|FN1|MYO1C|MYH10|TGM2|DLL1|COPA|FLNA|SPTAN1|DNM1|ANXA1|TBC1D4|XRCC5|MYO1B|CDC42|PDIA6|SPTBN2|ARF1	2.64E-08	GO.0016192
21	GO Process	blood vessel development	MYH9|MMP2|CCL2|PPP3R1|LAMA5|PDGFRA|THBS1|COL8A1|RORA|FLT1|NOS3|MMP14|CAV1|CDH5|ANXA2|FN1|EPHA2|MYH10|DLL1|MMRN2|CLIC4	2.88E-08	GO.0001568
30	GO Process	regulation of anatomical structure morphogenesis	MAP2K5|MYH9|CCL2|HSPB1|THBS1|AKT3|NGEF|ICAM1|FLT1|CSF1R|KIT|NOS3|BRSK1|DAB2|CALR|CDH5|FYN|FN1|EPHA2|MYH10|DLL1|FLNA|MMRN2|ANXA1|PPP3CA|CAMK2B|BCL2|SEPT7|CDC42|CFL1	2.88E-08	GO.0022603
36	GO Process	response to endogenous stimulus	MMP2|APLP1|CCL2|PDGFRA|THBS1|RORA|CAD|ICAM1|APP|KIT|CAPN2|ITGA2|BCL2L1|MMP14|NQO1|CALR|ZYX|RORC|RARB|NR1I2|SNCA|CAV1|CDH5|ANXA2|FYN|RXRG|PTPN1|TLR4|RXRB|ANXA1|TBC1D4|SDC1|BCL2|CAMK2A|RARG|NR1H4	2.95E-08	GO.0009719
37	GO Process	response to oxygen-containing compound	CYP24A1|MMP2|APLP1|CCL2|PDGFRA|THBS1|RORA|CAD|ICAM1|APP|CCR5|CAPN2|ITGA2|NOS3|BCL2L1|NQO1|CALR|RORC|SNCA|CAV1|PTGES|FYN|FAS|PTPN1|DNM1|TLR4|ANXA1|TBC1D4|SDC1|XRCC5|G6PD|PPP3CA|BCL2|CAMK2A|RARG|CFL1|NR1H4	3.22E-08	GO.1901700
48	GO Process	regulation of catalytic activity	ARHGEF5|MAP2K5|CCL2|GAPDH|PLEK|CDC25B|HSPB1|PDGFRA|THBS1|DNAJB6|NGEF|ARHGAP31|ICAM1|FLT1|APP|CSF1R|KIT|ANXA5|ITGA2|NOS3|CCT2|PLK1|CDC25A|ARF4|NQO1|CDC25C|LCK|SNCA|CAV1|RGS12|MAP4K4|ANXA2|FYN|FN1|FAS|EPHA2|ARHGEF15|ARHGAP30|SLK|CNN3|PTPN1|SDC4|TLR4|ANXA1|TBC1D4|TPM2|XRCC5|BCL2	3.26E-08	GO.0050790
35	GO Process	regulation of response to stress	ARHGEF5|MMP2|PLEK|ENO1|HSPB1|PDGFRA|THBS1|RORA|DNAJB6|PRKCG|AKT3|APP|ITGA2|NOS3|BCL2L1|ZYX|SNCA|CAV1|USP1|MAP4K4|CDH5|ANXA2|FYN|FAS|SFPQ|TGM2|PTPN1|TLR4|ANXA1|XRCC5|PPP3CA|DYRK1A|PBK|NR1H4|ABCB1	3.77E-08	GO.0080134
19	GO Process	supramolecular fiber organization	PDGFRA|DNAJB6|ADD1|APP|ZYX|SNCA|TPM4|ANXA2|MYH10|TPM3|SLK|FLNA|TPM2|FSCN1|MYO1B|BCL2|CDC42|CFL1|VIM	4.42E-08	GO.0097435
38	GO Process	neurogenesis	CCL2|PPP3R1|RORA|NGEF|APP|CSF1R|KIT|ARF4|MMP14|BRSK1|CALR|JAG2|PRKCH|RARB|MAP4K4|DOK4|FYN|FN1|MYH10|DLL1|FLNA|SDC4|SPTAN1|ANXA1|XRCC5|PPP3CA|CAMK2B|BCL2|CAMK2A|CDC42|SUN1|TOP2B|WEE1|SPTBN2|CFL1|VIM|NUMB|CAMK1D	4.58E-08	GO.0022008
21	GO Process	regulation of vesicle-mediated transport	EHD4|CCL2|PRKCG|APP|ITGA2|DAB2|CALR|STXBP6|SNCA|CAV1|ANXA2|DLL1|PTPN1|SDC4|DNM1|ANXA1|TBC1D4|SDC1|PPP3CA|ARF1|CAMK1D	4.75E-08	GO.0060627
31	GO Process	response to abiotic stimulus	PLOD1|MMP2|THBS1|RORA|CNN2|ICAM1|PLOD2|APP|KIT|CAPN2|ITGA2|NOS3|DDB1|BCL2L1|CDC25A|MMP14|BRSK1|NQO1|CYP2R1|CAV1|USP1|FYN|FAS|DPP4|TLR4|ANXA1|PSIP1|XRCC5|BCL2|SUN1|PBK	5.03E-08	GO.0009628
31	GO Process	positive regulation of phosphate metabolic process	ARHGEF5|MAP2K5|EHD4|CCL2|PLEK|ENO1|CDC25B|PDGFRA|THBS1|ICAM1|FLT1|APP|CSF1R|KIT|ITGA2|NOS3|PLK1|DAB2|SNCA|CAV1|MAP4K4|ANXA2|FYN|FAS|SLK|PTPN1|SDC4|TLR4|XRCC5|BCL2|NR1H4	5.03E-08	GO.0045937
24	GO Process	symbiont process	CCL2|GAPDH|PDGFRA|ICAM1|CCR5|ITGA2|DDB1|BCL2L1|CDC25C|ZYX|LCK|CAV1|ANXA2|FYN|FN1|EPHA2|DPP4|PSIP1|XRCC5|DYRK1A|CDC42|CFL1|ARF1|VIM	7.09E-08	GO.0044403
27	GO Process	response to lipid	CYP24A1|CCL2|THBS1|RORA|CAD|ICAM1|CCR5|ITGA2|NOS3|NQO1|CALR|RORC|RARB|NR1I2|SNCA|CAV1|PTGES|FAS|RXRG|TLR4|RXRB|ANXA1|SDC1|XRCC5|BCL2|RARG|NR1H4	7.70E-08	GO.0033993
39	GO Process	tissue development	PLOD1|ITGB4|MMP2|PPP3R1|LAMA5|PDGFRA|COL8A1|ICAM1|TJP1|CSF1R|ITGA2|NOS3|MMP14|BRSK1|DAB2|CALR|RORC|JAG2|RARB|CAV1|FN1|ARHGEF26|EPHA2|MYH10|TGM2|DLL1|FLNA|CNN3|SDC4|CLIC4|ANXA1|SDC1|PPP3CA|BCL2|CDC42|RARG|FLNB|CFL1|VIM	7.94E-08	GO.0009888
27	GO Process	anatomical structure formation involved in morphogenesis	ITGB4|MYH9|MMP2|CCL2|PPP3R1|LAMA5|PDGFRA|THBS1|COL8A1|RORA|FLT1|CAPN2|ITGA2|NOS3|MMP14|CAV1|ANXA2|FN1|EPHA2|MYH10|TGM2|DLL1|MMRN2|SDC4|CLIC4|RARG|CFL1	8.80E-08	GO.0048646
24	GO Process	positive regulation of cell death	ARHGEF5|CCL2|GAPDH|THBS1|NGEF|APP|MELK|BCL2L1|NQO1|RARB|LCK|SNCA|CAV1|FYN|FAS|ARHGEF26|SFPQ|TGM2|TLR4|ANXA1|BCL2|CAMK2A|RARG|CAMK1D	9.98E-08	GO.0010942
17	GO Process	regulation of supramolecular fiber organization	ARHGEF5|PLEK|TUBB4A|ADD1|ICAM1|APP|SNCA|MYO1C|ARHGEF15|FLNA|SDC4|SPTAN1|DYRK1A|CDC42|SPTBN2|CFL1|ARF1	1.01E-07	GO.1902903
32	GO Process	negative regulation of signal transduction	MAP2K5|APLP1|PLEK|ENO1|HSPB1|BCL2L2|PXDN|THBS1|RORA|ICAM1|APP|NOS3|BCL2L1|MMP14|DAB2|CALR|SNCA|CAV1|RGS12|FYN|FAS|EPHA2|GSTO1|PTPN1|MMRN2|DNM1|TLR4|BCL2|DYRK1A|CDC42|PBK|NR1H4	1.16E-07	GO.0009968
30	GO Process	plasma membrane bounded cell projection organization	MYH9|PLEK|LAMA5|TUBB4A|APP|CSF1R|KIT|PLK1|TUBA1A|ARF4|BRSK1|TUBB4B|MAP4K4|DOK4|FYN|ARHGEF26|MYH10|FLNA|SDC4|SPTAN1|FSCN1|BCL2|CAMK2A|SEPT7|CDC42|TOP2B|WEE1|SPTBN2|ARF1|NUMB	1.27E-07	GO.0120036
56	GO Process	regulation of signal transduction	ARHGEF5|MAP2K5|APLP1|CCL2|PLEK|ENO1|HSPB1|BCL2L2|PXDN|PDGFRA|THBS1|RORA|AKT3|NGEF|ARHGAP31|ICAM1|FLT1|APP|CSF1R|KIT|NOS3|BCL2L1|MMP14|DAB2|CALR|JAG2|PRKCH|LCK|SNCA|CAV1|RGS12|MAP4K4|FYN|FN1|FAS|ARHGEF26|SFPQ|EPHA2|MYO1C|ARHGEF15|TGM2|DLL1|ARHGAP30|COPA|GSTO1|FLNA|PTPN1|MMRN2|DNM1|TLR4|PPP3CA|BCL2|DYRK1A|CDC42|PBK|NR1H4	1.31E-07	GO.0009966

<table>
<tr><td>46</td><td>GO Process</td><td>multi-organism process</td><td>MMP2|CCL2|GAPDH|ENO1|CDC25B|HSPB1|BCL2L2|PDGFRA|CAD|ICAM1|APP|KIT|CCR5|ITGA2|NOS3|CCT2|PLK1|DDB1|BCL2L1|CALR|CDC25C|ZYX|JAG2|LCK|SNCA|CAV1|PTGES|ANXA2|FYN|FN1|FAS|EPHA2|DPP4|TLR4|CLIC4|PSIP1|XRCC5|BCL2|DYRK1A|SEPT7|CDC42|SUN1|CFL1|ARF1|VIM|NR1H4</td><td>1.85E-07</td><td>GO.0051704</td></tr>
<tr><td>23</td><td>GO Process</td><td>negative regulation of cellular component organization</td><td>THBS1|DNAJB6|NGEF|TUBB4A|ADD1|APP|PLK1|BCL2L1|MMP14|SNCA|CAV1|MAP4K4|FYN|DPP4|ARHGEF15|SPTAN1|TBC1D4|XRCC5|PPP3CA|DYRK1A|CDC42|SPTBN2|VIM</td><td>1.91E-07</td><td>GO.0051129</td></tr>
<tr><td>21</td><td>GO Process</td><td>hemopoiesis</td><td>MYH9|PLEK|PDGFRA|RORA|ADD1|APP|CSF1R|KIT|MELK|RORC|JAG2|LCK|ANXA2|EPHA2|DLL1|ANXA1|PIR|XRCC5|G6PD|BCL2|CDC42</td><td>1.98E-07</td><td>GO.0030097</td></tr>
<tr><td>26</td><td>GO Process</td><td>circulatory system development</td><td>MAP2K5|MYH9|MMP2|CCL2|PPP3R1|LAMA5|PDGFRA|THBS1|COL8A1|RORA|CAD|FLT1|NOS3|MMP14|CALR|RARB|CAV1|CDH5|ANXA2|FN1|EPHA2|MYH10|ARHGEF15|DLL1|MMRN2|CLIC4</td><td>1.99E-07</td><td>GO.0072359</td></tr>
<tr><td>17</td><td>GO Process</td><td>response to metal ion</td><td>MT2A|THBS1|ADD1|ICAM1|APP|KIT|NQO1|CALR|CYP2R1|SNCA|CAV1|PTGES|CLIC4|SDC1|G6PD|PPP3CA|BCL2</td><td>2.54E-07</td><td>GO.0010038</td></tr>
<tr><td>16</td><td>GO Process</td><td>regulation of actin cytoskeleton organization</td><td>ARHGEF5|PLEK|PDGFRA|ADD1|ICAM1|CSF1R|MYO1C|ARHGEF15|FLNA|SDC4|SPTAN1|FSCN1|CDC42|SPTBN2|CFL1|ARF1</td><td>2.74E-07</td><td>GO.0032956</td></tr>
<tr><td>58</td><td>GO Process</td><td>positive regulation of metabolic process</td><td>ARHGEF5|MAP2K5|MYH9|EHD4|CCL2|PPP3R1|PLEK|ENO1|CDC25B|HSPB1|PDGFRA|THBS1|RORA|PRKCG|ICAM1|FLT1|APP|CSF1R|KIT|ITGA2|NOS3|CCT2|PLK1|DDB1|ARF4|MMP14|DAB2|CALR|RORC|RARB|NR1I2|LCK|SNCA|CAV1|MAP4K4|ANXA2|FYN|FN1|FAS|SFPQ|MYO1C|RXRG|DLL1|SLK|PTPN1|SDC4|TLR4|RXRB|ANXA1|PSIP1|XRCC5|PPP3CA|BCL2|DYRK1A|RARG|VIM|NR1H4|CAMK1D</td><td>2.78E-07</td><td>GO.0009893</td></tr>
<tr><td>20</td><td>GO Process</td><td>response to inorganic substance</td><td>MT2A|THBS1|ADD1|ICAM1|APP|KIT|NOS3|NQO1|CALR|CYP2R1|SNCA|CAV1|PTGES|FYN|CLIC4|ANXA1|SDC1|G6PD|PPP3CA|BCL2</td><td>3.25E-07</td><td>GO.0010035</td></tr>
<tr><td>27</td><td>GO Process</td><td>cellular response to oxygen-containing compound</td><td>CYP24A1|MMP2|APLP1|CCL2|PDGFRA|RORA|ICAM1|APP|CCR5|CAPN2|ITGA2|NOS3|BCL2L1|NQO1|RORC|SNCA|CAV1|FYN|PTPN1|TLR4|ANXA1|TBC1D4|XRCC5|PPP3CA|CAMK2A|RARG|NR1H4</td><td>3.67E-07</td><td>GO.1901701</td></tr>
<tr><td>56</td><td>GO Process</td><td>organelle organization</td><td>ARHGEF5|MYH9|CCL2|GAPDH|PLEK|CDC25B|LAMA5|PDGFRA|DNAJB6|CNN2|AKT3|TUBB4A|ADD1|KIT|NOS3|PLK1|TUBA1A|TUBA1C|DDB1|BCL2L1|BRSK1|CALR|ZYX|SNCA|CAV1|TUBB4B|PTMA|TPM4|ANXA2|SFPQ|MYH10|TPM3|SLK|FLNA|CNN3|PTPN1|SPTAN1|DNM1|ANXA1|TPM2|FSCN1|XRCC5|MYO1B|BCL2|CAMK2A|SEPT7|CDC42|SUN1|TOP2B|WEE1|FLNB|SPTBN2|CFL1|VIM|NR1H4|TLN2</td><td>3.78E-07</td><td>GO.0006996</td></tr>
<tr><td>22</td><td>GO Process</td><td>positive regulation of apoptotic process</td><td>ARHGEF5|CCL2|GAPDH|THBS1|NGEF|APP|MELK|BCL2L1|NQO1|RARB|LCK|SNCA|CAV1|FAS|ARHGEF26|SFPQ|TGM2|ANXA1|BCL2|CAMK2A|RARG|CAMK1D</td><td>3.87E-07</td><td>GO.0043065</td></tr>
<tr><td>45</td><td>GO Process</td><td>nervous system development</td><td>APLP1|CCL2|PPP3R1|RORA|PRKCG|AKT3|NGEF|APP|CSF1R|KIT|CKB|ARF4|MMP14|BRSK1|CALR|JAG2|PRKCH|RARB|MAP4K4|DOK4|FYN|FN1|MYH10|DLL1|FLNA|SDC4|SPTAN1|ANXA1|XRCC5|PPP3CA|CAMK2B|BCL2|CAMK2A|DYRK1A|CDC42|SUN1|RARG|TOP2B|WEE1|SPTBN2|CFL1|VIM|NUMB|APBA2|CAMK1D</td><td>4.10E-07</td><td>GO.0007399</td></tr>
<tr><td>32</td><td>GO Process</td><td>defense response</td><td>CCL2|GAPDH|MT2A|THBS1|ICAM1|APP|CSF1R|KIT|CCR5|ZYX|LCK|SNCA|TUBB4B|PTGES|FYN|FN1|FAS|SFPQ|MYO1C|DPP4|TLR4|ANXA1|SDC1|XRCC5|CAMK2B|LTB4R|BCL2|CAMK2A|FLNB|VIM|NR1H4|CAMK1D</td><td>4.34E-07</td><td>GO.0006952</td></tr>
<tr><td>37</td><td>GO Process</td><td>regulation of cell population proliferation</td><td>MAP2K5|MMP2|CCL2|CDC25B|LAMA5|PDGFRA|THBS1|AKT3|FLT1|APP|CSF1R|KIT|ITGA2|NOS3|BCL2L1|TENC1|CALR|JAG2|PRKCH|RARB|LCK|CAV1|PTGES|CDH5|ANXA2|FYN|FN1|FAS|DPP4|TGM2|DLL1|FLNA|SDC4|ANXA1|XRCC5|BCL2|RARG</td><td>4.46E-07</td><td>GO.0042127</td></tr>
<tr><td>30</td><td>GO Process</td><td>cellular response to endogenous stimulus</td><td>MMP2|APLP1|CCL2|PDGFRA|RORA|CAD|ICAM1|APP|KIT|CAPN2|ITGA2|BCL2L1|CALR|ZYX|RORC|RARB|NR1I2|SNCA|CAV1|CDH5|FYN|RXRG|PTPN1|TLR4|RXRB|ANXA1|TBC1D4|CAMK2A|RARG|NR1H4</td><td>4.87E-07</td><td>GO.0071495</td></tr>
<tr><td>55</td><td>GO Process</td><td>positive regulation of macromolecule metabolic process</td><td>ARHGEF5|MAP2K5|MYH9|EHD4|CCL2|PPP3R1|ENO1|CDC25B|HSPB1|PDGFRA|THBS1|RORA|PRKCG|ICAM1|FLT1|APP|CSF1R|KIT|ITGA2|NOS3|CCT2|PLK1|DDB1|ARF4|MMP14|DAB2|CALR|RORC|RARB|NR1I2|LCK|SNCA|CAV1|MAP4K4|ANXA2|FYN|FN1|FAS|SFPQ|MYO1C|RXRG|DLL1|SLK|PTPN1|SDC4|TLR4|RXRB|PSIP1|XRCC5|PPP3CA|BCL2|DYRK1A|RARG|VIM|NR1H4</td><td>5.53E-07</td><td>GO.0010604</td></tr>
<tr><td>54</td><td>GO Process</td><td>cellular protein modification process</td><td>RCN1|MAP2K5|PLOD1|CCL2|GAPDH|PPP3R1|CDC25B|PDGFRA|THBS1|PRKCG|AKT3|CAD|FLT1|PLOD2|APP|CSF1R|KIT|CCR5|MELK|PLK1|DDB1|CDC25A|ARF4|BRSK1|TENC1|CDC25C|PRKCH|LCK|USP1|MAP4K4|FYN|FN1|SFPQ|EPHA2|TGM2|SLK|PTPN1|SPTAN1|TLR4|ANXA1|PPP3CA|CAMK2B|BCL2|CAMK2A|DYRK1A|CDC42|PDIA6|WEE1|PBK|SPTBN2|CFL1|NR1H4|PRKCSH|CAMK1D</td><td>5.89E-07</td><td>GO.0006464</td></tr>
<tr><td>36</td><td>GO Process</td><td>positive regulation of multicellular organismal process</td><td>GAPDH|PLEK|ENO1|HSPB1|THBS1|RORA|AKT3|ICAM1|FLT1|APP|CSF1R|KIT|ITGA2|NOS3|MMP14|DAB2|CALR|PRKCH|RARB|CAV1|CDH5|ANXA2|FYN|FN1|DLL1|GSTO1|FLNA|TLR4|ANXA1|XRCC5|PPP3CA|CAMK2B|BCL2|NR1H4|NUMB|CAMK1D</td><td>7.09E-07</td><td>GO.0051240</td></tr>
<tr><td>13</td><td>GO Process</td><td>protein autophosphorylation</td><td>PDGFRA|PRKCG|CAD|FLT1|CSF1R|KIT|MELK|LCK|FYN|SLK|CAMK2B|CAMK2A|DYRK1A</td><td>7.28E-07</td><td>GO.0046777</td></tr>
<tr><td>11</td><td>GO Process</td><td>positive regulation of endocytosis</td><td>CCL2|APP|ITGA2|DAB2|CALR|SNCA|ANXA2|DLL1|DNM1|PPP3CA|CAMK1D</td><td>7.42E-07</td><td>GO.0045807</td></tr>
<tr><td>16</td><td>GO Process</td><td>response to steroid hormone</td><td>THBS1|RORA|CAD|ICAM1|CALR|RORC|RARB|NR1I2|CAV1|RXRG|RXRB|ANXA1|SDC1|BCL2|RARG|NR1H4</td><td>7.63E-07</td><td>GO.0048545</td></tr>
<tr><td>53</td><td>GO Process</td><td>positive regulation of nitrogen compound metabolic process</td><td>ARHGEF5|MAP2K5|MYH9|EHD4|CCL2|PPP3R1|ENO1|CDC25B|HSPB1|PDGFRA|THBS1|RORA|PRKCG|ICAM1|FLT1|APP|CSF1R|KIT|ITGA2|NOS3|CCT2|PLK1|DDB1|ARF4|MMP14|DAB2|RORC|RARB|NR1I2|LCK|SNCA|CAV1|MAP4K4|ANXA2|FYN|FN1|FAS|SFPQ|RXRG|DLL1|SLK|PTPN1|SDC4|TLR4|RXRB|PSIP1|XRCC5|PPP3CA|BCL2|DYRK1A|RARG|VIM|NR1H4</td><td>8.35E-07</td><td>GO.0051173</td></tr>
<tr><td>37</td><td>GO Process</td><td>regulation of phosphate metabolic process</td><td>ARHGEF5|MAP2K5|EHD4|CCL2|PLEK|ENO1|CDC25B|HSPB1|PDGFRA|THBS1|ICAM1|FLT1|APP|CSF1R|KIT|ITGA2|NOS3|PLK1|CDC25A|DAB2|CDC25C|SNCA|CAV1|MAP4K4|ANXA2|FYN|FN1|FAS|EPHA2|SLK|PTPN1|SDC4|TLR4|XRCC5|BCL2|PBK|NR1H4</td><td>1.11E-06</td><td>GO.0019220</td></tr>
<tr><td>54</td><td>GO Process</td><td>positive regulation of cellular metabolic process</td><td>ARHGEF5|MAP2K5|MYH9|EHD4|CCL2|PPP3R1|PLEK|ENO1|CDC25B|HSPB1|PDGFRA|THBS1|RORA|PRKCG|ICAM1|FLT1|APP|CSF1R|KIT|ITGA2|NOS3|CCT2|PLK1|ARF4|MMP14|DAB2|RORC|RARB|NR1I2|LCK|SNCA|CAV1|MAP4K4|ANXA2|FYN|FN1|FAS|SFPQ|RXRG|DLL1|SLK|PTPN1|SDC4|TLR4|RXRB|ANXA1|PSIP1|XRCC5|PPP3CA|BCL2|DYRK1A|RARG|VIM|NR1H4</td><td>1.11E-06</td><td>GO.0031325</td></tr>
<tr><td>36</td><td>GO Process</td><td>positive regulation of protein metabolic process</td><td>ARHGEF5|MAP2K5|MYH9|EHD4|CCL2|ENO1|CDC25B|HSPB1|PDGFRA|THBS1|ICAM1|FLT1|APP|CSF1R|KIT|ITGA2|PLK1|DDB1|MMP14|DAB2|LCK|SNCA|CAV1|MAP4K4|ANXA2|FYN|FN1|FAS|SLK|PTPN1|SDC4|TLR4|XRCC5|BCL2|DYRK1A|VIM</td><td>1.18E-06</td><td>GO.0051247</td></tr>
<tr><td>42</td><td>GO Process</td><td>positive regulation of response to stimulus</td><td>ARHGEF5|MAP2K5|MMP2|CCL2|PLEK|HSPB1|PDGFRA|THBS1|PRKCG|AKT3|ICAM1|FLT1|APP|CSF1R|KIT|ITGA2|BCL2L1|DAB2|CALR|JAG2|PRKCH|LCK|SNCA|CAV1|MAP4K4|FYN|FAS|SFPQ|MYO1C|TGM2|DLL1|GSTO1|FLNA|PTPN1|TLR4|ANXA1|XRCC5|PPP3CA|BCL2|CDC42|NR1H4|CAMK1D</td><td>1.21E-06</td><td>GO.0048584</td></tr>
<tr><td>31</td><td>GO Process</td><td>regulation of hydrolase activity</td><td>ARHGEF5|MAP2K5|CCL2|GAPDH|PLEK|PDGFRA|THBS1|DNAJB6|NGEF|ARHGAP31|ICAM1|FLT1|APP|KIT|ITGA2|NOS3|ARF4|LCK|SNCA|CAV1|RGS12|FYN|FN1|FAS|EPHA2|ARHGEF15|ARHGAP30|CNN3|ANXA1|TBC1D4|TPM2</td><td>1.45E-06</td><td>GO.0051336</td></tr>
<tr><td>15</td><td>GO Process</td><td>extracellular matrix organization</td><td>ITGB4|MMP2|LAMA4|PXDN|LAMA5|PDGFRA|THBS1|COL8A1|VWF|ICAM1|APP|ITGA2|MMP14|ANXA2|FN1</td><td>1.46E-06</td><td>GO.0030198</td></tr>
<tr><td>25</td><td>GO Process</td><td>peptidyl-amino acid modification</td><td>MAP2K5|PLOD1|GAPDH|PDGFRA|PRKCG|AKT3|CAD|FLT1|PLOD2|CSF1R|KIT|MELK|PLK1|PRKCH|LCK|FYN|EPHA2|TGM2|CAMK2B|BCL2|CAMK2A|DYRK1A|WEE1|NR1H4|CAMK1D</td><td>1.52E-06</td><td>GO.0018193</td></tr>
<tr><td>18</td><td>GO Process</td><td>regulation of cell morphogenesis</td><td>MYH9|CCL2|NGEF|ICAM1|CSF1R|KIT|BRSK1|CALR|FYN|FN1|MYH10|FLNA|ANXA1|PPP3CA|CAMK2B|SEPT7|CDC42|CFL1</td><td>1.55E-06</td><td>GO.0022604</td></tr>
<tr><td>12</td><td>GO Process</td><td>response to interferon-gamma</td><td>CCL2|GAPDH|MT2A|ICAM1|ZYX|SNCA|MYO1C|TLR4|CAMK2B|CAMK2A|FLNB|VIM</td><td>1.58E-06</td><td>GO.0034341</td></tr>
<tr><td>12</td><td>GO Process</td><td>epithelial cell development</td><td>ICAM1|TJP1|RARB|ARHGEF26|EPHA2|DLL1|FLNA|CLIC4|SDC1|RARG|FLNB|VIM</td><td>1.87E-06</td><td>GO.0002064</td></tr>
</table>

<table>
<tr><td></td><td></td><td></td><td></td><td></td><td></td></tr>
<tr><td>25</td><td>GO Process</td><td>response to hormone</td><td>THBS1|RORA|CAD|ICAM1|KIT|ITGA2|MMP14|NQO1|CALR|RORC|RARB|NR1I2|CAV1|ANXA2|FYN|RXRG|PTPN1|RXRB|ANXA1|TBC1D4|SDC1|BCL2|CAMK2A|RARG|NR1H4</td><td>1.92E-06</td><td>GO.0009725</td></tr>
<tr><td>39</td><td>GO Process</td><td>response to external stimulus</td><td>ARHGEF5|CYP24A1|CCL2|GAPDH|ENO1|HSPB1|PDGFRA|THBS1|CNN2|CAD|ICAM1|FLT1|APP|CSF1R|KIT|CCR5|ITGA2|NOS3|BCL2L1|MMP14|NQO1|SNCA|CAV1|PTGES|DOK4|FYN|FAS|EPHA2|MYH10|SPTAN1|TLR4|ANXA1|G6PD|BCL2|SUN1|SPTBN2|CFL1|ARF1|NR1H4</td><td>2.01E-06</td><td>GO.0009605</td></tr>
<tr><td>22</td><td>GO Process</td><td>cell population proliferation</td><td>BCL2L2|LAMA5|COL8A1|RORA|CSF1R|KIT|ITGA2|MELK|PLK1|BCL2L1|CDC25A|MMP14|DAB2|CDC25C|FYN|EPHA2|MYH10|FSCN1|XRCC5|BCL2|NUMB|ABCB1</td><td>2.17E-06</td><td>GO.0008283</td></tr>
<tr><td>35</td><td>GO Process</td><td>immune response</td><td>CCL2|GAPDH|MT2A|PXDN|THBS1|RORA|CNN2|ICAM1|APP|CSF1R|KIT|CCR5|CCT2|ZYX|RORC|LCK|SNCA|TUBB4B|ANXA2|FYN|FAS|SFPQ|MYO1C|DLL1|SPTAN1|TLR4|ANXA1|XRCC5|CAMK2B|LTB4R|BCL2|CAMK2A|FLNB|VIM|NR1H4</td><td>2.30E-06</td><td>GO.0006955</td></tr>
<tr><td>23</td><td>GO Process</td><td>positive regulation of hydrolase activity</td><td>ARHGEF5|CCL2|PLEK|PDGFRA|DNAJB6|ARHGAP31|ICAM1|FLT1|APP|KIT|ITGA2|ARF4|LCK|SNCA|CAV1|RGS12|FYN|FN1|FAS|EPHA2|ARHGEF15|ARHGAP30|TBC1D4</td><td>2.47E-06</td><td>GO.0051345</td></tr>
<tr><td>34</td><td>GO Process</td><td>positive regulation of cellular protein metabolic process</td><td>ARHGEF5|MAP2K5|MYH9|EHD4|CCL2|ENO1|CDC25B|PDGFRA|THBS1|ICAM1|FLT1|APP|CSF1R|KIT|ITGA2|PLK1|MMP14|DAB2|LCK|SNCA|CAV1|MAP4K4|ANXA2|FYN|FN1|FAS|SLK|PTPN1|SDC4|TLR4|XRCC5|BCL2|DYRK1A|VIM</td><td>2.68E-06</td><td>GO.0032270</td></tr>
<tr><td>33</td><td>GO Process</td><td>generation of neurons</td><td>RORA|NGEF|APP|CSF1R|KIT|ARF4|BRSK1|CALR|JAG2|PRKCH|RARB|MAP4K4|DOK4|FYN|FN1|MYH10|DLL1|FLNA|SDC4|SPTAN1|XRCC5|PPP3CA|CAMK2B|BCL2|CAMK2A|CDC42|TOP2B|WEE1|SPTBN2|CFL1|VIM|NUMB|CAMK1D</td><td>2.68E-06</td><td>GO.0048699</td></tr>
<tr><td>15</td><td>GO Process</td><td>leukocyte differentiation</td><td>MYH9|RORA|APP|CSF1R|KIT|RORC|JAG2|LCK|ANXA2|EPHA2|DLL1|ANXA1|PIR|BCL2|CDC42</td><td>2.70E-06</td><td>GO.0002521</td></tr>
<tr><td>20</td><td>GO Process</td><td>viral process</td><td>CCL2|PDGFRA|ICAM1|CCR5|ITGA2|DDB1|BCL2L1|CDC25C|ZYX|LCK|CAV1|FYN|EPHA2|DPP4|PSIP1|XRCC5|DYRK1A|CDC42|ARF1|VIM</td><td>2.74E-06</td><td>GO.0016032</td></tr>
<tr><td>26</td><td>GO Process</td><td>positive regulation of protein phosphorylation</td><td>ARHGEF5|MAP2K5|EHD4|CCL2|CDC25B|PDGFRA|THBS1|ICAM1|FLT1|APP|CSF1R|KIT|PLK1|DAB2|SNCA|CAV1|MAP4K4|ANXA2|FYN|FAS|SLK|PTPN1|SDC4|TLR4|XRCC5|BCL2</td><td>2.93E-06</td><td>GO.0001934</td></tr>
<tr><td>65</td><td>GO Process</td><td>protein metabolic process</td><td>RCN1|MAP2K5|PLOD1|MYH9|MMP2|CCL2|GAPDH|PPP3R1|CDC25B|PDGFRA|THBS1|VWF|PRKCG|AKT3|CAD|ADD1|FLT1|PLOD2|APP|CSF1R|KIT|NPC1L1|CCR5|CAPN2|MELK|PLK1|DDB1|CDC25A|ARF4|MMP14|BRSK1|TENC1|CALR|CDC25C|PRKCH|LCK|SNCA|USP1|MAP4K4|ANXA2|FYN|FN1|SFPQ|EPHA2|DPP4|TGM2|SLK|PTPN1|SPTAN1|TLR4|ANXA1|PPP3CA|CAMK2B|BCL2|CAMK2A|DYRK1A|CDC42|PDIA6|WEE1|PBK|SPTBN2|CFL1|NR1H4|PRKCSH|CAMK1D</td><td>2.93E-06</td><td>GO.0019538</td></tr>
<tr><td>22</td><td>GO Process</td><td>head development</td><td>MMP2|PDGFRA|RORA|AKT3|APP|CSF1R|CKB|ARF4|MMP14|RARB|FYN|MYH10|DLL1|FLNA|XRCC5|PPP3CA|BCL2|SUN1|RARG|TOP2B|SPTBN2|NUMB</td><td>3.04E-06</td><td>GO.0060322</td></tr>
<tr><td>19</td><td>GO Process</td><td>tissue morphogenesis</td><td>ITGB4|PPP3R1|LAMA5|CSF1R|ITGA2|NOS3|MMP14|BRSK1|JAG2|EPHA2|TGM2|DLL1|FLNA|SDC4|CLIC4|BCL2|CDC42|RARG|CFL1</td><td>3.23E-06</td><td>GO.0048729</td></tr>
<tr><td>32</td><td>GO Process</td><td>regulation of protein phosphorylation</td><td>ARHGEF5|MAP2K5|EHD4|CCL2|CDC25B|HSPB1|PDGFRA|THBS1|ICAM1|FLT1|APP|CSF1R|KIT|PLK1|CDC25A|DAB2|CDC25C|SNCA|CAV1|MAP4K4|ANXA2|FYN|FN1|FAS|EPHA2|SLK|PTPN1|SDC4|TLR4|XRCC5|BCL2|PBK</td><td>3.60E-06</td><td>GO.0001932</td></tr>
<tr><td>11</td><td>GO Process</td><td>cellular response to interferon-gamma</td><td>CCL2|GAPDH|MT2A|ICAM1|ZYX|MYO1C|TLR4|CAMK2B|CAMK2A|FLNB|VIM</td><td>3.60E-06</td><td>GO.0071346</td></tr>
<tr><td>27</td><td>GO Process</td><td>cell activation</td><td>MYH9|PDGFRA|VWF|RORA|CNN2|PRKCG|ICAM1|APP|KIT|CCT2|RORC|JAG2|PRKCH|LCK|SNCA|TUBB4B|ANXA2|FYN|DPP4|DLL1|FLNA|SPTAN1|TLR4|ANXA1|XRCC5|PPP3CA|BCL2</td><td>3.91E-06</td><td>GO.0001775</td></tr>
<tr><td>13</td><td>GO Process</td><td>regulation of actin filament organization</td><td>ARHGEF5|PLEK|ADD1|ICAM1|MYO1C|ARHGEF15|FLNA|SDC4|SPTAN1|CDC42|SPTBN2|CFL1|ARF1</td><td>3.96E-06</td><td>GO.0110053</td></tr>
<tr><td>10</td><td>GO Process</td><td>G2/M transition of mitotic cell cycle</td><td>CDC25B|TUBB4A|MELK|PLK1|TUBA1A|CDC25A|CDC25C|TUBB4B|WEE1|ABCB1</td><td>4.04E-06</td><td>GO.0000086</td></tr>
<tr><td>65</td><td>GO Process</td><td>establishment of localization</td><td>MYH9|EHD4|APLP1|PPP3R1|PLEK|HSPB1|ARF3|THBS1|VWF|CNN2|CAD|ADD1|EXOC6B|FERMT3|APP|KIT|NPC1L1|CCR5|ANXA5|ITGA2|CCT2|PLK1|TUBA1A|TUBA1C|BCL2L1|ARF4|BRSK1|DAB2|CALR|STXBP6|NR1I2|LCK|SNCA|CAV1|TUBB4B|ANXA2|ANXA6|FYN|FN1|MYO1C|MYH10|TGM2|DLL1|COPA|FLNA|SPTAN1|DNM1|TLR4|CLIC4|ANXA1|TBC1D4|PSIP1|XRCC5|MYO1B|PPP3CA|BCL2|CAMK2A|CDC42|SUN1|PDIA6|SPTBN2|ARF1|NR1H4|APBA2|ABCB1</td><td>4.55E-06</td><td>GO.0051234</td></tr>
<tr><td>18</td><td>GO Process</td><td>regulation of body fluid levels</td><td>PLEK|PDGFRA|THBS1|VWF|PRKCG|CAD|ANXA5|ITGA2|NOS3|PRKCH|LCK|CAV1|ANXA2|FYN|COPA|FLNA|TLR4|CDC42</td><td>4.81E-06</td><td>GO.0050878</td></tr>
<tr><td>18</td><td>GO Process</td><td>cellular response to lipid</td><td>CYP24A1|CCL2|RORA|ICAM1|CCR5|ITGA2|NOS3|CALR|RORC|RARB|NR1I2|RXRG|TLR4|RXRB|ANXA1|XRCC5|RARG|NR1H4</td><td>5.23E-06</td><td>GO.0071396</td></tr>
<tr><td>24</td><td>GO Process</td><td>regulation of cell development</td><td>NGEF|ADD1|APP|KIT|BRSK1|CALR|PRKCH|RARB|MAP4K4|CDH5|FYN|FN1|DLL1|FLNA|XRCC5|G6PD|PPP3CA|CAMK2B|BCL2|CDC42|CFL1|VIM|NUMB|CAMK1D</td><td>5.55E-06</td><td>GO.0060284</td></tr>
<tr><td>75</td><td>GO Process</td><td>organonitrogen compound metabolic process</td><td>RCN1|MAP2K5|PLOD1|MYH9|MMP2|CCL2|GAPDH|PPP3R1|ENO1|CDC25B|PDGFRA|THBS1|VWF|RORA|PRKCG|AKT3|CAD|ADD1|FLT1|PLOD2|APP|CSF1R|KIT|NPC1L1|CCR5|CAPN2|NOS3|MELK|CKB|PLK1|DDB1|CDC25A|ARF4|MMP14|BRSK1|TENC1|CALR|CDC25C|PRKCH|LCK|SNCA|USP1|MAP4K4|ANXA2|FYN|FN1|SFPQ|EPHA2|DPP4|TGM2|GSTO1|SLK|PTPN1|SDC4|SPTAN1|TLR4|ANXA1|SDC1|G6PD|PPP3CA|CAMK2B|LDHB|BCL2|CAMK2A|DYRK1A|PAICS|CDC42|PDIA6|WEE1|PBK|SPTBN2|CFL1|NR1H4|PRKCSH|CAMK1D</td><td>5.62E-06</td><td>GO.1901564</td></tr>
<tr><td>35</td><td>GO Process</td><td>positive regulation of signaling</td><td>ARHGEF5|MAP2K5|CCL2|PDGFRA|THBS1|AKT3|ICAM1|FLT1|APP|CSF1R|KIT|ITGA2|BCL2L1|DAB2|CALR|JAG2|PRKCH|LCK|SNCA|CAV1|MAP4K4|ANXA2|FYN|FAS|SFPQ|MYO1C|TGM2|DLL1|GSTO1|FLNA|PTPN1|TLR4|PPP3CA|BCL2|NR1H4</td><td>6.41E-06</td><td>GO.0023056</td></tr>
<tr><td>27</td><td>GO Process</td><td>epithelium development</td><td>PPP3R1|LAMA5|ICAM1|TJP1|CSF1R|ITGA2|MMP14|BRSK1|DAB2|JAG2|RARB|ARHGEF26|EPHA2|TGM2|DLL1|FLNA|CNN3|SDC4|CLIC4|ANXA1|SDC1|BCL2|CDC42|RARG|FLNB|CFL1|VIM</td><td>6.50E-06</td><td>GO.0060429</td></tr>
<tr><td>33</td><td>GO Process</td><td>homeostatic process</td><td>CCL2|RBP1|MT2A|PDGFRA|RORA|AKT3|ADD1|ICAM1|APP|KIT|CCR5|NOS3|CKB|DDB1|TENC1|CALR|LCK|SNCA|CAV1|ANXA6|TGM2|DLL1|GSTO1|TLR4|CLIC4|ANXA1|XRCC5|G6PD|PPP3CA|BCL2|PDIA6|ARF1|NR1H4</td><td>6.83E-06</td><td>GO.0042592</td></tr>
<tr><td>22</td><td>GO Process</td><td>regulation of response to external stimulus</td><td>CCL2|PLEK|HSPB1|PDGFRA|THBS1|RORA|PRKCG|APP|ITGA2|NOS3|CALR|ZYX|SNCA|CAV1|CDH5|ANXA2|TGM2|TLR4|ANXA1|PBK|NR1H4|CAMK1D</td><td>6.98E-06</td><td>GO.0032101</td></tr>
<tr><td>14</td><td>GO Process</td><td>leukocyte migration</td><td>ARHGEF5|MYH9|CCL2|ICAM1|FLT1|KIT|CCR5|LCK|CAV1|FYN|FN1|SDC4|ANXA1|SDC1</td><td>7.46E-06</td><td>GO.0050900</td></tr>
<tr><td>21</td><td>GO Process</td><td>innate immune response</td><td>CCL2|GAPDH|MT2A|ICAM1|APP|CSF1R|ZYX|LCK|SNCA|TUBB4B|FYN|SFPQ|MYO1C|TLR4|ANXA1|XRCC5|CAMK2B|CAMK2A|FLNB|VIM|NR1H4</td><td>7.80E-06</td><td>GO.0045087</td></tr>
<tr><td>63</td><td>GO Process</td><td>transport</td><td>MYH9|EHD4|APLP1|PPP3R1|PLEK|HSPB1|ARF3|THBS1|VWF|CNN2|CAD|ADD1|EXOC6B|FERMT3|APP|KIT|NPC1L1|CCR5|ANXA5|CCT2|TUBA1A|TUBA1C|BCL2L1|ARF4|BRSK1|DAB2|CALR|STXBP6|NR1I2|LCK|SNCA|CAV1|TUBB4B|ANXA2|ANXA6|FYN|FN1|MYO1C|MYH10|TGM2|DLL1|COPA|FLNA|SPTAN1|DNM1|TLR4|CLIC4|ANXA1|TBC1D4|PSIP1|XRCC5|MYO1B|PPP3CA|BCL2|CAMK2A|CDC42|SUN1|PDIA6|SPTBN2|ARF1|NR1H4|APBA2|ABCB1</td><td>8.35E-06</td><td>GO.0006810</td></tr>
<tr><td>13</td><td>GO Process</td><td>mitotic cell cycle phase transition</td><td>CDC25B|TUBB4A|MELK|PLK1|TUBA1A|CDC25A|CDC25C|TUBB4B|PPP3CA|CAMK2A|WEE1|ABCB1|MCM3</td><td>8.40E-06</td><td>GO.0044772</td></tr>
<tr><td>18</td><td>GO Process</td><td>cellular response to organic cyclic compound</td><td>CYP24A1|APLP1|CCL2|RORA|ICAM1|APP|ITGA2|CALR|RORC|RARB|NR1I2|SNCA|CAV1|RXRG|RXRB|ANXA1|RARG|NR1H4</td><td>8.40E-06</td><td>GO.0071407</td></tr>
<tr><td>23</td><td>GO Process</td><td>cellular homeostasis</td><td>CCL2|MT2A|PDGFRA|ADD1|ICAM1|APP|CCR5|NOS3|CKB|TENC1|CALR|LCK|SNCA|CAV1|ANXA6|TGM2|GSTO1|CLIC4|PPP3CA|BCL2|PDIA6|ARF1|NR1H4</td><td>8.59E-06</td><td>GO.0019725</td></tr>
<tr><td>18</td><td>GO Process</td><td>regulation of cell activation</td><td>CCL2|PLEK|PDGFRA|THBS1|RORA|APP|NOS3|MMP14|LCK|SNCA|CAV1|FYN|DPP4|SDC4|TLR4|ANXA1|BCL2|CDC42</td><td>8.59E-06</td><td>GO.0050865</td></tr>
<tr><td>19</td><td>GO Process</td><td>mitotic cell cycle process</td><td>CDC25B|TUBB4A|MELK|PLK1|TUBA1A|BCL2L1|CDC25A|BRSK1|CDC25C|TUBB4B|MYH10|FLNA|PPP3CA|CAMK2A|TOP2B|WEE1|CFL1|ABCB1|MCM3</td><td>8.83E-06</td><td>GO.1903047</td></tr>
</table>

18	GO Process	endocytosis	MYH9|EHD4|APLP1|THBS1|APP|BCL2L1|DAB2|CALR|SNCA|CAV1|FYN|MYO1C|TGM2|DLL1|DNM1|ANXA1|CDC42|PDIA6	9.45E-06	GO.0006897
20	GO Process	mitotic cell cycle	CDC25B|TUBB4A|MELK|PLK1|TUBA1A|BCL2L1|CDC25A|BRSK1|CDC25C|TUBB4B|MYH10|FLNA|PPP3CA|CAMK2A|TOP2B|WEE1|PBK|CFL1|ABCB1|MCM3	1.00E-05	GO.0000278
23	GO Process	regulation of nervous system development	NGEF|APP|KIT|BRSK1|CALR|PRKCH|RARB|SNCA|MAP4K4|FYN|FN1|ARHGEF15|DLL1|FLNA|XRCC5|PPP3CA|CAMK2B|BCL2|RARG|CFL1|VIM|NUMB|CAMK1D	1.06E-05	GO.0051960
12	GO Process	negative regulation of apoptotic signaling pathway	MAP2K5|ENO1|HSPB1|BCL2L2|THBS1|ICAM1|NOS3|BCL2L1|FYN|FAS|PTPN1|BCL2	1.11E-05	GO.2001234
57	GO Process	cellular protein metabolic process	RCN1|MAP2K5|PLOD1|MMP2|CCL2|GAPDH|PPP3R1|CDC25B|PDGFRA|THBS1|PRKCG|AKT3|CAD|FLT1|PLOD2|APP|CSF1R|KIT|CCR5|CAPN2|MELK|PLK1|DDB1|CDC25A|ARF4|BRSK1|TENC1|CDC25C|PRKCH|LCK|SNCA|USP1|MAP4K4|FYN|FN1|SFPQ|EPHA2|TGM2|SLK|PTPN1|SPTAN1|TLR4|ANXA1|PPP3CA|CAMK2B|BCL2|CAMK2A|DYRK1A|CDC42|PDIA6|WEE1|PBK|SPTBN2|CFL1|NR1H4|PRKCSH|CAMK1D	1.21E-05	GO.0044267
10	GO Process	regulation of cell shape	MYH9|CCL2|ICAM1|CSF1R|KIT|FYN|FN1|MYH10|ANXA1|SEPT7	1.30E-05	GO.0008360
16	GO Process	morphogenesis of an epithelium	PPP3R1|LAMA5|CSF1R|MMP14|BRSK1|JAG2|EPHA2|TGM2|DLL1|FLNA|SDC4|CLIC4|BCL2|CDC42|RARG|CFL1	1.31E-05	GO.0002009
31	GO Process	regulation of immune system process	MMP2|CCL2|PPP3R1|THBS1|RORA|ICAM1|APP|KIT|ITGA2|MMP14|CALR|PRKCH|LCK|SNCA|CAV1|FYN|SFPQ|MYO1C|DPP4|DLL1|PTPN1|SDC4|TLR4|ANXA1|XRCC5|PPP3CA|BCL2|CDC42|RARG|NR1H4|CAMK1D	1.32E-05	GO.0002682
24	GO Process	regulation of protein localization	ARHGEF5|GAPDH|DNAJB6|APP|CSF1R|CCT2|PLK1|BCL2L1|DAB2|PRKCH|MAP4K4|FYN|FN1|EPHA2|MYO1C|MYH10|DPP4|PTPN1|TLR4|ANXA1|PPP3CA|BCL2|NR1H4|NUMB	1.46E-05	GO.0032880
12	GO Process	T cell activation	MYH9|RORA|ICAM1|KIT|RORC|JAG2|LCK|FYN|DPP4|ANXA1|PPP3CA|BCL2	1.46E-05	GO.0042110
34	GO Process	positive regulation of cell communication	ARHGEF5|MAP2K5|CCL2|PDGFRA|THBS1|AKT3|ICAM1|FLT1|APP|CSF1R|KIT|ITGA2|BCL2L1|DAB2|CALR|JAG2|PRKCH|LCK|SNCA|CAV1|MAP4K4|FYN|FAS|SFPQ|MYO1C|TGM2|DLL1|GSTO1|FLNA|PTPN1|TLR4|PPP3CA|BCL2|NR1H4	1.52E-05	GO.0010647
20	GO Process	epithelial cell differentiation	PPP3R1|LAMA5|ICAM1|TJP1|ITGA2|DAB2|JAG2|RARB|ARHGEF26|EPHA2|DLL1|FLNA|CNN3|CLIC4|ANXA1|SDC1|CDC42|RARG|FLNB|VIM	1.55E-05	GO.0030855
8	GO Process	regulation of coagulation	PLEK|PDGFRA|THBS1|ANXA5|NOS3|CAV1|ANXA2|TLR4	1.55E-05	GO.0050818
16	GO Process	regulation of protein complex assembly	ARHGEF5|PLEK|TUBB4A|ADD1|ICAM1|DDB1|STXBP6|SNCA|MYO1C|SPTAN1|TLR4|FSCN1|DYRK1A|CDC42|SPTBN2|ARF1	1.77E-05	GO.0043254
8	GO Process	regulation of cell junction assembly	THBS1|TJP1|MMP14|PRKCH|CAV1|MYO1C|SLK|SDC4	1.82E-05	GO.1901888
17	GO Process	inflammatory response	CCL2|THBS1|ICAM1|APP|CSF1R|KIT|CCR5|SNCA|PTGES|FN1|FAS|TLR4|ANXA1|SDC1|LTB4R|NR1H4|CAMK1D	1.87E-05	GO.0006954
32	GO Process	positive regulation of signal transduction	ARHGEF5|MAP2K5|CCL2|PDGFRA|THBS1|AKT3|ICAM1|FLT1|APP|CSF1R|KIT|BCL2L1|DAB2|CALR|JAG2|PRKCH|LCK|CAV1|MAP4K4|FYN|FAS|SFPQ|MYO1C|TGM2|DLL1|GSTO1|FLNA|PTPN1|TLR4|PPP3CA|BCL2|NR1H4	1.87E-05	GO.0009967
15	GO Process	positive regulation of cell adhesion	CCL2|COL8A1|ICAM1|ITGA2|CALR|LCK|CAV1|FYN|FN1|DPP4|TGM2|FLNA|SDC4|ANXA1|CDC42	1.89E-05	GO.0045785
8	GO Process	integrin-mediated signaling pathway	ITGB4|MYH9|PLEK|LAMA5|FERMT3|ITGA2|ZYX|CDC42	1.94E-05	GO.0007229
44	GO Process	regulation of cellular protein metabolic process	ARHGEF5|MAP2K5|MYH9|EHD4|CCL2|GAPDH|ENO1|CDC25B|HSPB1|PDGFRA|THBS1|DNAJB6|PRKCG|ICAM1|FLT1|APP|CSF1R|KIT|ITGA2|PLK1|CDC25A|MMP14|DAB2|CALR|CDC25C|LCK|SNCA|CAV1|MAP4K4|ANXA2|FYN|FN1|FAS|EPHA2|SLK|PTPN1|SDC4|TLR4|XRCC5|G6PD|BCL2|DYRK1A|PBK|VIM	2.07E-05	GO.0032268
46	GO Process	regulation of protein metabolic process	ARHGEF5|MAP2K5|MYH9|EHD4|CCL2|GAPDH|ENO1|CDC25B|HSPB1|PDGFRA|THBS1|DNAJB6|PRKCG|ICAM1|FLT1|APP|CSF1R|KIT|ITGA2|PLK1|DDB1|CDC25A|MMP14|DAB2|CALR|CDC25C|LCK|SNCA|CAV1|MAP4K4|ANXA2|FYN|FN1|FAS|EPHA2|SLK|FLNA|PTPN1|SDC4|TLR4|XRCC5|G6PD|BCL2|DYRK1A|PBK|VIM	2.22E-05	GO.0051246
18	GO Process	response to wounding	ITGB4|PDGFRA|VWF|PRKCG|ANXA5|ITGA2|ARF4|PRKCH|LCK|FYN|FN1|MYH10|FLNA|SDC4|ANXA1|SDC1|BCL2|CDC42	2.25E-05	GO.0009611
19	GO Process	regulation of cell projection organization	NGEF|ICAM1|APP|KIT|ITGA2|BRSK1|CAV1|MAP4K4|FYN|FN1|EPHA2|FSCN1|PPP3CA|CAMK2B|SEPT7|CDC42|CFL1|VIM|CAMK1D	2.33E-05	GO.0031344
35	GO Process	regulation of protein modification process	ARHGEF5|MAP2K5|EHD4|CCL2|CDC25B|HSPB1|PDGFRA|THBS1|PRKCG|ICAM1|FLT1|APP|CSF1R|KIT|PLK1|CDC25A|DAB2|CDC25C|SNCA|CAV1|MAP4K4|ANXA2|FYN|FN1|FAS|EPHA2|SLK|PTPN1|SDC4|TLR4|XRCC5|G6PD|BCL2|DYRK1A|PBK	2.35E-05	GO.0031399
11	GO Process	peptidyl-tyrosine phosphorylation	MAP2K5|PDGFRA|FLT1|CSF1R|KIT|MELK|LCK|FYN|EPHA2|DYRK1A|WEE1	2.40E-05	GO.0018108
18	GO Process	positive regulation of organelle organization	ARHGEF5|PLEK|ICAM1|CCT2|ANXA2|SFPQ|MYO1C|ARHGEF15|FLNA|SDC4|ANXA1|SDC1|FSCN1|XRCC5|BCL2|SEPT7|CDC42|CFL1	2.50E-05	GO.0010638
8	GO Process	regulation of blood vessel endothelial cell migration	MAP2K5|HSPB1|THBS1|AKT3|NOS3|EPHA2|MMRN2|ANXA1	2.59E-05	GO.0043535
11	GO Process	cellular response to steroid hormone stimulus	RORA|ICAM1|CALR|RORC|RARB|NR1I2|RXRG|RXRB|ANXA1|RARG|NR1H4	2.60E-05	GO.0071383
27	GO Process	positive regulation of protein modification process	ARHGEF5|MAP2K5|EHD4|CCL2|CDC25B|PDGFRA|THBS1|ICAM1|FLT1|APP|CSF1R|KIT|PLK1|DAB2|SNCA|CAV1|MAP4K4|ANXA2|FYN|FAS|SLK|PTPN1|SDC4|TLR4|XRCC5|BCL2|DYRK1A	2.69E-05	GO.0031401
15	GO Process	regulation of apoptotic signaling pathway	MAP2K5|ENO1|HSPB1|BCL2L2|THBS1|ICAM1|NOS3|BCL2L1|LCK|CAV1|FYN|FAS|SFPQ|PTPN1|BCL2	2.69E-05	GO.2001233
11	GO Process	actin filament organization	ADD1|ZYX|TPM4|TPM3|FLNA|TPM2|FSCN1|MYO1B|BCL2|CDC42|CFL1	2.93E-05	GO.0007015
23	GO Process	response to organonitrogen compound	MMP2|APLP1|PDGFRA|PRKCG|CAD|ICAM1|APP|CAPN2|ITGA2|BCL2L1|SNCA|CAV1|FYN|PTPN1|DNM1|TLR4|ANXA1|TBC1D4|SDC1|PPP3CA|CAMK2A|CFL1|NR1H4	2.93E-05	GO.0010243
23	GO Process	positive regulation of cell population proliferation	MAP2K5|MMP2|CDC25B|PDGFRA|THBS1|AKT3|FLT1|CSF1R|KIT|ITGA2|BCL2L1|CALR|PRKCH|RARB|ANXA2|FN1|DPP4|TGM2|DLL1|FLNA|ANXA1|BCL2|RARG	3.02E-05	GO.0008284
34	GO Process	regulation of cell differentiation	PDGFRA|THBS1|RORA|NGEF|ADD1|APP|KIT|MMP14|BRSK1|DAB2|CALR|RORC|PRKCH|RARB|CAV1|MAP4K4|CDH5|FYN|FN1|DLL1|FLNA|TLR4|ANXA1|XRCC5|G6PD|PPP3CA|CAMK2B|BCL2|CDC42|RARG|CFL1|VIM|NUMB|CAMK1D	3.20E-05	GO.0045595
17	GO Process	response to growth factor	MAP2K5|EHD4|CCL2|HSPB1|PDGFRA|THBS1|CAD|FLT1|APP|ZYX|SNCA|CAV1|CDH5|FYN|EPHA2|MYO1C|ANXA1	3.34E-05	GO.0070848
19	GO Process	positive regulation of transferase activity	ARHGEF5|MAP2K5|CDC25B|PDGFRA|THBS1|FLT1|APP|CSF1R|KIT|CCT2|PLK1|SNCA|MAP4K4|FYN|SLK|PTPN1|SDC4|TLR4|XRCC5	3.65E-05	GO.0051347
23	GO Process	embryo development	ITGB4|MYH9|MMP2|LAMA5|PDGFRA|COL8A1|ADD1|FLT1|KIT|CAPN2|ITGA2|NOS3|BCL2L1|MMP14|JAG2|RARB|FN1|MYH10|DLL1|SDC4|RARG|CFL1|APBA2	3.71E-05	GO.0009790
24	GO Process	positive regulation of intracellular signal transduction	ARHGEF5|MAP2K5|CCL2|PDGFRA|THBS1|AKT3|ICAM1|FLT1|APP|CSF1R|KIT|BCL2L1|CALR|LCK|CAV1|MAP4K4|FYN|SFPQ|TGM2|GSTO1|PTPN1|TLR4|PPP3CA|BCL2	3.73E-05	GO.1902533
17	GO Process	negative regulation of intracellular signal transduction	APLP1|PLEK|ENO1|HSPB1|BCL2L2|THBS1|RORA|BCL2L1|CAV1|EPHA2|GSTO1|PTPN1|TLR4|BCL2|DYRK1A|PBK|NR1H4	3.93E-05	GO.1902532
10	GO Process	cell-cell junction organization	LAMA5|TJP1|CSF1R|ITGA2|CDH5|FSCN1|BCL2|NR1H4|NUMB|TLN2	4.36E-05	GO.0045216
21	GO Process	regulation of cellular localization	APP|CCT2|PLK1|BCL2L1|BRSK1|DAB2|PRKCH|SNCA|CAV1|MAP4K4|ANXA2|FYN|EPHA2|MYO1C|GSTO1|PTPN1|DNM1|BCL2|CAMK2A|NR1H4|NUMB	4.36E-05	GO.0060341
10	GO Process	regulation of reactive oxygen species metabolic process	THBS1|ICAM1|APP|ARF4|SNCA|CAV1|FYN|TLR4|G6PD|BCL2	4.57E-05	GO.2000377
9	GO Process	steroid hormone mediated signaling pathway	RORA|CALR|RORC|RARB|NR1I2|RXRG|RXRB|RARG|NR1H4	4.63E-05	GO.0043401
9	GO Process	male gonad development	BCL2L2|PDGFRA|ICAM1|KIT|BCL2L1|MMP14|FLNA|SDC1|BCL2	4.89E-05	GO.0008584
18	GO Process	cellular response to hormone stimulus	RORA|ICAM1|KIT|ITGA2|CALR|RORC|RARB|NR1I2|CAV1|FYN|RXRG|PTPN1|RXRB|ANXA1|TBC1D4|CAMK2A|RARG|NR1H4	5.08E-05	GO.0032870
13	GO Process	positive regulation of cell activation	CCL2|PLEK|THBS1|APP|MMP14|LCK|CAV1|FYN|DPP4|TLR4|ANXA1|BCL2|CDC42	5.16E-05	GO.0050867
22	GO Process	cell adhesion	ITGB4|APLP1|CCL2|LAMA4|LAMA5|PDGFRA|THBS1|COL8A1|VWF|ICAM1|FERMT3|APP|KIT|ITGA2|ZYX|CDH5|FN1|EPHA2|MYH10|DPP4|BCL2|TLN2	5.19E-05	GO.0007155
10	GO Process	intracellular receptor signaling pathway	CYP24A1|RORA|CALR|RORC|RARB|NR1I2|RXRG|RXRB|RARG|NR1H4	5.42E-05	GO.0030522

22	GO Process	regulation of kinase activity	ARHGEF5|MAP2K5|CDC25B|HSPB1|PDGFRA|THBS1|FLT1|APP|CSF1R|KIT|PLK1|CDC25A|CDC25C|SNCA|CAV1|MAP4K4|FYN|SLK|PTPN1|SDC4|TLR4|XRCC5	5.75E-05	GO.0043549
24	GO Process	response to nitrogen compound	MMP2|APLP1|PDGFRA|PRKCG|CAD|ICAM1|APP|CAPN2|ITGA2|BCL2L1|NQO1|SNCA|CAV1|FYN|PTPN1|DNM1|TLR4|ANXA1|TBC1D4|SDC1|PPP3CA|CAMK2A|CFL1|NR1H4	5.82E-05	GO.1901698
16	GO Process	cell part morphogenesis	APP|CSF1R|BCL2L1|BRSK1|MAP4K4|DOK4|FYN|MYH10|SPTAN1|BCL2|CAMK2A|CDC42|TOP2B|WEE1|SPTBN2|NUMB	5.85E-05	GO.0032990
41	GO Process	cellular component assembly	ITGB4|EHD4|LAMA5|PDGFRA|VWF|TUBB4A|ADD1|FERMT3|TJP1|APP|KIT|ITGA2|CCT2|PLK1|TUBA1A|DDB1|CALR|ZYX|FLNC|CAV1|TUBB4B|CDH5|ANXA2|ANXA6|FN1|FAS|ARHGEF26|RXRG|MYH10|TGM2|FLNA|DNM1|PSIP1|FSCN1|MYO1B|BCL2|SEPT7|CDC42|SPTBN2|NR1H4|TLN2	6.26E-05	GO.0022607
16	GO Process	cellular response to growth factor stimulus	MAP2K5|EHD4|CCL2|HSPB1|PDGFRA|THBS1|CAD|FLT1|APP|ZYX|SNCA|CAV1|CDH5|FYN|MYO1C|ANXA1	6.27E-05	GO.0071363
21	GO Process	regulation of protein kinase activity	ARHGEF5|MAP2K5|CDC25B|HSPB1|THBS1|FLT1|APP|CSF1R|KIT|PLK1|CDC25A|CDC25C|SNCA|CAV1|MAP4K4|FYN|SLK|PTPN1|SDC4|TLR4|XRCC5	6.29E-05	GO.0045859
8	GO Process	myeloid leukocyte differentiation	MYH9|APP|CSF1R|KIT|ANXA2|EPHA2|PIR|CDC42	6.39E-05	GO.0002573
18	GO Process	regulation of plasma membrane bounded cell projection organization	NGEF|ICAM1|APP|KIT|BRSK1|CAV1|MAP4K4|FYN|FN1|EPHA2|FSCN1|PPP3CA|CAMK2B|SEPT7|CDC42|CFL1|VIM|CAMK1D	6.80E-05	GO.0120035
20	GO Process	regulation of neurogenesis	NGEF|APP|KIT|BRSK1|CALR|PRKCH|RARB|MAP4K4|FYN|FN1|DLL1|FLNA|XRCC5|PPP3CA|CAMK2B|BCL2|CFL1|VIM|NUMB|CAMK1D	7.23E-05	GO.0050767
10	GO Process	positive regulation of supramolecular fiber organization	ARHGEF5|PLEK|ICAM1|APP|MYO1C|ARHGEF15|FLNA|SDC4|CDC42|CFL1	7.23E-05	GO.1902905
8	GO Process	negative regulation of extrinsic apoptotic signaling pathway	MAP2K5|THBS1|ICAM1|NOS3|BCL2L1|FYN|FAS|BCL2	7.23E-05	GO.2001237
11	GO Process	regulation of muscle system process	ENO1|KIT|ITGA2|NOS3|CAV1|ANXA6|GSTO1|FLNA|G6PD|PPP3CA|CAMK2B	7.35E-05	GO.0090257
14	GO Process	response to oxidative stress	PXDN|PDGFRA|APP|NOS3|MELK|MMP14|NQO1|SNCA|FYN|ANXA1|PSIP1|SDC1|G6PD|BCL2	7.40E-05	GO.0006979
18	GO Process	microtubule-based process	MYH9|GAPDH|HSPB1|TUBB4A|APP|PLK1|TUBA1A|TUBA1C|BRSK1|TUBB4B|MYO1C|MYH10|SLK|FLNA|MYO1B|CDC42|SUN1|WEE1	7.43E-05	GO.0007017
13	GO Process	response to oxygen levels	PLOD1|MMP2|THBS1|RORA|ICAM1|PLOD2|CAPN2|ITGA2|MMP14|CAV1|FAS|DPP4|BCL2	7.43E-05	GO.0070482
8	GO Process	actin filament-based movement	MYH9|TPM4|MYO1C|MYH10|TPM3|TPM2|MYO1B|VIM	7.56E-05	GO.0030048
13	GO Process	response to acid chemical	MMP2|PDGFRA|ICAM1|CAPN2|ITGA2|BCL2L1|NQO1|PTGES|FYN|XRCC5|RARG|CFL1|NR1H4	7.86E-05	GO.0001101
16	GO Process	regulation of protein serine/threonine kinase activity	ARHGEF5|MAP2K5|HSPB1|THBS1|FLT1|APP|CSF1R|KIT|PLK1|CDC25A|CDC25C|SNCA|CAV1|MAP4K4|PTPN1|TLR4	7.90E-05	GO.0071900
6	GO Process	interleukin-12-mediated signaling pathway	CNN2|ANXA2|GSTO1|CDC42|CFL1|ARF1	7.96E-05	GO.0035722
16	GO Process	positive regulation of cell development	ADD1|APP|KIT|CALR|PRKCH|RARB|CDH5|FYN|FN1|FLNA|XRCC5|CAMK2B|BCL2|CDC42|NUMB|CAMK1D	8.39E-05	GO.0010720
17	GO Process	positive regulation of kinase activity	ARHGEF5|MAP2K5|CDC25B|PDGFRA|THBS1|FLT1|APP|CSF1R|KIT|SNCA|MAP4K4|FYN|SLK|PTPN1|SDC4|TLR4|XRCC5	8.86E-05	GO.0033674
22	GO Process	positive regulation of immune system process	MMP2|CCL2|THBS1|ICAM1|APP|ITGA2|MMP14|CALR|PRKCH|LCK|CAV1|FYN|SFPQ|MYO1C|DPP4|TLR4|ANXA1|XRCC5|BCL2|CDC42|NR1H4|CAMK1D	9.49E-05	GO.0002684
31	GO Process	cellular response to stress	MAP2K5|CCL2|BCL2L2|PDGFRA|THBS1|RORA|ADD1|ICAM1|NPC1L1|NOS3|MELK|PLK1|DDB1|BCL2L1|BRSK1|NQO1|CALR|CDC25C|SNCA|CAV1|USP1|FAS|SFPQ|EPHA2|SLK|PTPN1|ANXA1|XRCC5|G6PD|BCL2|PDIA6	9.49E-05	GO.0033554
18	GO Process	regulation of cellular response to stress	ARHGEF5|ENO1|HSPB1|DNAJB6|PRKCG|AKT3|APP|BCL2L1|CAV1|USP1|MAP4K4|FYN|FAS|SFPQ|PTPN1|TLR4|DYRK1A|PBK	9.49E-05	GO.0080135
7	GO Process	regulation of blood coagulation	PLEK|PDGFRA|THBS1|NOS3|CAV1|ANXA2|TLR4	9.85E-05	GO.0030193
10	GO Process	regulation of cell-substrate adhesion	THBS1|COL8A1|MMP14|CALR|FN1|SLK|FLNA|SDC4|BCL2|CDC42	1.00E-04	GO.0010810
9	GO Process	regulation of endothelial cell migration	MAP2K5|HSPB1|THBS1|AKT3|NOS3|CALR|EPHA2|MMRN2|ANXA1	1.10E-04	GO.0010594
22	GO Process	cell cycle process	MYH9|CDC25B|THBS1|TUBB4A|MELK|PLK1|TUBA1A|BCL2L1|CDC25A|BRSK1|CDC25C|TUBB4B|MYH10|FLNA|PPP3CA|CAMK2A|SUN1|TOP2B|WEE1|CFL1|ABCB1|MCM3	1.10E-04	GO.0022402
22	GO Process	leukocyte activation	MYH9|RORA|CNN2|ICAM1|APP|KIT|CCT2|RORC|JAG2|LCK|SNCA|TUBB4B|ANXA2|FYN|DPP4|DLL1|SPTAN1|TLR4|ANXA1|XRCC5|PPP3CA|BCL2	1.10E-04	GO.0045321
23	GO Process	regulation of transferase activity	ARHGEF5|MAP2K5|CDC25B|HSPB1|PDGFRA|THBS1|FLT1|APP|CSF1R|KIT|CCT2|PLK1|CDC25A|CDC25C|SNCA|CAV1|MAP4K4|FYN|SLK|PTPN1|SDC4|TLR4|XRCC5	1.10E-04	GO.0051338
11	GO Process	regulation of calcium ion transport	CCL2|ICAM1|NOS3|SNCA|CAV1|FYN|GSTO1|G6PD|CAMK2B|BCL2|CAMK2A	1.10E-04	GO.0051924
12	GO Process	response to hypoxia	PLOD1|MMP2|THBS1|RORA|ICAM1|PLOD2|CAPN2|ITGA2|MMP14|CAV1|DPP4|BCL2	1.20E-04	GO.0001666
22	GO Process	response to drug	MT2A|THBS1|PRKCG|CAD|ICAM1|NPC1L1|ITGA2|BCL2L1|NQO1|CALR|NR1I2|LCK|SNCA|CAV1|FYN|ANXA1|SDC1|XRCC5|G6PD|PPP3CA|BCL2|ABCB1	1.20E-04	GO.0042493
15	GO Process	neuron projection morphogenesis	APP|CSF1R|BRSK1|MAP4K4|DOK4|FYN|MYH10|SPTAN1|BCL2|CAMK2A|CDC42|TOP2B|WEE1|SPTBN2|NUMB	1.20E-04	GO.0048812
10	GO Process	gonad development	BCL2L2|PDGFRA|ICAM1|KIT|NOS3|BCL2L1|MMP14|FLNA|SDC1|BCL2	1.40E-04	GO.0008406
7	GO Process	multicellular organism growth	ADD1|TENC1|RARB|CLIC4|RARG|SPTBN2|APBA2	1.40E-04	GO.0035264
16	GO Process	positive regulation of protein kinase activity	ARHGEF5|MAP2K5|CDC25B|THBS1|FLT1|APP|CSF1R|KIT|SNCA|MAP4K4|FYN|SLK|PTPN1|SDC4|TLR4|XRCC5	1.40E-04	GO.0045860
22	GO Process	negative regulation of developmental process	MAP2K5|THBS1|RORA|AKT3|NGEF|APP|NOS3|CALR|RARB|CAV1|MAP4K4|ANXA2|ARHGEF15|DLL1|MMRN2|TLR4|ANXA1|G6PD|PPP3CA|BCL2|RARG|VIM	1.40E-04	GO.0051093
38	GO Process	cellular localization	MYH9|EHD4|PPP3R1|PLEK|HSPB1|LAMA5|TUBB4A|EXOC6B|APP|KIT|CCR5|PLK1|TUBA1A|TUBA1C|ARF4|BRSK1|CALR|STXBP6|LCK|SNCA|CAV1|TUBB4B|ANXA2|MYO1C|MYH10|COPA|FLNA|SPTAN1|DNM1|TBC1D4|PSIP1|XRCC5|PPP3CA|CDC42|SUN1|FLNB|SPTBN2|TLN2	1.50E-04	GO.0051641
12	GO Process	positive regulation of leukocyte activation	CCL2|THBS1|APP|MMP14|LCK|CAV1|FYN|DPP4|TLR4|ANXA1|BCL2|CDC42	1.60E-04	GO.0002696
7	GO Process	female gonad development	PDGFRA|ICAM1|KIT|NOS3|BCL2L1|MMP14|BCL2	1.60E-04	GO.0008585
13	GO Process	signal transduction by protein phosphorylation	MAP2K5|CCL2|PDGFRA|KIT|CCR5|MAP4K4|FYN|SLK|PTPN1|SPTAN1|CAMK2B|CAMK2A|SPTBN2	1.60E-04	GO.0023014
15	GO Process	wound healing	PDGFRA|VWF|PRKCG|ANXA5|ITGA2|PRKCH|LCK|FYN|FN1|MYH10|FLNA|SDC4|ANXA1|SDC1|CDC42	1.60E-04	GO.0042060
9	GO Process	regulation of extrinsic apoptotic signaling pathway	MAP2K5|THBS1|ICAM1|NOS3|BCL2L1|CAV1|FYN|FAS|BCL2	1.60E-04	GO.2001236
9	GO Process	regulation of intrinsic apoptotic signaling pathway	ENO1|HSPB1|BCL2L2|BCL2L1|LCK|CAV1|SFPQ|PTPN1|BCL2	1.60E-04	GO.2001242
18	GO Process	brain development	RORA|AKT3|APP|CSF1R|CKB|ARF4|RARB|FYN|MYH10|DLL1|FLNA|XRCC5|PPP3CA|BCL2|SUN1|TOP2B|SPTBN2|NUMB	1.70E-04	GO.0007420
10	GO Process	myeloid cell differentiation	MYH9|ADD1|APP|CSF1R|KIT|ANXA2|EPHA2|PIR|G6PD|CDC42	1.70E-04	GO.0030099
24	GO Process	secretion	PLEK|THBS1|VWF|CNN2|CAD|EXOC6B|FERMT3|APP|KIT|ANXA5|CCT2|BRSK1|CAV1|TUBB4B|ANXA2|FN1|MYH10|COPA|FLNA|SPTAN1|TLR4|ANXA1|XRCC5|NR1H4	1.80E-04	GO.0046903
15	GO Process	regulation of leukocyte activation	CCL2|THBS1|RORA|APP|MMP14|LCK|SNCA|CAV1|FYN|DPP4|SDC4|TLR4|ANXA1|BCL2|CDC42	1.90E-04	GO.0002694
6	GO Process	actin cytoskeleton reorganization	MYH9|PLEK|KIT|FLNA|PTPN1|ANXA1	1.90E-04	GO.0031532
13	GO Process	lymphocyte activation	MYH9|RORA|ICAM1|KIT|RORC|JAG2|LCK|FYN|DPP4|DLL1|ANXA1|PPP3CA|BCL2	2.00E-04	GO.0046649
8	GO Process	regulation of wound healing	PLEK|PDGFRA|THBS1|NOS3|CAV1|ANXA2|TLR4|ANXA1	2.00E-04	GO.0061041
12	GO Process	regulation of vasculature development	MAP2K5|HSPB1|THBS1|AKT3|FLT1|KIT|NOS3|CDH5|EPHA2|DLL1|MMRN2|ANXA1	2.00E-04	GO.1901342

<table>
<tr><td>21</td><td>GO Process</td><td>animal organ morphogenesis</td><td>ITGB4|MMP2|APLP1|CCL2|LAMA5|PDGFRA|COL8A1|AKT3|CSF1R|ITGA2|NOS3|MMP14|JAG2|RARB|CAV1|EPHA2|TGM2|DLL1|SDC1|BCL2|RARG</td><td>2.10E-04</td><td>GO.0009887</td></tr>
<tr><td>10</td><td>GO Process</td><td>regulation of epithelial cell migration</td><td>MAP2K5|HSPB1|THBS1|AKT3|ITGA2|NOS3|CALR|EPHA2|MMRN2|ANXA1</td><td>2.10E-04</td><td>GO.0010632</td></tr>
<tr><td>6</td><td>GO Process</td><td>negative regulation of protein polymerization</td><td>TUBB4A|ADD1|SNCA|SPTAN1|DYRK1A|SPTBN2</td><td>2.10E-04</td><td>GO.0032272</td></tr>
<tr><td>18</td><td>GO Process</td><td>cellular chemical homeostasis</td><td>MT2A|PDGFRA|ICAM1|APP|CCR5|CKB|CALR|LCK|SNCA|CAV1|ANXA6|TGM2|GSTO1|CLIC4|PPP3CA|BCL2|ARF1|NR1H4</td><td>2.20E-04</td><td>GO.0055082</td></tr>
<tr><td>9</td><td>GO Process</td><td>regulation of cell cycle G2/M phase transition</td><td>CDC25B|TUBB4A|APP|PLK1|TUBA1A|CDC25A|BRSK1|CDC25C|TUBB4B</td><td>2.30E-04</td><td>GO.1902749</td></tr>
<tr><td>6</td><td>GO Process</td><td>positive regulation of actin filament bundle assembly</td><td>ARHGEF5|PLEK|ARHGEF15|FLNA|SDC4|CDC42</td><td>2.40E-04</td><td>GO.0032233</td></tr>
<tr><td>17</td><td>GO Process</td><td>regulation of mitotic cell cycle</td><td>MAP2K5|CCL2|CDC25B|TUBB4A|APP|PLK1|TUBA1A|DDB1|BCL2L1|BRSK1|CDC25C|TUBB4B|MAP4K4|SLK|ANXA1|BCL2|TOP2B</td><td>2.50E-04</td><td>GO.0007346</td></tr>
<tr><td>8</td><td>GO Process</td><td>platelet degranulation</td><td>PLEK|THBS1|VWF|FERMT3|APP|ANXA5|FN1|FLNA</td><td>2.60E-04</td><td>GO.0002576</td></tr>
<tr><td>15</td><td>GO Process</td><td>cellular response to organonitrogen compound</td><td>MMP2|APLP1|PDGFRA|ICAM1|APP|CAPN2|BCL2L1|SNCA|CAV1|FYN|PTPN1|TLR4|TBC1D4|CAMK2A|NR1H4</td><td>2.60E-04</td><td>GO.0071417</td></tr>
<tr><td>4</td><td>GO Process</td><td>vascular endothelial growth factor signaling pathway</td><td>HSPB1|PDGFRA|FLT1|MYO1C</td><td>2.70E-04</td><td>GO.0038084</td></tr>
<tr><td>7</td><td>GO Process</td><td>gland morphogenesis</td><td>LAMA5|CSF1R|CAV1|EPHA2|TGM2|BCL2|RARG</td><td>2.80E-04</td><td>GO.0022612</td></tr>
<tr><td>15</td><td>GO Process</td><td>chemotaxis</td><td>ARHGEF5|CCL2|PDGFRA|FLT1|APP|CSF1R|KIT|CCR5|DOK4|FYN|EPHA2|MYH10|SPTAN1|ANXA1|SPTBN2</td><td>2.90E-04</td><td>GO.0006935</td></tr>
<tr><td>9</td><td>GO Process</td><td>peptidyl-serine phosphorylation</td><td>PRKCG|AKT3|PLK1|PRKCH|CAMK2B|BCL2|CAMK2A|DYRK1A|CAMK1D</td><td>2.90E-04</td><td>GO.0018105</td></tr>
<tr><td>17</td><td>GO Process</td><td>neuron projection development</td><td>APP|CSF1R|ARF4|BRSK1|MAP4K4|DOK4|FYN|MYH10|SDC4|SPTAN1|BCL2|CAMK2A|CDC42|TOP2B|WEE1|SPTBN2|NUMB</td><td>2.90E-04</td><td>GO.0031175</td></tr>
<tr><td>5</td><td>GO Process</td><td>cellular response to vascular endothelial growth factor stimulus</td><td>HSPB1|PDGFRA|FLT1|MYO1C|ANXA1</td><td>2.90E-04</td><td>GO.0035924</td></tr>
<tr><td>9</td><td>GO Process</td><td>regulation of leukocyte migration</td><td>CCL2|THBS1|ICAM1|APP|ITGA2|MMP14|CALR|ANXA1|CAMK1D</td><td>3.10E-04</td><td>GO.0002685</td></tr>
<tr><td>12</td><td>GO Process</td><td>MAPK cascade</td><td>MAP2K5|CCL2|PDGFRA|KIT|CCR5|MAP4K4|FYN|PTPN1|SPTAN1|CAMK2B|CAMK2A|SPTBN2</td><td>3.20E-04</td><td>GO.0000165</td></tr>
<tr><td>4</td><td>GO Process</td><td>retinoic acid receptor signaling pathway</td><td>RARB|RXRG|RXRB|RARG</td><td>3.20E-04</td><td>GO.0048384</td></tr>
<tr><td>5</td><td>GO Process</td><td>regulation of cell migration involved in sprouting angiogenesis</td><td>MAP2K5|THBS1|AKT3|MMRN2|ANXA1</td><td>3.20E-04</td><td>GO.0090049</td></tr>
<tr><td>27</td><td>GO Process</td><td>reproductive process</td><td>MYH9|MMP2|CDC25B|BCL2L2|PDGFRA|CAD|ICAM1|APP|KIT|ITGA2|NOS3|CCT2|PLK1|BCL2L1|MMP14|CALR|CDC25C|JAG2|FLNA|CLIC4|ANXA1|SDC1|BCL2|SEPT7|SUN1|RARG|TOP2B</td><td>3.30E-04</td><td>GO.0022414</td></tr>
<tr><td>15</td><td>GO Process</td><td>positive regulation of cellular component biogenesis</td><td>ARHGEF5|PLEK|ICAM1|KIT|DDB1|CAV1|MYO1C|ARHGEF15|FLNA|SDC4|TLR4|SDC1|FSCN1|SEPT7|CDC42</td><td>3.30E-04</td><td>GO.0044089</td></tr>
<tr><td>6</td><td>GO Process</td><td>regulation of sprouting angiogenesis</td><td>MAP2K5|THBS1|AKT3|DLL1|MMRN2|ANXA1</td><td>3.30E-04</td><td>GO.1903670</td></tr>
<tr><td>15</td><td>GO Process</td><td>transmembrane receptor protein tyrosine kinase signaling pathway</td><td>MMP2|HSPB1|PDGFRA|NGEF|FLT1|CSF1R|KIT|ARF4|LCK|FYN|EPHA2|MYO1C|PTPN1|DNM1|CDC42</td><td>3.40E-04</td><td>GO.0007169</td></tr>
<tr><td>9</td><td>GO Process</td><td>positive regulation of vasculature development</td><td>HSPB1|THBS1|AKT3|FLT1|KIT|NOS3|CDH5|DLL1|ANXA1</td><td>3.60E-04</td><td>GO.1904018</td></tr>
<tr><td>18</td><td>GO Process</td><td>enzyme linked receptor protein signaling pathway</td><td>MMP2|HSPB1|PDGFRA|NGEF|FLT1|CSF1R|KIT|ARF4|ZYX|LCK|CDH5|FYN|EPHA2|MYO1C|PTPN1|DNM1|CDC42|VIM</td><td>3.70E-04</td><td>GO.0007167</td></tr>
<tr><td>6</td><td>GO Process</td><td>regulation of phospholipase activity</td><td>PDGFRA|FLT1|KIT|ARF4|SNCA|ANXA1</td><td>3.70E-04</td><td>GO.0010517</td></tr>
<tr><td>13</td><td>GO Process</td><td>regulation of cell cycle phase transition</td><td>CCL2|CDC25B|TUBB4A|APP|PLK1|TUBA1A|DDB1|CDC25A|BRSK1|CDC25C|TUBB4B|ANXA1|BCL2</td><td>3.70E-04</td><td>GO.1901987</td></tr>
<tr><td>11</td><td>GO Process</td><td>regulation of angiogenesis</td><td>MAP2K5|HSPB1|THBS1|AKT3|FLT1|NOS3|CDH5|EPHA2|DLL1|MMRN2|ANXA1</td><td>3.80E-04</td><td>GO.0045765</td></tr>
<tr><td>26</td><td>GO Process</td><td>positive regulation of developmental process</td><td>HSPB1|THBS1|AKT3|ADD1|FLT1|APP|KIT|NOS3|MMP14|DAB2|CALR|PRKCH|RARB|CDH5|FYN|FN1|DLL1|FLNA|ANXA1|XRCC5|CAMK2B|BCL2|CDC42|NR1H4|NUMB|CAMK1D</td><td>3.90E-04</td><td>GO.0051094</td></tr>
<tr><td>9</td><td>GO Process</td><td>striated muscle cell differentiation</td><td>MYH9|PDGFRA|CAPN2|CALR|FLNC|RARB|MYH10|SDC1|PPP3CA</td><td>4.00E-04</td><td>GO.0051146</td></tr>
<tr><td>10</td><td>GO Process</td><td>muscle cell differentiation</td><td>MYH9|PDGFRA|RORA|CAPN2|CALR|FLNC|RARB|MYH10|SDC1|PPP3CA</td><td>4.10E-04</td><td>GO.0042692</td></tr>
<tr><td>5</td><td>GO Process</td><td>cell-substrate junction assembly</td><td>ITGB4|LAMA5|ITGA2|FN1|BCL2</td><td>4.30E-04</td><td>GO.0007044</td></tr>
<tr><td>5</td><td>GO Process</td><td>regulation of macrophage activation</td><td>THBS1|RORA|APP|SNCA|TLR4</td><td>4.30E-04</td><td>GO.0043030</td></tr>
<tr><td>14</td><td>GO Process</td><td>negative regulation of transport</td><td>THBS1|ICAM1|NOS3|STXBP6|SNCA|CAV1|MAP4K4|FN1|GSTO1|ANXA1|TBC1D4|PPP3CA|BCL2|NR1H4</td><td>4.30E-04</td><td>GO.0051051</td></tr>
<tr><td>7</td><td>GO Process</td><td>response to amino acid</td><td>MMP2|PDGFRA|ICAM1|CAPN2|BCL2L1|FYN|CFL1</td><td>4.60E-04</td><td>GO.0043200</td></tr>
<tr><td>18</td><td>GO Process</td><td>regulation of MAPK cascade</td><td>ARHGEF5|MAP2K5|CCL2|PDGFRA|THBS1|ICAM1|FLT1|APP|CSF1R|KIT|CAV1|MAP4K4|FN1|FAS|EPHA2|PTPN1|TLR4|PBK</td><td>4.70E-04</td><td>GO.0043408</td></tr>
<tr><td>14</td><td>GO Process</td><td>muscle structure development</td><td>MYH9|LAMA5|PDGFRA|RORA|CAPN2|CALR|FLNC|RARB|CAV1|MYH10|DLL1|SDC1|PPP3CA|FLNB</td><td>4.90E-04</td><td>GO.0061061</td></tr>
<tr><td>13</td><td>GO Process</td><td>cell morphogenesis involved in neuron differentiation</td><td>APP|CSF1R|BRSK1|DOK4|FYN|MYH10|SPTAN1|BCL2|CAMK2A|CDC42|TOP2B|SPTBN2|NUMB</td><td>5.20E-04</td><td>GO.0048667</td></tr>
<tr><td>6</td><td>GO Process</td><td>endothelial cell differentiation</td><td>LAMA5|ICAM1|TJP1|ARHGEF26|DLL1|CLIC4</td><td>5.30E-04</td><td>GO.0045446</td></tr>
<tr><td>8</td><td>GO Process</td><td>ameboidal-type cell migration</td><td>MYH9|LAMA5|KIT|NOS3|FN1|DPP4|MMRN2|CFL1</td><td>5.40E-04</td><td>GO.0001667</td></tr>
<tr><td>9</td><td>GO Process</td><td>regulation of protein polymerization</td><td>TUBB4A|ADD1|ICAM1|SNCA|MYO1C|SPTAN1|DYRK1A|SPTBN2|ARF1</td><td>5.70E-04</td><td>GO.0032271</td></tr>
<tr><td>12</td><td>GO Process</td><td>positive regulation of proteolysis</td><td>MYH9|ENO1|APP|PLK1|MMP14|DAB2|LCK|SNCA|CAV1|FYN|FN1|FAS</td><td>5.70E-04</td><td>GO.0045862</td></tr>
<tr><td>9</td><td>GO Process</td><td>protein dephosphorylation</td><td>PPP3R1|CDC25B|CDC25A|TENC1|CDC25C|LCK|PTPN1|PPP3CA|BCL2</td><td>6.10E-04</td><td>GO.0006470</td></tr>
<tr><td>18</td><td>GO Process</td><td>membrane organization</td><td>MYH9|THBS1|CCR5|PLK1|BCL2L1|SDPR|DAB2|CALR|SNCA|CAV1|ANXA2|MYH10|DNM1|BCL2|CAMK2A|CDC42|SUN1|ABCB1</td><td>6.10E-04</td><td>GO.0061024</td></tr>
<tr><td>17</td><td>GO Process</td><td>regulation of establishment of protein localization</td><td>ARHGEF5|GAPDH|APP|CSF1R|CCT2|MAP4K4|FYN|FN1|MYO1C|MYH10|DPP4|PTPN1|TLR4|ANXA1|PPP3CA|BCL2|NR1H4</td><td>6.20E-04</td><td>GO.0070201</td></tr>
<tr><td>23</td><td>GO Process</td><td>negative regulation of multicellular organismal process</td><td>MAP2K5|PDGFRA|THBS1|NGEF|APP|ANXA5|NOS3|CALR|RARB|MAP4K4|ANXA2|FN1|ARHGEF15|DLL1|MMRN2|TLR4|ANXA1|G6PD|PPP3CA|BCL2|RARG|VIM|NR1H4</td><td>6.50E-04</td><td>GO.0051241</td></tr>
<tr><td>11</td><td>GO Process</td><td>epithelial tube morphogenesis</td><td>PPP3R1|LAMA5|CSF1R|MMP14|EPHA2|DLL1|SDC4|CLIC4|BCL2|RARG|CFL1</td><td>6.70E-04</td><td>GO.0060562</td></tr>
<tr><td>11</td><td>GO Process</td><td>urogenital system development</td><td>ITGB4|LAMA5|PDGFRA|TENC1|RARB|DLL1|SDC4|ANXA1|SDC1|BCL2|RARG</td><td>6.90E-04</td><td>GO.0001655</td></tr>
<tr><td>18</td><td>GO Process</td><td>regulation of proteolysis</td><td>MAP2K5|MYH9|GAPDH|ENO1|THBS1|DNAJB6|PRKCG|APP|PLK1|MMP14|DAB2|LCK|SNCA|CAV1|FYN|FN1|FAS|PBK</td><td>7.50E-04</td><td>GO.0030162</td></tr>
<tr><td>21</td><td>GO Process</td><td>secretion by cell</td><td>PLEK|THBS1|VWF|CNN2|EXOC6B|FERMT3|APP|KIT|ANXA5|CCT2|BRSK1|TUBB4B|ANXA2|FN1|MYH10|FLNA|SPTAN1|TLR4|ANXA1|XRCC5|NR1H4</td><td>7.60E-04</td><td>GO.0032940</td></tr>
<tr><td>12</td><td>GO Process</td><td>regulation of metal ion transport</td><td>CCL2|ICAM1|NOS3|SNCA|CAV1|FYN|GSTO1|FLNA|G6PD|CAMK2B|BCL2|CAMK2A</td><td>7.80E-04</td><td>GO.0010959</td></tr>
<tr><td>17</td><td>GO Process</td><td>regulation of defense response</td><td>MMP2|RORA|APP|ITGA2|ZYX|SNCA|CAV1|CDH5|FYN|SFPQ|TGM2|PTPN1|TLR4|ANXA1|XRCC5|PBK|NR1H4</td><td>7.80E-04</td><td>GO.0031347</td></tr>
<tr><td>6</td><td>GO Process</td><td>regulation of reactive oxygen species biosynthetic process</td><td>ICAM1|APP|SNCA|CAV1|FYN|TLR4</td><td>7.80E-04</td><td>GO.1903426</td></tr>
<tr><td>15</td><td>GO Process</td><td>embryonic morphogenesis</td><td>ITGB4|MMP2|LAMA5|PDGFRA|COL8A1|FLT1|ITGA2|MMP14|JAG2|RARB|FN1|DLL1|SDC4|RARG|CFL1</td><td>8.00E-04</td><td>GO.0048598</td></tr>
<tr><td>8</td><td>GO Process</td><td>interaction with host</td><td>GAPDH|ICAM1|CCR5|ITGA2|BCL2L1|CAV1|EPHA2|DPP4</td><td>8.00E-04</td><td>GO.0051701</td></tr>
<tr><td>9</td><td>GO Process</td><td>positive regulation of cytoskeleton organization</td><td>ARHGEF5|PLEK|ICAM1|MYO1C|ARHGEF15|FLNA|SDC4|CDC42|CFL1</td><td>8.30E-04</td><td>GO.0051495</td></tr>
<tr><td>6</td><td>GO Process</td><td>ephrin receptor signaling pathway</td><td>MMP2|NGEF|FYN|EPHA2|DNM1|CDC42</td><td>8.70E-04</td><td>GO.0048013</td></tr>
<tr><td>13</td><td>GO Process</td><td>response to radiation</td><td>ICAM1|APP|KIT|DDB1|BCL2L1|CDC25A|BRSK1|CYP2R1|USP1|ANXA1|XRCC5|BCL2|PBK</td><td>8.80E-04</td><td>GO.0009314</td></tr>
<tr><td>17</td><td>GO Process</td><td>regulation of cell cycle process</td><td>CCL2|CDC25B|TUBB4A|APP|PLK1|TUBA1A|DDB1|BCL2L1|CDC25A|BRSK1|CALR|CDC25C|TUBB4B|SFPQ|ANXA1|BCL2|CDC42</td><td>8.80E-04</td><td>GO.0010564</td></tr>
<tr><td>5</td><td>GO Process</td><td>positive regulation of phospholipid metabolic process</td><td>PDGFRA|FLT1|APP|KIT|NR1H4</td><td>8.80E-04</td><td>GO.1903727</td></tr>
<tr><td>6</td><td>GO Process</td><td>peptidyl-threonine phosphorylation</td><td>CAD|CAMK2B|BCL2|CAMK2A|DYRK1A|CAMK1D</td><td>9.10E-04</td><td>GO.0018107</td></tr>
<tr><td>4</td><td>GO Process</td><td>cerebellar Purkinje cell layer development</td><td>RORA|MYH10|DLL1|SPTBN2</td><td>9.10E-04</td><td>GO.0021680</td></tr>
</table>

<table>
<tr><td>16</td><td>GO Process</td><td>regulation of ion transport</td><td>CCL2|THBS1|ICAM1|APP|NOS3|SNCA|CAV1|FYN|GSTO1|FLNA|CLIC4|G6PD|CAMK2B|BCL2|CAMK2A|ABCB1</td><td>9.10E-04</td><td>GO.0043269</td></tr>
<tr><td>4</td><td>GO Process</td><td>regulation of long-term neuronal synaptic plasticity</td><td>APP|KIT|SNCA|CAMK2B</td><td>9.10E-04</td><td>GO.0048169</td></tr>
<tr><td>8</td><td>GO Process</td><td>regulation of actin polymerization or depolymerization</td><td>PLEK|ADD1|ICAM1|MYO1C|SPTAN1|SPTBN2|CFL1|ARF1</td><td>9.20E-04</td><td>GO.0008064</td></tr>
<tr><td>5</td><td>GO Process</td><td>ovarian follicle development</td><td>ICAM1|KIT|BCL2L1|MMP14|BCL2</td><td>9.40E-04</td><td>GO.0001541</td></tr>
<tr><td>5</td><td>GO Process</td><td>regulation of actin filament depolymerization</td><td>PLEK|ADD1|SPTAN1|SPTBN2|CFL1</td><td>9.40E-04</td><td>GO.0030834</td></tr>
<tr><td>3</td><td>GO Process</td><td>negative regulation of glial cell apoptotic process</td><td>CCL2|PRKCH|DLL1</td><td>9.80E-04</td><td>GO.0034351</td></tr>
<tr><td>8</td><td>GO Process</td><td>transcription initiation from RNA polymerase II promoter</td><td>RORA|RORC|RARB|NR1I2|RXRG|RXRB|RARG|NR1H4</td><td>9.90E-04</td><td>GO.0006367</td></tr>
<tr><td>8</td><td>GO Process</td><td>cell-substrate adhesion</td><td>ITGB4|LAMA5|VWF|FERMT3|ITGA2|ZYX|FN1|BCL2</td><td>9.90E-04</td><td>GO.0031589</td></tr>
<tr><td>**8**</td><td>**GO Process**</td><td>**positive regulation of angiogenesis**</td><td>**HSPB1|THBS1|AKT3|FLT1|NOS3|CDH5|DLL1|ANXA1**</td><td>**9.90E-04**</td><td>**GO.0045766**</td></tr>
<tr><td>8</td><td>GO Process</td><td>establishment or maintenance of cell polarity</td><td>MYH9|ARF4|BRSK1|CLIC4|FSCN1|CDC42|WEE1|CFL1</td><td>0.001</td><td>GO.0007163</td></tr>
<tr><td>10</td><td>GO Process</td><td>response to xenobiotic stimulus</td><td>RORA|PRKCG|CAD|ICAM1|NQO1|RORC|NR1I2|SNCA|GSTO1|PPP3CA</td><td>0.001</td><td>GO.0009410</td></tr>
<tr><td>9</td><td>GO Process</td><td>response to mechanical stimulus</td><td>THBS1|CNN2|KIT|ITGA2|MMP14|FYN|FAS|TLR4|SUN1</td><td>0.001</td><td>GO.0009612</td></tr>
<tr><td>7</td><td>GO Process</td><td>platelet activation</td><td>PDGFRA|VWF|PRKCG|PRKCH|LCK|FYN|FLNA</td><td>0.001</td><td>GO.0030168</td></tr>
<tr><td>8</td><td>GO Process</td><td>regulation of protein localization to membrane</td><td>BCL2L1|DAB2|PRKCH|FYN|EPHA2|MYO1C|BCL2|NUMB</td><td>0.001</td><td>GO.1905475</td></tr>
<tr><td>9</td><td>GO Process</td><td>DNA-templated transcription, initiation</td><td>RORA|RORC|RARB|NR1I2|PTRF|RXRG|RXRB|RARG|NR1H4</td><td>0.0011</td><td>GO.0006352</td></tr>
<tr><td>8</td><td>GO Process</td><td>viral life cycle</td><td>CCL2|ICAM1|CCR5|ITGA2|CAV1|EPHA2|DPP4|CDC42</td><td>0.0011</td><td>GO.0019058</td></tr>
<tr><td>7</td><td>GO Process</td><td>negative regulation of protein complex assembly</td><td>TUBB4A|ADD1|SNCA|SPTAN1|DYRK1A|CDC42|SPTBN2</td><td>0.0011</td><td>GO.0031333</td></tr>
<tr><td>5</td><td>GO Process</td><td>regulation of neuronal synaptic plasticity</td><td>APP|KIT|SNCA|CAMK2B|CAMK2A</td><td>0.0011</td><td>GO.0048168</td></tr>
<tr><td>5</td><td>GO Process</td><td>T cell costimulation</td><td>LCK|CAV1|FYN|DPP4|CDC42</td><td>0.0012</td><td>GO.0031295</td></tr>
<tr><td>98</td><td>GO Process</td><td>primary metabolic process</td><td>RCN1|MAP2K5|PLOD1|MYH9|CYP24A1|MMP2|CCL2|GAPDH|RBP1|PPP3R1|PLEK|ENO1|CDC25B|ARF3|PDGFRA|THBS1|VWF|RORA|PRKCG|AKT3|CAD|ADD1|FLT1|PLOD2|APP|CSF1R|KIT|NPC1L1|CCR5|CAPN2|NOS3|MELK|CKB|PLK1|DDB1|CDC25A|ARF4|MMP14|BRSK1|TENC1|CALR|CDC25C|RORC|PRKCH|RARB|CYP2R1|NR1I2|LCK|SNCA|CAV1|RGS12|PTGES|USP1|MAP4K4|PTMA|ANXA2|FYN|FN1|PTRF|SFPQ|EPHA2|RXRG|DPP4|TGM2|GSTO1|SLK|FLNA|PTPN1|SDC4|SPTAN1|TLR4|RXRB|ANXA1|PIR|PSIP1|SDC1|XRCC5|G6PD|PPP3CA|CAMK2B|LDHB|BCL2|CAMK2A|DYRK1A|PAICS|CDC42|PDIA6|RARG|TOP2B|WEE1|PBK|SPTBN2|CFL1|ARF1|NR1H4|PRKCSH|CAMK1D|MCM3</td><td>0.0012</td><td>GO.0044238</td></tr>
<tr><td>21</td><td>GO Process</td><td>chemical homeostasis</td><td>RBP1|MT2A|PDGFRA|RORA|ICAM1|APP|CCR5|CKB|DDB1|CALR|LCK|SNCA|CAV1|ANXA6|TGM2|GSTO1|CLIC4|PPP3CA|BCL2|ARF1|NR1H4</td><td>0.0012</td><td>GO.0048878</td></tr>
<tr><td>31</td><td>GO Process</td><td>positive regulation of gene expression</td><td>MAP2K5|MYH9|PPP3R1|ENO1|THBS1|RORA|APP|KIT|ITGA2|NOS3|ARF4|MMP14|DAB2|CALR|RORC|RARB|NR1I2|CAV1|FN1|SFPQ|MYO1C|RXRG|DLL1|TLR4|RXRB|PSIP1|PPP3CA|DYRK1A|RARG|VIM|NR1H4</td><td>0.0013</td><td>GO.0010628</td></tr>
<tr><td>77</td><td>GO Process</td><td>cellular macromolecule metabolic process</td><td>RCN1|MAP2K5|PLOD1|MMP2|CCL2|GAPDH|PPP3R1|ENO1|CDC25B|PDGFRA|THBS1|RORA|PRKCG|AKT3|CAD|ADD1|FLT1|PLOD2|APP|CSF1R|KIT|CCR5|CAPN2|MELK|PLK1|DDB1|CDC25A|ARF4|BRSK1|TENC1|CDC25C|RORC|PRKCH|RARB|NR1I2|LCK|SNCA|CAV1|RGS12|USP1|MAP4K4|PTMA|FYN|FN1|PTRF|SFPQ|EPHA2|RXRG|TGM2|SLK|FLNA|PTPN1|SPTAN1|DNM1|TLR4|RXRB|ANXA1|PIR|PSIP1|XRCC5|PPP3CA|CAMK2B|BCL2|CAMK2A|DYRK1A|CDC42|PDIA6|RARG|TOP2B|WEE1|PBK|SPTBN2|CFL1|NR1H4|PRKCSH|CAMK1D|MCM3</td><td>0.0013</td><td>GO.0044260</td></tr>
<tr><td>4</td><td>GO Process</td><td>positive regulation of cell cycle G2/M phase transition</td><td>CDC25B|APP|CDC25A|CDC25C</td><td>0.0013</td><td>GO.1902751</td></tr>
<tr><td>7</td><td>GO Process</td><td>positive regulation of leukocyte migration</td><td>THBS1|ICAM1|APP|ITGA2|MMP14|CALR|CAMK1D</td><td>0.0014</td><td>GO.0002687</td></tr>
<tr><td>19</td><td>GO Process</td><td>central nervous system development</td><td>RORA|AKT3|APP|CSF1R|CKB|ARF4|RARB|FYN|MYH10|DLL1|FLNA|XRCC5|PPP3CA|BCL2|SUN1|TOP2B|SPTBN2|VIM|NUMB</td><td>0.0014</td><td>GO.0007417</td></tr>
<tr><td>14</td><td>GO Process</td><td>sensory organ development</td><td>PDGFRA|COL8A1|KIT|JAG2|RARB|EPHA2|MYH10|ARHGEF15|DLL1|SDC4|CLIC4|BCL2|RARG|VIM</td><td>0.0014</td><td>GO.0007423</td></tr>
<tr><td>15</td><td>GO Process</td><td>cellular ion homeostasis</td><td>MT2A|PDGFRA|APP|CCR5|CKB|CALR|LCK|SNCA|CAV1|ANXA6|TGM2|GSTO1|CLIC4|BCL2|ARF1</td><td>0.0015</td><td>GO.0006873</td></tr>
<tr><td>11</td><td>GO Process</td><td>negative regulation of organelle organization</td><td>TUBB4A|ADD1|APP|PLK1|BCL2L1|SNCA|SPTAN1|TBC1D4|XRCC5|DYRK1A|SPTBN2</td><td>0.0015</td><td>GO.0010639</td></tr>
<tr><td>20</td><td>GO Process</td><td>neuron differentiation</td><td>RORA|APP|CSF1R|ARF4|BRSK1|JAG2|MAP4K4|DOK4|FYN|MYH10|DLL1|SDC4|SPTAN1|BCL2|CAMK2A|CDC42|TOP2B|WEE1|SPTBN2|NUMB</td><td>0.0015</td><td>GO.0030182</td></tr>
<tr><td>11</td><td>GO Process</td><td>regulation of cellular component size</td><td>PLEK|AKT3|ADD1|ICAM1|FN1|MYO1C|SPTAN1|RARG|SPTBN2|CFL1|ARF1</td><td>0.0015</td><td>GO.0032535</td></tr>
<tr><td>86</td><td>GO Process</td><td>macromolecule metabolic process</td><td>RCN1|MAP2K5|PLOD1|MYH9|MMP2|CCL2|GAPDH|PPP3R1|ENO1|CDC25B|PDGFRA|THBS1|VWF|RORA|PRKCG|AKT3|CAD|ADD1|FLT1|PLOD2|APP|CSF1R|KIT|NPC1L1|CCR5|CAPN2|MELK|PLK1|DDB1|CDC25A|ARF4|MMP14|BRSK1|TENC1|CALR|CDC25C|RORC|PRKCH|RARB|NR1I2|LCK|SNCA|CAV1|RGS12|USP1|MAP4K4|PTMA|ANXA2|FYN|FN1|PTRF|SFPQ|EPHA2|RXRG|DPP4|TGM2|SLK|FLNA|PTPN1|SDC4|SPTAN1|DNM1|TLR4|RXRB|ANXA1|PIR|PSIP1|SDC1|XRCC5|PPP3CA|CAMK2B|BCL2|CAMK2A|DYRK1A|CDC42|PDIA6|RARG|TOP2B|WEE1|PBK|SPTBN2|CFL1|NR1H4|PRKCSH|CAMK1D|MCM3</td><td>0.0015</td><td>GO.0043170</td></tr>
<tr><td>5</td><td>GO Process</td><td>positive regulation of blood vessel endothelial cell migration</td><td>HSPB1|THBS1|AKT3|NOS3|ANXA1</td><td>0.0015</td><td>GO.0043536</td></tr>
<tr><td>7</td><td>GO Process</td><td>response to calcium ion</td><td>THBS1|ADD1|CAV1|PTGES|CLIC4|SDC1|PPP3CA</td><td>0.0015</td><td>GO.0051592</td></tr>
<tr><td>11</td><td>GO Process</td><td>regulation of small molecule metabolic process</td><td>PLEK|ENO1|RORA|KIT|NOS3|NQO1|RORC|SNCA|CAV1|ANXA1|NR1H4</td><td>0.0015</td><td>GO.0062012</td></tr>
<tr><td>5</td><td>GO Process</td><td>positive regulation of intrinsic apoptotic signaling pathway</td><td>BCL2L1|LCK|CAV1|SFPQ|BCL2</td><td>0.0015</td><td>GO.2001244</td></tr>
<tr><td>24</td><td>GO Process</td><td>cell cycle</td><td>MYH9|CDC25B|THBS1|TUBB4A|MELK|PLK1|TUBA1A|BCL2L1|CDC25A|BRSK1|CDC25C|TUBB4B|MYH10|FLNA|PPP3CA|CAMK2A|SEPT7|SUN1|TOP2B|WEE1|PBK|CFL1|ABCB1|MCM3</td><td>0.0016</td><td>GO.0007049</td></tr>
<tr><td>7</td><td>GO Process</td><td>T cell differentiation</td><td>RORA|KIT|RORC|JAG2|LCK|ANXA1|BCL2</td><td>0.0016</td><td>GO.0030217</td></tr>
<tr><td>19</td><td>GO Process</td><td>regulation of immune response</td><td>MMP2|PPP3R1|ICAM1|APP|KIT|PRKCH|LCK|CAV1|FYN|SFPQ|MYO1C|PTPN1|TLR4|ANXA1|XRCC5|PPP3CA|BCL2|CDC42|NR1H4</td><td>0.0016</td><td>GO.0050776</td></tr>
<tr><td>5</td><td>GO Process</td><td>regulation of extrinsic apoptotic signaling pathway via death domain receptors</td><td>THBS1|ICAM1|NOS3|BCL2L1|FAS</td><td>0.0016</td><td>GO.1902041</td></tr>
<tr><td>4</td><td>GO Process</td><td>response to amyloid-beta</td><td>APP|FYN|DNM1|TLR4</td><td>0.0016</td><td>GO.1904645</td></tr>
<tr><td>6</td><td>GO Process</td><td>viral entry into host cell</td><td>ICAM1|CCR5|ITGA2|CAV1|EPHA2|DPP4</td><td>0.0017</td><td>GO.0046718</td></tr>
<tr><td>11</td><td>GO Process</td><td>developmental growth</td><td>ADD1|APP|TENC1|RARB|DLL1|CLIC4|ANXA1|BCL2|RARG|SPTBN2|APBA2</td><td>0.0017</td><td>GO.0048589</td></tr>
<tr><td>11</td><td>GO Process</td><td>regulation of inflammatory response</td><td>RORA|APP|ITGA2|ZYX|SNCA|CDH5|TGM2|TLR4|ANXA1|PBK|NR1H4</td><td>0.0017</td><td>GO.0050727</td></tr>
<tr><td>11</td><td>GO Process</td><td>positive regulation of protein serine/threonine kinase activity</td><td>ARHGEF5|MAP2K5|THBS1|FLT1|APP|CSF1R|KIT|SNCA|MAP4K4|PTPN1|TLR4</td><td>0.0017</td><td>GO.0071902</td></tr>
<tr><td>15</td><td>GO Process</td><td>regulation of neuron differentiation</td><td>NGEF|APP|BRSK1|CALR|RARB|MAP4K4|FYN|FN1|DLL1|PPP3CA|CAMK2B|BCL2|CFL1|VIM|CAMK1D</td><td>0.0018</td><td>GO.0045664</td></tr>
<tr><td>6</td><td>GO Process</td><td>tissue remodeling</td><td>NOS3|MMP14|CAV1|EPHA2|TGM2|ANXA1</td><td>0.0018</td><td>GO.0048771</td></tr>
<tr><td>12</td><td>GO Process</td><td>regulation of DNA-binding transcription factor activity</td><td>ARHGEF5|MAP2K5|ICAM1|APP|KIT|PRKCH|FLNA|TLR4|PPP3CA|CAMK2A|NR1H4|CAMK1D</td><td>0.0018</td><td>GO.0051090</td></tr>
<tr><td>13</td><td>GO Process</td><td>regulation of anatomical structure size</td><td>PLEK|AKT3|ADD1|ICAM1|NOS3|CAV1|FN1|MYO1C|SPTAN1|RARG|SPTBN2|CFL1|ARF1</td><td>0.0018</td><td>GO.0090066</td></tr>
<tr><td>11</td><td>GO Process</td><td>axonogenesis</td><td>APP|CSF1R|BRSK1|DOK4|FYN|MYH10|SPTAN1|BCL2|TOP2B|SPTBN2|NUMB</td><td>0.0019</td><td>GO.0007409</td></tr>
<tr><td>10</td><td>GO Process</td><td>blood coagulation</td><td>PDGFRA|VWF|PRKCG|ANXA5|ITGA2|PRKCH|LCK|FYN|FLNA|CDC42</td><td>0.0019</td><td>GO.0007596</td></tr>
<tr><td>4</td><td>GO Process</td><td>regulation of platelet activation</td><td>PLEK|PDGFRA|NOS3|TLR4</td><td>0.0019</td><td>GO.0010543</td></tr>
</table>

<table>
<tr><td>7</td><td>GO Process</td><td>positive regulation of epithelial cell migration</td><td>HSPB1|THBS1|AKT3|ITGA2|NOS3|CALR|ANXA1</td><td>0.0019</td><td>GO.0010634</td></tr>
<tr><td>3</td><td>GO Process</td><td>positive regulation of tumor necrosis factor biosynthetic process</td><td>HSPB1|THBS1|TLR4</td><td>0.0019</td><td>GO.0042535</td></tr>
<tr><td>22</td><td>GO Process</td><td>negative regulation of molecular function</td><td>MAP2K5|GAPDH|HSPB1|PXDN|THBS1|DNAJB6|APP|ANXA5|NOS3|PLK1|DAB2|NQO1|SNCA|CAV1|ANXA2|GSTO1|FLNA|CNN3|PTPN1|ANXA1|PPP3CA|NR1H4</td><td>0.0019</td><td>GO.0044092</td></tr>
<tr><td>12</td><td>GO Process</td><td>reproductive structure development</td><td>BCL2L2|PDGFRA|ICAM1|KIT|NOS3|BCL2L1|MMP14|FLNA|ANXA1|SDC1|BCL2|RARG</td><td>0.0019</td><td>GO.0048608</td></tr>
<tr><td>7</td><td>GO Process</td><td>retina development in camera-type eye</td><td>PDGFRA|RARB|MYH10|ARHGEF15|DLL1|CLIC4|RARG</td><td>0.0019</td><td>GO.0060041</td></tr>
<tr><td>8</td><td>GO Process</td><td>cell chemotaxis</td><td>ARHGEF5|CCL2|PDGFRA|FLT1|KIT|CCR5|EPHA2|ANXA1</td><td>0.0019</td><td>GO.0060326</td></tr>
<tr><td>5</td><td>GO Process</td><td>regulation of postsynapse organization</td><td>NGEF|APP|FYN|CAMK2B|CFL1</td><td>0.0019</td><td>GO.0099175</td></tr>
<tr><td>10</td><td>GO Process</td><td>regulation of neuron death</td><td>CCL2|PRKCG|APP|BCL2L1|NQO1|SNCA|FYN|TLR4|G6PD|BCL2</td><td>0.0019</td><td>GO.1901214</td></tr>
<tr><td>5</td><td>GO Process</td><td>regulation of adherens junction organization</td><td>THBS1|ADD1|MMP14|SLK|SDC4</td><td>0.0019</td><td>GO.1903391</td></tr>
<tr><td>8</td><td>GO Process</td><td>phagocytosis</td><td>MYH9|THBS1|FYN|MYO1C|TGM2|ANXA1|CDC42|PDIA6</td><td>0.002</td><td>GO.0006909</td></tr>
<tr><td>6</td><td>GO Process</td><td>positive regulation of endothelial cell migration</td><td>HSPB1|THBS1|AKT3|NOS3|CALR|ANXA1</td><td>0.002</td><td>GO.0010595</td></tr>
<tr><td>5</td><td>GO Process</td><td>cellular response to amino acid stimulus</td><td>MMP2|PDGFRA|CAPN2|BCL2L1|FYN</td><td>0.002</td><td>GO.0071230</td></tr>
<tr><td>6</td><td>GO Process</td><td>negative regulation of intrinsic apoptotic signaling pathway</td><td>ENO1|HSPB1|BCL2L2|BCL2L1|PTPN1|BCL2</td><td>0.002</td><td>GO.2001243</td></tr>
<tr><td>6</td><td>GO Process</td><td>actomyosin structure organization</td><td>MYH9|PDGFRA|CNN2|ZYX|MYH10|CNN3</td><td>0.0021</td><td>GO.0031032</td></tr>
<tr><td>10</td><td>GO Process</td><td>camera-type eye development</td><td>PDGFRA|COL8A1|RARB|EPHA2|MYH10|ARHGEF15|DLL1|CLIC4|RARG|VIM</td><td>0.0021</td><td>GO.0043010</td></tr>
<tr><td>22</td><td>GO Process</td><td>regulation of cell cycle</td><td>MAP2K5|CCL2|CDC25B|THBS1|TUBB4A|APP|PLK1|TUBA1A|DDB1|BCL2L1|CDC25A|BRSK1|CALR|CDC25C|TUBB4B|MAP4K4|SFPQ|SLK|ANXA1|BCL2|CDC42|TOP2B</td><td>0.0021</td><td>GO.0051726</td></tr>
<tr><td>11</td><td>GO Process</td><td>regulation of mitotic cell cycle phase transition</td><td>CCL2|TUBB4A|APP|PLK1|TUBA1A|DDB1|BRSK1|CDC25C|TUBB4B|ANXA1|BCL2</td><td>0.0021</td><td>GO.1901990</td></tr>
<tr><td>10</td><td>GO Process</td><td>regulation of neurotransmitter levels</td><td>RORA|ICAM1|APP|NOS3|BRSK1|NQO1|SNCA|CAV1|TLR4|CAMK2A</td><td>0.0022</td><td>GO.0001505</td></tr>
<tr><td>3</td><td>GO Process</td><td>negative regulation of microtubule polymerization</td><td>TUBB4A|SNCA|DYRK1A</td><td>0.0022</td><td>GO.0031115</td></tr>
<tr><td>8</td><td>GO Process</td><td>regulation of chemotaxis</td><td>CCL2|HSPB1|PDGFRA|THBS1|APP|ITGA2|CALR|CAMK1D</td><td>0.0022</td><td>GO.0050920</td></tr>
<tr><td>4</td><td>GO Process</td><td>positive regulation of phosphatidylinositol 3-kinase activity</td><td>PDGFRA|FLT1|APP|KIT</td><td>0.0023</td><td>GO.0043552</td></tr>
<tr><td>4</td><td>GO Process</td><td>homeostasis of number of cells within a tissue</td><td>AKT3|ADD1|NOS3|BCL2</td><td>0.0023</td><td>GO.0048873</td></tr>
<tr><td>14</td><td>GO Process</td><td>chordate embryonic development</td><td>MYH9|PDGFRA|ADD1|CAPN2|NOS3|BCL2L1|MMP14|JAG2|MYH10|DLL1|SDC4|RARG|CFL1|APBA2</td><td>0.0024</td><td>GO.0043009</td></tr>
<tr><td>7</td><td>GO Process</td><td>negative regulation of ion transport</td><td>THBS1|ICAM1|NOS3|SNCA|CAV1|GSTO1|BCL2</td><td>0.0024</td><td>GO.0043271</td></tr>
<tr><td>19</td><td>GO Process</td><td>positive regulation of cell differentiation</td><td>ADD1|APP|KIT|MMP14|DAB2|CALR|PRKCH|RARB|CDH5|FYN|FN1|FLNA|ANXA1|XRCC5|CAMK2B|BCL2|CDC42|NUMB|CAMK1D</td><td>0.0024</td><td>GO.0045597</td></tr>
<tr><td>13</td><td>GO Process</td><td>cell division</td><td>MYH9|CDC25B|KIT|PLK1|TUBA1A|TUBA1C|CDC25A|CDC25C|MYH10|SEPT7|WEE1|CFL1|NUMB</td><td>0.0024</td><td>GO.0051301</td></tr>
<tr><td>18</td><td>GO Process</td><td>response to other organism</td><td>CCL2|GAPDH|ENO1|HSPB1|ICAM1|APP|CCR5|NOS3|BCL2L1|SNCA|CAV1|PTGES|FAS|TLR4|BCL2|CFL1|ARF1|NR1H4</td><td>0.0024</td><td>GO.0051707</td></tr>
<tr><td>7</td><td>GO Process</td><td>hindbrain development</td><td>RORA|CKB|MYH10|DLL1|FLNA|BCL2|SPTBN2</td><td>0.0025</td><td>GO.0030902</td></tr>
<tr><td>3</td><td>GO Process</td><td>nitric oxide biosynthetic process</td><td>RORA|NOS3|NQO1</td><td>0.0026</td><td>GO.0006809</td></tr>
<tr><td>3</td><td>GO Process</td><td>cerebellar Purkinje cell layer morphogenesis</td><td>RORA|DLL1|SPTBN2</td><td>0.0026</td><td>GO.0021692</td></tr>
<tr><td>6</td><td>GO Process</td><td>establishment of cell polarity</td><td>MYH9|BRSK1|FSCN1|CDC42|WEE1|CFL1</td><td>0.0026</td><td>GO.0030010</td></tr>
<tr><td>15</td><td>GO Process</td><td>regulation of protein transport</td><td>ARHGEF5|GAPDH|APP|CSF1R|MAP4K4|FYN|FN1|MYO1C|MYH10|DPP4|PTPN1|TLR4|ANXA1|PPP3CA|NR1H4</td><td>0.0026</td><td>GO.0051223</td></tr>
<tr><td>5</td><td>GO Process</td><td>regulation of oxidative stress-induced cell death</td><td>HSPB1|APP|FYN|SFPQ|TLR4</td><td>0.0026</td><td>GO.1903201</td></tr>
<tr><td>8</td><td>GO Process</td><td>regulation of neuron apoptotic process</td><td>CCL2|PRKCG|BCL2L1|NQO1|SNCA|FYN|G6PD|BCL2</td><td>0.0027</td><td>GO.0043523</td></tr>
<tr><td>12</td><td>GO Process</td><td>positive regulation of neurogenesis</td><td>APP|KIT|PRKCH|RARB|FYN|FN1|FLNA|XRCC5|CAMK2B|BCL2|NUMB|CAMK1D</td><td>0.0027</td><td>GO.0050769</td></tr>
<tr><td>17</td><td>GO Process</td><td>exocytosis</td><td>PLEK|THBS1|VWF|CNN2|EXOC6B|FERMT3|APP|KIT|ANXA5|CCT2|TUBB4B|ANXA2|FN1|MYH10|FLNA|SPTAN1|XRCC5</td><td>0.0028</td><td>GO.0006887</td></tr>
<tr><td>6</td><td>GO Process</td><td>metencephalon development</td><td>RORA|CKB|MYH10|DLL1|BCL2|SPTBN2</td><td>0.0028</td><td>GO.0022037</td></tr>
<tr><td>11</td><td>GO Process</td><td>regulation of cell-cell adhesion</td><td>MAP2K5|CCL2|ICAM1|FERMT3|LCK|CAV1|FYN|DPP4|SDC4|ANXA1|CDC42</td><td>0.0028</td><td>GO.0022407</td></tr>
<tr><td>11</td><td>GO Process</td><td>positive regulation of defense response</td><td>MMP2|APP|ITGA2|SNCA|CAV1|FYN|SFPQ|TGM2|TLR4|XRCC5|NR1H4</td><td>0.0028</td><td>GO.0031349</td></tr>
<tr><td>8</td><td>GO Process</td><td>gliogenesis</td><td>CCL2|PPP3R1|APP|MMP14|DLL1|ANXA1|SUN1|VIM</td><td>0.0028</td><td>GO.0042063</td></tr>
<tr><td>9</td><td>GO Process</td><td>positive regulation of DNA-binding transcription factor activity</td><td>ARHGEF5|ICAM1|APP|KIT|PRKCH|TLR4|PPP3CA|CAMK2A|CAMK1D</td><td>0.0028</td><td>GO.0051091</td></tr>
<tr><td>70</td><td>GO Process</td><td>regulation of nitrogen compound metabolic process</td><td>ARHGEF5|MAP2K5|MYH9|EHD4|CCL2|GAPDH|PPP3R1|PLEK|ENO1|CDC25B|HSPB1|PDGFRA|THBS1|RORA|DNAJB6|PRKCG|ICAM1|FLT1|APP|CSF1R|KIT|ITGA2|NOS3|CCT2|PLK1|DDB1|CDC25A|ARF4|MMP14|DAB2|NQO1|CALR|CDC25C|RORC|PRKCH|RARB|NR1I2|LCK|SNCA|CAV1|USP1|MAP4K4|ANXA2|FYN|FN1|FAS|PTRF|SFPQ|EPHA2|RXRG|DLL1|SLK|FLNA|PTPN1|SDC4|TLR4|RXRB|PIR|PSIP1|XRCC5|G6PD|PPP3CA|BCL2|CAMK2A|DYRK1A|RARG|PBK|VIM|NR1H4|CAMK1D</td><td>0.0028</td><td>GO.0051171</td></tr>
<tr><td>8</td><td>GO Process</td><td>cellular response to acid chemical</td><td>MMP2|PDGFRA|CAPN2|BCL2L1|FYN|XRCC5|RARG|NR1H4</td><td>0.0028</td><td>GO.0071229</td></tr>
<tr><td>10</td><td>GO Process</td><td>cellular response to external stimulus</td><td>CYP24A1|CNN2|ICAM1|ITGA2|CAV1|FYN|FAS|TLR4|BCL2|NR1H4</td><td>0.0028</td><td>GO.0071496</td></tr>
<tr><td>99</td><td>GO Process</td><td>organic substance metabolic process</td><td>RCN1|MAP2K5|PLOD1|MYH9|CYP24A1|MMP2|CCL2|GAPDH|RBP1|PPP3R1|PLEK|ENO1|CDC25B|ARF3|PDGFRA|THBS1|VWF|RORA|PRKCG|AKT3|CAD|ADD1|FLT1|PLOD2|APP|CSF1R|KIT|NPC1L1|CCR5|CAPN2|NOS3|MELK|CKB|PLK1|DDB1|CDC25A|ARF4|MMP14|BRSK1|TENC1|CALR|CDC25C|RORC|PRKCH|RARB|CYP2R1|NR1I2|LCK|SNCA|CAV1|RGS12|PTGES|USP1|MAP4K4|PTMA|ANXA2|FYN|FN1|PTRF|SFPQ|EPHA2|RXRG|DPP4|TGM2|GSTO1|SLK|FLNA|PTPN1|SDC4|SPTAN1|DNM1|TLR4|RXRB|ANXA1|PIR|PSIP1|SDC1|XRCC5|G6PD|PPP3CA|CAMK2B|LDHB|BCL2|CAMK2A|DYRK1A|PAICS|CDC42|PDIA6|RARG|TOP2B|WEE1|PBK|SPTBN2|CFL1|ARF1|NR1H4|PRKCSH|CAMK1D|MCM3</td><td>0.0028</td><td>GO.0071704</td></tr>
<tr><td>5</td><td>GO Process</td><td>multicellular organismal response to stress</td><td>THBS1|PRKCG|DPP4|PPP3CA|BCL2</td><td>0.0029</td><td>GO.0033555</td></tr>
<tr><td>11</td><td>GO Process</td><td>regulation of protein catabolic process</td><td>PRKCG|APP|PLK1|DDB1|DAB2|SNCA|CAV1|ANXA2|FYN|FLNA|PBK</td><td>0.0029</td><td>GO.0042176</td></tr>
<tr><td>4</td><td>GO Process</td><td>neuron projection organization</td><td>APP|FYN|CDC42|ARF1</td><td>0.0029</td><td>GO.0106027</td></tr>
<tr><td>4</td><td>GO Process</td><td>negative regulation of extrinsic apoptotic signaling pathway via death domain receptors</td><td>ICAM1|NOS3|BCL2L1|FAS</td><td>0.0029</td><td>GO.1902042</td></tr>
<tr><td>3</td><td>GO Process</td><td>cellular copper ion homeostasis</td><td>MT2A|APP|ARF1</td><td>0.003</td><td>GO.0006878</td></tr>
<tr><td>3</td><td>GO Process</td><td>Sertoli cell development</td><td>ICAM1|FLNA|SDC1</td><td>0.003</td><td>GO.0060009</td></tr>
<tr><td>5</td><td>GO Process</td><td>positive regulation of organelle assembly</td><td>ARHGEF5|SDC4|SDC1|FSCN1|SEPT7</td><td>0.003</td><td>GO.1902117</td></tr>
<tr><td>13</td><td>GO Process</td><td>cellular metal ion homeostasis</td><td>MT2A|PDGFRA|APP|CCR5|CALR|LCK|SNCA|CAV1|ANXA6|TGM2|GSTO1|BCL2|ARF1</td><td>0.0031</td><td>GO.0006875</td></tr>
<tr><td>7</td><td>GO Process</td><td>cytoskeleton-dependent intracellular transport</td><td>HSPB1|APP|TUBA1A|TUBA1C|MYO1C|CDC42|SUN1</td><td>0.0031</td><td>GO.0030705</td></tr>
<tr><td>16</td><td>GO Process</td><td>ion homeostasis</td><td>MT2A|PDGFRA|APP|CCR5|CKB|CALR|LCK|SNCA|CAV1|ANXA6|TGM2|GSTO1|CLIC4|BCL2|ARF1|NR1H4</td><td>0.0031</td><td>GO.0050801</td></tr>
<tr><td>4</td><td>GO Process</td><td>negative regulation of extrinsic apoptotic signaling pathway in absence of ligand</td><td>MAP2K5|BCL2L1|FYN|BCL2</td><td>0.0031</td><td>GO.2001240</td></tr>
<tr><td>7</td><td>GO Process</td><td>response to unfolded protein</td><td>CCL2|HSPB1|THBS1|ADD1|CALR|PTPN1|PDIA6</td><td>0.0032</td><td>GO.0006986</td></tr>
<tr><td>14</td><td>GO Process</td><td>cellular cation homeostasis</td><td>MT2A|PDGFRA|APP|CCR5|CALR|LCK|SNCA|CAV1|ANXA6|TGM2|GSTO1|CLIC4|BCL2|ARF1</td><td>0.0032</td><td>GO.0030003</td></tr>
<tr><td>5</td><td>GO Process</td><td>regulation of phagocytosis</td><td>CCL2|PRKCG|ITGA2|CALR|CAMK1D</td><td>0.0032</td><td>GO.0050764</td></tr>
<tr><td>72</td><td>GO Process</td><td>regulation of macromolecule metabolic process</td><td>ARHGEF5|MAP2K5|MYH9|EHD4|CCL2|GAPDH|PPP3R1|PLEK|ENO1|CDC25B|HSPB1|PDGFRA|THBS1|RORA|DNAJB6|PRKCG|ICAM1|FLT1|APP|CSF1R|KIT|ITGA2|NOS3|CCT2|PLK1|DDB1|CDC25A|ARF4|MMP14|DAB2|CALR|CDC25C|RORC|PRKCH|RARB|NR1I2|LCK|SNCA|CAV1|USP1|MAP4K4|ANXA2|FYN|FN1|FAS|PTRF|SFPQ|EPHA2|MYO1C|RXRG|DLL1|SLK|FLNA|PTPN1|SDC4|TLR4|RXRB|PIR|PSIP1|XRCC5|G6PD|PPP3CA|BCL2|CAMK2A|DYRK1A|RARG|PBK|ARF1|VIM|NR1H4|APBA2|CAMK1D</td><td>0.0032</td><td>GO.0060255</td></tr>
</table>

8	GO Process	positive regulation of leukocyte cell-cell adhesion	CCL2|ICAM1|LCK|CAV1|FYN|DPP4|ANXA1|CDC42	0.0032	GO.1903039
4	GO Process	negative regulation of endothelial cell proliferation	CCL2|THBS1|FLT1|CAV1	0.0033	GO.0001937
6	GO Process	positive regulation of cell-substrate adhesion	COL8A1|CALR|FN1|FLNA|SDC4|CDC42	0.0033	GO.0010811
4	GO Process	muscle filament sliding	TPM4|TPM3|TPM2|VIM	0.0033	GO.0030049
72	GO Process	regulation of cellular metabolic process	ARHGEF5|MAP2K5|MYH9|EHD4|CCL2|GAPDH|PPP3R1|PLEK|ENO1|CDC25B|HSPB1|PDGFRA|THBS1|RORA|DNAJB6|PRKCG|ICAM1|FLT1|APP|CSF1R|KIT|ITGA2|NOS3|CCT2|PLK1|BCL2L1|CDC25A|ARF4|MMP14|DAB2|NQO1|CALR|CDC25C|RORC|PRKCH|RARB|NR1I2|LCK|SNCA|CAV1|USP1|MAP4K4|ANXA2|FYN|FN1|FAS|PTRF|SFPQ|EPHA2|RXRG|DLL1|SLK|FLNA|PTPN1|SDC4|TLR4|RXRB|ANXA1|PIR|PSIP1|XRCC5|G6PD|PPP3CA|BCL2|CAMK2A|DYRK1A|RARG|PBK|ARF1|VIM|NR1H4|CAMK1D	0.0033	GO.0031323
15	GO Process	inorganic ion homeostasis	MT2A|PDGFRA|APP|CCR5|CKB|CALR|LCK|SNCA|CAV1|ANXA6|TGM2|GSTO1|CLIC4|BCL2|ARF1	0.0033	GO.0098771
14	GO Process	organelle localization	MYH9|PLEK|TUBB4A|EXOC6B|KIT|PLK1|TUBA1A|SNCA|TUBB4B|MYO1C|MYH10|DNM1|CDC42|SUN1	0.0034	GO.0051640
2	GO Process	cellular response to norepinephrine stimulus	APLP1|APP	0.0034	GO.0071874
2	GO Process	T cell extravasation	CCL2|ICAM1	0.0034	GO.0072683
71	GO Process	regulation of primary metabolic process	ARHGEF5|MAP2K5|MYH9|EHD4|CCL2|GAPDH|PPP3R1|PLEK|ENO1|CDC25B|HSPB1|PDGFRA|THBS1|RORA|DNAJB6|PRKCG|ICAM1|FLT1|APP|CSF1R|KIT|ITGA2|NOS3|CCT2|PLK1|DDB1|CDC25A|ARF4|MMP14|DAB2|NQO1|CALR|CDC25C|RORC|PRKCH|RARB|NR1I2|LCK|SNCA|CAV1|USP1|MAP4K4|ANXA2|FYN|FN1|FAS|PTRF|SFPQ|EPHA2|RXRG|DLL1|SLK|FLNA|PTPN1|SDC4|TLR4|RXRB|ANXA1|PIR|PSIP1|XRCC5|G6PD|PPP3CA|BCL2|CAMK2A|DYRK1A|RARG|PBK|VIM|NR1H4|CAMK1D	0.0034	GO.0080090
**3**	**GO Process**	**negative regulation of cell migration involved in sprouting angiogenesis**	**MAP2K5|THBS1|MMRN2**	**0.0034**	**GO.0090051**
7	GO Process	regulation of muscle contraction	ENO1|KIT|ITGA2|CAV1|ANXA6|GSTO1|FLNA	0.0035	GO.0006937
9	GO Process	positive regulation of lymphocyte activation	CCL2|MMP14|LCK|CAV1|FYN|DPP4|ANXA1|BCL2|CDC42	0.0035	GO.0051251
102	GO Process	metabolic process	RCN1|MAP2K5|PLOD1|ITGB4|MYH9|CYP24A1|MMP2|CCL2|GAPDH|RBP1|PPP3R1|PLEK|ENO1|CDC25B|PXDN|ARF3|PDGFRA|THBS1|VWF|RORA|PRKCG|AKT3|CAD|ADD1|FLT1|PLOD2|APP|CSF1R|KIT|NPC1L1|CCR5|CAPN2|NOS3|MELK|CKB|PLK1|DDB1|CDC25A|ARF4|MMP14|BRSK1|TENC1|NQO1|CALR|CDC25C|RORC|PRKCH|RARB|CYP2R1|NR1I2|LCK|SNCA|CAV1|RGS12|PTGES|USP1|MAP4K4|PTMA|ANXA2|FYN|FN1|PTRF|SFPQ|EPHA2|RXRG|DPP4|TGM2|GSTO1|SLK|FLNA|PTPN1|SDC4|SPTAN1|DNM1|TLR4|RXRB|ANXA1|PIR|PSIP1|SDC1|XRCC5|G6PD|PPP3CA|CAMK2B|LDHB|BCL2|CAMK2A|DYRK1A|PAICS|CDC42|PDIA6|RARG|TOP2B|WEE1|PBK|SPTBN2|CFL1|ARF1|NR1H4|PRKCSH|CAMK1D|MCM3	0.0036	GO.0008152
30	GO Process	positive regulation of biosynthetic process	MAP2K5|CCL2|PPP3R1|ENO1|HSPB1|THBS1|RORA|ICAM1|APP|ITGA2|NOS3|CCT2|ARF4|DAB2|RORC|RARB|NR1I2|SNCA|SFPQ|RXRG|DLL1|TLR4|RXRB|ANXA1|PSIP1|XRCC5|PPP3CA|RARG|VIM|NR1H4	0.0037	GO.0009891
9	GO Process	regulation of cell morphogenesis involved in differentiation	NGEF|BRSK1|CALR|FN1|FLNA|PPP3CA|CAMK2B|CDC42|CFL1	0.0037	GO.0010769
13	GO Process	positive regulation of MAPK cascade	ARHGEF5|MAP2K5|CCL2|PDGFRA|THBS1|ICAM1|FLT1|APP|CSF1R|KIT|MAP4K4|PTPN1|TLR4	0.0037	GO.0043410
9	GO Process	positive regulation of MAP kinase activity	ARHGEF5|MAP2K5|THBS1|FLT1|APP|KIT|MAP4K4|PTPN1|TLR4	0.0038	GO.0043406
13	GO Process	regulation of system process	ENO1|ICAM1|APP|KIT|ITGA2|NOS3|CAV1|ANXA6|GSTO1|FLNA|G6PD|PPP3CA|CAMK2B	0.0039	GO.0044057
4	GO Process	neuron apoptotic process	GAPDH|APP|BCL2L1|BCL2	0.0039	GO.0051402
3	GO Process	retina vasculature development in camera-type eye	PDGFRA|ARHGEF15|CLIC4	0.0039	GO.0061298
3	GO Process	positive regulation of oxidative stress-induced cell death	APP|SFPQ|TLR4	0.0039	GO.1903209
8	GO Process	phosphatidylinositol metabolic process	PLEK|ARF3|PDGFRA|CSF1R|KIT|LCK|FYN|ARF1	0.004	GO.0046488
7	GO Process	morphogenesis of a branching epithelium	PPP3R1|LAMA5|MMP14|EPHA2|TGM2|CLIC4|BCL2	0.004	GO.0061138
6	GO Process	formation of primary germ layer	ITGB4|MMP2|COL8A1|ITGA2|MMP14|FN1	0.0041	GO.0001704
11	GO Process	cellular calcium ion homeostasis	PDGFRA|APP|CCR5|CALR|LCK|SNCA|CAV1|ANXA6|TGM2|GSTO1|BCL2	0.0041	GO.0006874
5	GO Process	adherens junction organization	LAMA5|ITGA2|CDH5|BCL2|NUMB	0.0041	GO.0034332
4	GO Process	endodermal cell differentiation	MMP2|COL8A1|MMP14|FN1	0.0041	GO.0035987
7	GO Process	regulation of synapse organization	NGEF|APP|SNCA|FYN|ARHGEF15|CAMK2B|CFL1	0.0041	GO.0050807
6	GO Process	positive regulation of calcium ion transport	CCL2|SNCA|CAV1|GSTO1|G6PD|CAMK2A	0.0041	GO.0051928
9	GO Process	renal system development	ITGB4|LAMA5|PDGFRA|TENC1|RARB|DLL1|SDC4|SDC1|BCL2	0.0041	GO.0072001
6	GO Process	negative regulation of supramolecular fiber organization	TUBB4A|ADD1|SNCA|SPTAN1|DYRK1A|SPTBN2	0.0041	GO.1902904
5	GO Process	interaction with symbiont	GAPDH|DDB1|ANXA2|FN1|CFL1	0.0042	GO.0051702
7	GO Process	regulation of plasma membrane bounded cell projection assembly	ICAM1|KIT|CAV1|EPHA2|FSCN1|SEPT7|CDC42	0.0043	GO.0120032
5	GO Process	cell-cell junction assembly	TJP1|CDH5|FSCN1|NR1H4|TLN2	0.0044	GO.0007043
3	GO Process	nitric oxide metabolic process	RORA|NOS3|NQO1	0.0045	GO.0046209
7	GO Process	regulation of synaptic plasticity	APP|KIT|BRSK1|SNCA|CAMK2B|CAMK2A|ARF1	0.0045	GO.0048167
15	GO Process	negative regulation of cell population proliferation	CCL2|THBS1|FLT1|APP|CSF1R|NOS3|TENC1|RARB|CAV1|PTGES|CDH5|DLL1|SDC4|BCL2|RARG	0.0046	GO.0008285
95	GO Process	cellular metabolic process	RCN1|MAP2K5|PLOD1|ITGB4|CYP24A1|MMP2|CCL2|GAPDH|RBP1|PPP3R1|PLEK|ENO1|CDC25B|PXDN|ARF3|PDGFRA|THBS1|RORA|PRKCG|AKT3|CAD|ADD1|FLT1|PLOD2|APP|CSF1R|KIT|CCR5|CAPN2|NOS3|MELK|CKB|PLK1|DDB1|CDC25A|ARF4|BRSK1|TENC1|NQO1|CDC25C|RORC|PRKCH|RARB|CYP2R1|NR1I2|LCK|SNCA|CAV1|RGS12|PTGES|USP1|MAP4K4|PTMA|FYN|FN1|PTRF|SFPQ|EPHA2|RXRG|TGM2|GSTO1|SLK|FLNA|PTPN1|SDC4|SPTAN1|DNM1|TLR4|RXRB|ANXA1|PIR|PSIP1|SDC1|XRCC5|G6PD|PPP3CA|CAMK2B|LDHB|BCL2|CAMK2A|DYRK1A|PAICS|CDC42|PDIA6|RARG|TOP2B|WEE1|PBK|SPTBN2|CFL1|ARF1|NR1H4|PRKCSH|CAMK1D|MCM3	0.0046	GO.0044237
11	GO Process	gland development	LAMA5|PDGFRA|CAD|CSF1R|ITGA2|CAV1|EPHA2|TGM2|ANXA1|BCL2|RARG	0.0046	GO.0048732
4	GO Process	endothelial cell development	ICAM1|TJP1|ARHGEF26|CLIC4	0.0047	GO.0001885
6	GO Process	cell-matrix adhesion	ITGB4|LAMA5|ITGA2|ZYX|FN1|BCL2	0.0047	GO.0007160
4	GO Process	establishment or maintenance of apical/basal cell polarity	ARF4|CLIC4|FSCN1|CDC42	0.0047	GO.0035088
4	GO Process	negative regulation of G protein-coupled receptor signaling pathway	APLP1|PLEK|SNCA|DNM1	0.0047	GO.0045744
9	GO Process	regulation of ERK1 and ERK2 cascade	CCL2|PDGFRA|ICAM1|APP|CSF1R|FN1|EPHA2|PTPN1|TLR4	0.0047	GO.0070372
15	GO Process	regulation of secretion by cell	GAPDH|CSF1R|STXBP6|SNCA|MAP4K4|FN1|MYH10|DPP4|SDC4|TLR4|ANXA1|SDC1|PPP3CA|CAMK2A|NR1H4	0.0047	GO.1903530
11	GO Process	positive regulation of establishment of protein localization	ARHGEF5|GAPDH|APP|CSF1R|CCT2|FYN|MYO1C|MYH10|TLR4|BCL2|NR1H4	0.0047	GO.1904951
12	GO Process	regulation of cellular protein localization	APP|CCT2|PLK1|BCL2L1|DAB2|PRKCH|FYN|EPHA2|MYO1C|PTPN1|BCL2|NUMB	0.0049	GO.1903827
3	GO Process	glandular epithelial cell development	RARB|DLL1|RARG	0.005	GO.0002068

<table>
<thead><tr><th></th><th></th><th></th><th></th><th></th><th></th></tr></thead>
<tbody>
<tr><td>91</td><td>GO Process</td><td>nitrogen compound metabolic process</td><td>RCN1|MAP2K5|PLOD1|MYH9|MMP2|CCL2|GAPDH|PPP3R1|ENO1|CDC25B|PDGFRA|THBS1|VWF|RORA|PRKCG|AKT3|CAD|ADD1|FLT1|PLOD2|APP|CSF1R|KIT|NPC1L1|CCR5|CAPN2|NOS3|MELK|CKB|PLK1|DDB1|CDC25A|ARF4|MMP14|BRSK1|TENC1|NQO1|CALR|CDC25C|RORC|PRKCH|RARB|NR1I2|LCK|SNCA|RGS12|USP1|MAP4K4|PTMA|ANXA2|FYN|FN1|PTRF|SFPQ|EPHA2|RXRG|DPP4|TGM2|GSTO1|SLK|FLNA|PTPN1|SDC4|SPTAN1|TLR4|RXRB|ANXA1|PIR|PSIP1|SDC1|XRCC5|G6PD|PPP3CA|CAMK2B|LDHB|BCL2|CAMK2A|DYRK1A|PAICS|CDC42|PDIA6|RARG|TOP2B|WEE1|PBK|SPTBN2|CFL1|NR1H4|PRKCSH|CAMK1D|MCM3</td><td>0.005</td><td>GO.0006807</td></tr>
<tr><td>2</td><td>GO Process</td><td>regulation of nitrogen utilization</td><td>BCL2|NR1H4</td><td>0.005</td><td>GO.0006808</td></tr>
<tr><td>12</td><td>GO Process</td><td>response to toxic substance</td><td>PXDN|ICAM1|NOS3|BCL2L1|NQO1|FYN|GSTO1|ANXA1|SDC1|G6PD|PPP3CA|BCL2</td><td>0.005</td><td>GO.0009636</td></tr>
<tr><td>4</td><td>GO Process</td><td>response to amine</td><td>CAD|ICAM1|ITGA2|PPP3CA</td><td>0.005</td><td>GO.0014075</td></tr>
<tr><td>75</td><td>GO Process</td><td>regulation of metabolic process</td><td>ARHGEF5|MAP2K5|MYH9|EHD4|CCL2|GAPDH|PPP3R1|PLEK|ENO1|CDC25B|HSPB1|PDGFRA|THBS1|RORA|DNAJB6|PRKCG|ICAM1|FLT1|APP|CSF1R|KIT|ITGA2|NOS3|CCT2|PLK1|DDB1|BCL2L1|CDC25A|ARF4|MMP14|DAB2|NQO1|CALR|CDC25C|RORC|PRKCH|RARB|NR1I2|LCK|SNCA|CAV1|USP1|MAP4K4|ANXA2|FYN|FN1|FAS|PTRF|SFPQ|EPHA2|MYO1C|RXRG|DLL1|SLK|FLNA|PTPN1|SDC4|TLR4|RXRB|ANXA1|PIR|PSIP1|XRCC5|G6PD|PPP3CA|BCL2|CAMK2A|DYRK1A|RARG|PBK|ARF1|VIM|NR1H4|APBA2|CAMK1D</td><td>0.005</td><td>GO.0019222</td></tr>
<tr><td>7</td><td>GO Process</td><td>membrane docking</td><td>PLEK|TUBB4A|ICAM1|EXOC6B|PLK1|TUBA1A|TUBB4B</td><td>0.005</td><td>GO.0022406</td></tr>
<tr><td>3</td><td>GO Process</td><td>monocyte differentiation</td><td>MYH9|CSF1R|PIR</td><td>0.005</td><td>GO.0030224</td></tr>
<tr><td>8</td><td>GO Process</td><td>cellular response to oxidative stress</td><td>PDGFRA|NOS3|MELK|NQO1|SNCA|ANXA1|G6PD|BCL2</td><td>0.005</td><td>GO.0034599</td></tr>
<tr><td>6</td><td>GO Process</td><td>negative regulation of protein catabolic process</td><td>PRKCG|SNCA|ANXA2|FYN|FLNA|PBK</td><td>0.005</td><td>GO.0042177</td></tr>
<tr><td>10</td><td>GO Process</td><td>regulation of MAP kinase activity</td><td>ARHGEF5|MAP2K5|THBS1|FLT1|APP|KIT|CAV1|MAP4K4|PTPN1|TLR4</td><td>0.005</td><td>GO.0043405</td></tr>
<tr><td>2</td><td>GO Process</td><td>hydroxylysine metabolic process</td><td>PLOD1|PLOD2</td><td>0.005</td><td>GO.0046946</td></tr>
<tr><td>2</td><td>GO Process</td><td>hydroxylysine biosynthetic process</td><td>PLOD1|PLOD2</td><td>0.005</td><td>GO.0046947</td></tr>
<tr><td>6</td><td>GO Process</td><td>negative regulation of cytoskeleton organization</td><td>TUBB4A|ADD1|SNCA|SPTAN1|DYRK1A|SPTBN2</td><td>0.005</td><td>GO.0051494</td></tr>
<tr><td>9</td><td>GO Process</td><td>regulation of leukocyte cell-cell adhesion</td><td>CCL2|ICAM1|LCK|CAV1|FYN|DPP4|SDC4|ANXA1|CDC42</td><td>0.005</td><td>GO.1903037</td></tr>
<tr><td>3</td><td>GO Process</td><td>regulation of vascular endothelial cell proliferation</td><td>CCL2|AKT3|FLT1</td><td>0.005</td><td>GO.1905562</td></tr>
<tr><td>3</td><td>GO Process</td><td>negative regulation of anoikis</td><td>BCL2L1|CAV1|BCL2</td><td>0.005</td><td>GO.2000811</td></tr>
<tr><td>5</td><td>GO Process</td><td>hematopoietic progenitor cell differentiation</td><td>PLEK|PDGFRA|KIT|XRCC5|BCL2</td><td>0.0052</td><td>GO.0002244</td></tr>
<tr><td>5</td><td>GO Process</td><td>epithelial cell migration</td><td>MYH9|KIT|NOS3|DPP4|MMRN2</td><td>0.0052</td><td>GO.0010631</td></tr>
<tr><td>9</td><td>GO Process</td><td>negative regulation of response to external stimulus</td><td>CCL2|PDGFRA|THBS1|RORA|NOS3|CDH5|ANXA2|PBK|NR1H4</td><td>0.0053</td><td>GO.0032102</td></tr>
<tr><td>15</td><td>GO Process</td><td>negative regulation of cell differentiation</td><td>RORA|NGEF|APP|CALR|RARB|CAV1|MAP4K4|DLL1|TLR4|ANXA1|G6PD|PPP3CA|BCL2|RARG|VIM</td><td>0.0053</td><td>GO.0045596</td></tr>
<tr><td>7</td><td>GO Process</td><td>positive regulation of apoptotic signaling pathway</td><td>THBS1|BCL2L1|LCK|CAV1|FAS|SFPQ|BCL2</td><td>0.0053</td><td>GO.2001235</td></tr>
<tr><td>20</td><td>GO Process</td><td>cell-cell signaling</td><td>PPP3R1|PRKCG|TJP1|CCR5|DDB1|BRSK1|DAB2|NQO1|ZYX|SNCA|CAV1|DLL1|DNM1|ANXA1|SDC1|PPP3CA|CAMK2A|CDC42|RARG|APBA2</td><td>0.0054</td><td>GO.0007267</td></tr>
<tr><td>13</td><td>GO Process</td><td>behavior</td><td>THBS1|PRKCG|APP|KIT|ARF4|BRSK1|SNCA|FYN|DPP4|DNM1|BCL2|SPTBN2|APBA2</td><td>0.0054</td><td>GO.0007610</td></tr>
<tr><td>29</td><td>GO Process</td><td>positive regulation of cellular biosynthetic process</td><td>MAP2K5|PPP3R1|ENO1|HSPB1|THBS1|RORA|ICAM1|APP|ITGA2|NOS3|CCT2|ARF4|DAB2|RORC|RARB|NR1I2|SNCA|SFPQ|RXRG|DLL1|TLR4|RXRB|ANXA1|PSIP1|XRCC5|PPP3CA|RARG|VIM|NR1H4</td><td>0.0054</td><td>GO.0031328</td></tr>
<tr><td>9</td><td>GO Process</td><td>cellular response to abiotic stimulus</td><td>CNN2|ITGA2|DDB1|BCL2L1|CDC25A|FAS|TLR4|XRCC5|PBK</td><td>0.0054</td><td>GO.0071214</td></tr>
<tr><td>5</td><td>GO Process</td><td>positive regulation of cell cycle phase transition</td><td>CDC25B|APP|CDC25A|CDC25C|ANXA1</td><td>0.0054</td><td>GO.1901989</td></tr>
<tr><td>8</td><td>GO Process</td><td>lymphocyte differentiation</td><td>RORA|KIT|RORC|JAG2|LCK|DLL1|ANXA1|BCL2</td><td>0.0055</td><td>GO.0030098</td></tr>
<tr><td>17</td><td>GO Process</td><td>regulation of catabolic process</td><td>GAPDH|HSPB1|PRKCG|APP|PLK1|DDB1|BCL2L1|DAB2|SNCA|CAV1|ANXA2|FYN|FLNA|PTPN1|BCL2|PBK|VIM</td><td>0.0056</td><td>GO.0009894</td></tr>
<tr><td>6</td><td>GO Process</td><td>Fc receptor signaling pathway</td><td>PPP3R1|KIT|FYN|MYO1C|PPP3CA|CDC42</td><td>0.0058</td><td>GO.0038093</td></tr>
<tr><td>15</td><td>GO Process</td><td>regulated exocytosis</td><td>PLEK|THBS1|VWF|CNN2|FERMT3|APP|KIT|ANXA5|CCT2|TUBB4B|ANXA2|FN1|FLNA|SPTAN1|XRCC5</td><td>0.0058</td><td>GO.0045055</td></tr>
<tr><td>5</td><td>GO Process</td><td>epithelial cell proliferation</td><td>BCL2L2|COL8A1|KIT|MMP14|EPHA2</td><td>0.0058</td><td>GO.0050673</td></tr>
<tr><td>18</td><td>GO Process</td><td>multi-organism reproductive process</td><td>MMP2|CDC25B|BCL2L2|CAD|APP|KIT|ITGA2|NOS3|CCT2|PLK1|BCL2L1|CALR|CDC25C|JAG2|CLIC4|BCL2|SEPT7|SUN1</td><td>0.0059</td><td>GO.0044703</td></tr>
<tr><td>4</td><td>GO Process</td><td>positive regulation of muscle contraction</td><td>ENO1|KIT|ITGA2|GSTO1</td><td>0.0059</td><td>GO.0045933</td></tr>
<tr><td>4</td><td>GO Process</td><td>positive regulation of phagocytosis</td><td>CCL2|ITGA2|CALR|CAMK1D</td><td>0.0059</td><td>GO.0050766</td></tr>
<tr><td>11</td><td>GO Process</td><td>plasma membrane bounded cell projection assembly</td><td>LAMA5|TUBB4A|KIT|PLK1|TUBA1A|TUBB4B|ARHGEF26|MYH10|FLNA|FSCN1|SEPT7</td><td>0.0059</td><td>GO.0120031</td></tr>
<tr><td>3</td><td>GO Process</td><td>regulation of bicellular tight junction assembly</td><td>TJP1|PRKCH|MYO1C</td><td>0.0061</td><td>GO.2000810</td></tr>
<tr><td>4</td><td>GO Process</td><td>actin filament bundle assembly</td><td>ADD1|ZYX|FSCN1|MYO1B</td><td>0.0062</td><td>GO.0051017</td></tr>
<tr><td>6</td><td>GO Process</td><td>kidney epithelium development</td><td>LAMA5|RARB|DLL1|SDC4|SDC1|BCL2</td><td>0.0062</td><td>GO.0072073</td></tr>
<tr><td>7</td><td>GO Process</td><td>positive regulation of peptidase activity</td><td>APP|LCK|SNCA|CAV1|FYN|FN1|FAS</td><td>0.0063</td><td>GO.0010952</td></tr>
<tr><td>9</td><td>GO Process</td><td>positive regulation of response to external stimulus</td><td>HSPB1|THBS1|APP|ITGA2|CALR|SNCA|TGM2|TLR4|CAMK1D</td><td>0.0063</td><td>GO.0032103</td></tr>
<tr><td>5</td><td>GO Process</td><td>positive regulation of neuron death</td><td>APP|NQO1|SNCA|FYN|TLR4</td><td>0.0063</td><td>GO.1901216</td></tr>
<tr><td>7</td><td>GO Process</td><td>negative regulation of secretion by cell</td><td>STXBP6|SNCA|MAP4K4|FN1|ANXA1|PPP3CA|NR1H4</td><td>0.0063</td><td>GO.1903531</td></tr>
<tr><td>5</td><td>GO Process</td><td>ureteric bud development</td><td>LAMA5|RARB|SDC4|SDC1|BCL2</td><td>0.0066</td><td>GO.0001657</td></tr>
<tr><td>6</td><td>GO Process</td><td>leukocyte chemotaxis</td><td>ARHGEF5|CCL2|FLT1|KIT|CCR5|ANXA1</td><td>0.0066</td><td>GO.0030595</td></tr>
<tr><td>7</td><td>GO Process</td><td>positive regulation of peptidyl-tyrosine phosphorylation</td><td>EHD4|ICAM1|APP|CSF1R|KIT|FYN|PTPN1</td><td>0.0066</td><td>GO.0050731</td></tr>
<tr><td>11</td><td>GO Process</td><td>regulation of peptidase activity</td><td>MAP2K5|GAPDH|THBS1|DNAJB6|APP|LCK|SNCA|CAV1|FYN|FN1|FAS</td><td>0.0066</td><td>GO.0052547</td></tr>
<tr><td>3</td><td>GO Process</td><td>macrophage differentiation</td><td>APP|CSF1R|CDC42</td><td>0.0067</td><td>GO.0030225</td></tr>
<tr><td>2</td><td>GO Process</td><td>positive regulation of extracellular exosome assembly</td><td>SDC4|SDC1</td><td>0.0067</td><td>GO.1903553</td></tr>
<tr><td>2</td><td>GO Process</td><td>negative regulation of vascular endothelial cell proliferation</td><td>CCL2|FLT1</td><td>0.0067</td><td>GO.1905563</td></tr>
<tr><td>9</td><td>GO Process</td><td>apoptotic signaling pathway</td><td>BCL2L2|MELK|BCL2L1|CAV1|ANXA6|FAS|EPHA2|TLR4|BCL2</td><td>0.0068</td><td>GO.0097190</td></tr>
<tr><td>5</td><td>GO Process</td><td>regulation of protein localization to plasma membrane</td><td>BCL2L1|DAB2|PRKCH|EPHA2|NUMB</td><td>0.0068</td><td>GO.1903076</td></tr>
<tr><td>4</td><td>GO Process</td><td>alpha-beta T cell differentiation</td><td>RORA|RORC|ANXA1|BCL2</td><td>0.0069</td><td>GO.0046632</td></tr>
<tr><td>8</td><td>GO Process</td><td>regulation of peptidyl-tyrosine phosphorylation</td><td>EHD4|ICAM1|APP|CSF1R|KIT|CAV1|FYN|PTPN1</td><td>0.0069</td><td>GO.0050730</td></tr>
<tr><td>4</td><td>GO Process</td><td>positive regulation of stress fiber assembly</td><td>ARHGEF5|ARHGEF15|SDC4|CDC42</td><td>0.0069</td><td>GO.0051496</td></tr>
<tr><td>4</td><td>GO Process</td><td>negative regulation of reactive oxygen species metabolic process</td><td>CAV1|FYN|G6PD|BCL2</td><td>0.0069</td><td>GO.2000378</td></tr>
<tr><td>9</td><td>GO Process</td><td>negative regulation of cell development</td><td>NGEF|APP|CALR|MAP4K4|DLL1|G6PD|PPP3CA|BCL2|VIM</td><td>0.0072</td><td>GO.0010721</td></tr>
<tr><td>9</td><td>GO Process</td><td>response to lipopolysaccharide</td><td>CCL2|ICAM1|CCR5|NOS3|SNCA|PTGES|FAS|TLR4|NR1H4</td><td>0.0072</td><td>GO.0032496</td></tr>
<tr><td>21</td><td>GO Process</td><td>lipid metabolic process</td><td>CYP24A1|RBP1|PLEK|ARF3|PDGFRA|APP|CSF1R|KIT|NPC1L1|CYP2R1|NR1I2|LCK|SNCA|CAV1|PTGES|FYN|SDC4|SDC1|G6PD|ARF1|NR1H4</td><td>0.0074</td><td>GO.0006629</td></tr>
<tr><td>6</td><td>GO Process</td><td>branching morphogenesis of an epithelial tube</td><td>PPP3R1|LAMA5|MMP14|EPHA2|CLIC4|BCL2</td><td>0.0074</td><td>GO.0048754</td></tr>
<tr><td>4</td><td>GO Process</td><td>negative regulation of coagulation</td><td>PDGFRA|ANXA5|NOS3|ANXA2</td><td>0.0074</td><td>GO.0050819</td></tr>
<tr><td>4</td><td>GO Process</td><td>positive regulation of wound healing</td><td>PLEK|THBS1|TLR4|ANXA1</td><td>0.0074</td><td>GO.0090303</td></tr>
<tr><td>3</td><td>GO Process</td><td>negative regulation of synapse organization</td><td>NGEF|FYN|ARHGEF15</td><td>0.0074</td><td>GO.1905809</td></tr>
<tr><td>5</td><td>GO Process</td><td>dendrite development</td><td>APP|ARF4|FYN|CAMK2A|CDC42</td><td>0.0076</td><td>GO.0016358</td></tr>
</tbody>
</table>

6	GO Process	positive regulation of lipid metabolic process	PDGFRA|FLT1|APP|KIT|ANXA1|NR1H4	0.0076	GO.0045834
5	GO Process	regulation of protein tyrosine kinase activity	APP|CSF1R|CAV1|FYN|PTPN1	0.0076	GO.0061097
10	GO Process	immune response-regulating signaling pathway	PPP3R1|KIT|LCK|FYN|MYO1C|TLR4|PPP3CA|BCL2|CDC42|NR1H4	0.0077	GO.0002764
11	GO Process	response to peptide	CAD|ICAM1|APP|CAV1|FYN|PTPN1|DNM1|TLR4|ANXA1|TBC1D4|CAMK2A	0.0077	GO.1901652
9	GO Process	Wnt signaling pathway	PPP3R1|DDB1|DAB2|CAV1|SDC1|PPP3CA|CAMK2A|CDC42|RARG	0.0078	GO.0016055
8	GO Process	regulation of cysteine-type endopeptidase activity	MAP2K5|THBS1|DNAJB6|APP|LCK|SNCA|FYN|FAS	0.0078	GO.2000116
7	GO Process	response to reactive oxygen species	PDGFRA|NOS3|NQO1|FYN|ANXA1|SDC1|BCL2	0.0081	GO.0000302
3	GO Process	ruffle organization	PLEK|CSF1R|ARHGEF26	0.0081	GO.0031529
3	GO Process	endoplasmic reticulum calcium ion homeostasis	APP|TGM2|BCL2	0.0081	GO.0032469
3	GO Process	positive regulation of macrophage activation	THBS1|APP|TLR4	0.0081	GO.0043032
7	GO Process	synapse organization	APP|SNCA|FYN|MYH10|CDC42|SPTBN2|ARF1	0.0081	GO.0050808
3	GO Process	negative regulation of protein localization to plasma membrane	BCL2L1|DAB2|NUMB	0.0081	GO.1903077
9	GO Process	in utero embryonic development	MYH9|PDGFRA|ADD1|CAPN2|NOS3|BCL2L1|JAG2|MYH10|APBA2	0.0082	GO.0001701
6	GO Process	negative regulation of neuron apoptotic process	CCL2|PRKCG|BCL2L1|SNCA|FYN|BCL2	0.0082	GO.0043524
6	GO Process	positive regulation of chemotaxis	HSPB1|THBS1|APP|ITGA2|CALR|CAMK1D	0.0082	GO.0050921
6	GO Process	response to UV	DDB1|CDC25A|BRSK1|USP1|BCL2|PBK	0.0085	GO.0009411
7	GO Process	negative regulation of neuron death	CCL2|PRKCG|APP|BCL2L1|SNCA|FYN|BCL2	0.0085	GO.1901215
8	GO Process	steroid metabolic process	CYP24A1|PDGFRA|APP|NPC1L1|CYP2R1|NR1I2|G6PD|NR1H4	0.0086	GO.0008202
4	GO Process	regulation of nitric oxide biosynthetic process	ICAM1|APP|CAV1|TLR4	0.0086	GO.0045428
4	GO Process	myeloid cell development	APP|KIT|ANXA2|G6PD	0.0086	GO.0061515
2	GO Process	positive regulation of platelet activation	PLEK|TLR4	0.0087	GO.0010572
10	GO Process	regulation of lipid metabolic process	PDGFRA|RORA|FLT1|APP|KIT|RORC|SNCA|CAV1|ANXA1|NR1H4	0.0087	GO.0019216
3	GO Process	focal adhesion assembly	LAMA5|ITGA2|BCL2	0.0087	GO.0048041
2	GO Process	establishment of Sertoli cell barrier	ICAM1|FLNA	0.0087	GO.0097368
2	GO Process	positive regulation of establishment of endothelial barrier	ADD1|CDH5	0.0087	GO.1903142
2	GO Process	positive regulation of oxidative stress-induced neuron death	APP|TLR4	0.0087	GO.1903223
5	GO Process	positive regulation of reactive oxygen species metabolic process	THBS1|ICAM1|APP|SNCA|TLR4	0.0087	GO.2000379
6	GO Process	regulation of actin filament polymerization	ADD1|ICAM1|MYO1C|SPTAN1|SPTBN2|ARF1	0.0088	GO.0030833
16	GO Process	negative regulation of catalytic activity	MAP2K5|GAPDH|HSPB1|THBS1|DNAJB6|APP|ANXA5|NOS3|PLK1|NQO1|SNCA|CAV1|ANXA2|CNN3|PTPN1|ANXA1	0.0088	GO.0043086
8	GO Process	rhythmic process	PDGFRA|RORA|PRKCG|NOS3|RORC|SFPQ|ANXA1|DYRK1A	0.0088	GO.0048511
7	GO Process	homeostasis of number of cells	AKT3|ADD1|KIT|NOS3|ANXA1|G6PD|BCL2	0.0088	GO.0048872
7	GO Process	positive regulation of T cell activation	CCL2|LCK|CAV1|FYN|DPP4|ANXA1|CDC42	0.0088	GO.0050870
8	GO Process	kidney development	LAMA5|PDGFRA|TENC1|RARB|DLL1|SDC4|SDC1|BCL2	0.009	GO.0001822
8	GO Process	positive regulation of cytosolic calcium ion concentration	PDGFRA|APP|CCR5|LCK|SNCA|CAV1|TGM2|GSTO1	0.009	GO.0007204
6	GO Process	regulation of G protein-coupled receptor signaling pathway	APLP1|PLEK|APP|SNCA|RGS12|DNM1	0.0091	GO.0008277
8	GO Process	positive regulation of ion transport	CCL2|SNCA|CAV1|GSTO1|FLNA|G6PD|CAMK2A|ABCB1	0.0092	GO.0043270
4	GO Process	positive regulation of phospholipase activity	PDGFRA|FLT1|KIT|ARF4	0.0094	GO.0010518
5	GO Process	cerebellum development	RORA|CKB|MYH10|DLL1|SPTBN2	0.0094	GO.0021549
5	GO Process	maintenance of protein location	CAV1|FLNA|SUN1|FLNB|TLN2	0.0094	GO.0045185
4	GO Process	regulation of focal adhesion assembly	THBS1|MMP14|SLK|SDC4	0.0094	GO.0051893
3	GO Process	positive regulation of protein processing	MYH9|ENO1|MMP14	0.0095	GO.0010954
13	GO Process	positive regulation of immune response	MMP2|PRKCH|LCK|CAV1|FYN|SFPQ|MYO1C|TLR4|ANXA1|XRCC5|BCL2|CDC42|NR1H4	0.0095	GO.0050778
8	GO Process	regulation of cellular response to growth factor stimulus	THBS1|FLT1|DAB2|CAV1|MYO1C|DLL1|PTPN1|MMRN2	0.0095	GO.0090287
5	GO Process	regulation of calcium ion transport into cytosol	SNCA|CAV1|FYN|GSTO1|BCL2	0.0096	GO.0010522
10	GO Process	cell surface receptor signaling pathway involved in cell-cell signaling	PPP3R1|DDB1|DAB2|SNCA|CAV1|SDC1|PPP3CA|CAMK2A|CDC42|RARG	0.0101	GO.1905114
6	GO Process	cellular response to lipopolysaccharide	CCL2|ICAM1|CCR5|NOS3|TLR4|NR1H4	0.0102	GO.0071222
3	GO Process	positive regulation of blood coagulation	PLEK|THBS1|TLR4	0.0104	GO.0030194
3	GO Process	detection of mechanical stimulus involved in sensory perception	KIT|ITGA2|FYN	0.0104	GO.0050974
4	GO Process	negative regulation of response to cytokine stimulus	MAP2K5|PXDN|CAV1|NR1H4	0.0104	GO.0060761
12	GO Process	regulation of transmembrane transport	THBS1|APP|SNCA|CAV1|FYN|GSTO1|FLNA|CLIC4|G6PD|BCL2|NR1H4|ABCB1	0.0105	GO.0034762
11	GO Process	response to nutrient levels	CYP24A1|CAD|ICAM1|ITGA2|NQO1|CAV1|FYN|FAS|G6PD|BCL2|NR1H4	0.0107	GO.0031667
6	GO Process	regulation of muscle cell differentiation	KIT|MMP14|DLL1|G6PD|BCL2|CDC42	0.0107	GO.0051147
6	GO Process	activation of MAPK activity	MAP2K5|THBS1|APP|KIT|PTPN1|TLR4	0.0109	GO.0000187
2	GO Process	regulation of the force of heart contraction by chemical signal	NOS3|CAV1	0.0109	GO.0003057
2	GO Process	retinal pigment epithelium development	RARB|RARG	0.0109	GO.0003406
6	GO Process	response to ionizing radiation	ICAM1|BCL2L1|CYP2R1|ANXA1|XRCC5|BCL2	0.0109	GO.0010212
6	GO Process	regulation of G2/M transition of mitotic cell cycle	TUBB4A|APP|PLK1|TUBA1A|BRSK1|TUBB4B	0.0109	GO.0010389
4	GO Process	peptide cross-linking	THBS1|FN1|TGM2|ANXA1	0.0109	GO.0018149
2	GO Process	astrocyte cell migration	CCL2|MMP14	0.0109	GO.0043615
7	GO Process	regulation of protein kinase B signaling	PDGFRA|THBS1|APP|KIT|LCK|FYN|EPHA2	0.0109	GO.0051896
2	GO Process	modulation by symbiont of host programmed cell death	GAPDH|BCL2L1	0.0109	GO.0052040
2	GO Process	modulation by organism of apoptotic process in other organism involved in symbiotic interaction	GAPDH|BCL2L1	0.0109	GO.0052433
3	GO Process	response to sterol	RORA|CCR5|RORC	0.0111	GO.0036314
6	GO Process	maintenance of location	CALR|CAV1|FLNA|SUN1|FLNB|TLN2	0.0112	GO.0051235
17	GO Process	apoptotic process	APLP1|GAPDH|BCL2L2|APP|MELK|BCL2L1|DAB2|JAG2|SNCA|CAV1|ANXA6|FAS|EPHA2|SLK|TLR4|BCL2|CAMK2A	0.0113	GO.0006915
4	GO Process	positive regulation of protein tyrosine kinase activity	APP|CSF1R|FYN|PTPN1	0.0113	GO.0061098
29	GO Process	protein localization	MYH9|PPP3R1|PLEK|HSPB1|LAMA5|ARF3|EXOC6B|ITGA2|PLK1|ARF4|DAB2|CALR|STXBP6|CAV1|ANXA2|MYO1C|COPA|FLNA|TLR4|ANXA1|TBC1D4|XRCC5|PPP3CA|SUN1|FLNB|ARF1|NR1H4|APBA2|TLN2	0.0114	GO.0008104
7	GO Process	negative regulation of neuron differentiation	NGEF|APP|CALR|MAP4K4|DLL1|PPP3CA|VIM	0.0114	GO.0045665
7	GO Process	cellular response to nutrient levels	CYP24A1|ICAM1|CAV1|FYN|FAS|BCL2|NR1H4	0.0116	GO.0031669
5	GO Process	endoplasmic reticulum unfolded protein response	CCL2|ADD1|CALR|PTPN1|PDIA6	0.0118	GO.0030968
4	GO Process	endothelial cell migration	MYH9|NOS3|DPP4|MMRN2	0.0118	GO.0043542
5	GO Process	reactive oxygen species metabolic process	PXDN|RORA|NOS3|NQO1|BCL2	0.0118	GO.0072593

5	GO Process	myeloid leukocyte migration	ARHGEF5|CCL2|FLT1|KIT|ANXA1	0.0118	GO.0097529
8	GO Process	immune response-regulating cell surface receptor signaling pathway	PPP3R1|KIT|LCK|FYN|MYO1C|PPP3CA|BCL2|CDC42	0.0119	GO.0002768
3	GO Process	regulation of myeloid cell apoptotic process	CCR5|ANXA1|BCL2	0.012	GO.0033032
15	GO Process	sexual reproduction	CDC25B|BCL2L2|APP|KIT|NOS3|CCT2|PLK1|BCL2L1|CALR|CDC25C|JAG2|CLIC4|BCL2|SEPT7|SUN1	0.0122	GO.0019953
4	GO Process	mesenchymal cell development	LAMA5|FN1|BCL2|CFL1	0.0124	GO.0014031
7	GO Process	regulation of cysteine-type endopeptidase activity involved in apoptotic process	MAP2K5|THBS1|DNAJB6|APP|LCK|SNCA|FAS	0.0125	GO.0043281
15	GO Process	multicellular organismal reproductive process	CDC25B|BCL2L2|PDGFRA|APP|KIT|NOS3|PLK1|BCL2L1|CALR|CDC25C|JAG2|ANXA1|BCL2|SEPT7|SUN1	0.0125	GO.0048609
5	GO Process	regulation of cell-matrix adhesion	THBS1|MMP14|SLK|SDC4|BCL2	0.0126	GO.0001952
25	GO Process	establishment of localization in cell	MYH9|EHD4|PPP3R1|HSPB1|APP|KIT|CCR5|TUBA1A|TUBA1C|ARF4|BRSK1|CALR|LCK|SNCA|MYO1C|MYH10|COPA|SPTAN1|DNM1|TBC1D4|PSIP1|PPP3CA|CDC42|SUN1|SPTBN2	0.0126	GO.0051649
32	GO Process	macromolecule localization	MYH9|PPP3R1|PLEK|HSPB1|LAMA5|ARF3|EXOC6B|NPC1L1|ITGA2|CCT2|PLK1|ARF4|DAB2|CALR|STXBP6|CAV1|ANXA2|MYO1C|COPA|FLNA|TLR4|ANXA1|TBC1D4|XRCC5|PPP3CA|SUN1|FLNB|ARF1|NR1H4|APBA2|TLN2|ABCB1	0.0129	GO.0033036
3	GO Process	astrocyte development	APP|DLL1|VIM	0.013	GO.0014002
8	GO Process	striated muscle tissue development	PDGFRA|CALR|RARB|CAV1|MYH10|DLL1|PPP3CA|FLNB	0.013	GO.0014706
3	GO Process	apoptotic cell clearance	THBS1|TGM2|PDIA6	0.013	GO.0043277
5	GO Process	response to alkaloid	PRKCG|CAD|ICAM1|BCL2L1|SNCA	0.013	GO.0043279
10	GO Process	regulation of lymphocyte activation	CCL2|MMP14|LCK|CAV1|FYN|DPP4|SDC4|ANXA1|BCL2|CDC42	0.013	GO.0051249
3	GO Process	dendritic spine organization	FYN|CDC42|ARF1	0.013	GO.0097061
2	GO Process	cellular response to erythropoietin	MT2A|KIT	0.0131	GO.0036018
2	GO Process	positive regulation of cell migration by vascular endothelial growth factor signaling pathway	HSPB1|MYO1C	0.0131	GO.0038089
2	GO Process	cellular response to hyperoxia	CAV1|FAS	0.0131	GO.0071455
6	GO Process	positive regulation of protein kinase B signaling	PDGFRA|THBS1|APP|KIT|LCK|FYN	0.0134	GO.0051897
6	GO Process	cellular response to xenobiotic stimulus	RORA|ICAM1|NQO1|RORC|NR1I2|GSTO1	0.0134	GO.0071466
4	GO Process	maintenance of protein location in cell	CAV1|SUN1|FLNB|TLN2	0.0135	GO.0032507
7	GO Process	regulation of protein binding	ADD1|APP|PLK1|DAB2|CAV1|ANXA2|BCL2	0.0135	GO.0043393
8	GO Process	negative regulation of catabolic process	PRKCG|BCL2L1|SNCA|ANXA2|FYN|FLNA|BCL2|PBK	0.0137	GO.0009895
13	GO Process	developmental process involved in reproduction	CDC25B|BCL2L2|PDGFRA|ICAM1|KIT|NOS3|BCL2L1|MMP14|FLNA|ANXA1|SDC1|BCL2|RARG	0.0138	GO.0003006
5	GO Process	xenobiotic metabolic process	RORA|NQO1|RORC|NR1I2|GSTO1	0.0138	GO.0006805
3	GO Process	regulation of lamellipodium assembly	EPHA2|FSCN1|CDC42	0.0139	GO.0010591
3	GO Process	regulation of vascular endothelial growth factor receptor signaling pathway	FLT1|PTPN1|MMRN2	0.0139	GO.0030947
3	GO Process	negative regulation of exocytosis	STXBP6|SNCA|ANXA1	0.0139	GO.0045920
3	GO Process	regulation of defense response to virus by virus	LCK|FYN|ARF1	0.0139	GO.0050690
6	GO Process	organelle localization by membrane tethering	PLEK|TUBB4A|EXOC6B|PLK1|TUBA1A|TUBB4B	0.014	GO.0140056
4	GO Process	positive regulation of phosphatidylinositol 3-kinase signaling	PDGFRA|FLT1|KIT|FYN	0.0141	GO.0014068
4	GO Process	negative regulation of calcium ion transport	ICAM1|NOS3|GSTO1|BCL2	0.0141	GO.0051926
8	GO Process	microtubule-based movement	MYH9|HSPB1|APP|MYO1C|MYH10|MYO1B|CDC42|SUN1	0.0142	GO.0007018
3	GO Process	epithelial cell morphogenesis	ARHGEF26|CLIC4|FLNB	0.0149	GO.0003382
3	GO Process	phagocytosis, engulfment	MYH9|THBS1|CDC42	0.0149	GO.0006911
9	GO Process	positive regulation of cell projection organization	KIT|ITGA2|FYN|FN1|FSCN1|CAMK2B|SEPT7|CDC42|CAMK1D	0.0149	GO.0031346
3	GO Process	lipopolysaccharide-mediated signaling pathway	CCL2|NOS3|TLR4	0.0149	GO.0031663
8	GO Process	negative regulation of nervous system development	NGEF|APP|CALR|MAP4K4|ARHGEF15|DLL1|PPP3CA|VIM	0.0149	GO.0051961
3	GO Process	extrinsic apoptotic signaling pathway in absence of ligand	BCL2L2|BCL2L1|BCL2	0.0149	GO.0097192
6	GO Process	regulation of response to cytokine stimulus	MAP2K5|PXDN|CAV1|PTPN1|TLR4|NR1H4	0.0151	GO.0060759
6	GO Process	cellular response to metal ion	MT2A|ADD1|NQO1|CALR|SNCA|CLIC4	0.0151	GO.0071248
4	GO Process	vascular endothelial growth factor receptor signaling pathway	HSPB1|FLT1|FYN|CDC42	0.0153	GO.0048010
2	GO Process	blood vessel maturation	MMP2|CDH5	0.0156	GO.0001955
5	GO Process	regulation of leukocyte chemotaxis	CCL2|THBS1|APP|CALR|CAMK1D	0.0156	GO.0002688
7	GO Process	axon guidance	APP|CSF1R|DOK4|FYN|MYH10|SPTAN1|SPTBN2	0.0156	GO.0007411
2	GO Process	peptidyl-lysine hydroxylation	PLOD1|PLOD2	0.0156	GO.0017185
2	GO Process	positive regulation of vesicle fusion	ANXA2|ANXA1	0.0156	GO.0031340
2	GO Process	regulation of cell-cell adhesion mediated by integrin	FERMT3|DPP4	0.0156	GO.0033632
5	GO Process	response to hydrogen peroxide	NQO1|FYN|ANXA1|SDC1|BCL2	0.0156	GO.0042542
5	GO Process	negative regulation of protein secretion	MAP4K4|FN1|ANXA1|PPP3CA|NR1H4	0.0156	GO.0050709
2	GO Process	T-helper 17 cell differentiation	RORA|RORC	0.0156	GO.0072539
2	GO Process	establishment of integrated proviral latency	PSIP1|XRCC5	0.0156	GO.0075713
2	GO Process	regulation of membrane repolarization during action potential	CAV1|FLNA	0.0156	GO.0098903
3	GO Process	response to iron ion	SNCA|G6PD|BCL2	0.0158	GO.0010039
5	GO Process	odontogenesis	ITGB4|LAMA5|PDGFRA|JAG2|SDC1	0.0158	GO.0042476
3	GO Process	actin filament capping	ADD1|SPTAN1|SPTBN2	0.0158	GO.0051693
3	GO Process	positive regulation of substrate adhesion-dependent cell spreading	CALR|FLNA|CDC42	0.0158	GO.1900026
18	GO Process	programmed cell death	APLP1|GAPDH|BCL2L2|APP|KIT|MELK|BCL2L1|DAB2|JAG2|SNCA|CAV1|ANXA6|FAS|EPHA2|SLK|TLR4|BCL2|CAMK2A	0.0163	GO.0012501
4	GO Process	interferon-gamma-mediated signaling pathway	MT2A|ICAM1|CAMK2B|CAMK2A	0.0165	GO.0060333
4	GO Process	body fluid secretion	CAD|CAV1|ANXA2|COPA	0.0172	GO.0007589
4	GO Process	regulation of interleukin-1 production	HSPB1|TLR4|ANXA1|NR1H4	0.0172	GO.0032652
4	GO Process	regulation of tyrosine phosphorylation of STAT protein	CSF1R|KIT|CAV1|FYN	0.0172	GO.0042509
10	GO Process	regulation of protein secretion	GAPDH|CSF1R|MAP4K4|FN1|MYH10|DPP4|TLR4|ANXA1|PPP3CA|NR1H4	0.0172	GO.0050708
6	GO Process	regulation of organelle assembly	ARHGEF5|PLK1|SDC4|SDC1|FSCN1|SEPT7	0.0172	GO.1902115
4	GO Process	cytokinesis	MYH9|PLK1|MYH10|CFL1	0.0179	GO.0000910
4	GO Process	intrinsic apoptotic signaling pathway in response to DNA damage	BCL2L2|BCL2L1|EPHA2|BCL2	0.0179	GO.0008630
8	GO Process	response to light stimulus	APP|KIT|DDB1|CDC25A|BRSK1|USP1|BCL2|PBK	0.0179	GO.0009416
4	GO Process	regulation of vesicle fusion	STXBP6|ANXA2|ANXA1|TBC1D4	0.0179	GO.0031338
4	GO Process	organ growth	RARB|DLL1|BCL2|RARG	0.0179	GO.0035265
9	GO Process	regulation of binding	ADD1|APP|ITGA2|PLK1|DAB2|CAV1|ANXA2|PPP3CA|BCL2	0.0179	GO.0051098
5	GO Process	positive regulation of protein localization to membrane	PRKCH|FYN|EPHA2|MYO1C|BCL2	0.0179	GO.1905477
2	GO Process	integrin activation	FERMT3|FN1	0.0181	GO.0033622

2	GO Process	retina vasculature morphogenesis in camera-type eye	ARHGEF15|CLIC4	0.0181	GO.0061299
3	GO Process	neural crest cell migration	LAMA5|FN1|CFL1	0.0193	GO.0001755
3	GO Process	fat-soluble vitamin metabolic process	CYP24A1|RBP1|CYP2R1	0.0193	GO.0006775
3	GO Process	Wnt signaling pathway, calcium modulating pathway	PPP3R1|PPP3CA|CAMK2A	0.0193	GO.0007223
3	GO Process	regulation of cellular amine metabolic process	NQO1|SNCA|NR1H4	0.0193	GO.0033238
9	GO Process	regulation of innate immune response	MMP2|APP|CAV1|FYN|SFPQ|PTPN1|TLR4|XRCC5|NR1H4	0.0193	GO.0045088
3	GO Process	cellular response to catecholamine stimulus	APLP1|APP|SNCA	0.0193	GO.0071870
3	GO Process	regulation of actin cytoskeleton reorganization	PDGFRA|CSF1R|CDC42	0.0193	GO.2000249
5	GO Process	positive regulation of inflammatory response	APP|ITGA2|SNCA|TGM2|TLR4	0.0195	GO.0050729
6	GO Process	regulation of cytokine secretion	GAPDH|CSF1R|FN1|TLR4|ANXA1|NR1H4	0.0198	GO.0050707
7	GO Process	response to alcohol	CAD|ICAM1|CCR5|BCL2L1|NQO1|FYN|G6PD	0.0198	GO.0097305
4	GO Process	regulation of chemokine production	MAP2K5|CSF1R|TLR4|NR1H4	0.0201	GO.0032642
10	GO Process	regulation of ion transmembrane transport	THBS1|APP|SNCA|CAV1|FYN|GSTO1|FLNA|CLIC4|G6PD|ABCB1	0.0201	GO.0034765
5	GO Process	striated muscle cell development	PDGFRA|FLNC|MYH10|SDC1|PPP3CA	0.0201	GO.0055002
5	GO Process	anatomical structure maturation	MMP2|CDC25B|APP|CDH5|G6PD	0.0201	GO.0071695
9	GO Process	positive regulation of protein transport	ARHGEF5|GAPDH|APP|CSF1R|FYN|MYO1C|MYH10|TLR4|NR1H4	0.0204	GO.0051222
3	GO Process	regulation of dendritic spine morphogenesis	NGEF|CAMK2B|CFL1	0.0204	GO.0061001
5	GO Process	regulation of endothelial cell proliferation	CCL2|THBS1|AKT3|FLT1|CAV1	0.0206	GO.0001936
5	GO Process	regulation of cellular ketone metabolic process	NQO1|SNCA|CAV1|ANXA1|NR1H4	0.0206	GO.0010565
9	GO Process	forebrain development	APP|CSF1R|RARB|FYN|MYH10|FLNA|SUN1|TOP2B|NUMB	0.0206	GO.0030900
5	GO Process	G1/S transition of mitotic cell cycle	CDC25A|PPP3CA|CAMK2A|WEE1|MCM3	0.021	GO.0000082
2	GO Process	membrane raft assembly	CAV1|ANXA2	0.021	GO.0001765
2	GO Process	endothelial cell morphogenesis	ARHGEF26|CLIC4	0.021	GO.0001886
2	GO Process	cytoskeletal anchoring at plasma membrane	FLNB|TLN2	0.021	GO.0007016
2	GO Process	regulation of low-density lipoprotein particle clearance	ANXA2|NR1H4	0.021	GO.0010988
2	GO Process	lateral ventricle development	MYH10|NUMB	0.021	GO.0021670
7	GO Process	negative regulation of cell migration	MAP2K5|CCL2|THBS1|CALR|MMRN2|CLIC4|BCL2	0.021	GO.0030336
5	GO Process	mammary gland development	CAD|CSF1R|ITGA2|CAV1|EPHA2	0.021	GO.0030879
2	GO Process	negative regulation of vascular endothelial growth factor receptor signaling pathway	PTPN1|MMRN2	0.021	GO.0030948
14	GO Process	regulation of cellular catabolic process	GAPDH|HSPB1|PRKCG|APP|PLK1|BCL2L1|DAB2|SNCA|CAV1|ANXA2|PTPN1|BCL2|PBK|VIM	0.021	GO.0031329
2	GO Process	calcineurin-NFAT signaling cascade	PPP3R1|PPP3CA	0.021	GO.0033173
2	GO Process	xenobiotic transport	NR1I2|ABCB1	0.021	GO.0042908
18	GO Process	negative regulation of protein metabolic process	MAP2K5|GAPDH|HSPB1|THBS1|DNAJB6|PRKCG|APP|PLK1|CALR|SNCA|CAV1|ANXA2|FYN|FLNA|PTPN1|TLR4|G6PD|PBK	0.021	GO.0051248
2	GO Process	establishment of endothelial intestinal barrier	ICAM1|TJP1	0.021	GO.0090557
2	GO Process	positive regulation of receptor binding	APP|ANXA2	0.021	GO.1900122
2	GO Process	regulation of receptor catabolic process	ANXA2|PTPN1	0.021	GO.2000644
3	GO Process	morphogenesis of a polarized epithelium	LAMA5|BRSK1|CDC42	0.0213	GO.0001738
3	GO Process	positive regulation of response to endoplasmic reticulum stress	APP|CAV1|PTPN1	0.0213	GO.1905898
8	GO Process	regulation of T cell activation	CCL2|LCK|CAV1|FYN|DPP4|SDC4|ANXA1|CDC42	0.0214	GO.0050863
5	GO Process	regulation of embryonic development	MAP2K5|LAMA4|LAMA5|DLL1|SEPT7	0.0221	GO.0045995
7	GO Process	response to endoplasmic reticulum stress	CCL2|THBS1|ADD1|CALR|PTPN1|BCL2|PDIA6	0.0222	GO.0034976
4	GO Process	regulation of dendrite morphogenesis	NGEF|PPP3CA|CAMK2B|CFL1	0.0222	GO.0048814
10	GO Process	regulation of neuron projection development	NGEF|BRSK1|MAP4K4|FYN|FN1|PPP3CA|CAMK2B|CFL1|VIM|CAMK1D	0.0223	GO.0010975
9	GO Process	skin development	ITGB4|LAMA5|ITGA2|EPHA2|DLL1|CLIC4|ANXA1|BCL2|FLNB	0.0225	GO.0043588
8	GO Process	response to antibiotic	ICAM1|BCL2L1|NQO1|FYN|ANXA1|SDC1|G6PD|BCL2	0.0225	GO.0046677
3	GO Process	glial cell migration	CCL2|MMP14|SUN1	0.0226	GO.0008347
3	GO Process	osteoclast differentiation	CSF1R|ANXA2|EPHA2	0.0226	GO.0030316
7	GO Process	positive regulation of protein complex assembly	ARHGEF5|PLEK|ICAM1|DDB1|MYO1C|TLR4|FSCN1	0.0226	GO.0031334
3	GO Process	substrate adhesion-dependent cell spreading	LAMA5|FERMT3|FN1	0.0226	GO.0034446
5	GO Process	digestive tract development	ITGB4|PDGFRA|KIT|RARB|BCL2	0.0226	GO.0048565
5	GO Process	regulation of smooth muscle cell proliferation	MMP2|THBS1|ITGA2|TGM2|XRCC5	0.0226	GO.0048660
**3**	**GO Process**	**positive regulation of sprouting angiogenesis**	**AKT3|DLL1|ANXA1**	**0.0226**	**GO.1903672**
7	GO Process	calcium ion transport	CCR5|LCK|CAV1|ANXA6|FYN|PPP3CA|CAMK2A	0.0228	GO.0006816
4	GO Process	odontogenesis of dentin-containing tooth	ITGB4|LAMA5|PDGFRA|JAG2	0.0228	GO.0042475
4	GO Process	cellular response to mechanical stimulus	CNN2|ITGA2|FAS|TLR4	0.0228	GO.0071260
7	GO Process	negative regulation of mitotic cell cycle	CCL2|PLK1|BCL2L1|BRSK1|CDC25C|BCL2|TOP2B	0.0232	GO.0045930
2	GO Process	lymphoid progenitor cell differentiation	KIT|BCL2	0.0235	GO.0002320
2	GO Process	detection of mechanical stimulus involved in sensory perception of pain	ITGA2|FYN	0.0235	GO.0050966
4	GO Process	cardiac muscle cell differentiation	PDGFRA|CALR|RARB|MYH10	0.0235	GO.0055007
2	GO Process	hepatocyte differentiation	ITGA2|ANXA1	0.0235	GO.0070365
2	GO Process	positive regulation of podosome assembly	ARHGEF5|FSCN1	0.0235	GO.0071803
2	GO Process	regulation of synapse maturation	ARHGEF15|CAMK2B	0.0235	GO.0090128
6	GO Process	response to ketone	THBS1|CAD|ICAM1|BCL2L1|CALR|CAV1	0.0235	GO.1901654
3	GO Process	response to ischemia	CAV1|BCL2|CAMK2A	0.0236	GO.0002931
7	GO Process	negative regulation of cytokine production	MAP2K5|THBS1|FN1|DLL1|TLR4|ANXA1|NR1H4	0.024	GO.0001818
9	GO Process	regulation of cellular amide metabolic process	GAPDH|HSPB1|THBS1|APP|ITGA2|CALR|SNCA|VIM|NR1H4	0.0242	GO.0034248
5	GO Process	positive regulation of small molecule metabolic process	ENO1|NOS3|SNCA|ANXA1|NR1H4	0.0242	GO.0062013
4	GO Process	positive regulation of smooth muscle cell proliferation	MMP2|THBS1|ITGA2|TGM2	0.0243	GO.0048661
17	GO Process	organophosphate metabolic process	GAPDH|PLEK|ENO1|ARF3|PDGFRA|RORA|CAD|CSF1R|KIT|LCK|SNCA|FYN|EPHA2|G6PD|LDHB|PAICS|ARF1	0.0245	GO.0019637
16	GO Process	immune effector process	RORA|CNN2|ICAM1|APP|KIT|CCT2|RORC|TUBB4B|ANXA2|FYN|MYO1C|DLL1|SPTAN1|XRCC5|BCL2|CDC42	0.0246	GO.0002252
5	GO Process	mesenchymal cell differentiation	PPP3R1|LAMA5|FN1|BCL2|CFL1	0.0248	GO.0048762
3	GO Process	positive regulation of phospholipase C activity	PDGFRA|FLT1|KIT	0.0249	GO.0010863
17	GO Process	negative regulation of cellular protein metabolic process	MAP2K5|GAPDH|HSPB1|THBS1|DNAJB6|PRKCG|APP|PLK1|CALR|SNCA|CAV1|ANXA2|FYN|PTPN1|TLR4|G6PD|PBK	0.0251	GO.0032269
4	GO Process	regulation of muscle adaptation	NOS3|G6PD|PPP3CA|CAMK2B	0.0252	GO.0043502
4	GO Process	regulation of receptor-mediated endocytosis	APP|DAB2|ANXA2|ARF1	0.0252	GO.0048259
6	GO Process	mesenchyme development	PPP3R1|LAMA5|NOS3|FN1|BCL2|CFL1	0.0254	GO.0060485
5	GO Process	stem cell differentiation	LAMA5|KIT|FN1|XRCC5|CFL1	0.0255	GO.0048863
5	GO Process	regulation of dendrite development	NGEF|PPP3CA|CAMK2B|CFL1|CAMK1D	0.0255	GO.0050773
6	GO Process	respiratory system development	PPP3R1|LAMA5|PDGFRA|NOS3|MMP14|RARG	0.0259	GO.0060541
3	GO Process	monocyte chemotaxis	CCL2|FLT1|ANXA1	0.0262	GO.0002548
2	GO Process	regulation of plasminogen activation	ENO1|THBS1	0.0262	GO.0010755
5	GO Process	calcium-mediated signaling	PPP3R1|APP|CCR5|GSTO1|PPP3CA	0.0262	GO.0019722
7	GO Process	regulation of protein stability	GAPDH|CCT2|PLK1|CALR|SNCA|FLNA|BCL2	0.0262	GO.0031647

2	GO Process	negative regulation of monooxygenase activity	SNCA|CAV1	0.0262	GO.0032769
3	GO Process	response to testosterone	THBS1|CAD|CALR	0.0262	GO.0033574
3	GO Process	regulation of cell adhesion mediated by integrin	FERMT3|EPHA2|DPP4	0.0262	GO.0033628
2	GO Process	angiotensin-activated signaling pathway	CAV1|CAMK2A	0.0262	GO.0038166
3	GO Process	macrophage activation	APP|SNCA|TLR4	0.0262	GO.0042116
2	GO Process	regulation of interleukin-8 biosynthetic process	MAP2K5|TLR4	0.0262	GO.0045414
8	GO Process	modulation of chemical synaptic transmission	APP|KIT|BRSK1|SNCA|PPP3CA|CAMK2B|CAMK2A|ARF1	0.0262	GO.0050804
3	GO Process	mammary gland morphogenesis	CSF1R|CAV1|EPHA2	0.0262	GO.0060443
2	GO Process	regulation of dendritic spine maintenance	APP|FYN	0.0262	GO.1902950
2	GO Process	regulation of bile acid metabolic process	KIT|NR1H4	0.0262	GO.1904251
3	GO Process	positive regulation of nitric oxide biosynthetic process	ICAM1|APP|TLR4	0.0274	GO.0045429
3	GO Process	positive regulation of nitric oxide metabolic process	ICAM1|APP|TLR4	0.0274	GO.1904407
12	GO Process	regulation of cytokine production	MAP2K5|GAPDH|HSPB1|THBS1|RORA|CSF1R|FN1|DLL1|TLR4|ANXA1|XRCC5|NR1H4	0.0275	GO.0001817
4	GO Process	regulation of cytokinesis	CDC25B|PLK1|BCL2L1|CDC42	0.0275	GO.0032465
4	GO Process	positive regulation of muscle cell differentiation	KIT|MMP14|BCL2|CDC42	0.0285	GO.0051149
5	GO Process	positive regulation of cell morphogenesis involved in differentiation	CALR|FN1|FLNA|CAMK2B|CDC42	0.0286	GO.0010770
9	GO Process	activation of immune response	LCK|FYN|SFPQ|MYO1C|TLR4|XRCC5|BCL2|CDC42|NR1H4	0.0288	GO.0002253
9	GO Process	regulation of endopeptidase activity	MAP2K5|GAPDH|THBS1|DNAJB6|APP|LCK|SNCA|FYN|FAS	0.0288	GO.0052548
25	GO Process	positive regulation of macromolecule biosynthetic process	MAP2K5|CCL2|PPP3R1|HSPB1|THBS1|RORA|APP|ITGA2|CCT2|ARF4|DAB2|RORC|RARB|NR1I2|SFPQ|RXRG|DLL1|TLR4|RXRB|PSIP1|XRCC5|PPP3CA|RARG|VIM|NR1H4	0.0289	GO.0010557
6	GO Process	positive regulation of transmembrane transport	SNCA|GSTO1|FLNA|G6PD|NR1H4|ABCB1	0.0289	GO.0034764
12	GO Process	cell activation involved in immune response	RORA|CNN2|ICAM1|APP|KIT|CCT2|RORC|TUBB4B|ANXA2|DLL1|SPTAN1|XRCC5	0.029	GO.0002263
2	GO Process	chronic inflammatory response	THBS1|PTGES	0.0292	GO.0002544
2	GO Process	growth plate cartilage development	RARB|RARG	0.0292	GO.0003417
5	GO Process	learning	APP|KIT|ARF4|BRSK1|FYN	0.0292	GO.0007612
2	GO Process	regulation of skeletal muscle adaptation	PPP3CA|CAMK2B	0.0292	GO.0014733
2	GO Process	removal of superoxide radicals	NOS3|NQO1	0.0292	GO.0019430
2	GO Process	positive regulation of actin filament depolymerization	PLEK|CFL1	0.0292	GO.0030836
5	GO Process	regulation of epithelial cell differentiation	ADD1|PRKCH|CAV1|CDH5|DLL1	0.0292	GO.0030856
2	GO Process	positive regulation of pseudopodium assembly	KIT|CDC42	0.0292	GO.0031274
21	GO Process	intracellular transport	EHD4|PPP3R1|HSPB1|APP|KIT|TUBA1A|TUBA1C|ARF4|CALR|SNCA|MYO1C|MYH10|COPA|SPTAN1|DNM1|TBC1D4|PSIP1|PPP3CA|CDC42|SUN1|SPTBN2	0.0292	GO.0046907
2	GO Process	lens fiber cell development	EPHA2|VIM	0.0292	GO.0070307
2	GO Process	positive regulation of phospholipid biosynthetic process	APP|NR1H4	0.0292	GO.0071073
4	GO Process	cellular oxidant detoxification	PXDN|NOS3|NQO1|GSTO1	0.0292	GO.0098869
5	GO Process	response to glucocorticoid	CAD|ICAM1|ANXA1|SDC1|BCL2	0.0296	GO.0051384
7	GO Process	positive regulation of innate immune response	MMP2|CAV1|FYN|SFPQ|TLR4|XRCC5|NR1H4	0.0297	GO.0045089
4	GO Process	non-canonical Wnt signaling pathway	PPP3R1|PPP3CA|CAMK2A|CDC42	0.03	GO.0035567
5	GO Process	positive regulation of cysteine-type endopeptidase activity	APP|LCK|SNCA|FYN|FAS	0.0304	GO.2001056
10	GO Process	regulation of GTPase activity	ARHGEF5|CCL2|NGEF|ARHGAP31|ICAM1|RGS12|EPHA2|ARHGEF15|ARHGAP30|TBC1D4	0.0314	GO.0043087
9	GO Process	glycerolipid metabolic process	PLEK|ARF3|PDGFRA|CSF1R|KIT|LCK|CAV1|FYN|ARF1	0.0316	GO.0046486
9	GO Process	phospholipid metabolic process	PLEK|ARF3|PDGFRA|CSF1R|KIT|LCK|SNCA|FYN|ARF1	0.032	GO.0006644
5	GO Process	morphogenesis of embryonic epithelium	LAMA5|JAG2|SDC4|RARG|CFL1	0.032	GO.0016331
2	GO Process	positive regulation of receptor recycling	SNCA|ANXA2	0.0323	GO.0001921
12	GO Process	gamete generation	CDC25B|BCL2L2|KIT|NOS3|PLK1|BCL2L1|CALR|CDC25C|JAG2|BCL2|SEPT7|SUN1	0.0323	GO.0007276
9	GO Process	epidermis development	PLOD1|LAMA5|JAG2|EPHA2|DLL1|CLIC4|ANXA1|BCL2|FLNB	0.0323	GO.0008544
2	GO Process	vitamin D metabolic process	CYP24A1|CYP2R1	0.0323	GO.0042359
2	GO Process	protein kinase C signaling	PLEK|PRKCH	0.0323	GO.0070528
2	GO Process	positive regulation of exosomal secretion	SDC4|SDC1	0.0323	GO.1903543
2	GO Process	negative regulation of cardiac muscle cell differentiation	DLL1|G6PD	0.0323	GO.2000726
5	GO Process	positive regulation of ion transmembrane transport	SNCA|GSTO1|FLNA|G6PD|ABCB1	0.0325	GO.0034767
5	GO Process	positive regulation of NF-kappaB transcription factor activity	ICAM1|APP|PRKCH|TLR4|CAMK2A	0.0325	GO.0051092
11	GO Process	response to bacterium	CCL2|ICAM1|APP|CCR5|NOS3|SNCA|CAV1|PTGES|FAS|TLR4|NR1H4	0.0328	GO.0009617
**3**	**GO Process**	**sprouting angiogenesis**	**THBS1|DLL1|MMRN2**	**0.0329**	**GO.0002040**
32	GO Process	negative regulation of cellular metabolic process	MAP2K5|GAPDH|PLEK|ENO1|HSPB1|THBS1|DNAJB6|PRKCG|APP|PLK1|BCL2L1|DAB2|CALR|RORC|RARB|NR1I2|SNCA|CAV1|ANXA2|FYN|SFPQ|FLNA|PTPN1|TLR4|XRCC5|G6PD|PPP3CA|BCL2|DYRK1A|RARG|PBK|NR1H4	0.0329	GO.0031324
4	GO Process	positive regulation of protein binding	ADD1|APP|CAV1|ANXA2	0.0329	GO.0032092
3	GO Process	regulation of synaptic vesicle cycle	BRSK1|SNCA|DNM1	0.0329	GO.0098693
3	GO Process	regulation of endothelial cell apoptotic process	CCL2|THBS1|ICAM1	0.0329	GO.2000351
9	GO Process	positive regulation of GTPase activity	ARHGEF5|CCL2|ARHGAP31|ICAM1|RGS12|EPHA2|ARHGEF15|ARHGAP30|TBC1D4	0.0333	GO.0043547
6	GO Process	regulation of symbiosis, encompassing mutualism through parasitism	GAPDH|DDB1|CAV1|ANXA2|BCL2|CFL1	0.0337	GO.0043903
4	GO Process	columnar/cuboidal epithelial cell differentiation	JAG2|RARB|DLL1|RARG	0.0338	GO.0002065
4	GO Process	positive regulation of leukocyte chemotaxis	THBS1|APP|CALR|CAMK1D	0.0338	GO.0002690
4	GO Process	regulation of Notch signaling pathway	KIT|MMP14|JAG2|DLL1	0.0338	GO.0008593
4	GO Process	glial cell development	PPP3R1|APP|DLL1|VIM	0.0338	GO.0021782
4	GO Process	negative regulation of cysteine-type endopeptidase activity involved in apoptotic process	MAP2K5|THBS1|DNAJB6|SNCA	0.0338	GO.0043154
5	GO Process	eye morphogenesis	COL8A1|RARB|EPHA2|DLL1|RARG	0.0346	GO.0048592
2	GO Process	regulation of extracellular matrix disassembly	DPP4|FSCN1	0.0353	GO.0010715
2	GO Process	regulation of inositol phosphate biosynthetic process	PLEK|SNCA	0.0353	GO.0010919
2	GO Process	negative regulation of myeloid cell apoptotic process	CCR5|BCL2	0.0353	GO.0033033
2	GO Process	negative regulation of dendrite morphogenesis	NGEF|PPP3CA	0.0353	GO.0050774
2	GO Process	negative regulation of focal adhesion assembly	THBS1|MMP14	0.0353	GO.0051895
2	GO Process	positive regulation of macrophage migration	THBS1|MMP14	0.0353	GO.1905523
3	GO Process	negative regulation of blood coagulation	PDGFRA|NOS3|ANXA2	0.0356	GO.0030195
3	GO Process	peptidyl-tyrosine autophosphorylation	LCK|FYN|DYRK1A	0.0356	GO.0038083
3	GO Process	positive regulation of Notch signaling pathway	KIT|JAG2|DLL1	0.0356	GO.0045747
3	GO Process	face development	MMP2|PDGFRA|RARG	0.0356	GO.0060324

4	GO Process	extrinsic apoptotic signaling pathway	BCL2L2|BCL2L1|FAS|BCL2	0.0356	GO.0097191
4	GO Process	positive regulation of plasma membrane bounded cell projection assembly	KIT|FSCN1|SEPT7|CDC42	0.0356	GO.0120034
5	GO Process	response to interleukin-1	CCL2|RORA|ICAM1|SNCA|ANXA1	0.0358	GO.0070555
6	GO Process	regulation of stress-activated MAPK cascade	ARHGEF5|APP|MAP4K4|FAS|PTPN1|PBK	0.0366	GO.0032872
7	GO Process	positive regulation of cell cycle process	CDC25B|APP|CDC25A|CDC25C|SFPQ|ANXA1|CDC42	0.0366	GO.0090068
3	GO Process	endochondral bone morphogenesis	MMP14|RARB|RARG	0.0372	GO.0060350
6	GO Process	receptor-mediated endocytosis	DAB2|CALR|SNCA|CAV1|DLL1|DNM1	0.0373	GO.0006898
4	GO Process	ciliary basal body-plasma membrane docking	TUBB4A|PLK1|TUBA1A|TUBB4B	0.0377	GO.0097711
2	GO Process	regulation of cellular amino acid metabolic process	NQO1|NR1H4	0.0386	GO.0006521
2	GO Process	regulation of developmental pigmentation	KIT|BCL2	0.0386	GO.0048070
2	GO Process	positive regulation of nitric-oxide synthase biosynthetic process	CCL2|TLR4	0.0386	GO.0051770
2	GO Process	cardiac myofibril assembly	PDGFRA|MYH10	0.0386	GO.0055003
2	GO Process	branching involved in salivary gland morphogenesis	LAMA5|TGM2	0.0386	GO.0060445
2	GO Process	epithelial cell fate commitment	JAG2|DLL1	0.0386	GO.0072148
2	GO Process	regulation of vascular endothelial growth factor signaling pathway	MYO1C|DLL1	0.0386	GO.1900746
3	GO Process	mitotic cytokinesis	PLK1|MYH10|CFL1	0.0387	GO.0000281
4	GO Process	regulation of cytokine biosynthetic process	MAP2K5|HSPB1|THBS1|TLR4	0.0387	GO.0042035
3	GO Process	positive regulation of receptor-mediated endocytosis	APP|DAB2|ANXA2	0.0387	GO.0048260
4	GO Process	regulation of striated muscle cell differentiation	MMP14|DLL1|G6PD|BCL2	0.0387	GO.0051153
8	GO Process	activation of protein kinase activity	MAP2K5|THBS1|APP|KIT|MAP4K4|SLK|PTPN1|TLR4	0.0389	GO.0032147
9	GO Process	organic hydroxy compound metabolic process	CYP24A1|APP|NPC1L1|CYP2R1|SNCA|G6PD|LDHB|BCL2|NR1H4	0.0389	GO.1901615
8	GO Process	negative regulation of proteolysis	MAP2K5|GAPDH|THBS1|DNAJB6|PRKCG|APP|SNCA|PBK	0.04	GO.0045861
6	GO Process	protein folding	GNB4|HSPB1|DNAJB6|CCT2|CALR|PDIA6	0.0406	GO.0006457
5	GO Process	mitotic cell cycle checkpoint	PLK1|BCL2L1|BRSK1|CDC25C|TOP2B	0.0409	GO.0007093
4	GO Process	regulation of circadian rhythm	RORA|PRKCG|RORC|SFPQ	0.0409	GO.0042752
5	GO Process	cellular response to radiation	DDB1|BCL2L1|CDC25A|XRCC5|PBK	0.0409	GO.0071478
25	GO Process	system process	PRKCG|ICAM1|APP|KIT|NPC1L1|ITGA2|NOS3|ARF4|BRSK1|JAG2|SNCA|CAV1|TPM4|FYN|MYH10|DLL1|COPA|TPM3|DNM1|TLR4|TPM2|PPP3CA|LTB4R|BCL2|VIM	0.0412	GO.0003008
6	GO Process	ossification	CYP24A1|MMP2|MMP14|EPHA2|BCL2|SUN1	0.0413	GO.0001503
2	GO Process	arginine metabolic process	CAD|NOS3	0.0419	GO.0006525
2	GO Process	intrinsic apoptotic signaling pathway in response to oxidative stress	MELK|BCL2	0.0419	GO.0008631
2	GO Process	regulation of hydrogen peroxide metabolic process	SNCA|FYN	0.0419	GO.0010310
3	GO Process	myotube differentiation	MYH9|CAPN2|PPP3CA	0.0419	GO.0014902
2	GO Process	melanocyte differentiation	KIT|BCL2	0.0419	GO.0030318
2	GO Process	cell adhesion mediated by integrin	ICAM1|ITGA2	0.0419	GO.0033627
2	GO Process	toll-like receptor 4 signaling pathway	TLR4|NR1H4	0.0419	GO.0034142
2	GO Process	establishment of apical/basal cell polarity	FSCN1|CDC42	0.0419	GO.0035089
3	GO Process	IRE1-mediated unfolded protein response	ADD1|PTPN1|PDIA6	0.0419	GO.0036498
4	GO Process	cellular transition metal ion homeostasis	MT2A|APP|LCK|ARF1	0.0419	GO.0046916
2	GO Process	regulation of transcription involved in cell fate commitment	RORA|RORC	0.0419	GO.0060850
7	GO Process	multicellular organismal homeostasis	AKT3|ADD1|NOS3|TENC1|DLL1|TLR4|BCL2	0.042	GO.0048871
5	GO Process	tissue homeostasis	AKT3|ADD1|NOS3|TLR4|BCL2	0.0422	GO.0001894
4	GO Process	peptidyl-tyrosine dephosphorylation	CDC25B|CDC25A|CDC25C|PTPN1	0.043	GO.0035335
15	GO Process	oxidation-reduction process	PLOD1|CYP24A1|GAPDH|ENO1|PXDN|PLOD2|NOS3|NQO1|CYP2R1|SNCA|GSTO1|PIR|G6PD|LDHB|PDIA6	0.0449	GO.0055114
7	GO Process	positive regulation of cellular protein localization	CCT2|PLK1|PRKCH|FYN|EPHA2|MYO1C|BCL2	0.0449	GO.1903829
5	GO Process	intrinsic apoptotic signaling pathway	BCL2L2|MELK|BCL2L1|EPHA2|BCL2	0.0452	GO.0097193
3	GO Process	negative regulation of cytokine-mediated signaling pathway	PXDN|CAV1|NR1H4	0.0454	GO.0001960
4	GO Process	myeloid cell homeostasis	ADD1|KIT|ANXA1|G6PD	0.0454	GO.0002262
2	GO Process	nuclear migration	MYH10|SUN1	0.0454	GO.0007097
2	GO Process	negative regulation of calcium ion transport into cytosol	GSTO1|BCL2	0.0454	GO.0010523
2	GO Process	negative regulation of platelet activation	PDGFRA|NOS3	0.0454	GO.0010544
2	GO Process	positive regulation of lamellipodium assembly	FSCN1|CDC42	0.0454	GO.0010592
2	GO Process	response to magnesium ion	THBS1|SNCA	0.0454	GO.0032026
2	GO Process	negative regulation of chemokine production	MAP2K5|NR1H4	0.0454	GO.0032682
3	GO Process	collagen metabolic process	MMP2|MMP14|TENC1	0.0454	GO.0032963
2	GO Process	cellular response to sterol	RORA|RORC	0.0454	GO.0036315
21	GO Process	establishment of protein localization	MYH9|PPP3R1|PLEK|HSPB1|ARF3|EXOC6B|ITGA2|PLK1|ARF4|DAB2|CALR|MYO1C|COPA|FLNA|TLR4|ANXA1|TBC1D4|PPP3CA|ARF1|NR1H4|APBA2	0.0454	GO.0045184
2	GO Process	nucleobase biosynthetic process	CAD|PAICS	0.0454	GO.0046112
3	GO Process	positive regulation of fibroblast proliferation	PDGFRA|ANXA2|FN1	0.0454	GO.0048146
3	GO Process	dendrite morphogenesis	FYN|CAMK2A|CDC42	0.0454	GO.0048813
2	GO Process	regulation of synaptic vesicle recycling	SNCA|DNM1	0.0454	GO.1903421
2	GO Process	positive regulation of endothelial cell apoptotic process	CCL2|THBS1	0.0454	GO.2000353
5	GO Process	regulation of cell size	PLEK|AKT3|ADD1|FN1|RARG	0.0455	GO.0008361
5	GO Process	positive regulation of cell growth	MAP2K5|MMP14|FN1|BCL2|CDC42	0.0455	GO.0030307
10	GO Process	protein complex oligomerization	EHD4|VWF|APP|CAV1|ANXA2|ANXA6|RXRG|TGM2|DNM1|SEPT7	0.0455	GO.0051259
7	GO Process	cellular response to peptide	APP|CAV1|FYN|PTPN1|TLR4|TBC1D4|CAMK2A	0.0455	GO.1901653
4	GO Process	phosphatidylinositol phosphorylation	PDGFRA|KIT|LCK|FYN	0.046	GO.0046854
5	GO Process	negative regulation of mitotic cell cycle phase transition	CCL2|PLK1|BRSK1|CDC25C|BCL2	0.0463	GO.1901991
8	GO Process	response to peptide hormone	CAD|ICAM1|CAV1|FYN|PTPN1|ANXA1|TBC1D4|CAMK2A	0.0464	GO.0043434
7	GO Process	muscle system process	NOS3|TPM4|TPM3|TPM2|PPP3CA|LTB4R|VIM	0.0467	GO.0003012
3	GO Process	response to gamma radiation	BCL2L1|XRCC5|BCL2	0.0467	GO.0010332
3	GO Process	positive regulation of calcium ion transport into cytosol	SNCA|CAV1|GSTO1	0.0467	GO.0010524
3	GO Process	monosaccharide biosynthetic process	GAPDH|ENO1|G6PD	0.0467	GO.0046364
4	GO Process	lymphocyte activation involved in immune response	RORA|ICAM1|RORC|DLL1	0.0471	GO.0002285
4	GO Process	digestion	NPC1L1|COPA|TLR4|PIR	0.0471	GO.0007586
9	GO Process	negative regulation of hydrolase activity	MAP2K5|GAPDH|THBS1|DNAJB6|APP|NOS3|SNCA|CNN3|ANXA1	0.0471	GO.0051346
5	GO Process	meiotic cell cycle process	MYH9|CDC25B|PLK1|SUN1|TOP2B	0.0471	GO.1903046
8	GO Process	positive regulation of secretion by cell	GAPDH|CSF1R|SNCA|MYH10|SDC4|TLR4|SDC1|NR1H4	0.0474	GO.1903532

5	GO Process	lung development	PPP3R1|LAMA5|PDGFRA|NOS3|MMP14	0.0481	GO.0030324
2	GO Process	activation of MAPKKK activity	APP|MAP4K4	0.0482	GO.0000185
2	GO Process	dendritic cell chemotaxis	ARHGEF5|CCR5	0.0482	GO.0002407
2	GO Process	myoblast fusion	MYH9|CAPN2	0.0482	GO.0007520
3	GO Process	receptor internalization	SNCA|CAV1|DNM1	0.0482	GO.0031623
2	GO Process	negative regulation of chondrocyte differentiation	RARB|RARG	0.0482	GO.0032331
2	GO Process	negative regulation of interleukin-8 production	MAP2K5|ANXA1	0.0482	GO.0032717
3	GO Process	regulation of monooxygenase activity	NOS3|SNCA|CAV1	0.0482	GO.0032768
4	GO Process	positive regulation of peptidyl-serine phosphorylation	APP|SNCA|CAV1|BCL2	0.0482	GO.0033138
2	GO Process	positive regulation of circadian rhythm	RORA|RORC	0.0482	GO.0042753
2	GO Process	ventricular cardiac muscle cell differentiation	RARB|MYH10	0.0482	GO.0055012
7	GO Process	regulation of cation transmembrane transport	APP|SNCA|CAV1|FYN|GSTO1|FLNA|G6PD	0.0482	GO.1904062
5	GO Process	negative regulation of cell projection organization	NGEF|MAP4K4|FYN|PPP3CA|VIM	0.0486	GO.0031345
5	GO Process	sensory perception of mechanical stimulus	ICAM1|KIT|ITGA2|FYN|DNM1	0.0486	GO.0050954
4	GO Process	meiosis I	CDC25B|PLK1|SUN1|TOP2B	0.0492	GO.0007127
24	GO Process	positive regulation of nucleobase-containing compound metabolic process	MAP2K5|PPP3R1|ENO1|RORA|PRKCG|APP|NOS3|CCT2|ARF4|DAB2|RORC|RARB|NR1I2|SFPQ|RXRG|DLL1|TLR4|RXRB|PSIP1|XRCC5|PPP3CA|DYRK1A|RARG|NR1H4	0.0492	GO.0045935
5	GO Process	protein localization to nucleus	PPP3R1|PLK1|CALR|PPP3CA|SUN1	0.0496	GO.0034504
5	GO Process	cellular response to oxygen levels	RORA|ICAM1|CAV1|FAS|BCL2	0.0496	GO.0071453
21	InterPro Domains	Protein kinase, ATP binding site	MAP2K5|PDGFRA|PRKCG|AKT3|FLT1|CSF1R|KIT|MELK|PLK1|BRSK1|PRKCH|LCK|MAP4K4|FYN|EPHA2|SLK|CAMK2B|CAMK2A|DYRK1A|WEE1|CAMK1D	1.10E-08	IPR017441
22	InterPro Domains	Protein kinase domain	MAP2K5|PDGFRA|PRKCG|AKT3|FLT1|CSF1R|KIT|MELK|PLK1|BRSK1|PRKCH|LCK|MAP4K4|FYN|EPHA2|SLK|CAMK2B|CAMK2A|DYRK1A|WEE1|PBK|CAMK1D	6.60E-08	IPR000719
22	InterPro Domains	Protein kinase-like domain superfamily	MAP2K5|PDGFRA|PRKCG|AKT3|FLT1|CSF1R|KIT|MELK|PLK1|BRSK1|PRKCH|LCK|MAP4K4|FYN|EPHA2|SLK|CAMK2B|CAMK2A|DYRK1A|WEE1|PBK|CAMK1D	2.09E-07	IPR011009
8	InterPro Domains	Nuclear hormone receptor, ligand-binding domain	RORA|RORC|RARB|NR1I2|RXRG|RXRB|RARG|NR1H4	1.95E-06	IPR000536
8	InterPro Domains	Zinc finger, nuclear hormone receptor-type	RORA|RORC|RARB|NR1I2|RXRG|RXRB|RARG|NR1H4	1.95E-06	IPR001628
8	InterPro Domains	Nuclear hormone receptor	RORA|RORC|RARB|NR1I2|RXRG|RXRB|RARG|NR1H4	1.95E-06	IPR001723
18	InterPro Domains	PH-like domain superfamily	ARHGEF5|PLEK|AKT3|NGEF|FERMT3|APP|DAB2|TENC1|RGS12|DOK4|ARHGEF26|ARHGEF15|DNM1|TBC1D4|SPTBN2|NUMB|APBA2|TLN2	1.95E-06	IPR011993
8	InterPro Domains	Nuclear hormone receptor-like domain superfamily	RORA|RORC|RARB|NR1I2|RXRG|RXRB|RARG|NR1H4	1.95E-06	IPR035500
8	InterPro Domains	Zinc finger, NHR/GATA-type	RORA|RORC|RARB|NR1I2|RXRG|RXRB|RARG|NR1H4	3.43E-06	IPR013088
15	InterPro Domains	Serine/threonine-protein kinase, active site	MAP2K5|PRKCG|AKT3|MELK|PLK1|BRSK1|PRKCH|MAP4K4|SLK|CAMK2B|CAMK2A|DYRK1A|WEE1|PBK|CAMK1D	5.05E-06	IPR008271
4	InterPro Domains	Tyrosine-protein kinase, receptor class III, conserved site	PDGFRA|FLT1|CSF1R|KIT	1.10E-04	IPR001824
6	InterPro Domains	PTB/PI domain	DAB2|TENC1|RGS12|TBC1D4|NUMB|APBA2	1.10E-04	IPR006020
7	InterPro Domains	Tyrosine-protein kinase, catalytic domain	PDGFRA|FLT1|CSF1R|KIT|LCK|FYN|EPHA2	3.10E-04	IPR020635
3	InterPro Domains	M-phase inducer phosphatase	CDC25B|CDC25A|CDC25C	3.90E-04	IPR000751
4	InterPro Domains	Annexin	ANXA5|ANXA2|ANXA6|ANXA1	3.90E-04	IPR001464
3	InterPro Domains	Apoptosis regulator, Bcl-2 protein, BH4	BCL2L2|BCL2L1|BCL2	3.90E-04	IPR003093
4	InterPro Domains	Annexin repeat, conserved site	ANXA5|ANXA2|ANXA6|ANXA1	3.90E-04	IPR018252
4	InterPro Domains	Annexin repeat	ANXA5|ANXA2|ANXA6|ANXA1	3.90E-04	IPR018502
3	InterPro Domains	Apoptosis regulator, Bcl-2, BH4 motif, conserved site	BCL2L2|BCL2L1|BCL2	3.90E-04	IPR020731
4	InterPro Domains	Annexin superfamily	ANXA5|ANXA2|ANXA6|ANXA1	3.90E-04	IPR037104
3	InterPro Domains	Tropomyosin	TPM4|TPM3|TPM2	5.00E-04	IPR000533
7	InterPro Domains	Tyrosine-protein kinase, active site	PDGFRA|FLT1|CSF1R|KIT|LCK|FYN|EPHA2	5.60E-04	IPR008266
4	InterPro Domains	Tubulin/FtsZ, C-terminal domain superfamily	TUBB4A|TUBA1A|TUBA1C|TUBB4B	0.0012	IPR037103
4	InterPro Domains	Tubulin/FtsZ, 2-layer sandwich domain	TUBB4A|TUBA1A|TUBA1C|TUBB4B	0.0013	IPR018316
4	InterPro Domains	Tubulin	TUBB4A|TUBA1A|TUBA1C|TUBB4B	0.0015	IPR000217
4	InterPro Domains	Actinin-type actin-binding domain, conserved site	FLNC|FLNA|FLNB|SPTBN2	0.0015	IPR001589
6	InterPro Domains	Calponin homology domain	CNN2|FLNC|FLNA|CNN3|FLNB|SPTBN2	0.0015	IPR001715
4	InterPro Domains	Tubulin/FtsZ, GTPase domain	TUBB4A|TUBA1A|TUBA1C|TUBB4B	0.0015	IPR003008
4	InterPro Domains	Tubulin/FtsZ, C-terminal	TUBB4A|TUBA1A|TUBA1C|TUBB4B	0.0015	IPR008280
4	InterPro Domains	Tubulin, conserved site	TUBB4A|TUBA1A|TUBA1C|TUBB4B	0.0015	IPR017975
3	InterPro Domains	Apoptosis regulator, Bcl-2, BH1 motif, conserved site	BCL2L2|BCL2L1|BCL2	0.0015	IPR020717
3	InterPro Domains	Apoptosis regulator, Bcl-2, BH2 motif, conserved site	BCL2L2|BCL2L1|BCL2	0.0015	IPR020726
4	InterPro Domains	Tubulin, C-terminal	TUBB4A|TUBA1A|TUBA1C|TUBB4B	0.0015	IPR023123
4	InterPro Domains	Tubulin/FtsZ, GTPase domain superfamily	TUBB4A|TUBA1A|TUBA1C|TUBB4B	0.0015	IPR036525
6	InterPro Domains	CH domain superfamily	CNN2|FLNC|FLNA|CNN3|FLNB|SPTBN2	0.0016	IPR036872
3	InterPro Domains	Filamin/ABP280 repeat	FLNC|FLNA|FLNB	0.0018	IPR001298
7	InterPro Domains	Serine-threonine/tyrosine-protein kinase, catalytic domain	PDGFRA|FLT1|CSF1R|KIT|LCK|FYN|EPHA2	0.0022	IPR001245
3	InterPro Domains	Blc2 family	BCL2L2|BCL2L1|BCL2	0.0022	IPR026298
3	InterPro Domains	Bcl2-like	BCL2L2|BCL2L1|BCL2	0.0027	IPR002475
3	InterPro Domains	Filamin/ABP280 repeat-like	FLNC|FLNA|FLNB	0.0027	IPR017868
3	InterPro Domains	Blc2-like superfamily	BCL2L2|BCL2L1|BCL2	0.0046	IPR036834
4	InterPro Domains	VWFC domain	PXDN|THBS1|VWF|JAG2	0.0052	IPR001007
4	InterPro Domains	Myosin head, motor domain	MYH9|MYO1C|MYH10|MYO1B	0.0052	IPR001609
2	InterPro Domains	Apoptosis regulator, Bcl-2/ BclX	BCL2L1|BCL2	0.0052	IPR004725
9	InterPro Domains	Pleckstrin homology domain	ARHGEF5|PLEK|AKT3|NGEF|FERMT3|DOK4|ARHGEF26|DNM1|SPTBN2	0.0064	IPR001849
2	InterPro Domains	Procollagen-lysine 5-dioxygenase, conserved site	PLOD1|PLOD2	0.0079	IPR001006
2	InterPro Domains	Nuclear receptor ROR	RORA|RORC	0.0079	IPR003079
2	InterPro Domains	Amyloidogenic glycoprotein, extracellular	APLP1|APP	0.0079	IPR008154
2	InterPro Domains	Amyloidogenic glycoprotein	APLP1|APP	0.0079	IPR008155
2	InterPro Domains	Amyloidogenic glycoprotein, copper-binding	APLP1|APP	0.0079	IPR011178
2	InterPro Domains	Amyloidogenic glycoprotein, heparin-binding	APLP1|APP	0.0079	IPR015849
2	InterPro Domains	Beta-amyloid precursor protein C-terminal	APLP1|APP	0.0079	IPR019543
2	InterPro Domains	Amyloidogenic glycoprotein, extracellular domain conserved site	APLP1|APP	0.0079	IPR019744
2	InterPro Domains	Amyloidogenic glycoprotein, intracellular domain, conserved site	APLP1|APP	0.0079	IPR019745
2	InterPro Domains	Amyloidogenic glycoprotein, E2 domain	APLP1|APP	0.0079	IPR024329
2	InterPro Domains	E2 domain superfamily	APLP1|APP	0.0079	IPR036176
2	InterPro Domains	Amyloidogenic glycoprotein, heparin-binding domain superfamily	APLP1|APP	0.0079	IPR036454

2	InterPro Domains	Amyloidogenic glycoprotein, copper-binding domain superfamily	APLP1|APP	0.0079	IPR036669
2	InterPro Domains	Retinoid X receptor/HNF4	RXRG|RXRB	0.0092	IPR000003
2	InterPro Domains	Syndecan	SDC4|SDC1	0.0092	IPR001050
2	InterPro Domains	Delta/Serrate/lag-2 (DSL) protein	JAG2|DLL1	0.0092	IPR001774
2	InterPro Domains	Calponin/LIMCH1	CNN2|CNN3	0.0092	IPR001997
2	InterPro Domains	Retinoic acid receptor	RARB|RARG	0.0092	IPR003078
2	InterPro Domains	Notch ligand, N-terminal domain	JAG2|DLL1	0.0092	IPR011651
2	InterPro Domains	Calcium/calmodulin-dependent protein kinase II, association-domain	CAMK2B|CAMK2A	0.0092	IPR013543
2	InterPro Domains	Cavin family	SDPR|PTRF	0.0092	IPR026752
2	InterPro Domains	Syndecan, conserved site	SDC4|SDC1	0.0092	IPR030479
2	InterPro Domains	Calcium/calmodulin-dependent protein kinase type II	CAMK2B|CAMK2A	0.0092	IPR039071
3	InterPro Domains	Rhodanese-like domain	CDC25B|CDC25A|CDC25C	0.01	IPR001763
3	InterPro Domains	Rhodanese-like domain superfamily	CDC25B|CDC25A|CDC25C	0.01	IPR036873
2	InterPro Domains	Laminin alpha, domain I	LAMA4|LAMA5	0.0107	IPR009254
2	InterPro Domains	Laminin domain II	LAMA4|LAMA5	0.0107	IPR010307
2	InterPro Domains	Calponin repeat	CNN2|CNN3	0.0138	IPR000557
3	InterPro Domains	Protein kinase, C-terminal	PRKCG|AKT3|PRKCH	0.0171	IPR017892
2	InterPro Domains	Syndecan/Neurexin domain	SDC4|SDC1	0.0171	IPR027789
7	InterPro Domains	SH3-like domain superfamily	ARHGEF5|NGEF|TJP1|LCK|FYN|ARHGEF26|SPTAN1	0.0171	IPR036028
7	InterPro Domains	SH3 domain	ARHGEF5|NGEF|TJP1|LCK|FYN|ARHGEF26|SPTAN1	0.0207	IPR001452
2	InterPro Domains	Alpha tubulin	TUBA1A|TUBA1C	0.0207	IPR002452
3	InterPro Domains	Small GTPase superfamily, ARF/SAR type	ARF3|ARF4|ARF1	0.0207	IPR006689
2	InterPro Domains	Class I myosin tail homology domain	MYO1C|MYO1B	0.0207	IPR010926
2	InterPro Domains	Apoptosis regulator, Bcl-2, BH3 motif, conserved site	BCL2L1|BCL2	0.0207	IPR020728
2	InterPro Domains	Class I myosin, motor domain	MYO1C|MYO1B	0.0207	IPR036072
2	InterPro Domains	PGBD superfamily	MMP2|MMP14	0.0207	IPR036366
4	InterPro Domains	Dbl homology (DH) domain	ARHGEF5|NGEF|ARHGEF26|ARHGEF15	0.0227	IPR000219
4	InterPro Domains	Dbl homology (DH) domain superfamily	ARHGEF5|NGEF|ARHGEF26|ARHGEF15	0.0236	IPR035899
6	InterPro Domains	EF-Hand 1, calcium-binding site	RCN1|EHD4|PPP3R1|CAPN2|SPTAN1|PRKCSH	0.0237	IPR018247
2	InterPro Domains	Beta tubulin, autoregulation binding site	TUBB4A|TUBB4B	0.0266	IPR013838
2	InterPro Domains	Flavoprotein-like superfamily	NOS3|NQO1	0.0266	IPR029039
2	InterPro Domains	Beta tubulin	TUBB4A|TUBB4B	0.0306	IPR002453
2	InterPro Domains	Smooth muscle protein/calponin	CNN2|CNN3	0.0306	IPR003096
4	InterPro Domains	Kinesin motor domain superfamily	MYH9|MYO1C|MYH10|MYO1B	0.0306	IPR036961
4	InterPro Domains	IQ motif, EF-hand binding site	MYH9|MYO1C|MYH10|MYO1B	0.0324	IPR000048
13	InterPro Domains	Immunoglobulin-like fold	ITGB4|PXDN|PDGFRA|ICAM1|FLT1|CSF1R|KIT|FLNC|FN1|EPHA2|TGM2|FLNA|FLNB	0.0424	IPR013783
2	InterPro Domains	Neurexin/syndecan/glycophorin C	SDC4|SDC1	0.0439	IPR003585
2	InterPro Domains	Dynamin-type guanine nucleotide-binding (G) domain	EHD4|DNM1	0.0439	IPR030381
2	InterPro Domains	Peptidase M10A, cysteine switch, zinc binding site	MMP2|MMP14	0.0484	IPR021158
2	InterPro Domains	Dynamin superfamily	EHD4|DNM1	0.0484	IPR022812
20	KEGG Pathways	Focal adhesion	ITGB4|LAMA4|LAMA5|PDGFRA|THBS1|VWF|PRKCG|AKT3|FLT1|CAPN2|ITGA2|ZYX|FLNC|CAV1|FYN|FLNA|BCL2|CDC42|FLNB|TLN2	3.60E-13	hsa04510
24	KEGG Pathways	Pathways in cancer	MMP2|LAMA4|GNB4|LAMA5|PDGFRA|PRKCG|AKT3|CSF1R|KIT|ITGA2|BCL2L1|NQO1|JAG2|RARB|FAS|RXRG|DLL1|TPM3|GSTO1|RXRB|CAMK2B|BCL2|CAMK2A|CDC42	2.16E-09	hsa05200
16	KEGG Pathways	Proteoglycans in cancer	MMP2|THBS1|PRKCG|AKT3|ITGA2|FLNC|CAV1|FAS|FLNA|SDC4|TLR4|SDC1|CAMK2B|CAMK2A|CDC42|FLNB	2.16E-09	hsa05205
18	KEGG Pathways	MAPK signaling pathway	MAP2K5|PPP3R1|CDC25B|HSPB1|PDGFRA|PRKCG|AKT3|FLT1|CSF1R|KIT|FLNC|MAP4K4|FAS|EPHA2|FLNA|PPP3CA|CDC42|FLNB	6.49E-09	hsa04010
13	KEGG Pathways	Fluid shear stress and atherosclerosis	MAP2K5|MMP2|CCL2|AKT3|ICAM1|NOS3|NQO1|CAV1|CDH5|GSTO1|SDC4|SDC1|BCL2	1.17E-08	hsa05418
17	KEGG Pathways	PI3K-Akt signaling pathway	ITGB4|LAMA4|GNB4|LAMA5|PDGFRA|THBS1|VWF|AKT3|FLT1|CSF1R|KIT|ITGA2|NOS3|BCL2L1|EPHA2|TLR4|BCL2	3.70E-07	hsa04151
12	KEGG Pathways	MicroRNAs in cancer	CYP24A1|CDC25B|BCL2L2|PDGFRA|THBS1|PRKCG|CDC25A|CDC25C|FSCN1|BCL2|VIM|ABCB1	3.70E-07	hsa05206
10	KEGG Pathways	HIF-1 signaling pathway	GAPDH|ENO1|PRKCG|AKT3|FLT1|NOS3|TLR4|CAMK2B|BCL2|CAMK2A	6.15E-07	hsa04066
9	KEGG Pathways	Small cell lung cancer	LAMA4|LAMA5|AKT3|ITGA2|BCL2L1|RARB|RXRG|RXRB|BCL2	3.93E-06	hsa05222
7	KEGG Pathways	Pathogenic Escherichia coli infection	TUBB4A|TUBA1A|TUBA1C|TUBB4B|FYN|TLR4|CDC42	1.38E-05	hsa05130
8	KEGG Pathways	ECM-receptor interaction	ITGB4|LAMA4|LAMA5|THBS1|VWF|ITGA2|SDC4|SDC1	1.49E-05	hsa04512
8	KEGG Pathways	Salmonella infection	MYH9|TJP1|FLNC|MYH10|FLNA|TLR4|CDC42|FLNB	1.76E-05	hsa05132
7	KEGG Pathways	VEGF signaling pathway	PPP3R1|HSPB1|PRKCG|AKT3|NOS3|PPP3CA|CDC42	2.05E-05	hsa04370
8	KEGG Pathways	Gap junction	MAP2K5|PDGFRA|PRKCG|TUBB4A|TJP1|TUBA1A|TUBA1C|TUBB4B	2.05E-05	hsa04540
10	KEGG Pathways	Rap1 signaling pathway	PDGFRA|THBS1|PRKCG|AKT3|FLT1|CSF1R|KIT|EPHA2|CDC42|TLN2	1.70E-04	hsa04015
9	KEGG Pathways	Axon guidance	PPP3R1|NGEF|FYN|EPHA2|PPP3CA|CAMK2B|CAMK2A|CDC42|CFL1	2.90E-04	hsa04360
8	KEGG Pathways	Apoptosis	AKT3|CAPN2|TUBA1A|TUBA1C|BCL2L1|FAS|SPTAN1|BCL2	3.30E-04	hsa04210
7	KEGG Pathways	AGE-RAGE signaling pathway in diabetic complications	MMP2|CCL2|AKT3|ICAM1|NOS3|BCL2|CDC42	3.30E-04	hsa04933
10	KEGG Pathways	Ras signaling pathway	GNB4|PDGFRA|PRKCG|AKT3|FLT1|CSF1R|KIT|BCL2L1|EPHA2|CDC42	3.50E-04	hsa04014
7	KEGG Pathways	Th17 cell differentiation	PPP3R1|RORA|RORC|LCK|RXRG|RXRB|PPP3CA	3.80E-04	hsa04659
6	KEGG Pathways	Platinum drug resistance	AKT3|BCL2L1|FAS|GSTO1|BCL2|TOP2B	4.10E-04	hsa01524
8	KEGG Pathways	Phagosome	THBS1|TUBB4A|ITGA2|TUBA1A|TUBA1C|CALR|TUBB4B|TLR4	4.20E-04	hsa04145
8	KEGG Pathways	Oxytocin signaling pathway	MAP2K5|PPP3R1|PRKCG|NOS3|PPP3CA|CAMK2B|CAMK2A|CAMK1D	4.80E-04	hsa04921
7	KEGG Pathways	Toxoplasmosis	LAMA4|LAMA5|AKT3|CCR5|BCL2L1|TLR4|BCL2	4.80E-04	hsa05145
7	KEGG Pathways	Cholinergic synapse	GNB4|PRKCG|AKT3|FYN|CAMK2B|BCL2|CAMK2A	5.00E-04	hsa04725
5	KEGG Pathways	Malaria	CCL2|THBS1|ICAM1|TLR4|SDC1	5.80E-04	hsa05144
7	KEGG Pathways	Oocyte meiosis	PPP3R1|PLK1|CDC25C|SLK|PPP3CA|CAMK2B|CAMK2A	6.10E-04	hsa04114
7	KEGG Pathways	Platelet activation	VWF|AKT3|FERMT3|ITGA2|NOS3|FYN|TLN2	8.30E-04	hsa04611
7	KEGG Pathways	Natural killer cell mediated cytotoxicity	PPP3R1|PRKCG|ICAM1|LCK|FYN|FAS|PPP3CA	8.40E-04	hsa04650
9	KEGG Pathways	Endocytosis	EHD4|ARF3|PDGFRA|CCR5|DAB2|CAV1|DNM1|CDC42|ARF1	0.0018	hsa04144
6	KEGG Pathways	T cell receptor signaling pathway	PPP3R1|AKT3|LCK|FYN|PPP3CA|CDC42	0.0018	hsa04660
5	KEGG Pathways	Long-term potentiation	PPP3R1|PRKCG|PPP3CA|CAMK2B|CAMK2A	0.0018	hsa04720
6	KEGG Pathways	Glucagon signaling pathway	PPP3R1|AKT3|PPP3CA|CAMK2B|LDHB|CAMK2A	0.0018	hsa04922
5	KEGG Pathways	Amphetamine addiction	PPP3R1|PRKCG|PPP3CA|CAMK2B|CAMK2A	0.0018	hsa05031
4	KEGG Pathways	African trypanosomiasis	LAMA4|PRKCG|ICAM1|FAS	0.0018	hsa05143
9	KEGG Pathways	HTLV-I infection	PPP3R1|PDGFRA|AKT3|ICAM1|BCL2L1|CALR|LCK|PPP3CA|TLN2	0.0019	hsa05166
5	KEGG Pathways	Non-small cell lung cancer	PRKCG|AKT3|RARB|RXRG|RXRB	0.0019	hsa05223
5	KEGG Pathways	Glioma	PDGFRA|PRKCG|AKT3|CAMK2B|CAMK2A	0.002	hsa05214
6	KEGG Pathways	Neurotrophin signaling pathway	MAP2K5|AKT3|CAMK2B|BCL2|CAMK2A|CDC42	0.003	hsa04722
5	KEGG Pathways	EGFR tyrosine kinase inhibitor resistance	PDGFRA|PRKCG|AKT3|BCL2L1|BCL2	0.0034	hsa01521
7	KEGG Pathways	Alzheimer's disease	GAPDH|PPP3R1|APP|CAPN2|SNCA|FAS|PPP3CA	0.0035	hsa05010
6	KEGG Pathways	Cell cycle	CDC25B|PLK1|CDC25A|CDC25C|WEE1|MCM3	0.0038	hsa04110
6	KEGG Pathways	Osteoclast differentiation	PPP3R1|AKT3|CSF1R|LCK|FYN|PPP3CA	0.0038	hsa04380

7	KEGG Pathways	Tuberculosis	PPP3R1|AKT3|TLR4|PPP3CA|CAMK2B|BCL2|CAMK2A	0.0038	hsa05152
6	KEGG Pathways	Dopaminergic synapse	GNB4|PRKCG|AKT3|PPP3CA|CAMK2B|CAMK2A	0.0043	hsa04728
7	KEGG Pathways	Calcium signaling pathway	PPP3R1|PDGFRA|PRKCG|NOS3|PPP3CA|CAMK2B|CAMK2A	0.0044	hsa04020
5	KEGG Pathways	Th1 and Th2 cell differentiation	PPP3R1|JAG2|LCK|DLL1|PPP3CA	0.0048	hsa04658
5	KEGG Pathways	GnRH signaling pathway	MMP2|MMP14|CAMK2B|CAMK2A|CDC42	0.0048	hsa04912
4	KEGG Pathways	Amyotrophic lateral sclerosis (ALS)	PPP3R1|BCL2L1|PPP3CA|BCL2	0.0048	hsa05014
7	KEGG Pathways	Kaposi's sarcoma-associated herpesvirus infection	GNB4|PPP3R1|AKT3|ICAM1|CCR5|FAS|PPP3CA	0.0048	hsa05167
5	KEGG Pathways	NF-kappa B signaling pathway	ICAM1|BCL2L1|LCK|TLR4|BCL2	0.0057	hsa04064
5	KEGG Pathways	Endocrine resistance	MMP2|AKT3|JAG2|DLL1|BCL2	0.0059	hsa01522
5	KEGG Pathways	Progesterone-mediated oocyte maturation	CDC25B|AKT3|PLK1|CDC25A|CDC25C	0.0059	hsa04914
5	KEGG Pathways	Amoebiasis	LAMA4|HSPB1|LAMA5|PRKCG|TLR4	0.0059	hsa05146
6	KEGG Pathways	Hepatitis B	PRKCG|AKT3|DDB1|FAS|TLR4|BCL2	0.0059	hsa05161
6	KEGG Pathways	Phospholipase D signaling pathway	PDGFRA|AKT3|KIT|FYN|DNM1|ARF1	0.0063	hsa04072
9	KEGG Pathways	Human papillomavirus infection	ITGB4|LAMA4|LAMA5|THBS1|VWF|AKT3|ITGA2|FAS|CDC42	0.0063	hsa05165
6	KEGG Pathways	Gastric cancer	AKT3|RARB|RXRG|RXRB|BCL2|ABCB1	0.0066	hsa05226
5	KEGG Pathways	Chagas disease (American trypanosomiasis)	CCL2|AKT3|CALR|FAS|TLR4	0.007	hsa05142
7	KEGG Pathways	Regulation of actin cytoskeleton	ITGB4|MYH9|PDGFRA|ITGA2|MYH10|CDC42|CFL1	0.0072	hsa04810
6	KEGG Pathways	Necroptosis	CAPN2|FAS|TLR4|CAMK2B|BCL2|CAMK2A	0.0081	hsa04217
5	KEGG Pathways	TNF signaling pathway	CCL2|AKT3|ICAM1|MMP14|FAS	0.0088	hsa04668
4	KEGG Pathways	Central carbon metabolism in cancer	PDGFRA|AKT3|KIT|G6PD	0.0089	hsa05230
5	KEGG Pathways	Leukocyte transendothelial migration	MMP2|PRKCG|ICAM1|CDH5|CDC42	0.0099	hsa04670
6	KEGG Pathways	Tight junction	MYH9|TJP1|TUBA1A|TUBA1C|MYH10|CDC42	0.0108	hsa04530
5	KEGG Pathways	Thyroid hormone signaling pathway	PRKCG|AKT3|RXRG|RXRB|TBC1D4	0.0108	hsa04919
5	KEGG Pathways	Sphingolipid signaling pathway	PRKCG|AKT3|NOS3|FYN|BCL2	0.0109	hsa04071
4	KEGG Pathways	Adherens junction	TJP1|FYN|PTPN1|CDC42	0.0111	hsa04520
4	KEGG Pathways	Bacterial invasion of epithelial cells	CAV1|ARHGEF26|DNM1|CDC42	0.0115	hsa05100
3	KEGG Pathways	Thyroid cancer	RXRG|TPM3|RXRB	0.0136	hsa05216
4	KEGG Pathways	ErbB signaling pathway	PRKCG|AKT3|CAMK2B|CAMK2A	0.0181	hsa04012
4	KEGG Pathways	Rheumatoid arthritis	CCL2|ICAM1|FLT1|TLR4	0.0186	hsa05323
6	KEGG Pathways	Epstein-Barr virus infection	HSPB1|AKT3|ICAM1|PTMA|BCL2|VIM	0.0194	hsa05169
5	KEGG Pathways	Adrenergic signaling in cardiomyocytes	AKT3|TPM3|CAMK2B|BCL2|CAMK2A	0.0206	hsa04261
7	KEGG Pathways	Cytokine-cytokine receptor interaction	CCL2|PDGFRA|FLT1|CSF1R|KIT|CCR5|FAS	0.0214	hsa04060
4	KEGG Pathways	Fc gamma R-mediated phagocytosis	PRKCG|AKT3|CDC42|CFL1	0.0214	hsa04666
5	KEGG Pathways	Wnt signaling pathway	PPP3R1|PRKCG|PPP3CA|CAMK2B|CAMK2A	0.0221	hsa04310
4	KEGG Pathways	Inflammatory mediator regulation of TRP channels	PRKCG|PRKCH|CAMK2B|CAMK2A	0.0232	hsa04750
3	KEGG Pathways	Notch signaling pathway	JAG2|DLL1|NUMB	0.0237	hsa04330
4	KEGG Pathways	Circadian entrainment	GNB4|PRKCG|CAMK2B|CAMK2A	0.0237	hsa04713
4	KEGG Pathways	Aldosterone synthesis and secretion	PRKCG|CAMK2B|CAMK2A|CAMK1D	0.0237	hsa04925
4	KEGG Pathways	Melanogenesis	PRKCG|KIT|CAMK2B|CAMK2A	0.0272	hsa04916
5	KEGG Pathways	Cellular senescence	PPP3R1|AKT3|CAPN2|CDC25A|PPP3CA	0.0289	hsa04218
5	KEGG Pathways	Protein processing in endoplasmic reticulum	CAPN2|CALR|BCL2|PDIA6|PRKCSH	0.0322	hsa04141
2	KEGG Pathways	Steroid biosynthesis	CYP24A1|CYP2R1	0.0327	hsa00100
5	KEGG Pathways	Hepatocellular carcinoma	PRKCG|AKT3|BCL2L1|NQO1|GSTO1	0.0327	hsa05225
3	KEGG Pathways	Viral myocarditis	ICAM1|CAV1|FYN	0.0327	hsa05416
4	KEGG Pathways	Insulin resistance	AKT3|NOS3|PTPN1|TBC1D4	0.0337	hsa04931
5	KEGG Pathways	Influenza A	CCL2|AKT3|ICAM1|FAS|TLR4	0.0358	hsa05164
5	KEGG Pathways	Transcriptional misregulation in cancer	FLT1|CSF1R|BCL2L1|RXRG|RXRB	0.0362	hsa05202
4	KEGG Pathways	Glutamatergic synapse	GNB4|PPP3R1|PRKCG|PPP3CA	0.0378	hsa04724
3	KEGG Pathways	Inflammatory bowel disease (IBD)	RORA|RORC|TLR4	0.0393	hsa05321
5	KEGG Pathways	Chemokine signaling pathway	CCL2|GNB4|AKT3|CCR5|CDC42	0.0454	hsa04062
3	KEGG Pathways	Acute myeloid leukemia	AKT3|CSF1R|KIT	0.0454	hsa05221
3	KEGG Pathways	Glycolysis / Gluconeogenesis	GAPDH|ENO1|LDHB	0.0481	hsa00010
3	KEGG Pathways	Adipocytokine signaling pathway	AKT3|RXRG|RXRB	0.0494	hsa04920
22	Pfam	Protein kinase domain	MAP2K5|PDGFRA|PRKCG|AKT3|FLT1|CSF1R|KIT|MELK|PLK1|BRSK1|PRKCH|LCK|MAP4K4|FYN|EPHA2|SLK|CAMK2B|CAMK2A|DYRK1A|WEE1|PBK|CAMK1D	5.39E-08	PF00069
22	Pfam	Protein tyrosine kinase	MAP2K5|PDGFRA|PRKCG|AKT3|FLT1|CSF1R|KIT|MELK|PLK1|BRSK1|PRKCH|LCK|MAP4K4|FYN|EPHA2|SLK|CAMK2B|CAMK2A|DYRK1A|WEE1|PBK|CAMK1D	5.39E-08	PF07714
8	Pfam	Zinc finger, C4 type (two domains)	RORA|RORC|RARB|NR1I2|RXRG|RXRB|RARG|NR1H4	1.23E-06	PF00105
8	Pfam	Ligand-binding domain of nuclear hormone receptor	RORA|RORC|RARB|NR1I2|RXRG|RXRB|RARG|NR1H4	1.24E-06	PF00104
4	Pfam	Annexin	ANXA5|ANXA2|ANXA6|ANXA1	5.70E-04	PF00191
3	Pfam	M-phase inducer phosphatase	CDC25B|CDC25A|CDC25C	5.70E-04	PF06617
3	Pfam	Tropomyosin	TPM4|TPM3|TPM2	7.00E-04	PF00261
3	Pfam	Bcl-2 homology region 4	BCL2L2|BCL2L1|BCL2	7.00E-04	PF02180
3	Pfam	Tropomyosin like	TPM4|TPM3|TPM2	7.00E-04	PF12718
6	Pfam	Calponin homology (CH) domain	CNN2|FLNC|FLNA|CNN3|FLNB|SPTBN2	8.30E-04	PF00307
4	Pfam	Tubulin C-terminal domain	TUBB4A|TUBA1A|TUBA1C|TUBB4B	0.0016	PF03953
4	Pfam	Tubulin/FtsZ family, GTPase domain	TUBB4A|TUBA1A|TUBA1C|TUBB4B	0.002	PF00091
3	Pfam	Filamin/ABP280 repeat	FLNC|FLNA|FLNB	0.003	PF00630
4	Pfam	Phosphotyrosine interaction domain (PTB/PID)	DAB2|TBC1D4|NUMB|APBA2	0.003	PF00640
3	Pfam	Apoptosis regulator proteins, Bcl-2 family	BCL2L2|BCL2L1|BCL2	0.0033	PF00452
4	Pfam	Myosin head (motor domain)	MYH9|MYO1C|MYH10|MYO1B	0.0064	PF00063
2	Pfam	Amyloid A4 N-terminal heparin-binding	APLP1|APP	0.0102	PF02177
7	Pfam	Variant SH3 domain	ARHGEF5|NGEF|TJP1|LCK|FYN|ARHGEF26|SPTAN1	0.0102	PF07653
2	Pfam	beta-amyloid precursor protein C-terminus	APLP1|APP	0.0102	PF10515
2	Pfam	Copper-binding of amyloid precursor, CuBD	APLP1|APP	0.0102	PF12924
2	Pfam	E2 domain of amyloid precursor protein	APLP1|APP	0.0102	PF12925
3	Pfam	Rhodanese-like domain	CDC25B|CDC25A|CDC25C	0.0117	PF00581
2	Pfam	Delta serrate ligand	JAG2|DLL1	0.0117	PF01414
2	Pfam	N terminus of Notch ligand	JAG2|DLL1	0.0117	PF07657
2	Pfam	Calcium/calmodulin dependent protein kinase II association domain	CAMK2B|CAMK2A	0.0117	PF08332
2	Pfam	SnoaL-like domain	CAMK2B|CAMK2A	0.0117	PF13474
2	Pfam	Domain of unknown function (DUF4440)	CAMK2B|CAMK2A	0.0117	PF14534
2	Pfam	PTRF/SDPR family	SDPR|PTRF	0.0117	PF15237
2	Pfam	Laminin Domain I	LAMA4|LAMA5	0.0124	PF06008
2	Pfam	Laminin Domain II	LAMA4|LAMA5	0.0124	PF06009
3	Pfam	von Willebrand factor type C domain	PXDN|THBS1|VWF	0.0154	PF00093
2	Pfam	Calponin family repeat	CNN2|CNN3	0.0154	PF00402
2	Pfam	C-terminal leucine zipper domain of cyclic nucleotide-gated channels	TPM4|TPM3	0.0154	PF16526
6	Pfam	Variant SH3 domain	ARHGEF5|NGEF|LCK|FYN|ARHGEF26|SPTAN1	0.0163	PF14604
2	Pfam	Syndecan domain	SDC4|SDC1	0.0174	PF01034
2	Pfam	2OG-Fe(II) oxygenase superfamily	PLOD1|PLOD2	0.0174	PF03171
3	Pfam	Protein kinase C terminal domain	PRKCG|AKT3|PRKCH	0.0177	PF00433

2	Pfam	Unconventional myosin tail, actin- and lipid-binding	MYO1C|MYO1B	0.0199	PF06017
6	Pfam	SH3 domain	ARHGEF5|NGEF|LCK|FYN|ARHGEF26|SPTAN1	0.0216	PF00018
4	Pfam	RhoGEF domain	ARHGEF5|NGEF|ARHGEF26|ARHGEF15	0.0226	PF00621
4	Pfam	Kinase-like	AKT3|MELK|PLK1|CAMK1D	0.0317	PF14531
3	Pfam	IQ calmodulin-binding motif	MYO1C|MYH10|MYO1B	0.0378	PF00612
6	Pfam	PH domain	PLEK|AKT3|NGEF|FERMT3|DNM1|SPTBN2	0.0394	PF00169
2	Pfam	Fibronectin type II domain	MMP2|FN1	0.0444	PF00040
2	Pfam	Myosin N-terminal SH3-like domain	MYH9|MYH10	0.0444	PF02736
2	Pfam	Phosphotyrosine-binding domain	TENC1|NUMB	0.0444	PF08416
3	Pfam	Signal recognition particle receptor beta subunit	ARF3|ARF4|ARF1	0.0444	PF09439
4	Pfam	EF hand	RCN1|PPP3R1|SPTAN1|PRKCSH	0.0444	PF13202
2	Pfam	Glutathione S-transferase, C-terminal domain	GSTO1|CLIC4	0.0444	PF13410
2	Pfam	Pleckstrin homology domain	PLEK|SPTBN2	0.0444	PF15410
2	Pfam	Dynamin family	EHD4|DNM1	0.0483	PF00350
64	Reactome Pathways	Signal Transduction	ARHGEF5|MAP2K5|MYH9|LAMA4|RBP1|GNB4|PPP3R1|HSPB1|LAMA5|PDGFRA|THBS1|VWF|PRKCG|AKT3|NGEF|TUBB4A|ARHGAP31|TJP1|FLT1|APP|KIT|CCR5|ITGA2|NOS3|PLK1|TUBA1A|TUBA1C|CDC25C|JAG2|PRKCH|RARB|LCK|CAV1|RGS12|TUBB4B|CDH5|FYN|FAS|ARHGEF26|SFPQ|RXRG|MYH10|ARHGEF15|DLL1|ARHGAP30|FLNA|PTPN1|SDC4|SPTAN1|DNM1|RXRB|ANXA1|SDC1|PPP3CA|CAMK2B|LTB4R|BCL2|CAMK2A|SEPT7|CDC42|RARG|SPTBN2|CFL1|NUMB	2.03E-13	HSA-162582
29	Reactome Pathways	Cytokine Signaling in Immune system	MMP2|CCL2|MT2A|LAMA5|RORA|CNN2|ICAM1|APP|CSF1R|CCR5|BCL2L1|RORC|LCK|ANXA2|FYN|GSTO1|FLNA|PTPN1|ANXA1|SDC1|FSCN1|CAMK2B|BCL2|CAMK2A|CDC42|FLNB|CFL1|ARF1|VIM	1.37E-10	HSA-1280215
23	Reactome Pathways	Signaling by Interleukins	MMP2|CCL2|LAMA5|RORA|CNN2|ICAM1|APP|CSF1R|CCR5|BCL2L1|RORC|LCK|ANXA2|FYN|GSTO1|ANXA1|SDC1|FSCN1|BCL2|CDC42|CFL1|ARF1|VIM	1.30E-09	HSA-449147
26	Reactome Pathways	Hemostasis	GNB4|PLEK|THBS1|VWF|PRKCG|TUBB4A|FERMT3|APP|ANXA5|ITGA2|NOS3|TUBA1A|TUBA1C|PRKCH|LCK|CAV1|TUBB4B|ANXA2|FYN|FLNA|PTPN1|SDC4|SDC1|CDC42|WEE1|CFL1	2.20E-09	HSA-109582
47	Reactome Pathways	Immune System	MYH9|MMP2|CCL2|PPP3R1|MT2A|LAMA5|RORA|CNN2|AKT3|TUBB4A|ICAM1|APP|CSF1R|CCR5|NOS3|CCT2|TUBA1A|TUBA1C|BCL2L1|CALR|RORC|LCK|TUBB4B|ANXA2|FYN|MYO1C|GSTO1|FLNA|PTPN1|SPTAN1|DNM1|TLR4|ANXA1|SDC1|FSCN1|XRCC5|PPP3CA|CAMK2B|BCL2|CAMK2A|CDC42|FLNB|SPTBN2|CFL1|ARF1|VIM|PRKCSH	2.20E-09	HSA-168256
17	Reactome Pathways	Extracellular matrix organization	PLOD1|ITGB4|MMP2|LAMA4|PXDN|LAMA5|THBS1|COL8A1|VWF|ICAM1|PLOD2|APP|CAPN2|ITGA2|MMP14|SDC4|SDC1	1.33E-07	HSA-1474244
11	Reactome Pathways	Interleukin-4 and Interleukin-13 signaling	MMP2|CCL2|LAMA5|RORA|ICAM1|BCL2L1|RORC|ANXA1|FSCN1|BCL2|VIM	3.62E-07	HSA-6785807
15	Reactome Pathways	Platelet activation, signaling and aggregation	GNB4|PLEK|THBS1|VWF|PRKCG|FERMT3|APP|ANXA5|PRKCH|LCK|FYN|FLNA|PTPN1|CDC42|CFL1	7.04E-07	HSA-76002
10	Reactome Pathways	COPI-mediated anterograde transport	ARF3|TUBB4A|TUBA1A|TUBA1C|ARF4|TUBB4B|COPA|SPTAN1|SPTBN2|ARF1	1.87E-06	HSA-6807878
18	Reactome Pathways	Signaling by Receptor Tyrosine Kinases	MAP2K5|LAMA4|HSPB1|LAMA5|PDGFRA|THBS1|AKT3|FLT1|KIT|ITGA2|NOS3|LCK|CAV1|CDH5|FYN|PTPN1|DNM1|CDC42	3.22E-06	HSA-9006934
17	Reactome Pathways	Signaling by Rho GTPases	ARHGEF5|MYH9|NGEF|TUBB4A|ARHGAP31|PLK1|TUBA1A|TUBA1C|CDC25C|TUBB4B|ARHGEF26|MYH10|ARHGEF15|ARHGAP30|FLNA|CDC42|CFL1	4.93E-06	HSA-194315
9	Reactome Pathways	EPH-Ephrin signaling	MYH9|MMP2|NGEF|FYN|EPHA2|MYH10|DNM1|CDC42|CFL1	8.43E-06	HSA-2682334
7	Reactome Pathways	Gap junction trafficking and regulation	TUBB4A|TJP1|TUBA1A|TUBA1C|DAB2|TUBB4B|DNM1	1.58E-05	HSA-157858
7	Reactome Pathways	Non-integrin membrane-ECM interactions	ITGB4|LAMA4|LAMA5|THBS1|ITGA2|SDC4|SDC1	4.71E-05	HSA-3000171
9	Reactome Pathways	L1CAM interactions	TUBB4A|ITGA2|TUBA1A|TUBA1C|TUBB4B|SPTAN1|DNM1|SPTBN2|NUMB	4.71E-05	HSA-373760
18	Reactome Pathways	Axon guidance	MYH9|MMP2|NGEF|TUBB4A|ITGA2|TUBA1A|TUBA1C|TUBB4B|DOK4|FYN|EPHA2|MYH10|SPTAN1|DNM1|CDC42|SPTBN2|CFL1|NUMB	4.71E-05	HSA-422475
10	Reactome Pathways	Apoptosis	PPP3R1|AKT3|ADD1|TJP1|BCL2L1|FAS|SPTAN1|TLR4|BCL2|VIM	6.07E-05	HSA-109581
25	Reactome Pathways	Disease	CYP24A1|RBP1|CDC25B|PDGFRA|THBS1|VWF|AKT3|APP|KIT|CCR5|CAPN2|CDC25A|CALR|CDC25C|JAG2|CYP2R1|LCK|FYN|DLL1|SDC4|TLR4|PSIP1|SDC1|XRCC5|ARF1	6.07E-05	HSA-1643685
6	Reactome Pathways	Nuclear Receptor transcription pathway	RORA|RORC|RARB|RXRG|RXRB|RARG	6.07E-05	HSA-383280
6	Reactome Pathways	Gene and protein expression by JAK-STAT signaling after Interleukin-12 stimulation	CNN2|ANXA2|GSTO1|CDC42|CFL1|ARF1	6.07E-05	HSA-8950505
8	Reactome Pathways	COPI-dependent Golgi-to-ER retrograde traffic	ARF3|TUBB4A|TUBA1A|TUBA1C|ARF4|TUBB4B|COPA|ARF1	7.19E-05	HSA-6811434
9	Reactome Pathways	Response to elevated platelet cytosolic Ca2+	PLEK|THBS1|VWF|PRKCG|FERMT3|APP|ANXA5|FLNA|CFL1	7.19E-05	HSA-76005
5	Reactome Pathways	Deregulated CDK5 triggers multiple neurodegenerative pathways in Alzheimer's disease models	CDC25B|APP|CAPN2|CDC25A|CDC25C	7.19E-05	HSA-8862803
7	Reactome Pathways	Translocation of SLC2A4 (GLUT4) to the plasma membrane	MYH9|TUBB4A|TUBA1A|TUBA1C|TUBB4B|MYO1C|TBC1D4	7.58E-05	HSA-1445148
5	Reactome Pathways	Formation of tubulin folding intermediates by CCT/TriC	TUBB4A|CCT2|TUBA1A|TUBA1C|TUBB4B	7.58E-05	HSA-389960
19	Reactome Pathways	Vesicle-mediated transport	MYH9|ARF3|AKT3|TUBB4A|TJP1|APP|TUBA1A|TUBA1C|ARF4|DAB2|CALR|TUBB4B|MYO1C|COPA|SPTAN1|DNM1|TBC1D4|SPTBN2|ARF1	7.58E-05	HSA-5653656
6	Reactome Pathways	Recycling pathway of L1	TUBB4A|TUBA1A|TUBA1C|TUBB4B|DNM1|NUMB	8.86E-05	HSA-437239
6	Reactome Pathways	Gap junction trafficking	TUBB4A|TUBA1A|TUBA1C|DAB2|TUBB4B|DNM1	9.46E-05	HSA-190828
8	Reactome Pathways	Signaling by VEGF	HSPB1|AKT3|FLT1|NOS3|CAV1|CDH5|FYN|CDC42	9.46E-05	HSA-194138
5	Reactome Pathways	Cyclin A/B1/B2 associated events during G2/M transition	CDC25B|PLK1|CDC25A|CDC25C|WEE1	9.46E-05	HSA-69273
18	Reactome Pathways	Membrane Trafficking	MYH9|ARF3|AKT3|TUBB4A|TJP1|APP|TUBA1A|TUBA1C|ARF4|DAB2|TUBB4B|MYO1C|COPA|SPTAN1|DNM1|TBC1D4|SPTBN2|ARF1	9.88E-05	HSA-199991
5	Reactome Pathways	Syndecan interactions	ITGB4|THBS1|ITGA2|SDC4|SDC1	9.88E-05	HSA-3000170
5	Reactome Pathways	Prefoldin mediated transfer of substrate to CCT/TriC	TUBB4A|CCT2|TUBA1A|TUBA1C|TUBB4B	9.88E-05	HSA-389957
5	Reactome Pathways	EPHA-mediated growth cone collapse	MYH9|NGEF|FYN|EPHA2|MYH10	1.30E-04	HSA-3928663
5	Reactome Pathways	RHO GTPases activate IQGAPs	TUBB4A|TUBA1A|TUBA1C|TUBB4B|CDC42	1.30E-04	HSA-5626467
12	Reactome Pathways	Asparagine N-linked glycosylation	ARF3|TUBB4A|TUBA1A|TUBA1C|ARF4|CALR|TUBB4B|COPA|SPTAN1|SPTBN2|ARF1|PRKCSH	1.80E-04	HSA-446203
8	Reactome Pathways	Platelet degranulation	PLEK|THBS1|VWF|FERMT3|APP|ANXA5|FLNA|CFL1	2.40E-04	HSA-114608
4	Reactome Pathways	Polo-like kinase mediated events	PLK1|CDC25A|CDC25C|WEE1	2.90E-04	HSA-156711
7	Reactome Pathways	VEGFA-VEGFR2 Pathway	HSPB1|AKT3|NOS3|CAV1|CDH5|FYN|CDC42	3.30E-04	HSA-4420097
4	Reactome Pathways	Microtubule-dependent trafficking of connexons from Golgi to the plasma membrane	TUBB4A|TUBA1A|TUBA1C|TUBB4B	3.40E-04	HSA-190840
11	Reactome Pathways	RHO GTPase Effectors	MYH9|TUBB4A|PLK1|TUBA1A|TUBA1C|CDC25C|TUBB4B|MYH10|FLNA|CDC42|CFL1	3.60E-04	HSA-195258
11	Reactome Pathways	MAPK family signaling cascades	HSPB1|PDGFRA|VWF|KIT|FYN|SPTAN1|CAMK2B|CAMK2A|SEPT7|CDC42|SPTBN2	3.60E-04	HSA-5683057
4	Reactome Pathways	Post-chaperonin tubulin folding pathway	TUBB4A|TUBA1A|TUBA1C|TUBB4B	5.50E-04	HSA-389977
4	Reactome Pathways	RHO GTPases activate PAKs	MYH9|MYH10|FLNA|CDC42	5.50E-04	HSA-5627123
9	Reactome Pathways	G2/M Transition	CDC25B|TUBB4A|PLK1|TUBA1A|TUBA1C|CDC25A|CDC25C|TUBB4B|WEE1	5.50E-04	HSA-69275
7	Reactome Pathways	MHC class II antigen presentation	TUBB4A|TUBA1A|TUBA1C|TUBB4B|DNM1|SPTBN2|ARF1	0.0011	HSA-2132295
4	Reactome Pathways	VEGFR2 mediated vascular permeability	AKT3|NOS3|CAV1|CDH5	0.0014	HSA-5218920
21	Reactome Pathways	Innate Immune System	MYH9|PPP3R1|CNN2|APP|NOS3|CCT2|BCL2L1|LCK|TUBB4B|ANXA2|FYN|MYO1C|SPTAN1|DNM1|TLR4|XRCC5|PPP3CA|BCL2|CDC42|CFL1|PRKCSH	0.0016	HSA-168249

21	Reactome Pathways	Developmental Biology	MYH9|MMP2|AKT3|NGEF|TUBB4A|ITGA2|TUBA1A|TUBA1C|RARB|TUBB4B|DOK4|FYN|EPHA2|MYH10|SPTAN1|DNM1|CDC42|RARG|SPTBN2|CFL1|NUMB	0.0018	HSA-1266738
4	Reactome Pathways	Laminin interactions	ITGB4|LAMA4|LAMA5|ITGA2	0.0019	HSA-3000157
6	Reactome Pathways	Chaperonin-mediated protein folding	GNB4|TUBB4A|CCT2|TUBA1A|TUBA1C|TUBB4B	0.0019	HSA-390466
4	Reactome Pathways	CD28 co-stimulation	AKT3|LCK|FYN|CDC42	0.0021	HSA-389356
3	Reactome Pathways	CD28 dependent Vav1 pathway	LCK|FYN|CDC42	0.0021	HSA-389359
7	Reactome Pathways	Rho GTPase cycle	ARHGEF5|NGEF|ARHGAP31|ARHGEF26|ARHGEF15|ARHGAP30|CDC42	0.0023	HSA-194840
4	Reactome Pathways	Smooth Muscle Contraction	ANXA2|ANXA6|TPM3|ANXA1	0.0023	HSA-445355
3	Reactome Pathways	Caspase-mediated cleavage of cytoskeletal proteins	ADD1|SPTAN1|VIM	0.0024	HSA-264870
3	Reactome Pathways	Regulation of KIT signaling	KIT|LCK|FYN	0.0029	HSA-1433559
3	Reactome Pathways	Early Phase of HIV Life Cycle	CCR5|PSIP1|XRCC5	0.0033	HSA-162594
4	Reactome Pathways	Apoptotic cleavage of cellular proteins	ADD1|TJP1|SPTAN1|VIM	0.0034	HSA-111465
3	Reactome Pathways	Activation of BAD and translocation to mitochondria	PPP3R1|AKT3|BCL2	0.0038	HSA-111447
3	Reactome Pathways	Platelet Adhesion to exposed collagen	VWF|ITGA2|FYN	0.0038	HSA-75892
4	Reactome Pathways	Carboxyterminal post-translational modifications of tubulin	TUBB4A|TUBA1A|TUBA1C|TUBB4B	0.0039	HSA-8955332
5	Reactome Pathways	The role of GTSE1 in G2/M progression after G2 checkpoint	TUBB4A|PLK1|TUBA1A|TUBA1C|TUBB4B	0.0042	HSA-8852276
11	Reactome Pathways	G alpha (i) signalling events	RBP1|GNB4|PPP3R1|PRKCG|APP|CCR5|RGS12|SDC4|ANXA1|SDC1|PPP3CA	0.0044	HSA-418594
16	Reactome Pathways	Adaptive Immune System	PPP3R1|AKT3|TUBB4A|ICAM1|TUBA1A|TUBA1C|CALR|LCK|TUBB4B|FYN|DNM1|TLR4|PPP3CA|CDC42|SPTBN2|ARF1	0.0045	HSA-1280218
4	Reactome Pathways	Intrinsic Pathway for Apoptosis	PPP3R1|AKT3|BCL2L1|BCL2	0.0053	HSA-109606
5	Reactome Pathways	G alpha (12/13) signalling events	ARHGEF5|GNB4|NGEF|ARHGEF26|ARHGEF15	0.0053	HSA-416482
4	Reactome Pathways	Signaling by Retinoic Acid	RARB|RXRG|RXRB|RARG	0.0053	HSA-5362517
2	Reactome Pathways	The NLRP1 inflammasome	BCL2L1|BCL2	0.0055	HSA-844455
3	Reactome Pathways	RHO GTPases Activate ROCKs	MYH9|MYH10|CFL1	0.0062	HSA-5627117
6	Reactome Pathways	Cell-Cell communication	ITGB4|FLNC|CDH5|FYN|FLNA|SPTAN1	0.0067	HSA-1500931
5	Reactome Pathways	Integrin cell surface interactions	THBS1|COL8A1|VWF|ICAM1|ITGA2	0.0067	HSA-216083
8	Reactome Pathways	RAF/MAP kinase cascade	PDGFRA|VWF|KIT|FYN|SPTAN1|CAMK2B|CAMK2A|SPTBN2	0.0067	HSA-5673001
5	Reactome Pathways	Fcgamma receptor (FCGR) dependent phagocytosis	MYH9|FYN|MYO1C|CDC42|CFL1	0.0068	HSA-2029480
5	Reactome Pathways	PI3K/AKT Signaling in Cancer	PDGFRA|AKT3|KIT|LCK|FYN	0.0068	HSA-2219528
3	Reactome Pathways	CTLA4 inhibitory signaling	AKT3|LCK|FYN	0.0068	HSA-389513
3	Reactome Pathways	Inflammasomes	APP|BCL2L1|BCL2	0.0068	HSA-622312
12	Reactome Pathways	Cell Cycle, Mitotic	CDC25B|AKT3|TUBB4A|PLK1|TUBA1A|TUBA1C|CDC25A|CDC25C|TUBB4B|DYRK1A|WEE1|MCM3	0.0068	HSA-69278
4	Reactome Pathways	COPI-independent Golgi-to-ER retrograde traffic	TUBB4A|TUBA1A|TUBA1C|TUBB4B	0.007	HSA-6811436
5	Reactome Pathways	Interferon gamma signaling	MT2A|ICAM1|PTPN1|CAMK2B|CAMK2A	0.007	HSA-877300
2	Reactome Pathways	NOSTRIN mediated eNOS trafficking	NOS3|CAV1	0.0071	HSA-203641
6	Reactome Pathways	RHO GTPases Activate Formins	TUBB4A|PLK1|TUBA1A|TUBA1C|TUBB4B|CDC42	0.0071	HSA-5663220
4	Reactome Pathways	EPH-ephrin mediated repulsion of cells	MMP2|FYN|EPHA2|DNM1	0.0073	HSA-3928665
4	Reactome Pathways	Intraflagellar transport	TUBB4A|TUBA1A|TUBA1C|TUBB4B	0.0073	HSA-5620924
5	Reactome Pathways	Collagen formation	PLOD1|ITGB4|PXDN|COL8A1|PLOD2	0.0076	HSA-1474290
3	Reactome Pathways	CD28 dependent PI3K/Akt signaling	AKT3|LCK|FYN	0.0076	HSA-389357
5	Reactome Pathways	Recruitment of NuMA to mitotic centrosomes	TUBB4A|PLK1|TUBA1A|TUBA1C|TUBB4B	0.0081	HSA-380320
5	Reactome Pathways	Negative regulation of the PI3K/AKT network	PDGFRA|AKT3|KIT|LCK|FYN	0.0083	HSA-199418
4	Reactome Pathways	HSP90 chaperone cycle for steroid hormone receptors (SHR)	TUBB4A|TUBA1A|TUBA1C|TUBB4B	0.0083	HSA-3371497
7	Reactome Pathways	Interferon Signaling	MT2A|ICAM1|FLNA|PTPN1|CAMK2B|CAMK2A|FLNB	0.0083	HSA-913531
13	Reactome Pathways	Cell Cycle	CDC25B|AKT3|TUBB4A|PLK1|TUBA1A|TUBA1C|CDC25A|CDC25C|TUBB4B|DYRK1A|SUN1|WEE1|MCM3	0.0096	HSA-1640170
7	Reactome Pathways	Cilium Assembly	TUBB4A|CCT2|PLK1|TUBA1A|TUBA1C|ARF4|TUBB4B	0.0096	HSA-5617833
7	Reactome Pathways	Muscle contraction	ANXA2|ANXA6|TPM3|ANXA1|CAMK2B|CAMK2A|VIM	0.0103	HSA-397014
3	Reactome Pathways	The role of Nef in HIV-1 replication and disease pathogenesis	LCK|FYN|ARF1	0.0108	HSA-164952
4	Reactome Pathways	NRAGE signals death through JNK	ARHGEF5|NGEF|ARHGEF26|ARHGEF15	0.0108	HSA-193648
4	Reactome Pathways	Constitutive Signaling by Aberrant PI3K in Cancer	PDGFRA|KIT|LCK|FYN	0.0108	HSA-2219530
4	Reactome Pathways	Transcriptional Regulation by MECP2	DLL1|PTPN1|CAMK2B|CAMK2A	0.0108	HSA-8986944
4	Reactome Pathways	Kinesins	TUBB4A|TUBA1A|TUBA1C|TUBB4B	0.0108	HSA-983189
2	Reactome Pathways	Vitamins	CYP24A1|CYP2R1	0.0109	HSA-211916
4	Reactome Pathways	Ca2+ pathway	GNB4|PPP3R1|PPP3CA|CAMK2A	0.0109	HSA-4086398
4	Reactome Pathways	Regulation of actin dynamics for phagocytic cup formation	MYH9|MYO1C|CDC42|CFL1	0.0112	HSA-2029482
3	Reactome Pathways	Trafficking of AMPA receptors	PRKCG|CAMK2B|CAMK2A	0.0112	HSA-399719
6	Reactome Pathways	Factors involved in megakaryocyte development and platelet production	TUBB4A|TUBA1A|TUBA1C|TUBB4B|CDC42|WEE1	0.012	HSA-983231
2	Reactome Pathways	2-LTR circle formation	PSIP1|XRCC5	0.0129	HSA-164843
2	Reactome Pathways	Nef and signal transduction	LCK|FYN	0.0129	HSA-164944
2	Reactome Pathways	Constitutive Signaling by NOTCH1 t(7;9)(NOTCH1:M1580_K2555) Translocation Mutant	JAG2|DLL1	0.0129	HSA-2660826
4	Reactome Pathways	Semaphorin interactions	MYH9|FYN|MYH10|CFL1	0.0129	HSA-373755
2	Reactome Pathways	Formyl peptide receptors bind formyl peptides and many other ligands	APP|ANXA1	0.0129	HSA-444473
3	Reactome Pathways	MET activates PTK2 signaling	LAMA4|LAMA5|ITGA2	0.0129	HSA-8874081
5	Reactome Pathways	Hedgehog 'off' state	TUBB4A|TUBA1A|TUBA1C|TUBB4B|NUMB	0.0132	HSA-5610787
3	Reactome Pathways	Activated NOTCH1 Transmits Signal to the Nucleus	JAG2|DLL1|NUMB	0.0144	HSA-2122948
2	Reactome Pathways	BH3-only proteins associate with and inactivate anti-apoptotic BCL-2 members	BCL2L1|BCL2	0.0151	HSA-111453
4	Reactome Pathways	Loss of Nlp from mitotic centrosomes	TUBB4A|PLK1|TUBA1A|TUBB4B	0.0154	HSA-380259
3	Reactome Pathways	GPVI-mediated activation cascade	LCK|FYN|CDC42	0.0176	HSA-114604
4	Reactome Pathways	AURKA Activation by TPX2	TUBB4A|PLK1|TUBA1A|TUBB4B	0.0176	HSA-8854518
2	Reactome Pathways	Nef Mediated CD4 Down-regulation	LCK|ARF1	0.0177	HSA-167590
2	Reactome Pathways	Calcineurin activates NFAT	PPP3R1|PPP3CA	0.0177	HSA-2025928
5	Reactome Pathways	Resolution of Sister Chromatid Cohesion	TUBB4A|PLK1|TUBA1A|TUBA1C|TUBB4B	0.0177	HSA-2500257
9	Reactome Pathways	Diseases of signal transduction	RBP1|PDGFRA|VWF|AKT3|KIT|JAG2|LCK|FYN|DLL1	0.0181	HSA-5663202
4	Reactome Pathways	ECM proteoglycans	LAMA4|LAMA5|APP|ITGA2	0.0202	HSA-3000178
5	Reactome Pathways	Regulation of Insulin-like Growth Factor (IGF) transport and uptake by Insulin-like Growth Factor Binding Proteins (IGFBPs)	RCN1|MMP2|APP|PDIA6|PRKCSH	0.0203	HSA-381426
3	Reactome Pathways	Activation of ATR in response to replication stress	CDC25A|CDC25C|MCM3	0.0206	HSA-176187

4	Reactome Pathways	Signaling by MET	LAMA4|LAMA5|ITGA2|PTPN1	0.0206	HSA-6806834
18	Reactome Pathways	GPCR downstream signalling	ARHGEF5|RBP1|GNB4|PPP3R1|PRKCG|AKT3|NGEF|APP|CCR5|PRKCH|RGS12|ARHGEF26|ARHGEF15|SDC4|ANXA1|SDC1|PPP3CA|LTB4R	0.0213	HSA-388396
2	Reactome Pathways	Formation of annular gap junctions	DAB2|DNM1	0.0229	HSA-196025
2	Reactome Pathways	Vitamin D (calciferol) metabolism	CYP24A1|CYP2R1	0.0229	HSA-196791
2	Reactome Pathways	eNOS activation	NOS3|CAV1	0.0229	HSA-203615
2	Reactome Pathways	PECAM1 interactions	LCK|FYN	0.0229	HSA-210990
4	Reactome Pathways	Recruitment of mitotic centrosome proteins and complexes	TUBB4A|PLK1|TUBA1A|TUBB4B	0.0229	HSA-380270
2	Reactome Pathways	GP1b-IX-V activation signalling	VWF|FLNA	0.0229	HSA-430116
2	Reactome Pathways	CLEC7A (Dectin-1) induces NFAT activation	PPP3R1|PPP3CA	0.0229	HSA-5607763
4	Reactome Pathways	Opioid Signalling	GNB4|PPP3R1|PRKCG|PPP3CA	0.0245	HSA-111885
3	Reactome Pathways	EPHB-mediated forward signaling	FYN|CDC42|CFL1	0.0245	HSA-3928662
4	Reactome Pathways	Cyclin A:Cdk2-associated events at S phase entry	CDC25B|AKT3|CDC25A|WEE1	0.0245	HSA-69656
5	Reactome Pathways	Cell surface interactions at the vascular wall	LCK|CAV1|FYN|SDC4|SDC1	0.0263	HSA-202733
4	Reactome Pathways	Regulation of PLK1 Activity at G2/M Transition	TUBB4A|PLK1|TUBA1A|TUBB4B	0.0268	HSA-2565942
4	Reactome Pathways	PI5P, PP2A and IER3 Regulate PI3K/AKT Signaling	PDGFRA|KIT|LCK|FYN	0.0268	HSA-6811558
27	Reactome Pathways	Metabolism of proteins	RCN1|MMP2|CCL2|GNB4|ARF3|THBS1|RORA|TUBB4A|ADD1|APP|CCT2|TUBA1A|TUBA1C|DDB1|CDC25A|ARF4|CALR|SNCA|TUBB4B|DPP4|COPA|SPTAN1|PDIA6|TOP2B|SPTBN2|ARF1|PRKCSH	0.0271	HSA-392499
2	Reactome Pathways	Chk1/Chk2(Cds1) mediated inactivation of Cyclin B:Cdk1 complex	CDC25C|WEE1	0.0272	HSA-75035
2	Reactome Pathways	Advanced glycosylation endproduct receptor signaling	APP|PRKCSH	0.0272	HSA-879415
3	Reactome Pathways	Retinoid metabolism and transport	RBP1|SDC4|SDC1	0.028	HSA-975634
5	Reactome Pathways	Degradation of the extracellular matrix	MMP2|LAMA5|COL8A1|CAPN2|MMP14	0.0283	HSA-1474228
5	Reactome Pathways	Death Receptor Signalling	ARHGEF5|NGEF|FAS|ARHGEF26|ARHGEF15	0.0283	HSA-73887
5	Reactome Pathways	Beta-catenin independent WNT signaling	GNB4|PPP3R1|PRKCG|PPP3CA|CAMK2A	0.0288	HSA-3858494
3	Reactome Pathways	Interleukin-10 signaling	CCL2|ICAM1|CCR5	0.029	HSA-6783783
2	Reactome Pathways	Defective EXT2 causes exostoses 2	SDC4|SDC1	0.0296	HSA-3656237
2	Reactome Pathways	Defective EXT1 causes exostoses 1, TRPS2 and CHDS	SDC4|SDC1	0.0296	HSA-3656253
4	Reactome Pathways	Cell junction organization	ITGB4|FLNC|CDH5|FLNA	0.0306	HSA-446728
3	Reactome Pathways	G alpha (z) signalling events	GNB4|PRKCG|PRKCH	0.031	HSA-418597
5	Reactome Pathways	Mitotic G1-G1/S phases	AKT3|CDC25A|DYRK1A|WEE1|MCM3	0.031	HSA-453279
2	Reactome Pathways	Caspase activation via Death Receptors in the presence of ligand	FAS|TLR4	0.0316	HSA-140534
2	Reactome Pathways	Constitutive Signaling by NOTCH1 HD Domain Mutants	JAG2|DLL1	0.0316	HSA-2691232
2	Reactome Pathways	Nucleobase biosynthesis	CAD|PAICS	0.0316	HSA-8956320
4	Reactome Pathways	Unfolded Protein Response (UPR)	CCL2|ADD1|CALR|PDIA6	0.0326	HSA-381119
2	Reactome Pathways	Sema3A PAK dependent Axon repulsion	FYN|CFL1	0.0344	HSA-399954
4	Reactome Pathways	Anchoring of the basal body to the plasma membrane	TUBB4A|PLK1|TUBA1A|TUBB4B	0.0346	HSA-5620912
20	Reactome Pathways	Post-translational protein modification	RCN1|ARF3|THBS1|RORA|TUBB4A|APP|TUBA1A|TUBA1C|DDB1|CDC25A|ARF4|CALR|TUBB4B|COPA|SPTAN1|PDIA6|TOP2B|SPTBN2|ARF1|PRKCSH	0.0393	HSA-597592
5	Reactome Pathways	S Phase	CDC25B|AKT3|CDC25A|WEE1|MCM3	0.0393	HSA-69242
2	Reactome Pathways	Synthesis of PIPs at the Golgi membrane	ARF3|ARF1	0.0409	HSA-1660514
2	Reactome Pathways	CREB phosphorylation through the activation of CaMKII	CAMK2B|CAMK2A	0.0409	HSA-442729
2	Reactome Pathways	Cell-extracellular matrix interactions	FLNC|FLNA	0.0409	HSA-446353
6	Reactome Pathways	HIV Infection	CCR5|LCK|FYN|PSIP1|XRCC5|ARF1	0.0428	HSA-162906
17	Reactome Pathways	Generic Transcription Pathway	THBS1|RORA|AKT3|TJP1|KIT|CDC25C|RORC|RARB|FAS|RXRG|DLL1|PTPN1|RXRB|G6PD|CAMK2B|CAMK2A|RARG	0.0436	HSA-212436
2	Reactome Pathways	RHO GTPases activate CIT	MYH9|MYH10	0.0436	HSA-5625900
4	Reactome Pathways	Post-translational protein phosphorylation	RCN1|APP|PDIA6|PRKCSH	0.0449	HSA-8957275
2	Reactome Pathways	Defective B4GALT7 causes EDS, progeroid type	SDC4|SDC1	0.0473	HSA-3560783
2	Reactome Pathways	Defective B3GAT3 causes JDSSDHD	SDC4|SDC1	0.0473	HSA-3560801
2	Reactome Pathways	Sema4D induced cell migration and growth-cone collapse	MYH9|MYH10	0.0473	HSA-416572
2	Reactome Pathways	Unblocking of NMDA receptors, glutamate binding and activation	CAMK2B|CAMK2A	0.0473	HSA-438066
2	Reactome Pathways	Defective B3GALT6 causes EDSP2 and SEMDJL1	SDC4|SDC1	0.0473	HSA-4420332
2	Reactome Pathways	Ras activation upon Ca2+ influx through NMDA receptor	CAMK2B|CAMK2A	0.0473	HSA-442982
5	Reactome Pathways	Signaling by Nuclear Receptors	RARB|RXRG|RXRB|BCL2|RARG	0.0473	HSA-9006931
3	Reactome Pathways	Assembly of collagen fibrils and other multimeric structures	ITGB4|PXDN|COL8A1	0.0497	HSA-2022090
3	Reactome Pathways	NCAM signaling for neurite out-growth	FYN|SPTAN1|SPTBN2	0.0497	HSA-375165
15	Reference publications	(2014) Interaction of membranelipid rafts with the cytoskeleton: impact on signaling and function: membranelipid rafts, mediators of cytoskeletal arrangement and cell signaling.	APP|ANXA5|NOS3|SDPR|LCK|CAV1|ANXA2|ANXA6|FYN|FN1|PTRF|DPP4|FLNA|ANXA1|CDC42	8.40E-11	PMID.23899502
16	Reference publications	(2018) A multi-step transcriptional cascade underlies vascular regeneration in vivo.	MMP2|LAMA4|PXDN|THBS1|COL8A1|VWF|ICAM1|FLT1|CSF1R|NOS3|BCL2L1|MMP14|DAB2|CDH5|CLIC4|ANXA1	1.08E-10	PMID.29615716
12	Reference publications	(2016) Prostate stromal cell proteomics analysis discriminates normal from tumour reactive stromal phenotypes.	MYH9|GAPDH|ANXA5|TUBA1A|CAV1|TPM4|FN1|FLNA|ANXA1|TPM2|CFL1|VIM	1.91E-09	PMID.26934553
11	Reference publications	(2016) Mechanism of Fibronectin Binding to Human Trabecular Meshwork Exosomes and Its Modulation by Dexamethasone.	MMP2|CCL2|ANXA5|SDPR|MMP14|ANXA2|ANXA6|FN1|DPP4|ANXA1|SDC1	1.91E-09	PMID.27783649
14	Reference publications	(2012) A proteomic view at T cell costimulation.	MYH9|GAPDH|ICAM1|ANXA5|TUBA1A|TUBA1C|BCL2L1|CALR|LCK|TUBB4B|FYN|TPM3|FLNA|LDHB	1.16E-08	PMID.22539942
13	Reference publications	(2013) Annexin A2 heterotetramer: structure and function.	FLT1|ANXA5|CALR|CDH5|ANXA2|ANXA6|FN1|TLR4|ANXA1|SDC1|FSCN1|CDC42|CFL1	1.16E-08	PMID.23519104
17	Reference publications	(2012) Quantitative proteomics of extracellular vesicles derived from human primary and metastatic colorectal cancer cells.	ITGB4|MYH9|EHD4|GAPDH|ENO1|ARF3|ICAM1|ANXA5|ITGA2|LCK|ANXA2|ANXA6|MYH10|ANXA1|FSCN1|MYO1B|VIM	1.16E-08	PMID.24009881
15	Reference publications	(2016) Secreted primary human malignant mesothelioma exosome signature reflects oncogenic cargo.	MMP2|ENO1|THBS1|TUBB4A|CCT2|MMP14|CALR|TUBB4B|ANXA2|ANXA6|FN1|DNM1|ANXA1|G6PD|VIM	1.16E-08	PMID.27605433
13	Reference publications	(2014) Quantification of pancreatic cancer proteome and phosphorylome: indicates molecular events likely contributing to cancer and activity of drug targets.	ITGB4|MYH9|GAPDH|ARHGAP31|ZYX|FLNC|FYN|FN1|MYH10|FLNA|CDC42|FLNB|TLN2	2.31E-08	PMID.24670416

**12**	**Reference publications**	**(2014) PPARGamma activation but not PPARGamma haplodeficiency affects proangiogenic potential of endothelial cells and bone marrow-derived progenitors.**	**MMP2|THBS1|VWF|NGEF|ICAM1|FLT1|KIT|NOS3|CCT2|MMP14|CAV1|FN1**	**2.31E-08**	**PMID.25361524**
12	Reference publications	(2016) ISN Forefronts Symposium 2015: Nuclear Receptors and Diabetic Nephropathy.	CCL2|RORA|ICAM1|RORC|RARB|NR1I2|PTGES|FYN|FN1|RXRG|RARG|NR1H4	2.39E-08	PMID.28932823
14	Reference publications	(2010) Comprehensive identification and modified-site mapping of S-nitrosylated targets in prostate epithelial cells.	ITGB4|GAPDH|ENO1|NOS3|FLNC|TUBB4B|ANXA2|FLNA|CLIC4|ANXA1|XRCC5|PDIA6|FLNB|VIM	4.86E-08	PMID.20140087
15	Reference publications	(2010) Molecular characterization of c-Ablc-Src kinase inhibitors targeted against murine tumour progenitor cells that express stem cell markers.	ITGB4|CDC25B|THBS1|KIT|PLK1|TUBA1A|BCL2L1|CDC25C|FLNC|LCK|CAV1|FYN|FN1|TLR4|VIM	5.83E-08	PMID.21152443
13	Reference publications	(2014) Plasma membrane proteomics of human breast cancer cell lines identifies potential targets for breast cancer diagnosis and treatment.	MYH9|ICAM1|FLT1|KIT|FLNC|TPM4|EPHA2|MYO1C|MYH10|TPM3|FLNA|TPM2|FLNB	8.25E-08	PMID.25029196
13	Reference publications	(2016) More Than Tiny Sacks: Stem Cell Exosomes as Cell-Free Modality for Cardiac Repair.	CCL2|GAPDH|ICAM1|KIT|ANXA5|NOS3|BCL2L1|ANXA2|ANXA6|FLNA|PTPN1|TLR4|ANXA1	1.14E-07	PMID.26838317
11	Reference publications	(2008) Proteomic profiling of neuromas reveals alterations in protein composition and local protein synthesis in hyper-excitable nerves.	ENO1|ANXA5|CKB|CCT2|CALR|SNCA|ANXA2|ANXA6|ANXA1|FSCN1|LDHB	1.84E-07	PMID.18700027
14	Reference publications	(2011) Proteomic analysis of endothelial cold-adaptation.	GAPDH|ENO1|THBS1|FERMT3|PLOD2|CCT2|CALR|FN1|FLNA|ANXA1|FSCN1|G6PD|CDC42|VIM	2.50E-07	PMID.22192797
10	Reference publications	(2009) Comparative proteomic analysis of malformed umbilical cords from somatic cell nuclear transfer-derived piglets: implications for early postnatal death.	HSPB1|VWF|FLT1|ANXA5|TPM4|ANXA2|ANXA1|TPM2|LDHB|CFL1	2.55E-07	PMID.19889237
10	Reference publications	(2011) Analysis of the myosin-II-responsive focal adhesion proteome reveals a role for Beta-Pix in negative regulation of focal adhesion maturation.	GAPDH|CAPN2|DAB2|ZYX|FLNC|CAV1|FN1|FLNA|FLNB|CFL1	2.55E-07	PMID.21423176
10	Reference publications	(2011) Potential Agents against Plasma Leakage.	ICAM1|TJP1|ZYX|CAV1|CDH5|FYN|FN1|SDC1|CDC42|CFL1	2.55E-07	PMID.22084722
10	Reference publications	(2012) Novel processed form of syndecan-1 shed from SCC-9 cells plays a role in cell migration.	MMP2|GAPDH|LAMA5|THBS1|ANXA5|MMP14|FN1|SDC4|ANXA1|SDC1	2.55E-07	PMID.22905270
10	Reference publications	(2012) The Cytotoxicity Mechanism of 6-Shogaol-Treated HeLa Human Cervical Cancer Cells Revealed by Label-Free Shotgun Proteomics and Bioinformatics Analysis.	MYH9|ENO1|ANXA5|CALR|CDC25C|TUBB4B|ANXA2|ANXA1|CFL1|VIM	2.55E-07	PMID.23243437
12	Reference publications	(2011) Pathophysiological mechanisms in antiphospholipid syndrome.	MMP2|CCL2|VWF|ICAM1|FLT1|ANXA5|ITGA2|NR1I2|ANXA2|ANXA6|TLR4|ANXA1	2.55E-07	PMID.23487578
10	Reference publications	(2014) Tumor-derived exosomes: A message delivery system for tumor progression.	MMP2|PDGFRA|KIT|ANXA5|BCL2L1|ANXA2|ANXA6|FN1|ANXA1|VIM	3.61E-07	PMID.24778765
10	Reference publications	(2016) Endothelial Mechanosignaling: Does One Sensor Fit All?	ICAM1|NOS3|CDH5|FYN|FN1|PTRF|SDC4|DNM1|SDC1|CDC42	4.24E-07	PMID.27027326
10	Reference publications	(2017) Differential Impact of Single-Dose Fe Ion and X-Ray Irradiation on Endothelial Cell Transcriptomic and Proteomic Responses.	CCL2|VWF|ICAM1|NOS3|ARF4|FLNC|CAV1|CDH5|FN1|PTPN1	4.24E-07	PMID.28993729
11	Reference publications	(2012) Comparative expression profiling of distinct T cell subsets undergoing oxidative stress.	ENO1|ANXA5|TUBA1A|LCK|TPM4|TPM3|GSTO1|FLNA|TPM2|G6PD|CFL1	4.78E-07	PMID.22911781
10	Reference publications	(2015) Novel human-derived extracellular matrix induces in vitro and in vivo vascularization and inhibits fibrosis.	MMP2|LAMA4|LAMA5|THBS1|ICAM1|ANXA5|ANXA2|ANXA6|FN1|ANXA1	4.78E-07	PMID.25725553
11	Reference publications	(2007) The extracellular matrix and blood vessel formation: not just a scaffold.	MMP2|LAMA4|THBS1|VWF|TJP1|FLT1|MMP14|CDH5|FN1|SDC4|SDC1	5.30E-07	PMID.17488472
8	Reference publications	(2016) ARHGAP10, downregulated in ovarian cancer, suppresses tumorigenicity of ovarian cancer cells.	GAPDH|PLEK|ANXA5|PLK1|FN1|CDC42|ARF1|MCM3	5.30E-07	PMID.27010858
9	Reference publications	(2016) Proteome analysis of human amniotic mesenchymal stem cells (hA-MSCs) reveals impaired antioxidant ability, cytoskeleton and metabolic functionality in maternal obesity.	GAPDH|ENO1|HSPB1|ICAM1|ANXA5|ANXA2|ANXA6|ANXA1|VIM	5.30E-07	PMID.27125468
10	Reference publications	(2016) Progressive muscle proteome changes in a clinically relevant pig model of Duchenne muscular dystrophy.	ANXA5|FLNC|CAV1|TPM4|ANXA2|ANXA6|TPM3|ANXA1|TPM2|VIM	5.30E-07	PMID.27634466
10	Reference publications	(2009) Molecular mechanisms of endothelial hyperpermeability: implications in inflammation.	MMP2|ICAM1|TJP1|FLT1|NR1I2|LCK|CAV1|CDH5|FYN|CDC42	6.29E-07	PMID.19563700
11	Reference publications	(2011) Blood-brain barrier pathophysiology in traumatic brain injury.	MMP2|CCL2|THBS1|ICAM1|TJP1|FLT1|APP|NR1I2|CDH5|TLR4|ABCB1	6.29E-07	PMID.22299022
11	Reference publications	(2015) Neutrophil migration under normal and sepsis conditions.	MYH9|MMP2|CCL2|ICAM1|TJP1|KIT|NOS3|CAV1|CDH5|TLR4|ANXA1	6.29E-07	PMID.25567338
13	Reference publications	(2016) Extracellular Vesicles in Physiology, Pathology, and Therapy of the Immune and Central Nervous System, with Focus on Extracellular Vesicles Derived from Mesenchymal Stem Cells as Therapeutic Tools.	MMP2|GAPDH|ICAM1|APP|CCR5|ANXA5|SNCA|ANXA2|ANXA6|FN1|ANXA1|CDC42|CFL1	6.29E-07	PMID.27199663
8	Reference publications	(2016) Quantitative proteomics reveals FLNC as a potential progression marker for the development of hepatocellular carcinoma.	GAPDH|HSPB1|ITGA2|FLNC|CAV1|FLNA|CDC42|FLNB	6.29E-07	PMID.27626164
11	Reference publications	(2017) Multiple therapeutic effect of endothelial progenitor cell regulated by drugs in diabetes and diabetes related disorder.	MMP2|CCL2|VWF|ICAM1|FLT1|KIT|NOS3|CDH5|DPP4|DLL1|SDC4	6.29E-07	PMID.28859673
16	Reference publications	(2012) Amniotic fluid and amniotic membrane stem cells: marker discovery.	CCL2|THBS1|VWF|ICAM1|KIT|PLK1|CAV1|TPM4|ANXA2|FN1|TGM2|TPM3|CLIC4|TPM2|CFL1|VIM	6.56E-07	PMID.22701492
14	Reference publications	(2010) Comparative proteomics of human embryonic stem cells and embryonal carcinoma cells.	GAPDH|MT2A|HSPB1|APP|ARF4|ZYX|FLNC|CAV1|FLNA|ANXA1|PSIP1|LDHB|CDC42|FLNB	6.99E-07	PMID.20104618
13	Reference publications	(2014) Discovery of new glomerular disease-relevant genes by translational profiling of podocytes in vivo.	MYH9|GAPDH|PLEK|THBS1|VWF|ITGA2|FN1|MYH10|SDC4|TLR4|MYO1B|FLNB|CFL1	7.09E-07	PMID.24940801

10	Reference publications	(2015) Exosomes serve as tumour markers for personalized diagnostics owing to their important role in cancer metastasis.	CCL2|VWF|ICAM1|TJP1|ANXA5|CDH5|ANXA2|ANXA6|FN1|ANXA1	7.64E-07	PMID.26095380
11	Reference publications	(....) Systems biology from virus to humans.	CCL2|GAPDH|ENO1|ANXA5|CAV1|ANXA2|ANXA6|FLNA|TLR4|ANXA1|CFL1	7.64E-07	PMID.26269748
12	Reference publications	(2014) Rho GAPs and GEFs: controling switches in endothelial cell adhesion.	PLEK|VWF|ARHGAP31|ICAM1|TJP1|CDH5|FN1|ARHGEF26|EPHA2|ARHGEF15|FLNA|CDC42	8.12E-07	PMID.24622613
14	Reference publications	(2016) Protein Co-Expression Analysis as a Strategy to Complement a Standard Quantitative Proteomics Approach: Case of a Glioblastoma Multiforme Study.	MYH9|ANXA5|CALR|SNCA|FN1|SFPQ|FLNA|DNM1|ANXA1|XRCC5|CAMK2A|PDIA6|VIM|PRKCSH	8.12E-07	PMID.27571357
10	Reference publications	(2011) Proteomic and transcriptomic analysis of heart failure due to volume overload in a rat aorto-caval fistula model provides support for new potential therapeutic targets - monoamine oxidase A and transglutaminase 2.	GAPDH|ENO1|HSPB1|ANXA5|CKB|CALR|ANXA2|ANXA6|TGM2|ANXA1	8.48E-07	PMID.22078724
9	Reference publications	(2010) Endocytosis of nanomedicines.	ICAM1|APP|NOS3|CALR|LCK|CAV1|FN1|PTRF|CDC42	1.34E-06	PMID.20226220
9	Reference publications	(2010) The PI3K p110alpha isoform regulates endothelial adherens junctions via Pyk2 and Rac1.	CCL2|GAPDH|PLEK|ICAM1|TJP1|CDH5|FYN|FN1|CDC42	1.34E-06	PMID.20308428
9	Reference publications	(2011) The physics of cancer: the role of physical interactions and mechanical forces in metastasis.	ICAM1|MMP14|ZYX|TPM4|FN1|TPM3|TPM2|FSCN1|SUN1	1.34E-06	PMID.21701513
11	Reference publications	(2011) Polymicrobial periodontal pathogen transcriptomes in calvarial bone and soft tissue.	MMP2|CCL2|THBS1|VWF|APP|MMP14|CDH5|FN1|SDC4|SDC1|LTB4R	1.34E-06	PMID.21896157
10	Reference publications	(2016) Stress-Induced Premature Senescence of Endothelial and Endothelial Progenitor Cells.	VWF|ICAM1|FLT1|KIT|NOS3|TENC1|CDH5|FN1|TGM2|DLL1	1.34E-06	PMID.27451101
**9**	**Reference publications**	**(2017) Talin Modulation by a Synthetic N-Acylurea Derivative Reduces Angiogenesis in Human Endothelial Cells.**	**GAPDH|VWF|ICAM1|FLT1|ANXA5|NOS3|CDH5|FN1|TLN2**	**1.34E-06**	**PMID.28117756**
13	Reference publications	(2017) Distinct proteome pathology of circulating microparticles in systemic lupus erythematosus.	MYH9|GAPDH|PLEK|ENO1|CAPN2|ANXA5|ITGA2|CALR|ANXA2|ANXA1|G6PD|CDC42|ARF1	1.34E-06	PMID.28649187
9	Reference publications	(1995) Identification of proteins that are abnormally regulated in differentiated cultured human keratinocytes.	ENO1|HSPB1|ANXA5|TPM4|ANXA2|ANXA6|TPM3|ANXA1|TPM2	1.34E-06	PMID.8824783
10	Reference publications	(2009) The role of tenascin-C in tissue injury and tumorigenesis.	MMP2|CCL2|THBS1|ICAM1|APP|ANXA2|FN1|FAS|SDC4|TLR4	1.38E-06	PMID.19838819
8	Reference publications	(2016) The protein kinase promiscuities in the cancer-preventive mechanisms of NSAIDs.	FLT1|CSF1R|KIT|FYN|EPHA2|RXRG|RXRB|RARG	1.42E-06	PMID.25714784
9	Reference publications	(2017) Perspective Insights of Exosomes in Neurodegenerative Diseases: A Critical Appraisal.	GAPDH|ICAM1|APP|ANXA5|SNCA|ANXA2|ANXA6|DPP4|ANXA1	1.42E-06	PMID.29033828
9	Reference publications	(2018) Proteomic Analysis of Secretomes of Oncolytic Herpes Simplex Virus-Infected Squamous Cell Carcinoma Cells.	MMP2|THBS1|CALR|FLNC|ANXA2|FN1|COPA|ANXA1|FSCN1	1.42E-06	PMID.29360750
16	Reference publications	(2009) Proteomic analysis of blastema formation in regenerating axolotl limbs.	MYH9|EHD4|HSPB1|PXDN|ANXA5|CCT2|PLK1|TUBB4B|CDH5|ANXA2|ANXA6|FN1|MYO1C|ANXA1|PDIA6|FLNB	1.51E-06	PMID.19948009
11	Reference publications	(2013) The hCMECD3 cell line as a model of the human blood brain barrier.	CCL2|VWF|ICAM1|TJP1|ANXA5|NR1I2|CDH5|ANXA2|ANXA6|ANXA1|ABCB1	1.51E-06	PMID.23531482
10	Reference publications	(2016) Mining kidney toxicogenomic data by using gene co-expression modules.	CCL2|ICAM1|LCK|MAP4K4|ANXA2|FN1|FLNA|TLR4|SDC1|VIM	1.51E-06	PMID.27724849
10	Reference publications	(2017) Caveolin-1 and Caveolin-2 Can Be Antagonistic Partners in Inflammation and Beyond.	MMP2|CCL2|NOS3|MMP14|CAV1|CDH5|FN1|PTRF|TLR4|CDC42	1.51E-06	PMID.29250058
9	Reference publications	(2017) The molecular biology in wound healing & non-healing wound.	MMP2|CCL2|VWF|ICAM1|NOS3|MMP14|CDH5|FN1|PRKCSH	1.55E-06	PMID.28712679
8	Reference publications	(2013) Interaction of proteins identified in human thyroid cells.	MYH9|CAPN2|ANXA5|ANXA2|FN1|FLNA|ANXA1|CFL1	1.65E-06	PMID.23303277
8	Reference publications	(2015) Identification of proteins responsible for adriamycin resistance in breast cancer cells using proteomics analysis.	HSPB1|ANXA5|ANXA2|ANXA6|ANXA1|G6PD|LDHB|ABCB1	1.65E-06	PMID.25818003
11	Reference publications	(2015) Integrin traffic - the update.	ARF4|MMP14|DAB2|ZYX|CAV1|MAP4K4|FN1|SDC4|SDC1|CDC42|NUMB	1.81E-06	PMID.25663697
9	Reference publications	(2018) STAT3-RXR-Nrf2 activates systemic redox and energy homeostasis upon steep decline in pO2 gradient.	CCL2|GAPDH|PPP3R1|CAPN2|ANXA5|ANXA2|ANXA6|ANXA1|VIM	1.81E-06	PMID.29078168
12	Reference publications	(2018) Exosome-Based Cell-Cell Communication in the Tumor Microenvironment.	MMP2|CCL2|ICAM1|TJP1|KIT|ANXA5|BCL2L1|ANXA2|FN1|EPHA2|TLR4|SDC1	1.94E-06	PMID.29515996
8	Reference publications	(2015) Microparticles as novel biomarkers and therapeutic targets in coronary heart disease.	MMP2|CCL2|VWF|ICAM1|FLT1|CSF1R|ANXA5|NOS3	2.00E-06	PMID.25591573
10	Reference publications	(2013) Signaling pathways in exosomes biogenesis, secretion and fate.	GAPDH|ENO1|ICAM1|APP|ANXA5|SNCA|ANXA2|ANXA6|ANXA1|CFL1	2.03E-06	PMID.24705158
9	Reference publications	(2016) Genome-wide analysis of Musashi-2 targets reveals novel functions in governing epithelial cell migration.	ITGB4|GAPDH|LAMA4|THBS1|ANXA5|ITGA2|DLL1|FLNB|NUMB	2.05E-06	PMID.27034466
9	Reference publications	(2017) Protein and chemotherapy profiling of extracellular vesicles harvested from therapeutic induced senescent triple negative breast cancer cells.	GAPDH|LAMA5|ANXA5|ANXA2|ANXA6|FN1|ANXA1|NR1H4|ABCB1	2.44E-06	PMID.28991260
8	Reference publications	(2005) Proteomic analysis of cellular response to osmotic stress in thick ascending limb of Henle's loop (TALH) cells.	GAPDH|ENO1|ANXA5|CALR|TPM4|ANXA2|ANXA6|ANXA1	2.45E-06	PMID.15975915
12	Reference publications	(2009) Inhibitor of DASH proteases affects expression of adhesion molecules in osteoclasts and reduces myeloma growth and bone disease.	ITGB4|MMP2|GAPDH|LAMA4|THBS1|COL8A1|ICAM1|ITGA2|MMP14|FN1|DPP4|SDC1	2.45E-06	PMID.19388929
8	Reference publications	(2012) Cytoskeleton as an emerging target of anthrax toxins.	PLEK|HSPB1|TJP1|FLT1|CDH5|FYN|CDC42|CFL1	2.45E-06	PMID.22474568
10	Reference publications	(2011) Quantitative proteomic and interaction network analysis of cisplatin resistance in HeLa cells.	ANXA5|DDB1|CALR|ANXA2|ANXA6|GSTO1|ANXA1|XRCC5|G6PD|PDIA6	2.51E-06	PMID.21637840
10	Reference publications	(2016) The biology of extracellular vesicles with focus on platelet microparticles and their role in cancer development and progression.	GAPDH|CCR5|ANXA5|ITGA2|MMP14|ANXA2|ANXA6|DPP4|ANXA1|ABCB1	2.51E-06	PMID.27629289

9	Reference publications	(2016) Differentiation of Human Embryonic Stem Cells to Endothelial Progenitor Cells on Laminins in Defined and Xeno-free Systems.	MMP2|GAPDH|LAMA4|VWF|FLT1|ITGA2|MMP14|CDH5|FN1	2.72E-06	PMID.27693424
12	Reference publications	(2015) Human miR-221222 in Physiological and Atherosclerotic Vascular Remodeling.	MMP2|CCL2|ICAM1|KIT|NOS3|CAV1|CDH5|TPM4|TPM3|TLR4|TPM2|CDC42	2.76E-06	PMID.26221589
9	Reference publications	(2016) A genome landscape of SRSF3-regulated splicing events and gene expression in human osteosarcoma U2OS cells.	GAPDH|CDC25B|MELK|PLK1|MAP4K4|CDH5|ANXA1|G6PD|NUMB	3.18E-06	PMID.26704980
9	Reference publications	(2016) Functional and Biological Role of Endothelial Precursor Cells in Tumour Progression: A New Potential Therapeutic Target in Haematological Malignancies.	CCL2|VWF|FLT1|KIT|NOS3|JAG2|CDH5|FN1|DLL1	3.18E-06	PMID.26788072
**10**	**Reference publications**	**(2017) Platelets and cancer angiogenesis nexus.**	**MMP2|THBS1|VWF|ICAM1|FLT1|KIT|ITGA2|NOS3|MMP14|FN1**	**3.18E-06**	**PMID.28681240**
**8**	**Reference publications**	**(2010) PPARalpha is essential for microparticle-induced differentiation of mouse bone marrow-derived endothelial progenitor cells and angiogenesis.**	**THBS1|ICAM1|FLT1|CSF1R|ANXA5|NOS3|CDH5|FN1**	**3.51E-06**	**PMID.20811625**
10	Reference publications	(2012) Endothelial progenitor cells in atherosclerosis.	CCL2|VWF|ICAM1|FLT1|KIT|CCR5|NOS3|CDH5|FN1|DPP4	3.51E-06	PMID.22652782
12	Reference publications	(2015) Endothelial progenitor cells in ischemic stroke: an exploration from hypothesis to therapy.	MMP2|CCL2|THBS1|VWF|ICAM1|FLT1|KIT|CCR5|NOS3|FN1|DPP4|CDC42	3.51E-06	PMID.25888494
11	Reference publications	(2013) Systems biology analysis of Brucella infected Peyer's patch reveals rapid invasion with modest transient perturbations of the host transcriptome.	MYH9|GAPDH|PRKCG|AKT3|FLT1|KIT|BCL2L1|MYH10|SPTAN1|SDC1|CDC42	3.54E-06	PMID.24349118
9	Reference publications	(2014) Comparative proteomic profiling of pancreatic ductal adenocarcinoma cell lines.	ITGB4|MYH9|GAPDH|CAV1|FN1|FLNA|SDC1|FLNB|VIM	3.54E-06	PMID.25518923
9	Reference publications	(2016) Microarray analyses to quantify advantages of 2D and 3D hydrogel culture systems in maintaining the native valvular interstitial cell phenotype.	MMP2|GAPDH|LAMA4|KIT|ITGA2|MMP14|FN1|TPM2|FLNB	3.54E-06	PMID.26433490
10	Reference publications	(2013) Quantitative proteomic analysis of gingival crevicular fluid in different periodontal conditions.	MYH9|HSPB1|ANXA5|TPM4|ANXA2|ANXA6|TPM3|ANXA1|TPM2|CFL1	3.78E-06	PMID.24098404
11	Reference publications	(2011) Acute lung injury: how macrophages orchestrate resolution of inflammation and tissue repair.	ITGB4|CCL2|THBS1|ICAM1|TJP1|KIT|CDH5|FN1|TLR4|ANXA1|LTB4R	3.86E-06	PMID.22566854
11	Reference publications	(2012) Motility, survival, and proliferation.	PLEK|HSPB1|BCL2L1|CAV1|TPM4|FN1|FAS|TPM3|TPM2|CDC42|CFL1	3.86E-06	PMID.23728975
9	Reference publications	(2013) A bright monomeric green fluorescent protein derived from Branchiostoma lanceolatum.	CCL2|TJP1|CALR|ZYX|CAV1|CDH5|FLNA|FSCN1|VIM	3.95E-06	PMID.23524392
12	Reference publications	(2009) Bone marrow microenvironment and the identification of new targets for myeloma therapy.	MMP2|HSPB1|ICAM1|FLT1|KIT|BCL2L1|JAG2|CAV1|FN1|DLL1|SDC1|XRCC5	4.02E-06	PMID.18843284
8	Reference publications	(2012) The tipping point for combination therapy: cancer vaccines with radiation, chemotherapy, or targeted small molecule inhibitors.	BCL2L2|ICAM1|FLT1|KIT|BCL2L1|CALR|FAS|TLR4	4.02E-06	PMID.22595055
8	Reference publications	(2012) The Rho-guanine nucleotide exchange factor Trio controls leukocyte transendothelial migration by promoting docking structure formation.	ICAM1|FLNC|CDH5|FN1|ARHGEF26|FLNA|CDC42|FLNB	4.02E-06	PMID.22696684
8	Reference publications	(2016) A humanized anti-CD26 monoclonal antibody inhibits cell growth of malignant mesothelioma via retarded G2M cell cycle transition.	GAPDH|CDC25B|KIT|CDC25A|CDC25C|CAV1|FN1|DPP4	4.02E-06	PMID.27134571
14	Reference publications	(2015) Emerging risk biomarkers in cardiovascular diseases and disorders.	MMP2|CCL2|HSPB1|VWF|ICAM1|CCR5|ANXA5|CDH5|ANXA2|ANXA6|DPP4|TLR4|ANXA1|ARF1	4.06E-06	PMID.25949827
9	Reference publications	(2011) Purification of tropomyosin, paramyosin, actin, tubulin, troponin and kinases for chemiproteomics and its application to different scientific fields.	MYH9|ANXA5|TPM4|ANXA2|ANXA6|MYH10|TPM3|ANXA1|TPM2	4.40E-06	PMID.21876731
10	Reference publications	(2010) Proteomic study of activated Taenia solium oncospheres.	GAPDH|CSF1R|ANXA5|TPM4|ANXA2|ANXA6|FN1|TPM3|ANXA1|TPM2	4.45E-06	PMID.20144663
7	Reference publications	(2017) SR-BI Mediated Transcytosis of HDL in Brain Microvascular Endothelial Cells Is Independent of Caveolin, Clathrin, and PDZK1.	GAPDH|TJP1|NOS3|CAV1|CDH5|PTPN1|CDC42	4.59E-06	PMID.29163190
8	SMART Domains	c4 zinc finger in nuclear hormone receptors	RORA|RORC|RARB|NR1I2|RXRG|RXRB|RARG|NR1H4	1.69E-06	SM00399
8	SMART Domains	Ligand binding domain of hormone receptors	RORA|RORC|RARB|NR1I2|RXRG|RXRB|RARG|NR1H4	1.69E-06	SM00430
13	SMART Domains	Serine/Threonine protein kinases, catalytic domain	MAP2K5|PRKCG|AKT3|MELK|PLK1|BRSK1|PRKCH|MAP4K4|SLK|CAMK2B|CAMK2A|DYRK1A|CAMK1D	2.20E-05	SM00220
6	SMART Domains	Phosphotyrosine-binding domain, phosphotyrosine-interaction (PI) domain	DAB2|TENC1|RGS12|TBC1D4|NUMB|APBA2	2.20E-05	SM00462
7	SMART Domains	Tyrosine kinase, catalytic domain	PDGFRA|FLT1|CSF1R|KIT|LCK|FYN|EPHA2	3.00E-04	SM00219
4	SMART Domains	Annexin repeats	ANXA5|ANXA2|ANXA6|ANXA1	3.00E-04	SM00335
3	SMART Domains	BH4 Bcl-2 homology region 4	BCL2L2|BCL2L1|BCL2	3.20E-04	SM00265
6	SMART Domains	Calponin homology domain	CNN2|FLNC|FLNA|CNN3|FLNB|SPTBN2	3.70E-04	SM00033
4	SMART Domains	Tubulin/FtsZ family, C-terminal domain	TUBB4A|TUBA1A|TUBA1C|TUBB4B	9.00E-04	SM00865
4	SMART Domains	Tubulin/FtsZ family, GTPase domain	TUBB4A|TUBA1A|TUBA1C|TUBB4B	9.40E-04	SM00864
3	SMART Domains	Filamin-type immunoglobulin domains	FLNC|FLNA|FLNB	0.0016	SM00557
4	SMART Domains	von Willebrand factor (vWF) type C domain	PXDN|THBS1|VWF|JAG2	0.0028	SM00214
3	SMART Domains	BCL (B-Cell lymphoma)	BCL2L2|BCL2L1|BCL2	0.0028	SM00337
4	SMART Domains	Myosin. Large ATPases.	MYH9|MYO1C|MYH10|MYO1B	0.0034	SM00242
2	SMART Domains	amyloid A4	APLP1|APP	0.0053	SM00006
9	SMART Domains	Pleckstrin homology domain.	ARHGEF5|PLEK|AKT3|NGEF|FERMT3|DOK4|ARHGEF26|DNM1|SPTBN2	0.0053	SM00233
2	SMART Domains	delta serrate ligand	JAG2|DLL1	0.0069	SM00051
3	SMART Domains	ARF-like small GTPases	ARF3|ARF4|ARF1	0.0074	SM00177
3	SMART Domains	Rhodanese Homology Domain	CDC25B|CDC25A|CDC25C	0.0074	SM00450
4	SMART Domains	Guanine nucleotide exchange factor for Rho/Rac/Cdc42-like GTPases	ARHGEF5|NGEF|ARHGEF26|ARHGEF15	0.0152	SM00325
7	SMART Domains	Src homology 3 domains	ARHGEF5|NGEF|TJP1|LCK|FYN|ARHGEF26|SPTAN1	0.0152	SM00326
4	SMART Domains	Short calmodulin-binding motif containing conserved Ile and Gln residues.	MYH9|MYO1C|MYH10|MYO1B	0.0319	SM00015
4	SMART Domains	von Willebrand factor (vWF) type A domain	ITGB4|VWF|ITGA2|XRCC5	0.0331	SM00327
4	SMART Domains	Immunoglobulin like	PDGFRA|ICAM1|CSF1R|KIT	0.0356	SM00410

<table>
<tr><td>2</td><td>SMART Domains</td><td>Fibronectin type 2 domain</td><td>MMP2|FN1</td><td>0.0357</td><td>SM00059</td></tr>
<tr><td>2</td><td>SMART Domains</td><td>putative band 4.1 homologues' binding motif</td><td>SDC4|SDC1</td><td>0.0357</td><td>SM00294</td></tr>
<tr><td>2</td><td>SMART Domains</td><td>Phosphotyrosine-binding domain</td><td>DOK4|TLN2</td><td>0.0357</td><td>SM01244</td></tr>
<tr><td>2</td><td>SMART Domains</td><td>Prolyl 4-hydroxylase alpha subunit homologues.</td><td>PLOD1|PLOD2</td><td>0.0402</td><td>SM00702</td></tr>
<tr><td>130</td><td>UniProt Keywords</td><td>Phosphoprotein</td><td>RCN1|ARHGEF5|MAP2K5|ITGB4|MYH9|MMP2|EHD4|GAPDH|GNB4|PPP3R1|PLEK|ENO1|MT2A|CDC25B|HSPB1|BCL2L2|PXDN|PDGFRA|RORA|DNAJB6|CNN2|PRKCG|AKT3|NGEF|TUBB4A|ARHGAP31|CAD|ADD1|ICAM1|FERMT3|TJP1|FLT1|PLOD2|APP|CSF1R|KIT|CCR5|ANXA5|NOS3|MELK|CKB|CCT2|PLK1|TUBA1A|TUBA1C|DDB1|BCL2L1|CDC25A|SDPR|ARF4|MMP14|BRSK1|DAB2|TENC1|NQO1|CDC25C|ZYX|FLNC|JAG2|PRKCH|RARB|LCK|SNCA|CAV1|RGS12|TUBB4B|USP1|MAP4K4|CDH5|DOK4|PTMA|ANXA2|ANXA6|FYN|FAS|ARHGEF26|PTRF|SFPQ|EPHA2|MYO1C|MYH10|ARHGEF15|TGM2|DLL1|ARHGAP30|COPA|TPM3|GSTO1|SLK|FLNA|CNN3|PTPN1|SPTAN1|DNM1|CLIC4|ANXA1|TBC1D4|PSIP1|SDC1|FSCN1|XRCC5|MYO1B|G6PD|PPP3CA|CAMK2B|LDHB|LTB4R|BCL2|CAMK2A|DYRK1A|SEPT7|PAICS|CDC42|SUN1|PDIA6|TOP2B|WEE1|FLNB|PBK|SPTBN2|CFL1|VIM|NR1H4|NUMB|APBA2|TLN2|PRKCSH|ABCB1|CAMK1D|MCM3</td><td>1.12E-20</td><td>KW-0597</td></tr>
<tr><td>15</td><td>UniProt Keywords</td><td>Calmodulin-binding</td><td>MYH9|CNN2|ADD1|TJP1|NOS3|FAS|MYO1C|MYH10|CNN3|SPTAN1|MYO1B|PPP3CA|CAMK2B|CAMK2A|CAMK1D</td><td>1.28E-09</td><td>KW-0112</td></tr>
<tr><td>45</td><td>UniProt Keywords</td><td>Nucleotide-binding</td><td>MAP2K5|MYH9|EHD4|ARF3|PDGFRA|PRKCG|AKT3|TUBB4A|CAD|FLT1|CSF1R|KIT|MELK|CKB|CCT2|PLK1|TUBA1A|TUBA1C|ARF4|BRSK1|PRKCH|LCK|TUBB4B|MAP4K4|FYN|EPHA2|MYO1C|MYH10|SLK|DNM1|XRCC5|MYO1B|CAMK2B|CAMK2A|DYRK1A|SEPT7|PAICS|CDC42|TOP2B|WEE1|PBK|ARF1|ABCB1|CAMK1D|MCM3</td><td>1.28E-09</td><td>KW-0547</td></tr>
<tr><td>64</td><td>UniProt Keywords</td><td>Acetylation</td><td>MAP2K5|MYH9|EHD4|GAPDH|GNB4|PLEK|ENO1|MT2A|HSPB1|BCL2L2|CNN2|AKT3|CAD|ADD1|CAPN2|ANXA5|CCT2|PLK1|TUBA1A|TUBA1C|DDB1|SDPR|DAB2|CALR|CDC25C|STXBP6|ZYX|SNCA|CAV1|TUBB4B|MAP4K4|PTMA|ANXA2|ANXA6|PTRF|SFPQ|MYO1C|MYH10|TGM2|TPM3|GSTO1|FLNA|CNN3|PTPN1|SPTAN1|CLIC4|ANXA1|TBC1D4|FSCN1|XRCC5|G6PD|PPP3CA|LDHB|SEPT7|PAICS|TOP2B|FLNB|PBK|SPTBN2|CFL1|ARF1|VIM|NR1H4|MCM3</td><td>1.73E-09</td><td>KW-0007</td></tr>
<tr><td>16</td><td>UniProt Keywords</td><td>Actin-binding</td><td>MYH9|CNN2|ADD1|FLNC|MYO1C|MYH10|TPM3|FLNA|CNN3|SPTAN1|FSCN1|MYO1B|CAMK2B|FLNB|SPTBN2|CFL1</td><td>8.64E-08</td><td>KW-0009</td></tr>
<tr><td>49</td><td>UniProt Keywords</td><td>Ubl conjugation</td><td>MYH9|GAPDH|ENO1|PDGFRA|RORA|PRKCG|AKT3|ICAM1|FLT1|APP|CSF1R|KIT|ANXA5|CCT2|PLK1|DDB1|BCL2L1|CDC25A|NQO1|FLNC|SNCA|CAV1|RGS12|USP1|PTMA|ANXA2|PTRF|SFPQ|EPHA2|ARHGEF15|DLL1|FLNA|ANXA1|PSIP1|FSCN1|XRCC5|MYO1B|BCL2|SUN1|RARG|TOP2B|WEE1|FLNB|PBK|CFL1|VIM|NR1H4|NUMB|CAMK1D</td><td>8.64E-08</td><td>KW-0832</td></tr>
<tr><td>65</td><td>UniProt Keywords</td><td>Disease</td><td>ARHGEF5|PLOD1|ITGB4|MYH9|CYP24A1|MMP2|APLP1|LAMA4|GNB4|HSPB1|PXDN|PDGFRA|VWF|RORA|DNAJB6|PRKCG|AKT3|TUBB4A|CAD|FERMT3|PLOD2|APP|CSF1R|KIT|CCR5|TUBA1A|MMP14|DAB2|RORC|FLNC|RARB|CYP2R1|LCK|SNCA|CAV1|TUBB4B|FYN|FAS|PTRF|EPHA2|MYH10|COPA|TPM3|FLNA|SPTAN1|DNM1|TLR4|TBC1D4|XRCC5|G6PD|PPP3CA|CAMK2B|LDHB|BCL2|CAMK2A|DYRK1A|CDC42|TOP2B|FLNB|SPTBN2|ARF1|VIM|NR1H4|TLN2|PRKCSH</td><td>8.64E-08</td><td>KW-9995</td></tr>
<tr><td>35</td><td>UniProt Keywords</td><td>ATP-binding</td><td>MAP2K5|MYH9|EHD4|PDGFRA|PRKCG|AKT3|CAD|FLT1|CSF1R|KIT|MELK|CKB|CCT2|PLK1|BRSK1|PRKCH|LCK|MAP4K4|FYN|EPHA2|MYO1C|MYH10|SLK|XRCC5|MYO1B|CAMK2B|CAMK2A|DYRK1A|PAICS|TOP2B|WEE1|PBK|ABCB1|CAMK1D|MCM3</td><td>1.12E-07</td><td>KW-0067</td></tr>
<tr><td>23</td><td>UniProt Keywords</td><td>Kinase</td><td>MAP2K5|PDGFRA|PRKCG|AKT3|FLT1|CSF1R|KIT|MELK|CKB|PLK1|BRSK1|PRKCH|LCK|MAP4K4|FYN|EPHA2|SLK|CAMK2B|CAMK2A|DYRK1A|WEE1|PBK|CAMK1D</td><td>1.56E-07</td><td>KW-0418</td></tr>
<tr><td>76</td><td>UniProt Keywords</td><td>Cytoplasm</td><td>ARHGEF5|MYH9|MMP2|APLP1|GAPDH|RBP1|PPP3R1|ENO1|CDC25B|HSPB1|ARF3|DNAJB6|PRKCG|AKT3|NGEF|TUBB4A|CAD|ADD1|FLT1|APP|KIT|CAPN2|NOS3|CKB|CCT2|PLK1|TUBA1A|TUBA1C|DDB1|BCL2L1|SDPR|MMP14|BRSK1|DAB2|NQO1|CALR|STXBP6|ZYX|FLNC|PRKCH|RARB|LCK|SNCA|RGS12|TUBB4B|MAP4K4|ANXA6|FYN|PTRF|SFPQ|MYO1C|COPA|TPM3|GSTO1|SLK|FLNA|SPTAN1|DNM1|CLIC4|ANXA1|TBC1D4|PIR|FSCN1|PPP3CA|CAMK2B|LDHB|SEPT7|CDC42|TOP2B|FLNB|SPTBN2|CFL1|ARF1|VIM|TLN2|CAMK1D</td><td>1.80E-07</td><td>KW-0963</td></tr>
<tr><td>53</td><td>UniProt Keywords</td><td>Disease mutation</td><td>PLOD1|ITGB4|MYH9|CYP24A1|MMP2|LAMA4|GNB4|HSPB1|PXDN|VWF|RORA|DNAJB6|PRKCG|AKT3|TUBB4A|CAD|FERMT3|PLOD2|APP|CSF1R|KIT|TUBA1A|MMP14|RORC|FLNC|RARB|CYP2R1|LCK|SNCA|CAV1|TUBB4B|FAS|EPHA2|MYH10|COPA|TPM3|FLNA|SPTAN1|DNM1|G6PD|PPP3CA|LDHB|BCL2|CAMK2A|CDC42|TOP2B|FLNB|SPTBN2|ARF1|VIM|NR1H4|TLN2|PRKCSH</td><td>7.32E-07</td><td>KW-0225</td></tr>
<tr><td>10</td><td>UniProt Keywords</td><td>Tyrosine-protein kinase</td><td>MAP2K5|PDGFRA|FLT1|CSF1R|KIT|LCK|FYN|EPHA2|DYRK1A|WEE1</td><td>1.40E-06</td><td>KW-0829</td></tr>
<tr><td>26</td><td>UniProt Keywords</td><td>Methylation</td><td>ARHGEF5|MYH9|GAPDH|RBP1|HSPB1|BCL2L2|RORA|DNAJB6|TUBA1A|TUBA1C|BRSK1|TENC1|ZYX|FLNC|RGS12|SFPQ|MYO1C|MYH10|COPA|CNN3|DNM1|RXRB|TBC1D4|CDC42|RARG|NR1H4</td><td>3.38E-06</td><td>KW-0488</td></tr>
<tr><td>29</td><td>UniProt Keywords</td><td>Cytoskeleton</td><td>MYH9|GAPDH|CDC25B|HSPB1|TUBB4A|ADD1|NOS3|PLK1|TUBA1A|TUBA1C|BCL2L1|BRSK1|ZYX|FLNC|TUBB4B|TPM3|FLNA|SPTAN1|DNM1|CLIC4|FSCN1|CAMK2B|SEPT7|CDC42|FLNB|SPTBN2|CFL1|VIM|TLN2</td><td>5.71E-06</td><td>KW-0206</td></tr>
<tr><td>22</td><td>UniProt Keywords</td><td>Cell junction</td><td>ARHGEF5|ITGB4|PRKCG|ARHGAP31|FERMT3|TJP1|BCL2L1|BRSK1|TENC1|ZYX|SNCA|RGS12|CDH5|EPHA2|DPP4|DLL1|CLIC4|FSCN1|CAMK2B|CAMK2A|ARF1|TLN2</td><td>5.84E-06</td><td>KW-0965</td></tr>
<tr><td>17</td><td>UniProt Keywords</td><td>Cell adhesion</td><td>ITGB4|MYH9|APLP1|LAMA4|LAMA5|THBS1|COL8A1|VWF|ICAM1|FERMT3|APP|ITGA2|ZYX|CDH5|EPHA2|MYH10|DPP4</td><td>1.32E-05</td><td>KW-0130</td></tr>
<tr><td>23</td><td>UniProt Keywords</td><td>Calcium</td><td>RCN1|MMP2|EHD4|PPP3R1|PXDN|THBS1|PRKCG|CAPN2|ANXA5|ITGA2|NOS3|MELK|MMP14|CALR|JAG2|CDH5|ANXA2|ANXA6|TGM2|SPTAN1|ANXA1|PRKCSH|CAMK1D</td><td>2.04E-05</td><td>KW-0106</td></tr>
<tr><td>34</td><td>UniProt Keywords</td><td>Isopeptide bond</td><td>GAPDH|ENO1|RORA|TUBB4A|APP|ANXA5|CCT2|PLK1|TUBA1A|DDB1|NQO1|CAV1|RGS12|TUBB4B|PTMA|ANXA2|PTRF|SFPQ|DLL1|FLNA|ANXA1|PSIP1|FSCN1|XRCC5|MYO1B|SUN1|RARG|TOP2B|FLNB|PBK|CFL1|VIM|NR1H4|CAMK1D</td><td>3.25E-05</td><td>KW-1017</td></tr>
<tr><td>14</td><td>UniProt Keywords</td><td>Serine/threonine-protein kinase</td><td>MAP2K5|PRKCG|AKT3|MELK|PLK1|BRSK1|PRKCH|MAP4K4|SLK|CAMK2B|CAMK2A|DYRK1A|PBK|CAMK1D</td><td>9.51E-05</td><td>KW-0723</td></tr>
<tr><td>4</td><td>UniProt Keywords</td><td>Annexin</td><td>ANXA5|ANXA2|ANXA6|ANXA1</td><td>1.30E-04</td><td>KW-0041</td></tr>
<tr><td>113</td><td>UniProt Keywords</td><td>Alternative splicing</td><td>RCN1|ARHGEF5|MAP2K5|PLOD1|ITGB4|MYH9|CYP24A1|MMP2|APLP1|GAPDH|LAMA4|RBP1|CDC25B|BCL2L2|PXDN|LAMA5|ARF3|PDGFRA|THBS1|VWF|RORA|DNAJB6|CNN2|PRKCG|AKT3|NGEF|ADD1|EXOC6B|FERMT3|TJP1|FLT1|PLOD2|APP|CSF1R|KIT|NPC1L1|CAPN2|NOS3|MELK|CCT2|TUBA1A|DDB1|BCL2L1|CDC25A|BRSK1|DAB2|TENC1|NQO1|CDC25C|STXBP6|ZYX|FLNC|JAG2|PRKCH|RARB|NR1I2|LCK|SNCA|RGS12|MAP4K4|CDH5|PTMA|ANXA2|ANXA6|FYN|FAS|ARHGEF26|PTRF|SFPQ|EPHA2|MYO1C|MYH10|TGM2|DLL1|ARHGAP30|COPA|TPM3|GSTO1|SLK|FLNA|CNN3|SDC4|SPTAN1|DNM1|TLR4|RXRB|TBC1D4|PSIP1|MYO1B|G6PD|PPP3CA|CAMK2B|BCL2|CAMK2A|DYRK1A|SEPT7|PAICS|CDC42|SUN1|PDIA6|RARG|TOP2B|WEE1|FLNB|PBK|SPTBN2|NR1H4|NUMB|APBA2|PRKCSH|ABCB1|CAMK1D|MCM3</td><td>1.50E-04</td><td>KW-0025</td></tr>
<tr><td>4</td><td>UniProt Keywords</td><td>Calcium/phospholipid-binding</td><td>ANXA5|ANXA2|ANXA6|ANXA1</td><td>1.50E-04</td><td>KW-0111</td></tr>
<tr><td>14</td><td>UniProt Keywords</td><td>Host-virus interaction</td><td>PDGFRA|ICAM1|CCR5|ITGA2|DDB1|CDC25C|ZYX|LCK|CAV1|FYN|EPHA2|PSIP1|DYRK1A|VIM</td><td>2.60E-04</td><td>KW-0945</td></tr>
<tr><td>21</td><td>UniProt Keywords</td><td>Cell projection</td><td>ARHGEF5|PDGFRA|PRKCG|NGEF|ARHGAP31|FERMT3|TJP1|APP|RGS12|ARHGEF26|EPHA2|MYO1C|MYH10|DPP4|ARHGEF15|ANXA1|FSCN1|PPP3CA|CAMK2A|SEPT7|CFL1</td><td>3.40E-04</td><td>KW-0966</td></tr>
<tr><td>5</td><td>UniProt Keywords</td><td>Nitration</td><td>CKB|TUBA1A|TUBA1C|DNM1|PPP3CA</td><td>7.50E-04</td><td>KW-0944</td></tr>
<tr><td>10</td><td>UniProt Keywords</td><td>Extracellular matrix</td><td>MMP2|LAMA4|PXDN|LAMA5|THBS1|COL8A1|VWF|CALR|ANXA2|MMRN2</td><td>0.001</td><td>KW-0272</td></tr>
<tr><td>9</td><td>UniProt Keywords</td><td>Proto-oncogene</td><td>ARHGEF5|PDGFRA|CSF1R|KIT|RARB|LCK|FYN|TPM3|BCL2</td><td>0.0017</td><td>KW-0656</td></tr>
</table>

4	UniProt Keywords	Basement membrane	LAMA4|LAMA5|COL8A1|ANXA2	0.0046	KW-0084
10	UniProt Keywords	GTP-binding	ARF3|TUBB4A|TUBA1A|TUBA1C|ARF4|TUBB4B|DNM1|SEPT7|CDC42|ARF1	0.0062	KW-0342
6	UniProt Keywords	Protein phosphatase	CDC25B|CDC25A|TENC1|CDC25C|PTPN1|PPP3CA	0.0079	KW-0904
7	UniProt Keywords	Myristate	PPP3R1|ARF3|NOS3|ARF4|LCK|FYN|ARF1	0.0084	KW-0519
4	UniProt Keywords	Myosin	MYH9|MYO1C|MYH10|MYO1B	0.0087	KW-0518
9	UniProt Keywords	Iron	PLOD1|CYP24A1|PXDN|PLOD2|APP|NOS3|CYP2R1|PIR|PPP3CA	0.0103	KW-0408
4	UniProt Keywords	S-nitrosylation	GAPDH|ANXA5|PTPN1|VIM	0.0114	KW-0702
57	UniProt Keywords	Repeat	RCN1|ITGB4|MMP2|LAMA4|GNB4|PPP3R1|PLEK|PXDN|LAMA5|PDGFRA|THBS1|COL8A1|VWF|CNN2|PRKCG|CAD|ICAM1|TJP1|FLT1|CSF1R|KIT|CAPN2|ANXA5|ITGA2|PLK1|DDB1|MMP14|CALR|ZYX|FLNC|JAG2|PRKCH|SNCA|RGS12|CDH5|ANXA2|ANXA6|FAS|PTRF|SFPQ|EPHA2|MYO1C|DLL1|COPA|FLNA|CNN3|SPTAN1|TLR4|ANXA1|TBC1D4|MYO1B|PDIA6|FLNB|SPTBN2|APBA2|PRKCSH|ABCB1	0.0133	KW-0677
2	UniProt Keywords	Congenital generalized lipodystrophy	CAV1|PTRF	0.0137	KW-1022
46	UniProt Keywords	Metal-binding	RCN1|MAP2K5|PLOD1|CYP24A1|MMP2|EHD4|APLP1|PPP3R1|ENO1|MT2A|PXDN|RORA|PRKCG|CAD|PLOD2|APP|KIT|CAPN2|ITGA2|NOS3|MMP14|BRSK1|TENC1|CALR|ZYX|RORC|PRKCH|RARB|CYP2R1|NR1I2|SNCA|CDH5|FYN|RXRG|TGM2|SPTAN1|RXRB|ANXA1|PIR|PPP3CA|CAMK2A|RARG|TOP2B|WEE1|NR1H4|PRKCSH	0.0147	KW-0479
27	UniProt Keywords	Transferase	MAP2K5|GAPDH|PDGFRA|PRKCG|AKT3|CAD|FLT1|CSF1R|KIT|MELK|CKB|PLK1|BRSK1|PRKCH|LCK|MAP4K4|FYN|EPHA2|TGM2|GSTO1|SLK|CAMK2B|CAMK2A|DYRK1A|WEE1|PBK|CAMK1D	0.0156	KW-0808
6	UniProt Keywords	Inflammatory response	CCL2|CSF1R|TLR4|ANXA1|NR1H4|CAMK1D	0.0161	KW-0395
3	UniProt Keywords	Oxidation	GAPDH|APP|PTPN1	0.017	KW-0558
12	UniProt Keywords	Oxidoreductase	PLOD1|CYP24A1|GAPDH|PXDN|PLOD2|NOS3|NQO1|CYP2R1|GSTO1|PIR|G6PD|LDHB	0.0188	KW-0560
7	UniProt Keywords	SH3 domain	ARHGEF5|NGEF|TJP1|LCK|FYN|ARHGEF26|SPTAN1	0.019	KW-0728
4	UniProt Keywords	Host cell receptor for virus entry	ICAM1|CCR5|ITGA2|EPHA2	0.019	KW-1183
41	UniProt Keywords	Cell membrane	ITGB4|EHD4|APLP1|PPP3R1|ENO1|PDGFRA|PRKCG|ADD1|TJP1|FLT1|APP|CSF1R|KIT|NPC1L1|CCR5|CAPN2|NOS3|MELK|TENC1|LCK|CAV1|CDH5|FYN|FAS|PTRF|EPHA2|MYO1C|DPP4|DLL1|TLR4|CLIC4|ANXA1|PPP3CA|LTB4R|CAMK2A|CDC42|PDIA6|CFL1|ARF1|TLN2|ABCB1	0.0197	KW-1003
5	UniProt Keywords	Endocytosis	APLP1|APP|BCL2L1|DAB2|DNM1	0.0265	KW-0254
4	UniProt Keywords	Diabetes mellitus	CCR5|CAV1|PTRF|TBC1D4	0.0268	KW-0219
**5**	**UniProt Keywords**	**Angiogenesis**	**MMP2|COL8A1|FLT1|EPHA2|MMRN2**	**0.0297**	**KW-0037**
11	UniProt Keywords	Apoptosis	APLP1|GAPDH|BCL2L2|APP|MELK|BCL2L1|DAB2|FAS|EPHA2|SLK|BCL2	0.031	KW-0053
5	UniProt Keywords	Motor protein	MYH9|MYO1C|MYH10|DNM1|MYO1B	0.031	KW-0505
9	UniProt Keywords	Synapse	PRKCG|BCL2L1|BRSK1|SNCA|RGS12|CAMK2B|CAMK2A|ARF1|TLN2	0.0383	KW-0770
3	UniProt Keywords	Sarcoplasmic reticulum	THBS1|CALR|CAMK2B	0.0385	KW-0703
4	UniProt Keywords	ER-Golgi transport	ARF3|ARF4|COPA|ARF1	0.0401	KW-0931
21	UniProt Keywords	Receptor	ITGB4|PDGFRA|RORA|ICAM1|FLT1|CSF1R|KIT|CCR5|ITGA2|RORC|RARB|NR1I2|FAS|EPHA2|RXRG|DPP4|TLR4|RXRB|LTB4R|RARG|NR1H4	0.0432	KW-0675
3	UniProt Keywords	Notch signaling pathway	APP|JAG2|DLL1	0.0454	KW-0914
2	UniProt Keywords	Heparan sulfate	SDC4|SDC1	0.0464	KW-0357

Supplemental Table 3B. Altered genes in CCM models with 2 validations enrichment data. An enrichment category was exported along with Figure 1 that detailed altered pathways involved with the identified 152 genes. Information provided in the table includes the number of enriched genes in each enrichment category, description of the category, genes specifically involved, FDR value, and the term name for each category (which includes GO terms if applicable). Among the exported pathways identified in this group, a filter search was applied to identify all enrichment data involved with angiogenesis specifically which are bolded and re-summarized in Table 1.
