# Supplementary material for: Comparative omics of CCM signaling complex (CSC)
Source: Chin Neurosurg J. 2020 Jan 15;6:4. doi: 10.1186/s41016-019-0183-6 (PMC7398211; doi:10.1186/s41016-019-0183-6)
Supplement: Supplementary file 6 — Additional file 6: Table S4. Altered genes in CSC signaling pathways assessed with various validations. An enrichment category was exported that detailed altered pathways involved with the identified genes with A) 2, B) 3 or C) 4 validations. Among the exported pathways, a filter search was applied to identify all enrichment data involved with VEGF, MAPK, Integrin, Inflammatory, Angiogenesis, Blood, cell junction organization and cell cycle signaling pathways. Information provided in the table includes the number of enriched genes in each enrichment category, description of the category, genes specifically involved, False Discovery Rate (FDR) value, and the term name for each category (which includes GO terms if applicable). Duplicated genes within each pathway sub-categories were only counted once towards each signaling pathway to construct (Fig. 3). [file 41016_2019_183_MOESM6_ESM.pdf]

Altered genes in CSC signaling pathways assessed with two validations.

Angiogenesis/ Blood pathways

# enriched genes	category	description	enriched genes	FDR value	term name
19	GO Process	angiogenesis	MYH9, MMP2,CCL2,PPP3R1,LAMA5,PDGFRA,THBS1,COL8A1,RORA,FLT1,NOS3,MMP14,CAV1,ANXA2,FN1,EPHA2,DLL1,MMRN2,CLIC4	1.18E-09	GO.0001525
5	GO Process	regulation of cell migration involved in sprouting angiogenesis	MAP2K5,THBS1,AKT3,MMRN2,ANXA1	3.20E-04	GO.0090049
6	GO Process	regulation of sprouting angiogenesis	MAP2K5,THBS1,AKT3,DLL1,MMRN2,ANXA1	3.30E-04	GO.1903670
11	GO Process	regulation of angiogenesis	MAP2K5,HSPB1,THBS1,AKT3,FLT1,NOS3,CDH5,EPHA2,DLL1,MMRN2,ANXA1	3.80E-04	GO.0045765
8	GO Process	positive regulation of angiogenesis	HSPB1,THBS1,AKT3,FLT1,NOS3,CDH5,DLL1,ANXA1	9.90E-04	GO.0045766
3	GO Process	negative regulation of cell migration involved in sprouting angiogenesis	MAP2K5,THBS1,MMRN2	0.0034	GO.0090051
7	GO Process	positive regulation of peptidase activity	APP,LCK,SNCA,CAV1,FYN,FN1,FAS	0.0063	GO.0010952
3	GO Process	positive regulation of sprouting angiogenesis	AKT3,DLL1,ANXA1	0.0226	GO.1903672
3	GO Process	sprouting angiogenesis	THBS1,DLL1,MMRN2	0.0329	GO.0002040
21	GO Process	blood vessel development	MYH9,MMP2,CCL2,PPP3R1,LAMA5,PDGFRA,THBS1,COL8A1,RORA,FLT1,NOS3,MMP14,CAV1,CDH5,ANXA2,FN1,EPHA2,MYH10,DLL1,MMRN2, CLIC4	2.88E-08	GO.0001568
8	GO Process	regulation of blood vessel endothelial cell migration	MAP2K5,HSPB1,THBS1,AKT3,NOS3,EPHA2,MMRN2,ANXA1	2.59E-05	GO.0043535
7	GO Process	regulation of blood coagulation	PLEK,PDGFRA,THBS1,NOS3,CAV1,ANXA2,TLR4	9.85E-05	GO.0030193
5	GO Process	positive regulation of blood vessel endothelial cell migration	HSPB1,THBS1,AKT3,NOS3,ANXA1	0.0015	GO.0043536
10	GO Process	blood coagulation	PDGFRA,VWF,PRKCG,ANXA5,ITGA2,PRKCH,LCK,FYN,FLNA,CDC42	0.0019	GO.0007596
3	GO Process	positive regulation of blood coagulation	PLEK,THBS1,TLR4	0.0104	GO.0030194
2	GO Process	blood vessel maturation	MMP2,CDH5	0.0156	GO.0001955
3	GO Process	negative regulation of blood coagulation	PDGFRA,NOS3,ANXA2	0.0356	GO.0030195

VEGF pathways

# enriched genes	category	description	enriched genes	FDR value	term name
7	KEGG Pathways	VEGF signaling pathway	PPP3R1,HSPB1,PRKCG,AKT3,NOS3,PPP3CA,CDC42	2.05E-05	hsa04370
8	Reactome Pathways	Signaling by VEGF	HSPB1,AKT3,FLT1,NOS3,CAV1,CDH5,FYN,CDC42	9.46E-05	HSA-194138
7	Reactome Pathways	VEGFA-VEGFR2 Pathway	HSPB1,AKT3,NOS3,CAV1,CDH5,FYN,CDC42	3.30E-04	HSA-4420097
4	Reactome Pathways	VEGFR2 mediated vascular permeability	AKT3,NOS3,CAV1,CDH5	0.0014	HSA-5218920
2	GO Function	vascular endothelial growth factor-activated receptor activity	PDGFRA,FLT1	0.0122	GO.0005021
4	GO Process	vascular endothelial growth factor signaling pathway	HSPB1,PDGFRA,FLT1,MYO1C	2.70E-04	GO.0038084
5	GO Process	cellular response to vascular endothelial growth factor stimulus	HSPB1,PDGFRA,FLT1,MYO1C,ANXA1	2.90E-04	GO.0035924
2	GO Process	positive regulation of cell migration by vascular endothelial growth factor signaling pathway	HSPB1,MYO1C	0.0131	GO.0038089
3	GO Process	regulation of vascular endothelial growth factor receptor signaling pathway	FLT1,PTPN1,MMRN2	0.0139	GO.0030947
4	GO Process	vascular endothelial growth factor receptor signaling pathway	HSPB1,FLT1,FYN,CDC42	0.0153	GO.0048010
2	GO Process	negative regulation of vascular endothelial growth factor receptor signaling pathway	PTPN1,MMRN2	0.021	GO.0030948
2	GO Process	regulation of vascular endothelial growth factor signaling pathway	MYO1C,DLL1	0.0386	GO.1900746

MAPK pathways

# enriched genes	category	description	enriched genes	FDR value	term name
6	GO Process	activation of MAPK activity	MAP2K5,THBS1,APP,KIT,PTPN1,TLR4	0.0109	GO.0000187
6	GO Process	regulation of stress-activated MAPK cascade	ARHGEF5,APP,MAP4K4,FAS,PTPN1,PBK	0.0366	GO.0032872
2	GO Process	activation of MAPKKK activity	APP,MAP4K4	0.0482	GO.0000185
18	KEGG Pathways	MAPK signaling pathway	MAP2K5,PPP3R1,CDC25B,HSPB1,PDGFRA,PRKCG,AKT3,FLT1,CSF1R,KIT,FLNC,MAP4K4,FAS,EPHA2,FLNA,PPP3CA,CDC42,FLNB	6.49E-09	hsa04010
11	Reactome Pathways	MAPK family signaling cascades	HSPB1,PDGFRA,VWF,KIT,FYN,SPTAN1,CAMK2B,CAMK2A,SEPT7,CDC42,SPTBN2	3.60E-04	HSA-5683057
8	Reactome Pathways	RAF/MAP kinase cascade	PDGFRA,VWF,KIT,FYN,SPTAN1,CAMK2B,CAMK2A,SPTBN2	0.0067	HSA-5673001

Integrin pathways

# enriched genes	category	description	enriched genes	FDR value	term name
10	GO Function	integrin binding	LAMA5,THBS1,VWF,ICAM1,FERMT3,APP,ITGA2,MMP14,CALR,FN1	2.55E-06	GO.0005178
8	GO Process	integrin-mediated signaling pathway	ITGB4,MYH9,PLEK,LAMA5,FERMT3,ITGA2,ZYX,CDC42	1.94E-05	GO.0007229
2	GO Process	regulation of cell-cell adhesion mediated by integrin	FERMT3,DPP4	0.0156	GO.0033632
2	GO Process	integrin activation	FERMT3,FN1	0.0181	GO.0033622
3	GO Process	regulation of cell adhesion mediated by integrin	FERMT3,EPHA2,DPP4	0.0262	GO.0033628
2	GO Process	cell adhesion mediated by integrin	ICAM1,ITGA2	0.0419	GO.0033627
7	Reactome Pathways	Non-integrin membrane-ECM interactions	ITGB4,LAMA4,LAMA5,THBS1,ITGA2,SDC4,SDC1	4.71E-05	HSA-3000171
5	Reactome Pathways	Integrin cell surface interactions	THBS1,COL8A1,VWF,ICAM1,ITGA2	0.0067	HSA-216083

Inflammation pathways

# enriched genes	category	description	enriched genes	FDR value	term name
17	GO Process	inflammatory response	CCL2,THBS1,ICAM1,APP,CSF1R,KIT,CCR5,SNCA,PTGES,FN1,FAS,TLR4,ANXA1,SDC1,LTB4R,NR1H4,CAMK1D	1.87E-05	GO.0006954
11	GO Process	regulation of inflammatory response	RORA,APP,ITGA2,ZYX,SNCA,CDH5,TGM2,TLR4,ANXA1,PBK,NR1H4	0.0017	GO.0050727
5	GO Process	positive regulation of inflammatory response	APP,ITGA2,SNCA,TGM2,TLR4	0.0195	GO.0050729
2	GO Process	chronic inflammatory response	THBS1,PTGES	0.0292	GO.0002544
4	KEGG Pathways	Inflammatory mediator regulation of TRP channels	PRKCG,PRKCH,CAMK2B,CAMK2A	0.0232	hsa04750
3	KEGG Pathways	Inflammatory bowel disease (IBD)	RORA,RORC,TLR4	0.0393	hsa05321
2	Reactome Pathways	The NLRP1 inflammasome	BCL2L1,BCL2	0.0055	HSA-844455
3	Reactome Pathways	Inflammasomes	APP,BCL2L1,BCL2	0.0068	HSA-622312

Cell Cycle pathways

# enriched genes	category	description	enriched genes	FDR value	term name
10	GO Process	G2/M transition of mitotic cell cycle	CDC25B,TUBB4A,MELK,PLK1,TUBA1A,CDC25A,CDC25C,TUBB4B,WEE1,ABCB1	4.04E-06	GO.0000086
13	GO Process	mitotic cell cycle phase transition	CDC25B,TUBB4A,MELK,PLK1,TUBA1A,CDC25A,CDC25C,TUBB4B,PPP3CA,CAMK2A,WEE1,ABCB1,MCM3	8.40E-06	GO.0044772
19	GO Process	mitotic cell cycle process	CDC25B,TUBB4A,MELK,PLK1,TUBA1A,BCL2L1,CDC25A,BRSK1,CDC25C,TUBB4B,MYH10,FLNA,PPP3CA,CAMK2A,TOP2B,WEE1,CFL1,ABCB1,MCM3	8.83E-06	GO.1903047
20	GO Process	mitotic cell cycle	CDC25B,TUBB4A,MELK,PLK1,TUBA1A,BCL2L1,CDC25A,BRSK1,CDC25C,TUBB4B,MYH10,FLNA,PPP3CA,CAMK2A,TOP2B,WEE1,PBK,CFL1,ABCB1,MCM3	1.00E-05	GO.0000278
22	GO Process	cell cycle process	MYH9,CDC25B,THBS1,TUBB4A,MELK,PLK1,TUBA1A,BCL2L1,CDC25A,BRSK1,CDC25C,TUBB4B,MYH10,FLNA,PPP3CA,CAMK2A,SUN1,TOP2B,WEE1,CFL1,ABCB1,MCM3	1.10E-04	GO.0022402
9	GO Process	regulation of cell cycle G2/M phase transition	CDC25B,TUBB4A,APP,PLK1,TUBA1A,CDC25A,BRSK1,CDC25C,TUBB4B	2.30E-04	GO.1902749
17	GO Process	regulation of mitotic cell cycle	MAP2K5,CCL2,CDC25B,TUBB4A,APP,PLK1,TUBA1A,DDB1,BCL2L1,BRSK1,CDC25C,TUBB4B,MAP4K4,SLK,ANXA1,BCL2,TOP2B	2.50E-04	GO.0007346
13	GO Process	regulation of cell cycle phase transition	CCL2,CDC25B,TUBB4A,APP,PLK1,TUBA1A,DDB1,CDC25A,BRSK1,CDC25C,TUBB4B,ANXA1,BCL2	3.70E-04	GO.1901987
17	GO Process	regulation of cell cycle process	CCL2,CDC25B,TUBB4A,APP,PLK1,TUBA1A,DDB1,BCL2L1,CDC25A,BRSK1,CALR,CDC25C,TUBB4B,SFPQ,ANXA1,BCL2,CDC42	8.80E-04	GO.0010564
4	GO Process	positive regulation of cell cycle G2/M phase transition	CDC25B,APP,CDC25A,CDC25C	0.0013	GO.1902751
24	GO Process	cell cycle	MYH9,CDC25B,THBS1,TUBB4A,MELK,PLK1,TUBA1A,BCL2L1,CDC25A,BRSK1,CDC25C,TUBB4B,MYH10,FLNA,PPP3CA,CAMK2A,SEPT7,SUN1,TOP2B,WEE1,PBK,CFL1,ABCB1,MCM3	0.0016	GO.0007049
22	GO Process	regulation of cell cycle	MAP2K5,CCL2,CDC25B,THBS1,TUBB4A,APP,PLK1,TUBA1A,DDB1,BCL2L1,CDC25A,BRSK1,CALR,CDC25C,TUBB4B,MAP4K4,SFPQ,SLK,ANXA1,BCL2,CDC42,TOP2B	0.0021	GO.0051726
11	GO Process	regulation of mitotic cell cycle phase transition	CCL2,TUBB4A,APP,PLK1,TUBA1A,DDB1,BRSK1,CDC25C,TUBB4B,ANXA1,BCL2	0.0021	GO.1901990
5	GO Process	positive regulation of cell cycle phase transition	CDC25B,APP,CDC25A,CDC25C,ANXA1	0.0054	GO.1901989
6	GO Process	regulation of G2/M transition of mitotic cell cycle	TUBB4A,APP,PLK1,TUBA1A,BRSK1,TUBB4B	0.0109	GO.0010389
5	GO Process	G1/S transition of mitotic cell cycle	CDC25A,PPP3CA,CAMK2A,WEE1,MCM3	0.021	GO.0000082
7	GO Process	negative regulation of mitotic cell cycle	CCL2,PLK1,BCL2L1,BRSK1,CDC25C,BCL2,TOP2B	0.0232	GO.0045930
7	GO Process	positive regulation of cell cycle process	CDC25B,APP,CDC25A,CDC25C,SFPQ,ANXA1,CDC42	0.0366	GO.0090068
5	GO Process	mitotic cell cycle checkpoint	PLK1,BCL2L1,BRSK1,CDC25C,TOP2B	0.0409	GO.0007093
5	GO Process	negative regulation of mitotic cell cycle phase transition	CCL2,PLK1,BRSK1,CDC25C,BCL2	0.0463	GO.1901991
5	GO Process	meiotic cell cycle process	MYH9,CDC25B,PLK1,SUN1,TOP2B	0.0471	GO.1903046
6	KEGG Pathways	Cell cycle	CDC25B,PLK1,CDC25A,CDC25C,WEE1,MCM3	0.0038	hsa04110
12	Reactome Pathways	Cell Cycle, Mitotic	CDC25B,AKT3,TUBB4A,PLK1,TUBA1A,TUBA1C,CDC25A,CDC25C,TUBB4B,DYRK1A,WEE1,MCM3	0.0068	HSA-69278
13	Reactome Pathways	Cell Cycle	CDC25B,AKT3,TUBB4A,PLK1,TUBA1A,TUBA1C,CDC25A,CDC25C,TUBB4B,DYRK1A,SUN1,WEE1,MCM3	0.0096	HSA-1640170

Cell Junction organization pathways

# enriched genes	category	description	enriched genes	FDR value	term name
7	GO Component	focal adhesion	MYH9,ARHGAP31,ITGA2,TENC1,ZYX,EPHA2,SDC4	0.001	GO.0005925
3	GO Process	focal adhesion assembly	LAMA5,ITGA2,BCL2	0.0087	GO.0048041
4	GO Process	regulation of focal adhesion assembly	THBS1,MMP14,SLK,SDC4	0.0094	GO.0051893
2	GO Process	negative regulation of focal adhesion assembly	THBS1,MMP14	0.0353	GO.0051895
20	KEGG Pathways	Focal adhesion	ITGB4,LAMA4,LAMA5,PDGFRA,THBS1,VWF,PRKCG,AKT3,FLT1,CAPN2,ITGA2,ZYX,FLNC,CAV1,FYN,FLNA,BCL2,CDC42,FLNB,TLN2	3.60E-13	hsa04510
33	GO Component	cell junction	ARHGEF5,ITGB4,MYH9,PDGFRA,CNN2,PRKCG,ARHGAP31,ADD1,FERMT3,TJP1,APP,KIT,ITGA2,BCL2L1,BRSK1,TENC1,ZYX,PRKCH,SNCA,RGS12,CDH5,EPHA2,DPP4,DLL1,FLNA,SDC4,CLIC4,FSCN1,PPP3CA,CAMK2B,CAMK2A,SPTBN2,CFL1	1.26E-09	GO.0030054
16	GO Component	cell-cell junction	MYH9,CNN2,PRKCG,ADD1,TJP1,APP,KIT,ZYX,PRKCH,CDH5,DPP4,FLNA,CLIC4,FSCN1,PPP3CA,CFL1	4.54E-06	GO.0005911
11	GO Component	adherens junction	MYH9,ARHGAP31,ADD1,TJP1,ITGA2,TENC1,ZYX,CDH5,EPHA2,DLL1,SDC4	9.90E-05	GO.0005912
8	GO Component	cell-substrate junction	ITGB4,MYH9,ARHGAP31,ITGA2,TENC1,ZYX,EPHA2,SDC4	2.80E-04	GO.0030055
5	GO Component	cell-cell adherens junction	MYH9,ADD1,TJP1,ZYX,CDH5	0.0024	GO.0005913
3	GO Component	neuromuscular junction	MYH9,APP,MYH10	0.038	GO.0031594
15	GO Process	cell junction organization	ITGB4,LAMA5,TJP1,CSF1R,ITGA2,FLNC,CDH5,FN1,FLNA,FSCN1,BCL2,CDC42,NR1H4,NUMB,TLN2	1.40E-08	GO.0034330
13	GO Process	cell junction assembly	ITGB4,LAMA5,TJP1,ITGA2,FLNC,CDH5,FN1,FLNA,FSCN1,BCL2,CDC42,NR1H4,TLN2	1.53E-08	GO.0034329
8	GO Process	regulation of cell junction assembly	THBS1,TJP1,MMP14,PRKCH,CAV1,MYO1C,SLK,SDC4	1.82E-05	GO.1901888
10	GO Process	cell-cell junction organization	LAMA5,TJP1,CSF1R,ITGA2,CDH5,FSCN1,BCL2,NR1H4,NUMB,TLN2	4.36E-05	GO.0045216
5	GO Process	cell-substrate junction assembly	ITGB4,LAMA5,ITGA2,FN1,BCL2	4.30E-04	GO.0007044
5	GO Process	regulation of adherens junction organization	THBS1,ADD1,MMP14,SLK,SDC4	0.0019	GO.1903391
5	GO Process	adherens junction organization	LAMA5,ITGA2,CDH5,BCL2,NUMB	0.0041	GO.0034332
5	GO Process	cell-cell junction assembly	TJP1,CDH5,FSCN1,NR1H4,TLN2	0.0044	GO.0007043
3	GO Process	regulation of bicellular tight junction assembly	TJP1,PRKCH,MYO1C	0.0061	GO.2000810
8	KEGG Pathways	Gap junction	MAP2K5,PDGFRA,PRKCG,TUBB4A,TJP1,TUBA1A,TUBA1C,TUBB4B	2.05E-05	hsa04540
6	KEGG Pathways	Tight junction	MYH9,TJP1,TUBA1A,TUBA1C,MYH10,CDC42	0.0108	hsa04530
4	KEGG Pathways	Adherens junction	TJP1,FYN,PTPN1,CDC42	0.0111	hsa04520
7	Reactome Pathways	Gap junction trafficking and regulation	TUBB4A,TJP1,TUBA1A,TUBA1C,DAB2,TUBB4B,DNM1	1.58E-05	HSA-157858
6	Reactome Pathways	Gap junction trafficking	TUBB4A,TUBA1A,TUBA1C,DAB2,TUBB4B,DNM1	9.46E-05	HSA-190828
2	Reactome Pathways	Formation of annular gap junctions	DAB2,DNM1	0.0229	HSA-196025

4	Reactome Pathways	Cell junction organization	ITGB4,FLNC,CDH5,FLNA	0.0306	HSA-446728

Altered genes in CSC signaling pathways assessed with Three validations.

Integrin pathways

# enriched genes	category	description	enriched genes	FDR value	term name
4	InterPro Domains	Integrin domain superfamily	ITGA1,ITGB5,ITGA10,ITGA6	2.08E-07	IPR032695
3	InterPro Domains	Integrin alpha chain	ITGA1,ITGA10,ITGA6	1.04E-05	IPR000413
3	InterPro Domains	Integrin alpha beta-propellor	ITGA1,ITGA10,ITGA6	1.04E-05	IPR013519
3	InterPro Domains	Integrin alpha-2	ITGA1,ITGA10,ITGA6	1.04E-05	IPR013649
3	InterPro Domains	Integrin alpha, N-terminal	ITGA1,ITGA10,ITGA6	1.04E-05	IPR028994
2	InterPro Domains	Integrin beta subunit, VWA domain	ITGB4,ITGB5	1.20E-04	IPR002369
2	InterPro Domains	Integrin beta subunit, tail	ITGB4,ITGB5	1.20E-04	IPR012896
2	InterPro Domains	Integrin beta subunit	ITGB4,ITGB5	1.20E-04	IPR015812
2	InterPro Domains	Integrin beta N-terminal	ITGB4,ITGB5	1.20E-04	IPR033760
2	InterPro Domains	Integrin beta tail domain superfamily	ITGB4,ITGB5	1.20E-04	IPR036349
2	InterPro Domains	Integrin alpha chain, C-terminal cytoplasmic region, conserved site	ITGA1,ITGA6	2.80E-04	IPR018184
3	Pfam	Integrin alpha	ITGA1,ITGA10,ITGA6	1.11E-05	PF08441
2	Pfam	Integrin beta tail domain	ITGB4,ITGB5	1.30E-04	PF07965
2	Pfam	Integrin beta chain VWA domain	ITGB4,ITGB5	1.50E-04	PF00362
2	Pfam	Integrin plexin domain	ITGB4,ITGB5	1.50E-04	PF17205
5	Reactome Pathways	Non-integrin membrane-ECM interactions	ITGB4,LAMC2,ITGB5,LAMB3,ITGA6	7.25E-09	HSA-3000171
4	Reactome Pathways	Integrin cell surface interactions	ITGA1,ITGB5,ITGA10,ITGA6	3.95E-06	HSA-216083

Cell Junction organization pathways

# enriched genes	category	description	enriched genes	FDR value	term name
4	GO Component	cell-substrate junction	ITGB4 ,ITGA1 ,PLEC ,ITGA6	6.42E-05	GO.0030055
3	GO Component	adherens junction	ITGA1 ,PLEC ,ITGA6	0.006	GO.0005912
2	GO Component	focal adhesion	ITGA1 ,PLEC	0.0285	GO.0005925
2	GO Function	collagen binding involved in cell-matrix adhesion	ITGA1 ,ITGA10	3.40E-04	GO.0098639
7	GO Process	cell junction assembly	ITGB4 ,LAMC2 ,PLEC ,ACTB ,LAMB3 ,CD151 ,ITGA6	4.44E-10	GO.0034329
5	GO Process	cell-matrix adhesion	ITGB4 ,ITGA1 ,ITGB5 ,ITGA10 ,ITGA6	9.99E-07	GO.0007160
8	GO Process	cell adhesion	ITGB4 ,LAMC2 ,ITGA1 ,ITGB5 ,ITGA10 ,LAMB3 ,CD151 ,ITGA6	1.45E-06	GO.0007155
8	KEGG Pathways	Focal adhesion	ITGB4 ,LAMC2 ,ITGA1 ,ITGB5 ,ACTB ,ITGA10 ,LAMB3 ,ITGA6	6.45E-12	hsa04510
7	Reactome Pathways	Cell junction organization	ITGB4 ,LAMC2 ,PLEC ,ACTB ,LAMB3 ,CD151 ,ITGA6	7.10E-12	HSA-446728
2	Reactome Pathways	Platelet Adhesion to exposed collagen	ITGA1 ,ITGA10	5.40E-04	HSA-75892

Altered genes in CSC signaling pathways assessed with four validations.

Cell Cycle pathways

# enriched genes	category	description	enriched genes	FDR value	term name
5	GO Process	G2/M transition of mitotic cell cycle	TUBA4A,TUBA1A,TUBB,TUBB4B,CKAP5	4.58E-07	GO.0000086
7	GO Process	mitotic cell cycle	TUBA4A,TUBA1A,TUBB,TUBB4B,TBCD,CDC5L,CKAP5	5.21E-07	GO.0000278
5	GO Process	regulation of G2/M transition of mitotic cell cycle	TUBA4A,TUBA1A,TUBB,TUBB4B,CKAP5	5.21E-07	GO.0010389
6	GO Process	regulation of cell cycle	TUBA4A,TUBA1A,TUBB,TUBB4B,CDC5L,CKAP5	1.40E-04	GO.0051726

Cell Junction organization pathways

# enriched genes	category	description	enriched genes	FDR value	term name
7	KEGG Pathways	Gap junction	TUBA4A|TUBA1A|TUBA1C|TUBA1B|TUBB|TUBB4B|TUBB2A	4.57E-14	hsa04540
4	KEGG Pathways	Tight junction	TUBA4A|TUBA1A|TUBA1C|TUBA1B	2.12E-06	hsa04530

Supplemental Table 4. Altered genes in CSC signaling pathways assessed with various validations. An enrichment category was exported that detailed altered pathways involved with the identified genes with **A)** 2, **B)** 3 or **C)** 4 validations. Among the exported pathways, a filter search was applied to identify all enrichment data involved with VEGF, MAPK, Integrin, Inflammatory, Angiogenesis, Blood, cell junction organization and cell cycle signaling pathways. Information provided in the table includes the number of enriched genes in each enrichment category, description of the category, genes specifically involved, False Discovery Rate (FDR) value, and the term name for each category (which includes GO terms if applicable). Duplicated genes within each pathway sub-categories were only counted once towards each signaling pathway to construct (Figure 3).
