# Supplementary material for: Comparative omics of CCM signaling complex (CSC)
Source: Chin Neurosurg J. 2020 Jan 15;6:4. doi: 10.1186/s41016-019-0183-6 (PMC7398211; doi:10.1186/s41016-019-0183-6)
Supplement: Supplementary file 7 — Additional file 7: Table S5A. Detailed description of altered genes in CCM models with 3 validations. A total of 4 genes were analyzed that overlapped in three different CCM studies. Details provided for each protein include mechanisms associated with each, functions, binding partners, motifs and domains. Protein details were extracted from STRING enrichment data after construction of Fig. 4 Interactome. Proteins in bold are the 4 validated proteins, while other proteins are the 10 interactors added to interactome. [file 41016_2019_183_MOESM7_ESM.pdf]

Detailed description of altered genes in CCM models with 3 validations.

display name	description
**TPM4**	Tropomyosin alpha-4 chain; Tropomyosin 4; Tropomyosins
**ITGB4**	Integrin, beta 4; Integrin alpha-6/beta-4 is a receptor for laminin. Plays a critical structural role in the hemidesmosome of epithelial cells. Is required for the regulation of keratinocyte polarity and motility. ITGA6:ITGB4 binds to NRG1 (via EGF domain) and this binding is essential for NRG1-ERBB signaling. ITGA6:ITGB4 binds to IGF1 and this binding is essential for IGF1 signaling.
**ACTB**	Actin, cytoplasmic 1; Actins are highly conserved proteins that are involved in various types of cell motility and are ubiquitously expressed in all eukaryotic cells.
**PLEC**	Hemidesmosomal protein 1; Interlinks intermediate filaments with microtubules and microfilaments and anchors intermediate filaments to desmosomes or hemidesmosomes. Could also bind muscle proteins such as actin to membrane complexes in muscle. May be involved not only in the filaments network, but also in the regulation of their dynamics. Structural component of muscle. Isoform 9 plays a major role in the maintenance of myofiber integrity; Plakins
SYNE3	Spectrin repeat containing, nuclear envelope family member 3; As a component of the LINC (LInker of Nucleoskeleton and Cytoskeleton) complex involved in the connection between the nuclear lamina and the cytoskeleton. The nucleocytoplasmic interactions established by the LINC complex play an important role in the transmission of mechanical forces across the nuclear envelope and in nuclear movement and positioning. Probable anchoring protein which tethers the nucleus to the cytoskeleton by binding PLEC which can associate with the intermediate filament system. Plays a role in the regulation of aortic epithelial cell morphology, and is required for flow-induced centrosome polarization and directional migration in aortic endothelial cells; Belongs to the nesprin family.
ITGA1	CD49 antigen-like family member A; Integrin alpha-1/beta-1 is a receptor for laminin and collagen. It recognizes the proline-hydroxylated sequence G-F-P-G- E-R in collagen. Involved in anchorage-dependent, negative regulation of EGF-stimulated cell growth; CD molecules
CD151	Platelet-endothelial tetraspan antigen 3; Essential for the proper assembly of the glomerular and tubular basement membranes in kidney; Blood group antigens
ITGA6	CD49 antigen-like family member F; Integrin alpha-6/beta-1 is a receptor for laminin on platelets. Integrin alpha-6/beta-4 is a receptor for laminin in epithelial cells and it plays a critical structural role in the hemidesmosome (By similarity). ITGA6:ITGB4 binds to NRG1 (via EGF domain) and this binding is essential for NRG1-ERBB signaling. ITGA6:ITGB4 binds to IGF1 and this binding is essential for IGF1 signaling.

NAT9	N-acetyltransferase 9 (GCN5-related, putative); GCN5 related N-acetyltransferases; Belongs to the acetyltransferase family. GNAT subfamily.
ITGB5	Integrin, beta 5; Integrin alpha-V/beta-5 (ITGAV:ITGB5) is a receptor for fibronectin. It recognizes the sequence R-G-D in its ligand.
PFN3	Profilin III; Binds to actin and affects the structure of the cytoskeleton. Slightly reduces actin polymerization. Binds to poly-L-proline, phosphatidylinositol 3-phosphate (PtdIns(3)P), phosphatidylinositol 4,5-bisphosphate (PtdIns(4,5)P2) and phosphatidylinositol 4-phosphate (PtdIns(4)P). May be involved in spermatogenesis; Belongs to the profilin family.
ITGA10	Integrin, alpha 10; Integrin alpha-10/beta-1 is a receptor for collagen; Belongs to the integrin alpha chain family.
LAMC2	Large adhesive scatter factor 140 kDa subunit; Binding to cells via a high affinity receptor, laminin is thought to mediate the attachment, migration and organization of cells into tissues during embryonic development by interacting with other extracellular matrix components. Ladsin exerts cell-scattering activity toward a wide variety of cells, including epithelial, endothelial, and fibroblastic cells.
LAMB3	Epiligrin subunit bata; Binding to cells via a high affinity receptor, laminin is thought to mediate the attachment, migration and organization of cells into tissues during embryonic development by interacting with other extracellular matrix components.

Supplemental Table 5A. Detailed description of altered genes in CCM models with 3 validations. A total of 4 genes were analyzed that overlapped in three different CCM studies. Details provided for each protein include mechanisms associated with each, functions, binding partners, motifs and domains. Protein details were extracted from STRING enrichment data after construction of Figure 4 Interactome. Proteins in bold are the 4 validated proteins, while other proteins are the 10 interactors added to interactome.
