# Supplementary material for: Comparative omics of CCM signaling complex (CSC)
Source: Chin Neurosurg J. 2020 Jan 15;6:4. doi: 10.1186/s41016-019-0183-6 (PMC7398211; doi:10.1186/s41016-019-0183-6)
Supplement: Supplementary file 8 — Additional file 8: Table S5B. Altered genes in CCM models with 3 validations enrichment data. An enrichment category was exported along with Figure 4 that detailed altered pathways involved with the identified 4 genes and 10 interactors. Information provided in the table includes the number of enriched genes in each enrichment category, description of the category, genes specifically involved, FDR value, and the term name for each category (which includes GO terms if applicable). [file 41016_2019_183_MOESM8_ESM.pdf]

Altered genes in CCM models with 3 validations enrichment data

# enriched genes	category	description	enriched genes	FDR value	term name
5	GO Component	integrin complex	ITGB4|ITGA1|ITGB5|ITGA10|ITGA6	4.07E-09	GO.0008305
3	GO Component	hemidesmosome	ITGB4|PLEC|ITGA6	1.76E-06	GO.0030056
4	GO Component	basement membrane	LAMC2|LAMB3|CD151|ITGA6	1.52E-05	GO.0005604
4	GO Component	cell-substrate junction	ITGB4|ITGA1|PLEC|ITGA6	6.42E-05	GO.0030055
2	GO Component	laminin complex	LAMC2|LAMB3	3.20E-04	GO.0043256
6	GO Component	membrane protein complex	ITGB4|ITGA1|ITGB5|SYNE3|ITGA10|ITGA6	6.20E-04	GO.0098796
5	GO Component	cell surface	ITGB4|ITGA1|ITGB5|CD151|ITGA6	0.001	GO.0009986
10	GO Component	protein-containing complex	ITGB4|LAMC2|ITGA1|ITGB5|SYNE3|ACTB|NAT9 |ITGA10|LAMB3|ITGA6	0.0031	GO.0032991
6	GO Component	integral component of plasma membrane	ITGB4|ITGA1|ITGB5|ITGA10|CD151|ITGA6	0.0046	GO.0005887
2	GO Component	basal part of cell	ITGA1|ITGA6	0.0046	GO.0045178
3	GO Component	adherens junction	ITGA1|PLEC|ITGA6	0.006	GO.0005912
9	GO Component	cell periphery	ITGB4|LAMC2|ITGA1|ITGB5|PLEC|ACTB|ITGA10 |CD151|ITGA6	0.0268	GO.0071944
2	GO Component	focal adhesion	ITGA1|PLEC	0.0285	GO.0005925
7	GO Function	protein-containing complex binding	ITGA1|SYNE3|TPM4|ITGA10|LAMB3|CD151|ITG A6	1.30E-04	GO.0044877
2	GO Function	collagen binding involved in cell-matrix adhesion	ITGA1|ITGA10	3.40E-04	GO.0098639
4	GO Function	actin binding	PLEC|SYNE3|TPM4|PFN3	0.0028	GO.0003779
5	GO Function	cytoskeletal protein binding	PLEC|SYNE3|TPM4|ACTB|PFN3	0.0031	GO.0008092
2	GO Function	structural constituent of muscle	PLEC|TPM4	0.005	GO.0008307
4	GO Function	structural molecule activity	PLEC|TPM4|ACTB|LAMB3	0.0085	GO.0005198
2	GO Function	integrin binding	CD151|ITGA6	0.0238	GO.0005178
2	GO Function	actin filament binding	SYNE3|TPM4	0.0355	GO.0051015
6	GO Process	hemidesmosome assembly	ITGB4|LAMC2|PLEC|LAMB3|CD151|ITGA6	3.16E-13	GO.0031581
7	GO Process	cell junction assembly	ITGB4|LAMC2|PLEC|ACTB|LAMB3|CD151|ITGA 6	4.44E-10	GO.0034329
7	GO Process	extracellular matrix organization	ITGB4|LAMC2|ITGA1|ITGB5|ITGA10|LAMB3|IT GA6	5.52E-08	GO.0030198
5	GO Process	integrin-mediated signaling pathway	ITGB4|ITGA1|ITGB5|ITGA10|ITGA6	2.13E-07	GO.0007229
5	GO Process	cell-matrix adhesion	ITGB4|ITGA1|ITGB5|ITGA10|ITGA6	9.99E-07	GO.0007160
8	GO Process	cell adhesion	ITGB4|LAMC2|ITGA1|ITGB5|ITGA10|LAMB3|CD 151|ITGA6	1.45E-06	GO.0007155
13	GO Process	cellular component organization	ITGB4|LAMC2|ITGA1|ITGB5|PLEC|SYNE3|TPM4 |ACTB|PFN3|ITGA10|LAMB3|CD151|ITGA6	1.19E-05	GO.0016043
2	GO Process	nail development	ITGB4|ITGA6	9.90E-04	GO.0035878
8	GO Process	cellular component assembly	ITGB4|LAMC2|ITGB5|PLEC|ACTB|LAMB3|CD15 1|ITGA6	0.0019	GO.0022607
3	GO Process	formation of primary germ layer	ITGB4|ITGB5|LAMB3	0.0021	GO.0001704
2	GO Process	amelogenesis	ITGB4|ITGA6	0.0028	GO.0097186
4	GO Process	actin cytoskeleton organization	ITGB5|TPM4|ACTB|PFN3	0.0042	GO.0030036
6	GO Process	movement of cell or subcellular component	ITGB4|ITGA1|TPM4|ACTB|CD151|ITGA6	0.0043	GO.0006928
2	GO Process	brown fat cell differentiation	LAMB3|ITGA6	0.0043	GO.0050873
5	GO Process	cell motility	ITGB4|ITGA1|ACTB|CD151|ITGA6	0.0061	GO.0048870
5	GO Process	cytoskeleton organization	ITGB5|SYNE3|TPM4|ACTB|PFN3	0.0065	GO.0007010
7	GO Process	cell surface receptor signaling pathway	ITGB4|ITGA1|ITGB5|ACTB|ITGA10|CD151|ITGA 6	0.0065	GO.0007166
2	GO Process	endodermal cell differentiation	ITGB5|LAMB3	0.0067	GO.0035987
6	GO Process	tissue development	ITGB4|LAMC2|ITGB5|LAMB3|CD151|ITGA6	0.0085	GO.0009888
3	GO Process	muscle contraction	ITGA1|ITGB5|TPM4	0.0097	GO.0006936
6	GO Process	anatomical structure morphogenesis	ITGB4|ITGA1|ITGB5|LAMB3|CD151|ITGA6	0.0202	GO.0009653
4	GO Process	anatomical structure formation involved in morphogenesis	ITGB4|ITGB5|LAMB3|ITGA6	0.0281	GO.0048646
3	GO Process	positive regulation of cell migration	LAMC2|CD151|ITGA6	0.0408	GO.0030335
2	GO Process	digestive tract development	ITGB4|ITGA6	0.0408	GO.0048565
4	InterPro Domains	Integrin domain superfamily	ITGA1|ITGB5|ITGA10|ITGA6	2.08E-07	IPR032695
3	InterPro Domains	Integrin alpha chain	ITGA1|ITGA10|ITGA6	1.04E-05	IPR000413
3	InterPro Domains	FG-GAP repeat	ITGA1|ITGA10|ITGA6	1.04E-05	IPR013517
3	InterPro Domains	Integrin alpha beta-propellor	ITGA1|ITGA10|ITGA6	1.04E-05	IPR013519
3	InterPro Domains	Integrin alpha-2	ITGA1|ITGA10|ITGA6	1.04E-05	IPR013649
3	InterPro Domains	Integrin alpha, N-terminal	ITGA1|ITGA10|ITGA6	1.04E-05	IPR028994
4	InterPro Domains	von Willebrand factor A-like domain superfamily	ITGB4|ITGA1|ITGB5|ITGA10	1.04E-05	IPR036465
3	InterPro Domains	von Willebrand factor, type A	ITGA1|ITGB5|ITGA10	1.20E-04	IPR002035
2	InterPro Domains	Integrin beta subunit, VWA domain	ITGB4|ITGB5	1.20E-04	IPR002369
2	InterPro Domains	Integrin beta subunit, tail	ITGB4|ITGB5	1.20E-04	IPR012896
2	InterPro Domains	Integrin beta subunit	ITGB4|ITGB5	1.20E-04	IPR015812
2	InterPro Domains	Integrin beta N-terminal	ITGB4|ITGB5	1.20E-04	IPR033760
2	InterPro Domains	Integrin beta tail domain superfamily	ITGB4|ITGB5	1.20E-04	IPR036349
2	InterPro Domains	Integrin alpha chain, C-terminal cytoplasmic region, conserved site	ITGA1|ITGA6	2.80E-04	IPR018184
2	InterPro Domains	Spectrin/alpha-actinin	PLEC|SYNE3	7.90E-04	IPR018159
2	InterPro Domains	Laminin EGF domain	LAMC2|LAMB3	0.0011	IPR002049
2	InterPro Domains	PSI domain	ITGB4|ITGB5	0.0014	IPR016201
2	InterPro Domains	EGF-like, conserved site	ITGB4|LAMB3	0.0249	IPR013032
7	KEGG Pathways	ECM-receptor interaction	ITGB4|LAMC2|ITGA1|ITGB5|ITGA10|LAMB3|IT GA6	3.50E-12	hsa04512

8	KEGG Pathways	Focal adhesion	ITGB4|LAMC2|ITGA1|ITGB5|ACTB|ITGA10|LAMB3|ITGA6	6.45E-12	hsa04510
6	KEGG Pathways	Arrhythmogenic right ventricular cardiomyopathy (ARVC)	ITGB4|ITGA1|ITGB5|ACTB|ITGA10|ITGA6	1.15E-10	hsa05412
6	KEGG Pathways	Hypertrophic cardiomyopathy (HCM)	ITGB4|ITGA1|ITGB5|ACTB|ITGA10|ITGA6	1.70E-10	hsa05410
6	KEGG Pathways	Dilated cardiomyopathy (DCM)	ITGB4|ITGA1|ITGB5|ACTB|ITGA10|ITGA6	2.18E-10	hsa05414
7	KEGG Pathways	Regulation of actin cytoskeleton	ITGB4|ITGA1|ITGB5|ACTB|PFN3|ITGA10|ITGA6	3.05E-10	hsa04810
7	KEGG Pathways	Human papillomavirus infection	ITGB4|LAMC2|ITGA1|ITGB5|ITGA10|LAMB3|ITGA6	5.09E-09	hsa05165
7	KEGG Pathways	PI3K-Akt signaling pathway	ITGB4|LAMC2|ITGA1|ITGB5|ITGA10|LAMB3|ITGA6	8.41E-09	hsa04151
3	KEGG Pathways	Small cell lung cancer	LAMC2|LAMB3|ITGA6	1.50E-04	hsa05222
3	KEGG Pathways	Toxoplasmosis	LAMC2|LAMB3|ITGA6	2.30E-04	hsa05145
2	KEGG Pathways	Shigellosis	ACTB|PFN3	0.0031	hsa05131
2	KEGG Pathways	Salmonella infection	ACTB|PFN3	0.005	hsa05132
2	KEGG Pathways	Hematopoietic cell lineage	ITGA1|ITGA6	0.0058	hsa04640
2	KEGG Pathways	Amoebiasis	LAMC2|LAMB3	0.0058	hsa05146
2	KEGG Pathways	Phagosome	ITGB5|ACTB	0.0115	hsa04145
3	KEGG Pathways	Pathways in cancer	LAMC2|LAMB3|ITGA6	0.0121	hsa05200
2	KEGG Pathways	Proteoglycans in cancer	ITGB5|ACTB	0.0179	hsa05205
2	KEGG Pathways	Rap1 signaling pathway	ACTB|PFN3	0.0183	hsa04015
3	Pfam	FG-GAP repeat	ITGA1|ITGA10|ITGA6	1.11E-05	PF01839
3	Pfam	Integrin alpha	ITGA1|ITGA10|ITGA6	1.11E-05	PF08441
2	Pfam	Integrin beta tail domain	ITGB4|ITGB5	1.30E-04	PF07965
2	Pfam	Integrin beta chain VWA domain	ITGB4|ITGB5	1.50E-04	PF00362
2	Pfam	Integrin plexin domain	ITGB4|ITGB5	1.50E-04	PF17205
2	Pfam	Laminin EGF domain	LAMC2|LAMB3	0.0012	PF00053
2	Pfam	von Willebrand factor type A domain	ITGA1|ITGA10	0.0034	PF00092
2	Pfam	von Willebrand factor type A domain	ITGA1|ITGA10	0.0034	PF13519
6	Reactome Pathways	Type I hemidesmosome assembly	ITGB4|LAMC2|PLEC|LAMB3|CD151|ITGA6	4.78E-14	HSA-446107
9	Reactome Pathways	Extracellular matrix organization	ITGB4|LAMC2|ITGA1|ITGB5|PLEC|ITGA10|LAMB3|CD151|ITGA6	4.81E-12	HSA-1474244
7	Reactome Pathways	Cell junction organization	ITGB4|LAMC2|PLEC|ACTB|LAMB3|CD151|ITGA6	7.10E-12	HSA-446728
6	Reactome Pathways	Assembly of collagen fibrils and other multimeric structures	ITGB4|LAMC2|PLEC|LAMB3|CD151|ITGA6	6.90E-11	HSA-2022090
5	Reactome Pathways	Laminin interactions	ITGB4|LAMC2|ITGA1|LAMB3|ITGA6	4.51E-10	HSA-3000157
5	Reactome Pathways	Non-integrin membrane-ECM interactions	ITGB4|LAMC2|ITGB5|LAMB3|ITGA6	7.25E-09	HSA-3000171
4	Reactome Pathways	Integrin cell surface interactions	ITGA1|ITGB5|ITGA10|ITGA6	3.95E-06	HSA-216083
3	Reactome Pathways	Syndecan interactions	ITGB4|ITGB5|ITGA6	1.06E-05	HSA-3000170
2	Reactome Pathways	CHL1 interactions	ITGA1|ITGA10	2.40E-04	HSA-447041
2	Reactome Pathways	Anchoring fibril formation	LAMC2|LAMB3	5.40E-04	HSA-2214320
2	Reactome Pathways	Platelet Adhesion to exposed collagen	ITGA1|ITGA10	5.40E-04	HSA-75892
3	Reactome Pathways	L1CAM interactions	ITGA1|ACTB|ITGA10	5.50E-04	HSA-373760
2	Reactome Pathways	MET activates PTK2 signaling	LAMC2|LAMB3	0.0015	HSA-8874081
2	Reactome Pathways	Smooth Muscle Contraction	ITGA1|ITGB5	0.0017	HSA-445355
4	Reactome Pathways	Hemostasis	ITGA1|ACTB|ITGA10|ITGA6	0.004	HSA-109582
3	Reactome Pathways	Signaling by Receptor Tyrosine Kinases	LAMC2|ACTB|LAMB3	0.0172	HSA-9006934
2	Reactome Pathways	Degradation of the extracellular matrix	LAMC2|LAMB3	0.0213	HSA-1474228
6	Reference publications	(2017) Nonsense variant in COL7A1 causes recessive dystrophic epidermolysis bullosa in Central Asian Shepherd dogs.	ITGB4|LAMC2|PLEC|NAT9|LAMB3|ITGA6	3.82E-10	PMID.28493971
6	Reference publications	(2015) Molecular architecture and function of the hemidesmosome.	ITGB4|LAMC2|PLEC|LAMB3|CD151|ITGA6	1.25E-09	PMID.25487405
6	Reference publications	(2015) Molecular architecture and function of the hemidesmosome.	ITGB4|LAMC2|PLEC|LAMB3|CD151|ITGA6	1.25E-09	PMID.26017636
5	Reference publications	(2015) Whole-genome sequencing identifies a homozygous deletion encompassing exons 17 to 23 of the integrin beta 4 gene in a Charolais calf with junctional epidermolysis bullosa.	ITGB4|LAMC2|PLEC|LAMB3|ITGA6	3.07E-09	PMID.25935160
6	Reference publications	(2016) Differential Expression of Extracellular Matrix and Adhesion Molecules in Fetal-Origin Amniotic Epithelial Cells of Preeclamptic Pregnancy.	ITGB4|LAMC2|ITGA1|ITGB5|LAMB3|ITGA6	3.07E-09	PMID.27218821
5	Reference publications	(2010) Oral manifestations in the epidermolysis bullosa spectrum.	ITGB4|PLEC|NAT9|LAMB3|ITGA6	9.53E-09	PMID.19945630
5	Reference publications	(2017) Expression Profile of the Integrin Receptor Subunits in the Guinea Pig Sclera.	ITGB4|ITGA1|ITGB5|ITGA10|ITGA6	8.05E-08	PMID.28094579
5	Reference publications	(2000) The LIM-only protein DRALFHL2 binds to the cytoplasmic domain of several alpha and beta integrin chains and is recruited to adhesion complexes.	ITGB4|ITGA1|ITGB5|ITGA10|ITGA6	8.30E-08	PMID.10906324
5	Reference publications	(1996) A discrete site modulates activation of I domains. Application to integrin alphaMbeta2.	ITGB4|ITGA1|ITGB5|ITGA10|ITGA6	8.30E-08	PMID.8939940
5	Reference publications	(2002) Integrin activation involves a conformational change in the alpha 1 helix of the beta subunit A-domain.	ITGB4|ITGA1|ITGB5|ITGA10|ITGA6	9.07E-08	PMID.11893752

5	Reference publications	(2010) ADAM2 interactions with mouse eggs and cell lines expressing Alfa4Alfa9 (ITGA4ITGA9) integrins: implications for integrin-based adhesion and fertilization.	ITGB4|ITGA1|ITGB5|ACTB|ITGA6	1.05E-07	PMID.21060781
4	Reference publications	(2016) Laminin-binding integrin gene copy number alterations in distinct epithelial-type cancers.	ITGB4|PLEC|SYNE3|LAMB3	1.05E-07	PMID.27158381
5	Reference publications	(2013) The changing integrin expression and a role for integrin Beta8 in the chondrogenic differentiation of mesenchymal stem cells.	ITGB4|ITGA1|ITGB5|ITGA10|ITGA6	1.08E-07	PMID.24312400
5	Reference publications	(2018) Update on Genetic Conditions Affecting the Skin and the Kidneys.	ITGB4|LAMC2|PLEC|LAMB3|CD151	1.15E-07	PMID.29552546
4	Reference publications	(2015) Dermal eosinophilic infiltrate in junctional epidermolysis bullosa.	ITGB4|LAMC2|LAMB3|ITGA6	1.82E-07	PMID.25950805
6	Reference publications	(2009) Inhibitor of DASH proteases affects expression of adhesion molecules in osteoclasts and reduces myeloma growth and bone disease.	ITGB4|ITGA1|ITGB5|ACTB|LAMB3|ITGA6	1.87E-07	PMID.19388929
4	Reference publications	(2010) Epidermolysis bullosa with pyloric atresia.	ITGB4|PLEC|NAT9|ITGA6	2.24E-07	PMID.19945615
5	Reference publications	(2016) Colony-stimulating factor (CSF) 1 receptor blockade reduces inflammation in human and murine models of rheumatoid arthritis.	ITGB4|ITGA1|ITGB5|LAMB3|ITGA6	2.24E-07	PMID.27036883
5	Reference publications	(2002) Distribution and evolution of von Willebrandintegrin A domains: widely dispersed domains with roles in cell adhesion and elsewhere.	ITGB4|ITGA1|ITGB5|ITGA10|ITGA6	2.51E-07	PMID.12388743
5	Reference publications	(2012) Astroglial integrins in the development and regulation of neurovascular units.	ITGB4|ITGA1|ITGB5|ITGA10|ITGA6	2.51E-07	PMID.23304493
4	Reference publications	(2014) Molecular identification of collagen 17a1 as a major genetic modifier of laminin gamma 2 mutation-induced junctional epidermolysis bullosa in mice.	ITGB4|LAMC2|LAMB3|ITGA6	4.77E-07	PMID.24550734
4	Reference publications	(2013) In vitro analysis of photosensitizer accumulation for assessment of applicability of fluorescence diagnosis of squamous cell carcinoma of epidermolysis bullosa patients.	LAMC2|PLEC|NAT9|LAMB3	1.49E-06	PMID.23509735
4	Reference publications	(2017) Significant Role of Collagen XVII And Integrin Beta4 in Migration and Invasion of The Less Aggressive Squamous Cell Carcinoma Cells.	ITGB4|PLEC|ACTB|CD151	1.49E-06	PMID.28327550
4	Reference publications	(2010) Integrin expression profiling identifies integrin alpha5 and beta1 as prognostic factors in early stage non-small cell lung cancer.	ITGB4|ITGA1|ITGB5|ACTB	2.03E-06	PMID.20565758
5	Reference publications	(2012) Discovery of platyhelminth-specific AlfaBeta-integrin families and evidence for their role in reproduction in Schistosoma mansoni.	ITGB4|ITGA1|ITGB5|ITGA10|ITGA6	2.64E-06	PMID.23300694
4	Reference publications	(2013) ITGA6 gene silencing by RNA interference modulates the expression of a large number of cell migration-related genes in human thymic epithelial cells.	ITGB4|ITGA1|ACTB|ITGA6	3.15E-06	PMID.24564203
4	Reference publications	(2018) Inhibition of integrin Beta1-mediated oncogenic signalling by the antitumor microRNA-29 family in head and neck squamous cell carcinoma.	ITGB4|LAMC2|LAMB3|ITGA6	3.15E-06	PMID.29423074
4	Reference publications	(2016) Effects of Plectin Depletion on Keratin Network Dynamics and Organization.	ITGB4|PLEC|SYNE3|CD151	4.01E-06	PMID.27007410
4	Reference publications	(2011) miR-31 is a broad regulator of Beta1-integrin expression and function in cancer cells.	ITGA1|ITGB5|ITGA10|ITGA6	4.49E-06	PMID.21875932
4	Reference publications	(2014) Integrin Alfa3Beta1 can function to promote spontaneous metastasis and lung colonization of invasive breast carcinoma.	ITGB4|LAMC2|LAMB3|ITGA6	4.49E-06	PMID.24002891
4	Reference publications	(2014) Derivation and osmotolerance characterization of three immortalized tilapia (Oreochromis mossambicus) cell lines.	ITGB4|LAMC2|LAMB3|ITGA6	4.49E-06	PMID.24797371
4	Reference publications	(2015) Novel ITGB6 mutation in autosomal recessive amelogenesis imperfecta.	ITGB4|LAMC2|LAMB3|ITGA6	4.69E-06	PMID.25431241
4	Reference publications	(2014) Transforming growth factor-beta (TGF- Beta) signaling in paravertebral muscles in juvenile and adolescent idiopathic scoliosis.	ITGB4|ITGB5|ACTB|ITGA6	5.22E-06	PMID.25313366
3	Reference publications	(2005) Plectin gene mutations can cause epidermolysis bullosa with pyloric atresia.	ITGB4|PLEC|ITGA6	5.78E-06	PMID.15654962
3	Reference publications	(2008) Desquamative enteropathy and pyloric atresia without skin disease caused by a novel intracellular beta4 integrin mutation.	ITGB4|PLEC|ITGA6	5.78E-06	PMID.18955862
3	Reference publications	(2011) Nesprin-3 augments peripheral nuclear localization of intermediate filaments in zebrafish.	ITGB4|PLEC|SYNE3	5.78E-06	PMID.21303928
3	Reference publications	(2013) Pyloric atresia.	ITGB4|PLEC|ITGA6	5.78E-06	PMID.26023436
3	Reference publications	(2016) Identification of two rare and novel large deletions in ITGB4 gene causing epidermolysis bullosa with pyloric atresia.	ITGB4|PLEC|ITGA6	5.78E-06	PMID.26739954
4	Reference publications	(2016) Molecular Signatures of Membrane Protein Complexes Underlying Muscular Dystrophy.	ITGB4|PLEC|CD151|ITGA6	5.78E-06	PMID.27099343
4	Reference publications	(2016) Mesenchymal Stem Cells Reshape and Provoke Proliferation of Articular Chondrocytes by Paracrine Secretion.	ITGA1|ITGB5|ITGA10|ITGA6	5.78E-06	PMID.27596239

4	Reference publications	(2016) Integrin and gene network analysis reveals that ITGA5 and ITGB1 are prognostic in non-small-cell lung cancer.	ITGB4|ITGA1|ITGB5|ITGA6	6.18E-06	PMID.27143927
4	Reference publications	(2016) Laminin 521 maintains differentiation potential of mouse and human satellite cell-derived myoblasts during long-term culture expansion.	ITGB4|ITGA1|ITGB5|ITGA6	6.81E-06	PMID.27964750
3	Reference publications	(1999) Integrin alpha6beta1 plays a significant role in the attachment of hepatoma cells to laminin.	ITGB4|ITGA1|ITGA6	8.00E-06	PMID.10551399
3	Reference publications	(1997) A minimal region on the integrin beta4 subunit that is critical to its localization in hemidesmosomes regulates the distribution of HD1plectin in COS-7 cells.	ITGB4|PLEC|ITGA6	8.00E-06	PMID.9264458
4	Reference publications	(2017) Laminin-511 and -521-based matrices for efficient ex vivo-expansion of human limbal epithelial progenitor cells.	ITGB4|LAMC2|LAMB3|ITGA6	1.11E-05	PMID.28698551
3	Reference publications	(2001) Two different mutations in the cytoplasmic domain of the integrin beta 4 subunit in nonlethal forms of epidermolysis bullosa prevent interaction of beta 4 with plectin.	ITGB4|PLEC|ITGA6	1.20E-05	PMID.11886501
3	Reference publications	(2011) Case of epidermolysis bullosa with pyloric atresia.	PLEC|NAT9|ITGA6	1.20E-05	PMID.22028570
3	Reference publications	(2015) Association between polymorphisms of microRNA-binding sites in integrin genes and gastric cancer in Chinese Han population.	ITGB4|ITGB5|ITGA6	1.20E-05	PMID.25472585
3	Reference publications	(1995) Expression of hemidesmosomal and extracellular matrix proteins by normal and malignant human prostate tissue.	LAMC2|PLEC|ITGA6	1.20E-05	PMID.7778688
4	Reference publications	(2010) Lung squamous cell carcinoma mRNA expression subtypes are reproducible, clinically important, and correspond to normal cell types.	ITGB4|LAMC2|ITGB5|LAMB3	1.62E-05	PMID.20643781
4	Reference publications	(2015) Comparative analysis of KRAS codon 12, 13, 18, 61, and 117 mutations using human MCF10A isogenic cell lines.	ITGB4|LAMC2|LAMB3|ITGA6	1.62E-05	PMID.25705018
3	Reference publications	(2016) MYC and integrins interplay in colorectal cancer.	ITGB4|ITGA1|ITGA6	1.62E-05	PMID.27014720
4	Reference publications	(2017) Amelogenesis Imperfecta; Genes, Proteins, and Pathways.	ITGB4|LAMC2|LAMB3|ITGA6	1.62E-05	PMID.28694781
3	Reference publications	(1995) Mutations in the 180-kD bullous pemphigoid antigen (BPAG2), a hemidesmosomal transmembrane collagen (COL17A1), in generalized atrophic benign epidermolysis bullosa.	ITGB4|LAMC2|LAMB3	1.62E-05	PMID.7550320
3	Reference publications	(1996) Compound heterozygosity for a dominant glycine substitution and a recessive internal duplication mutation in the type XVII collagen gene results in junctional epidermolysis bullosa and abnormal dentition.	ITGB4|LAMC2|LAMB3	1.62E-05	PMID.8669466
3	Reference publications	(1997) Hemidesmosome assembly assessed by expression of a wild-type integrin beta 4 cDNA in junctional epidermolysis bullosa keratinocytes.	ITGB4|PLEC|ITGA6	1.62E-05	PMID.9389789
4	Reference publications	(2013) p63 attenuates epithelial to mesenchymal potential in an experimental prostate cell model.	ITGB4|LAMC2|LAMB3|ITGA6	1.70E-05	PMID.23658742
4	Reference publications	(2017) A Genome-wide Analysis of Human Pluripotent Stem Cell-Derived Endothelial Cells in 2D or 3D Culture.	ITGA1|ITGA10|LAMB3|ITGA6	1.70E-05	PMID.28343999
4	Reference publications	(2012) Genetic polymorphisms and microRNAs: new direction in molecular epidemiology of solid cancer.	ITGB4|ITGB5|LAMB3|ITGA6	1.79E-05	PMID.21692980
3	Reference publications	(1999) Mutation analysis and molecular genetics of epidermolysis bullosa.	LAMC2|PLEC|LAMB3	1.96E-05	PMID.10367729
4	Reference publications	(2013) Mechanisms of foot-and-mouth disease virus tropism inferred from differential tissue gene expression.	ITGA1|ITGB5|ITGA10|ITGA6	2.22E-05	PMID.23724025
4	Reference publications	(2014) Ameloblast transcriptome changes from secretory to maturation stages.	ITGB4|LAMC2|LAMB3|ITGA6	2.22E-05	PMID.25158176
3	Reference publications	(2008) Polymorphisms in predicted microRNA-binding sites in integrin genes and breast cancer: ITGB4 as prognostic marker.	ITGB4|ITGB5|ITGA6	2.57E-05	PMID.18550570
3	Reference publications	(2010) Observations of skin grafts derived from keratinocytes expressing selectively engineered mutant laminin-332 molecules.	ITGB4|LAMC2|ITGA6	3.37E-05	PMID.20393483
3	Reference publications	(2010) Plectin deficiency leads to both muscular dystrophy and pyloric atresia in epidermolysis bullosa simplex.	PLEC|ACTB|NAT9	3.37E-05	PMID.20665883
3	Reference publications	(2014) Reduced susceptibility to two-stage skin carcinogenesis in mice with epidermis-specific deletion of CD151.	ITGB4|CD151|ITGA6	3.37E-05	PMID.23792458
3	Reference publications	(2016) Fabrication, characterization, and biological assessment of multilayer laminin Gamma2 DNA coatings on titanium surfaces.	ITGB4|LAMC2|PLEC	3.37E-05	PMID.26996815

4	Reference publications	(2016) A rational approach for cancer stem-like cell isolation and characterization using CD44 and prominin-1(CD133) as selection markers.	ITGB4|ITGA1|ACTB|ITGA6	3.37E-05	PMID.27655682
3	Reference publications	(2016) Overexpression of Thy1 and ITGA6 is associated with invasion, metastasis and poor prognosis in human gallbladder carcinoma.	ITGB4|CD151|ITGA6	3.37E-05	PMID.28105220
5	Reference publications	(2012) Quantitative proteomics of extracellular vesicles derived from human primary and metastatic colorectal cancer cells.	ITGB4|ITGA1|ITGB5|ACTB|ITGA6	3.75E-05	PMID.24009881
3	Reference publications	(2012) Keratinocyte-targeted expression of human laminin Gamma2 rescues skin blistering and early lethality of laminin Gamma2 deficient mice.	LAMC2|PLEC|LAMB3	3.95E-05	PMID.23029085
3	Reference publications	(2015) The rod domain is not essential for the function of plectin in maintaining tissue integrity.	ITGB4|PLEC|SYNE3	3.95E-05	PMID.25971800
3	Reference publications	(2016) Novel sporadic and recurrent mutations in KRT5 and KRT14 genes in Polish epidermolysis bullosa simplex patients: further insights into epidemiology and genotype-phenotype correlation.	ITGB4|PLEC|ITGA6	3.95E-05	PMID.26432462
4	Reference publications	(2015) Extracellular Matrix can Recover the Downregulation of Adhesion Molecules after Cell Detachment and Enhance Endothelial Cell Engraftment.	ITGB4|ITGA1|ITGB5|ITGA6	4.64E-05	PMID.26039874
4	Reference publications	(2016) ITGAV and ITGA5 diversely regulate proliferation and adipogenic differentiation of human adipose derived stem cells.	ITGA1|ITGB5|ITGA10|ITGA6	4.64E-05	PMID.27363302
3	Reference publications	(2012) Boolean network inference from time series data incorporating prior biological knowledge.	ITGB4|CD151|ITGA6	4.69E-05	PMID.23134816
3	Reference publications	(2017) Integrins functioning in uterine endometrial stromal and epithelial cells in estrus.	ITGB4|ITGB5|ITGA6	5.79E-05	PMID.27998942
4	Reference publications	(2014) Pharmacological levels of Withaferin A (Withania somnifera) trigger clinically relevant anticancer effects specific to triple negative breast cancer cells.	ITGB4|ITGB5|ACTB|ITGA6	6.71E-05	PMID.24498382
3	Reference publications	(2013) Keratins mediate localization of hemidesmosomes and repress cell motility.	PLEC|NAT9|CD151	6.94E-05	PMID.22895363
3	Reference publications	(2016) MiRTargetLink--miRNAs, Genes and Interaction Networks.	ITGB4|ACTB|ITGA6	6.94E-05	PMID.27089332
3	Reference publications	(2006) Kidney failure in mice lacking the tetraspanin CD151.	ITGB4|CD151|ITGA6	8.22E-05	PMID.17015618
3	Reference publications	(2007) Expression of selected integrins and selectins in bullous pemphigoid.	ITGB4|PLEC|CD151	9.74E-05	PMID.17515951
3	Reference publications	(2016) Progress toward Treatment and Cure of Epidermolysis Bullosa: Summary of the DEBRA International Research Symposium EB2015.	PLEC|NAT9|LAMB3	9.74E-05	PMID.26802230
3	Reference publications	(1999) Targeted disruption of the LAMA3 gene in mice reveals abnormalities in survival and late stage differentiation of epithelial cells.	ITGB4|LAMC2|LAMB3	1.10E-04	PMID.10366601
3	Reference publications	(2015) Combination of X-ray crystallography, SAXS and DEER to obtain the structure of the FnIII-3,4 domains of integrin Alfa6Beta4.	ITGB4|PLEC|CD151	1.10E-04	PMID.25849406
3	Reference publications	(2015) Role of Integrin Beta4 in Lung Endothelial Cell Inflammatory Responses to Mechanical Stress.	ITGB4|ITGB5|ITGA6	1.10E-04	PMID.26572585
3	Reference publications	(2006) laminin alpha 1 gene is essential for normal lens development in zebrafish.	LAMC2|ACTB|LAMB3	1.30E-04	PMID.16522196
3	Reference publications	(2011) Zebrafish: a model system to study heritable skin diseases.	ITGB4|PLEC|ITGA6	1.30E-04	PMID.21191402
3	Reference publications	(2012) Integrin Beta1 regulates leiomyoma cytoskeletal integrity and growth.	ITGA1|ACTB|ITGA6	1.30E-04	PMID.23023061
3	Reference publications	(2015) Dynamics of Sun5 localization during spermatogenesis in wild type and Dpy19l2 knock-out mice indicates that Sun5 is not involved in acrosome attachment to the nuclear envelope.	PLEC|SYNE3|ACTB	1.30E-04	PMID.25775128
3	Reference publications	(2016) Regulation of the collagen cross-linking enzymes LOXL2 and PLOD2 by tumor-suppressive microRNA-26ab in renal cell carcinoma.	LAMC2|LAMB3|ITGA6	1.30E-04	PMID.26983694
3	Reference publications	(2008) Multiple alpha subunits of integrin are involved in cell-mediated responses of the Manduca immune system.	ITGA1|ITGA10|ITGA6	1.40E-04	PMID.17868866
3	Reference publications	(2010) A mouse model of generalized non-Herlitz junctional epidermolysis bullosa.	LAMC2|LAMB3|ITGA6	1.40E-04	PMID.20336083
3	Reference publications	(2010) The laminin binding integrin alpha6beta1 in prostate cancer perineural invasion.	LAMC2|LAMB3|ITGA6	1.40E-04	PMID.20432448
3	Reference publications	(2010) Protein interactions with the platelet integrin alpha(IIb) regulatory motif.	ITGA1|ITGA10|ITGA6	1.40E-04	PMID.20486118
3	Reference publications	(2013) Tumor suppressive microRNA-218 inhibits cancer cell migration and invasion by targeting focal adhesion pathways in cervical squamous cell carcinoma.	LAMC2|ACTB|LAMB3	1.40E-04	PMID.23483249
3	Reference publications	(2014) Integrins mediate the migration of HepG2 cells induced by low shear stress	ITGA1|ITGA10|ITGA6	1.40E-04	PMID.25039138

3	Reference publications	(2015) Biological Effects Induced by Specific Advanced Glycation End Products in the Reconstructed Skin Model of Aging.	LAMC2|ACTB|ITGA6	1.40E-04	PMID.26309782
3	Reference publications	(2017) Integrin suppresses neurogenesis and regulates brain tissue assembly in planarian regeneration.	ITGA1|ITGA10|ITGA6	1.40E-04	PMID.28126842
3	Reference publications	(2018) CTBP1 and metabolic syndrome induce an mRNA and miRNA expression profile critical for breast cancer progression and metastasis.	ITGB4|ITGA1|ACTB	1.40E-04	PMID.29568399
3	SMART Domains	Integrin alpha (beta-propellor repeats).	ITGA1|ITGA10|ITGA6	6.94E-06	SM00191
4	SMART Domains	von Willebrand factor (vWF) type A domain	ITGB4|ITGA1|ITGB5|ITGA10	6.94E-06	SM00327
2	SMART Domains	Integrin beta subunits (N-terminal portion of extracellular region)	ITGB4|ITGB5	1.00E-04	SM00187
2	SMART Domains	This is the beta tail domain of the Integrin protein. Integrins are receptors which are involved in cell-cell and cell-extracellular matrix interactions.	ITGB4|ITGB5	1.00E-04	SM01242
2	SMART Domains	Spectrin repeats	PLEC|SYNE3	0.001	SM00150
2	SMART Domains	Laminin-type epidermal growth factor-like domai	LAMC2|LAMB3	0.001	SM00180
2	SMART Domains	domain found in Plexins, Semaphorins and Integrins	ITGB4|ITGB5	0.0013	SM00423
6	UniProt Keywords	Epidermolysis bullosa	ITGB4|LAMC2|PLEC|LAMB3|CD151|ITGA6	1.54E-13	KW-0263
5	UniProt Keywords	Integrin	ITGB4|ITGA1|ITGB5|ITGA10|ITGA6	3.79E-10	KW-0401
7	UniProt Keywords	Cell adhesion	ITGB4|LAMC2|ITGA1|ITGB5|ITGA10|LAMB3|ITGA6	2.83E-07	KW-0130
2	UniProt Keywords	Laminin EGF-like domain	LAMC2|LAMB3	0.0031	KW-0424
2	UniProt Keywords	Basement membrane	LAMC2|LAMB3	0.0043	KW-0084
8	UniProt Keywords	Disulfide bond	ITGB4|LAMC2|ITGA1|ITGB5|SYNE3|ITGA10|LAMB3|ITGA6	0.0064	KW-1015
3	UniProt Keywords	Palmitate	ITGB4|CD151|ITGA6	0.0125	KW-0564
9	UniProt Keywords	Repeat	ITGB4|LAMC2|ITGA1|ITGB5|PLEC|SYNE3|ITGA10|LAMB3|ITGA6	0.0125	KW-0677
5	UniProt Keywords	Receptor	ITGB4|ITGA1|ITGB5|ITGA10|ITGA6	0.0141	KW-0675
7	UniProt Keywords	Signal	ITGB4|LAMC2|ITGA1|ITGB5|ITGA10|LAMB3|ITGA6	0.0214	KW-0732
8	UniProt Keywords	Glycoprotein	ITGB4|LAMC2|ITGA1|ITGB5|ITGA10|LAMB3|CD151|ITGA6	0.024	KW-0325
7	UniProt Keywords	Disease	ITGB4|LAMC2|PLEC|ACTB|LAMB3|CD151|ITGA6	0.0445	KW-9995
2	UniProt Keywords	Deafness	ACTB|CD151	0.0469	KW-0209

Supplemental Table 5B. Altered genes in CCM models with 3 validations enrichment data. An enrichment category was exported along with Figure 2 that detailed altered pathways involved with the identified 5 genes and 10 interactors. Information provided in the table includes the number of enriched genes in each enrichment category, description of the category, genes specifically involved, FDR value, and the term name for each category (which includes GO terms if applicable).
