# Supplementary material for: Comparative omics of CCM signaling complex (CSC)
Source: Chin Neurosurg J. 2020 Jan 15;6:4. doi: 10.1186/s41016-019-0183-6 (PMC7398211; doi:10.1186/s41016-019-0183-6)
Supplement: Supplementary file 9 — Additional file 9: Table S6A. Detailed description of altered gene in CCM models with 4 validations. 1 gene was analyzed that overlapped in four different CCM studies. Details provided for identified protein include mechanisms associated with each, functions, binding partners, motifs and domains. Protein details were extracted from STRING enrichment data after construction of Figure 5 Interactome. Protein in bold is the 1 validated protein, while other proteins are the 10 interactors added to interactome. [file 41016_2019_183_MOESM9_ESM.pdf]

Detailed description of altered gene in CCM models with 4 validations

Display name	Description
**TUBB4B**	Tubulin, beta 4B class IVb; Tubulin is the major constituent of microtubules. It binds two moles of GTP, one at an exchangeable site on the beta chain and one at a non-exchangeable site on the alpha chain; Belongs to the tubulin family.
TUBA1B	Tubulin alpha-ubiquitous chain; Tubulin is the major constituent of microtubules. It binds two moles of GTP, one at an exchangeable site on the beta chain and one at a non-exchangeable site on the alpha chain.
TUBB	Tubulin, beta class I; Tubulin is the major constituent of microtubules. It binds two moles of GTP, one at an exchangeable site on the beta chain and one at a non-exchangeable site on the alpha chain.
TUBB2A	Tubulin, beta 2A class IIa; Tubulin is the major constituent of microtubules. It binds two moles of GTP, one at an exchangeable site on the beta chain and one at a non-exchangeable site on the alpha chain (By similarity).
TUBA1A	Tubulin alpha-1A chain; Tubulin is the major constituent of microtubules. It binds two moles of GTP, one at an exchangeable site on the beta chain and one at a non-exchangeable site on the alpha chain.
TUBA4A	Testis-specific alpha-tubulin; Tubulin is the major constituent of microtubules. It binds two moles of GTP, one at an exchangeable site on the beta chain and one at a non-exchangeable site on the alpha chain.
CDC5L	Cell division cycle 5-like protein; DNA-binding protein involved in cell cycle control. May act as a transcription activator. Component of the PRP19-CDC5L complex that forms an integral part of the spliceosome and is required for activating pre-mRNA splicing. The PRP19-CDC5L complex may also play a role in the response to DNA damage (DDR).
TUBA1C	Tubulin alpha-1C chain; Tubulin is the major constituent of microtubules. It binds two moles of GTP, one at an exchangeable site on the beta chain and one at a non-exchangeable site on the alpha chain; Belongs to the tubulin family.

CKAP5	Colonic and hepatic tumor overexpressed gene protein; Binds to the plus end of microtubules and regulates microtubule dynamics and microtubule organization. Acts as processive microtubule polymerase. Promotes cytoplasmic microtubule nucleation and elongation. Plays a major role in organizing spindle poles. In spindle formation protects kinetochore microtubules from depolymerization by KIF2C and has an essential role in centrosomal microtubule assembly independently of KIF2C activity. Contributes to centrosome integrity. Acts as component of the TACC3/ch-TOG/clathrin complex proposed to contribute to stabilization of kinetochore fibers of the mitotic spindle by acting as inter-microtubule bridge. The TACC3/ch- TOG/clathrin complex is required for the maintenance of kinetochore fiber tension. Enhances the strength of NDC80 complex-mediated kinetochore-tip microtubule attachments; TOG domain containing.
TBCA	Tubulin-specific chaperone A; Tubulin-folding protein; involved in the early step of the tubulin folding pathway.
TBCD	Tubulin-specific chaperone D; Tubulin-folding protein implicated in the first step of the tubulin folding pathway and required for tubulin complex assembly. Involved in the regulation of microtubule polymerization or depolymerization, it modulates microtubule dynamics by capturing GTP-bound beta-tubulin (TUBB). Its ability to interact with beta tubulin is regulated via its interaction with ARL2. Acts as a GTPase-activating protein (GAP) for ARL2. Induces microtubule disruption in absence of ARL2. Increases degradation of beta tubulin, when overexpressed in polarized cells. Promotes epithelial cell detachment, a process antagonized by ARL2. Induces tight adherens and tight junction’s disassembly at the lateral cell membrane. Required for correct assembly and maintenance of the mitotic spindle, and proper progression of mitosis. Involved in neuron morphogenesis; Belongs to the TBCD family.

Supplemental Table 6A. Detailed description of altered gene in CCM models with 4 validations. 1 gene was analyzed that overlapped in four different CCM studies. Details provided for identified protein include mechanisms associated with each, functions, binding partners, motifs and domains. Protein details were extracted from STRING enrichment data after construction of Figure 5 Interactome. Protein in bold is the 1 validated protein, while other proteins are the 10 interactors added to interactome.
