# Supplementary material for: Comparative omics of CCM signaling complex (CSC)
Source: Chin Neurosurg J. 2020 Jan 15;6:4. doi: 10.1186/s41016-019-0183-6 (PMC7398211; doi:10.1186/s41016-019-0183-6)
Supplement: Supplementary file 10 — Additional file 10: Table S6B. Altered gene in CCM models with 4 validations enrichment data. An enrichment category was exported along with Figure 5 that detailed altered pathways involved with the identified 1 gene and 10 interactors. Information provided in the table includes the number of enriched genes in each enrichment category, description of the category, genes specifically involved, FDR value, and the term name for each category (which includes GO terms if applicable). [file 41016_2019_183_MOESM10_ESM.pdf]

Altered gene in CCM models with 4 validations enrichment data

# enriched genes	category	description	enriched genes	FDR value	term name
9	GO Component	microtubule	TUBA4A|TUBA1A|TUBA1C|TBCA|TUBA1B|TUBB|TUBB4B|TBCD|TUBB2A	2.73E-12	GO.0005874
10	GO Component	microtubule cytoskeleton	TUBA4A|TUBA1A|TUBA1C|TBCA|TUBA1B|TUBB|TUBB4B|TBCD|TUBB2A|CKAP5	1.40E-10	GO.0015630
10	GO Component	cytoskeletal part	TUBA4A|TUBA1A|TUBA1C|TBCA|TUBA1B|TUBB|TUBB4B|TBCD|TUBB2A|CKAP5	1.50E-09	GO.0044430
3	GO Component	myelin sheath	TUBA1A|TUBA1B|TUBB4B	8.00E-04	GO.0043209
11	GO Component	intracellular organelle part	TUBA4A|TUBA1A|TUBA1C|TBCA|TUBA1B|TUBB|TUBB4B|TBCD|CDC5L|TUBB2A|CKAP5	0.0015	GO.0044446
2	GO Component	cytoplasmic microtubule	TUBA1A|TUBA1B	0.0028	GO.0005881
3	GO Component	membrane raft	TUBA1A|TUBA1B|TUBB	0.0038	GO.0045121
2	GO Component	azurophil granule lumen	TUBB|TUBB4B	0.007	GO.0035578
11	GO Component	cytoplasm	TUBA4A|TUBA1A|TUBA1C|TBCA|TUBA1B|TUBB|TUBB4B|TBCD|CDC5L|TUBB2A|CKAP5	0.0123	GO.0005737
2	GO Component	cytoplasmic ribonucleoprotein granule	TUBA1A|TUBB	0.0227	GO.0036464
3	GO Component	ribonucleoprotein complex	TUBA1A|TUBB|CDC5L	0.0307	GO.1990904
7	GO Function	structural constituent of cytoskeleton	TUBA4A|TUBA1A|TUBA1C|TUBA1B|TUBB|TUBB4B|TUBB2A	4.69E-12	GO.0005200
7	GO Function	GTPase activity	TUBA4A|TUBA1A|TUBA1C|TUBA1B|TUBB|TUBB4B|TUBB2A	1.87E-09	GO.0003924
7	GO Function	GTP binding	TUBA4A|TUBA1A|TUBA1C|TUBA1B|TUBB|TUBB4B|TUBB2A	7.28E-09	GO.0005525
2	GO Function	MHC class I protein binding	TUBB|TUBB4B	2.50E-04	GO.0042288
2	GO Function	beta-tubulin binding	TBCA|TBCD	6.70E-04	GO.0048487
2	GO Function	double-stranded RNA binding	TUBA1B|TUBB4B	0.0021	GO.0003725
3	GO Function	tubulin binding	TBCA|TBCD|CKAP5	0.0023	GO.0015631
2	GO Function	chaperone binding	TBCA|TBCD	0.0039	GO.0051087
9	GO Function	protein binding	TUBA4A|TUBA1A|TBCA|TUBA1B|TUBB|TUBB4B|TBCD|CDC5L|CKAP5	0.004	GO.0005515
8	GO Function	heterocyclic compound binding	TUBA4A|TUBA1A|TUBA1C|TUBA1B|TUBB|TUBB4B|CDC5L|TUBB2A	0.0054	GO.1901363
8	GO Function	organic cyclic compound binding	TUBA4A|TUBA1A|TUBA1C|TUBA1B|TUBB|TUBB4B|CDC5L|TUBB2A	0.0058	GO.0097159
11	GO Function	binding	TUBA4A|TUBA1A|TUBA1C|TBCA|TUBA1B|TUBB|TUBB4B|TBCD|CDC5L|TUBB2A|CKAP5	0.01	GO.0005488
3	GO Function	protein domain specific binding	TUBA1A|TUBB|CDC5L	0.0147	GO.0019904
2	GO Function	ubiquitin protein ligase binding	TUBA1B|TUBB	0.0282	GO.0031625
8	GO Process	microtubule-based process	TUBA4A|TUBA1A|TUBA1C|TUBA1B|TUBB|TUBB4B|TUBB2A|CKAP5	3.89E-08	GO.0007017
5	GO Process	ciliary basal body-plasma membrane docking	TUBA4A|TUBA1A|TUBB|TUBB4B|CKAP5	2.06E-07	GO.0097711
5	GO Process	G2/M transition of mitotic cell cycle	TUBA4A|TUBA1A|TUBB|TUBB4B|CKAP5	4.58E-07	GO.0000086
8	GO Process	cytoskeleton organization	TUBA4A|TUBA1A|TUBA1C|TUBA1B|TUBB|TUBB4B|TUBB2A|CKAP5	4.58E-07	GO.0007010
7	GO Process	mitotic cell cycle	TUBA4A|TUBA1A|TUBB|TUBB4B|TBCD|CDC5L|CKAP5	5.21E-07	GO.0000278
5	GO Process	regulation of G2/M transition of mitotic cell cycle	TUBA4A|TUBA1A|TUBB|TUBB4B|CKAP5	5.21E-07	GO.0010389
4	GO Process	cytoskeleton-dependent intracellular transport	TUBA1A|TUBA1C|TUBA1B|TUBB	2.17E-05	GO.0030705
5	GO Process	cell division	TUBA1A|TUBA1C|TUBA1B|TUBB|CKAP5	5.31E-05	GO.0051301
2	GO Process	post-chaperonin tubulin folding pathway	TBCA|TBCD	5.67E-05	GO.0007023
2	GO Process	tubulin complex assembly	TBCA|TBCD	1.20E-04	GO.0007021
10	GO Process	cellular component organization	TUBA4A|TUBA1A|TUBA1C|TBCA|TUBA1B|TUBB|TUBB4B|TBCD|TUBB2A|CKAP5	1.40E-04	GO.0016043
6	GO Process	regulation of cell cycle	TUBA4A|TUBA1A|TUBB|TUBB4B|CDC5L|CKAP5	1.40E-04	GO.0051726
7	GO Process	cellular localization	TUBA4A|TUBA1A|TUBA1C|TUBA1B|TUBB|TUBB4B|CKAP5	4.10E-04	GO.0051641
7	GO Process	cellular component assembly	TUBA4A|TUBA1A|TBCA|TUBB|TUBB4B|TBCD|CKAP5	6.30E-04	GO.0022607
2	GO Process	natural killer cell mediated cytotoxicity	TUBB|TUBB4B	8.70E-04	GO.0042267
3	GO Process	microtubule cytoskeleton organization	TUBA1B|TUBB|CKAP5	0.0083	GO.0000226
2	GO Process	spindle organization	TUBB|CKAP5	0.0142	GO.0007051
7	GO Process	transport	TUBA4A|TUBA1A|TUBA1C|TUBA1B|TUBB|TUBB4B|CKAP5	0.0173	GO.0006810
3	GO Process	regulated exocytosis	TUBA4A|TUBB|TUBB4B	0.035	GO.0045055
7	InterPro Domains	Tubulin	TUBA4A|TUBA1A|TUBA1C|TUBA1B|TUBB|TUBB4B|TUBB2A	3.39E-17	IPR000217
7	InterPro Domains	Tubulin/FtsZ, GTPase domain	TUBA4A|TUBA1A|TUBA1C|TUBA1B|TUBB|TUBB4B|TUBB2A	3.39E-17	IPR003008
7	InterPro Domains	Tubulin/FtsZ, C-terminal	TUBA4A|TUBA1A|TUBA1C|TUBA1B|TUBB|TUBB4B|TUBB2A	3.39E-17	IPR008280
7	InterPro Domains	Tubulin, conserved site	TUBA4A|TUBA1A|TUBA1C|TUBA1B|TUBB|TUBB4B|TUBB2A	3.39E-17	IPR017975
7	InterPro Domains	Tubulin/FtsZ, 2-layer sandwich domain	TUBA4A|TUBA1A|TUBA1C|TUBA1B|TUBB|TUBB4B|TUBB2A	3.39E-17	IPR018316
7	InterPro Domains	Tubulin, C-terminal	TUBA4A|TUBA1A|TUBA1C|TUBA1B|TUBB|TUBB4B|TUBB2A	3.39E-17	IPR023123
7	InterPro Domains	Tubulin/FtsZ, GTPase domain superfamily	TUBA4A|TUBA1A|TUBA1C|TUBA1B|TUBB|TUBB4B|TUBB2A	3.39E-17	IPR036525
7	InterPro Domains	Tubulin/FtsZ, C-terminal domain superfamily	TUBA4A|TUBA1A|TUBA1C|TUBA1B|TUBB|TUBB4B|TUBB2A	3.39E-17	IPR037103
4	InterPro Domains	Alpha tubulin	TUBA4A|TUBA1A|TUBA1C|TUBA1B	5.62E-11	IPR002452
3	InterPro Domains	Beta tubulin, autoregulation binding site	TUBB|TUBB4B|TUBB2A	7.15E-08	IPR013838
3	InterPro Domains	Beta tubulin	TUBB|TUBB4B|TUBB2A	8.27E-08	IPR002453
2	InterPro Domains	Armadillo-like helical	TBCD|CKAP5	0.0073	IPR011989
2	InterPro Domains	Armadillo-type fold	TBCD|CKAP5	0.0212	IPR016024
7	KEGG Pathways	Pathogenic Escherichia coli infection	TUBA4A|TUBA1A|TUBA1C|TUBA1B|TUBB|TUBB4B|TUBB2A	3.47E-15	hsa05130
7	KEGG Pathways	Gap junction	TUBA4A|TUBA1A|TUBA1C|TUBA1B|TUBB|TUBB4B|TUBB2A	4.57E-14	hsa04540
7	KEGG Pathways	Phagosome	TUBA4A|TUBA1A|TUBA1C|TUBA1B|TUBB|TUBB4B|TUBB2A	9.52E-13	hsa04145
4	KEGG Pathways	Apoptosis	TUBA4A|TUBA1A|TUBA1C|TUBA1B	1.16E-06	hsa04210
4	KEGG Pathways	Tight junction	TUBA4A|TUBA1A|TUBA1C|TUBA1B	2.12E-06	hsa04530
7	Pfam	Tubulin/FtsZ family, GTPase domain	TUBA4A|TUBA1A|TUBA1C|TUBA1B|TUBB|TUBB4B|TUBB2A	1.84E-17	PF00091
7	Pfam	Tubulin C-terminal domain	TUBA4A|TUBA1A|TUBA1C|TUBA1B|TUBB|TUBB4B|TUBB2A	1.84E-17	PF03953
8	Reactome Pathways	Post-chaperonin tubulin folding pathway	TUBA4A|TUBA1A|TUBA1C|TBCA|TUBA1B|TUBB4B|TBCD|TUBB2A	7.66E-20	HSA-389977
8	Reactome Pathways	Recruitment of NuMA to mitotic centrosomes	TUBA4A|TUBA1A|TUBA1C|TUBA1B|TUBB|TUBB4B|TUBB2A|CKAP5	2.09E-15	HSA-380320
6	Reactome Pathways	Microtubule-dependent trafficking of connexons from Golgi to the plasma membrane	TUBA4A|TUBA1A|TUBA1C|TUBA1B|TUBB4B|TUBB2A	1.19E-14	HSA-190840
6	Reactome Pathways	Formation of tubulin folding intermediates by CCT/TriC	TUBA4A|TUBA1A|TUBA1C|TUBA1B|TUBB4B|TUBB2A	3.73E-14	HSA-389960
6	Reactome Pathways	RHO GTPases activate IQGAPs	TUBA4A|TUBA1A|TUBA1C|TUBA1B|TUBB4B|TUBB2A	1.09E-13	HSA-5626467
8	Reactome Pathways	Cilium Assembly	TUBA4A|TUBA1A|TUBA1C|TUBA1B|TUBB|TUBB4B|TUBB2A|CKAP5	1.30E-13	HSA-5617833
8	Reactome Pathways	G2/M Transition	TUBA4A|TUBA1A|TUBA1C|TUBA1B|TUBB|TUBB4B|TUBB2A|CKAP5	1.30E-13	HSA-69275
6	Reactome Pathways	Carboxyterminal post-translational modifications of tubulin	TUBA4A|TUBA1A|TUBA1C|TUBA1B|TUBB4B|TUBB2A	2.73E-13	HSA-8955332
6	Reactome Pathways	Recycling pathway of L1	TUBA4A|TUBA1A|TUBA1C|TUBA1B|TUBB4B|TUBB2A	5.62E-13	HSA-437239
7	Reactome Pathways	Resolution of Sister Chromatid Cohesion	TUBA4A|TUBA1A|TUBA1C|TUBA1B|TUBB4B|TUBB2A|CKAP5	5.90E-13	HSA-2500257
6	Reactome Pathways	COPI-independent Golgi-to-ER retrograde traffic	TUBA4A|TUBA1A|TUBA1C|TUBA1B|TUBB4B|TUBB2A	7.14E-13	HSA-6811436
6	Reactome Pathways	Intraflagellar transport	TUBA4A|TUBA1A|TUBA1C|TUBA1B|TUBB4B|TUBB2A	7.60E-13	HSA-5620924
7	Reactome Pathways	RHO GTPases Activate Formins	TUBA4A|TUBA1A|TUBA1C|TUBA1B|TUBB4B|TUBB2A|CKAP5	9.11E-13	HSA-5663220
6	Reactome Pathways	HSP90 chaperone cycle for steroid hormone receptors (SHR)	TUBA4A|TUBA1A|TUBA1C|TUBA1B|TUBB4B|TUBB2A	9.58E-13	HSA-3371497
6	Reactome Pathways	Kinesins	TUBA4A|TUBA1A|TUBA1C|TUBA1B|TUBB4B|TUBB2A	1.46E-12	HSA-983189
6	Reactome Pathways	Translocation of SLC2A4 (GLUT4) to the plasma membrane	TUBA4A|TUBA1A|TUBA1C|TUBA1B|TUBB4B|TUBB2A	3.75E-12	HSA-1445148
6	Reactome Pathways	The role of GTSE1 in G2/M progression after G2 checkpoint	TUBA4A|TUBA1A|TUBA1C|TUBA1B|TUBB4B|TUBB2A	4.79E-12	HSA-8852276
7	Reactome Pathways	Separation of Sister Chromatids	TUBA4A|TUBA1A|TUBA1C|TUBA1B|TUBB4B|TUBB2A|CKAP5	5.48E-12	HSA-2467813
5	Reactome Pathways	Prefoldin mediated transfer of substrate to CCT/TriC	TUBA4A|TUBA1A|TUBA1C|TUBB4B|TUBB2A	8.40E-12	HSA-389957
6	Reactome Pathways	COPI-dependent Golgi-to-ER retrograde traffic	TUBA4A|TUBA1A|TUBA1C|TUBA1B|TUBB4B|TUBB2A	1.89E-11	HSA-6811434

6	Reactome Pathways	COPI-mediated anterograde transport	TUBA4A|TUBA1A|TUBA1C|TUBA1B|TUBB4B|TUBB2A	2.19E-11	HSA-6807878
6	Reactome Pathways	Hedgehog 'off' state	TUBA4A|TUBA1A|TUBA1C|TUBA1B|TUBB4B|TUBB2A	3.52E-11	HSA-5610787
6	Reactome Pathways	MHC class II antigen presentation	TUBA4A|TUBA1A|TUBA1C|TUBA1B|TUBB4B|TUBB2A	5.69E-11	HSA-2132295
5	Reactome Pathways	Loss of Nlp from mitotic centrosomes	TUBA4A|TUBA1A|TUBB|TUBB4B|CKAP5	5.06E-10	HSA-380259
5	Reactome Pathways	AURKA Activation by TPX2	TUBA4A|TUBA1A|TUBB|TUBB4B|CKAP5	5.83E-10	HSA-8854518
5	Reactome Pathways	Recruitment of mitotic centrosome proteins and complexes	TUBA4A|TUBA1A|TUBB|TUBB4B|CKAP5	9.15E-10	HSA-380270
5	Reactome Pathways	Regulation of PLK1 Activity at G2/M Transition	TUBA4A|TUBA1A|TUBB|TUBB4B|CKAP5	1.25E-09	HSA-2565942
5	Reactome Pathways	Anchoring of the basal body to the plasma membrane	TUBA4A|TUBA1A|TUBB|TUBB4B|CKAP5	2.21E-09	HSA-5620912
7	Reactome Pathways	Immune System	TUBA4A|TUBA1A|TUBA1C|TUBA1B|TUBB|TUBB4B|TUBB2A	2.56E-05	HSA-168256
2	Reactome Pathways	Neutrophil degranulation	TUBB|TUBB4B	0.0326	HSA-6798695
6	Reference publications	(2014) A direct interaction between leucine-rich repeat kinase 2 and specific Beta-tubulin isoforms regulates tubulin acetylation.	TUBA4A|TUBA1A|TUBA1B|TUBB|TUBB4B|TUBB2A	4.22E-12	PMID.24275654
7	Reference publications	(2015) Branchial cilia and sperm flagella recruit distinct axonemal components.	TUBA4A|TUBA1A|TUBA1C|TUBA1B|TUBB|TUBB4B|TUBB2A	4.22E-12	PMID.25962172
5	Reference publications	(2014) Gene expression of peripheral blood cells reveals pathways downstream of glucocorticoid receptor antagonism and nab-paclitaxel treatment.	TUBA4A|TUBA1A|TUBA1B|TUBB|TUBB4B	1.13E-10	PMID.25000515
5	Reference publications	(2014) Gene expression profiling reveals epithelial mesenchymal transition (EMT) genes can selectively differentiate eribulin sensitive breast cancer cells.	TUBA4A|TUBA1B|TUBB|TUBB4B|TUBB2A	9.72E-09	PMID.25171249
5	Reference publications	(2010) Environmentally relevant exposure to 17alpha-ethinylestradiol affects the telencephalic proteome of male fathead minnows.	TUBA4A|TUBA1A|TUBB|TUBB4B|TUBB2A	2.65E-08	PMID.20381887
5	Reference publications	(2014) A snapshot of the hepatic transcriptome: ad libitum alcohol intake suppresses expression of cholesterol synthesis genes in alcohol-preferring (P) rats.	TUBA4A|TUBA1C|TUBB|TUBB4B|TUBB2A	4.07E-08	PMID.25542004
4	Reference publications	(2016) Mutations in TUBB8 and Human Oocyte Meiotic Arrest.	TBCA|TUBB4B|TBCD|TUBB2A	4.25E-08	PMID.26789871
4	Reference publications	(2013) Specific in vivo labeling of tyrosinated Alfa-tubulin and measurement of microtubule dynamics using a GFP tagged, cytoplasmically expressed recombinant antibody.	TUBA1A|TUBB|TUBB2A|CKAP5	4.95E-08	PMID.23555790
5	Reference publications	(2015) Modulation of macrophage activities in proliferation, lysosome, and phagosome by the nonspecific immunostimulator, mica.	TUBA1A|TUBA1B|TUBB|TUBB4B|TUBB2A	4.95E-08	PMID.25668030
4	Reference publications	(2016) Novel Alfa-tubulin mutation disrupts neural development and tubulin proteostasis.	TUBA1A|TUBA1C|TBCA|TBCD	6.66E-08	PMID.26658218
4	Reference publications	(2016) Reversal of axonal growth defects in an extraocular fibrosis model by engineering the kinesin-microtubule interface.	TUBA1A|TUBA1B|TUBB4B|TUBB2A	6.66E-08	PMID.26775887
4	Reference publications	(2013) Structural basis of tubulin tyrosination by tubulin tyrosine ligase.	TUBA1A|TUBA1C|TUBB4B|TUBB2A	7.03E-08	PMID.23358242
4	Reference publications	(2014) Mutations in tubulin genes are frequent causes of various foetal malformations of cortical development including microlissencephaly.	TUBA1A|TUBB|TUBB4B|TUBB2A	8.10E-08	PMID.25059107
4	Reference publications	(2012) Mutations in the Beta-tubulin gene TUBB5 cause microcephaly with structural brain abnormalities.	TUBA1A|TBCA|TUBB|TBCD	1.14E-07	PMID.23246003
4	Reference publications	(2017) The Immunosuppressant Mycophenolic Acid Alters Nucleotide and Lipid Metabolism in an Intestinal Cell Model.	TUBA4A|TUBA1C|TUBB|TUBB2A	1.83E-07	PMID.28327659
5	Reference publications	(2015) Plumbagin suppresses epithelial to mesenchymal transition and stemness via inhibiting Nrf2-mediated signaling pathway in human tongue squamous cell carcinoma cells.	TUBA1C|TUBA1B|TUBB|TUBB4B|TUBB2A	1.98E-07	PMID.26491260
5	Reference publications	(2012) A proteomic view at T cell costimulation.	TUBA4A|TUBA1A|TUBA1C|TUBA1B|TUBB4B	2.09E-07	PMID.22539942
4	Reference publications	(2010) Defective adult oligodendrocyte and Schwann cell development, pigment pattern, and craniofacial morphology in puma mutant zebrafish having an alpha tubulin mutation.	TUBA4A|TUBA1A|TUBA1C|TUBA1B	5.58E-07	PMID.20692250
4	Reference publications	(2016) The cytoskeletal arrangements necessary to neurogenesis.	TUBA4A|TUBA1A|TBCA|TUBB2A	1.15E-06	PMID.26760504
4	Reference publications	(2012) The Cytotoxicity Mechanism of 6-Shogaol-Treated HeLa Human Cervical Cancer Cells Revealed by Label-Free Shotgun Proteomics and Bioinformatics Analysis.	TUBA4A|TUBB|TUBB4B|TUBB2A	1.33E-06	PMID.23243437
3	Reference publications	(2013) Prospective nested case-control study of feature genes related to leukemic evolution of myelodysplastic syndrome.	TUBB|TUBB4B|TUBB2A	3.05E-06	PMID.23065273
3	Reference publications	(2013) Determination of the optimal tubulin isotype target as a method for the development of individualized cancer chemotherapy.	TUBB|TUBB4B|TUBB2A	3.05E-06	PMID.23634782
4	Reference publications	(2018) Defining the Akt1 interactome and its role in regulating the cell cycle.	TUBA1B|TUBB|TUBB4B|TUBB2A	3.44E-06	PMID.29358593
5	Reference publications	(2015) Proteomic Profiling of Hematopoietic StemProgenitor Cells after a Whole Body Exposure of CBACaJ Mice to Titanium (48Ti) Ions.	TUBA1C|TUBA1B|TUBB|TUBB4B|TUBB2A	4.14E-06	PMID.28248266
3	Reference publications	(2012) Computational predictions of volatile anesthetic interactions with the microtubule cytoskeleton: implications for side effects of general anesthesia.	TUBA4A|TUBA1A|TUBB4B	4.69E-06	PMID.22761654
3	Reference publications	(2018) Interaction of microtubule depolymerizing agent indanocine with different human AlfaBeta tubulin isotypes.	TUBB|TUBB4B|TUBB2A	4.69E-06	PMID.29584771
4	Reference publications	(2014) Liver transcriptome analysis in gilthead sea bream upon exposure to low temperature.	TUBA4A|TUBA1C|TUBB|TUBB2A	5.38E-06	PMID.25194679
3	Reference publications	(2010) Comparative proteomics analysis of human osteosarcomas and benign tumor of bone.	TUBA1C|TUBB|TUBB2A	5.44E-06	PMID.20362224
3	Reference publications	(2013) Altered TUBB3 expression contributes to the epothilone response of mitotic cells.	TUBB|TUBB4B|TUBB2A	6.69E-06	PMID.23321512
3	Reference publications	(2016) Divergent microtubule assembly rates after short- versus long-term loss of end-modulating kinesins.	TUBB|TUBB2A|CKAP5	6.69E-06	PMID.26912793
5	Reference publications	(2015) Plumbagin elicits differential proteomic responses mainly involving cell cycle, apoptosis, autophagy, and epithelial-to-mesenchymal transition pathways in human prostate cancer PC-3 and DU145 cells.	TUBA1A|TUBA1C|TUBB|TUBB4B|TUBB2A	7.39E-06	PMID.25609920
3	Reference publications	(2011) Development of single blastomeres derived from two-cell embryos produced in vitro in pigs.	TUBA4A|TUBB|TUBB2A	7.57E-06	PMID.21396700
3	Reference publications	(2013) Comparing the Chemical Structure and Protein Content of ChEMBL, DrugBank, Human Metabolome Database and the Therapeutic Target Database.	TUBA1A|TUBB|TUBB4B	7.57E-06	PMID.24533037
3	Reference publications	(2002) Mutation analysis of 12 candidate genes for distal hereditary motor neuropathy type II (distal HMN II) linked to 12q24.3.	TUBA1A|TUBA1C|TUBA1B	8.77E-06	PMID.12090300
3	Reference publications	(2017) Stability and function of a putative microtubule-organizing center in the human parasite Toxoplasma gondii.	TUBA4A|TUBB4B|TUBB2A	8.77E-06	PMID.28331073
3	Reference publications	(2012) Differential expression of fourteen proteins between uveal melanoma from patients who subsequently developed distant metastases versus those who did Not.	TUBA1B|TUBB|TUBB2A	1.01E-05	PMID.22570344
3	Reference publications	(2017) TUBB2B Mutation in an Adult Patient with Myoclonus-Dystonia.	TUBA4A|TUBA1A|TUBB2A	1.01E-05	PMID.28966590
3	Reference publications	(2012) Parallel evolution under chemotherapy pressure in 29 breast cancer cell lines results in dissimilar mechanisms of resistance.	TUBA1C|TUBB4B|TUBB2A	1.14E-05	PMID.22319589
3	Reference publications	(2017) A tubulin alpha 8 mouse knockout model indicates a likely role in spermatogenesis but not in brain development.	TUBA1A|TUBB|TUBB4B	1.14E-05	PMID.28388629
3	Reference publications	(2017) Distinct effects of tubulin isotype mutations on neurite growth in Caenorhabditis elegans.	TUBA4A|TUBA1A|TUBB2A	1.14E-05	PMID.28835377
3	Reference publications	(2013) Human oral isolate Lactobacillus fermentum AGR1487 reduces intestinal barrier integrity by increasing the turnover of microtubules in Caco-2 cells.	TUBA4A|TUBA1B|TUBB	1.26E-05	PMID.24244356
3	Reference publications	(2017) Modeling Treatment Response for Lamin AC Related Dilated Cardiomyopathy in Human Induced Pluripotent Stem Cells.	TUBA4A|TUBB|TUBB2A	1.26E-05	PMID.28754655
3	Reference publications	(2018) Analysis of 17 genes detects mutations in 81% of 811 patients with lissencephaly.	TUBA1A|TUBB|TUBB2A	1.26E-05	PMID.29671837
3	Reference publications	(2012) Comparison of protein expression profiles of different stages of lymph nodes metastasis in breast cancer.	TUBA4A|TUBB|TUBB2A	1.38E-05	PMID.22393307
3	Reference publications	(2012) Discovery of biomarkers for osteosarcoma by proteomics approaches.	TUBA1C|TUBB|TUBB2A	1.38E-05	PMID.23226966
3	Reference publications	(2013) Tubulin-specific chaperones: components of a molecular machine that assembles the AlfaBeta heterodimer.	TUBA1A|TBCA|TBCD	1.54E-05	PMID.23973072
3	Reference publications	(2017) UniProt: the universal protein knowledgebase.	TUBA1A|TUBA1C|TUBA1B	2.00E-05	PMID.27899622
3	Reference publications	(2014) Exome-wide rare variant analysis identifies TUBA4A mutations associated with familial ALS.	TUBA4A|TUBA1A|TUBB	2.24E-05	PMID.25374358

3	Reference publications	(2017) BRCA1 controls the cell division axis and governs ploidy and phenotype in human mammary cells.	TUBA1B|TUBB|TUBB2A	2.24E-05	PMID.28427147
3	Reference publications	(2018) Regulation of programmed-death ligand in the human head and neck squamous cell carcinoma microenvironment is mediated through matrix metalloproteinase-mediated proteolytic cleavage.	TUBA4A|TUBA1C|TUBB2A	2.24E-05	PMID.29345283
4	Reference publications	(2004) Time- and dose-dependent effects of curcumin on gene expression in human colon cancer cells.	TUBA4A|TUBA1A|CDC5L|TUBB2A	2.67E-05	PMID.15140256
3	Reference publications	(2013) The intricate relationship between microtubules and their associated motor proteins during axon growth and maintenance.	TUBB|TUBB4B|TUBB2A	3.34E-05	PMID.24010872
3	Reference publications	(2013) A categorical network approach for discovering differentially expressed regulations in cancer.	TUBA1B|TUBB|TUBB2A	3.34E-05	PMID.24565081
3	Reference publications	(2015) BetaIII-Tubulin Regulates Breast Cancer Metastases to the Brain.	TUBB|TUBB4B|TUBB2A	3.99E-05	PMID.25724666
3	Reference publications	(2015) Characterisation of mutations of the phosphoinositide-3-kinase regulatory subunit, PIK3R2, in perisylvian polymicrogyria: a next-generation sequencing study.	TUBA1A|TUBB|TUBB2A	4.33E-05	PMID.26520804
3	Reference publications	(2017) ADAR1 restricts LINE-1 retrotransposition.	TUBB|TUBB4B|TUBB2A	4.33E-05	PMID.27658966
3	Reference publications	(2012) Mining functional subgraphs from cancer protein-protein interaction networks.	TUBA4A|TUBA1A|TUBA1B	5.56E-05	PMID.23282132
3	Reference publications	(2013) SASD: the Synthetic Alternative Splicing Database for identifying novel isoform from proteomics.	TUBA4A|TUBA1C|TUBA1B	5.97E-05	PMID.24267658
4	Reference publications	(2009) Independent component analysis of Alzheimer's DNA microarray gene expression data.	TUBA1C|TUBA1B|TUBB4B|TUBB2A	6.64E-05	PMID.19173745
3	Reference publications	(2018) Dissection of affinity captured LINE-1 macromolecular complexes.	TUBB|TUBB4B|TUBB2A	6.85E-05	PMID.29309035
3	Reference publications	(2014) PTHGRN: unraveling post-translational hierarchical gene regulatory networks using PPI, ChIP-seq and gene expression data.	TUBA4A|TUBB|TUBB2A	7.31E-05	PMID.24875471
3	Reference publications	(2015) An enhanced in vivo stable isotope labeling by amino acids in cell culture (SILAC) model for quantification of drug metabolism enzymes.	TUBA4A|TUBB4B|TUBB2A	7.31E-05	PMID.25561501
3	Reference publications	(2017) Co-expression network analysis identified six hub genes in association with progression and prognosis in human clear cell renal cell carcinoma (ccRCC).	TUBA1B|TUBB|TUBB2A	7.31E-05	PMID.29159069
3	Reference publications	(2014) Human Tra2 proteins jointly control a CHEK1 splicing switch among alternative and constitutive target exons.	TUBB|CDC5L|TUBB2A	7.55E-05	PMID.25208576
3	Reference publications	(2017) Gender-Specific Expression of Ubiquitin-Specific Peptidase 9 Modulates Tau Expression and Phosphorylation: Possible Implications for Tauopathies.	TUBA1C|TUBA1B|TUBB2A	7.55E-05	PMID.27878758
3	Reference publications	(2015) Everolimus Stabilizes Podocyte Microtubules via Enhancing TUBB2B and DCDC2 Expression.	TUBA1A|TUBB|TUBB2A	7.91E-05	PMID.26331477
3	Reference publications	(2016) A systematic High-Content Screening microscopy approach reveals key roles for Rab33b, OATL1 and Myo6 in nanoparticle trafficking in HeLa cells.	TUBA1B|TUBB|TUBB2A	7.91E-05	PMID.27374232
3	Reference publications	(2009) Expression profiling of human genetic and protein interaction networks in type 1 diabetes.	TUBB|TUBB4B|TUBB2A	9.58E-05	PMID.19609442
2	Reference publications	(2001) Genetic analysis of the beta-tubulin gene, TUBB, in non-small-cell lung cancer.	TUBB|TUBB2A	1.00E-04	PMID.11752014
2	Reference publications	(2002) The Tubingen questionnaire of treatment satisfaction	TUBB|TUBB2A	1.00E-04	PMID.11894188
2	Reference publications	(2002) Re: Genetic analysis of the beta-tubulin gene, TUBB, in non-small-cell lung cancer.	TUBB|TUBB2A	1.00E-04	PMID.12011232
2	Reference publications	(2002) Re: genetic analysis of the beta-tubulin gene, TUBB, in non-small-cell lung cancer.	TUBB|TUBB2A	1.00E-04	PMID.12011233
2	Reference publications	(2004) Diocesan licensing and medical practitioners in south-west England, 1660-1780.	TUBB|TUBB2A	1.00E-04	PMID.14968645
2	Reference publications	(2004) Tubulins in Aspergillus nidulans.	TUBB|TUBB2A	1.00E-04	PMID.14998525
2	Reference publications	(2005) Phase 1 study of ABT-751, a novel microtubule inhibitor, in patients with refractory hematologic malignancies.	TUBB|TUBB2A	1.00E-04	PMID.16166440
2	Reference publications	(2006) Ki-67 protein is associated with ribosomal RNA transcription in quiescent and proliferating cells.	TUBB|TUBB2A	1.00E-04	PMID.16206250
2	Reference publications	(1991) Two alpha-tubulin genes of Aspergillus nidulans encode divergent proteins.	TUBB|TUBB2A	1.00E-04	PMID.1672037
2	Reference publications	(2005) New choke diseases and their molecular phylogenetic analysis in Agropyron ciliare var. minus and Agropyron tsukushiense var. transiens.	TUBB|TUBB2A	1.00E-04	PMID.16722220
2	Reference publications	(2009) A new stromata-producing Neotyphodium species symbiotic with clonal grass Calamagrostis epigeios (L.) Roth. grown in China.	TUBB|TUBB2A	1.00E-04	PMID.19397192
2	Reference publications	(2009) Taxonomy of Neotyphodium endophytes of Chinese native Roegneria plants.	TUBB|TUBB2A	1.00E-04	PMID.19397194
2	Reference publications	(2009) Phylogenetic divergence, morphological and physiological differences distinguish a new Neotyphodium endophyte species in the grass Bromus auleticus from South America.	TUBB|TUBB2A	1.00E-04	PMID.19537207
2	Reference publications	(2009) A new Neotyphodium species from Festuca parvigluma Steud. grown in China.	TUBB|TUBB2A	1.00E-04	PMID.19750947
2	Reference publications	(2011) Prevalence of an intraspecific Neotyphodium hybrid in natural populations of stout wood reed (Cinna arundinacea L.) from eastern North America.	TUBB|TUBB2A	1.00E-04	PMID.20943524
2	Reference publications	(2011) Phytolith analysis for differentiating between foxtail millet (Setaria italica) and green foxtail (Setaria viridis).	TUBB|TUBB2A	1.00E-04	PMID.21573069
2	Reference publications	(2011) A new Epichloe species with interspecific hybrid origins from Poa pratensis ssp. pratensis in Liyang, China.	TUBB|TUBB2A	1.00E-04	PMID.21659456
2	Reference publications	(2012) Genetic diversity in epichloid endophytes of Hordelymus europaeus suggests repeated host jumps and interspecific hybridizations.	TUBB|TUBB2A	1.00E-04	PMID.22269059
2	Reference publications	(2012) Epichloe canadensis, a new interspecific epichloid hybrid symbiotic with Canada wildrye (Elymus canadensis).	TUBB|TUBB2A	1.00E-04	PMID.22675049
2	Reference publications	(2012) Evaluation of candidate reference genes for normalization of quantitative RT-PCR in soybean tissues under various abiotic stress conditions.	TUBB|TUBB2A	1.00E-04	PMID.23029532
2	Reference publications	(2014) Species diversity of Epichloe symbiotic with two grasses from southern Argentinean Patagonia.	TUBB|TUBB2A	1.00E-04	PMID.24782501
2	Reference publications	(2015) Stroma-bearing endophyte and its potential horizontal transmission ability in Achnatherum sibiricum.	TUBB|TUBB2A	1.00E-04	PMID.25344262
2	Reference publications	(2015) A mutualistic endophyte alters the niche dimensions of its host plant.	TUBB|TUBB2A	1.00E-04	PMID.25603965
2	Reference publications	(2015) Light-Controlled Delivery of Monoclonal Antibodies for Targeted Photoinactivation of Ki-67.	TUBB|TUBB2A	1.00E-04	PMID.26226545
2	Reference publications	(2016) Legal Professionals' Knowledge of Eyewitness Testimony in China: A Cross-Sectional Survey.	TUBB|TUBB2A	1.00E-04	PMID.26828933
2	Reference publications	(2016) The expression of beta-tubulin gene in myelodysplastic syndrome evoluting to leukemia	TUBB|TUBB2A	1.00E-04	PMID.27143188
2	Reference publications	(2016) Alteramide B is a microtubule antagonist of inhibiting Candida albicans.	TUBB|TUBB2A	1.00E-04	PMID.27373684
2	Reference publications	(2016) Infantile neurodegenerative disorder associated with mutations in TBCD, an essential gene in the tubulin heterodimer assembly pathway.	TBCA|TBCD	1.00E-04	PMID.28158450
2	Reference publications	(2017) The Effects of Alcohol Intoxication on Accuracy and the Confidence-Accuracy Relationship in Photographic Simultaneous Line-ups.	TUBB|TUBB2A	1.00E-04	PMID.28781426
2	Reference publications	(2017) Creativity: The Stronger, Blacker Sheep behind Great Papers - A Reply to Falkenberg and Tubb.	TUBB|TUBB2A	1.00E-04	PMID.29037460
2	Reference publications	(1994) Sequences controlling transcription of the Chlamydomonas reinhardtii beta 2-tubulin gene after deflagellation and during the cell cycle.	TUBB4B|TUBB2A	1.00E-04	PMID.8035797
2	Reference publications	(1996) Resistance mechanisms in human sarcoma mutants derived by single-step exposure to paclitaxel (Taxol).	TUBA1A|TUBA1B	1.00E-04	PMID.8640766
7	SMART Domains	Tubulin/FtsZ family, GTPase domain	TUBA4A|TUBA1A|TUBA1C|TUBA1B|TUBB|TUBB4B|TUBB2A	1.23E-17	SM00864
7	SMART Domains	Tubulin/FtsZ family, C-terminal domain	TUBA4A|TUBA1A|TUBA1C|TUBA1B|TUBB|TUBB4B|TUBB2A	1.23E-17	SM00865

8	UniProt Keywords	Microtubule	TUBA4A|TUBA1A|TUBA1C|TBCA|TUBA1B|TUBB|TUBB4B|TUBB2A	1.26E-11	KW-0493
10	UniProt Keywords	Cytoskeleton	TUBA4A|TUBA1A|TUBA1C|TBCA|TUBA1B|TUBB|TUBB4B|TBCD|TUBB2A|CKAP5	1.85E-10	KW-0206
7	UniProt Keywords	GTP-binding	TUBA4A|TUBA1A|TUBA1C|TUBA1B|TUBB|TUBB4B|TUBB2A	2.37E-09	KW-0342
4	UniProt Keywords	Nitration	TUBA4A|TUBA1A|TUBA1C|TUBA1B	1.53E-07	KW-0944
11	UniProt Keywords	Cytoplasm	TUBA4A|TUBA1A|TUBA1C|TBCA|TUBA1B|TUBB|TUBB4B|TBCD|CDC5L|TUBB2A|CKAP5	2.65E-06	KW-0963
9	UniProt Keywords	Acetylation	TUBA4A|TUBA1A|TUBA1C|TBCA|TUBA1B|TUBB|TUBB4B|TUBB2A|CKAP5	3.71E-05	KW-0007
6	UniProt Keywords	Methylation	TUBA4A|TUBA1A|TUBA1C|TUBA1B|TUBB|TUBB2A	3.71E-05	KW-0488
6	UniProt Keywords	Isopeptide bond	TUBA1A|TUBA1B|TUBB|TUBB4B|CDC5L|TUBB2A	7.30E-04	KW-1017
2	UniProt Keywords	Chaperone	TBCA|TBCD	0.0242	KW-0143
9	UniProt Keywords	Phosphoprotein	TUBA4A|TUBA1A|TUBA1C|TUBA1B|TUBB|TUBB4B|CDC5L|TUBB2A|CKAP5	0.0315	KW-0597
6	UniProt Keywords	Disease	TUBA4A|TUBA1A|TUBB|TUBB4B|TBCD|TUBB2A	0.0386	KW-9995

Supplemental Table 6B. Altered gene in CCM models with 4 validations enrichment data. An enrichment category was exported along with Figure 3 that detailed altered pathways involved with the identified 1 gene and 10 interactors. Information provided in the table includes the number of enriched genes in each enrichment category, description of the category, genes specifically involved, FDR value, and the term name for each category (which includes GO terms if applicable).
