# Supplementary material for: Comparative omics of CCM signaling complex (CSC)
Source: Chin Neurosurg J. 2020 Jan 15;6:4. doi: 10.1186/s41016-019-0183-6 (PMC7398211; doi:10.1186/s41016-019-0183-6)
Supplement: Supplementary file 11 — Additional file 11: Table S7. 9 CCM studies used for comparative omics analysis. A list of 9 CCM studies used for the comparative omics analysis are detailed. Corresponding numbers used for each reference are used in supplemental tables 1A, 1B and 2 and in Fig. 1. [file 41016_2019_183_MOESM11_ESM.pdf]

9 CCM studies used for comparative omics analysis

1. Zhang, J., et al., *Novel functions of CCM1 delimit the relationship of PTB/PH domains.* Biochim Biophys Acta Proteins Proteom, 2017. **1865**(10): p. 1274-1286.

2. Koskimaki, J., et al., *Comprehensive transcriptome analysis of cerebral cavernous malformation across multiple species and genotypes.* JCI Insight, 2019. **4**(3).

3. Cianfruglia, L., et al., *KRIT1 Loss-Of-Function Associated with Cerebral Cavernous Malformation Disease Leads to Enhanced S-Glutathionylation of Distinct Structural and Regulatory Proteins.* Antioxidants (Basel), 2019. **8**(1).

4. Chernaya, O., et al., *Biomechanics of Endothelial Tubule Formation Differentially Modulated by Cerebral Cavernous Malformation Proteins.* iScience, 2018. **9**: p. 347-358.

5. Otten, C., et al., *Systematic pharmacological screens uncover novel pathways involved in cerebral cavernous malformations.* EMBO Mol Med, 2018. **10**(10).

6. Lant, B., et al., *Interrogating the ccm-3 Gene Network.* Cell Rep, 2018. **24**(11): p. 2857-2868 e4.

7. Edelmann, A.R., et al., *Systems biology and proteomic analysis of cerebral cavernous malformation.* Expert Rev Proteomics, 2014. **11**(3): p. 395-404.

8. Hilder, T.L., et al., *Proteomic identification of the cerebral cavernous malformation signaling complex.* J Proteome Res, 2007. **6**(11): p. 4343-55.

9. Abou-Fadel, J., et al., *Systems-wide analysis unravels the new roles of CCM signal complex (CSC).* Heliyon, 2019. **5**(12): p. e02899.

Supplemental Table 7. 9 CCM studies used for comparative omics analysis. A list of 9 CCM studies used for the comparative omics analysis are detailed. Corresponding numbers used for each reference are used in supplemental tables 1A, 1B and 2 and in figure 1.
